# Transcriptome Analysis Identifies Novel Mechanisms Associated with the Antitumor Effect of Chitosan-Stabilized Selenium Nanoparticles

**DOI:** 10.3390/pharmaceutics13030356

**Published:** 2021-03-08

**Authors:** Hector Estevez, Estefania Garcia-Calvo, Jose Rivera-Torres, María Vallet-Regí, Blanca González, Jose L. Luque-Garcia

**Affiliations:** 1 Department of Analytical Chemistry, Faculty of Chemical Sciences, Complutense University of Madrid, 28040 Madrid, Spain; hestevez@ucm.es (H.E.); egcalvo@ucm.es (E.G.-C.); 2 Department of Pharmacy and Biotechnology, School of Biomedical and Health Sciences, European University of Madrid, 28670 Madrid, Spain; jose.rivera@universidadeuropea.es; 3 Department of Chemistry in Pharmaceutical Sciences, Faculty of Pharmacy, Instituto de Investigación Sanitaria Hospital 12 de Octubre (i+12), Complutense University of Madrid, 28040 Madrid, Spain; vallet@ucm.es (M.V.-R.); blancaortiz@ucm.es (B.G.); 4 Centro de Investigación Biomédica en Red de Bioingeniería, Biomateriales y Nanomedicina (CIBER-BBN), 28029 Madrid, Spain

**Keywords:** selenium nanoparticles, transcriptome analysis, senescence, antioxidant capacity, tumor suppressors, antitumor agent

## Abstract

Selenium nanoparticles (SeNPs) have been receiving special attention in recent years due to their antioxidant capacity and antitumor properties. However, the mechanisms associated with these properties remain to be elucidated. For this reason, a global transcriptome analysis has been designed in this work and it was carried out using human hepatocarcinoma cells and chitosan-stabilized SeNPs (Ch-SeNPs) to identify new targets and pathways related to the antitumor mechanisms associated with Ch-SeNPs. The results obtained confirm the alteration of the cell cycle and the effect of Ch-SeNPs on different tumor suppressors and other molecules involved in key mechanisms related to cancer progression. Furthermore, we demonstrated the antioxidant properties of these nanoparticles and their capacity to induce senescence, which was further confirmed through the measurement of β-galactosidase activity.

## 1. Introduction

A correct balance between ROS generation by pro-oxidants and the action of antioxidants maintains the normal cellular redox status. Loss of this balance might play a critical role in cellular signaling pathways, sometimes evolving into an uncontrolled proliferation leading to neocarcinogenesis [1,2]. ROS are generated as a defense barrier against extracellular pathogens, including bacterial and viral infections, but an excessive production is also closely related to cancer pathologies. Cancer therapy is, in the great majority of the cases, based on chemotherapy, usually in combination with radiation therapy and immunotherapy. One of the main side effects of the cytotoxic agents used in chemotherapy is the generation of ROS, which induce DNA damage or affect the DNA machinery [3]. To avoid this problem, many chemotherapeutic strategies are focused on using antioxidants to deplete tumor cells from ROS-induced survival signaling pathways, such treatments also having potential preventive functions.

The emerging field of nanotechnology in recent years has opened up a new field of study for the development of novel drugs for a wide variety of diseases [4,5] and, in particular, for the development of promising nanoparticles and nanosystems with antitumor properties [6,7]. The antitumor effect of some of these nanoparticles is based on their antioxidant properties. Such is the case of gold nanoparticles, which have been successfully tested against human breast cancer (MCF-7) cells [8].

Selenium is a well-known essential micronutrient that participates in a large number of key physiological processes. It is also considered an element with antioxidant properties and, as a matter of fact, different selenospecies have been proposed as potential antitumor agents against different types of cancer [9,10]. In addition, and although further investigations are needed, it seems that SeNPs are biocompatible when exposing non-cancer cells such as human dermal fibroblasts [11]. In our previous work, we demonstrated the unique effect of chitosan-stabilized selenium nanoparticles (Ch-SeNPs) in comparison to other organic and inorganic selenospecies to induce cell cycle arrest while preventing uncontrolled generation of ROS that would have induced apoptosis [12]. Other authors have also demonstrated the use of the cytotoxicity exerted by SeNPs as a tool against different diseases [13,14,15,16,17]. Based on the above, there is strong evidence regarding the potential use of SeNPs as a very promising therapeutic alternative. However, before bringing the use of these nanoparticles to clinical models, it is necessary to elucidate the mechanisms of action at the molecular level. Although several efforts have been carried out in this way [11,12,18], further efforts are still needed to confirm the specific targets and mechanisms responsible for the observed antitumor effects of SeNPs.

Transcriptomics represent a high-throughput screening tool that provides in-depth understanding of cellular functions and the genomic landscape of transcription, thanks to its capability to mirror post-genomic variations. Thus, it represents an essential strategy for the study of such a dynamic pathology as cancer [19,20].

Based on all of the above, we had synthesized and characterized selenium nanoparticles stabilized with chitosan (Ch-SeNPs), which were further used to treat human hepatocellular carcinoma cells (HepG2 cells). The design and application of a whole transcriptome analysis to this system provided a wide number of altered targets and pathways, thus allowing us to delve deeper into the mechanisms associated with the antitumor effect of Ch-SeNPs.

## 2. Materials and Methods

### 2.1. Synthesis and Characterization of SeNPs

Chitosan, ascorbic acid, acetic acid and sodium selenite were purchased from Sigma-Aldrich (St. Louis, MO, USA) Chitosan-stabilized SeNPs (Ch-SeNPs) were synthesized following the procedure described by Bai et al. [21]. An aqueous chitosan polysaccharide solution (0.5% *w/v*) was prepared using 0.5 M acetic acid. Then, 10 mL of this chitosan solution were mixed with 7.5 mL of ascorbic acid, 0.23 M, and 5 mL of acetic acid, 2.4 M. To the resulting solution, 0.25 mL of sodium selenite, 0.51 M, were slowly added. The observed change of the solution from colorless to red was indicative of the reaction progression and the Ch-SeNPs formation. After the synthesis, the colloidal suspension was diluted to 50 mL with distilled water, resulting in final concentrations of 200 mg/L of Se and 0.1% of chitosan. Finally, the colloidal suspension was dialyzed for two hours at room temperature at a ratio of 10 mL against 2 L of distilled water and using a 12-kDa molecular weight cut-off (MWCO) membrane.

Ch-SeNPs were characterized by transmission electron microscopy (TEM) and energy dispersive X-ray spectroscopy (EDX) with a JEOL JEM 1400 PLUS operating at 120 kV and equipped with a charge-coupled device CCD camera (KeenView Camera) (JEOL Ltd., Tokyo, Japan). Sample preparation was performed by placing one or two drops of the Ch-SeNP colloidal suspension onto carbon-coated copper grids.

Electrophoretic mobility measurements for the Ch-SeNP colloidal suspension in water were used to calculate the zeta-potential (ζ-potential) values of the nanoparticles. Measurements were performed in a Zetasizer Nano ZS (Malvem Instruments Ltd., Malvern, UK) equipped with a 633 nm red laser. For this purpose, dilutions of the initial suspension were performed if needed. Measurements were recorded by placing ca. 1 mL of the suspension in disposable DTS1070 folded capillary cells (Malvern Instruments). The hydrodynamic size of the nanoparticles was measured by dynamic light scattering (DLS) with the same Malvern instrument. Values presented are means ± SD from quintuplicated measurements.

Stability of Ch-SeNPs was determined using an Agilent HP 7700x inductively coupled plasma mass spectrometer (ICP-MS) (Agilent, Santa Clara, CA, USA).

### 2.2. Cell Culture

A hepatocellular carcinoma cell line (*Homo sapiens*) known as HepG2 cells (HEPG2, ATCC HB-8065 TM, American Type Culture Collection, Manassas, VA, USA) was selected for this study. The cells were maintained in a Dulbecco’s modified Eagle´s medium (DMEM) supplemented with 10% fetal bovine serum (FBS) and 1% penicillin/streptomycin at 37 °C and 5% CO_2_. Human breast cancer cells (MBA-MD-231) and human cervical cancer cells (HeLa) (American Type Culture Collection, Manassas, VA, USA) used to validate the senescence assay were cultured under the same conditions.

### 2.3. Cytotoxicity Assay

To evaluate the cytotoxicity induced by Ch-SeNPs, HepG2 cells were seeded into 96-well plates and incubated for 24 h. Then, the cells were exposed to ranging concentrations of Ch-SeNPs (0.1 to 5 mg/L) for 72 h. After this time, 20 μL of 3-(4,5-dimethyl-thiazol-2-yl)2,5-diphenyl tetrazolium bromide (MTT, 5 mg/mL) were added to each well and incubated for five hours at 37 °C. Then, the culture medium was removed, and 100 μL of dimethyl sulfoxide were added to dissolve the insoluble purple formazan products. Absorbance was measured at 595 nm using a microplate reader (TECAN). The final results were calculated on the basis of five replicates of the experiment.

### 2.4. Transcriptome Analysis

To evaluate potential alterations in the mRNA expression levels of HepG2 cells treated with Ch-SeNPs, a transcriptome microarray analysis was performed. The cells were seeded in culture plates for 24 h and then exposed to 1 mg/L of Ch-SeNPs for 72 h (37 °C and 5% CO_2_). Control (untreated) cells were seeded in parallel and incubated at 37 °C and 5% CO_2_ for 96 h (24 h + 72 h). After the exposure time, mRNA was extracted and purified using a commercial kit (PureLink^®^, Invitrogen, Carlsbad, CA, USA). Shortly thereafter, the cells were trypsinized and centrifuged and the supernatant was removed. The cells were lysed with a lysis buffer containing 2-mercaptoethanol. Lysates were then centrifuged and one volume of 70% ethanol was added to the samples. Seven hundred microliters of this volume were transferred to a conical tube and centrifuged. Afterwards, the samples were rinsed with a washing buffer and RNase-free water was added to the spin cartridge prior to centrifugation. The purified RNA was stored at −80 °C for further analysis. The samples were processed with a GeneChip^®^ WT PLUS Reagent Kit (Applied Biosystems, Foster City, CA, USA), hybridized with Clariom™ D Array, human (Applied Biosystems) and scanned with a GeneChip^®^ Scanner 3000 7 G (Applied Biosystems). Raw data were processed with the robust multi-array average (RMA) algorithm included in the Transcriptome Analysis Console (Applied Biosystems) for normalization and gene level analysis. For each experimental condition, three microarray experiments corresponding to three independent RNA replicates were processed and analyzed. Fold changes between experimental conditions were calculated as a quotient between the means of the gene expression signals. Statistical analysis was performed with ebayes limma included in the Transcriptome Analysis Console (Applied Biosystems). Those values with a false discovery rate (FDR) (adjusted *p*-value) ≤0.05 were considered significant.

### 2.5. Senescence Assay

A cytochemical staining kit for the observation of β-galactosidase expression (Sigma-Aldrich) was selected to evaluate whether or not Ch-SeNPs induce senescence on HepG2, MBA-MD-231 and HeLa cells. The cells were seeded and exposed to either 1 mg/L of Ch-SeNPs, 10 μM of etoposide (positive senescence control) or 50 μM of etoposide (positive apoptosis control) during 72 h at 37 °C and 5% CO_2_. After the exposure time, cell culture solutions were removed and the cells were washed with PBS. The cells were then fixed with 20% formaldehyde, 2% glutaraldehyde, 70.4 mM Na2HPO4, 14.7 mM KH2PO4, 1.37 M NaCl and 26.8 mM KCl fixing buffer for seven minutes at room temperature. After removing the fixing buffer, the cells were washed with PBS and incubated with the staining mixture. Finally, the cells were incubated at 37 °C without CO_2_ overnight and then examined using a phase contrast microscope (Motic AE31).

## 3. Results and Discussion

### 3.1. Synthesis and Characterization of Ch-SeNPs

**Scheme 1:**
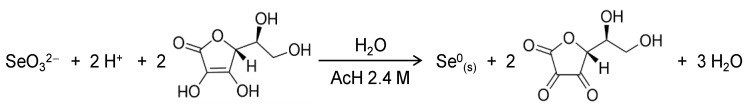
Redox reaction involved in the formation of SeNPs.

Selenium nanoparticles (SeNPs) were prepared using chemical reduction of selenite with ascorbic acid in the presence of chitosan as a soft template to control nucleation and growth of the inorganic selenium nanoparticles. Ascorbic acid acts as a reducing agent following the redox reaction (Scheme 1) in water where an acid medium is provided by acetic acid in our system [22]. The role of the polysaccharide chitosan in this redox system is to act as a stabilizer and a capping agent, affording red elemental selenium in colloidal state [23]. Furthermore, chitosan was selected because it has been shown to increase the bioavailability of SeNPs while improving the intrinsic antioxidant properties of Se [24,25].

**Figure 1:**
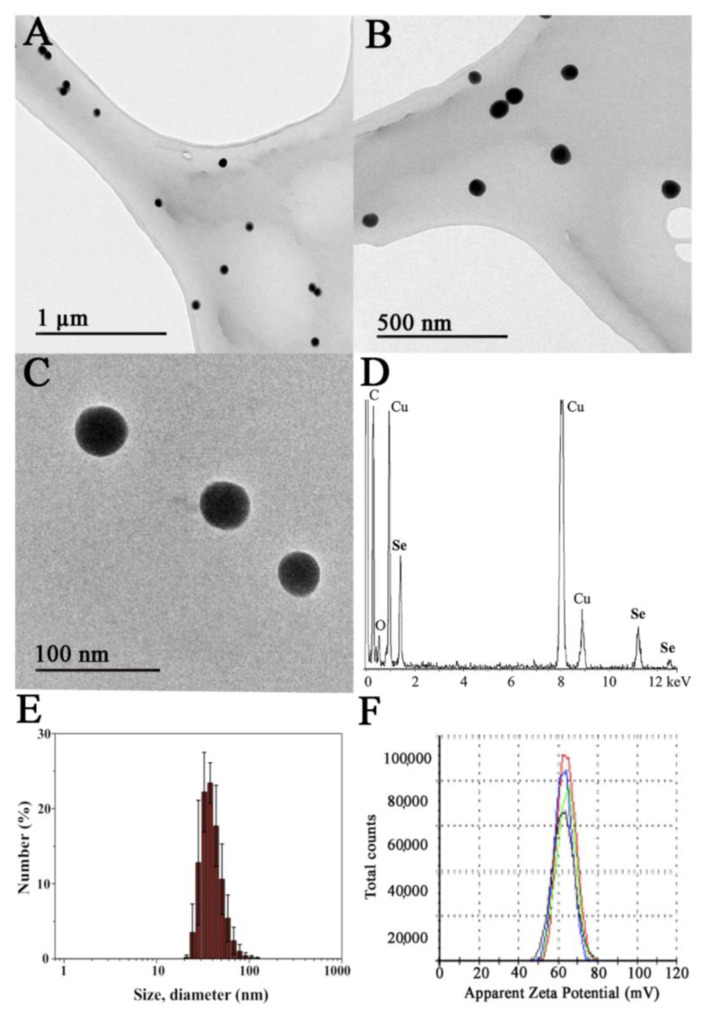
Characterization of the synthesized Ch-SeNPs. TEM images of a 200 mg/L suspension of Ch-SeNPs at different magnifications (**A**–**C**). Energy-dispersive X-ray (EDS) spectrum of Ch-SeNPs (**D**). Hydrodynamic size distribution of the Ch-SeNPs in an aqueous colloidal suspension measured by dynamic light scattering (**E**) and ζ-potential measurements of the same sample (**F**).

A final concentration of chitosan of 0.1% was employed to prepare the SeNPs since, under these conditions, the nanoparticles are colloidally stable and possess an adequate diameter range, as we described in previous works [11,26]. Well-dispersed nanoparticles were obtained as shown by transmission electron microscopy images, where Ch-SeNPs exhibit spherical morphology and homogenous sizes of around 40–60 nm (Figure 1A–C). Their composition in selenium was confirmed by energy dispersive spectroscopy analysis (Figure 1D). Signals for C and O from chitosan and the carbon-coated copper grid were also observed in the spectrum. The X-ray diffraction XRD pattern registered for Ch-SeNPs shows broad peaks and low signal-to-noise ratios that can be ascribed to small crystalline domain size in the range of few nanometres (see Figure S1 in the Supplementary Material).

The hydrodynamic diameter of the Ch-SeNPs was measured in the aqueous colloidal suspension giving a monomodal distribution in the range of ca. 25 to 100 nm, with the maximum centered at 37.8 ± 2.7 nm (Figure 1E). These values are in concordance with the size of the inorganic selenium nanoparticles observed in the TEM images.

The surface stabilization of the SeNPs with chitosan was also confirmed by means of electrophoretic mobility measurements of the Ch-SeNP colloidal suspension in water (Figure 1F). The highly positive ζ-potential value of 62.5 ± 5.2 mV falls in the zone of colloidal stability and is ascribed to the equilibrium for the protonation of the amino groups of chitosan in water. As expected, chitosan allows SeNPs to form stable colloidal suspensions due to both electrostatic as well as steric stabilization.

The synthesized Ch-SeNPs were proven to be colloidally stable, with no flocculated material observed even two months after the synthesis. No significant differences in the SeNP size or shape were observed by TEM and the hydrodynamic size distribution did not undergo a displacement of its maximum in the DLS measurements. Moreover, the possible oxidation of Ch-SeNPs to ionic selenium was also discarded. The Ch-SeNP suspension was centrifuged weekly using a 10-KDa MWCO filter and the liquid fraction was analyzed by ICP-MS. The amount of Se found in all cases was less than 0.1%, which also demonstrates good stability of the synthesized Ch-SeNPs.

### 3.2. Cytotoxicity of Ch-SeNPs

**Figure 2:**
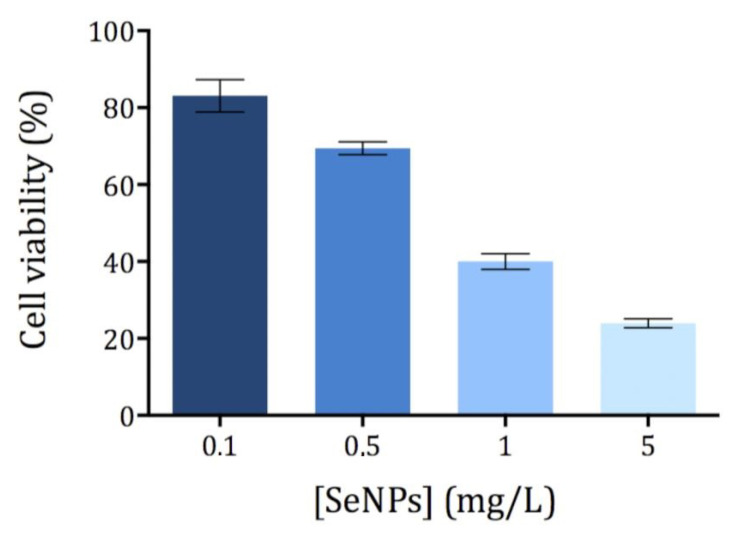
Viability of the HepG2 cells exposed to Ch-SeNPs (*n* = 5).

The cytotoxicity of Ch-SeNPs was evaluated by means of the MTT assay, which correlates the reduction potential of the cells with their viability. While healthy cells are able to reduce MTT to formazan (a colored compound), non-viable cells are not able to do so. Different concentrations of Ch-SeNPs ranging from 0.1 to 5 mg/L were tested using an exposure time of 72 h. All measurements showed a relative standard deviation below 10% (*n* = 5). The exposure time (72 h) was selected as optimal based on the previously published studies [11,17]. As expected, cell viability decreased with increasing concentrations of Ch-SeNPs (Figure 2). The cell viability of HepG2 was significantly compromised after exposure to Ch-SeNPs > 1.0 mg/L, falling below 30% at the highest concentration tested (5 mg/L). In order to investigate the molecular mechanisms involved in the potential antitumor effect exerted by Ch-SeNPs without drastically compromising the cell viability, 1 mg/L was selected for further experiments.

### 3.3. Revealing the Mechanisms Associated with the Antitumor Effect of Ch-SeNPs by Transcriptomic Analysis

**Figure 3:**
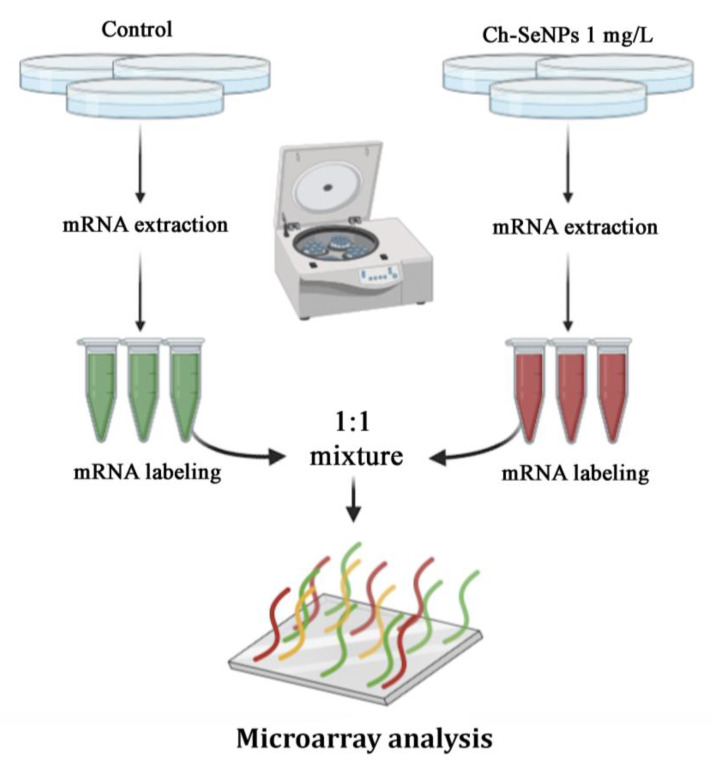
General scheme of the transcriptome analysis.

In a previous study, we confirmed the ability of Ch-SeNPs to inhibit proliferation and migration of HepG2 cells and demonstrated that Ch-SeNPs do not induce apoptosis but cell cycle arrest by partially inhibiting the expression of CDK1, which regulates the promotion of the cell cycle from the S/G2 phase to mitosis [11]. This effect was unique for Ch-SeNPs as compared to other selenospecies including Se (IV), Se (VI), SeCys2, SeMet and Se-MeSeCys [11]. Furthermore, we demonstrated that the observed effect was due exclusively to SeNPs, as the cells treated with chitosan (Ch) alone did not experience any effect compared to the control cells [17]. Since partial inhibition of CDK1 represents a way of precluding tumor cells progression, we carried out a pairwise differential gene expression analysis to compare the control cells with the cells exposed to Ch-SeNPs (Figure 3) to get a deeper insight into the molecular mechanisms underlying the potential antitumor effect of Ch-SeNPs.

Among the more than 20,000 well-annotated human genes analyzed (see Table S1 in the Supplementary Material), 279 genes were found differentially expressed with a log2 fold change of 2.0 and 0.5 (with a *p*-value < 0.05) (Table A1). From those differentially expressed genes, 221 were found upregulated (ratios above 2.0), while 58 were found downregulated (ratios below 0.5).

**Figure 4:**
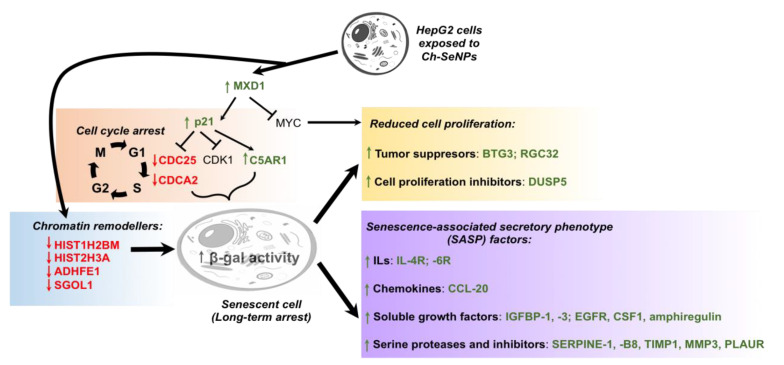
Proposed mechanism leading to cell cycle arrest and senescence in the HepG2 cells exposed to Ch-SeNPs.Since senescence plays a key role in cell cycle arrest, we investigated the pattern expression of key senescence targets after exposure to Ch-SeNPs. Senescence consists of a stable cell cycle arrest in which cells become resistant to any growth stimuli, generally in response to DNA damage. This state implies several morphological and metabolic changes, chromatin reorganization, deregulations in gene expression and even production of an inflammatory phenotype known as the senescence-associated secretory phenotype (SASP) [35]. Expression patterns for some of those genes were indeed found inhibited in the cells exposed to Ch-SeNPs; such were the cases of HIST1H2BM (FC = 0.43), HIST2H3A (FC = 0.44), ADHFE1 (FC = 0.49) and SGO1 (FC = 0.42) [36,37,38]. In addition, previous studies also demonstrated that inhibition of C5AR1 can attenuate acquisition of the senescent phenotype [39]. Remarkably, C5AR1 (FC = 5.64) was found highly overexpressed in our study. This result, together with the fact that overexpression of C5AR1 has been related to the overexpression of p21 and other genes associated with the appearance of a senescent phenotype [40] such as IL-6 (FC = 2.83) and PAI-1 (FC = 7.03), also found overexpressed after Ch-SeNPs treatment, supports the potential of Ch-SeNPs to induce senescence in HepG2 cells. Particularly interesting is the overexpression of PAI-1, also known as SERPINE 1, a serine protease inhibitor that functions as the main inhibitor of the tissue plasminogen activator (tPA) (Figure 4).

Supporting the inhibition of CDK1 found in the previous work [11], overexpression of CDKN1A (fold change (FC) = 2.67), also known as p21, was observed after Ch-SeNP exposure. This upregulation correlated with reduced expression levels of other cell cycle participant genes such as cyclin CDK1 (FC = 0.80), CDC25 (FC = 0.47) and CDCA2 (FC = 0.50) [27,28]. While CDC25 is essential for the G1–S transition associated with the activation of the cell cycle kinase cyclin E-CDK2 [27,29], knockdown of CDCA2 significantly inhibits cellular proliferation by arresting cell cycle progression at the G1 phase, effect that has also been observed when using a well-known antitumor compound such as cisplatin [30,31]. The activation of p21 upon Ch-SeNP exposure is supported by the fact that this gene, together with p15 and p27, is directly activated by MXD1, which was overexpressed in our experiment (FC = 2.42). The tumor suppressor MXD family encompasses a group of transcriptional repressors that antagonize the activation of genes mediated by oncogenic MYC [32]. Diminishing expression of angiopoietin-like 3 protein (ANGPTL3) has been correlated with reduced cellular growth and cell cycle arrest at the G1 phase [33]. Interestingly, ANGPTL3, a target downstream of p21, showed strong downregulation (FC = 0.49) upon Ch-SeNP exposure. Moreover, FAM111B, a gene, the silencing whereof induces cell cycle arrest at the G2/M phase through the p53-signaling pathway [34], was also found downregulated (FC = 0.50) in our analysis. Taken together and as expected, alteration of the genes involved in cell cycle regulation was confirmed upon Ch-SeNP exposure, thus supporting our previous studies and suggesting the cell cycle as an interesting target on which Ch-SeNPs exert their antitumor action (Figure 4).

Besides the effect of Ch-SeNPs on cell cycle arrest and their potential to induce senescence in HepG2 cells, well-known tumor suppressors were also found altered. That is the case of RASD1 (FC = 3.42), a key inhibitory player in glioma tumor progression [41]. Similarly, EGR1, which controls a network of suppressor gene products and is even considered a tumor suppressor itself [42], was also found significantly overexpressed (FC = 4.22). In addition, it has been described that EGR1 induces the expression of PAI-1 that, as stated above, was found clearly upregulated in the cells exposed to Ch-SeNPs [43]. Another tumor suppressor that was found overexpressed in the transcriptome analysis was CSTA (FC = 4.4), which is considered a potential biomarker for lung cancer and tumor differentiation. Although the role of CSTA in cancer has not yet been fully elucidated, Ma et al. [44] confirmed that CSTA exerts a tumor suppressive function by inhibiting the MAPK and the AKT pathways. CSTA has been found downregulated in several lung cancers and its restoration after chemotherapy is considered a sign of good prognosis [44]. DUSP5 (FC = 14.23), which is also considered a tumor suppressor, was highly overexpressed in cells treated with Ch-SeNPs. DUSP5 has also been related to the inhibition of the ERK2/MAPK pathways. Loss of DUSP5 expression has been detected in advanced gastric and prostate cancer; furthermore, the reexpression of DUSP5 in gastric cancer cell lines has been demonstrated to reduce both cell proliferation and colony-forming ability in vitro [45]. Additional tumor suppressors that were found upregulated upon Ch-SeNPs treatment were GPRC5A (FC = 5.90) and AKAP12 (FC = 8.11). Deletion of these transcripts has been demonstrated to promote tumor initiation and progression [46,47]. In addition to these overexpressed tumor suppressors, other interesting transcripts that are currently being considered as therapeutic targets of special interest against cancer and that were found downregulated after exposing HepG2 cells to Ch-SeNPs were ROR1 (FC = 0.50), PRLR (FC = 0.44), HMMR (FC = 0.40) and CENPI (FC = 0.29). While ROR1 knockdown resulted in decreased proliferation and migration but enhanced resistance to apoptosis and anoikis [48], PRLR overexpression has been linked with increased proliferation, viability, clonogenicity, chemoresistance and matrix metalloproteinase activity [49]. As for HMMR, its overexpression promotes germline stem cell self-renewal and intracranial tumor propagation [50], while upregulation of CENPI has been associated with tumorigenesis and drug resistance through chromosome instability [51]. Considering other potential therapeutic targets that have been found to be altered after exposure of HepG2 cells to Ch-SeNPs, the inhibition observed in the ALDOB transcript (FC = 0.20) is noteworthy. Metabolic reprogramming has been proposed as an alternative mechanism used by metastatic cells to obtain the high levels of energy they need. In particular, it has been described that metastatic cells from the liver upregulate the enzyme ALDOB, which provides fuel for several pathways of the central carbon metabolism, thus promoting the fructose metabolism. Based on this, targeting the expression of ALDOB could represent a powerful chemotherapeutic strategy against metastatic cells [52]. Interestingly, treatment with Ch-SeNPs was able to drastically inhibit the expression of ALDOB in HepG2 cells.

Oxidative stress is defined by the difference between the production of reactive oxygen species (ROS) and their elimination through metabolism. Hypoxia and oxidative stress are involved in cell cycle progression and proliferation, cell survival and apoptosis, energy metabolism, cell morphology, cell–cell adhesion, cell motility and angiogenesis and thereby can promote tumorigenesis [53,54]. Many chemotherapeutic strategies focus on the use of antioxidants to deplete tumor cells from ROS-induced survival signaling pathways. Such treatment may also have preventive functions. Since selenium has been considered as an antioxidant element, we sought to investigate the potential role of Ch-SeNPs as a promising candidate to be used in this type of chemotherapeutic strategies; thus, expression of the transcripts potentially involved in these mechanisms was also investigated. Our analysis showed downregulation of ANGPTL8 (FC = 0.30) and CD24 (FC = 0.48) in the cells exposed to Ch-SeNPs. While ANGPTL8 is considered an inflammatory marker [55], CD24 is also a much-highlighted marker for some types of cancers (e.g., breast cancer). Interestingly, it has been demonstrated that their suppression correlates with lower cell proliferation rates and reduced ROS production [56]. In addition, several transcripts from the p450 cytochrome (CYP) family, one of the main enzymatic sources of ROS production [57,58], were found downregulated in our assay at different levels: CYP7A1 (FC = 0.5), CYP4F2 (FC = 0.5), CYP3A7 (FC = 0.46), CYP3A5 (FC = 0.44) and CYPA43 (FC = 0.41). Furthermore, ADHFE1 (FC = 0.49) was also found inhibited. ADHFE1 is considered an oncoprotein associated with disease survival and known for promoting reduction of the glutamine metabolism while increasing formation of D-2-hydroxyglutarate and mitochondrial reactive oxygen species (ROS) [59]. The fact that Ch-SeNP exposure inhibited the expression of ADHFE1 also supports the antioxidant role of Ch-SeNPs. Similarly, the transcription factor SOX6 (FC = 0.48), which also promotes elevated levels of oxidative stress and is normally overexpressed in tumors such as the Ewing sarcoma, was found inhibited in HepG2 cells treated with Ch-SeNPs [60].

### 3.4. Ch-SeNPs Induce Senescence in a Preclinical Human Cell Model

**Figure 5:**
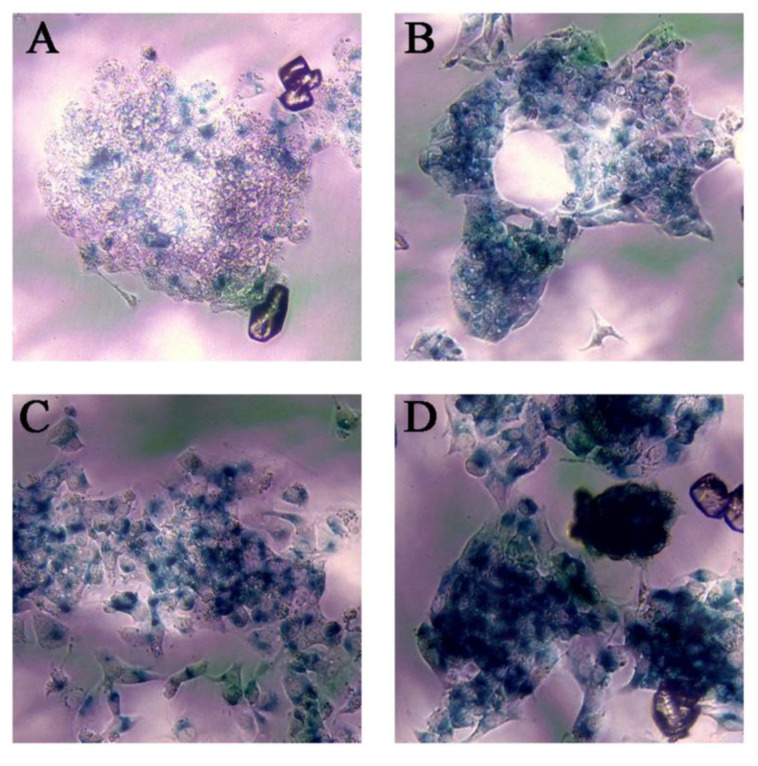
Senescence assay. Blue staining shows β-galactosidase activity. Control cells (**A**), cells exposed to 1 mg/L of Ch-SeNPs (**B**), cells exposed to 10 μM of etoposide—positive senescence control (**C**), and cells exposed to 50 μM of etoposide—positive apoptosis control (**D**).

One of the aspects considered most relevant after studying the results of the transcriptomic analysis was the ability of Ch-SeNPs to induce senescence. In order to validate such interesting results, we performed an experiment to measure the activity of β-galactosidase, a well-known marker of senescence, in HepG2 cells after exposure to Ch-SeNPs (Figure 5). The results demonstrated that mock HepG2 cells showed background levels of senescence (Figure 5A), whilst the Ch-SeNP-exposed cells did show a significant expression of β-galactosidase staining (Figure 5B). These levels were similar to those observed in cells treated with 10 μM etoposide (Figure 5C), a bona fide inducer of senescence in HepG2 cells. On the contrary, cells treated with 50 μM etoposide (Figure 5D), which is a positive control for apoptosis, showed stronger staining compared to cells treated with Ch-SeNPs, thus suggesting that Ch-SeNPs induce senescence in human hepatocarcinoma cells rather than apoptosis. In order to investigate whether or not Ch-SeNPs were able to induce senescence in different cancer cell lines, additional experiments to measure the activity of β-galactosidase in MBA-MD-231 and HeLa cells (see Figures S2 and S3 in the Supplementary Material) were also carried out. In both cases, the results were comparable to those obtained for HepG2 cells. The expression levels of β-galactosidase were similar in the cells exposed to Ch-SeNPs and the positive control of senescence (10 mM etoposide).

## 4. Conclusions

This study was carried out with the aim of identifying new mechanisms associated with the antitumor potential of the use of Ch-SeNPs. For this purpose, a model of human hepatocellular carcinoma cells (HepG2 cells) was selected and a whole transcriptome analysis was carried out. This analysis showed the alteration of different groups of transcripts involved in the arrest of the cell cycle and in the induction of senescence. Likewise, it has been proven that exposure to Ch-SeNPs induces the alteration of tumor suppressors, which supports the capacity of Ch-SeNPs to inhibit tumor proliferation. In addition, several transcripts related to ROS generation and the induction of oxidative stress were also found altered, demonstrating the antioxidant capacity of Ch-SeNPs. Overall, the set of altered transcripts allowed confirming the antitumor potential of Ch-SeNPs through their effect on several key mechanisms related to cancer progression. Especially relevant was the discovery of the capacity of Ch-SeNPs to induce senescence, which was confirmed by measuring β-galactosidase activity. Based on the promising results obtained and the proven capability of Ch-SeNPs as potential antitumor agents, the focus of future work will be on the evaluation of the effect of Ch-SeNPs in other cell lines and in pre-clinical animal models. Additionally, efforts will be made to design novel hybrid nanosystems for cancer cell targeting and selective delivery of SeNPs.

## Data Availability

Not applicable.

## Supplementary Materials

The following are available online at https://www.mdpi.com/1999-4923/13/3/356/s1, Figure S1: TEM image and XRD pattern of Ch-SeNPs, Figure S2: Senescence assay of MBA-MD-231 cells exposed to Ch-SeNPs, Figure S3: Senescence assay of HeLa cells exposed to Ch-SeNPs, Table S1: Whole transcriptome analysis of HepG2 cells exposed to Ch-SeNPs.

## Appendix A

**Table A1:** Differentially expressed transcripts in the HepG2 cells exposed to Ch-SeNPs.

Fold Change (FC)	Gene Code	Gene Name
14.23	DUSP5	dual specificity phosphatase 5
13.90	RGCC	regulator of cell cycle
9.70	PAQR5	progestin and AdipoQ receptor family member V
9.01	TMC7	transmembrane channel-like 7
8.82	AQP3	aquaporin 3 (Gill blood group)
8.11	AKAP12	A-kinase (PRKA) anchor protein 12
7.11	PALLD	palladin, cytoskeletal associated protein
7.03	PAI-1	serpin peptidase inhibitor, clade E (nexin, plasminogen activator inhibitor type 1), member 1
5.96	LURAP1L	leucine-rich adaptor protein 1-like
5.90	CSGALNACT2	chondroitin sulfate N-acetylgalactosaminyltransferase 2
5.90	GPRC5A; MIR614	G protein-coupled receptor, class C, group 5, member A; microRNA 614
5.64	C5AR1	complement component 5a receptor 1
5.42	EMP3	epithelial membrane protein 3
5.39	GPAT3	glycerol-3-phosphate acyltransferase 3
5.19	TM4SF19-TCTEX1D2	TM4SF19-TCTEX1D2 readthrough (NMD candidate)
5.02	RBM24	RNA-binding motif protein 24
4.88	TM4SF19	transmembrane 4 L six family member 19
4.88	SERPINB8	serpin peptidase inhibitor, clade B (ovalbumin), member 8
4.82	IER3	immediate early response 3
4.75	PDGFA	platelet-derived growth factor alpha polypeptide
4.65	MMP3	matrix metallopeptidase 3
4.40	CSTA	cystatin A (stefin A)
4.38	ITGA2	integrin, alpha 2 (CD49B, alpha 2 subunit of VLA-2 receptor)
4.31	CAPN2	calpain 2, (m/II) large subunit
4.25	SLC51B	solute carrier family 51, beta subunit
4.22	EGR1	early growth response 1
4.15	SLC16A6	solute carrier family 16, member 6
4.11	JAG1	jagged 1
4.09	ESAM	endothelial cell adhesion molecule
4.09	AXL	AXL receptor tyrosine kinase
4.04	PAEP	progestogen-associated endometrial protein
3.95	HEY1	hes-related family bHLH transcription factor with YRPW motif 1
3.93	FHL2	four and a half LIM domains 2
3.88	TIMP1	TIMP metallopeptidase inhibitor 1
3.85	IGFBP3	insulin-like growth factor binding protein 3
3.69	SPRY4	sprouty RTK signaling antagonist 4
3.65	F2RL1	coagulation factor II (thrombin) receptor-like 1
3.56	ITPR3	inositol 1,4,5-trisphosphate receptor, type 3
3.49	PLEKHH2	pleckstrin homology domain containing, family H (with MyTH4 domain) member 2
3.43	S100A11	S100 calcium-binding protein A11
3.42	RASD1	RAS, dexamethasone-induced 1
3.38	TUSC3	tumor suppressor candidate 3
3.37	CD55	CD55 molecule, decay-accelerating factor for complement (Cromer blood group)
3.36	HKDC1	hexokinase domain containing 1
3.35	SPIRE1	spire-type actin nucleation factor 1
3.32	ARG2	arginase 2
3.31	NKAP	NFKB-activating protein
3.26	GDF15	growth differentiation factor 15
3.25	ACSL5	acyl-CoA synthetase long-chain family member 5
3.24	AREG	amphiregulin
3.23	EGFR	epidermal growth factor receptor
3.21	SLC1A2	solute carrier family 1 (glial high-affinity glutamate transporter), member 2
3.21	ARHGEF2	Rho/Rac guanine nucleotide exchange factor 2
3.18	SFN	stratifin
3.13	IGFBP1	insulin-like growth factor-binding protein 1
3.09	LGALS3	lectin, galactoside-binding, soluble, 3
3.07	ID1	inhibitor of DNA binding 1, dominant negative helix–loop–helix protein
3.06	SRGAP1	SLIT-ROBO Rho GTPase-activating protein 1
3.05	NTSR1	neurotensin receptor 1 (high affinity)
3.05	ELK3	ELK3, ETS-domain protein (SRF accessory protein 2)
3.00	CACNA2D4	calcium channel, voltage-dependent, alpha 2/delta subunit 4
2.99	HPCAL1	hippocalcin-like 1
2.99	GTPBP2	GTP-binding protein 2
2.95	MCTP1	multiple C2 domains, transmembrane 1
2.93	TCP11L2	t-complex 11, testis-specific-like 2
2.92	SH3RF1	SH3 domain containing ring finger 1
2.91	FOSL1	FOS-like antigen 1
2.90	ATP6V0D2	ATPase, H+ transporting, lysosomal 38kDa, V0 subunit d2
2.87	DYNC2H1	dynein, cytoplasmic 2, heavy-chain 1
2.85	CLIP4	CAP-GLY domain-containing linker protein family, member 4
2.85	RAP1GAP2	RAP1 GTPase-activating protein 2
2.83	SERPINE2	serpin peptidase inhibitor, clade E (nexin, plasminogen activator inhibitor type 1), member 2
2.83	IL6R	interleukin 6 receptor
2.81	KLF5	Kruppel-like factor 5 (intestinal)
2.80	NDE1; MIR484	nudE neurodevelopment protein 1; microRNA 484
2.80	ATF6	activating transcription factor 6
2.78	CCPG1; MIR628	cell cycle progression 1; microRNA 628
2.78	ARAP2	ArfGAP with RhoGAP domain, ankyrin repeat and PH domain 2
2.77	TRIB1	tribbles pseudokinase 1
2.74	ERRFI1	ERBB receptor feedback inhibitor 1
2.74	GCLC	glutamate-cysteine ligase, catalytic subunit
2.73	SGMS2	sphingomyelin synthase 2
2.73	CD22; MIR5196	CD22 molecule; microRNA 5196
2.71	MT2A	metallothionein 2A
2.69	GLIPR1	GLI pathogenesis-related 1
2.67	CDKN1A	cyclin-dependent kinase inhibitor 1A (p21, Cip1)
2.67	CEMIP	cell migration inducing protein, hyaluronan-binding
2.66	TGFB1	transforming growth factor beta 1
2.65	CD58	CD58 molecule
2.63	MEP1A	meprin A, alpha (PABA peptide hydrolase)
2.62	BHLHE40	basic helix–loop–helix family, member e40
2.62	TIMM9	translocase of inner mitochondrial membrane 9 homolog (yeast)
2.62	BMP6	bone morphogenetic protein 6
2.62	IL4R	interleukin 4 receptor
2.60	TMEM2	transcript identified by AceView, Entrez Gene ID(s) 23670
2.58	DMRTA1	DMRT-like family A1
2.58	ANKRD1	ankyrin repeat domain 1 (cardiac muscle)
2.55	MT1B; MT1CP	metallothionein 1B; metallothionein 1C, pseudogene
2.51	AKR1B10	aldo-keto reductase family 1, member B10 (aldose reductase)
2.49	CD109	CD109 molecule
2.48	SLC20A1	solute carrier family 20 (phosphate transporter), member 1
2.46	ATF3	activating transcription factor 3
2.46	TAGLN3	transgelin 3
2.45	MOSPD1	motile sperm domain-containing 1
2.45	IL11	interleukin 11
2.43	KDM7A	lysine (K)-specific demethylase 7A
2.42	CCR7	chemokine (C-C motif) receptor 7
2.42	MXD1	MAX dimerization protein 1
2.40	PITPNC1	phosphatidylinositol transfer protein, cytoplasmic 1
2.40	PLAUR	plasminogen activator, urokinase receptor
2.40	RABGGTB; SNORD45B; SNORD45A; SNORD45C	Rab geranylgeranyltransferase, beta subunit; small nucleolar RNA, C/D box 45B; small nucleolar RNA, C/D box 45A; small nucleolar RNA, C/D box 45C
2.39	LETM2	leucine zipper-EF-hand-containing transmembrane protein 2
2.36	C6orf48; SNORD52; SNORD48	chromosome 6 open reading frame 48; small nucleolar RNA, C/D box 52; small nucleolar RNA, C/D box 48
2.36	AP1S3	adaptor-related protein complex 1 sigma 3 subunit
2.36	PPP1R15A	protein phosphatase 1, regulatory subunit 15A
2.36	ARL8A	ADP-ribosylation factor like GTPase 8A
2.36	PAQR3	progestin and AdipoQ receptor family member III
2.35	LCP1	lymphocyte cytosolic protein 1 (L-plastin)
2.34	HMGA1	high-mobility group AT-hook 1
2.33	RCL1	RNA terminal phosphate cyclase-like 1
2.33	ANTXR2	anthrax toxin receptor 2
2.31	KPNA5	karyopherin alpha 5 (importin alpha 6)
2.30	RRAS2	related RAS viral (r-ras) oncogene homolog 2
2.30	CCL20	chemokine (C–C motif) ligand 20
2.30	TUBE1	tubulin, epsilon 1
2.30	LRRC8B	leucine-rich repeat-containing 8 family, member B
2.29	DFNA5	deafness, autosomal dominant 5
2.28	ERN1	endoplasmic reticulum to nucleus signaling 1
2.28	ZADH2	zinc binding alcohol dehydrogenase domain-containing 2
2.27	PPP1R18	protein phosphatase 1, regulatory subunit 18
2.26	ASB2	ankyrin repeat and SOCS box-containing 2
2.26	IDS	iduronate 2-sulfatase
2.25	MAGI2	membrane-associated guanylate kinase, WW and PDZ domain-containing 2
2.25	MT1A	metallothionein 1A
2.25	ANXA3	annexin A3
2.25	ZAK	sterile alpha motif and leucine zipper-containing kinase
2.24	CSF1	colony-stimulating factor 1 (macrophage)
2.24	PIM1	Pim-1 proto-oncogene, serine/threonine kinase
2.23	MSANTD3	Myb/SANT-like DNA-binding domain containing 3
2.22	NAGS	N-acetylglutamate synthase
2.22	REXO2	RNA exonuclease 2
2.22	VASP	vasodilator-stimulated phosphoprotein
2.21	CREB5	cAMP responsive element-binding protein 5
2.21	STEAP2	STEAP family member 2, metalloreductase
2.21	MRGPRX4	MAS-related GPR, member X4
2.20	ITPRIP	inositol 1,4,5-trisphosphate receptor-interacting protein
2.20	DIEXF	digestive organ expansion factor homolog (zebrafish)
2.20	MT1X	metallothionein 1X
2.19	PTGR1	prostaglandin reductase 1
2.19	C9orf72	chromosome 9 open reading frame 72
2.19	ABR	active BCR-related
2.18	HIVEP2	human immunodeficiency virus type I enhancer-binding protein 2
2.18	KLF11	Kruppel-like factor 11
2.18	VNN1	Vanin 1
2.17	LAPTM5	lysosomal protein transmembrane 5
2.17	MT1IP	metallothionein 1I, pseudogene
2.17	CAMSAP2	calmodulin regulated spectrin-associated protein family, member 2
2.16	DPY19L4	dpy-19-like 4 (C. elegans)
2.16	SLC22A15	solute carrier family 22, member 15
2.16	PTPN3	protein tyrosine phosphatase, non-receptor type 3
2.16	SPP1	secreted phosphoprotein 1
2.16	NLRC4	NLR family, CARD domain-containing 4
2.16	CMSS1	cms1 ribosomal small subunit homolog (yeast)
2.15	HBEGF	heparin-binding EGF-like growth factor
2.15	TGM2	transglutaminase 2
2.15	NLN	neurolysin (metallopeptidase M3 family)
2.14	APOPT1	apoptogenic 1, mitochondrial
2.14	UPP1	uridine phosphorylase 1
2.13	LAMP3	lysosomal-associated membrane protein 3
2.13	C5orf28	chromosome 5 open reading frame 28
2.13	P2RX5-TAX1BP3	P2RX5-TAX1BP3 readthrough (NMD candidate)
2.13	PHF21A	PHD finger protein 21A
2.12	CTSB	cathepsin B
2.12	S100P	S100 calcium-binding protein P
2.11	ANKRA2	ankyrin repeat, family A (RFXANK-like), 2
2.11	FLOT1	transcript identified by AceView, Entrez Gene ID(s) 10211
2.11	CEP290	centrosomal protein 290kDa
2.11	MT1H	metallothionein 1H
2.10	RIT1	Ras-like without CAAX 1
2.09	CEP295NL; TIMP2	CEP295 N-terminal like; TIMP metallopeptidase inhibitor 2
2.09	CES1	carboxylesterase 1
2.09	ASF1A	anti-silencing function 1A histone chaperone
2.09	OPTN	optineurin
2.09	GRB10	growth factor receptor-bound protein 10
2.09	AKR1B15	aldo-keto reductase family 1, member B15
2.08	MTMR6	myotubularin-related protein 6
2.08	FAN1	FANCD2/FANCI-associated nuclease 1
2.08	ACTR10	actin-related protein 10 homolog (*S. cerevisiae*)
2.08	RNF19B	ring finger protein 19B
2.08	NFIL3	nuclear factor, interleukin 3-regulated
2.08	NQO1	NAD(P)H dehydrogenase, quinone 1
2.08	DHRS7	dehydrogenase/reductase (SDR family) member 7
2.08	MT1L	metallothionein 1L (gene/pseudogene)
2.07	LIF	leukemia inhibitory factor
2.07	DUSP12	dual specificity phosphatase 12
2.07	KCNMB3	potassium channel subfamily M regulatory beta subunit 3
2.06	PXK	PX domain-containing serine/threonine kinase
2.06	CD9	CD9 molecule
2.06	H1F0	H1 histone family, member 0
2.06	ADORA2B	adenosine A2b receptor
2.06	KRT23	keratin 23, type I
2.06	BTG3	BTG family, member 3
2.05	AOX1	aldehyde oxidase 1
2.04	SNORA17A; SNORA17B; SNHG7	small nucleolar RNA, H/ACA box 17A; small nucleolar RNA, H/ACA box 17B; small nucleolar RNA host gene 7
2.04	CPNE8	copine VIII
2.04	EIF4A2; SNORA63; SNORD2; SNORA4; SNORA81; MIR1248	eukaryotic translation initiation factor 4A2; small nucleolar RNA, H/ACA box 63; small nucleolar RNA, C/D box 2; small nucleolar RNA, H/ACA box 4; small nucleolar RNA, H/ACA box 81; microRNA 1248
2.03	SMAD6	SMAD family member 6
2.03	YIPF4	Yip1 domain family member 4
2.03	FASTKD1	FAST kinase domains 1
2.03	TMTC3	transmembrane and tetratricopeptide repeat containing 3
2.02	ADAT2	adenosine deaminase, tRNA-specific 2
2.02	ago-02	argonaute RISC catalytic component 2
2.02	TMEM167B	transmembrane protein 167B
2.01	DCAF10	DDB1 and CUL4 associated factor 10
2.01	RAB3GAP1	RAB3 GTPase-activating protein subunit 1 (catalytic)
2.00	GBE1	glucan (1,4-alpha-), branching enzyme 1
2.00	CLIP2	CAP-GLY domain-containing linker protein 2
2.00	SOWAHC	sosondowah ankyrin repeat domain family member C
2.00	NEK3	NIMA-related kinase 3
2.00	IFRD1	interferon-related developmental regulator 1
2.00	TBPL1	TBP-like 1
0.50	ROR1	receptor tyrosine kinase-like orphan receptor 1
0.50	FAM111B	family with sequence similarity 111, member B
0.50	CDCA2	cell division cycle-associated 2
0.50	DIO1	deiodinase, iodothyronine, type I
0.50	HIST1H1B	histone cluster 1, H1b
0.50	HOOK2	hook microtubule-tethering protein 2
0.50	CYP7A1	cytochrome P450, family 7, subfamily A, polypeptide 1
0.50	CYP4F2	cytochrome P450, family 4, subfamily F, polypeptide 2
0.50	PNPLA3	patatin-like phospholipase domain-containing 3
0.49	TSACC	TSSK6-activating co-chaperone
0.49	HIST1H4A	histone cluster 1, H4a
0.49	ANGPTL3	angiopoietin-like 3
0.49	ADHFE1; C8orf46	alcohol dehydrogenase, iron-containing 1; chromosome 8 open reading frame 46
0.49	GINS2	GINS complex subunit 2 (Psf2 homolog)
0.49	ARHGEF39	Rho guanine nucleotide exchange factor 39
0.49	POTEF	POTE ankyrin domain family, member F
0.49	HIST1H2AG	histone cluster 1, H2ag
0.49	ZNF341	zinc finger protein 341
0.49	NAT6	N-acetyltransferase 6 (GCN5-related)
0.48	FRY	FRY microtubule-binding protein
0.48	SOX6; MIR6073	SRY box 6; microRNA 6073
0.48	SGOL2	shugoshin-like 2 (*S. pombe*)
0.48	MTHFR	methylenetetrahydrofolate reductase (NAD(P)H)
0.48	CD24	CD24 molecule
0.47	ITIH1	inter-alpha-trypsin inhibitor heavy-chain 1
0.47	SCARA3	scavenger receptor class A, member 3
0.47	TMEM143	transmembrane protein 143
0.47	CDC25C	cell division cycle 25C
0.46	LEAP2	liver-expressed antimicrobial peptide 2
0.46	ZNF565	zinc finger protein 565
0.46	E2F8	E2F transcription factor 8
0.46	CYP3A7; CYP3A7-CYP3A51P	cytochrome P450, family 3, subfamily A, polypeptide 7; CYP3A7-CYP3A51P readthrough
0.45	ESCO2	establishment of sister chromatid cohesion N-acetyltransferase 2
0.45	ODAM	odontogenic, ameloblast-associated
0.45	C1orf116	chromosome 1 open reading frame 116
0.44	HIST2H3A	histone cluster 2, H3a
0.44	CHGB	chromogranin B
0.44	PRLR	prolactin receptor
0.44	HIST2H3A; HIST2H3C	histone cluster 2, H3a; histone cluster 2, H3c
0.44	CYP3A5	cytochrome P450, family 3, subfamily A, polypeptide 5
0.43	HIST1H2BM	histone cluster 1, H2bm
0.43	SNAI2	snail family zinc finger 2
0.43	PRRG2	proline-rich Gla (G-carboxyglutamic acid) 2
0.42	SGOL1	shugoshin-like 1 (*S. pombe*)
0.42	SLC22A7	solute carrier family 22 (organic anion transporter), member 7
0.41	CYP3A43	cytochrome P450, family 3, subfamily A, polypeptide 43
0.41	LINC00612	long intergenic non-protein coding RNA 612
0.41	IFIT3	interferon-induced protein with tetratricopeptide repeats 3
0.40	HMMR	hyaluronan-mediated motility receptor (RHAMM)
0.39	CDH1	cadherin 1, type 1
0.39	HIST1H3B	histone cluster 1, H3b
0.38	AFM	afamin
0.36	DEPDC4	DEP domain-containing 4
0.36	YPEL2	yippee like 2
0.32	G6PC	glucose-6-phosphatase, catalytic subunit
0.30	ANGPTL8	angiopoietin like 8
0.29	CENPI	centromere protein I
0.20	ALDOB	aldolase B, fructose-bisphosphate
