# Supplementary material for: Transcriptome Analysis Identifies Novel Mechanisms Associated with the Antitumor Effect of Chitosan-Stabilized Selenium Nanoparticles
Source: Pharmaceutics. 2021 Mar 8;13(3):356. doi: 10.3390/pharmaceutics13030356 (PMC8000472; doi:10.3390/pharmaceutics13030356)
Supplement: Supplementary file 1 [file pharmaceutics-13-00356-s001.pdf]

pharmaceutics

MDPI

Supplementary Material: Transcriptome Analysis Identifies Novel Mechanisms Associated with the Antitumor Effect of Chitosan-Stabilized Selenium Nanoparticles

Hector Estevez, Estefania Garcia-Calvo, Jose Rivera-Torres, María Vallet-Regí, Blanca González and Jose L. Luque-Garcia

Citation: Estevez, H.; Garcia-Calvo, E.; Rivera-Torres, J.; Vallet-Regí, M.; González, B.; Luque-Garcia, J.L. Transcriptome Analysis Identifies Novel Mechanisms Associated to the Anti-Tumoral Effect of Chitosan-Stabilized Selenium Nanoparticles. *Pharmaceutics* **2021**, *13*

Publisher's Note: MDPI stays neutral with regard to jurisdictional claims in published maps and institutional affiliations.

Copyright: © 2021 by the authors. Submitted for possible open access publication under the terms and conditions of the Creative Commons Attribution (CC BY) license (http://creativecommons.org/licenses/by/4.0/).

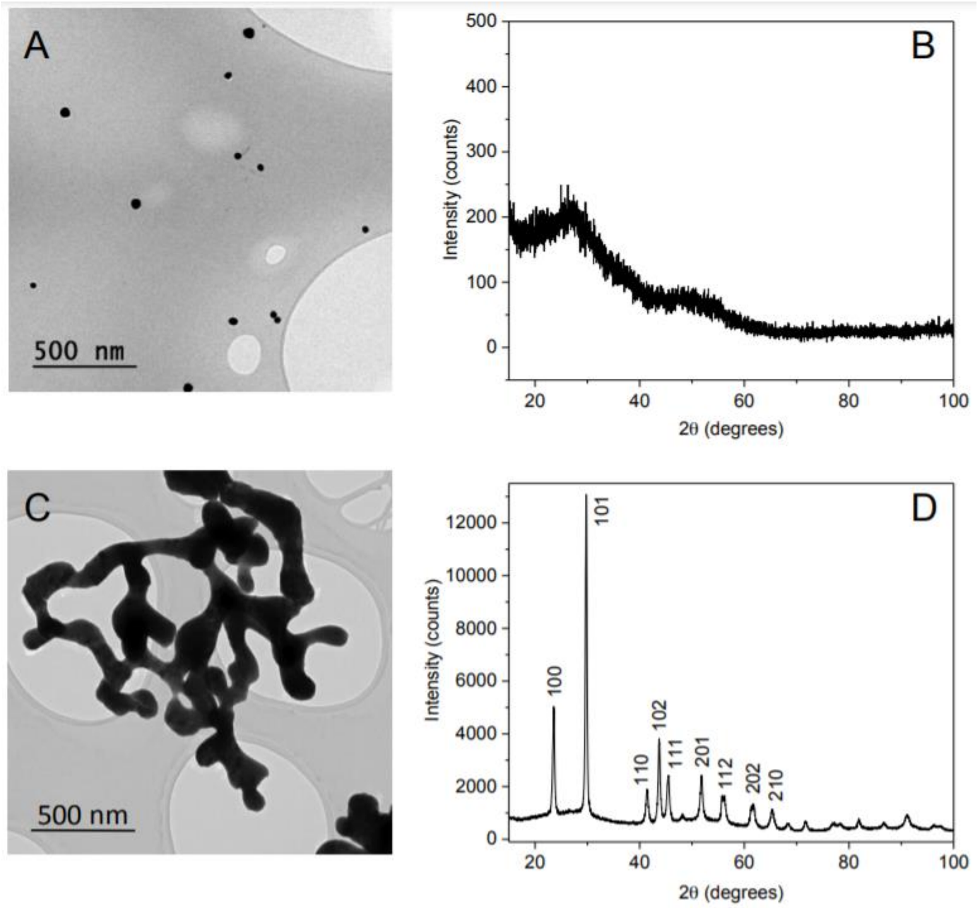

Figure S1. TEM image and XRD pattern of Ch-SeNPs ((**A**) and (**B**), respectively) and TEM image and XRD pattern of a selenium sample synthesized following the same chemical reduction route and conditions used for Ch-SeNPs but in the absence of chitosan ((**C**) and (**D**), respectively). X-ray diffractogram in figure **D**) also displays the Miller indices indexed to crystalline selenium (JCPDS card no. 06-0362).

The XRD pattern of Ch-SeNPs (Figure S1-B) shows broad peaks and low signal-to-noise ratios that is ascribed to a small crystalline domain size in the range of few nm. Therefore, the Xray diffractogram of a selenium sample obtained following the same chemical reduction route and conditions used for Ch-SeNPs but in the absence of chitosan is also displayed (Figure S1-D). In the absence of chitosan as stabilizer, elemental selenium precipitates out of the nanoscale as shown in the TEM image (Figure S1-C). The XRD pattern of this sample shows well-defined Bragg peaks, which can be indexed to crystalline trigonal selenium in good agreement with the JCPDS card nº 06-0362 and literature [1–5]. The broad peaks in the X-ray diffractogram of Ch-SeNPs centered around

27° and 50° 2θ would correspond to (100) and (101) and (102), (111) and (201) reflections, respectively.

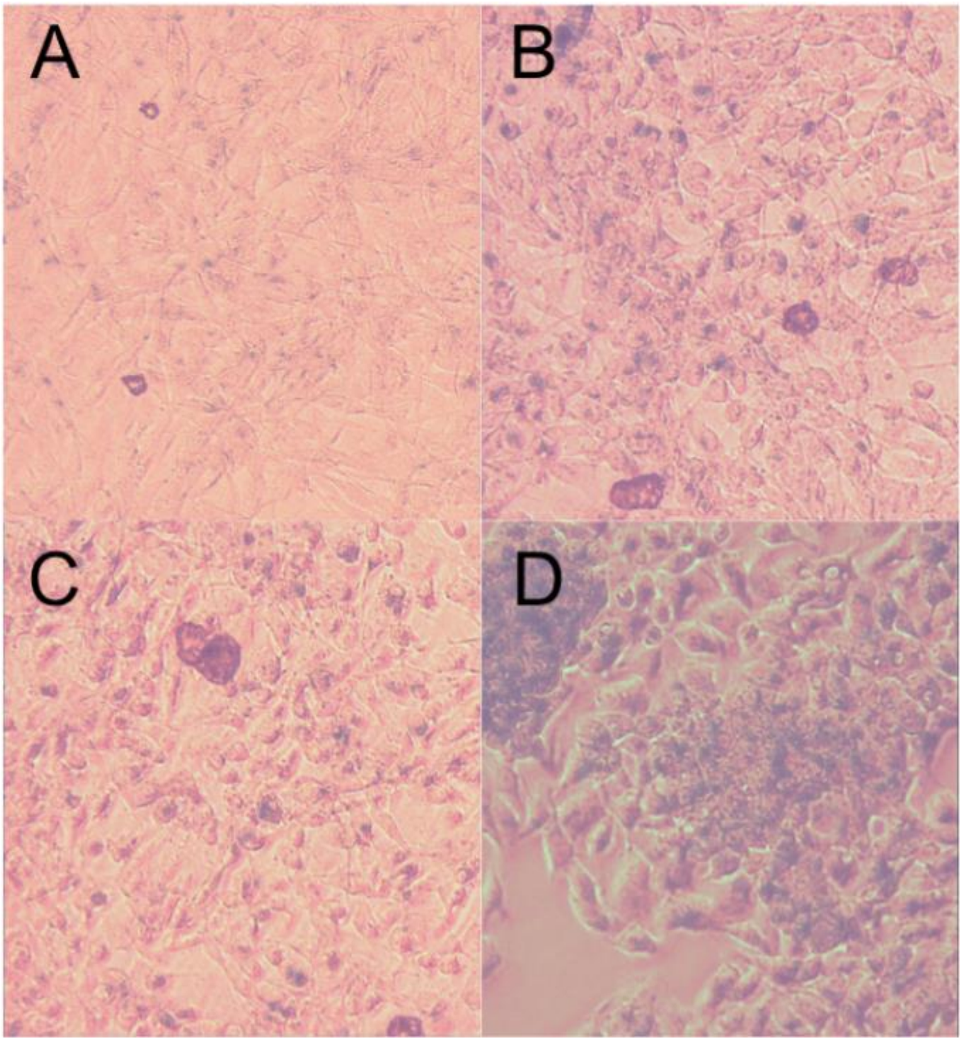

Figure S2. Senescence assay. Blue staining shows β-galactosidase activity in MBA-MD-231 cells. Control cells (**A**), cells exposed to 1 mg/L of Ch-SeNPs (**B**), cells exposed to 10 μM of etoposide – senescence positive control (**C**), and cells exposed to 50 μM of etoposide – apoptosis positive control (**D**).

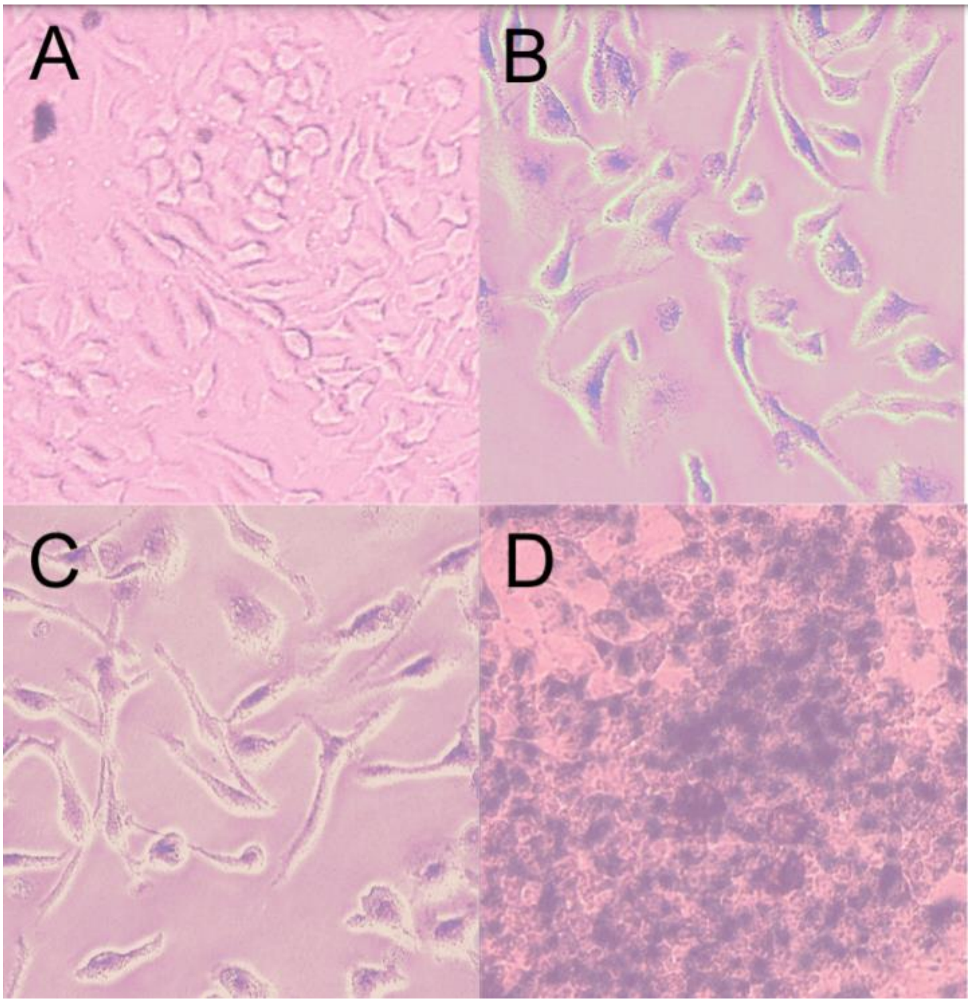

Figure S3. Senescence assay. Blue staining shows β-galactosidase activity in HeLa cells. Control cells (**A**), cells exposed to 1 mg/L of Ch-SeNPs (**B**), cells exposed to 10 μM of etoposide – senescence positive control (**C**), and cells exposed to 50 μM of etoposide – apoptosis positive control (**D**).

Table S1. Whole transcriptome analysis of HepG2 cells exposed to ChSeNPs.

**SeNPs *vs.* Control**	**Gene code**	**Gene name**
14.23	DUSP5	dual specificity phosphatase 5
13.90	RGCC	regulator of cell cycle
9.70	PAQR5	progestin and adipoQ receptor family member V
9.01	TMC7	transmembrane channel like 7
8.82	AQP3	aquaporin 3 (Gill blood group)
8.11	AKAP12	A kinase (PRKA) anchor protein 12
7.11	PALLD	palladin. cytoskeletal associated protein

7.03	SERPINE1	serpin peptidase inhibitor. clade E (nexin. plasminogen activator inhibitor type 1). member 1
5.96	LURAP1L	leucine rich adaptor protein 1-like
5.90	CSGALNACT2	chondroitin sulfate N-acetylgalactosaminyltransferase 2
5.90	GPRC5A; MIR614	G protein-coupled receptor. class C. group 5. member A; microRNA 614
5.64	C5AR1	complement component 5a receptor 1
5.42	EMP3	epithelial membrane protein 3
5.39	GPAT3	glycerol-3-phosphate acyltransferase 3
5.19	TM4SF19-TCTEX1D2	TM4SF19-TCTEX1D2 readthrough (NMD candidate)
5.02	RBM24	RNA binding motif protein 24
4.88	TM4SF19	transmembrane 4 L six family member 19
4.88	SERPINB8	serpin peptidase inhibitor. clade B (ovalbumin). member 8
4.82	IER3	immediate early response 3
4.75	PDGFA	platelet-derived growth factor alpha polypeptide
4.65	MMP3	matrix metallopeptidase 3
4.40	CSTA	cystatin A (stefin A)
4.38	ITGA2	integrin. alpha 2 (CD49B. alpha 2 subunit of VLA-2 receptor)
4.31	CAPN2	calpain 2. (m/II) large subunit
4.25	SLC51B	solute carrier family 51. beta subunit
4.22	EGR1	early growth response 1
4.15	SLC16A6	solute carrier family 16. member 6
4.11	JAG1	jagged 1
4.09	ESAM	endothelial cell adhesion molecule
4.09	AXL	AXL receptor tyrosine kinase
4.04	PAEP	progestagen-associated endometrial protein
3.95	HEY1	hes-related family bHLH transcription factor with YRPW motif 1

3.93	FHL2	four and a half LIM domains 2
3.88	TIMP1	TIMP metallopeptidase inhibitor 1
3.85	IGFBP3	insulin like growth factor binding protein 3
3.69	SPRY4	sprouty RTK signaling antagonist 4
3.65	F2RL1	coagulation factor II (thrombin) receptor-like 1
3.56	ITPR3	inositol 1.4.5-trisphosphate receptor. type 3
3.49	PLEKHH2	pleckstrin homology domain containing. family H (with MyTH4 domain) member 2
3.43	S100A11	S100 calcium binding protein A11
3.42	RASD1	RAS. dexamethasone-induced 1
3.38	TUSC3	tumor suppressor candidate 3
3.37	CD55	CD55 molecule. decay accelerating factor for complement (Cromer blood group)
3.36	HKDC1	hexokinase domain containing 1
3.35	SPIRE1	spire-type actin nucleation factor 1
3.32	ARG2	arginase 2
3.31	NKAP	NFKB activating protein
3.26	GDF15	growth differentiation factor 15
3.25	ACSL5	acyl-CoA synthetase long-chain family member 5
3.24	AREG	amphiregulin
3.23	EGFR	epidermal growth factor receptor
3.21	SLC1A2	solute carrier family 1 (glial high affinity glutamate transporter). member 2
3.21	ARHGEF2	Rho/Rac guanine nucleotide exchange factor 2
3.18	SFN	stratifin
3.13	IGFBP1	insulin like growth factor binding protein 1
3.09	LGALS3	lectin. galactoside-binding. soluble. 3
3.07	ID1	inhibitor of DNA binding 1. dominant negative helix-loop-helix protein

3.06	SRGAP1	SLIT-ROBO Rho GTPase activating protein 1
3.05	NTSR1	neurotensin receptor 1 (high affinity)
3.05	ELK3	ELK3. ETS-domain protein (SRF accessory protein 2)
3.00	CACNA2D4	calcium channel. voltage-dependent. alpha 2/delta subunit 4
2.99	HPCAL1	hippocalcin-like 1
2.99	GTPBP2	GTP binding protein 2
2.95	MCTP1	multiple C2 domains. transmembrane 1
2.93	TCP11L2	t-complex 11. testis-specific-like 2
2.92	SH3RF1	SH3 domain containing ring finger 1
2.91	FOSL1	FOS-like antigen 1
2.90	ATP6V0D2	ATPase. H+ transporting. lysosomal 38kDa. V0 subunit d2
2.87	DYNC2H1	dynein. cytoplasmic 2. heavy chain 1
2.85	CLIP4	CAP-GLY domain containing linker protein family. member 4
2.85	RAP1GAP2	RAP1 GTPase activating protein 2
2.83	SERPINE2	serpin peptidase inhibitor. clade E (nexin. plasminogen activator inhibitor type 1). member 2
2.83	IL6R	interleukin 6 receptor
2.81	KLF5	Kruppel-like factor 5 (intestinal)
2.80	NDE1; MIR484	nudE neurodevelopment protein 1; microRNA 484
2.80	ATF6	activating transcription factor 6
2.78	CCPG1; MIR628	cell cycle progression 1; microRNA 628
2.78	ARAP2	ArfGAP with RhoGAP domain. ankyrin repeat and PH domain 2
2.77	TRIB1	tribbles pseudokinase 1
2.74	ERRFI1	ERBB receptor feedback inhibitor 1
2.74	GCLC	glutamate-cysteine ligase. catalytic subunit
2.73	SGMS2	sphingomyelin synthase 2

2.73	CD22; MIR5196	CD22 molecule; microRNA 5196
2.71	MT2A	metallothionein 2A
2.69	GLIPR1	GLI pathogenesis-related 1
2.67	CDKN1A	cyclin-dependent kinase inhibitor 1A (p21. Cip1)
2.67	CEMIP	cell migration inducing protein. hyaluronan binding
2.66	TGFB1	transforming growth factor beta 1
2.65	CD58	CD58 molecule
2.63	MEP1A	meprin A. alpha (PABA peptide hydrolase)
2.62	BHLHE40	basic helix-loop-helix family. member e40
2.62	TIMM9	translocase of inner mitochondrial membrane 9 homolog (yeast)
2.62	BMP6	bone morphogenetic protein 6
2.62	IL4R	interleukin 4 receptor
2.60	TMEM2	Transcript Identified by AceView. Entrez Gene ID(s) 23670
2.58	DMRTA1	DMRT-like family A1
2.58	ANKRD1	ankyrin repeat domain 1 (cardiac muscle)
2.55	MT1B; MT1CP	metallothionein 1B; metallothionein 1C. pseudogene
2.51	AKR1B10	aldo-keto reductase family 1. member B10 (aldose reductase)
2.49	CD109	CD109 molecule
2.48	SLC20A1	solute carrier family 20 (phosphate transporter). member 1
2.46	ATF3	activating transcription factor 3
2.46	TAGLN3	transgelin 3
2.45	MOSPD1	motile sperm domain containing 1
2.45	IL11	interleukin 11
2.43	KDM7A	lysine (K)-specific demethylase 7A
2.42	CCR7	chemokine (C-C motif) receptor 7
2.42	MXD1	MAX dimerization protein 1

2.40	PITPNC1	phosphatidylinositol transfer protein. cytoplasmic 1
2.40	PLAUR	plasminogen activator. urokinase receptor
2.40	RABGGTB; SNORD45B; SNORD45A; SNORD45C	Rab geranylgeranyltransferase. beta subunit; small nucleolar RNA. C/D box 45B; small nucleolar RNA. C/D box 45A; small nucleolar RNA. C/D box 45C
2.39	LETM2	leucine zipper-EF-hand containing transmembrane protein 2
2.36	C6orf48; SNORD52; SNORD48	chromosome 6 open reading frame 48; small nucleolar RNA. C/D box 52; small nucleolar RNA. C/D box 48
2.36	AP1S3	adaptor-related protein complex 1 sigma 3 subunit
2.36	PPP1R15A	protein phosphatase 1. regulatory subunit 15A
2.36	ARL8A	ADP-ribosylation factor like GTPase 8A
2.36	PAQR3	progestin and adipoQ receptor family member III
2.35	LCP1	lymphocyte cytosolic protein 1 (L-plastin)
2.34	HMGA1	high mobility group AT-hook 1
2.33	RCL1	RNA terminal phosphate cyclase-like 1
2.33	ANTXR2	anthrax toxin receptor 2
2.31	KPNA5	karyopherin alpha 5 (importin alpha 6)
2.30	RRAS2	related RAS viral (r-ras) oncogene homolog 2
2.30	CCL20	chemokine (C-C motif) ligand 20
2.30	TUBE1	tubulin. epsilon 1
2.30	LRRC8B	leucine rich repeat containing 8 family. member B
2.29	DFNA5	deafness. autosomal dominant 5
2.28	ERN1	endoplasmic reticulum to nucleus signaling 1
2.28	ZADH2	zinc binding alcohol dehydrogenase domain containing 2
2.27	PPP1R18	protein phosphatase 1. regulatory subunit 18
2.26	ASB2	ankyrin repeat and SOCS box containing 2
2.26	IDS	iduronate 2-sulfatase
2.25	MAGI2	membrane associated guanylate kinase. WW and PDZ domain containing 2

2.25	MT1A	metallothionein 1A
2.25	ANXA3	annexin A3
2.25	ZAK; pk	sterile alpha motif and leucine zipper containing kinase AZK; Mitogen-activated protein kinase kinase kinase MLT [Source:UniProtKB/Swiss-Prot;Acc:Q9NYL2]; Salzman2013 ANNOTATED. CDS. coding. INTERNAL. OVCODE. OVERLAPTX. OVEXON best transcript NM_133646; Salzman2013 ANNOTATED. CDS. coding. OVCODE. OVERLAPTX. OVEXON. UTR3 best transcript NM_016653; Salzman2013 ANNOTATED. CDS. coding. OVCODE. OVERLAPTX. OVEXON. UTR3 best transcript NM_133646; Salzman2013 ANNOTATED. CDS. coding. INTERNAL. OVCODE. OVERLAPTX. OVEXON best transcript NM_016653; Transcript Identified by AceView. Entrez Gene ID(s) 51776
2.24	CSF1	colony stimulating factor 1 (macrophage)
2.24	PIM1	Pim-1 proto-oncogene. serine/threonine kinase
2.23	MSANTD3	Myb/SANT-like DNA-binding domain containing 3
2.22	NAGS	N-acetylglutamate synthase
2.22	REXO2	RNA exonuclease 2
2.22	VASP	vasodilator-stimulated phosphoprotein
2.21	CREB5	cAMP responsive element binding protein 5
2.21	STEAP2	STEAP family member 2. metalloreductase
2.21	MRGPRX4	MAS-related GPR. member X4
2.20	ITPRIP	inositol 1.4.5-trisphosphate receptor interacting protein
2.20	DIEXF	digestive organ expansion factor homolog (zebrafish)
2.20	MT1X	metallothionein 1X
2.19	PTGR1	prostaglandin reductase 1
2.19	C9orf72	chromosome 9 open reading frame 72
2.19	ABR	active BCR-related
2.18	HIVEP2	human immunodeficiency virus type I enhancer binding protein 2
2.18	KLF11	Kruppel-like factor 11

2.18	VNN1	vanin 1
2.17	LAPTM5	lysosomal protein transmembrane 5
2.17	MT1IP	metallothionein 1I. pseudogene
2.17	CAMSAP2	calmodulin regulated spectrin-associated protein family. member 2
2.16	DPY19L4	dpy-19-like 4 (C. elegans)
2.16	SLC22A15	solute carrier family 22. member 15
2.16	PTPN3	protein tyrosine phosphatase. non-receptor type 3
2.16	SPP1	secreted phosphoprotein 1
2.16	NLRC4	NLR family. CARD domain containing 4
2.16	CMSS1	cms1 ribosomal small subunit homolog (yeast)
2.15	HBEGF	heparin-binding EGF-like growth factor
2.15	TGM2	transglutaminase 2
2.15	NLN	neurolysin (metallopeptidase M3 family)
2.14	APOPT1	apoptogenic 1. mitochondrial
2.14	UPP1	uridine phosphorylase 1
2.13	LAMP3	lysosomal-associated membrane protein 3
2.13	C5orf28	chromosome 5 open reading frame 28
2.13	P2RX5-TAX1BP3	P2RX5-TAX1BP3 readthrough (NMD candidate)
2.13	PHF21A	PHD finger protein 21A
2.12	CTSB	cathepsin B
2.12	S100P	S100 calcium binding protein P
2.11	ANKRA2	ankyrin repeat. family A (RFXANK-like). 2
2.11	FLOT1	Transcript Identified by AceView. Entrez Gene ID(s) 10211
2.11	CEP290	centrosomal protein 290kDa
2.11	MT1H	metallothionein 1H
2.10	RIT1	Ras-like without CAAX 1

2.09	CEP295NL; TIMP2	CEP295 N-terminal like; TIMP metallopeptidase inhibitor 2
2.09	CES1	carboxylesterase 1
2.09	ASF1A	anti-silencing function 1A histone chaperone
2.09	OPTN	optineurin
2.09	GRB10	growth factor receptor bound protein 10
2.09	AKR1B15	aldo-keto reductase family 1. member B15
2.08	MTMR6	myotubularin related protein 6
2.08	FAN1	FANCD2/FANCI-associated nuclease 1
2.08	ACTR10	actin-related protein 10 homolog (S. cerevisiae)
2.08	RNF19B	ring finger protein 19B
2.08	NFIL3	nuclear factor. interleukin 3 regulated
2.08	NQO1	NAD(P)H dehydrogenase. quinone 1
2.08	DHRS7	dehydrogenase/reductase (SDR family) member 7
2.08	MT1L	metallothionein 1L (gene/pseudogene)
2.07	LIF	leukemia inhibitory factor
2.07	DUSP12	dual specificity phosphatase 12
2.07	KCNMB3	potassium channel subfamily M regulatory beta subunit 3
2.06	PXK	PX domain containing serine/threonine kinase
2.06	CD9	CD9 molecule
2.06	H1F0	H1 histone family. member 0
2.06	ADORA2B	adenosine A2b receptor
2.06	KRT23	keratin 23. type I
2.06	BTG3	BTG family. member 3
2.05	AOX1	aldehyde oxidase 1
2.04	SNORA17A; SNORA17B; SNHG7	small nucleolar RNA. H/ACA box 17A; small nucleolar RNA. H/ACA box 17B; small nucleolar RNA host gene 7

2.04	CPNE8	copine VIII
2.04	EIF4A2; SNORA63; SNORD2; SNORA4; SNORA81; MIR1248	eukaryotic translation initiation factor 4A2; small nucleolar RNA. H/ACA box 63; small nucleolar RNA. C/D box 2; small nucleolar RNA. H/ACA box 4; small nucleolar RNA. H/ACA box 81; microRNA 1248
2.03	SMAD6	SMAD family member 6
2.03	YIPF4	Yip1 domain family member 4
2.03	FASTKD1	FAST kinase domains 1
2.03	TMTC3	transmembrane and tetratricopeptide repeat containing 3
2.02	ADAT2	adenosine deaminase. tRNA-specific 2
2.02	ago-02	argonaute RISC catalytic component 2
2.02	TMEM167B	transmembrane protein 167B
2.01	DCAF10	DDB1 and CUL4 associated factor 10
2.01	RAB3GAP1	RAB3 GTPase activating protein subunit 1 (catalytic)
2.00	GBE1	glucan (1.4-alpha-). branching enzyme 1
2.00	CLIP2	CAP-GLY domain containing linker protein 2
2.00	SOWAHC	sosondowah ankyrin repeat domain family member C
2.00	NEK3	NIMA-related kinase 3
2.00	IFRD1	interferon-related developmental regulator 1
2.00	TBPL1	TBP-like 1
1.99	ZNHIT6	zinc finger. HIT-type containing 6
1.98	KPNA7	karyopherin alpha 7 (importin alpha 8)
1.98	EFNA2	ephrin-A2
1.98	CLDN12	claudin 12
1.98	TEAD4	TEA domain family member 4
1.98	EPS15	epidermal growth factor receptor pathway substrate 15
1.98	SERINC2	serine incorporator 2
1.97	PLIN2	perilipin 2

1.97	RHOT1	ras homolog family member T1
1.97	SLC9A1	solute carrier family 9. subfamily A (NHE1. cation proton antiporter 1). member 1
1.97	RAD54L2	RAD54-like 2 (S. cerevisiae)
1.96	ZCCHC4	zinc finger. CCHC domain containing 4
1.96	GDI1	GDP dissociation inhibitor 1
1.96	TMSB10	thymosin beta 10
1.96	C11orf74	chromosome 11 open reading frame 74
1.95	SEL1L3	sel-1 suppressor of lin-12-like 3 (C. elegans)
1.95	AHI1	Abelson helper integration site 1
1.95	LDLRAD1	low density lipoprotein receptor class A domain containing 1
1.95	STX3	syntaxin 3
1.95	CLCF1	cardiotrophin-like cytokine factor 1
1.95	HNF4G	hepatocyte nuclear factor 4. gamma
1.95	WDPCP	WD repeat containing planar cell polarity effector
1.94	CDK8	cyclin-dependent kinase 8
1.94	CORO2B	coronin. actin binding protein. 2B
1.94	ASAP2	ArfGAP with SH3 domain. ankyrin repeat and PH domain 2
1.93	KYNU	kynureninase
1.92	RAB3B	RAB3B. member RAS oncogene family
1.92	ZFP36	ZFP36 ring finger protein
1.92	GAREM1	GRB2 associated regulator of MAPK1 1
1.92	SLC35A1	solute carrier family 35 (CMP-sialic acid transporter). member A1
1.92	FHL3	four and a half LIM domains 3
1.92	SAV1	salvador family WW domain containing protein 1
1.92	NABP1	nucleic acid binding protein 1

1.92	PIK3CB	phosphatidylinositol-4.5-bisphosphate 3-kinase. catalytic subunit beta
1.92	DPH5	diphthamide biosynthesis 5
1.91	AIFM2	apoptosis-inducing factor. mitochondrion-associated. 2
1.91	SLC7A11	solute carrier family 7 (anionic amino acid transporter light chain. xc-system). member 11
1.91	VLDLR	very low density lipoprotein receptor
1.91	STEAP1	six transmembrane epithelial antigen of the prostate 1
1.90	MT1G	metallothionein 1G
1.90	PHLDA1	pleckstrin homology-like domain. family A. member 1
1.89	PXDC1	PX domain containing 1
1.89	USP12	ubiquitin specific peptidase 12
1.89	SGCB	sarcoglycan beta
1.89	RAI14	retinoic acid induced 14
1.89	PRKAA2	protein kinase. AMP-activated. alpha 2 catalytic subunit
1.89	MSANTD3-TMEFF1	MSANTD3-TMEFF1 readthrough
1.88	TNFRSF12A	tumor necrosis factor receptor superfamily. member 12A
1.88	ZSWIM6	zinc finger. SWIM-type containing 6
1.88	PLK3	polo-like kinase 3
1.88	MRPL39	mitochondrial ribosomal protein L39
1.87	CDYL	chromodomain protein. Y-like
1.87	CSRNP1	cysteine-serine-rich nuclear protein 1
1.87	LTBP2	latent transforming growth factor beta binding protein 2
1.87	TMEFF1	transmembrane protein with EGF-like and two follistatin-like domains 1
1.86	SERTAD1	SERTA domain containing 1
1.86	C4orf36	chromosome 4 open reading frame 36
1.85	BCL2A1	BCL2-related protein A1

1.85	SH3BP2	SH3-domain binding protein 2
1.85	DEF6	Memczak2013 ANTISENSE. CDS. coding. INTERNAL best transcript NM_022047
1.85	EPHA2	EPH receptor A2
1.85	TMEM136	transmembrane protein 136
1.85	ABLIM3	actin binding LIM protein family. member 3
1.85	RUSC2	RUN and SH3 domain containing 2
1.84	C3orf52	chromosome 3 open reading frame 52
1.84	PTPDC1	protein tyrosine phosphatase domain containing 1
1.84	MGAT4B	mannosyl (alpha-1.3-)-glycoprotein beta-1.4-N-acetylglucosaminyltransferase. isozyme B
1.84	CPEB4	cytoplasmic polyadenylation element binding protein 4
1.84	CCNJL	cyclin J-like
1.84	ANKDD1B	ankyrin repeat and death domain containing 1B
1.84	SCP2	Jeck2013 ANTISENSE. coding. INTERNAL. OVEXON. UTR3 best transcript NM_001007098
1.84	LMO2	LIM domain only 2 (rhombotin-like 1)
1.83	MNAT1	MNAT CDK-activating kinase assembly factor 1
1.83	CASP10	caspase 10
1.83	DYRK4	dual specificity tyrosine-(Y)-phosphorylation regulated kinase 4
1.83	C17orf75	chromosome 17 open reading frame 75
1.83	SQSTM1	sequestosome 1
1.83	GPR3	G protein-coupled receptor 3
1.82	HSPA13	heat shock protein 70kDa family. member 13
1.82	SLC7A1	solute carrier family 7 (cationic amino acid transporter. y+ system). member 1
1.82	CRBN	cereblon

1.82	ABCB7	ATP binding cassette subfamily B member 7
1.82	GTF3C3	general transcription factor IIIC subunit 3
1.82	NDRG4	NDRG family member 4
1.82	C4orf32	chromosome 4 open reading frame 32
1.82	LTA4H	leukotriene A4 hydrolase
1.82	IER2	immediate early response 2
1.82	DNAJC12	DnaJ (Hsp40) homolog. subfamily C. member 12
1.82	FAF1	Fas (TNFRSF6) associated factor 1
1.81	ZMYM6NB; ZMYM6	ZMYM6 neighbor; zinc finger. MYM-type 6
1.81	CYP2S1	cytochrome P450. family 2. subfamily S. polypeptide 1
1.81	TXNRD1	thioredoxin reductase 1
1.81	ELF4	E74-like factor 4 (ets domain transcription factor)
1.81	INPP1	inositol polyphosphate-1-phosphatase
1.81	METRNL	meteorin. glial cell differentiation regulator-like
1.81	ZFAND1	zinc finger. AN1-type domain 1
1.81	WDR75	WD repeat domain 75
1.81	ITPKA	inositol-trisphosphate 3-kinase A
1.81	AGK	acylglycerol kinase
1.80	PTPRH	protein tyrosine phosphatase. receptor type. H
1.80	BAIAP2L1	BAI1-associated protein 2-like 1
1.79	SLC35F2	solute carrier family 35. member F2
1.79	IKBKG	inhibitor of kappa light polypeptide gene enhancer in B-cells. kinase gamma
1.79	RAB12	RAB12. member RAS oncogene family
1.79	NEK1	NIMA-related kinase 1
1.79	HNRNPA1L2	heterogeneous nuclear ribonucleoprotein A1-like 2
1.79	ICT1	immature colon carcinoma transcript 1

1.78	DUSP1	dual specificity phosphatase 1
1.78	LONRF3	LON peptidase N-terminal domain and ring finger 3
1.78	ADGRE5	adhesion G protein-coupled receptor E5
1.78	YBX3	Y box binding protein 3
1.78	CLCN3	chloride channel. voltage-sensitive 3
1.78	OAS1	2-5-oligoadenylate synthetase 1
1.77	GPR137B	G protein-coupled receptor 137B
1.77	MAPK13	mitogen-activated protein kinase 13
1.77	FARSB	phenylalanyl-tRNA synthetase beta subunit
1.77	CSRNP2	cysteine-serine-rich nuclear protein 2
1.77	SLC4A11	solute carrier family 4. sodium borate transporter. member 11
1.77	IPO7; SNORA23	importin 7; small nucleolar RNA. H/ACA box 23
1.76	WDR7	WD repeat domain 7
1.76	ACVR1	activin A receptor type I
1.76	UCHL3	ubiquitin C-terminal hydrolase L3
1.76	BEND7	BEN domain containing 7
1.76	PLK2	polo-like kinase 2
1.76	CAST	calpastatin
1.76	GPR83	G protein-coupled receptor 83
1.75	PPP3CC	protein phosphatase 3. catalytic subunit. gamma isozyme
1.75	RND1	Rho family GTPase 1
1.75	PHLDA2	pleckstrin homology-like domain. family A. member 2
1.75	PLEKHO1	pleckstrin homology domain containing. family O member 1
1.75	OTUD4	OTU deubiquitinase 4
1.75	TP53I3	tumor protein p53 inducible protein 3
1.75	WWP1	Transcript Identified by AceView. Entrez Gene ID(s) 11059

1.74	SH2D5	SH2 domain containing 5
1.74	STEAP1B	STEAP family member 1B
1.74	RUFY3	RUN and FYVE domain containing 3
1.74	SKIL	SKI-like proto-oncogene
1.74	SLC7A6OS	solute carrier family 7. member 6 opposite strand
1.73	FLNA	filamin A. alpha
1.73	CLMN	calmin (calponin-like. transmembrane)
1.73	SLC35A3	solute carrier family 35 (UDP-N-acetylglucosamine (UDP-GlcNAc) transporter). member A3
1.73	UBASH3B	ubiquitin associated and SH3 domain containing B
1.73	GOLT1A	golgi transport 1A
1.73	STK39	serine threonine kinase 39
1.73	SPRY1	sprouty RTK signaling antagonist 1
1.73	RCAN3	RCAN family member 3
1.73	MVP; PAGR1	major vault protein; PAXIP1 associated glutamate-rich protein 1
1.72	RAD52	RAD52 homolog. DNA repair protein
1.72	MMP10	matrix metallopeptidase 10
1.72	QPCT	glutaminyl-peptide cyclotransferase
1.72	PDE9A	phosphodiesterase 9A
1.72	RBM28	RNA binding motif protein 28
1.72	RANBP6	RAN binding protein 6
1.72	ABI2	abl-interactor 2
1.72	BAZ1A	bromodomain adjacent to zinc finger domain 1A
1.72	VPS50	VPS50 EARP/GARPII complex subunit
1.71	MTHFD2	methylenetetrahydrofolate dehydrogenase (NADP+ dependent) 2. methenyltetrahydrofolate cyclohydrolase
1.71	CDA	cytidine deaminase

1.71	TTC27; MIR4765	tetratricopeptide repeat domain 27; microRNA 4765
1.71	MIOS	missing oocyte. meiosis regulator. homolog (Drosophila)
1.71	GTPBP8	GTP-binding protein 8 (putative)
1.71	CFAP97	cilia and flagella associated protein 97
1.71	SPRED1	sprouty-related. EVH1 domain containing 1
1.70	GAB2	GRB2-associated binding protein 2
1.70	NDUFAF4	NADH dehydrogenase (ubiquinone) complex I. assembly factor 4
1.70	RBM23	RNA binding motif protein 23
1.70	LGALS8	lectin. galactoside-binding. soluble. 8
1.70	NDUFA5	NADH dehydrogenase (ubiquinone) 1 alpha subcomplex. 5
1.70	OSGIN1	oxidative stress induced growth inhibitor 1
1.70	POLR3G	polymerase (RNA) III (DNA directed) polypeptide G (32kD)
1.70	MXI1	MAX interactor 1. dimerization protein
1.70	CES1P1; CES1	carboxylesterase 1 pseudogene 1; carboxylesterase 1
1.69	QSOX1	quiescin Q6 sulfhydryl oxidase 1
1.69	SMG8	SMG8 nonsense mediated mRNA decay factor
1.69	CYGB	cytoglobin
1.69	PLEKHM1; MIR4315-1	pleckstrin homology domain containing. family M (with RUN domain) member 1; microRNA 4315-1
1.69	NR3C1	nuclear receptor subfamily 3. group C. member 1 (glucocorticoid receptor)
1.69	MAB21L3	mab-21-like 3 (C. elegans)
1.69	ARL14EP	ADP-ribosylation factor like GTPase 14 effector protein
1.69	FABP4	fatty acid binding protein 4. adipocyte
1.69	AAK1	AP2 associated kinase 1
1.69	DPY19L1	dpy-19-like 1 (C. elegans)
1.69	DAAM1	dishevelled associated activator of morphogenesis 1

1.69	CHD2; MIR3175	chromodomain helicase DNA binding protein 2; microRNA 3175
1.69	SAT1	spermidine/spermine N1-acetyltransferase 1
1.69	MTMR1	myotubularin related protein 1
1.68	STOM	stomatin
1.68	PPP2CB	protein phosphatase 2. catalytic subunit. beta isozyme
1.68	FERMT2	fermitin family member 2
1.68	FEZ2	fasciculation and elongation protein zeta 2 (zygin II)
1.68	PFKP	phosphofructokinase. platelet
1.68	COX7A2L	cytochrome c oxidase subunit VIIa polypeptide 2 like
1.67	SMOX	spermine oxidase
1.67	SLC7A2	solute carrier family 7 (cationic amino acid transporter. y+ system). member 2
1.67	CSNK2A2	casein kinase 2. alpha prime polypeptide
1.67	RBPMS2	RNA binding protein with multiple splicing 2
1.67	S100A16	S100 calcium binding protein A16
1.67	ZC3H12C	zinc finger CCCH-type containing 12C
1.67	DNAJC10	DnaJ (Hsp40) homolog. subfamily C. member 10
1.67	TTL	tubulin tyrosine ligase
1.67	ARFGEF1	ADP-ribosylation factor guanine nucleotide-exchange factor 1 (brefeldin A-inhibited)
1.67	PPRC1	peroxisome proliferator-activated receptor gamma. coactivator-related 1
1.67	P2RX5	purinergic receptor P2X. ligand gated ion channel. 5
1.66	SERPINA3	serpin peptidase inhibitor. clade A (alpha-1 antiproteinase. antitrypsin). member 3
1.66	WEE1	WEE1 G2 checkpoint kinase
1.66	MARS; MIR6758	methionyl-tRNA synthetase; microRNA 6758
1.66	WWC3	WWC family member 3

1.66	TERF2IP	telomeric repeat binding factor 2. interacting protein
1.66	WDR47	WD repeat domain 47
1.66	UBL3	ubiquitin-like 3
1.66	EIF3J	eukaryotic translation initiation factor 3. subunit J
1.66	NAA30	N(alpha)-acetyltransferase 30. NatC catalytic subunit
1.65	EIF2B3	eukaryotic translation initiation factor 2B. subunit 3 gamma. 58kDa
1.65	MT1F	metallothionein 1F
1.65	TACO1	translational activator of mitochondrially encoded cytochrome c oxidase I
1.65	GLRX2	glutaredoxin 2
1.65	COMMD10	COMM domain containing 10
1.65	DNAH14	dynein. axonemal. heavy chain 14
1.65	SLC35D2	solute carrier family 35 (UDP-GlcNAc/UDP-glucose transporter). member D2
1.64	GCLM	glutamate-cysteine ligase. modifier subunit
1.64	ALKBH8	alkB homolog 8. tRNA methyltransferase
1.64	TNFRSF21	tumor necrosis factor receptor superfamily. member 21
1.64	BCL7A	B-cell CLL/lymphoma 7A
1.64	SNAI1	snail family zinc finger 1
1.64	SEPSECS	Sep (O-phosphoserine) tRNA:Sec (selenocysteine) tRNA synthase
1.64	HDLBP	Jeck2013 ANTISENSE. coding. INTERNAL. intronic best transcript NM_005336
1.64	SLC39A10	Transcript Identified by AceView. Entrez Gene ID(s) 57181
1.64	THEM4	thioesterase superfamily member 4
1.64	S100A6	S100 calcium binding protein A6
1.64	ACSF3	acyl-CoA synthetase family member 3
1.64	PUS7	pseudouridylate synthase 7 (putative)
1.63	BOLA3	bolA family member 3
1.63	PEA15	phosphoprotein enriched in astrocytes 15

1.63	FER	fer (fps/fes related) tyrosine kinase
1.63	KIAA0586	KIAA0586
1.63	AGTPBP1	ATP/GTP binding protein 1
1.63	FEM1B	fem-1 homolog b (C. elegans)
1.63	MTMR11	myotubularin related protein 11
1.63	MMGT1	membrane magnesium transporter 1
1.63	SERPINB6	serpin peptidase inhibitor. clade B (ovalbumin). member 6
1.62	COMMD2	COMM domain containing 2
1.62	BRAF	B-Raf proto-oncogene. serine/threonine kinase
1.62	SAV1	salvador family WW domain containing protein 1
1.62	SPECC1	sperm antigen with calponin homology and coiled-coil domains 1
1.62	ZCCHC8	zinc finger. CCHC domain containing 8
1.62	REEP2	receptor accessory protein 2
1.62	SLC4A7	solute carrier family 4. sodium bicarbonate cotransporter. member 7
1.62	RINT1	RAD50 interactor 1
1.62	DST	dystonin
1.62	ZDHHC18	zinc finger. DHHC-type containing 18
1.62	BDP1	B double prime 1. subunit of RNA polymerase III transcription initiation factor IIIB
1.62	KMT5B	lysine (K)-specific methyltransferase 5B
1.62	POLK	polymerase (DNA directed) kappa
1.62	ZNF501	zinc finger protein 501 [Source:HGNC Symbol;Acc:HGNC:23717]
1.62	MKLN1	muskelin 1. intracellular mediator containing kelch motifs
1.62	STAM	signal transducing adaptor molecule (SH3 domain and ITAM motif) 1
1.62	MYO5A	myosin VA
1.61	DCP1B	decapping mRNA 1B

1.61	GABPB1	GA binding protein transcription factor. beta subunit 1
1.61	NMNAT3	nicotinamide nucleotide adenylyltransferase 3
1.61	MYO9A	myosin IXA
1.61	SH3GLB1	SH3-domain GRB2-like endophilin B1
1.61	FYCO1	FYVE and coiled-coil domain containing 1
1.61	PLP2	proteolipid protein 2 (colonic epithelium-enriched)
1.61	MTMR2	myotubularin related protein 2
1.61	GPX2	glutathione peroxidase 2
1.61	KRT12	keratin 12. type I
1.61	ORM1	orosomucoid 1
1.60	JAK2	Janus kinase 2
1.60	GMFG	glia maturation factor. gamma
1.60	NFIB	nuclear factor I/B
1.60	TES	testin LIM domain protein
1.60	MTF2	metal response element binding transcription factor 2
1.60	HEXB	hexosaminidase B (beta polypeptide)
1.60	OSCAR	osteoclast associated. immunoglobulin-like receptor
1.60	NPM3	nucleophosmin/nucleoplasmin 3
1.60	HES1	hes family bHLH transcription factor 1
1.60	UTP14A	UTP14A small subunit (SSU) processome component
1.60	RPGR	retinitis pigmentosa GTPase regulator
1.60	CALCOCO1	calcium binding and coiled-coil domain 1
1.60	GNAZ	guanine nucleotide binding protein (G protein). alpha z polypeptide
1.60	FLNA	Jeck2013 ANTISENSE. CDS. coding. INTERNAL. OVCODE. OVEXON best transcript NM_001110556
1.60	STAMBPL1	STAM binding protein-like 1

1.60	RASGRF2	Ras protein-specific guanine nucleotide-releasing factor 2
1.60	POLR3F	polymerase (RNA) III (DNA directed) polypeptide F. 39 kDa
1.60	R3HCC1	R3H domain and coiled-coil containing 1
1.60	KATNBL1	katanin p80 subunit B-like 1
1.60	CDC42BPB	CDC42 binding protein kinase beta (DMPK-like)
1.60	JUN	jun proto-oncogene
1.60	TBCK	Memczak2013 ALT_ACCEPTOR. ALT_DONOR. coding. INTERNAL. intronic best transcript NM_001163435
1.60	CDC16	cell division cycle 16
1.60	ZNF883	zinc finger protein 883
1.59	CASC3; MIR6866	cancer susceptibility candidate 3; microRNA 6866
1.59	sept-15	15 kDa selenoprotein; Salzman2013 ANNOTATED. CDS. coding. INTERNAL. OVCODE. OVEXON best transcript NM_004261; Transcript Identified by AceView. Entrez Gene ID(s) 9403. RefSeq ID(s) NM_203341; Transcript Identified by AceView. Entrez Gene ID(s) 9403. RefSeq ID(s) NM_004261; 15 kDa selenoprotein [Source:EntrezGene;Acc:9403]
1.59	EMP1	epithelial membrane protein 1
1.59	LYVE1	lymphatic vessel endothelial hyaluronan receptor 1
1.59	FERMT1	fermitin family member 1
1.59	CSNK1G3	casein kinase 1. gamma 3
1.59	TRMT12	tRNA methyltransferase 12 homolog (S. cerevisiae)
1.59	BCS1L	BCS1 homolog. ubiquinol-cytochrome c reductase complex chaperone
1.59	ZNF383	zinc finger protein 383
1.59	ZUFSP	zinc finger with UFM1-specific peptidase domain
1.59	RIOK3	RIO kinase 3
1.59	PLSCR4	phospholipid scramblase 4
1.59	CPEB2	cytoplasmic polyadenylation element binding protein 2
1.59	FBXO17	F-box protein 17

1.59	TEX37	testis expressed 37
1.59	PLCB1	phospholipase C. beta 1 (phosphoinositide-specific)
1.59	ANKLE2	ankyrin repeat and LEM domain containing 2
1.59	EIF4G3	eukaryotic translation initiation factor 4 gamma. 3
1.58	PPP2R5E	protein phosphatase 2. regulatory subunit B. epsilon isoform
1.58	DYNC1I2	dynein. cytoplasmic 1. intermediate chain 2
1.58	TMEM261	transmembrane protein 261
1.58	RGS10	regulator of G-protein signaling 10
1.58	SPSB1	splA/ryanodine receptor domain and SOCS box containing 1
1.58	AMPD3	adenosine monophosphate deaminase 3
1.58	LINC00494	long intergenic non-protein coding RNA 494
1.58	MLXIP	MLX interacting protein
1.58	MCL1	myeloid cell leukemia 1
1.58	ZFAND2A	zinc finger. AN1-type domain 2A
1.58	TRAF3	TNF receptor-associated factor 3
1.58	CHMP4C	charged multivesicular body protein 4C
1.58	CYR61	cysteine-rich. angiogenic inducer. 61
1.58	PRNP	prion protein
1.58	EREG	epiregulin
1.58	LOXL4	lysyl oxidase-like 4
1.58	AMMECR1L	AMMECR1 like
1.58	DNAJC2	DnaJ (Hsp40) homolog. subfamily C. member 2
1.57	PVRL1	poliovirus receptor-related 1 (herpesvirus entry mediator C)
1.57	MICALL1	MICAL-like 1
1.57	PAPOLG	poly(A) polymerase gamma
1.57	ARMCX5-GPRASP2	ARMCX5-GPRASP2 readthrough

1.57	DENND4B	DENN/MADD domain containing 4B
1.57	INTS7	integrator complex subunit 7
1.57	CYP27C1	cytochrome P450. family 27. subfamily C. polypeptide 1
1.57	ARL6	ADP-ribosylation factor like GTPase 6
1.57	TSPAN7	tetraspanin 7
1.57	FLVCR2	feline leukemia virus subgroup C cellular receptor family. member 2
1.57	MAL	mal. T-cell differentiation protein
1.57	UGCG	UDP-glucose ceramide glucosyltransferase
1.57	MLLT3	Jeck2013 ALT_ACCEPTOR. ALT_DONOR. coding. INTERNAL. intronic best transcript NM_004529
1.57	USP31	ubiquitin specific peptidase 31
1.57	KIF21A	kinesin family member 21A
1.57	MTHFD1L	methylenetetrahydrofolate dehydrogenase (NADP+ dependent) 1-like
1.57	ERBB2IP	erbb2 interacting protein
1.56	MIR6809; TNS1	microRNA 6809; tensin 1
1.56	ABCA12	ATP binding cassette subfamily A member 12
1.56	EPDR1	ependymin related 1
1.56	LMO4	LIM domain only 4
1.56	HADHA	Transcript Identified by AceView. Entrez Gene ID(s) 3030
1.56	SPTBN1	spectrin. beta. non-erythrocytic 1
1.56	RNF2	ring finger protein 2
1.56	SERTAD2	SERTA domain containing 2
1.56	GPN1	GPN-loop GTPase 1
1.56	NOC3L	NOC3-like DNA replication regulator
1.56	EDA2R	ectodysplasin A2 receptor
1.56	RASAL2	RAS protein activator like 2

1.56	ABCF2	ATP binding cassette subfamily F member 2
1.56	REPS1	RALBP1 associated Eps domain containing 1
1.56	ZBTB34	zinc finger and BTB domain containing 34
1.56	ASNS	asparagine synthetase (glutamine-hydrolyzing)
1.56	ATXN3	ataxin 3
1.56	BNIP1	BCL2/adenovirus E1B 19kDa interacting protein 1
1.56	TINAGL1	tubulointerstitial nephritis antigen-like 1
1.56	COMMD7	COMM domain containing 7
1.56	ZNF131	zinc finger protein 131
1.55	NOSTRIN	nitric oxide synthase trafficking
1.55	CMTM3	CKLF-like MARVEL transmembrane domain containing 3
1.55	FUZ	fuzzy planar cell polarity protein
1.55	ETS2	v-ets avian erythroblastosis virus E26 oncogene homolog 2
1.55	NDRG1	N-myc downstream regulated 1
1.55	RIOK2	RIO kinase 2
1.55	IL2RG	interleukin 2 receptor. gamma
1.55	GALNT11	polypeptide N-acetylgalactosaminyltransferase 11
1.55	ZNF654	zinc finger protein 654
1.55	LPCAT2	lysophosphatidylcholine acyltransferase 2
1.55	S100A13	S100 calcium binding protein A13
1.55	ZNF560	zinc finger protein 560
1.55	NSUN4	NOP2/Sun domain family. member 4
1.55	ACADVL	acyl-CoA dehydrogenase. very long chain
1.55	GNA13	guanine nucleotide binding protein (G protein). alpha 13
1.55	SEMA4C	sema domain. immunoglobulin domain (Ig). transmembrane domain (TM) and short cytoplasmic domain. (semaphorin) 4C

1.55	UCKL1; MIR1914	uridine-cytidine kinase 1-like 1; microRNA 1914
1.54	IMPDH2	IMP (inosine 5-monophosphate) dehydrogenase 2
1.54	VPS37B	vacuolar protein sorting 37 homolog B (S. cerevisiae)
1.54	CAPN8	calpain 8
1.54	SF3A3	splicing factor 3a subunit 3
1.54	GTF2E2	general transcription factor IIE subunit 2
1.54	AZI2	5-azacytidine induced 2
1.54	SGTB	small glutamine-rich tetratricopeptide repeat (TPR)-containing. beta
1.54	CBL	Cbl proto-oncogene. E3 ubiquitin protein ligase
1.54	CHSY1	chondroitin sulfate synthase 1
1.54	TCP11L1	t-complex 11. testis-specific-like 1
1.54	TMA16	translation machinery associated 16 homolog
1.54	SLC3A2	solute carrier family 3 (amino acid transporter heavy chain). member 2
1.54	DRAM2	DNA-damage regulated autophagy modulator 2
1.54	SLC27A4	solute carrier family 27 (fatty acid transporter). member 4
1.54	APOOL	apolipoprotein O-like
1.54	STK17A	serine/threonine kinase 17a
1.54	CYCS	cytochrome c. somatic
1.54	ADAMTS16	ADAM metallopeptidase with thrombospondin type 1 motif 16
1.54	RICTOR	RPTOR independent companion of MTOR. complex 2
1.54	C12orf73	chromosome 12 open reading frame 73
1.54	MYC	v-myc avian myelocytomatosis viral oncogene homolog
1.54	CDC73	cell division cycle 73
1.54	AIMP1	aminoacyl tRNA synthetase complex-interacting multifunctional protein 1
1.54	SPOCD1	SPOC domain containing 1
1.54	ZDHHC6	zinc finger. DHHC-type containing 6

1.54	FBXL3	F-box and leucine-rich repeat protein 3
1.53	CLDND1	claudin domain containing 1
1.53	DUSP16	dual specificity phosphatase 16
1.53	SATB2	SATB homeobox 2
1.53	PTEN	phosphatase and tensin homolog
1.53	SMIM13	small integral membrane protein 13
1.53	METTL15	methyltransferase like 15
1.53	VPS51	vacuolar protein sorting 51 homolog (S. cerevisiae)
1.53	SHB	Src homology 2 domain containing adaptor protein B
1.53	PAK1	p21 protein (Cdc42/Rac)-activated kinase 1
1.53	ID3	inhibitor of DNA binding 3. dominant negative helix-loop-helix protein
1.53	KLHL23	kelch-like family member 23
1.53	PIK3CB	Salzman2013 ANNOTATED. CDS. coding. INTERNAL. OVCODE. OVEXON best transcript NM_006219
1.53	AMBRA1	autophagy/beclin-1 regulator 1
1.53	LRP10	LDL receptor related protein 10
1.53	QRSL1	glutaminyl-tRNA synthase (glutamine-hydrolyzing)-like 1
1.53	ARL5C	ADP-ribosylation factor like GTPase 5C
1.53	ZBTB8A	zinc finger and BTB domain containing 8A
1.53	ZEB1	zinc finger E-box binding homeobox 1
1.53	RP9	retinitis pigmentosa 9 (autosomal dominant)
1.53	CBX6	chromobox homolog 6
1.53	RASA1	RAS p21 protein activator (GTPase activating protein) 1
1.53	OSGIN2	oxidative stress induced growth inhibitor family member 2
1.53	ITIH3	inter-alpha-trypsin inhibitor heavy chain 3
1.53	MBD3L1	methyl-CpG binding domain protein 3-like 1

1.53	KIF1BP	KIF1 binding protein
1.53	UBE2D1	ubiquitin conjugating enzyme E2D 1
1.53	RAD17	RAD17 checkpoint clamp loader component
1.53	CTSS	cathepsin S
1.53	TRIM13; KCNRG	tripartite motif containing 13; potassium channel regulator
1.53	PRAMEF2	PRAME family member 2
1.53	SLC40A1	solute carrier family 40 (iron-regulated transporter). member 1
1.53	RPL36A	ribosomal protein L36a
1.53	ENAH	enabled homolog (Drosophila)
1.52	LOC389602; AC021218.2	uncharacterized LOC389602; novel transcript
1.52	FKBP4	FK506 binding protein 4
1.52	METTL18	methyltransferase like 18
1.52	SNX25	sorting nexin 25
1.52	PNPT1	polyribonucleotide nucleotidyltransferase 1
1.52	RPL7	ribosomal protein L7
1.52	SLC25A12	solute carrier family 25 (aspartate/glutamate carrier). member 12
1.52	THOC7	THO complex 7
1.52	STX8	syntaxin 8
1.52	HEATR1	HEAT repeat containing 1
1.52	STRN	striatin. calmodulin binding protein
1.52	SLC25A24	solute carrier family 25 (mitochondrial carrier; phosphate carrier). member 24
1.52	MAFK	v-maf avian musculoaponeurotic fibrosarcoma oncogene homolog K
1.52	PALM2-AKAP2	PALM2-AKAP2 readthrough
1.52	NEK7	NIMA-related kinase 7
1.52	PCID2	PCI domain containing 2

1.52	ZNF37A	zinc finger protein 37A
1.52	GABRE; MIR224; MIR452	gamma-aminobutyric acid (GABA) A receptor. epsilon; microRNA 224; microRNA 452
1.52	CHN1	chimerin 1
1.52	MTMR10	myotubularin related protein 10
1.52	PNMA1	paraneoplastic Ma antigen 1
1.52	DSTYK	dual serine/threonine and tyrosine protein kinase
1.52	RHPN2	rhophilin. Rho GTPase binding protein 2
1.52	ROCK2	Rho-associated. coiled-coil containing protein kinase 2
1.52	VPS72	vacuolar protein sorting 72 homolog (S. cerevisiae)
1.52	TMEM242	transmembrane protein 242
1.52	MOB3A	MOB kinase activator 3A
1.52	PFDN2	prefoldin subunit 2
1.52	DCBLD2	discoidin. CUB and LCCL domain containing 2
1.52	PEX3	peroxisomal biogenesis factor 3
1.51	ACOT9	acyl-CoA thioesterase 9
1.51	WARS	tryptophanyl-tRNA synthetase
1.51	ZNF781	zinc finger protein 781
1.51	FXR1	fragile X mental retardation. autosomal homolog 1
1.51	DUS4L	dihydrouridine synthase 4-like
1.51	KCMF1	potassium channel modulatory factor 1
1.51	CRCP	CGRP receptor component
1.51	C5orf30	chromosome 5 open reading frame 30
1.51	TMEM128	transmembrane protein 128
1.51	FNBP1L	formin binding protein 1-like
1.51	CCNI	cyclin I

1.51	STON2	stonin 2
1.51	SLX4IP	SLX4 interacting protein
1.51	IRF2BPL	interferon regulatory factor 2 binding protein-like
1.51	SPAST	spastin
1.51	HAX1	HCLS1 associated protein X-1
1.51	LACE1	lactation elevated 1
1.51	PABPC1L	poly(A) binding protein. cytoplasmic 1-like
1.51	PRSS8	protease. serine. 8
1.51	A2BP1	Transcript Identified by AceView. Entrez Gene ID(s) 54715
1.50	SCRN1	secernin 1
1.50	HPD	4-hydroxyphenylpyruvate dioxygenase
1.50	SURF2	surfeit 2 [Source:HGNC Symbol;Acc:HGNC:11475]
1.50	RPL5	Memczak2013 ALT_ACCEPTOR. ALT_DONOR. coding. INTERNAL. intronic best transcript NM_000969
1.50	DCUN1D4	DCN1. defective in cullin neddylation 1. domain containing 4
1.50	FDXR	Zhang2013 ALT_ACCEPTOR. ALT_DONOR. coding. INTERNAL. intronic best transcript NM_004110
1.50	MEMO1	mediator of cell motility 1
1.50	RPL7L1	ribosomal protein L7-like 1
1.50	GTF2IRD1	GTF2I repeat domain containing 1
1.50	MBNL1	muscleblind like splicing regulator 1
1.50	SPRED2	sprouty-related. EVH1 domain containing 2
1.50	ARL1	ADP-ribosylation factor like GTPase 1
1.50	ADGRL2	adhesion G protein-coupled receptor L2
1.50	PVR	poliovirus receptor
1.50	MARS2	methionyl-tRNA synthetase 2. mitochondrial
1.50	FNTA	farnesyltransferase. CAAX box. alpha

1.50	PRKCI	protein kinase C. iota
1.50	VPS54	vacuolar protein sorting 54 homolog (S. cerevisiae)
1.50	NDUFAF5	NADH dehydrogenase (ubiquinone) complex I. assembly factor 5
1.50	LAMA1	laminin. alpha 1
1.50	RAB2B	RAB2B. member RAS oncogene family
1.50	UTP6	UTP6. small subunit (SSU) processome component. homolog (yeast)
1.50	RNF111	ring finger protein 111
1.50	AASDHPPT	aminoadipate-semialdehyde dehydrogenase-phosphopantetheinyl transferase
1.50	SPTSSB	serine palmitoyltransferase. small subunit B
1.50	UBE2E2	ubiquitin conjugating enzyme E2E 2
1.50	TTC39B	tetratricopeptide repeat domain 39B
1.50	KIAA1549	KIAA1549
1.50	CHIC2	cysteine rich hydrophobic domain 2
1.50	LY96	lymphocyte antigen 96
1.50	MTERF3	mitochondrial transcription termination factor 3
1.50	TIMM21	translocase of inner mitochondrial membrane 21 homolog (yeast)
1.50	CHUK	conserved helix-loop-helix ubiquitous kinase
1.50	MLXIP	MLX interacting protein
1.50	TMEM2	transmembrane protein 2
1.50	CCDC88A	coiled-coil domain containing 88A
1.50	HP	haptoglobin
1.50	ZDHHC1	zinc finger. DHHC-type containing 1
1.50	PPP1CC	protein phosphatase 1. catalytic subunit. gamma isozyme
1.49	PSMD3	proteasome 26S subunit. non-ATPase 3
1.49	RSPH3	radial spoke 3 homolog (Chlamydomonas)

1.49	UBE2D4	ubiquitin-conjugating enzyme E2D 4 (putative)
1.49	EIF2S1	eukaryotic translation initiation factor 2. subunit 1 alpha. 35kDa
1.49	MYADM	Memczak2013 ANTISENSE. CDS. coding. INTERNAL best transcript NM_001020819
1.49	CXCR2	chemokine (C-X-C motif) receptor 2
1.49	FAM65A	family with sequence similarity 65. member A
1.49	KIAA1324L	KIAA1324-like
1.49	HNRNPCL1	heterogeneous nuclear ribonucleoprotein C-like 1
1.49	MFHAS1	malignant fibrous histiocytoma amplified sequence 1
1.49	BCAR1	breast cancer anti-estrogen resistance 1
1.49	SMURF1	SMAD specific E3 ubiquitin protein ligase 1
1.49	NAP1L1	nucleosome assembly protein 1-like 1
1.49	LAMB1	laminin. beta 1
1.49	PLEKHA6	Transcript Identified by AceView. Entrez Gene ID(s) 22874
1.49	KRTDAP	keratinocyte differentiation-associated protein
1.49	PSMA1	proteasome subunit alpha 1
1.49	DNASE1L2	deoxyribonuclease I-like 2
1.49	PTHLH	parathyroid hormone-like hormone
1.49	RAB10	RAB10. member RAS oncogene family
1.49	SBDS	Shwachman-Bodian-Diamond syndrome
1.49	GLRX	glutaredoxin
1.49	KIAA0368	KIAA0368
1.49	SLC19A1	solute carrier family 19 (folate transporter). member 1
1.49	CAP2	CAP. adenylate cyclase-associated protein. 2 (yeast)
1.49	PARD3	par-3 family cell polarity regulator
1.49	BHLHE41	basic helix-loop-helix family. member e41

1.49	TRIM38	tripartite motif containing 38
1.49	PTPRU	protein tyrosine phosphatase. receptor type. U
1.49	BTBD3	BTB (POZ) domain containing 3
1.49	ZBTB26	zinc finger and BTB domain containing 26
1.49	VRK2	vaccinia related kinase 2
1.49	DOCK5	dedicator of cytokinesis 5
1.49	RIF1	replication timing regulatory factor 1
1.49	SEMA3B; MIR6872	sema domain. immunoglobulin domain (Ig). short basic domain. secreted. (semaphorin) 3B; microRNA 6872
1.48	CCDC138	coiled-coil domain containing 138
1.48	OGT	O-linked N-acetylglucosamine (GlcNAc) transferase
1.48	YARS	tyrosyl-tRNA synthetase
1.48	GNL2	guanine nucleotide binding protein-like 2 (nucleolar)
1.48	AGFG1; MIR5703	ArfGAP with FG repeats 1; microRNA 5703
1.48	FAM60A	family with sequence similarity 60. member A
1.48	CEPT1	choline/ethanolamine phosphotransferase 1
1.48	MYO1E	myosin IE
1.48	PRAMEF2	PRAME family member 2
1.48	CEP85L	centrosomal protein 85kDa-like
1.48	ANKRD40	ankyrin repeat domain 40
1.48	DDX21	DEAD (Asp-Glu-Ala-Asp) box helicase 21
1.48	CD68	CD68 molecule
1.48	CCNJ	cyclin J

1.48	RPL21	Homo sapiens ribosomal protein L21. mRNA (cDNA clone MGC:2150 IMAGE:3543702). complete cds.; Homo sapiens ribosomal protein L21. mRNA (cDNA clone MGC:4136 IMAGE:2964441). complete cds.; Homo sapiens ribosomal protein L21. mRNA (cDNA clone MGC:71252 IMAGE:6084268). complete cds.; Homo sapiens ribosomal protein L21. mRNA (cDNA clone MGC:88165 IMAGE:4281405). complete cds.; Homo sapiens ribosomal protein L21. mRNA (cDNA clone MGC:88326 IMAGE:4248655). complete cds.; Homo sapiens ribosomal protein L21. mRNA (cDNA clone MGC:88334 IMAGE:3931616). complete cds.; Homo sapiens ribosomal protein L21. mRNA (cDNA clone MGC:88581 IMAGE:4901492). complete cds.; Homo sapiens ribosomal protein L21. mRNA (cDNA clone MGC:104275 IMAGE:6724053). complete cds.
1.48	ZNF146	zinc finger protein 146
1.48	FLNA	Memczak2013 ANTISENSE. CDS. coding. INTERNAL best transcript NM_001456
1.47	ABHD13	abhydrolase domain containing 13
1.47	CCT7	chaperonin containing TCP1. subunit 7 (eta)
1.47	DDIT3	DNA-damage-inducible transcript 3
1.47	CCDC68	coiled-coil domain containing 68
1.47	FRMD4A	FERM domain containing 4A
1.47	HK1	hexokinase 1
1.47	ERCC4	excision repair cross-complementation group 4
1.47	EFTUD1	elongation factor Tu GTP binding domain containing 1
1.47	PIM3	Pim-3 proto-oncogene. serine/threonine kinase
1.47	PDCD10	programmed cell death 10
1.47	RNF24	ring finger protein 24
1.47	PRKCZ	protein kinase C. zeta
1.47	SIPA1	signal-induced proliferation-associated 1
1.47	ABCC3	ATP binding cassette subfamily C member 3
1.47	SOCS6	suppressor of cytokine signaling 6

1.47	BCAP29	B-cell receptor-associated protein 29
1.47	CCDC126	coiled-coil domain containing 126
1.47	MFSD12	major facilitator superfamily domain containing 12
1.47	TPD52L2	tumor protein D52-like 2
1.47	TTC37	tetratricopeptide repeat domain 37
1.47	EIF3D	eukaryotic translation initiation factor 3. subunit D
1.47	C10orf2	chromosome 10 open reading frame 2
1.47	SH2B3	SH2B adaptor protein 3
1.47	OXCT1	3-oxoacid CoA-transferase 1
1.47	CRLF3	cytokine receptor-like factor 3
1.47	BCL10	B-cell CLL/lymphoma 10
1.47	ZSCAN9	zinc finger and SCAN domain containing 9
1.47	CYGB; RP11-666A8.8	Transcript Identified by AceView. Entrez Gene ID(s) 114757; novel transcript. antisense to PRCD
1.47	CNTRL	centriolin
1.47	CXCL16	chemokine (C-X-C motif) ligand 16
1.47	GEMIN5	gem nuclear organelle associated protein 5
1.46	PACSIN2	protein kinase C and casein kinase substrate in neurons 2
1.46	DUSP4	dual specificity phosphatase 4
1.46	LMBRD2	LMBR1 domain containing 2
1.46	ATP2B4	ATPase. Ca++ transporting. plasma membrane 4
1.46	IFNGR2	interferon gamma receptor 2 (interferon gamma transducer 1)
1.46	KBTBD8	kelch repeat and BTB (POZ) domain containing 8
1.46	TXNRD2	thioredoxin reductase 2
1.46	KRAS	Kirsten rat sarcoma viral oncogene homolog
1.46	DPH6	diphthamine biosynthesis 6

1.46	PRKAA1	protein kinase. AMP-activated. alpha 1 catalytic subunit
1.46	STRN3	striatin. calmodulin binding protein 3
1.46	GEMIN6	gem nuclear organelle associated protein 6
1.46	HUS1	HUS1 checkpoint clamp component
1.46	WDR35	WD repeat domain 35
1.46	RAB27A	RAB27A. member RAS oncogene family
1.46	EIF2A	eukaryotic translation initiation factor 2A. 65kDa
1.46	PLGLB1	Transcript Identified by AceView. Entrez Gene ID(s) 285074; 5343
1.46	TAMM41	TAM41 mitochondrial translocator assembly and maintenance homolog
1.46	ZCWPW2	zinc finger. CW type with PWWP domain 2
1.46	FAM89A; MIR1182	family with sequence similarity 89. member A; microRNA 1182
1.46	ENO2	enolase 2 (gamma. neuronal)
1.46	ATP10A	ATPase. class V. type 10A
1.46	BCL2L14	BCL2-like 14 (apoptosis facilitator)
1.46	TRPM7	transient receptor potential cation channel. subfamily M. member 7
1.46	CLDN4	claudin 4
1.46	ARHGEF28	Rho guanine nucleotide exchange factor 28
1.46	ERCC1	excision repair cross-complementation group 1
1.45	ZZZ3	Transcript Identified by AceView. Entrez Gene ID(s) 26009
1.45	GOLM1	golgi membrane protein 1
1.45	ABCE1	ATP binding cassette subfamily E member 1
1.45	NMD3	NMD3 ribosome export adaptor
1.45	ABCC8	ATP binding cassette subfamily C member 8
1.45	SLK	STE20-like kinase
1.45	CLDN34	claudin 34
1.45	OR51E1	olfactory receptor. family 51. subfamily E. member 1

1.45	CALM2	calmodulin 2 (phosphorylase kinase. delta)
1.45	HIPK3	homeodomain interacting protein kinase 3
1.45	MET	MET proto-oncogene. receptor tyrosine kinase
1.45	FBXO45	F-box protein 45
1.45	CDHR1	cadherin-related family member 1
1.45	TMEM69	transmembrane protein 69
1.45	ITGA3	integrin alpha 3
1.45	PES1	pescadillo ribosomal biogenesis factor 1
1.45	ZNRD1	zinc ribbon domain containing 1
1.45	PREX2	phosphatidylinositol-3.4.5-trisphosphate-dependent Rac exchange factor 2
1.45	SLCO4A1	solute carrier organic anion transporter family. member 4A1
1.45	UBE2Z	ubiquitin-conjugating enzyme E2Z
1.45	OR3A1	olfactory receptor. family 3. subfamily A. member 1
1.45	EOGT	EGF domain-specific O-linked N-acetylglucosamine (GlcNAc) transferase
1.45	LRCH1	leucine-rich repeats and calponin homology (CH) domain containing 1
1.45	HOMER2	homer scaffolding protein 2
1.45	SLC51A	solute carrier family 51. alpha subunit
1.45	POLR3C	polymerase (RNA) III (DNA directed) polypeptide C (62kD)
1.45	ZDHHC2	zinc finger. DHHC-type containing 2
1.45	LZTR1	leucine-zipper-like transcription regulator 1
1.45	CNIH4	cornichon family AMPA receptor auxiliary protein 4
1.45	ESF1	ESF1 nucleolar pre-rRNA processing protein homolog
1.45	SLC9A2	solute carrier family 9. subfamily A (NHE2. cation proton antiporter 2). member 2
1.45	P2RX1	purinergic receptor P2X. ligand gated ion channel. 1
1.45	PIGC	phosphatidylinositol glycan anchor biosynthesis class C

1.45	STX1B	syntaxin 1B
1.45	BFAR	bifunctional apoptosis regulator
1.45	KCTD13	potassium channel tetramerization domain containing 13
1.44	DBN1	drebrin 1
1.44	CMC4; MTCP1	C-x(9)-C motif containing 4; mature T-cell proliferation 1
1.44	CAST	calpastatin
1.44	GPHN	gephyrin
1.44	PXN	paxillin
1.44	TAF9; AK6	TAF9 RNA polymerase II. TATA box binding protein (TBP)-associated factor. 32kDa; adenylate kinase 6
1.44	EIF2B5	eukaryotic translation initiation factor 2B. subunit 5 epsilon. 82kDa
1.44	DAGLA	diacylglycerol lipase. alpha
1.44	RPL29	ribosomal protein L29
1.44	JADE3	jade family PHD finger 3
1.44	MGC27345	uncharacterized protein MGC27345
1.44	FAM47A	family with sequence similarity 47. member A
1.44	SERTAD2	SERTA domain containing 2
1.44	CCT4	chaperonin containing TCP1. subunit 4 (delta)
1.44	CCDC122	coiled-coil domain containing 122
1.44	PDCD2L	programmed cell death 2-like
1.44	RCN1	reticulocalbin 1. EF-hand calcium binding domain
1.44	CASQ2	calsequestrin 2 (cardiac muscle)
1.44	BEST1	bestrophin 1
1.44	VDAC2	voltage-dependent anion channel 2
1.44	OSGEP	O-sialoglycoprotein endopeptidase
1.44	FAM45A	Transcript Identified by AceView. Entrez Gene ID(s) 404636

1.44	FOSL2	FOS-like antigen 2
1.44	ERAL1	Era-like 12S mitochondrial rRNA chaperone 1
1.44	SLC29A3	solute carrier family 29 (equilibrative nucleoside transporter). member 3
1.44	ARHGAP5	Rho GTPase activating protein 5
1.44	ZFP36L1	ZFP36 ring finger protein-like 1
1.44	ABHD5	abhydrolase domain containing 5
1.44	ACBD5	acyl-CoA binding domain containing 5
1.44	DPPA3	developmental pluripotency associated 3
1.44	MARCH5	membrane associated ring finger 5
1.44	ZNF546	zinc finger protein 546
1.44	LNX2	ligand of numb-protein X 2
1.44	TRIP10	thyroid hormone receptor interactor 10
1.44	KPNA4	karyopherin alpha 4 (importin alpha 3)
1.43	SMAD1	SMAD family member 1
1.43	TRIP11	thyroid hormone receptor interactor 11
1.43	FBXW7	F-box and WD repeat domain containing 7. E3 ubiquitin protein ligase
1.43	EI24	etoposide induced 2.4
1.43	ZNF317	zinc finger protein 317
1.43	CHST10	carbohydrate sulfotransferase 10
1.43	KLF6	Kruppel-like factor 6
1.43	C3orf62	chromosome 3 open reading frame 62
1.43	CTNNAL1	catenin (cadherin-associated protein). alpha-like 1
1.43	OR4N5	olfactory receptor. family 4. subfamily N. member 5
1.43	RPL22L1	ribosomal protein L22-like 1
1.43	PTBP2	polypyrimidine tract binding protein 2
1.43	ZC2HC1A	zinc finger. C2HC-type containing 1A

1.43	KIF5A	kinesin family member 5A
1.43	SFRP2	secreted frizzled-related protein 2
1.43	SGPP1	sphingosine-1-phosphate phosphatase 1
1.43	PLEKHA1	pleckstrin homology domain containing. family A (phosphoinositide binding specific) member 1
1.43	GPR35	G protein-coupled receptor 35
1.43	LATS1	large tumor suppressor kinase 1
1.43	GEMIN7	gem nuclear organelle associated protein 7
1.43	MB21D2	Mab-21 domain containing 2
1.43	SGK223	homolog of rat pragma of Rnd2; Tyrosine-protein kinase SgK223 [Source:UniProtKB/Swiss-Prot;Acc:Q86YV5]
1.43	OSBPL3	oxysterol binding protein-like 3
1.43	DUSP16	dual specificity phosphatase 16
1.43	CEP57	centrosomal protein 57kDa
1.43	GOLGA7	golgin A7
1.43	PEX11B	peroxisomal biogenesis factor 11 beta
1.43	POGK	pogo transposable element with KRAB domain
1.43	ANKRD44	ankyrin repeat domain 44
1.43	PCCA	propionyl-CoA carboxylase alpha subunit
1.43	LOC100129216	beta-defensin 131-like
1.43	BCAT1	branched chain amino-acid transaminase 1. cytosolic
1.43	KIAA1033	KIAA1033
1.43	TRIB3	tribbles pseudokinase 3
1.43	WDR66	WD repeat domain 66
1.43	MLPH	melanophilin
1.43	PKIB	protein kinase (cAMP-dependent. catalytic) inhibitor beta
1.43	SAMD1	sterile alpha motif domain containing 1

1.43	C5AR2	complement component 5a receptor 2
1.43	GCNT4	glucosaminyl (N-acetyl) transferase 4. core 2
1.42	TOP2B; MIR4442	topoisomerase (DNA) II beta; microRNA 4442
1.42	ELOVL4	ELOVL fatty acid elongase 4
1.42	ATP13A3	ATPase type 13A3
1.42	UCK2; MIR3658	uridine-cytidine kinase 2; microRNA 3658
1.42	FBXO42	F-box protein 42
1.42	KLHDC7B	kelch domain containing 7B
1.42	TIGAR	TP53 induced glycolysis regulatory phosphatase
1.42	BLMH	bleomycin hydrolase
1.42	GPAT4	glycerol-3-phosphate acyltransferase 4
1.42	AARS2	alanyl-tRNA synthetase 2. mitochondrial
1.42	AP1S2	adaptor-related protein complex 1 sigma 2 subunit
1.42	LTBR	lymphotoxin beta receptor (TNFR superfamily. member 3)
1.42	C10orf88	chromosome 10 open reading frame 88
1.42	PAK1IP1	PAK1 interacting protein 1
1.42	DIMT1	DIM1 dimethyladenosine transferase 1 homolog
1.42	ACBD6	acyl-CoA binding domain containing 6
1.42	SAGE1	sarcoma antigen 1
1.42	RPL36A	ribosomal protein L36a
1.42	PDSS1	prenyl (decaprenyl) diphosphate synthase. subunit 1
1.42	MDM4	MDM4. p53 regulator
1.42	NOL8	nucleolar protein 8
1.42	DLGAP1	Transcript Identified by AceView. Entrez Gene ID(s) 9229
1.42	TNFAIP8	tumor necrosis factor. alpha-induced protein 8
1.42	DIAPH2	diaphanous-related formin 2

1.42	GMFB	glia maturation factor. beta
1.42	CDC42BPA	CDC42 binding protein kinase alpha (DMPK-like)
1.42	CEP120	centrosomal protein 120kDa
1.42	TAS2R5	taste receptor. type 2. member 5
1.42	MRPS5	mitochondrial ribosomal protein S5
1.42	ACTA2	actin. alpha 2. smooth muscle. aorta
1.42	DDX1	DEAD (Asp-Glu-Ala-Asp) box helicase 1
1.42	METAP2	methionyl aminopeptidase 2
1.42	LIN28B	lin-28 homolog B (C. elegans)
1.42	RPAP3	RNA polymerase II associated protein 3
1.42	DEGS1	delta(4)-desaturase. sphingolipid 1
1.42	BCL2L1	BCL2-like 1
1.42	ZNF562	zinc finger protein 562
1.42	PRKAR1A	Memczak2013 ALT_ACCEPTOR. ALT_DONOR. coding. INTERNAL. intronic best transcript NM_212471
1.42	IL31RA	interleukin 31 receptor A
1.42	MBTPS2; YY2	membrane bound transcription factor peptidase. site 2; YY2 transcription factor
1.42	RRN3	RRN3 homolog. RNA polymerase I transcription factor
1.42	ATXN7	ataxin 7
1.42	UBE2E1	ubiquitin conjugating enzyme E2E 1
1.41	SCCPDH	saccharopine dehydrogenase (putative)
1.41	SLC44A5	solute carrier family 44. member 5
1.41	MLXIP	MLX interacting protein
1.41	TMEM44	transmembrane protein 44
1.41	TMEM161B	transmembrane protein 161B
1.41	CAMLG	calcium modulating ligand

1.41	POGZ	Transcript Identified by AceView. Entrez Gene ID(s) 23126
1.41	EML6	echinoderm microtubule associated protein like 6
1.41	OLFML2B	olfactomedin like 2B
1.41	LRRC8E	leucine rich repeat containing 8 family. member E
1.41	EFTUD2	elongation factor Tu GTP binding domain containing 2
1.41	YBEY	ybeY metallopeptidase (putative)
1.41	USP25	ubiquitin specific peptidase 25
1.41	B3GNT3	UDP-GlcNAc:betaGal beta-1.3-N-acetylglucosaminyltransferase 3
1.41	SOCS4	suppressor of cytokine signaling 4
1.41	SARS	seryl-tRNA synthetase
1.41	PERP	PERP. TP53 apoptosis effector
1.41	FRMPD2B; FRMPD2	FERM and PDZ domain containing 2B. pseudogene; FERM and PDZ domain containing 2
1.41	ABCB9	ATP binding cassette subfamily B member 9
1.41	ESD	Transcript Identified by AceView. Entrez Gene ID(s) 2098
1.41	TAX1BP1	Tax1 (human T-cell leukemia virus type I) binding protein 1
1.41	KLC2	kinesin light chain 2
1.41	CYP4A22	cytochrome P450. family 4. subfamily A. polypeptide 22
1.41	FOXP1	forkhead box P1
1.41	GP1BB; SEPT5	glycoprotein Ib (platelet). beta polypeptide; septin 5
1.41	PCNXL4	pecanex-like 4 (Drosophila)
1.41	TSEN2	TSEN2 tRNA splicing endonuclease subunit
1.41	NUP58	nucleoporin 58kDa
1.41	SEC14L1; SCARNA16; SNHG20; MIR6516	SEC14-like lipid binding 1; small Cajal body-specific RNA 16; small nucleolar RNA host gene 20; microRNA 6516
1.41	SLC1A5	solute carrier family 1 (neutral amino acid transporter). member 5
1.41	MRPL46	mitochondrial ribosomal protein L46

1.41	FAM198A	family with sequence similarity 198. member A
1.41	FAM174A	family with sequence similarity 174. member A
1.41	SORL1	sortilin-related receptor. L(DLR class) A repeats containing
1.41	CPED1	cadherin-like and PC-esterase domain containing 1
1.41	ARSG	arylsulfatase G
1.41	FUNDC2	FUN14 domain containing 2
1.41	AKAP6	A kinase (PRKA) anchor protein 6
1.41	GABRR1	gamma-aminobutyric acid (GABA) A receptor. rho 1
1.41	MREG	melanoregulin
1.41	BAIAP2L2	BAI1-associated protein 2-like 2
1.41	CREBRF	CREB3 regulatory factor
1.41	VEZF1	vascular endothelial zinc finger 1
1.41	ST7L	suppression of tumorigenicity 7 like
1.40	MRPL9	mitochondrial ribosomal protein L9
1.40	MRPL15	mitochondrial ribosomal protein L15
1.40	NOP16	NOP16 nucleolar protein
1.40	HS3ST5	heparan sulfate (glucosamine) 3-O-sulfotransferase 5
1.40	LACC1	laccase (multicopper oxidoreductase) domain containing 1
1.40	PRKD3	protein kinase D3
1.40	PFN2	profilin 2
1.40	ARHGAP42	Rho GTPase activating protein 42
1.40	KCNQ3	potassium channel. voltage gated KQT-like subfamily Q. member 3
1.40	PIAS2	protein inhibitor of activated STAT 2
1.40	NDUFA12	NADH dehydrogenase (ubiquinone) 1 alpha subcomplex. 12
1.40	ARMCX5	armadillo repeat containing. X-linked 5
1.40	NR2C2	nuclear receptor subfamily 2. group C. member 2

1.40	TSC22D2	TSC22 domain family. member 2
1.40	MICAL1	microtubule associated monooxygenase. calponin and LIM domain containing 1
1.40	C5	complement component 5
1.40	CEBPG	CCAAT/enhancer binding protein (C/EBP). gamma
1.40	VGF	VGF nerve growth factor inducible
1.40	PTRHD1	peptidyl-tRNA hydrolase domain containing 1
1.40	ZNF711	zinc finger protein 711
1.40	EHD2	EH domain containing 2
1.40	CLPTM1L	CLPTM1-like
1.40	SSRP1	structure specific recognition protein 1
1.40	REXO4	REX4 homolog. 3'-5' exonuclease [Source:HGNC Symbol;Acc:HGNC:12820]
1.40	NCR3LG1	natural killer cell cytotoxicity receptor 3 ligand 1
1.40	SRSF12	serine/arginine-rich splicing factor 12
1.40	ESD	esterase D
1.40	ALAS1	5-aminolevulinate synthase 1
1.40	RFK	riboflavin kinase
1.40	CSNK1A1	casein kinase 1. alpha 1
1.40	PRORY	proline rich. Y-linked
1.40	TRIM44	tripartite motif containing 44
1.40	SPEM1	spermatid maturation 1
1.40	HYPM	huntingtin interacting protein M
1.40	FAM47B	family with sequence similarity 47. member B
1.40	ME2	malic enzyme 2. NAD(+)-dependent. mitochondrial
1.40	EGLN1	egl-9 family hypoxia-inducible factor 1
1.40	PABPC4	poly(A) binding protein. cytoplasmic 4 (inducible form)

1.40	KLF8	Kruppel-like factor 8
1.40	CPT1A	carnitine palmitoyltransferase 1A (liver)
1.40	TMC8	transmembrane channel like 8
1.40	CCDC112	coiled-coil domain containing 112
1.40	XAGE2	X antigen family. member 2
1.40	BID	BH3 interacting domain death agonist
1.40	FBXO8	F-box protein 8
1.40	KLHL42	kelch-like family member 42
1.40	RPL21	ribosomal protein L21
1.40	MT1E	metallothionein 1E
1.40	POU4F3	POU class 4 homeobox 3
1.40	PTER	phosphotriesterase related
1.40	CTNNA2	catenin (cadherin-associated protein). alpha 2
1.40	KIAA0040	KIAA0040
1.39	GRIN1	glutamate receptor. ionotropic. N-methyl D-aspartate 1
1.39	PRMT5	protein arginine methyltransferase 5
1.39	ACAN	aggrecan
1.39	AAED1	AhpC/TSA antioxidant enzyme domain containing 1
1.39	ZNF74	zinc finger protein 74
1.39	SLC38A9	solute carrier family 38. member 9
1.39	HSPA9	heat shock 70kDa protein 9 (mortalin)
1.39	NLE1	notchless homolog 1 (Drosophila)
1.39	TEP1	telomerase-associated protein 1
1.39	FILIP1L	filamin A interacting protein 1-like
1.39	RPL36A-HNRNPH2	RPL36A-HNRNPH2 readthrough
1.39	NUDT9	nudix hydrolase 9

1.39	IGBP1	immunoglobulin (CD79A) binding protein 1
1.39	EPRS	glutamyl-prolyl-tRNA synthetase
1.39	STK3	serine/threonine kinase 3
1.39	GOT1	glutamic-oxaloacetic transaminase 1. soluble
1.39	MTRR	5-methyltetrahydrofolate-homocysteine methyltransferase reductase
1.39	STARD7-AS1	STARD7 antisense RNA 1
1.39	NSUN2	NOP2/Sun RNA methyltransferase family. member 2
1.39	TNS4	tensin 4
1.39	SEMA3A	sema domain. immunoglobulin domain (Ig). short basic domain. secreted. (semaphorin) 3A
1.39	LOC285692; CTD-2143L24.1; TAS2R1	uncharacterized LOC285692; novel transcript; Transcript Identified by AceView. Entrez Gene ID(s) 285692; 50834
1.39	GRPEL2	GrpE-like 2. mitochondrial (E. coli)
1.39	C8orf4	chromosome 8 open reading frame 4
1.39	STX2	syntaxin 2
1.39	HPGDS	hematopoietic prostaglandin D synthase
1.39	MIDN	midnolin
1.39	GOLGA8M	golgin A8 family. member M
1.39	LRP8	LDL receptor related protein 8
1.39	HECW2	HECT. C2 and WW domain containing E3 ubiquitin protein ligase 2
1.39	MLXIP	MLX interacting protein
1.39	KRTAP19-5	keratin associated protein 19-5
1.39	NFAT5	nuclear factor of activated T-cells 5. tonicity-responsive
1.39	MRPS33	mitochondrial ribosomal protein S33
1.39	TBC1D19	TBC1 domain family. member 19
1.39	COL4A3BP	collagen. type IV. alpha 3 (Goodpasture antigen) binding protein
1.39	ATP12A	ATPase. H+/K+ transporting. nongastric. alpha polypeptide

1.39	LOC100128554; RP5-944M2.3	uncharacterized LOC100128554; Transcript Identified by AceView. Entrez Gene ID(s) 144678; 100128554; novel transcript
1.39	DNMBP	dynamin binding protein
1.39	SLC25A25	Zhang2013 ALT_ACCEPTOR. ALT_DONOR. coding. INTERNAL. intronic best transcript NM_001006641
1.38	ITGA6	integrin alpha 6
1.38	ZNF609	zinc finger protein 609
1.38	IL1R1	interleukin 1 receptor. type I
1.38	NHLRC2	NHL repeat containing 2
1.38	PHF20	PHD finger protein 20
1.38	SLC7A5	solute carrier family 7 (amino acid transporter light chain. L system). member 5
1.38	GNAQ	guanine nucleotide binding protein (G protein). q polypeptide
1.38	INPP5F	inositol polyphosphate-5-phosphatase F
1.38	ZNF580; ZNF581	zinc finger protein 580; zinc finger protein 581
1.38	UAP1L1	UDP-N-acetylglucosamine pyrophosphorylase 1 like 1
1.38	CLASP2	cytoplasmic linker associated protein 2
1.38	HINFP	histone H4 transcription factor
1.38	MT1M	metallothionein 1M
1.38	RSL24D1	ribosomal L24 domain containing 1
1.38	TCEA1	transcription elongation factor A (SII). 1
1.38	DEFA3	defensin. alpha 3. neutrophil-specific
1.38	MFSD1	major facilitator superfamily domain containing 1
1.38	WDR12	WD repeat domain 12
1.38	ZNF142	zinc finger protein 142
1.38	BLACAT1; LEMD1	bladder cancer associated transcript 1 (non-protein coding); LEM domain containing 1

1.38	IL18	interleukin 18
1.38	TTLL12	tubulin tyrosine ligase-like family member 12
1.38	ITSN2	intersectin 2
1.38	GLRX3	glutaredoxin 3
1.38	NCF2	neutrophil cytosolic factor 2
1.38	PRR5L	proline rich 5 like
1.38	SURF2	surfeit 2 [Source:HGNC Symbol;Acc:HGNC:11475]
1.38	GRB14	growth factor receptor bound protein 14
1.38	UTP15	UTP15. U3 small nucleolar ribonucleoprotein. homolog (S. cerevisiae)
1.38	SULF2	sulfatase 2
1.38	UTP18	UTP18 small subunit (SSU) processome component
1.38	FGL1	fibrinogen like 1
1.38	RPF2	ribosome production factor 2 homolog
1.38	RCOR1	REST corepressor 1
1.38	DEFB107B; DEFB107A	defensin. beta 107B; defensin. beta 107A
1.38	HCAR2	hydroxycarboxylic acid receptor 2
1.38	YARS2	tyrosyl-tRNA synthetase 2. mitochondrial
1.38	WNT2B	wingless-type MMTV integration site family. member 2B
1.38	TROVE2	TROVE domain family. member 2
1.38	REXO4	REX4 homolog. 3'-5' exonuclease [Source:HGNC Symbol;Acc:HGNC:12820]
1.38	CT47B1	cancer/testis antigen family 47. member B1
1.38	GK	glycerol kinase
1.38	THOC2	THO complex 2
1.38	RIPK2	receptor-interacting serine-threonine kinase 2
1.38	GOLGA7B	golgin A7 family. member B
1.38	SIRT1	sirtuin 1

1.38	NEDD9	Memczak2013 ALT_ACCEPTOR. ALT_DONOR. coding. INTERNAL. intronic best transcript NM_001142393
1.38	HMGCL	3-hydroxymethyl-3-methylglutaryl-CoA lyase
1.38	KLRB1	killer cell lectin-like receptor subfamily B. member 1
1.38	SLC38A5	solute carrier family 38. member 5
1.38	SESN2	sestrin 2
1.38	C9orf91	chromosome 9 open reading frame 91
1.38	KLF13	Memczak2013 ANTISENSE. coding. INTERNAL. intronic best transcript NM_015995
1.37	CPEB3	cytoplasmic polyadenylation element binding protein 3
1.37	KHDRBS3	KH domain containing. RNA binding. signal transduction associated 3
1.37	TOX	thymocyte selection-associated high mobility group box
1.37	ATP11C	ATPase. class VI. type 11C
1.37	SEC22A	SEC22 homolog A. vesicle trafficking protein
1.37	AKAP10	A kinase (PRKA) anchor protein 10
1.37	ARNTL2	aryl hydrocarbon receptor nuclear translocator-like 2
1.37	PIK3CA	phosphatidylinositol-4.5-bisphosphate 3-kinase. catalytic subunit alpha
1.37	PRTG	protogenin
1.37	SNORD49A; SNORD65; LRRC75A-AS1	small nucleolar RNA. C/D box 49A; small nucleolar RNA. C/D box 65; LRRC75A antisense RNA 1
1.37	ZNF426	zinc finger protein 426
1.37	ADAM9	ADAM metallopeptidase domain 9
1.37	FCGR2A	Fc fragment of IgG. low affinity IIa. receptor (CD32)
1.37	RWDD1	RWD domain containing 1
1.37	MOGAT3	monoacylglycerol O-acyltransferase 3
1.37	RBM11	RNA binding motif protein 11
1.37	CDK17	cyclin-dependent kinase 17

1.37	CAMSAP1	calmodulin regulated spectrin-associated protein 1
1.37	KIAA1671	KIAA1671
1.37	FRA10AC1	fragile site. folic acid type. rare. fra(10)(q23.3) or fra(10)(q24.2) candidate 1
1.37	PIPOX	pipecolic acid oxidase
1.37	MAP7D2	MAP7 domain containing 2
1.37	PDE4D	phosphodiesterase 4D. cAMP-specific
1.37	FBXO30	F-box protein 30
1.37	TSNAX	Memczak2013 ANTISENSE. CDS. coding. INTERNAL. UTR3 best transcript NM_005999
1.37	PDCD11	programmed cell death 11
1.37	DUSP16	dual specificity phosphatase 16
1.37	NXPH4	neurexophilin 4
1.37	DNAJB2	DnaJ (Hsp40) homolog. subfamily B. member 2
1.37	LCE1F	late cornified envelope 1F
1.37	ICE1	interactor of little elongation complex ELL subunit 1
1.37	LPIN2	lipin 2
1.37	ADAMTSL4-AS1	ADAMTSL4 antisense RNA 1
1.37	MPP6	membrane protein. palmitoylated 6
1.37	C12orf29	chromosome 12 open reading frame 29
1.37	NOS3	nitric oxide synthase 3 (endothelial cell)
1.37	JOSD1	Josephin domain containing 1
1.37	SDC4	syndecan 4
1.37	PEX13	peroxisomal biogenesis factor 13
1.37	ATP11B	ATPase. class VI. type 11B
1.37	PPT1	palmitoyl-protein thioesterase 1
1.37	CDCP1	CUB domain containing protein 1

1.37	SERPINB7	serpin peptidase inhibitor. clade B (ovalbumin). member 7
1.37	ARHGAP12	Rho GTPase activating protein 12
1.37	KNDC1	Transcript Identified by AceView. Entrez Gene ID(s) 85442
1.37	PRAMEF8	PRAME family member 8
1.37	TTC5	tetratricopeptide repeat domain 5
1.37	XPOT	exportin. tRNA
1.37	TEX35	testis expressed 35
1.36	CCDC50	coiled-coil domain containing 50
1.36	STKLD1	serine/threonine kinase-like domain containing 1 [Source:HGNC Symbol;Acc:HGNC:28669]
1.36	ORM2	orosomucoid 2
1.36	SLC18B1	solute carrier family 18. subfamily B. member 1
1.36	HARBI1	harbinger transposase derived 1
1.36	FLRT3	fibronectin leucine rich transmembrane protein 3
1.36	GARS	glycyl-tRNA synthetase
1.36	ACER2	alkaline ceramidase 2
1.36	LY6H	lymphocyte antigen 6 complex. locus H
1.36	OSMR	oncostatin M receptor
1.36	CEP97	centrosomal protein 97kDa
1.36	PPARG	peroxisome proliferator-activated receptor gamma
1.36	FAM135A	family with sequence similarity 135. member A
1.36	ATR	ATR serine/threonine kinase
1.36	KLC1	kinesin light chain 1
1.36	ITGB1	integrin beta 1
1.36	TM4SF5	transmembrane 4 L six family member 5
1.36	DKC1; SNORA36A; SNORA56; MIR664B	dyskeratosis congenita 1. dyskerin; small nucleolar RNA. H/ACA box 36A; small nucleolar RNA. H/ACA box 56; microRNA 664b

1.36	RPS13; SNORD14B	ribosomal protein S13; small nucleolar RNA. C/D box 14B
1.36	RGS12	regulator of G-protein signaling 12
1.36	SETBP1	Memczak2013 ALT_ACCEPTOR. ALT_DONOR. coding. INTERNAL. intronic best transcript NM_001130110
1.36	HNRNPCL4	heterogeneous nuclear ribonucleoprotein C-like 4 [Source:HGNC Symbol;Acc:HGNC:51333]
1.36	RPAIN	RPA interacting protein
1.36	CDKN2AIPNL	CDKN2A interacting protein N-terminal like
1.36	ZNF322	zinc finger protein 322
1.36	TSC1	tuberous sclerosis 1
1.36	ZYX	zyxin
1.36	DCBLD1	discoidin. CUB and LCCL domain containing 1
1.36	MRPS2	mitochondrial ribosomal protein S2
1.36	B4GALT6	UDP-Gal:betaGlcNAc beta 1.4- galactosyltransferase. polypeptide 6
1.36	TEX28	testis expressed 28
1.36	USP6NL	USP6 N-terminal like
1.36	CYLC2	cylicin. basic protein of sperm head cytoskeleton 2
1.36	ZNF501	zinc finger protein 501 [Source:HGNC Symbol;Acc:HGNC:23717]
1.36	CAPNS2	calpain. small subunit 2
1.36	RHOBTB3	Rho-related BTB domain containing 3
1.36	APIP	Transcript Identified by AceView. Entrez Gene ID(s) 51074
1.36	KRT18	keratin 18. type I
1.36	SWAP70	SWAP switching B-cell complex 70kDa subunit
1.36	TRIQK	triple QxxK/R motif containing
1.36	IFITM3	interferon induced transmembrane protein 3
1.36	CNIH1	cornichon family AMPA receptor auxiliary protein 1
1.36	LPAR2	lysophosphatidic acid receptor 2

1.36	SMIM20	small integral membrane protein 20
1.36	BIRC7	baculoviral IAP repeat containing 7
1.36	BEX4	brain expressed X-linked 4
1.36	RALGAPB	Ral GTPase activating protein. beta subunit (non-catalytic)
1.36	CR2	complement component (3d/Epstein Barr virus) receptor 2
1.36	CHML	choroideremia-like (Rab escort protein 2)
1.36	ANGPTL5	angiopoietin like 5
1.36	SENP3	SUMO1/sentrin/SMT3 specific peptidase 3
1.36	ZBTB45	Memczak2013 ALT_ACCEPTOR. ALT_DONOR. coding. INTERNAL. intronic best transcript NM_032792
1.36	CMPK2	cytidine monophosphate (UMP-CMP) kinase 2. mitochondrial
1.36	TEX19	testis expressed 19
1.36	SLCO6A1	solute carrier organic anion transporter family. member 6A1
1.36	EIF4ENIF1	eukaryotic translation initiation factor 4E nuclear import factor 1
1.36	HS2ST1	heparan sulfate 2-O-sulfotransferase 1
1.35	TAZ	tafazzin
1.35	SNX5	Jeck2013 ALT_DONOR. coding. INTERNAL. intronic best transcript NM_152227
1.35	RAET1E	retinoic acid early transcript 1E
1.35	OR10S1	olfactory receptor. family 10. subfamily S. member 1
1.35	KAT2B	K(lysine) acetyltransferase 2B
1.35	PRPS1	phosphoribosyl pyrophosphate synthetase 1
1.35	FAM175B	family with sequence similarity 175. member B
1.35	SNAPC1	small nuclear RNA activating complex polypeptide 1
1.35	MICU1	mitochondrial calcium uptake 1
1.35	FZD6	frizzled class receptor 6
1.35	MYO6	myosin VI

1.35	BLVRA	biliverdin reductase A
1.35	WSB2	WD repeat and SOCS box containing 2
1.35	RPL9	ribosomal protein L9
1.35	PINX1; MIR1322	PIN2/TERF1 interacting. telomerase inhibitor 1; microRNA 1322
1.35	IMPA1	inositol(myo)-1(or 4)-monophosphatase 1
1.35	FOXC1	forkhead box C1
1.35	SAG	S-antigen; retina and pineal gland (arrestin)
1.35	VTI1B	vesicle transport through interaction with t-SNAREs 1B
1.35	VPS4A	vacuolar protein sorting 4 homolog A (S. cerevisiae)
1.35	CIZ1	CDKN1A interacting zinc finger protein 1
1.35	DEF8	differentially expressed in FDCP 8 homolog (mouse)
1.35	NR2C2AP	nuclear receptor 2C2-associated protein
1.35	SPAG9	sperm associated antigen 9
1.35	INPP5D	inositol polyphosphate-5-phosphatase D
1.35	GART	phosphoribosylglycinamide formyltransferase. phosphoribosylglycinamide synthetase. phosphoribosylaminoimidazole synthetase
1.35	AP4S1	adaptor-related protein complex 4. sigma 1 subunit
1.35	LRP6	LDL receptor related protein 6
1.35	FYTTD1	forty-two-three domain containing 1
1.35	SLC9A6	solute carrier family 9. subfamily A (NHE6. cation proton antiporter 6). member 6
1.35	LINC00668	long intergenic non-protein coding RNA 668
1.35	KIF3C	kinesin family member 3C
1.35	NCBP3	nuclear cap binding subunit 3
1.35	ANO10	anoctamin 10
1.35	ATG16L1	autophagy related 16-like 1
1.35	TEX261	testis expressed 261

1.35	PPP1R8	protein phosphatase 1. regulatory subunit 8
1.35	FAM129B	family with sequence similarity 129. member B
1.35	DDX5	Memczak2013 ANTISENSE. CDS. coding. INTERNAL best transcript NM_004396
1.35	TCN1	transcobalamin I (vitamin B12 binding protein. R binder family)
1.35	SCAF11	Memczak2013 ANTISENSE. CDS. coding. INTERNAL best transcript NM_004719
1.35	N4BP2L1	NEDD4 binding protein 2-like 1
1.35	PYGB	phosphorylase. glycogen; brain
1.35	MARVELD2	MARVEL domain containing 2
1.35	ZMYM2	zinc finger. MYM-type 2
1.35	RAB9A	RAB9A. member RAS oncogene family
1.35	ULBP2	UL16 binding protein 2
1.35	ST6GAL2	Transcript Identified by AceView. Entrez Gene ID(s) 84620
1.35	FKTN	fukutin
1.35	RPS4Y2	ribosomal protein S4. Y-linked 2
1.35	KIAA0430; MIR6506	KIAA0430; microRNA 6506
1.35	SOX2	SRY box 2
1.35	GTF2H4	general transcription factor IIH subunit 4
1.35	VGLL3	vestigial-like family member 3
1.35	PKD2	polycystic kidney disease 2 (autosomal dominant)
1.35	SORL1	Memczak2013 ANTISENSE. CDS. coding. INTERNAL best transcript NM_003105
1.35	SLC33A1	solute carrier family 33 (acetyl-CoA transporter). member 1
1.35	LCN9	lipocalin 9
1.35	PPP3CB	protein phosphatase 3. catalytic subunit. beta isozyme
1.35	EGR3	early growth response 3

1.35	RPL12	Zhang2013 ALT_ACCEPTOR. ALT_DONOR. coding. INTERNAL. intronic best transcript NM_000976
1.35	HDAC5	histone deacetylase 5
1.35	GRAPL	GRB2-related adaptor protein-like
1.35	WDR72	WD repeat domain 72
1.35	CABP1	calcium binding protein 1
1.35	AVEN	apoptosis. caspase activation inhibitor
1.35	UBIAD1	UbiA prenyltransferase domain containing 1
1.35	PGM2	phosphoglucomutase 2
1.35	STAT3	signal transducer and activator of transcription 3 (acute-phase response factor)
1.35	VKORC1L1	vitamin K epoxide reductase complex subunit 1 like 1
1.35	SFXN4	sideroflexin 4
1.35	COG2	component of oligomeric golgi complex 2
1.34	NME5	NME/NM23 family member 5
1.34	CYSTM1	cysteine-rich transmembrane module containing 1
1.34	WDR41	WD repeat domain 41
1.34	DRAM1	DNA-damage regulated autophagy modulator 1
1.34	METTL8	methyltransferase like 8
1.34	SNORD102; SNORA27; RPL21	small nucleolar RNA. C/D box 102; small nucleolar RNA. H/ACA box 27; ribosomal protein L21
1.34	OR52D1	olfactory receptor. family 52. subfamily D. member 1
1.34	CYP2B6	cytochrome P450. family 2. subfamily B. polypeptide 6
1.34	PPP6R1	Memczak2013 ANTISENSE. CDS. coding. INTERNAL best transcript NM_014931
1.34	FBXL20	F-box and leucine-rich repeat protein 20
1.34	ATXN1	ataxin 1

1.34	ACTR6	ARP6 actin-related protein 6 homolog (yeast)
1.34	PRCC	papillary renal cell carcinoma (translocation-associated)
1.34	IFNAR2	interferon (alpha. beta and omega) receptor 2
1.34	SLC16A1	solute carrier family 16 (monocarboxylate transporter). member 1
1.34	RCBTB1	regulator of chromosome condensation (RCC1) and BTB (POZ) domain containing protein 1
1.34	SNAPC3	small nuclear RNA activating complex polypeptide 3
1.34	MEF2A	myocyte enhancer factor 2A
1.34	TRIM36	tripartite motif containing 36
1.34	ASTL	astacin-like metallo-endopeptidase (M12 family)
1.34	HOXC8	homeobox C8
1.34	DOC2B	double C2-like domains. beta
1.34	PEX14	peroxisomal biogenesis factor 14
1.34	SPTA1	spectrin. alpha. erythrocytic 1
1.34	ZNF502	zinc finger protein 502
1.34	LGALS1	lectin. galactoside-binding. soluble. 1
1.34	PDPN	podoplanin
1.34	PDLIM3	PDZ and LIM domain 3
1.34	HIBADH	Salzman2013 ALT_ACCEPTOR. ALT_DONOR. coding. INTERNAL. intronic best transcript NM_152740
1.34	ZNF229	zinc finger protein 229
1.34	GPCPD1	Transcript Identified by AceView. Entrez Gene ID(s) 56261
1.34	SLC10A1	solute carrier family 10 (sodium/bile acid cotransporter). member 1
1.34	IQSEC2	IQ motif and Sec7 domain 2
1.34	CLIC1	chloride intracellular channel 1
1.34	GTF2B	general transcription factor IIB
1.34	HYOU1	hypoxia up-regulated 1

1.34	CSNK1E	casein kinase 1. epsilon
1.34	ACE	angiotensin I converting enzyme
1.34	LRRC71	leucine rich repeat containing 71
1.34	FAM3C2	FAM3C pseudogene
1.34	NOS3	Transcript Identified by AceView. Entrez Gene ID(s) 4846
1.34	ZNF639	zinc finger protein 639
1.34	CLK1	CDC like kinase 1
1.34	CEL	carboxyl ester lipase
1.34	CTC1	CTS telomere maintenance complex component 1
1.34	GPRC5B	G protein-coupled receptor. class C. group 5. member B
1.34	IFITM2	interferon induced transmembrane protein 2
1.34	GALNT7	polypeptide N-acetylgalactosaminyltransferase 7
1.34	RDH10	retinol dehydrogenase 10 (all-trans)
1.34	FAM212B	family with sequence similarity 212. member B
1.34	RBMS1; MIR4785	RNA binding motif. single stranded interacting protein 1; microRNA 4785
1.34	ADGRF4	adhesion G protein-coupled receptor F4
1.34	URGCP	Salzman2013 ANNOTATED. coding. OVEXON. UTR5 best transcript NM_001077664
1.33	TRIP4	thyroid hormone receptor interactor 4
1.33	COA1	cytochrome c oxidase assembly factor 1 homolog
1.33	LRIG2	leucine-rich repeats and immunoglobulin-like domains 2
1.33	SLC24A3	solute carrier family 24 (sodium/potassium/calcium exchanger). member 3
1.33	SLC25A51	solute carrier family 25. member 51
1.33	DUSP16	dual specificity phosphatase 16
1.33	UBALD2	UBA-like domain containing 2
1.33	ABCC2	ATP binding cassette subfamily C member 2

1.33	METAP1	methionyl aminopeptidase 1
1.33	VIPR2	vasoactive intestinal peptide receptor 2
1.33	TRAPPC8	trafficking protein particle complex 8
1.33	LAP3	leucine aminopeptidase 3
1.33	LZTS3; ProSAPiP1	leucine zipper. putative tumor suppressor family member 3; Leucine zipper putative tumor suppressor 3 [Source:UniProtKB/Swiss-Prot;Acc:O60299]; Salzman2013 ANNOTATED. CDS. coding. OVCODE. OVEXON. UTR5 best transcript NM_014731
1.33	WDR36	WD repeat domain 36
1.33	DPY30	dpy-30 histone methyltransferase complex regulatory subunit
1.33	RNF41	ring finger protein 41. E3 ubiquitin protein ligase
1.33	ZACN	zinc activated ligand-gated ion channel
1.33	GCC2	GRIP and coiled-coil domain containing 2
1.33	SRP68	signal recognition particle 68kDa
1.33	ALG9	ALG9. alpha-1.2-mannosyltransferase
1.33	MRPS23	mitochondrial ribosomal protein S23
1.33	HNRNPLL	heterogeneous nuclear ribonucleoprotein L-like
1.33	ZNF285	zinc finger protein 285
1.33	PMAIP1	phorbol-12-myristate-13-acetate-induced protein 1
1.33	UBA2	ubiquitin-like modifier activating enzyme 2
1.33	SVIL	supervillin
1.33	PLPP1; RNF138P1	phospholipid phosphatase 1; ring finger protein 138. E3 ubiquitin protein ligase pseudogene 1
1.33	ARL8B	ADP-ribosylation factor like GTPase 8B
1.33	ELAC2	elaC ribonuclease Z 2
1.33	KCNV2	potassium channel. voltage gated modifier subfamily V. member 2
1.33	ZNF404	zinc finger protein 404

1.33	PCNXL2	pecanex-like 2 (Drosophila)
1.33	ATAD2B	ATPase family. AAA domain containing 2B
1.33	HNRNPCL4	heterogeneous nuclear ribonucleoprotein C-like 4
1.33	INPP5D	inositol polyphosphate-5-phosphatase D
1.33	COX4I1	cytochrome c oxidase subunit IV isoform 1
1.33	DBNDD2	dysbindin (dystrobrevin binding protein 1) domain containing 2
1.33	TTC26	tetratricopeptide repeat domain 26
1.33	SLC16A1	solute carrier family 16 (monocarboxylate transporter). member 1
1.33	ATP1B3	ATPase. Na+/K+ transporting. beta 3 polypeptide
1.33	FAM13C	family with sequence similarity 13. member C
1.33	RNF139	ring finger protein 139
1.33	MUC5AC	mucin 5AC. oligomeric mucus/gel-forming
1.33	MKKS	McKusick-Kaufman syndrome
1.33	POLE4	polymerase (DNA-directed). epsilon 4. accessory subunit
1.33	LOC100421561	family with sequence similarity 133. member A pseudogene
1.33	IQGAP1	IQ motif containing GTPase activating protein 1
1.33	TGFB1I1	transforming growth factor beta 1 induced transcript 1
1.33	CMBL	carboxymethylenebutenolidase homolog (Pseudomonas)
1.33	ABCC4	ATP binding cassette subfamily C member 4
1.33	IL1R2	interleukin 1 receptor. type II
1.33	CSNK1D	casein kinase 1. delta
1.33	HACD1	3-hydroxyacyl-CoA dehydratase 1
1.33	TRIAP1	TP53 regulated inhibitor of apoptosis 1
1.33	ADCK1	aarF domain containing kinase 1
1.33	MPZL2	myelin protein zero-like 2
1.33	DDX60L	DEAD (Asp-Glu-Ala-Asp) box polypeptide 60-like

1.33	SIRT4	sirtuin 4
1.33	DNAJC16	DnaJ (Hsp40) homolog. subfamily C. member 16
1.33	GADL1	glutamate decarboxylase like 1
1.33	ASAP1; ASAP1-IT2	ArfGAP with SH3 domain. ankyrin repeat and PH domain 1; ASAP1 intronic transcript 2
1.33	TNXB	tenascin XB
1.33	ADAM17	ADAM metallopeptidase domain 17
1.33	PRR4	proline rich 4 (lacrimal)
1.33	PCMTD1	protein-L-isoaspartate (D-aspartate) O-methyltransferase domain containing 1
1.33	PIGU	phosphatidylinositol glycan anchor biosynthesis class U
1.33	CTHRC1	collagen triple helix repeat containing 1
1.33	PHKA1	phosphorylase kinase. alpha 1 (muscle)
1.33	APOBEC3C	apolipoprotein B mRNA editing enzyme. catalytic polypeptide-like 3C
1.33	CAMK2D	calcium/calmodulin-dependent protein kinase II delta
1.33	DUSP8	dual specificity phosphatase 8
1.32	ATAD1	ATPase family. AAA domain containing 1
1.32	GCNT3	glucosaminyl (N-acetyl) transferase 3. mucin type
1.32	COPS7B	COP9 signalosome subunit 7B
1.32	PUM3	pumilio RNA binding family member 3
1.32	DUSP16	dual specificity phosphatase 16
1.32	FOXN2	forkhead box N2
1.32	LST1	leukocyte specific transcript 1
1.32	C16orf45	chromosome 16 open reading frame 45
1.32	SLC38A1	solute carrier family 38. member 1
1.32	KCNG3	potassium channel. voltage gated modifier subfamily G. member 3
1.32	CLUAP1	clusterin associated protein 1

1.32	SMURF2	SMAD specific E3 ubiquitin protein ligase 2
1.32	SLC17A6	solute carrier family 17 (vesicular glutamate transporter). member 6
1.32	ATP6V1E2	ATPase. H+ transporting. lysosomal 31kDa. V1 subunit E2
1.32	CYP7B1	cytochrome P450. family 7. subfamily B. polypeptide 1
1.32	SPANXN4	SPANX family. member N4
1.32	ZBTB39	zinc finger and BTB domain containing 39
1.32	MRPS10	mitochondrial ribosomal protein S10
1.32	RHBDD1	rhomboid domain containing 1
1.32	OR13A1	olfactory receptor. family 13. subfamily A. member 1
1.32	MACROD1	MACRO domain containing 1
1.32	IQCF3	Transcript Identified by AceView. Entrez Gene ID(s) 401067
1.32	SLC35B1	solute carrier family 35. member B1
1.32	SRPX2	sushi-repeat containing protein. X-linked 2
1.32	FAM73A	family with sequence similarity 73. member A
1.32	PLEKHM3	pleckstrin homology domain containing. family M. member 3
1.32	FIGNL2	fidgetin-like 2
1.32	DHX36	DEAH (Asp-Glu-Ala-His) box polypeptide 36
1.32	TBKBP1	TBK1 binding protein 1
1.32	GPATCH11	G-patch domain containing 11
1.32	SLC8B1	solute carrier family 8 (sodium/lithium/calcium exchanger). member B1
1.32	KITLG	KIT ligand
1.32	KRTAP9-2	keratin associated protein 9-2
1.32	LRP6	LDL receptor related protein 6
1.32	SLC39A3	solute carrier family 39 (zinc transporter). member 3
1.32	ATP5S	ATP synthase. H+ transporting. mitochondrial Fo complex subunit s (factor B)
1.32	LINC00483	long intergenic non-protein coding RNA 483

1.32	COMMD1	copper metabolism (Murr1) domain containing 1
1.32	DCAF8L2	DDB1 and CUL4 associated factor 8-like 2
1.32	ARRDC2	arrestin domain containing 2
1.32	ANGPT1	angiopoietin 1
1.32	DENND4A	DENN/MADD domain containing 4A
1.32	REV1	REV1. DNA directed polymerase
1.32	BZW2	basic leucine zipper and W2 domains 2
1.32	RPL9P7	ribosomal protein L9 pseudogene 7
1.32	SMCO2	single-pass membrane protein with coiled-coil domains 2
1.32	ZPR1	ZPR1 zinc finger
1.32	ANGPTL2	angiopoietin like 2
1.32	FAM175A	family with sequence similarity 175. member A
1.32	TMCC2	transmembrane and coiled-coil domain family 2
1.32	FEZF1	FEZ family zinc finger 1
1.32	USP46	ubiquitin specific peptidase 46
1.32	ZNF567	zinc finger protein 567
1.32	SETD9	SET domain containing 9
1.32	GPR89B	G protein-coupled receptor 89B
1.32	RPL9	ribosomal protein L9
1.32	THRAP3	Jeck2013 ALT_ACCEPTOR. ALT_DONOR. coding. INTERNAL. intronic best transcript NM_005119
1.32	JAKMIP2-AS1	JAKMIP2 antisense RNA 1
1.32	COA7	cytochrome c oxidase assembly factor 7 (putative)
1.32	POGLUT1	protein O-glucosyltransferase 1
1.32	TOMM34	translocase of outer mitochondrial membrane 34
1.32	CTNNA3	catenin (cadherin-associated protein). alpha 3

1.32	SVIP	small VCP/p97-interacting protein
1.32	TMEM92	transmembrane protein 92
1.32	HSD17B4	hydroxysteroid (17-beta) dehydrogenase 4
1.32	PRLH	prolactin releasing hormone
1.32	CD3EAP	CD3e molecule. epsilon associated protein
1.32	TXLNGY	taxilin gamma pseudogene. Y-linked
1.32	EZR	ezrin
1.32	KLF12	Kruppel-like factor 12
1.32	GFRA4	GDNF family receptor alpha 4
1.32	PAN3; RNU6-82P	PAN3 poly(A) specific ribonuclease subunit; RNA. U6 small nuclear 82. pseudogene
1.32	FURIN	furin (paired basic amino acid cleaving enzyme)
1.32	NIFK	nucleolar protein interacting with the FHA domain of MKI67
1.32	TM4SF1	transmembrane 4 L six family member 1
1.32	JMJD6	jumonji domain containing 6
1.31	POC1B; POC1B-GALNT4; GALNT4	POC1 centriolar protein B; POC1B-GALNT4 readthrough; polypeptide N-acetylgalactosaminyltransferase 4
1.31	VARS	valyl-tRNA synthetase
1.31	PLA2G2A	phospholipase A2. group IIA (platelets. synovial fluid)
1.31	ZBTB10	zinc finger and BTB domain containing 10
1.31	GABARAPL1	GABA(A) receptor-associated protein like 1
1.31	IL2	interleukin 2
1.31	NETO2	neuropilin (NRP) and tolloid (TLL)-like 2
1.31	CTTNBP2NL	CTTNBP2 N-terminal like
1.31	ABCC1	ATP binding cassette subfamily C member 1
1.31	FAM126B	family with sequence similarity 126. member B
1.31	DTNA	dystrobrevin. alpha

1.31	SIK1	salt-inducible kinase 1
1.31	FMOD	fibromodulin
1.31	CCT6A	chaperonin containing TCP1. subunit 6A (zeta 1)
1.31	RAB43	Memczak2013 ALT_ACCEPTOR. ALT_DONOR. coding. INTERNAL. intronic best transcript NM_001204883
1.31	FAM27E3	family with sequence similarity 27. member E3
1.31	PAFAH1B1	platelet-activating factor acetylhydrolase 1b. regulatory subunit 1 (45kDa)
1.31	SNAPIN	SNAP-associated protein
1.31	CRIM1	cysteine rich transmembrane BMP regulator 1 (chordin-like)
1.31	CSMD1	Transcript Identified by AceView. Entrez Gene ID(s) 64478
1.31	TEX14	testis expressed 14
1.31	PTP4A2	protein tyrosine phosphatase type IVA. member 2
1.31	LRRC30	leucine rich repeat containing 30
1.31	PPL	periplakin
1.31	SOS2	SOS Ras/Rho guanine nucleotide exchange factor 2
1.31	PPM1B	Memczak2013 ANTISENSE. coding. INTERNAL. intronic best transcript NM_001033556
1.31	RBBP5	retinoblastoma binding protein 5
1.31	FASTKD2	FAST kinase domains 2
1.31	ISYNA1	inositol-3-phosphate synthase 1
1.31	ARID3A	AT rich interactive domain 3A (BRIGHT-like)
1.31	GPKOW	G-patch domain and KOW motifs
1.31	SPHK1	sphingosine kinase 1
1.31	NVL	nuclear VCP-like
1.31	TNFRSF10B	tumor necrosis factor receptor superfamily. member 10b
1.31	EHBP1	EH domain binding protein 1
1.31	SYNJ2BP-COX16; COX16;	SYNJ2BP-COX16 readthrough; COX16 cytochrome c oxidase assembly

	SYNJ2BP	homolog; synaptojanin 2 binding protein
1.31	ASCC3	activating signal cointegrator 1 complex subunit 3
1.31	FAM204A	family with sequence similarity 204. member A
1.31	ALG5	ALG5. dolichyl-phosphate beta-glucosyltransferase
1.31	TRAPPC6B	trafficking protein particle complex 6B
1.31	MDM2	MDM2 proto-oncogene. E3 ubiquitin protein ligase
1.31	OR6K2	olfactory receptor. family 6. subfamily K. member 2
1.31	BTNL2	butyrophilin-like 2
1.31	ETS1	v-ets avian erythroblastosis virus E26 oncogene homolog 1
1.31	GTF2F2	general transcription factor IIF subunit 2
1.31	PGLYRP4	peptidoglycan recognition protein 4
1.31	MAP1LC3B	microtubule-associated protein 1 light chain 3 beta
1.31	SPATA32	spermatogenesis associated 32
1.31	ZNF674	zinc finger protein 674
1.31	LOC151121; AC079586.1	uncharacterized LOC151121; novel transcript
1.31	SMYD2	SET and MYND domain containing 2
1.31	TBC1D9	TBC1 domain family. member 9 (with GRAM domain)
1.31	BAZ2B	bromodomain adjacent to zinc finger domain 2B
1.31	LILRA2	leukocyte immunoglobulin-like receptor. subfamily A (with TM domain). member 2
1.31	ZNF330	zinc finger protein 330
1.31	BRPF3	bromodomain and PHD finger containing 3
1.31	RPS4X	Zhang2013 ALT_ACCEPTOR. ALT_DONOR. coding. INTERNAL. intronic best transcript NM_001007
1.31	GBP5	guanylate binding protein 5
1.31	ZNF449	zinc finger protein 449
1.31	PIP4K2A	phosphatidylinositol-5-phosphate 4-kinase. type II. alpha

1.31	CALCOCO2	calcium binding and coiled-coil domain 2
1.31	GABARAPL2	GABA(A) receptor-associated protein like 2
1.31	ANKH	ANKH inorganic pyrophosphate transport regulator
1.31	CEBPZ	CCAAT/enhancer binding protein (C/EBP). zeta
1.31	DDX3Y	DEAD (Asp-Glu-Ala-Asp) box helicase 3. Y-linked
1.31	EPS8L3	EPS8-like 3
1.31	MESDC1	mesoderm development candidate 1
1.31	TNFRSF1A	tumor necrosis factor receptor superfamily. member 1A
1.31	ANP32B	acidic nuclear phosphoprotein 32 family member B
1.31	TIMM22	translocase of inner mitochondrial membrane 22 homolog (yeast)
1.31	XPO6	exportin 6
1.31	REXO4	REX4 homolog. 3'-5' exonuclease [Source:HGNC Symbol;Acc:HGNC:12820]
1.31	YES1	YES proto-oncogene 1. Src family tyrosine kinase
1.31	EED; MIR6755	embryonic ectoderm development; microRNA 6755
1.31	SERP1	stress-associated endoplasmic reticulum protein 1
1.31	ASPH	aspartate beta-hydroxylase
1.31	VPS33B	vacuolar protein sorting 33 homolog B (yeast)
1.31	CYP2C18	cytochrome P450. family 2. subfamily C. polypeptide 18
1.31	CYTH3	cytohesin 3
1.31	ACER1	alkaline ceramidase 1
1.31	PPP5C	protein phosphatase 5. catalytic subunit
1.30	S100A2	S100 calcium binding protein A2
1.30	ZNF502	zinc finger protein 502
1.30	DEFA1B; DEFA1; DEFA3	defensin. alpha 1B; defensin. alpha 1; defensin. alpha 3. neutrophil-specific
1.30	DEFA1; DEFA1B	defensin. alpha 1; defensin. alpha 1B
1.30	SAG	S-antigen; retina and pineal gland (arrestin)

1.30	CLN8	ceroid-lipofuscinosis. neuronal 8
1.30	ETV4	ets variant 4
1.30	ARL4A	ADP-ribosylation factor like GTPase 4A
1.30	ALOX5AP	arachidonate 5-lipoxygenase-activating protein
1.30	DDX19B	DEAD (Asp-Glu-Ala-Asp) box polypeptide 19B
1.30	NDUFS5	NADH dehydrogenase (ubiquinone) Fe-S protein 5. 15kDa (NADH-coenzyme Q reductase)
1.30	CDC14A	cell division cycle 14A
1.30	UCN2	urocortin 2
1.30	TKT	transketolase
1.30	PPARD	peroxisome proliferator-activated receptor delta
1.30	FAM45A	family with sequence similarity 45. member A
1.30	TRIM15	tripartite motif containing 15
1.30	RABEP1	rabaptin. RAB GTPase binding effector protein 1
1.30	ALPI	alkaline phosphatase. intestinal
1.30	PAPPA2	pappalysin 2
1.30	SCN3A	sodium channel. voltage gated. type III alpha subunit
1.30	OSBPL7	oxysterol binding protein-like 7
1.30	TNFRSF10A	tumor necrosis factor receptor superfamily. member 10a
1.30	DNAJC11	Zhang2013 ALT_ACCEPTOR. ALT_DONOR. coding. INTERNAL. intronic best transcript NM_018198
1.30	CAMKV	CaM kinase-like vesicle-associated
1.30	FLII	flightless I actin binding protein
1.30	HKR1	Memczak2013 ANTISENSE. CDS. coding. INTERNAL best transcript NM_181786
1.30	YRDC	yrdC N(6)-threonylcarbamoyltransferase domain containing
1.30	ITPRIPL2	inositol 1.4.5-trisphosphate receptor interacting protein-like 2

1.30	PREPL	prolyl endopeptidase-like
1.30	CFLAR	CASP8 and FADD like apoptosis regulator
1.30	TMEM39B	transmembrane protein 39B
1.30	TBC1D2B	TBC1 domain family. member 2B
1.30	L3MBTL3	l(3)mbt-like 3 (Drosophila)
1.30	SLC1A6	solute carrier family 1 (high affinity aspartate/glutamate transporter). member 6
1.30	HEPHL1	hephaestin-like 1
1.30	RBM41	RNA binding motif protein 41
1.30	SMAP1	small ArfGAP 1
1.30	INPP5D	inositol polyphosphate-5-phosphatase D
1.30	TACC1	transforming. acidic coiled-coil containing protein 1
1.30	HOMER1	homer scaffolding protein 1
1.30	TRIM61	tripartite motif containing 61
1.30	LINC00273	long intergenic non-protein coding RNA 273
1.30	F8A3	coagulation factor VIII-associated 3
1.30	CAT	Memczak2013 ANTISENSE. CDS. coding. INTERNAL. UTR3 best transcript NM_001752
1.30	CT47A5; CT47A2; CT47A1; CT47A11; CT47A12	cancer/testis antigen family 47. member A5; cancer/testis antigen family 47. member A2; cancer/testis antigen family 47. member A1; cancer/testis antigen family 47. member A11; cancer/testis antigen family 47. member A12
1.30	STK24	serine/threonine kinase 24
1.30	PARD6B	par-6 family cell polarity regulator beta
1.30	UFM1	ubiquitin-fold modifier 1
1.30	DEFB131	defensin. beta 131
1.30	POFUT1; MIR1825	protein O-fucosyltransferase 1; microRNA 1825
1.30	CD79A	CD79a molecule. immunoglobulin-associated alpha

1.30	KLHL2	kelch-like family member 2
1.30	DMTF1	cyclin D binding myb-like transcription factor 1
1.30	REXO4	REX4 homolog. 3-5 exonuclease
1.30	FMO1	Transcript Identified by AceView. Entrez Gene ID(s) 2326
1.30	ITGB5	integrin beta 5
1.30	CLGN	calmegin
1.30	KRT14	keratin 14. type I
1.30	FBXO18	F-box protein. helicase. 18
1.30	DNAL1	dynein. axonemal. light chain 1
1.30	CERS5	ceramide synthase 5
1.30	BNIP2	BCL2/adenovirus E1B 19kDa interacting protein 2
1.30	VPS37A	vacuolar protein sorting 37 homolog A (S. cerevisiae)
1.30	TARS2; MIR6878	threonyl-tRNA synthetase 2. mitochondrial (putative); microRNA 6878
1.30	PKN2	protein kinase N2
1.30	PAX3	paired box 3
1.30	PGA4	pepsinogen 4. group I (pepsinogen A)
1.30	TMEM60	transmembrane protein 60
1.30	PEMT	phosphatidylethanolamine N-methyltransferase
1.30	TESC	tescalcin
1.30	FAT3	FAT atypical cadherin 3
1.30	SH3D19	SH3 domain containing 19
1.30	DGKG	diacylglycerol kinase gamma
1.30	WTIP	Wilms tumor 1 interacting protein
1.30	DDAH2	dimethylarginine dimethylaminohydrolase 2
1.30	SNX10	sorting nexin 10
1.30	OR4A16	olfactory receptor. family 4. subfamily A. member 16

1.30	EEF1B2; SNORA41	eukaryotic translation elongation factor 1 beta 2; small nucleolar RNA. H/ACA box 41
1.30	HEBP2	heme binding protein 2
1.30	ENDOD1	endonuclease domain containing 1
1.30	PIGBOS1; RAB27A	PIGB opposite strand 1; RAB27A. member RAS oncogene family
1.30	FOXJ2	forkhead box J2
1.30	SRC	SRC proto-oncogene. non-receptor tyrosine kinase
1.30	SURF2	surfeit 2
1.30	ANKRD36	Transcript Identified by AceView. Entrez Gene ID(s) 375248
1.30	OLAH	oleoyl-ACP hydrolase
1.30	STMN4	stathmin-like 4
1.30	SLC6A14	solute carrier family 6 (amino acid transporter). member 14
1.30	ALOX12	arachidonate 12-lipoxygenase
1.30	SLC48A1	solute carrier family 48 (heme transporter). member 1
1.30	CERS1; GDF1	ceramide synthase 1; growth differentiation factor 1
1.30	RBM47	RNA binding motif protein 47
1.30	RPS3A; SNORD73A	ribosomal protein S3A; small nucleolar RNA. C/D box 73A
1.30	PNO1	partner of NOB1 homolog
1.30	NSG1; D4S234E	neuron specific gene family member 1; Neuron-specific protein family member 1 [Source:UniProtKB/Swiss-Prot;Acc:P42857]
1.30	SPANXN2	SPANX family. member N2
1.30	MEN1	multiple endocrine neoplasia I
1.30	KBTBD12	kelch repeat and BTB (POZ) domain containing 12
1.30	DUSP16	dual specificity phosphatase 16
1.30	MRPL1	mitochondrial ribosomal protein L1
1.30	TRIM8	tripartite motif containing 8
1.29	ZNF473	zinc finger protein 473

1.29	LRIG1	
1.29	KCTD20	potassium channel tetramerization domain containing 20
1.29	RNF166	ring finger protein 166
1.29	NKRF	NFKB repressing factor
1.29	ZNF354B	zinc finger protein 354B
1.29	C3orf17	chromosome 3 open reading frame 17
1.29	ODC1; SNORA80B	ornithine decarboxylase 1; small nucleolar RNA. H/ACA box 80B
1.29	INPP5D	inositol polyphosphate-5-phosphatase D
1.29	SUCO	SUN domain containing ossification factor
1.29	UTP4	UTP4 small subunit (SSU) processome component
1.29	ZFYVE9	zinc finger. FYVE domain containing 9
1.29	CUEDC1	CUE domain containing 1
1.29	MPHOSPH6	M-phase phosphoprotein 6
1.29	EFCAB3	EF-hand calcium binding domain 3
1.29	NUDT13	nudix hydrolase 13
1.29	STK10	serine/threonine kinase 10
1.29	CDC37; MIR1181	cell division cycle 37; microRNA 1181
1.29	PRR19	proline rich 19
1.29	CT62	cancer/testis antigen 62
1.29	ILK	integrin linked kinase
1.29	ZDHHC21	zinc finger. DHHC-type containing 21
1.29	SCN4A	sodium channel. voltage gated. type IV alpha subunit
1.29	PRSS22	protease. serine. 22
1.29	CTAGE1	cutaneous T-cell lymphoma-associated antigen 1
1.29	RD3L	retinal degeneration 3-like
1.29	QSER1	glutamine and serine rich 1

1.29	KIF13A	kinesin family member 13A
1.29	OR4E2	olfactory receptor. family 4. subfamily E. member 2
1.29	CUL1	cullin 1
1.29	STAT5B	Transcript Identified by AceView. Entrez Gene ID(s) 6777
1.29	TAAR5	trace amine associated receptor 5
1.29	KRT20	keratin 20. type I
1.29	PA2G4	proliferation-associated 2G4
1.29	CBFB	core-binding factor. beta subunit
1.29	DHX33	DEAH (Asp-Glu-Ala-His) box polypeptide 33
1.29	TNFRSF11A	tumor necrosis factor receptor superfamily. member 11a. NFKB activator
1.29	ZMIZ1	zinc finger. MIZ-type containing 1
1.29	TATDN1; MIR6844	TatD DNase domain containing 1; microRNA 6844
1.29	GPR39	G protein-coupled receptor 39
1.29	EMC8	ER membrane protein complex subunit 8
1.29	ZG16B	zymogen granule protein 16B
1.29	SFT2D2	SFT2 domain containing 2
1.29	KLHL8	kelch-like family member 8
1.29	CCL26	chemokine (C-C motif) ligand 26
1.29	BOP1; MIR7112	block of proliferation 1; microRNA 7112
1.29	ATP8B4	ATPase. class I. type 8B. member 4
1.29	MAP4K3	mitogen-activated protein kinase kinase kinase kinase 3
1.29	RPS9	ribosomal protein S9
1.29	STAP1	signal transducing adaptor family member 1
1.29	MBNL2	muscleblind-like splicing regulator 2
1.29	RSPH4A	radial spoke head 4 homolog A (Chlamydomonas)
1.29	TAF4B	TAF4b RNA polymerase II. TATA box binding protein (TBP)-associated factor.

		105kDa
1.29	TBC1D12	TBC1 domain family. member 12
1.29	CDC42EP1	CDC42 effector protein (Rho GTPase binding) 1
1.29	ZNF622	zinc finger protein 622
1.29	CAMK2N1	calcium/calmodulin-dependent protein kinase II inhibitor 1
1.29	SLC39A2	solute carrier family 39 (zinc transporter). member 2
1.29	GNL3; SNORD19B	guanine nucleotide binding protein-like 3 (nucleolar); small nucleolar RNA. C/D box 19B
1.29	USP45	ubiquitin specific peptidase 45
1.29	EPPIN	epididymal peptidase inhibitor
1.29	SETD2	SET domain containing 2
1.29	BAZ2A	bromodomain adjacent to zinc finger domain 2A
1.29	TMCC3; MIR7844	transmembrane and coiled-coil domain family 3; microRNA 7844
1.29	WWC1	WW and C2 domain containing 1
1.29	PGAP1	post-GPI attachment to proteins 1
1.29	FAM122B	family with sequence similarity 122B
1.29	TGIF1	TGFB-induced factor homeobox 1
1.29	TTLL3	tubulin tyrosine ligase-like family member 3
1.29	UBE3A	ubiquitin protein ligase E3A
1.29	FAM90A1	family with sequence similarity 90. member A1
1.29	CC2D2A	Transcript Identified by AceView. Entrez Gene ID(s) 57545
1.29	JPH2	junctophilin 2
1.29	MT1HL1	metallothionein 1H-like 1
1.29	USP47	ubiquitin specific peptidase 47
1.29	NOLC1	nucleolar and coiled-body phosphoprotein 1
1.29	ABHD17C	abhydrolase domain containing 17C

1.29	TTI2	TELO2 interacting protein 2
1.29	YAP1	Yes-associated protein 1
1.29	ZNF214	zinc finger protein 214
1.29	FABP5	fatty acid binding protein 5 (psoriasis-associated)
1.29	ANKRD49	ankyrin repeat domain 49
1.29	SNX16	sorting nexin 16
1.29	CDC42	cell division cycle 42
1.29	TNFSF4	tumor necrosis factor (ligand) superfamily. member 4
1.29	ABCA10	ATP binding cassette subfamily A member 10
1.29	SCML2	sex comb on midleg-like 2 (Drosophila)
1.29	ABCA5	ATP binding cassette subfamily A member 5
1.29	RNF187	ring finger protein 187
1.29	SCAMP1	secretory carrier membrane protein 1
1.29	CFHR5	complement factor H-related 5
1.29	MICALCL	MICAL C-terminal like
1.29	ZIC5	Zic family member 5
1.29	PPFIBP1	PTPRF interacting protein. binding protein 1 (liprin beta 1)
1.29	SNX5	Zhang2013 ALT_ACCEPTOR. ALT_DONOR. coding. INTERNAL. intronic best transcript NM_152227
1.29	RBM18	RNA binding motif protein 18
1.29	NANOGNB	NANOG neighbor homeobox
1.29	PAGE5	P antigen family. member 5 (prostate associated)
1.29	SPANXC; SPANXD	SPANX family. member C; SPANX family. member D
1.29	OR6C2	olfactory receptor. family 6. subfamily C. member 2
1.29	TYRP1	tyrosinase-related protein 1
1.29	SYTL4	synaptotagmin-like 4

1.29	TRAPPC12	trafficking protein particle complex 12
1.29	TAF1D; SNORA8; SNORA18; SNORA40; SNORA1; SNORA25; SNORA32; SNORD5; MIR1304	TATA box binding protein associated factor 1D; small nucleolar RNA. H/ACA box 8; small nucleolar RNA. H/ACA box 18; small nucleolar RNA. H/ACA box 40; small nucleolar RNA. H/ACA box 1; small nucleolar RNA. H/ACA box 25; small nucleolar RNA. H/ACA box 32; small nucleolar RNA. C/D box 5; microRNA 1304
1.28	NAV2	neuron navigator 2
1.28	RDX	radixin
1.28	ZDHHC13	zinc finger. DHHC-type containing 13
1.28	MRTO4	MRT4 homolog. ribosome maturation factor
1.28	MSMP	microseminoprotein. prostate associated
1.28	BATF	basic leucine zipper transcription factor. ATF-like
1.28	TMEM59	transmembrane protein 59
1.28	DENND5A	DENN/MADD domain containing 5A
1.28	DEFB106B; DEFB106A	defensin. beta 106B; defensin. beta 106A
1.28	CSMD1	CUB and Sushi multiple domains 1
1.28	CHST15	carbohydrate (N-acetylgalactosamine 4-sulfate 6-O) sulfotransferase 15
1.28	CD63	CD63 molecule
1.28	CCL21	chemokine (C-C motif) ligand 21
1.28	CLIP1	CAP-GLY domain containing linker protein 1
1.28	C2orf88	chromosome 2 open reading frame 88
1.28	TMOD3	tropomodulin 3 (ubiquitous)
1.28	L1CAM	L1 cell adhesion molecule
1.28	PPP1R1C	protein phosphatase 1. regulatory (inhibitor) subunit 1C
1.28	HGS	hepatocyte growth factor-regulated tyrosine kinase substrate
1.28	TFRC	transferrin receptor
1.28	PCDH20	protocadherin 20

1.28	NUAK1	NUAK family. SNF1-like kinase. 1
1.28	HAO1	hydroxyacid oxidase (glycolate oxidase) 1
1.28	PCBP4	poly(rC) binding protein 4
1.28	C1S	complement component 1. s subcomponent
1.28	FAM118B	family with sequence similarity 118. member B
1.28	CCT8	chaperonin containing TCP1. subunit 8 (theta)
1.28	FXYD5	FXYD domain containing ion transport regulator 5
1.28	TRPC1	transient receptor potential cation channel. subfamily C. member 1
1.28	PRDM11	PR domain containing 11
1.28	MECR	mitochondrial trans-2-enoyl-CoA reductase
1.28	TM4SF1-AS1	TM4SF1 antisense RNA 1
1.28	USP4	ubiquitin specific peptidase 4 (proto-oncogene)
1.28	TMEM5	transmembrane protein 5
1.28	TGIF2-C20orf24	TGIF2-C20orf24 readthrough
1.28	RAB30	RAB30. member RAS oncogene family
1.28	URGCP	upregulator of cell proliferation
1.28	PTH2R	parathyroid hormone 2 receptor
1.28	AIP	aryl hydrocarbon receptor interacting protein
1.28	SMIM12	small integral membrane protein 12
1.28	DYDC2	DPY30 domain containing 2
1.28	C2orf49	chromosome 2 open reading frame 49
1.28	PACS1	phosphofurin acidic cluster sorting protein 1
1.28	LIPT1	lipoyltransferase 1
1.28	MKRN2	makorin ring finger protein 2
1.28	NRAS	neuroblastoma RAS viral (v-ras) oncogene homolog
1.28	HPRT1	hypoxanthine phosphoribosyltransferase 1

1.28	PRRG4	proline rich Gla (G-carboxyglutamic acid) 4 (transmembrane)
1.28	PLAG1	pleiomorphic adenoma gene 1
1.28	NEURL4	neuralized E3 ubiquitin protein ligase 4
1.28	ZNF252P-AS1	ZNF252P antisense RNA 1
1.28	MTO1	mitochondrial tRNA translation optimization 1
1.28	SLC51A	solute carrier family 51. alpha subunit
1.28	XRCC5	Memczak2013 ALT_ACCEPTOR. ALT_DONOR. coding. INTERNAL. intronic best transcript NM_021141
1.28	DOT1L	DOT1-like histone H3K79 methyltransferase
1.28	PROSER1	proline and serine rich 1
1.28	ZNF865	zinc finger protein 865
1.28	XG; XGY2	Xg blood group; Xg pseudogene. Y-linked 2
1.28	AKR1C2	aldo-keto reductase family 1. member C2
1.28	KLF13	Kruppel-like factor 13
1.28	ZNF131	zinc finger protein 131
1.28	GOLGA6L3	golgin A6 family-like 3
1.28	LRRC72	leucine rich repeat containing 72
1.28	FAM216A	family with sequence similarity 216. member A
1.28	RPL18A	ribosomal protein L18a
1.28	GSTP1	glutathione S-transferase pi 1
1.28	NUPL2	nucleoporin like 2
1.28	GGT6	gamma-glutamyltransferase 6
1.28	OPA1	optic atrophy 1 (autosomal dominant)
1.28	CSNK1E	casein kinase 1. epsilon
1.28	ITPR1	inositol 1.4.5-trisphosphate receptor. type 1
1.28	CCT2	chaperonin containing TCP1. subunit 2 (beta)

1.28	TMED1	transmembrane p24 trafficking protein 1
1.28	RPL15	ribosomal protein L15
1.28	DGKH	diacylglycerol kinase. eta
1.28	DEFB105A	defensin. beta 105A
1.28	FADS3	fatty acid desaturase 3
1.28	TRMT1	tRNA methyltransferase 1
1.28	GPR25	G protein-coupled receptor 25
1.28	ARID2	AT rich interactive domain 2 (ARID. RFX-like)
1.28	FKBP5	FK506 binding protein 5
1.28	CA3	carbonic anhydrase III
1.28	EIF3M	eukaryotic translation initiation factor 3. subunit M
1.28	FAM104A	family with sequence similarity 104. member A
1.28	TOP3B	topoisomerase (DNA) III beta
1.27	IL1RAPL1	interleukin 1 receptor accessory protein-like 1
1.27	KDSR	3-ketodihydrosphingosine reductase
1.27	FASTKD3	FAST kinase domains 3
1.27	STC2	stanniocalcin 2
1.27	ZNF382	zinc finger protein 382
1.27	KMT5A	lysine (K)-specific methyltransferase 5A
1.27	SAMD4A	sterile alpha motif domain containing 4A
1.27	PHLDB1; MIR6716	pleckstrin homology-like domain. family B. member 1; microRNA 6716
1.27	ADAMTS7	ADAM metallopeptidase with thrombospondin type 1 motif 7
1.27	SYF2	SYF2 pre-mRNA-splicing factor
1.27	ATG16L1	autophagy related 16-like 1
1.27	METTL21B	methyltransferase like 21B
1.27	IMMP1L	inner mitochondrial membrane peptidase subunit 1

1.27	C4BPB	complement component 4 binding protein. beta
1.27	ZNF133	zinc finger protein 133
1.27	FTSJ1	FtsJ RNA methyltransferase homolog 1 (E. coli)
1.27	PDGFC	platelet derived growth factor C
1.27	RPL18	ribosomal protein L18
1.27	DOPEY1	dopey family member 1
1.27	DYNLT1	dynein. light chain. Tctex-type 1
1.27	TBCE	tubulin folding cofactor E
1.27	USP28	ubiquitin specific peptidase 28
1.27	GGT1	gamma-glutamyltransferase 1
1.27	MTMR9	myotubularin related protein 9
1.27	NUDCD1	NudC domain containing 1
1.27	MOSPD2	motile sperm domain containing 2
1.27	MORN4	MORN repeat containing 4
1.27	FRRS1	ferric-chelate reductase 1
1.27	STXBP5	syntaxin binding protein 5 (tomosyn)
1.27	RFX3	regulatory factor X. 3 (influences HLA class II expression)
1.27	OR2Y1	olfactory receptor. family 2. subfamily Y. member 1
1.27	ZSCAN26	zinc finger and SCAN domain containing 26
1.27	ALKBH3; SEC14L1P1	alkB homolog 3. alpha-ketoglutarate-dependent dioxygenase; SEC14-like 1 pseudogene 1
1.27	FAM169A	family with sequence similarity 169. member A
1.27	THG1L	tRNA-histidine guanylyltransferase 1-like
1.27	UQCC2	ubiquinol-cytochrome c reductase complex assembly factor 2
1.27	HIGD2A	HIG1 hypoxia inducible domain family. member 2A
1.27	ELP2	elongator acetyltransferase complex subunit 2

1.27	OXA1L	oxidase (cytochrome c) assembly 1-like
1.27	ZNF852	zinc finger protein 852
1.27	ECHDC3	enoyl-CoA hydratase domain containing 3
1.27	NR1D1	nuclear receptor subfamily 1. group D. member 1
1.27	PHLPP2	PH domain and leucine rich repeat protein phosphatase 2
1.27	F8A2; F8A3; F8A1	coagulation factor VIII-associated 2; coagulation factor VIII-associated 3; coagulation factor VIII-associated 1
1.27	ADPRH	ADP-ribosylarginine hydrolase
1.27	CSNK1E	casein kinase 1. epsilon
1.27	TMEM38B	transmembrane protein 38B
1.27	RBM34	RNA binding motif protein 34
1.27	ZNFX1	zinc finger. NFX1-type containing 1
1.27	OR1L4	olfactory receptor. family 1. subfamily L. member 4
1.27	LRRCC1	Transcript Identified by AceView. Entrez Gene ID(s) 85444
1.27	BEND6	BEN domain containing 6
1.27	FUBP3	Zhang2013 ALT_ACCEPTOR. ALT_DONOR. coding. INTERNAL. intronic best transcript NM_003934
1.27	NPIPB3	nuclear pore complex interacting protein family. member B3
1.27	GRIN3B	glutamate receptor. ionotropic. N-methyl-D-aspartate 3B
1.27	TNFRSF17	tumor necrosis factor receptor superfamily. member 17
1.27	MPP3	membrane protein. palmitoylated 3
1.27	FAM214A	family with sequence similarity 214. member A
1.27	SYCP3	synaptonemal complex protein 3
1.27	ZNF768	zinc finger protein 768
1.27	IRGQ	immunity-related GTPase family. Q
1.27	FOXD2	forkhead box D2
1.27	RAB42	RAB42. member RAS oncogene family

1.27	ZNF181	zinc finger protein 181
1.27	HRCT1	histidine rich carboxyl terminus 1
1.27	HSDL1	hydroxysteroid dehydrogenase like 1
1.27	DDX31	DEAD (Asp-Glu-Ala-Asp) box polypeptide 31
1.27	CCDC13	coiled-coil domain containing 13
1.27	WDR70	WD repeat domain 70
1.27	CDK6	cyclin-dependent kinase 6
1.27	KATNAL2	katanin p60 subunit A-like 2
1.27	SLC16A3; MIR6787	solute carrier family 16 (monocarboxylate transporter). member 3; microRNA 6787
1.27	PEX7	peroxisomal biogenesis factor 7
1.27	RBM22	RNA binding motif protein 22
1.27	RHOC	ras homolog family member C
1.27	ACAP2	ArfGAP with coiled-coil. ankyrin repeat and PH domains 2
1.27	PPP1R3B	protein phosphatase 1. regulatory subunit 3B
1.27	RAPGEF4	Rap guanine nucleotide exchange factor 4
1.27	TAB1	TGF-beta activated kinase 1/MAP3K7 binding protein 1
1.27	MAPK6	mitogen-activated protein kinase 6
1.27	RPL4; SNORD16; SNORD18A; SNORD18B; SNORD18C	ribosomal protein L4; small nucleolar RNA. C/D box 16; small nucleolar RNA. C/D box 18A; small nucleolar RNA. C/D box 18B; small nucleolar RNA. C/D box 18C
1.27	PANX1	pannexin 1
1.27	PRPF39	pre-mRNA processing factor 39
1.27	PIK3CB	phosphatidylinositol-4.5-bisphosphate 3-kinase. catalytic subunit beta
1.27	LEO1	LEO1 homolog. Paf1/RNA polymerase II complex component
1.27	RPS27L	ribosomal protein S27-like
1.27	RTN4	reticulon 4

1.27	COQ10A	coenzyme Q10A
1.27	ADAM23	ADAM metallopeptidase domain 23
1.27	EFNA3	ephrin-A3
1.27	PIGG	phosphatidylinositol glycan anchor biosynthesis class G
1.27	TMEM254	transmembrane protein 254
1.27	RBM5	RNA binding motif protein 5
1.27	GPR157	G protein-coupled receptor 157
1.27	CPA2	carboxypeptidase A2 (pancreatic)
1.27	INPPL1	inositol polyphosphate phosphatase-like 1
1.27	OR8H3	olfactory receptor. family 8. subfamily H. member 3
1.27	NPIPB4	nuclear pore complex interacting protein family. member B4
1.27	GNB5	guanine nucleotide binding protein (G protein). beta 5
1.27	NCOA7	nuclear receptor coactivator 7
1.27	GLS	glutaminase
1.27	EPC1	enhancer of polycomb homolog 1 (Drosophila)
1.27	B3GNTL1	UDP-GlcNAc:betaGal beta-1.3-N-acetylglucosaminyltransferase-like 1
1.27	ZCCHC17	zinc finger. CCHC domain containing 17
1.27	NTNG1	netrin G1
1.27	DLEU1	deleted in lymphocytic leukemia 1 (non-protein coding)
1.27	UHRF2	ubiquitin-like with PHD and ring finger domains 2. E3 ubiquitin protein ligase
1.27	AARS	alanyl-tRNA synthetase
1.27	RHOD	ras homolog family member D
1.26	PPP2R3C	protein phosphatase 2. regulatory subunit B. gamma
1.26	SERINC5	Memczak2013 ALT_ACCEPTOR. ALT_DONOR. coding. INTERNAL. intronic best transcript NM_001174072
1.26	SPATA31C2	SPATA31 subfamily C. member 2

1.26	SH2B2	SH2B adaptor protein 2
1.26	NOCT	nocturnin
1.26	MEX3C	mex-3 RNA binding family member C
1.26	BBX	bobby sox homolog (Drosophila)
1.26	FCHO2	FCH domain only 2
1.26	KIF2A	kinesin heavy chain member 2A
1.26	EMC7	ER membrane protein complex subunit 7
1.26	ZNF572	zinc finger protein 572
1.26	IER5	immediate early response 5
1.26	JAK2	Memczak2013 ALT_ACCEPTOR. ALT_DONOR. coding. INTERNAL. intronic best transcript NM_004972
1.26	GPR15	G protein-coupled receptor 15
1.26	STRIP1	striatin interacting protein 1
1.26	PKD2L2	polycystic kidney disease 2-like 2
1.26	STX6	syntaxin 6
1.26	OAS3	2-5-oligoadenylate synthetase 3
1.26	EXOC2	exocyst complex component 2
1.26	SCG5	secretogranin V
1.26	PRAMEF10	PRAME family member 10
1.26	INPP5D	inositol polyphosphate-5-phosphatase D
1.26	TNKS1BP1	tankyrase 1 binding protein 1
1.26	MAP3K19	mitogen-activated protein kinase kinase kinase 19
1.26	SHOC2	SHOC2 leucine-rich repeat scaffold protein
1.26	MAP9	microtubule-associated protein 9
1.26	ALS2CR12	amyotrophic lateral sclerosis 2 chromosome region candidate 12
1.26	ZNF185	zinc finger protein 185 (LIM domain)

1.26	PLXND1	plexin D1
1.26	EIF1	eukaryotic translation initiation factor 1
1.26	SSX6	synovial sarcoma. X breakpoint 6 (pseudogene)
1.26	ZBED5	zinc finger. BED-type containing 5
1.26	PDLIM7	PDZ and LIM domain 7 (enigma)
1.26	LRRC75A	leucine rich repeat containing 75A
1.26	TMCO3	transmembrane and coiled-coil domains 3
1.26	RPL12	ribosomal protein L12
1.26	TUBGCP3	Transcript Identified by AceView. Entrez Gene ID(s) 10426
1.26	DUSP6	dual specificity phosphatase 6
1.26	CCDC71	coiled-coil domain containing 71
1.26	PELO; ITGA1	pelota homolog (Drosophila); integrin alpha 1
1.26	NKX6-1	NK6 homeobox 1
1.26	CHD7	chromodomain helicase DNA binding protein 7
1.26	ZNF33B	zinc finger protein 33B
1.26	LGALS12	lectin. galactoside-binding. soluble. 12
1.26	C16orf78	chromosome 16 open reading frame 78
1.26	SCGB3A1	secretoglobin. family 3A. member 1
1.26	GBA3	Jeck2013 ANTISENSE. coding. INTERNAL. intronic best transcript NM_020973
1.26	MBD3L2	methyl-CpG binding domain protein 3-like 2
1.26	GYG1	glycogenin 1
1.26	WDR73	WD repeat domain 73
1.26	ATP13A5	ATPase type 13A5
1.26	CACHD1	cache domain containing 1
1.26	KRT26	keratin 26. type I
1.26	PRR33	proline rich 33

1.26	TRPV2	transient receptor potential cation channel. subfamily V. member 2
1.26	ITCH	itchy E3 ubiquitin protein ligase
1.26	LOC100287225; RP11-267C16.1	uncharacterized LOC100287225; novel transcript
1.26	AAR2	AAR2 splicing factor homolog
1.26	UQCRC2	ubiquinol-cytochrome c reductase core protein II
1.26	VWA8; MIR5006	von Willebrand factor A domain containing 8; microRNA 5006
1.26	SPATA12	spermatogenesis associated 12
1.26	ABCC5	ATP binding cassette subfamily C member 5
1.26	INPP5D	inositol polyphosphate-5-phosphatase D
1.26	GRAMD1A	GRAM domain containing 1A
1.26	DEFB107A; DEFB107B	defensin. beta 107A; defensin. beta 107B
1.26	RFPL4AL1	ret finger protein-like 4A-like 1
1.26	ANKRD28	ankyrin repeat domain 28
1.26	TSG101	tumor susceptibility 101
1.26	ALG11; UTP14C	ALG11. alpha-1.2-mannosyltransferase; UTP14. U3 small nucleolar ribonucleoprotein. homolog C (yeast)
1.26	RPS8; SNORD55; SNORD46; SNORD38A; SNORD38B	ribosomal protein S8; small nucleolar RNA. C/D box 55; small nucleolar RNA. C/D box 46; small nucleolar RNA. C/D box 38A; small nucleolar RNA. C/D box 38B
1.26	GPR89A	G protein-coupled receptor 89A
1.26	NSMCE2	NSE2/MMS21 homolog. SMC5-SMC6 complex SUMO ligase
1.26	GDAP1L1	ganglioside induced differentiation associated protein 1-like 1
1.26	CNTNAP3P2; CNTNAP3	contactin associated protein-like 3 pseudogene 2; contactin associated protein-like 3
1.26	TCHP	trichoplein. keratin filament binding
1.26	SLC25A32	solute carrier family 25 (mitochondrial folate carrier). member 32
1.26	ZDHHC23	zinc finger. DHHC-type containing 23

1.26	ALDH1L2	aldehyde dehydrogenase 1 family. member L2
1.26	LOC102724323; RP11-67C2.2; MARCH8	uncharacterized LOC102724323; Transcript Identified by AceView. Entrez Gene ID(s) 220972; novel transcript
1.26	RNF11	ring finger protein 11
1.26	RNFT1	ring finger protein. transmembrane 1
1.26	ZNF12	zinc finger protein 12
1.26	NDUFA9	NADH dehydrogenase (ubiquinone) 1 alpha subcomplex. 9. 39kDa
1.26	SAAL1	serum amyloid A-like 1
1.26	C19orf38	chromosome 19 open reading frame 38
1.26	PLLP	plasmolipin
1.26	DVL1; MIR6808	dishevelled segment polarity protein 1; microRNA 6808
1.26	SYNJ2	synaptojanin 2
1.26	TICAM1	toll-like receptor adaptor molecule 1
1.26	SH3KBP1	SH3-domain kinase binding protein 1
1.26	RAB11A	RAB11A. member RAS oncogene family
1.26	TEX33	testis expressed 33
1.26	ZNF546	zinc finger protein 546
1.26	ADAP2	ArfGAP with dual PH domains 2
1.26	KEAP1	kelch-like ECH-associated protein 1
1.26	PTPN14	protein tyrosine phosphatase. non-receptor type 14
1.26	DSC2	desmocollin 2
1.26	PHF3	PHD finger protein 3
1.26	ZNF653	zinc finger protein 653
1.26	C11orf1	chromosome 11 open reading frame 1
1.26	C20orf24	chromosome 20 open reading frame 24
1.26	RAP2B	RAP2B. member of RAS oncogene family

1.26	TMEM40	transmembrane protein 40
1.26	ARFRP1	ADP-ribosylation factor related protein 1
1.26	C15orf62	chromosome 15 open reading frame 62
1.26	TMEM140	transmembrane protein 140
1.26	PPIE	peptidylprolyl isomerase E (cyclophilin E)
1.26	TMEM55A	transmembrane protein 55A
1.26	C9orf62	chromosome 9 open reading frame 62
1.26	DNAH6	Transcript Identified by AceView. Entrez Gene ID(s) 1768
1.26	ZNF423	zinc finger protein 423
1.26	LDLR; MIR6886	low density lipoprotein receptor; microRNA 6886
1.26	KIAA1462	KIAA1462
1.26	CXCL5	chemokine (C-X-C motif) ligand 5
1.26	PDCD5	programmed cell death 5
1.26	ZDHHC3	zinc finger. DHHC-type containing 3
1.26	DIO2	deiodinase. iodothyronine. type II
1.26	PI4KB	phosphatidylinositol 4-kinase. catalytic. beta
1.25	PNLIPRP3	pancreatic lipase-related protein 3
1.25	ARSE	arylsulfatase E (chondrodysplasia punctata 1)
1.25	GRIN2A	Transcript Identified by AceView. Entrez Gene ID(s) 2903
1.25	CNEP1R1	CTD nuclear envelope phosphatase 1 regulatory subunit 1
1.25	DOPEY2	dopey family member 2
1.25	DCP2	decapping mRNA 2
1.25	MLXIP	MLX interacting protein
1.25	N4BP2	NEDD4 binding protein 2
1.25	TMEM209	transmembrane protein 209
1.25	PLD4	phospholipase D family. member 4

1.25	KIAA0232	KIAA0232
1.25	RNF138	ring finger protein 138. E3 ubiquitin protein ligase
1.25	RPL26	ribosomal protein L26
1.25	GEM	GTP binding protein overexpressed in skeletal muscle
1.25	CCT8L2	chaperonin containing TCP1. subunit 8 (theta)-like 2
1.25	ZNF750	zinc finger protein 750
1.25	IGFL3	IGF like family member 3
1.25	CD59	CD59 molecule. complement regulatory protein
1.25	ZNF706	zinc finger protein 706
1.25	BACE2	beta-site APP-cleaving enzyme 2
1.25	LGALS14	lectin. galactoside-binding. soluble. 14
1.25	C3orf79	chromosome 3 open reading frame 79
1.25	MRPS31	mitochondrial ribosomal protein S31
1.25	HLA-DRA	major histocompatibility complex. class II. DR alpha
1.25	ZFAND5	zinc finger. AN1-type domain 5
1.25	SPIN2A	spindlin family. member 2A
1.25	SENP8	SUMO/sentrin peptidase family member. NEDD8 specific
1.25	HIP1R	huntingtin interacting protein 1 related
1.25	CDKAL1	Jeck2013 ALT_ACCEPTOR. ALT_DONOR. coding. INTERNAL. intronic best transcript NM_017774
1.25	KMT2A	lysine (K)-specific methyltransferase 2A
1.25	SERINC1	serine incorporator 1
1.25	C2orf42	chromosome 2 open reading frame 42
1.25	TMEM244	transmembrane protein 244
1.25	STARD3NL	STARD3 N-terminal like
1.25	ABLIM1	actin binding LIM protein 1

1.25	SMAD3	SMAD family member 3
1.25	BRWD1	bromodomain and WD repeat domain containing 1
1.25	UBXN2A	UBX domain protein 2A
1.25	TIFAB	TRAF-interacting protein with forkhead-associated domain. family member B
1.25	FAM219B	family with sequence similarity 219. member B
1.25	VPS13D	vacuolar protein sorting 13 homolog D (S. cerevisiae)
1.25	FAM57A	family with sequence similarity 57. member A
1.25	NAB2	NGFI-A binding protein 2 (EGR1 binding protein 2)
1.25	SOCS2	suppressor of cytokine signaling 2
1.25	APOBEC3D	apolipoprotein B mRNA editing enzyme. catalytic polypeptide-like 3D
1.25	RAN	RAN. member RAS oncogene family
1.25	KCTD21	potassium channel tetramerization domain containing 21
1.25	EEF1E1	eukaryotic translation elongation factor 1 epsilon 1
1.25	GOLGA6L5P; GOLGA6L17P	golgin A6 family-like 5. pseudogene; golgin A6 family-like 17. pseudogene
1.25	LPAR6	lysophosphatidic acid receptor 6
1.25	ANKRD30BL	ankyrin repeat domain 30B-like
1.25	FRMD5	FERM domain containing 5
1.25	ROCK1	Rho-associated. coiled-coil containing protein kinase 1
1.25	YOD1	YOD1 deubiquitinase
1.25	ST8SIA3	ST8 alpha-N-acetyl-neuraminide alpha-2.8-sialyltransferase 3
1.25	SSFA2	sperm specific antigen 2
1.25	SESN1	sestrin 1
1.25	TNNI1	troponin I type 1 (skeletal. slow)
1.25	TRMT11	tRNA methyltransferase 11 homolog
1.25	MRVI1	murine retrovirus integration site 1 homolog
1.25	IFT46	intraflagellar transport 46

1.25	ZNF780A	zinc finger protein 780A
1.25	APLF	aprataxin and PNKP like factor
1.25	TNFRSF11B	tumor necrosis factor receptor superfamily. member 11b
1.25	RRM2B	ribonucleotide reductase M2 B (TP53 inducible)
1.25	C16orf72	Memczak2013 ANTISENSE. coding. INTERNAL. intronic best transcript NM_014117
1.25	IMPDH1	IMP (inosine 5-monophosphate) dehydrogenase 1
1.25	LINC01219	long intergenic non-protein coding RNA 1219
1.25	GKAP1	G kinase anchoring protein 1
1.25	TBL1XR1	transducin (beta)-like 1 X-linked receptor 1
1.25	RRAGC	Memczak2013 ANTISENSE. coding. INTERNAL. intronic best transcript NM_022157
1.25	GLTP	glycolipid transfer protein
1.25	POLG2	polymerase (DNA directed). gamma 2. accessory subunit
1.25	KLHL9	kelch-like family member 9
1.25	FGFR3	fibroblast growth factor receptor 3
1.25	WDR43; SNORD53	WD repeat domain 43; small nucleolar RNA. C/D box 53
1.25	MIR205HG; MIR205	MIR205 host gene; microRNA 205
1.25	CLCN7	chloride channel. voltage-sensitive 7
1.25	INPP5E	inositol polyphosphate-5-phosphatase E
1.25	RPH3AL	rabphilin 3A-like (without C2 domains)
1.25	EPB41L3	erythrocyte membrane protein band 4.1-like 3
1.25	KTN1	kinectin 1 (kinesin receptor)
1.25	PWP1	PWP1 homolog. endonuclein
1.25	NOL4	nucleolar protein 4
1.25	ARHGEF4	Rho guanine nucleotide exchange factor 4
1.25	QDPR	quinoid dihydropteridine reductase

1.25	FCER1G	Fc fragment of IgE. high affinity I. receptor for; gamma polypeptide
1.25	FLOT1	flotillin 1
1.25	SSC5D	scavenger receptor cysteine rich family. 5 domains
1.25	OR4C6	olfactory receptor. family 4. subfamily C. member 6
1.25	COX7A2	cytochrome c oxidase subunit VIIa polypeptide 2 (liver)
1.25	TRAPPC3L	trafficking protein particle complex 3-like
1.25	XIAP	X-linked inhibitor of apoptosis. E3 ubiquitin protein ligase
1.25	MYNN	myoneurin
1.25	LRRC69; MIR4661	leucine rich repeat containing 69; microRNA 4661
1.25	HMGN5	high mobility group nucleosome binding domain 5
1.25	FAAP100	Fanconi anemia core complex associated protein 100
1.25	CNTNAP3B	contactin associated protein-like 3B
1.25	TTC9	tetratricopeptide repeat domain 9
1.25	ELOVL1; MIR6734	ELOVL fatty acid elongase 1; microRNA 6734
1.25	ZMYM4	zinc finger. MYM-type 4
1.25	CNTN1	contactin 1
1.25	ZCCHC11	zinc finger. CCHC domain containing 11
1.25	NGLY1	N-glycanase 1
1.25	SNX24	sorting nexin 24
1.25	RDH11	retinol dehydrogenase 11 (all-trans/9-cis/11-cis)
1.25	KIAA1586	KIAA1586
1.25	FBXL13	F-box and leucine-rich repeat protein 13
1.25	IRS2	insulin receptor substrate 2
1.25	STK40	serine/threonine kinase 40
1.25	NT5C3A	5-nucleotidase. cytosolic IIIA
1.25	TMEM262	transmembrane protein 262

1.25	ZNF280D	zinc finger protein 280D
1.25	ASB11	ankyrin repeat and SOCS box containing 11. E3 ubiquitin protein ligase
1.25	PSEN2	presenilin 2
1.25	OCIAD1	OCIA domain containing 1
1.25	FAM50A	family with sequence similarity 50. member A
1.25	NEMF	nuclear export mediator factor
1.25	GSR	glutathione reductase
1.25	UBA3	ubiquitin-like modifier activating enzyme 3
1.25	MRPL42	mitochondrial ribosomal protein L42
1.25	ARHGEF16	Rho guanine nucleotide exchange factor 16
1.25	SFTPA1	surfactant protein A1
1.25	CTPS2	Transcript Identified by AceView. Entrez Gene ID(s) 56474
1.25	SPANXB1	SPANX family. member B1
1.25	UVRAG	UV radiation resistance associated
1.25	CEBPB	CCAAT/enhancer binding protein (C/EBP). beta
1.24	NME1; NME1-NME2; NME2	NME/NM23 nucleoside diphosphate kinase 1; NME1-NME2 readthrough; NME/NM23 nucleoside diphosphate kinase 2
1.24	DDX46	DEAD (Asp-Glu-Ala-Asp) box polypeptide 46
1.24	MAB21L2	mab-21-like 2 (C. elegans)
1.24	RPTN	repetin
1.24	DCUN1D5	DCN1. defective in cullin neddylation 1. domain containing 5
1.24	RRP15	ribosomal RNA processing 15 homolog
1.24	HSPBAP1	HSPB (heat shock 27kDa) associated protein 1
1.24	DSC3	desmocollin 3
1.24	TK2	thymidine kinase 2. mitochondrial
1.24	BECN2	beclin 2

1.24	RBBP8; MIR4741	retinoblastoma binding protein 8; microRNA 4741
1.24	GPATCH4	G-patch domain containing 4
1.24	TRAF3IP2	TRAF3 interacting protein 2
1.24	FMNL2	formin like 2
1.24	SPANXA2	SPANX family. member A2
1.24	PVRL4	poliovirus receptor-related 4
1.24	NCEH1	neutral cholesterol ester hydrolase 1
1.24	INTS5	integrator complex subunit 5
1.24	OR2A4	olfactory receptor. family 2. subfamily A. member 4
1.24	MUM1	melanoma associated antigen (mutated) 1
1.24	UGT2B4	UDP glucuronosyltransferase 2 family. polypeptide B4
1.24	LLPH	LLP homolog. long-term synaptic facilitation (Aplysia)
1.24	SLC8A1	solute carrier family 8 (sodium/calcium exchanger). member 1
1.24	ATF4	activating transcription factor 4
1.24	CCDC73	coiled-coil domain containing 73
1.24	ERVV-1	endogenous retrovirus group V. member 1
1.24	SCAI; GOLGA1	suppressor of cancer cell invasion; golgin A1
1.24	RANBP3L	RAN binding protein 3-like
1.24	NAF1	nuclear assembly factor 1 ribonucleoprotein
1.24	CS	citrate synthase
1.24	CAMTA1	calmodulin binding transcription activator 1
1.24	RANBP9	RAN binding protein 9
1.24	VWA5B2	von Willebrand factor A domain containing 5B2
1.24	MTPAP	mitochondrial poly(A) polymerase
1.24	MTHFD2L	methylenetetrahydrofolate dehydrogenase (NADP+ dependent) 2-like
1.24	CLLU1	chronic lymphocytic leukemia up-regulated 1

1.24	SRFBP1	serum response factor binding protein 1
1.24	TCEB3	transcription elongation factor B (SIII). polypeptide 3 (110kDa. elongin A)
1.24	ADPRM	ADP-ribose/CDP-alcohol diphosphatase. manganese-dependent
1.24	APOLD1; DDX47	apolipoprotein L domain containing 1; DEAD (Asp-Glu-Ala-Asp) box polypeptide 47
1.24	HSP90B1	Transcript Identified by AceView. Entrez Gene ID(s) 7184
1.24	RPTOR	regulatory associated protein of MTOR. complex 1
1.24	ATIC	5-aminoimidazole-4-carboxamide ribonucleotide formyltransferase/IMP cyclohydrolase
1.24	AVPR1B	arginine vasopressin receptor 1B
1.24	ZNF282	zinc finger protein 282
1.24	ERGIC2	ERGIC and golgi 2
1.24	RPP21	ribonuclease P/MRP 21kDa subunit
1.24	DMXL2	Dmx-like 2
1.24	KIAA1109	KIAA1109
1.24	ATP5L2	ATP synthase. H+ transporting. mitochondrial Fo complex subunit G2
1.24	TUFT1	tuftelin 1
1.24	DYNAP	dynactin associated protein
1.24	PADI4	peptidyl arginine deiminase. type IV
1.24	KIAA1211	KIAA1211
1.24	FAM27L	family with sequence similarity 27-like
1.24	MAP4K5	mitogen-activated protein kinase kinase kinase kinase 5
1.24	BLOC1S2	biogenesis of lysosomal organelles complex-1. subunit 2
1.24	ATP5L	ATP synthase. H+ transporting. mitochondrial Fo complex subunit G
1.24	DPT	dermatopontin
1.24	ZNF767P	zinc finger family member 767. pseudogene
1.24	CCBE1	collagen and calcium binding EGF domains 1

1.24	LAP3	Transcript Identified by AceView. Entrez Gene ID(s) 51056
1.24	MYF5	myogenic factor 5
1.24	SH2D3C	SH2 domain containing 3C
1.24	ZNF496	zinc finger protein 496
1.24	ATG16L1	autophagy related 16-like 1
1.24	CTPS1	CTP synthase 1
1.24	SLC6A6	solute carrier family 6 (neurotransmitter transporter). member 6
1.24	KPRP	keratinocyte proline-rich protein
1.24	ORC4	origin recognition complex subunit 4
1.24	HARS	histidyl-tRNA synthetase
1.24	TRMT13	tRNA methyltransferase 13 homolog (S. cerevisiae)
1.24	HTR5A	5-hydroxytryptamine (serotonin) receptor 5A. G protein-coupled
1.24	TEAD2	TEA domain family member 2
1.24	TRAF6	TNF receptor-associated factor 6. E3 ubiquitin protein ligase
1.24	SYN2	synapsin II
1.24	LRFN2	leucine rich repeat and fibronectin type III domain containing 2
1.24	SNX9	sorting nexin 9
1.24	DSG2	desmoglein 2
1.24	ZNF354A	zinc finger protein 354A
1.24	BBC3; MIR3191	BCL2 binding component 3; microRNA 3191
1.24	ERO1A	endoplasmic reticulum oxidoreductase alpha
1.24	GLI1	GLI family zinc finger 1
1.24	TWF2	twinfilin actin binding protein 2
1.24	ATP2C2	ATPase. Ca++ transporting. type 2C. member 2
1.24	ATG16L1	autophagy related 16-like 1
1.24	B3GALT4	UDP-Gal:betaGlcNAc beta 1.3-galactosyltransferase 4

1.24	SMIM21	small integral membrane protein 21
1.24	RAB11FIP2	RAB11 family interacting protein 2 (class I)
1.24	C6orf201	chromosome 6 open reading frame 201
1.24	KCNE2	potassium channel. voltage gated subfamily E regulatory beta subunit 2
1.24	MGEA5	meningioma expressed antigen 5 (hyaluronidase)
1.24	PPP1R15B	protein phosphatase 1. regulatory subunit 15B
1.24	SUGT1	SGT1 homolog. MIS12 kinetochore complex assembly cochaperone
1.24	ANKRD13D	ankyrin repeat domain 13 family. member D
1.24	CLTB	clathrin. light chain B
1.24	DNAJB14	DnaJ (Hsp40) homolog. subfamily B. member 14
1.24	FAM208B	family with sequence similarity 208. member B
1.24	ARL14	ADP-ribosylation factor like GTPase 14
1.24	NUP133	nucleoporin 133kDa
1.24	PLEK	pleckstrin
1.24	MPP1	membrane protein. palmitoylated 1
1.24	C6orf10	chromosome 6 open reading frame 10
1.24	ATG12	autophagy related 12
1.24	MTA1	metastasis associated 1
1.24	SP140	SP140 nuclear body protein
1.24	KAT6B	K(lysine) acetyltransferase 6B
1.24	TLR4	toll-like receptor 4
1.24	SAMD9L	sterile alpha motif domain containing 9-like
1.24	GTF3C6	general transcription factor IIIC subunit 6
1.24	FAM197Y1	family with sequence similarity 197. Y-linked. member 1
1.24	TSTD1	thiosulfate sulfurtransferase (rhodanese)-like domain containing 1
1.24	KAT6B	K(lysine) acetyltransferase 6B

1.24	EIF1B	eukaryotic translation initiation factor 1B
1.24	COX7C; MIR3607	cytochrome c oxidase subunit VIIc; microRNA 3607
1.24	MRPS35	mitochondrial ribosomal protein S35
1.24	ATG16L1	autophagy related 16-like 1
1.24	MYO1B	myosin IB
1.24	NKAP	NFKB activating protein
1.24	ELF1	Transcript Identified by AceView. Entrez Gene ID(s) 1997; 100128628
1.24	ZNF112	zinc finger protein 112
1.24	SRGN	serglycin
1.24	MR1	major histocompatibility complex. class I-related
1.24	CREBL2	cAMP responsive element binding protein-like 2
1.24	CCDC110	coiled-coil domain containing 110
1.24	GTPBP4	GTP binding protein 4
1.24	MAX	MYC associated factor X
1.24	ARPP21	cAMP-regulated phosphoprotein 21kDa
1.24	CREB1	cAMP responsive element binding protein 1
1.24	FAM81A	family with sequence similarity 81. member A
1.23	ZNF205	zinc finger protein 205
1.23	SYPL1	synaptophysin-like 1
1.23	NKIRAS2	NFKB inhibitor interacting Ras-like 2
1.23	IL15	interleukin 15
1.23	ATF5; MIR4751	activating transcription factor 5; microRNA 4751
1.23	KLK1	kallikrein 1
1.23	C2orf70	chromosome 2 open reading frame 70
1.23	CPA1	carboxypeptidase A1 (pancreatic)
1.23	KRT15; MIR6510	keratin 15. type I; microRNA 6510

1.23	TRPM6	transient receptor potential cation channel. subfamily M. member 6
1.23	PKD2L1	polycystic kidney disease 2-like 1
1.23	SUSD3	sushi domain containing 3
1.23	INPP5A	Transcript Identified by AceView. Entrez Gene ID(s) 3632
1.23	PTGER4	prostaglandin E receptor 4 (subtype EP4)
1.23	SLC26A5	solute carrier family 26 (anion exchanger). member 5
1.23	MKRN2OS	MKRN2 opposite strand
1.23	BEX5	brain expressed X-linked 5
1.23	DLG5	discs. large homolog 5 (Drosophila)
1.23	TGM4	transglutaminase 4
1.23	CCDC86	coiled-coil domain containing 86
1.23	PSMD2	proteasome 26S subunit. non-ATPase 2
1.23	CRY1	cryptochrome circadian clock 1
1.23	CNBP	CCHC-type zinc finger. nucleic acid binding protein
1.23	RFX4	Transcript Identified by AceView. Entrez Gene ID(s) 5992
1.23	JTB	jumping translocation breakpoint
1.23	NUP205	nucleoporin 205kDa
1.23	HERC4	HECT and RLD domain containing E3 ubiquitin protein ligase 4
1.23	ITK	IL2-inducible T-cell kinase
1.23	FAM129A	family with sequence similarity 129. member A
1.23	RPL10A	ribosomal protein L10a
1.23	RECK	reversion-inducing-cysteine-rich protein with kazal motifs
1.23	RAB23	RAB23. member RAS oncogene family
1.23	PCDH19	protocadherin 19
1.23	LINC01219	long intergenic non-protein coding RNA 1219
1.23	TRIM16L	tripartite motif containing 16-like

1.23	RGS20	regulator of G-protein signaling 20
1.23	TRAPPC10	trafficking protein particle complex 10
1.23	LRIG1	
1.23	TRIM48	tripartite motif containing 48
1.23	YWHAH	tyrosine 3-monooxygenase/tryptophan 5-monooxygenase activation protein. eta
1.23	SAMD5	sterile alpha motif domain containing 5
1.23	SYK	Memczak2013 ANTISENSE. CDS. coding. INTERNAL best transcript NM_001174167
1.23	C18orf25	chromosome 18 open reading frame 25
1.23	IPO11; LRRC70	importin 11; leucine rich repeat containing 70
1.23	CLK4	CDC like kinase 4
1.23	POLRMT	polymerase (RNA) mitochondrial (DNA directed)
1.23	APOBEC3H	apolipoprotein B mRNA editing enzyme. catalytic polypeptide-like 3H
1.23	ISCU	iron-sulfur cluster assembly enzyme
1.23	PDE4A	phosphodiesterase 4A. cAMP-specific
1.23	CTNNA1	catenin (cadherin-associated protein). alpha 1
1.23	PSME4	proteasome activator subunit 4
1.23	LAPTM4B	lysosomal protein transmembrane 4 beta
1.23	ATP6V0A4	ATPase. H+ transporting. lysosomal V0 subunit a4
1.23	ANAPC7	anaphase promoting complex subunit 7
1.23	IRAK2	interleukin 1 receptor associated kinase 2
1.23	RPS12	ribosomal protein S12
1.23	MLXIP	MLX interacting protein
1.23	ZSWIM4	Transcript Identified by AceView. Entrez Gene ID(s) 65249
1.23	HCFC2	host cell factor C2
1.23	MOCOS	molybdenum cofactor sulfurase

1.23	CD44	Memczak2013 ALT_ACCEPTOR. ALT_DONOR. coding. INTERNAL. intronic best transcript NM_001202557
1.23	POLR3E	polymerase (RNA) III (DNA directed) polypeptide E (80kD)
1.23	THADA	Transcript Identified by AceView. Entrez Gene ID(s) 63892
1.23	VAMP5	vesicle associated membrane protein 5
1.23	IL9	interleukin 9
1.23	OR10A4	olfactory receptor. family 10. subfamily A. member 4
1.23	TBRG4; SNORA5B	transforming growth factor beta regulator 4; small nucleolar RNA. H/ACA box 5B
1.23	ENPP1	ectonucleotide pyrophosphatase/phosphodiesterase 1
1.23	RAB31	RAB31. member RAS oncogene family
1.23	NR4A1	nuclear receptor subfamily 4. group A. member 1
1.23	ATN1	atrophin 1
1.23	NLK	nemo-like kinase
1.23	INSL6	insulin-like 6
1.23	TMEM133	transmembrane protein 133
1.23	ERLIN1	ER lipid raft associated 1
1.23	KANSL2; SNORA2B	KAT8 regulatory NSL complex subunit 2; small nucleolar RNA. H/ACA box 2B
1.23	ARMC8	armadillo repeat containing 8
1.23	TEX10	testis expressed 10
1.23	TGS1	trimethylguanosine synthase 1
1.23	DSCAM	Down syndrome cell adhesion molecule
1.23	PUS3	pseudouridylate synthase 3
1.23	OR2S2	olfactory receptor. family 2. subfamily S. member 2 (gene/pseudogene)
1.23	MFN1	mitofusin 1
1.23	MRPL35	mitochondrial ribosomal protein L35
1.23	SURF6	surfeit 6

1.23	PIR	pirin
1.23	MTIF2	mitochondrial translational initiation factor 2
1.23	LRP6	LDL receptor related protein 6
1.23	BMP5	bone morphogenetic protein 5
1.23	AKTIP	AKT interacting protein
1.23	METTL2B	methyltransferase like 2B
1.23	STAU2	staufen double-stranded RNA binding protein 2
1.23	HPCA	hippocalcin
1.23	FAXDC2	fatty acid hydroxylase domain containing 2
1.23	SESTD1	SEC14 and spectrin domains 1
1.23	ASB5	ankyrin repeat and SOCS box containing 5
1.23	SENP6	SUMO1/sentrin specific peptidase 6
1.23	ZMYM1	zinc finger. MYM-type 1
1.23	ZFP69	ZFP69 zinc finger protein
1.23	SULT2B1	sulfotransferase family 2B member 1
1.23	ABTB2	ankyrin repeat and BTB (POZ) domain containing 2
1.23	ISG15	ISG15 ubiquitin-like modifier
1.23	TMEM164	transmembrane protein 164
1.23	KIF7	Memczak2013 ANTISENSE. CDS. coding. INTERNAL best transcript NM_198525
1.23	HCCS	holocytochrome c synthase
1.23	GNGT1	guanine nucleotide binding protein (G protein). gamma transducing activity polypeptide 1
1.23	SOX11	SRY box 11
1.23	EIF1AX	eukaryotic translation initiation factor 1A. X-linked
1.23	RNF19A	ring finger protein 19A. RBR E3 ubiquitin protein ligase
1.23	APP	amyloid beta (A4) precursor protein

1.23	UTP3	UTP3. small subunit (SSU) processome component. homolog (S. cerevisiae)
1.23	MEF2D	myocyte enhancer factor 2D
1.23	NSFL1C	NSFL1 (p97) cofactor (p47)
1.23	AF131215.3; XKR6	Memczak2013 ALT_ACCEPTOR. ALT_DONOR. coding. INTERNAL. intronic best transcript NM_173683; novel transcript
1.23	WDR1	WD repeat domain 1
1.23	CNNM4	cyclin and CBS domain divalent metal cation transport mediator 4
1.23	IKBKB	inhibitor of kappa light polypeptide gene enhancer in B-cells. kinase beta
1.23	RNF115	ring finger protein 115
1.23	RORA	RAR-related orphan receptor A
1.23	COPS2	COP9 signalosome subunit 2
1.23	OR52R1	olfactory receptor. family 52. subfamily R. member 1 (gene/pseudogene)
1.23	TMEM185B	transmembrane protein 185B
1.23	TMEM167A	Transcript Identified by AceView. Entrez Gene ID(s) 153339
1.23	PLCD3	phospholipase C. delta 3
1.23	PALM2	paralemmin 2
1.23	ATG16L1	autophagy related 16-like 1
1.23	ZNF197	zinc finger protein 197
1.23	CD3G	CD3g molecule. gamma (CD3-TCR complex)
1.23	SERHL2	serine hydrolase-like 2
1.23	TMEM31	transmembrane protein 31
1.23	C14orf166	chromosome 14 open reading frame 166
1.23	SLC16A1	solute carrier family 16 (monocarboxylate transporter). member 1
1.23	POU6F2	POU class 6 homeobox 2
1.23	TET3	tet methylcytosine dioxygenase 3
1.23	EML2; MIR330	echinoderm microtubule associated protein like 2; microRNA 330

1.23	AZIN2	antizyme inhibitor 2
1.23	TCF12	transcription factor 12
1.23	FAM120A	family with sequence similarity 120A
1.23	PPP4R3B	protein phosphatase 4. regulatory subunit 3B
1.23	EEPD1	endonuclease/exonuclease/phosphatase family domain containing 1
1.23	SPRR3	small proline-rich protein 3
1.23	CHKA	choline kinase alpha
1.23	IRX2	iroquois homeobox 2
1.23	HIF1AN	hypoxia inducible factor 1. alpha subunit inhibitor
1.22	OR5A2	olfactory receptor. family 5. subfamily A. member 2
1.22	GPD2	glycerol-3-phosphate dehydrogenase 2
1.22	SRPRB	signal recognition particle receptor. B subunit
1.22	GOT2	glutamic-oxaloacetic transaminase 2. mitochondrial
1.22	FAM47E; FAM47E-STBD1; STBD1	family with sequence similarity 47. member E; FAM47E-STBD1 readthrough; starch binding domain 1
1.22	ATG16L1	autophagy related 16-like 1
1.22	LOC400927; CSNK1E	TPTE and PTEN homologous inositol lipid phosphatase pseudogene; Transcript Identified by AceView. Entrez Gene ID(s) 1454; 400927
1.22	C1orf61; RP11-98G7.1	Transcript Identified by AceView. Entrez Gene ID(s) 10485; novel transcript
1.22	RSL1D1	ribosomal L1 domain containing 1
1.22	GPATCH1	G-patch domain containing 1
1.22	TSTD3	thiosulfate sulfurtransferase (rhodanese)-like domain containing 3
1.22	CT47A9; CT47A4; CT47A11	cancer/testis antigen family 47. member A9; cancer/testis antigen family 47. member A4; cancer/testis antigen family 47. member A11
1.22	CT47A3; CT47A10	cancer/testis antigen family 47. member A3; cancer/testis antigen family 47. member A10
1.22	CT47A10; CT47A8; CT47A9	cancer/testis antigen family 47. member A10; cancer/testis antigen family 47. member A8; cancer/testis antigen family 47. member A9

1.22	CT47A6; CT47A4	cancer/testis antigen family 47. member A6; cancer/testis antigen family 47. member A4
1.22	CT47A3	cancer/testis antigen family 47. member A3
1.22	CT47A2	cancer/testis antigen family 47. member A2
1.22	CT47A12; CT47A8	cancer/testis antigen family 47. member A12; cancer/testis antigen family 47. member A8
1.22	CALB2	calbindin 2
1.22	CCRL2	chemokine (C-C motif) receptor-like 2
1.22	PSMB8	proteasome subunit beta 8
1.22	MAPK11	mitogen-activated protein kinase 11
1.22	TCF4	transcription factor 4
1.22	SPX	spexin hormone
1.22	SQRDL	sulfide quinone reductase-like (yeast)
1.22	FAM3C	family with sequence similarity 3. member C
1.22	INHBE	inhibin beta E
1.22	HSPA6	heat shock 70kDa protein 6 (HSP70B)
1.22	AFAP1L2	actin filament associated protein 1-like 2
1.22	SORBS1	Transcript Identified by AceView. Entrez Gene ID(s) 10580
1.22	CCDC171	coiled-coil domain containing 171
1.22	AP3M1	adaptor-related protein complex 3. mu 1 subunit
1.22	MYL10	myosin light chain 10
1.22	SLC25A5	solute carrier family 25 (mitochondrial carrier; adenine nucleotide translocator). member 5
1.22	DOCK10	dedicator of cytokinesis 10
1.22	DAZ2; DAZ3	deleted in azoospermia 2; deleted in azoospermia 3
1.22	DIRAS3	DIRAS family. GTP-binding RAS-like 3
1.22	TMEM87B	transmembrane protein 87B

1.22	NCAM1	neural cell adhesion molecule 1
1.22	ZNF2	zinc finger protein 2
1.22	RCC2	regulator of chromosome condensation 2
1.22	RAG2	recombination activating gene 2
1.22	SNAP91	synaptosome associated protein 91kDa
1.22	SF3B6	splicing factor 3b subunit 6
1.22	RSF1	remodeling and spacing factor 1
1.22	LINC01465	long intergenic non-protein coding RNA 1465
1.22	WDR54	WD repeat domain 54
1.22	DYM	dymeclin
1.22	RPS6KA4	ribosomal protein S6 kinase. 90kDa. polypeptide 4
1.22	HADHB	hydroxyacyl-CoA dehydrogenase/3-ketoacyl-CoA thiolase/enoyl-CoA hydratase (trifunctional protein). beta subunit
1.22	NPAS2	neuronal PAS domain protein 2
1.22	SNX12	sorting nexin 12
1.22	NIN	ninein (GSK3B interacting protein)
1.22	KCNJ3	potassium channel. inwardly rectifying subfamily J. member 3
1.22	ZFAND4	zinc finger. AN1-type domain 4
1.22	GRTP1	growth hormone regulated TBC protein 1
1.22	ZP1	zona pellucida glycoprotein 1 (sperm receptor)
1.22	GGT5	gamma-glutamyltransferase 5
1.22	CEP170	centrosomal protein 170kDa
1.22	SLC35E3	solute carrier family 35. member E3
1.22	IL21R	interleukin 21 receptor
1.22	ZNF780A	zinc finger protein 780A
1.22	MYH13	myosin. heavy chain 13. skeletal muscle

1.22	FAM155B	family with sequence similarity 155. member B
1.22	IPO5	importin 5
1.22	TOM1L1	target of myb1 like 1 membrane trafficking protein
1.22	TBCK	TBC1 domain containing kinase
1.22	PTBP3	polypyrimidine tract binding protein 3
1.22	RAPH1	Ras association (RalGDS/AF-6) and pleckstrin homology domains 1
1.22	ARHGAP15	Rho GTPase activating protein 15
1.22	CHST3	carbohydrate (chondroitin 6) sulfotransferase 3
1.22	NDUFA10	NADH dehydrogenase (ubiquinone) 1 alpha subcomplex. 10. 42kDa
1.22	C12orf76	chromosome 12 open reading frame 76
1.22	KRT33A	keratin 33A. type I
1.22	PABPC1; MIR7705	poly(A) binding protein. cytoplasmic 1; microRNA 7705
1.22	LRRC3B	leucine rich repeat containing 3B
1.22	PRELID2	PRELI domain containing 2
1.22	PROSER2	proline and serine rich 2
1.22	C18orf8	chromosome 18 open reading frame 8
1.22	OCA2	oculocutaneous albinism II
1.22	IRX5	iroquois homeobox 5
1.22	KRBOX4	KRAB box domain containing 4
1.22	SERPINB1	serpin peptidase inhibitor. clade B (ovalbumin). member 1
1.22	BMP8B	bone morphogenetic protein 8b
1.22	DGKD	diacylglycerol kinase. delta 130kDa
1.22	RCOR3	REST corepressor 3
1.22	POLR1C	polymerase (RNA) I polypeptide C
1.22	SAR1B	secretion associated. Ras related GTPase 1B
1.22	PLA1A	phospholipase A1 member A

1.22	REXO1; MIR1909	REX1. RNA exonuclease 1 homolog; microRNA 1909
1.22	IFNA21	interferon. alpha 21
1.22	ZBTB7A	zinc finger and BTB domain containing 7A
1.22	C10orf12	chromosome 10 open reading frame 12
1.22	GPM6B	glycoprotein M6B
1.22	RPS26	Homo sapiens ribosomal protein S26. mRNA (cDNA clone MGC:104291 IMAGE:4287636). complete cds.
1.22	OXR1	oxidation resistance 1
1.22	MCC	mutated in colorectal cancers
1.22	MGAM	maltase-glucoamylase
1.22	SMYD3	Jeck2013 ALT_ACCEPTOR. ALT_DONOR. coding. INTERNAL. intronic best transcript NM_001167740
1.22	CCDC175	coiled-coil domain containing 175
1.22	TIMM17A	translocase of inner mitochondrial membrane 17 homolog A (yeast)
1.22	DCUN1D3	DCN1. defective in cullin neddylation 1. domain containing 3
1.22	LOC79999; LOC388436; AC007952.6	Homo sapiens uncharacterized LOC79999 (LOC79999). mRNA.; Homo sapiens uncharacterized protein ENSP00000382042 (LOC388436). mRNA.; novel transcript antisense to GRAPL
1.22	RGS2	regulator of G-protein signaling 2
1.22	DNTTIP2	deoxynucleotidyltransferase. terminal. interacting protein 2
1.22	NUP85	nucleoporin 85kDa
1.22	REXO4	REX4 homolog. 3'-5' exonuclease [Source:HGNC Symbol;Acc:HGNC:12820]
1.22	OR5A1	olfactory receptor. family 5. subfamily A. member 1
1.22	PLCB4	phospholipase C. beta 4
1.22	RPL7A	ribosomal protein L7a
1.22	RPS4X	ribosomal protein S4. X-linked
1.22	FAM26F	family with sequence similarity 26. member F

1.22	AEBP2	AE binding protein 2
1.22	RPS6KA2	ribosomal protein S6 kinase. 90kDa. polypeptide 2
1.22	PANK4	pantothenate kinase 4
1.22	MAK16	MAK16 homolog
1.22	USP16	ubiquitin specific peptidase 16
1.22	VPS41	vacuolar protein sorting 41 homolog (S. cerevisiae)
1.22	PTPN12	protein tyrosine phosphatase. non-receptor type 12
1.22	HADHA	hydroxyacyl-CoA dehydrogenase/3-ketoacyl-CoA thiolase/enoyl-CoA hydratase (trifunctional protein). alpha subunit
1.22	PCDHB14	protocadherin beta 14
1.22	ADCY6; MIR4701	adenylate cyclase 6; microRNA 4701
1.22	ESRRA	estrogen-related receptor alpha
1.22	GFM2	G elongation factor. mitochondrial 2
1.22	ADAMTS4	ADAM metallopeptidase with thrombospondin type 1 motif 4
1.22	ACTL7A	actin-like 7A
1.22	SLC35G4	solute carrier family 35. member G4
1.22	USP17L10	ubiquitin specific peptidase 17-like family member 10
1.22	STAG3L4	stromal antigen 3-like 4 (pseudogene)
1.22	TSC22D4	TSC22 domain family. member 4
1.22	PPIE	Transcript Identified by AceView. Entrez Gene ID(s) 10450
1.22	RNASE6	ribonuclease. RNase A family. k6
1.22	ATG16L1	autophagy related 16-like 1
1.22	GMPS	guanine monophosphate synthase
1.22	HNRNPC	heterogeneous nuclear ribonucleoprotein C (C1/C2)
1.22	MX2	MX dynamin-like GTPase 2
1.22	USP13	ubiquitin specific peptidase 13 (isopeptidase T-3)

1.22	IFRD2	interferon-related developmental regulator 2
1.22	OR2T29	olfactory receptor. family 2. subfamily T. member 29
1.22	LOC100287728; RP11-85L21.4; jerva	uncharacterized LOC100287728; Transcript Identified by AceView; novel transcript
1.22	GOPC	golgi-associated PDZ and coiled-coil motif containing
1.22	RTP5	receptor (chemosensory) transporter protein 5 (putative)
1.22	MSRB3	methionine sulfoxide reductase B3
1.22	PLEKHM2	pleckstrin homology domain containing. family M (with RUN domain) member 2
1.22	CCDC191	coiled-coil domain containing 191
1.22	GRN	Memczak2013 ANTISENSE. CDS. coding. INTERNAL best transcript NM_002087
1.22	CFB	complement factor B
1.22	STMND1	stathmin domain containing 1
1.22	DOCK7	dedicator of cytokinesis 7
1.22	EXOC5	exocyst complex component 5
1.22	MEAF6	MYST/Esa1-associated factor 6
1.22	ZNF555	zinc finger protein 555
1.22	MRPS16	mitochondrial ribosomal protein S16
1.22	BCL2L14	BCL2-like 14 (apoptosis facilitator)
1.22	ZNF501	zinc finger protein 501 [Source:HGNC Symbol;Acc:HGNC:23717]
1.22	GREM1	gremlin 1. DAN family BMP antagonist
1.22	ERCC8	excision repair cross-complementation group 8
1.22	LPIN1; MIR548S	lipin 1; microRNA 548s
1.22	MED17	mediator complex subunit 17
1.22	REV3L	REV3 like. DNA directed polymerase zeta catalytic subunit
1.22	GFPT2	glutamine-fructose-6-phosphate transaminase 2

1.22	MSRA	methionine sulfoxide reductase A
1.22	CDRT1	CMT1A duplicated region transcript 1
1.22	TRAK1	trafficking protein. kinesin binding 1
1.22	PAH	phenylalanine hydroxylase
1.22	KIF17	kinesin family member 17
1.22	EFCAB9	EF-hand calcium binding domain 9
1.22	MIER3	mesoderm induction early response 1. family member 3
1.22	EIF3E	eukaryotic translation initiation factor 3. subunit E
1.22	AEN	apoptosis enhancing nuclease
1.22	EFCC1	EF-hand and coiled-coil domain containing 1
1.22	INPP5D	inositol polyphosphate-5-phosphatase D
1.22	CHN2	chimerin 2
1.22	SYN1	synapsin I
1.22	CD163L1	CD163 molecule-like 1
1.22	SNTG1	syntrophin. gamma 1
1.22	YBX1	Zhang2013 ALT_ACCEPTOR. ALT_DONOR. coding. INTERNAL. intronic best transcript NM_004559
1.22	DIP2A	disco-interacting protein 2 homolog A
1.22	APOBEC3G	apolipoprotein B mRNA editing enzyme. catalytic polypeptide-like 3G
1.22	NBAS	neuroblastoma amplified sequence
1.22	MASP1	mannan-binding lectin serine peptidase 1 (C4/C2 activating component of Ra-reactive factor)
1.22	ZNF28	zinc finger protein 28
1.21	GIP	gastric inhibitory polypeptide
1.21	OLFML1	olfactomedin like 1
1.21	ITFG1	integrin alpha FG-GAP repeat containing 1
1.21	OR4A8	olfactory receptor. family 4. subfamily A. member 8 (gene/pseudogene)

		[Source:HGNC Symbol;Acc:HGNC:15165]
1.21	PAXBP1	PAX3 and PAX7 binding protein 1
1.21	LIMK2	LIM domain kinase 2
1.21	NALCN	sodium leak channel. non selective
1.21	AK2	adenylate kinase 2
1.21	FHL1	four and a half LIM domains 1
1.21	TMBIM4	transmembrane BAX inhibitor motif containing 4
1.21	UTY	ubiquitously transcribed tetratricopeptide repeat containing. Y-linked
1.21	AUP1	ancient ubiquitous protein 1
1.21	WHSC1L1	Wolf-Hirschhorn syndrome candidate 1-like 1
1.21	KRTAP20-3	keratin associated protein 20-3
1.21	ADAMTS13	ADAM metallopeptidase with thrombospondin type 1 motif 13
1.21	C18orf21	chromosome 18 open reading frame 21
1.21	LAMA3	laminin. alpha 3
1.21	KAZALD1	Kazal-type serine peptidase inhibitor domain 1
1.21	SMC6	structural maintenance of chromosomes 6
1.21	PSG5	pregnancy specific beta-1-glycoprotein 5
1.21	GRM8	glutamate receptor. metabotropic 8
1.21	MATN3	matrilin 3
1.21	SGCG	sarcoglycan gamma
1.21	GRSF1	G-rich RNA sequence binding factor 1
1.21	MARCH6	membrane associated ring finger 6
1.21	MED15	mediator complex subunit 15
1.21	DUS1L	dihydrouridine synthase 1-like
1.21	KAT6B	K(lysine) acetyltransferase 6B
1.21	CUEDC2	CUE domain containing 2

1.21	COLQ	collagen-like tail subunit (single strand of homotrimer) of asymmetric acetylcholinesterase
1.21	EGLN3	egl-9 family hypoxia-inducible factor 3
1.21	RNF44	ring finger protein 44
1.21	SLMAP	sarcolemma associated protein
1.21	RB1CC1	RB1-inducible coiled-coil 1
1.21	C6orf52	chromosome 6 open reading frame 52
1.21	QTRTD1	queuine tRNA-ribosyltransferase domain containing 1
1.21	OVCH1	ovochymase 1
1.21	RDH10-AS1	RDH10 antisense RNA 1
1.21	ANXA7	annexin A7
1.21	PADI4	peptidyl arginine deiminase. type IV
1.21	SPRY2	sprouty RTK signaling antagonist 2
1.21	TBX3	T-box 3
1.21	ACTG1	actin gamma 1
1.21	NR1H4	nuclear receptor subfamily 1. group H. member 4
1.21	ADAM7	ADAM metallopeptidase domain 7
1.21	LINC01537	long intergenic non-protein coding RNA 1537
1.21	MCAM; MIR6756	melanoma cell adhesion molecule; microRNA 6756
1.21	EIF2B2	eukaryotic translation initiation factor 2B. subunit 2 beta. 39kDa
1.21	QTRT1	queuine tRNA-ribosyltransferase 1
1.21	DUX4	double homeobox 4
1.21	C2orf81	chromosome 2 open reading frame 81
1.21	GBP3	guanylate binding protein 3
1.21	ZNF780A	zinc finger protein 780A
1.21	RPSA; SNORA62; SNORA6	ribosomal protein SA; small nucleolar RNA. H/ACA box 62; small nucleolar RNA. H/ACA box 6

1.21	NME6	NME/NM23 nucleoside diphosphate kinase 6
1.21	LRP6	LDL receptor related protein 6
1.21	PADI4	peptidyl arginine deiminase. type IV
1.21	ABCA8	ATP binding cassette subfamily A member 8
1.21	MAATS1	MYCBP-associated. testis expressed 1
1.21	APMAP	adipocyte plasma membrane associated protein
1.21	ENTPD8	ectonucleoside triphosphate diphosphohydrolase 8
1.21	RPS29; RPL32P29	ribosomal protein S29; ribosomal protein L32 pseudogene 29
1.21	HIVEP2	Jeck2013 ALT_ACCEPTOR. ALT_DONOR. coding. INTERNAL. intronic best transcript NM_006734
1.21	NPL	N-acetylneuraminate pyruvate lyase (dihydrodipicolinate synthase)
1.21	RASGEF1B	RasGEF domain family member 1B
1.21	ATP13A2	ATPase type 13A2
1.21	ACSS3	acyl-CoA synthetase short-chain family member 3
1.21	FAM131C	family with sequence similarity 131. member C
1.21	LINC01296; DUXAP10	long intergenic non-protein coding RNA 1296; double homeobox A pseudogene 10
1.21	ARMC3	armadillo repeat containing 3
1.21	NAT10	N-acetyltransferase 10 (GCN5-related)
1.21	CT47A1	cancer/testis antigen family 47. member A1
1.21	GSDMB	gasdermin B
1.21	HLA-DMB	major histocompatibility complex. class II. DM beta
1.21	ANKRD13C	ankyrin repeat domain 13C
1.21	HAND2-AS1	HAND2 antisense RNA 1 (head to head)
1.21	SOX8	SRY box 8
1.21	UCK2	Jeck2013 ALT_ACCEPTOR. ALT_DONOR. coding. INTERNAL. intronic best transcript NM_012474

1.21	FBXO32	F-box protein 32
1.21	MTUS1	microtubule associated tumor suppressor 1
1.21	BICD2	Memczak2013 ALT_ACCEPTOR. ALT_DONOR. coding. INTERNAL. intronic best transcript NM_001003800
1.21	ANKRD50	ankyrin repeat domain 50
1.21	HRH1	histamine receptor H1
1.21	PDE4C	phosphodiesterase 4C. cAMP-specific
1.21	ANLN	Transcript Identified by AceView. Entrez Gene ID(s) 54443
1.21	EIF3H	eukaryotic translation initiation factor 3. subunit H
1.21	ZBBX	zinc finger. B-box domain containing
1.21	MAP3K6	mitogen-activated protein kinase kinase kinase 6
1.21	EFHC2	EF-hand domain (C-terminal) containing 2
1.21	ZNF546	zinc finger protein 546
1.21	TRAP1	TNF receptor-associated protein 1
1.21	FAM19A2	family with sequence similarity 19 (chemokine (C-C motif)-like). member A2
1.21	EBI3	Epstein-Barr virus induced 3
1.21	GAGE12I; GAGE12F	G antigen 12I; G antigen 12F
1.21	VPS36	vacuolar protein sorting 36 homolog (S. cerevisiae)
1.21	F2R	coagulation factor II (thrombin) receptor
1.21	COX20	COX20 cytochrome c oxidase assembly factor
1.21	LSM10	LSM10. U7 small nuclear RNA associated
1.21	S100PBP	S100P binding protein
1.21	RCC2	regulator of chromosome condensation 2
1.21	PLA2G4F	phospholipase A2. group IVF
1.21	MS4A6A	membrane-spanning 4-domains. subfamily A. member 6A
1.21	C7orf71	chromosome 7 open reading frame 71

1.21	HIVEP1	human immunodeficiency virus type I enhancer binding protein 1
1.21	METTL16	methyltransferase like 16
1.21	DHX15	DEAH (Asp-Glu-Ala-His) box helicase 15
1.21	MPP4	membrane protein. palmitoylated 4
1.21	MTRR	5-methyltetrahydrofolate-homocysteine methyltransferase reductase
1.21	DPP8	dipeptidyl-peptidase 8
1.21	NAPSA	napsin A aspartic peptidase
1.21	NUMBL	numb homolog (Drosophila)-like
1.21	SF3B1	Memczak2013 ANTISENSE. CDS. coding. INTERNAL best transcript NM_012433
1.21	ZNF554	zinc finger protein 554
1.21	ANKRD31	ankyrin repeat domain 31
1.21	HS6ST2	heparan sulfate 6-O-sulfotransferase 2
1.21	PCSK2	Transcript Identified by AceView. Entrez Gene ID(s) 5126
1.21	SENP3-EIF4A1	SENP3-EIF4A1 readthrough (NMD candidate)
1.21	MSN	moesin
1.21	SIAH1	siah E3 ubiquitin protein ligase 1
1.21	GOLIM4	golgi integral membrane protein 4
1.21	TOP1MT	topoisomerase (DNA) I. mitochondrial
1.21	UBQLN3	ubiquilin 3
1.21	CHMP1B	charged multivesicular body protein 1B
1.21	RTCA	RNA 3-terminal phosphate cyclase
1.21	CACNG1	calcium channel. voltage-dependent. gamma subunit 1
1.21	UBE2F-SCLY	UBE2F-SCLY readthrough (NMD candidate)
1.21	BSPH1	binder of sperm protein homolog 1
1.21	TTC31	Memczak2013 ALT_ACCEPTOR. ALT_DONOR. coding. INTERNAL. intronic best transcript NM_022492

1.21	SLC29A1	solute carrier family 29 (equilibrative nucleoside transporter). member 1
1.21	NCKAP5	NCK-associated protein 5
1.21	MAPK8	mitogen-activated protein kinase 8
1.21	OR7A10	olfactory receptor. family 7. subfamily A. member 10
1.21	RFPL4A	ret finger protein-like 4A
1.21	METTL10	methyltransferase like 10
1.21	MAN2A2	mannosidase. alpha. class 2A. member 2
1.21	GPRC5C	G protein-coupled receptor. class C. group 5. member C
1.21	ABCB4	ATP binding cassette subfamily B member 4
1.21	CLDN10	claudin 10
1.21	CYC1	cytochrome c-1
1.21	TMEM179	transmembrane protein 179
1.21	TMEM18	transmembrane protein 18
1.21	KIF2C	Jeck2013 ALT_DONOR. coding. INTERNAL. intronic best transcript NM_006845
1.21	SH3D21	SH3 domain containing 21
1.21	SSUH2	ssu-2 homolog (C. elegans)
1.21	PRMT3	protein arginine methyltransferase 3
1.21	ARHGAP29	Rho GTPase activating protein 29
1.21	FRMD7	FERM domain containing 7
1.21	PARP4	poly(ADP-ribose) polymerase family member 4
1.21	LMBR1	Transcript Identified by AceView. Entrez Gene ID(s) 64327
1.21	TRPC4	transient receptor potential cation channel. subfamily C. member 4
1.21	HCRTR2	hypocretin (orexin) receptor 2
1.21	S100A14	S100 calcium binding protein A14
1.21	DEDD	death effector domain containing

1.21	LDLRAP1	low density lipoprotein receptor adaptor protein 1
1.21	ZNF366	zinc finger protein 366
1.21	IGFLR1	IGF like family receptor 1
1.21	CHD1	chromodomain helicase DNA binding protein 1
1.21	LIMD1	LIM domains containing 1
1.21	LRRC37A4P	leucine rich repeat containing 37. member A4. pseudogene
1.21	TMEM38A	transmembrane protein 38A
1.21	RRNAD1	ribosomal RNA adenine dimethylase domain containing 1
1.21	LRPPRC	leucine-rich pentatricopeptide repeat containing
1.21	CLYBL	citrate lyase beta like
1.20	PLEC	plectin
1.20	SOGA1	suppressor of glucose. autophagy associated 1
1.20	KCNJ2	potassium channel. inwardly rectifying subfamily J. member 2
1.20	GABBR2	gamma-aminobutyric acid (GABA) B receptor. 2
1.20	ZNF616	zinc finger protein 616
1.20	TRNT1	tRNA nucleotidyl transferase. CCA-adding. 1
1.20	ITGA2B	integrin alpha 2b
1.20	RIMBP3	RIMS binding protein 3
1.20	SNX27	sorting nexin family member 27
1.20	PPP6R3	protein phosphatase 6. regulatory subunit 3
1.20	RPL17-C18orf32	RPL17-C18orf32 readthrough
1.20	PATE3	prostate and testis expressed 3
1.20	SACS	sacsin molecular chaperone
1.20	GAL3ST4	galactose-3-O-sulfotransferase 4
1.20	SURF2	surfeit 2 [Source:HGNC Symbol;Acc:HGNC:11475]
1.20	P2RX7	purinergic receptor P2X. ligand gated ion channel. 7

1.20	FGF18	fibroblast growth factor 18
1.20	PHC2; MIR3605	polyhomeotic homolog 2 (Drosophila); microRNA 3605
1.20	C1orf216	chromosome 1 open reading frame 216
1.20	DR1	down-regulator of transcription 1
1.20	FAM131A	family with sequence similarity 131. member A
1.20	DUS3L	Memczak2013 ANTISENSE. CDS. coding. INTERNAL best transcript NM_020175
1.20	CACNA2D2	calcium channel. voltage-dependent. alpha 2/delta subunit 2
1.20	ASPA	aspartoacylase
1.20	POLR3B	polymerase (RNA) III (DNA directed) polypeptide B
1.20	RPUSD4	RNA pseudouridylate synthase domain containing 4
1.20	BPGM	Transcript Identified by AceView. Entrez Gene ID(s) 669
1.20	TSR2	TSR2. 20S rRNA accumulation. homolog (S. cerevisiae)
1.20	FAIM	Fas apoptotic inhibitory molecule
1.20	C9orf43	chromosome 9 open reading frame 43
1.20	ESRP1	epithelial splicing regulatory protein 1
1.20	KRTAP12-4	keratin associated protein 12-4
1.20	NSMAF	neutral sphingomyelinase activation associated factor
1.20	SHOX	short stature homeobox
1.20	HBD	hemoglobin. delta
1.20	TAF1B	TATA box binding protein (TBP)-associated factor. RNA polymerase I. B. 63kDa
1.20	LYZL1	lysozyme-like 1
1.20	IPPK	inositol 1.3.4.5.6-pentakisphosphate 2-kinase
1.20	VPS11	VPS11. CORVET/HOPS core subunit [Source:HGNC Symbol;Acc:HGNC:14583]
1.20	IL1RAPL2	interleukin 1 receptor accessory protein-like 2
1.20	SFMBT2	Scm-like with four mbt domains 2

1.20	CEACAM19	carcinoembryonic antigen-related cell adhesion molecule 19
1.20	BRIX1	BRX1. biogenesis of ribosomes
1.20	RALYL	RALY RNA binding protein-like
1.20	ALG13	ALG13. UDP-N-acetylglucosaminyltransferase subunit
1.20	FMN1	formin 1
1.20	DEFA6	defensin. alpha 6. Paneth cell-specific
1.20	C6orf136	chromosome 6 open reading frame 136
1.20	GNRHR	gonadotropin releasing hormone receptor
1.20	CEP95	centrosomal protein 95kDa
1.20	EIF3A	eukaryotic translation initiation factor 3. subunit A
1.20	LHX4-AS1; ACBD6	LHX4 antisense RNA 1; acyl-CoA binding domain containing 6
1.20	KCNJ5	potassium channel. inwardly rectifying subfamily J. member 5
1.20	PTRF	polymerase I and transcript release factor
1.20	SMAD7	Memczak2013 ALT_ACCEPTOR. ALT_DONOR. coding. INTERNAL. intronic best transcript NM_001190821
1.20	RASSF2	Memczak2013 ANTISENSE. coding. INTERNAL. UTR3 best transcript NM_014737
1.20	FAM151B	Memczak2013 ANTISENSE. coding. INTERNAL. intronic best transcript NM_205548
1.20	BNC1	basonuclin 1
1.20	DCAF12L1	DDB1 and CUL4 associated factor 12-like 1
1.20	CACFD1	calcium channel flower domain containing 1
1.20	GNAQ; skeyplorbu	Memczak2013 ANTISENSE. coding. INTERNAL. intronic best transcript NM_002072; Transcript Identified by AceView
1.20	OMP	olfactory marker protein
1.20	OR5G5P; OR5G3	olfactory receptor. family 5. subfamily G. member 5 pseudogene; olfactory receptor. family 5. subfamily G. member 3 (gene/pseudogene)
1.20	PABPC3	poly(A) binding protein. cytoplasmic 3

1.20	LONRF1	LON peptidase N-terminal domain and ring finger 1
1.20	KDM6B	lysine (K)-specific demethylase 6B
1.20	TMEM2	Transcript Identified by AceView. Entrez Gene ID(s) 23670
1.20	MIPEP	mitochondrial intermediate peptidase
1.20	ZNF415	zinc finger protein 415
1.20	MYADM	myeloid-associated differentiation marker
1.20	MYLK	myosin light chain kinase
1.20	OR4X2	olfactory receptor. family 4. subfamily X. member 2 (gene/pseudogene)
1.20	KIAA1715	KIAA1715
1.20	DDX5; MIR3064; MIR5047	DEAD (Asp-Glu-Ala-Asp) box helicase 5; microRNA 3064; microRNA 5047
1.20	KIAA1033	Memczak2013 ALT_ACCEPTOR. ALT_DONOR. coding. INTERNAL. intronic best transcript NM_015275
1.20	C1GALT1C1	C1GALT1 specific chaperone 1
1.20	RILPL1	Rab interacting lysosomal protein-like 1
1.20	E2F5	E2F transcription factor 5. p130-binding
1.20	MRPL10	mitochondrial ribosomal protein L10
1.20	HLA-DQA1	major histocompatibility complex. class II. DQ alpha 1
1.20	SEC24B	SEC24 homolog B. COPII coat complex component
1.20	SP6	Sp6 transcription factor
1.20	MRPL16	mitochondrial ribosomal protein L16
1.20	E2F3	E2F transcription factor 3
1.20	KPNA1	Transcript Identified by AceView. Entrez Gene ID(s) 3836
1.20	FAM78A	family with sequence similarity 78. member A
1.20	ZNF597	zinc finger protein 597
1.20	ARID3B	AT rich interactive domain 3B (BRIGHT-like)
1.20	MRC1	mannose receptor. C type 1

1.20	MSH3	mutS homolog 3
1.20	CFAP161	cilia and flagella associated protein 161
1.20	MYH1	myosin. heavy chain 1. skeletal muscle. adult
1.20	USP3	ubiquitin specific peptidase 3
1.20	NSA2	NSA2 ribosome biogenesis homolog
1.20	NRG4	neuregulin 4
1.20	PTH2	parathyroid hormone 2
1.20	SLC39A8	solute carrier family 39 (zinc transporter). member 8
1.20	RNF39	ring finger protein 39
1.20	BTBD11	BTB (POZ) domain containing 11
1.20	BCOR	BCL6 corepressor
1.20	SRP72	signal recognition particle 72kDa
1.20	NECAP1	NECAP endocytosis associated 1
1.20	MXD1	Memczak2013 ANTISENSE. coding. INTERNAL. intronic best transcript NM_001202514
1.20	BBS7	Bardet-Biedl syndrome 7
1.20	HVCN1	hydrogen voltage gated channel 1
1.20	MAP1LC3B	microtubule-associated protein 1 light chain 3 beta
1.20	XAB2	XPA binding protein 2
1.20	SLC25A6	solute carrier family 25 (mitochondrial carrier; adenine nucleotide translocator). member 6
1.20	MCPH1	microcephalin 1
1.20	GNPTAB	N-acetylglucosamine-1-phosphate transferase. alpha and beta subunits
1.20	MYOM1	myomesin 1
1.20	HBB	hemoglobin. beta
1.20	BFSP1	beaded filament structural protein 1. filensin
1.20	TYW3	tRNA-yW synthesizing protein 3 homolog (S. cerevisiae)

1.20	ARHGEF4	Rho guanine nucleotide exchange factor 4
1.20	SHC1	SHC (Src homology 2 domain containing) transforming protein 1
1.20	SLC16A1	solute carrier family 16 (monocarboxylate transporter). member 1
1.20	NRXN1	neurexin 1
1.20	XCL2	chemokine (C motif) ligand 2
1.20	MIPOL1	mirror-image polydactyly 1
1.20	SGCD	sarcoglycan delta
1.20	SLC9C2	solute carrier family 9. member C2 (putative)
1.20	MSL1	male-specific lethal 1 homolog (Drosophila)
1.20	RASA2	RAS p21 protein activator 2
1.20	BGN	biglycan
1.20	CHAC1	ChaC glutathione-specific gamma-glutamylcyclotransferase 1
1.20	KIRREL3	Jeck2013 ALT_ACCEPTOR. ALT_DONOR. coding. INTERNAL. intronic best transcript NM_032531
1.20	DYNLL1	dynein. light chain. LC8-type 1
1.20	ACSM6	acyl-CoA synthetase medium-chain family member 6
1.20	ACTL6A	actin-like 6A
1.20	SLC46A3	solute carrier family 46. member 3
1.20	RNF113A	ring finger protein 113A
1.20	KIF15	kinesin family member 15
1.20	PCSK5	proprotein convertase subtilisin/kexin type 5
1.20	DNAJA2	DnaJ (Hsp40) homolog. subfamily A. member 2
1.20	S100A7	S100 calcium binding protein A7
1.20	RPS4Y1	ribosomal protein S4. Y-linked 1
1.20	DKKL1	dickkopf-like 1
1.20	MINK1	misshapen-like kinase 1

1.20	N4BP2L2	NEDD4 binding protein 2-like 2
1.20	SPATA3	spermatogenesis associated 3
1.20	TM2D1	TM2 domain containing 1
1.20	LMOD1	leiomodin 1 (smooth muscle)
1.20	RNF214	ring finger protein 214
1.20	HAMP	hepcidin antimicrobial peptide
1.20	CYFIP1	cytoplasmic FMR1 interacting protein 1
1.20	NTHL1	nth-like DNA glycosylase 1
1.20	PRKCE	protein kinase C. epsilon
1.20	RPS18	ribosomal protein S18
1.20	PLA2G16	phospholipase A2. group XVI
1.20	OR2M2	olfactory receptor. family 2. subfamily M. member 2
1.20	KDM1B	lysine (K)-specific demethylase 1B
1.20	RSAD2	radical S-adenosyl methionine domain containing 2
1.20	UBFD1	ubiquitin family domain containing 1
1.20	RIPK3	receptor-interacting serine-threonine kinase 3
1.20	EXOSC10	exosome component 10
1.20	S100A7A	S100 calcium binding protein A7A
1.20	BAMBI	BMP and activin membrane-bound inhibitor
1.20	ZMIZ1	Zhang2013 ALT_ACCEPTOR. ALT_DONOR. coding. INTERNAL. intronic best transcript NM_020338
1.20	NR2C1	nuclear receptor subfamily 2. group C. member 1
1.20	GAS8	growth arrest-specific 8
1.20	FOXQ1	forkhead box Q1
1.20	TSN	translin
1.20	RMDN3	regulator of microtubule dynamics 3

1.20	NAT8L	N-acetyltransferase 8-like (GCN5-related. putative)
1.20	RNF14	ring finger protein 14
1.20	ZNF225	zinc finger protein 225
1.20	STK33	serine/threonine kinase 33
1.20	SBF2	SET binding factor 2
1.19	SPAG6	sperm associated antigen 6
1.19	GSPT2	G1 to S phase transition 2
1.19	BTRC	beta-transducin repeat containing E3 ubiquitin protein ligase
1.19	RCC2	regulator of chromosome condensation 2
1.19	RPS3; SNORD15A	ribosomal protein S3; small nucleolar RNA. C/D box 15A
1.19	WDR26; MIR4742	WD repeat domain 26; microRNA 4742
1.19	ERC1	ELKS/RAB6-interacting/CAST family member 1
1.19	GAGE1	G antigen 1
1.19	SAG	S-antigen; retina and pineal gland (arrestin)
1.19	MYO15B	myosin XVB
1.19	KRT77	keratin 77. type II
1.19	TMEM126B	transmembrane protein 126B
1.19	LAX1	lymphocyte transmembrane adaptor 1
1.19	NRIP3	nuclear receptor interacting protein 3
1.19	CA8	carbonic anhydrase VIII
1.19	HCN1	hyperpolarization activated cyclic nucleotide gated potassium channel 1
1.19	FBN3; mumari	Zhang2013 ALT_ACCEPTOR. ALT_DONOR. coding. INTERNAL. intronic best transcript NM_032447; Transcript Identified by AceView
1.19	MPLKIP	M-phase specific PLK1 interacting protein
1.19	TEX26	testis expressed 26
1.19	DBNDD1	dysbindin (dystrobrevin binding protein 1) domain containing 1

1.19	ZNF839	zinc finger protein 839
1.19	B3GALT1	UDP-Gal:betaGlcNAc beta 1.3-galactosyltransferase 1
1.19	IFITM1	interferon induced transmembrane protein 1
1.19	CRCT1	cysteine rich C-terminal 1
1.19	WDR74	WD repeat domain 74
1.19	OTUD6B	Transcript Identified by AceView. Entrez Gene ID(s) 51633
1.19	MCOLN3	mucolipin 3
1.19	SURF1	surfeit 1
1.19	S1PR2	sphingosine-1-phosphate receptor 2
1.19	INTS12	integrator complex subunit 12
1.19	DTX3	deltex 3. E3 ubiquitin ligase
1.19	SNU13	SNU13 homolog. small nuclear ribonucleoprotein (U4/U6.U5)
1.19	MSH4	mutS homolog 4
1.19	NAPG	N-ethylmaleimide-sensitive factor attachment protein. gamma
1.19	MS4A2	membrane-spanning 4-domains. subfamily A. member 2
1.19	ADAMTS12	ADAM metallopeptidase with thrombospondin type 1 motif 12
1.19	AVL9	AVL9 homolog (S. cerevisiase)
1.19	C11orf58	chromosome 11 open reading frame 58
1.19	C11orf72	chromosome 11 open reading frame 72
1.19	LINC00982	long intergenic non-protein coding RNA 982
1.19	TLR3	toll-like receptor 3
1.19	ACTR8	ARP8 actin-related protein 8 homolog (yeast)
1.19	PTPN21	protein tyrosine phosphatase. non-receptor type 21
1.19	SERPING1	serpin peptidase inhibitor. clade G (C1 inhibitor). member 1
1.19	USO1	Transcript Identified by AceView. Entrez Gene ID(s) 8615
1.19	OR13J1	olfactory receptor. family 13. subfamily J. member 1

1.19	PXDN	peroxidasin
1.19	SHC2	SHC (Src homology 2 domain containing) transforming protein 2
1.19	GPR78	G protein-coupled receptor 78
1.19	TEKT2	tektin 2 (testicular)
1.19	WRNIP1	Werner helicase interacting protein 1
1.19	PMPCB	peptidase (mitochondrial processing) beta
1.19	CLNS1A	chloride channel. nucleotide-sensitive. 1A
1.19	MAP3K15	mitogen-activated protein kinase kinase kinase 15
1.19	METTL2A	methyltransferase like 2A
1.19	ACRV1	acrosomal vesicle protein 1
1.19	CDK7	cyclin-dependent kinase 7
1.19	TMEM126A	transmembrane protein 126A
1.19	GTF2H1	general transcription factor IIH subunit 1
1.19	GPBP1L1	GC-rich promoter binding protein 1-like 1
1.19	SH2D7	SH2 domain containing 7
1.19	RBFOX2	RNA binding protein. fox-1 homolog (C. elegans) 2
1.19	POMT2	protein-O-mannosyltransferase 2
1.19	TTC17	tetratricopeptide repeat domain 17
1.19	NF1	neurofibromin 1
1.19	INTS2	integrator complex subunit 2
1.19	LRRC10B	leucine rich repeat containing 10B
1.19	B3GALNT2	beta-1.3-N-acetylgalactosaminyltransferase 2
1.19	WDR11	WD repeat domain 11
1.19	USP34	Memczak2013 ANTISENSE. CDS. coding. INTERNAL best transcript NM_014709
1.19	FOXO1	Memczak2013 ALT_ACCEPTOR. ALT_DONOR. coding. INTERNAL. intronic best transcript NM_002015

1.19	MAT2A	methionine adenosyltransferase II. alpha
1.19	ADAP1	ArfGAP with dual PH domains 1
1.19	ADGRB3	adhesion G protein-coupled receptor B3
1.19	MRPL13	mitochondrial ribosomal protein L13
1.19	WDR82	WD repeat domain 82
1.19	FAM96A	family with sequence similarity 96. member A
1.19	LCE1D	late cornified envelope 1D
1.19	LCORL	Transcript Identified by AceView. Entrez Gene ID(s) 254251
1.19	ICA1L	islet cell autoantigen 1 like
1.19	SH3BP4	SH3-domain binding protein 4
1.19	FBLIM1	filamin binding LIM protein 1
1.19	EPHA3	EPH receptor A3
1.19	BIRC3	baculoviral IAP repeat containing 3
1.19	UBE2E3	ubiquitin-conjugating enzyme E2E 3
1.19	ADTRP	androgen-dependent TFPI-regulating protein
1.19	OR5B2	olfactory receptor. family 5. subfamily B. member 2
1.19	DZIP1	Transcript Identified by AceView. Entrez Gene ID(s) 22873
1.19	GOLGA6C	golgin A6 family. member C
1.19	PPP4R3A	protein phosphatase 4. regulatory subunit 3A
1.19	BEND5	BEN domain containing 5
1.19	XRN1	5-3 exoribonuclease 1
1.19	UGDH	UDP-glucose 6-dehydrogenase
1.19	RNF6	ring finger protein (C3H2C3 type) 6
1.19	REEP5	receptor accessory protein 5
1.19	HAPLN4	hyaluronan and proteoglycan link protein 4
1.19	ABCB10	ATP binding cassette subfamily B member 10

1.19	SMN1; SMN2	survival of motor neuron 1. telomeric; survival of motor neuron 2. centromeric
1.19	SLC25A37	solute carrier family 25 (mitochondrial iron transporter). member 37
1.19	EMG1	EMG1 N1-specific pseudouridine methyltransferase
1.19	SRA1	steroid receptor RNA activator 1
1.19	F8A2	coagulation factor VIII-associated 2
1.19	WDR31	WD repeat domain 31
1.19	RALGDS	ral guanine nucleotide dissociation stimulator
1.19	CPSF4	cleavage and polyadenylation specific factor 4
1.19	GPRIN3	GPRIN family member 3
1.19	GSKIP	GSK3B interacting protein
1.19	LINC00266-1	long intergenic non-protein coding RNA 266-1
1.19	RNF10	ring finger protein 10
1.19	MFSD14B	major facilitator superfamily domain containing 14B
1.19	SEMA5B	sema domain. seven thrombospondin repeats (type 1 and type 1-like). transmembrane domain (TM) and short cytoplasmic domain. (semaphorin) 5B
1.19	FAM195A	family with sequence similarity 195. member A
1.19	NXPE4	neurexophilin and PC-esterase domain family. member 4
1.19	MAFG	v-maf avian musculoaponeurotic fibrosarcoma oncogene homolog G
1.19	ZNF665	zinc finger protein 665
1.19	MYEOV	myeloma overexpressed
1.19	LYZL2	lysozyme-like 2
1.19	NACC2	NACC family member 2. BEN and BTB (POZ) domain containing
1.19	CNTNAP3B	Salzman2013 ANNOTATED. CDS. coding. INTERNAL. OVCODE. OVEXON best transcript NM_001201380
1.19	TUBGCP3	tubulin. gamma complex associated protein 3

1.19	SRXN1	sulfiredoxin 1
1.19	SNRPD1	small nuclear ribonucleoprotein D1 polypeptide
1.19	EXOC1	exocyst complex component 1
1.19	MANSC1	MANSC domain containing 1
1.19	REEP3	receptor accessory protein 3
1.19	IGF2	insulin-like growth factor 2
1.19	SGMS1	sphingomyelin synthase 1
1.19	LOC646938	TBC1 domain family. member 2B pseudogene; Transcript Identified by AceView. Entrez Gene ID(s) 646938
1.19	GJA10	gap junction protein alpha 10
1.19	PTRH2	peptidyl-tRNA hydrolase 2
1.19	MAP3K8	mitogen-activated protein kinase kinase kinase 8
1.19	MAPRE3	microtubule-associated protein. RP/EB family. member 3
1.19	XPO5	exportin 5
1.19	CLEC1A	C-type lectin domain family 1. member A
1.19	FAS	Fas cell surface death receptor
1.19	TATDN3	TatD DNase domain containing 3
1.19	FOXC2	forkhead box C2
1.19	MED23	mediator complex subunit 23
1.19	RP11-380G5.2; PTEN	Jeck2013 ALT_ACCEPTOR. ALT_DONOR. coding. INTERNAL. intronic best transcript NM_000314; novel transcript. sense intronic to PTEN
1.19	DEFB4A	defensin. beta 4A
1.19	KCND1	potassium channel. voltage gated Shal related subfamily D. member 1
1.19	TBC1D29	TBC1 domain family. member 29
1.19	STARD6	StAR-related lipid transfer domain containing 6
1.19	TMEM68	transmembrane protein 68
1.19	AGER	advanced glycosylation end product-specific receptor

1.19	APBB1	amyloid beta (A4) precursor protein-binding. family B. member 1 (Fe65)
1.19	IRF2BP2	interferon regulatory factor 2 binding protein 2
1.19	CD38	CD38 molecule
1.19	KCNE3	potassium channel. voltage gated subfamily E regulatory beta subunit 3
1.19	TMC5	transmembrane channel like 5
1.19	RSRC1	arginine/serine-rich coiled-coil 1
1.19	USP26	ubiquitin specific peptidase 26
1.19	MAP3K4	mitogen-activated protein kinase kinase kinase 4
1.19	KPNA1	karyopherin alpha 1
1.19	CAV2	caveolin 2
1.19	ERP44	endoplasmic reticulum protein 44
1.19	ZNF558	zinc finger protein 558
1.19	OR3A4P; OR3A5P	olfactory receptor. family 3. subfamily A. member 4 pseudogene; olfactory receptor. family 3. subfamily A. member 5 pseudogene
1.19	C3orf58	chromosome 3 open reading frame 58
1.19	HAND1	heart and neural crest derivatives expressed 1
1.19	RIN2	Ras and Rab interactor 2
1.19	SERPINB9	serpin peptidase inhibitor. clade B (ovalbumin). member 9
1.19	GNG8	guanine nucleotide binding protein (G protein). gamma 8
1.19	OR5M10	olfactory receptor. family 5. subfamily M. member 10
1.19	EFNB1	ephrin-B1
1.19	ABCB1	ATP binding cassette subfamily B member 1
1.19	RNF186	ring finger protein 186
1.19	OR1N1	olfactory receptor. family 1. subfamily N. member 1
1.19	PRKCD	protein kinase C. delta
1.19	EMC2	ER membrane protein complex subunit 2

1.19	CD96	CD96 molecule
1.19	TMEM67	transmembrane protein 67
1.19	OR5W2	olfactory receptor. family 5. subfamily W. member 2
1.19	CD2AP	CD2-associated protein
1.19	STRAP	serine/threonine kinase receptor associated protein
1.19	DEFB127	defensin. beta 127
1.19	OXCT1	Transcript Identified by AceView. Entrez Gene ID(s) 5019
1.19	APEX1	APEX nuclease (multifunctional DNA repair enzyme) 1
1.19	NKAIN3	Jeck2013 ANTISENSE. coding. INTERNAL. intronic best transcript NM_173688
1.19	WDR46; MIR6873	WD repeat domain 46; microRNA 6873
1.19	S100Z	S100 calcium binding protein Z
1.19	ANO1	anoctamin 1. calcium activated chloride channel
1.19	ICAM1	intercellular adhesion molecule 1
1.19	FAM25A	family with sequence similarity 25. member A
1.19	NMRK2	nicotinamide riboside kinase 2
1.19	C5orf58	chromosome 5 open reading frame 58
1.19	TWISTNB	TWIST neighbor
1.19	RGPD2; RGPD1	RANBP2-like and GRIP domain containing 2; RANBP2-like and GRIP domain containing 1
1.19	PSMG1	proteasome (prosome. macropain) assembly chaperone 1
1.19	ARSB	arylsulfatase B
1.19	EIF3L	eukaryotic translation initiation factor 3. subunit L
1.19	MEIOB; LINC00254	meiosis specific with OB domains; long intergenic non-protein coding RNA 254
1.19	IL1RN	interleukin 1 receptor antagonist
1.19	GPC5	glypican 5
1.19	CFAP52	cilia and flagella associated protein 52

1.19	ASXL3	additional sex combs like transcriptional regulator 3
1.19	ago-04	argonaute RISC catalytic component 4
1.19	FAM63B	family with sequence similarity 63. member B
1.18	ACER3	alkaline ceramidase 3
1.18	TNPO3	transportin 3
1.18	OR2A5	olfactory receptor. family 2. subfamily A. member 5
1.18	PCGF6	polycomb group ring finger 6
1.18	NUBPL	nucleotide binding protein-like
1.18	RAC1	ras-related C3 botulinum toxin substrate 1 (rho family. small GTP binding protein Rac1)
1.18	SLC12A8	solute carrier family 12. member 8
1.18	LCE2A	late cornified envelope 2A
1.18	UGGT2	UDP-glucose glycoprotein glucosyltransferase 2
1.18	CGRRF1	cell growth regulator with ring finger domain 1
1.18	ERBB4	Transcript Identified by AceView. Entrez Gene ID(s) 2066
1.18	UNC119	unc-119 lipid binding chaperone
1.18	SIX4	SIX homeobox 4
1.18	PTPRZ1	protein tyrosine phosphatase. receptor-type. Z polypeptide 1
1.18	WNT5A	wingless-type MMTV integration site family. member 5A
1.18	RCC2	regulator of chromosome condensation 2
1.18	CD3D	CD3d molecule. delta (CD3-TCR complex)
1.18	ARL4C	ADP-ribosylation factor like GTPase 4C
1.18	B4GALT7	xylosylprotein beta 1.4-galactosyltransferase. polypeptide 7
1.18	APBA1	amyloid beta (A4) precursor protein-binding. family A. member 1
1.18	ANXA5	annexin A5
1.18	TMEM159	transmembrane protein 159

1.18	PROKR1	prokineticin receptor 1
1.18	ATPAF1	ATP synthase mitochondrial F1 complex assembly factor 1
1.18	NAV3	neuron navigator 3
1.18	ASUN	asunder spermatogenesis regulator
1.18	LUM	lumican
1.18	TSPAN14	tetraspanin 14
1.18	WFDC9	WAP four-disulfide core domain 9
1.18	ENTHD1	ENTH domain containing 1
1.18	PREP	prolyl endopeptidase
1.18	MAP7	microtubule-associated protein 7
1.18	KALRN	Transcript Identified by AceView. Entrez Gene ID(s) 8997
1.18	CEBPE	CCAAT/enhancer binding protein (C/EBP). epsilon
1.18	C8orf33	chromosome 8 open reading frame 33
1.18	KRTAP19-4	keratin associated protein 19-4
1.18	ITPR3	Zhang2013 ALT_ACCEPTOR. ALT_DONOR. coding. INTERNAL. intronic best transcript NM_002224
1.18	RENBP	renin binding protein
1.18	DUSP14	dual specificity phosphatase 14
1.18	LAMC1	laminin. gamma 1 (formerly LAMB2)
1.18	PTPRO	protein tyrosine phosphatase. receptor type. O
1.18	R3HDM4	R3H domain containing 4
1.18	DNAJC25-GNG10	DNAJC25-GNG10 readthrough
1.18	RPL35A	ribosomal protein L35a
1.18	RASD2	Memczak2013 ANTISENSE. coding. INTERNAL. UTR3 best transcript NM_014310
1.18	ARPC1A	actin related protein 2/3 complex subunit 1A
1.18	GNG7	Transcript Identified by AceView. Entrez Gene ID(s) 2788

1.18	RLIM	ring finger protein. LIM domain interacting
1.18	SLC44A1	solute carrier family 44 (choline transporter). member 1
1.18	YWHAEP7	tyrosine 3-monooxygenase/tryptophan 5-monooxygenase activation protein. epsilon pseudogene 7
1.18	SUZ12	SUZ12 polycomb repressive complex 2 subunit
1.18	TMEM168	transmembrane protein 168
1.18	SLC39A10	solute carrier family 39 (zinc transporter). member 10
1.18	NCLN	nicalin
1.18	FBL	fibrillarin
1.18	PTPN9	protein tyrosine phosphatase. non-receptor type 9
1.18	HBM	hemoglobin. mu
1.18	TMEM54	transmembrane protein 54
1.18	LCLAT1	lysocardiolipin acyltransferase 1
1.18	LYPD8	LY6/PLAUR domain containing 8
1.18	PRG3	proteoglycan 3
1.18	CFAP100	cilia and flagella associated protein 100
1.18	PTAFR	platelet-activating factor receptor
1.18	SEC14L2	SEC14-like lipid binding 2
1.18	CACNA1I	calcium channel. voltage-dependent. T type. alpha 1I subunit
1.18	KIAA1210	KIAA1210
1.18	C2CD5	C2 calcium-dependent domain containing 5
1.18	ZNF215	zinc finger protein 215
1.18	ZDHHC7	zinc finger. DHHC-type containing 7
1.18	FRMD1	FERM domain containing 1
1.18	OCLN	occludin
1.18	LRP6	LDL receptor related protein 6

1.18	EPS8	epidermal growth factor receptor pathway substrate 8
1.18	HMG20A	high mobility group 20A
1.18	SACM1L	SAC1 suppressor of actin mutations 1-like (yeast)
1.18	DTNB	dystrobrevin beta
1.18	GCKR	glucokinase (hexokinase 4) regulator
1.18	MRPL48	mitochondrial ribosomal protein L48
1.18	ZKSCAN8	zinc finger with KRAB and SCAN domains 8
1.18	MURC	muscle-related coiled-coil protein
1.18	TDRD1	tudor domain containing 1
1.18	ZER1	zyg-11 related. cell cycle regulator
1.18	CT45A6; CT45A7	cancer/testis antigen family 45. member A6; cancer/testis antigen family 45. member A7
1.18	TMEM71	transmembrane protein 71
1.18	MED13	mediator complex subunit 13
1.18	CYP39A1	cytochrome P450. family 39. subfamily A. polypeptide 1
1.18	TSKU	tsukushi. small leucine rich proteoglycan
1.18	EXOG	endo/exonuclease (5-3). endonuclease G-like
1.18	TGFBR3	transforming growth factor beta receptor III
1.18	TCTEX1D1	Tctex1 domain containing 1
1.18	DPRX	divergent-paired related homeobox
1.18	OR5M8	olfactory receptor. family 5. subfamily M. member 8
1.18	TRIM49B	tripartite motif containing 49B
1.18	KIF6	kinesin family member 6
1.18	ZNF518A	zinc finger protein 518A
1.18	ST6GAL2	ST6 beta-galactosamide alpha-2.6-sialyltranferase 2
1.18	ICE2	interactor of little elongation complex ELL subunit 2

1.18	PSG8	pregnancy specific beta-1-glycoprotein 8
1.18	F9	coagulation factor IX
1.18	SPRN	shadow of prion protein homolog (zebrafish)
1.18	VEZT	vezatin. adherens junctions transmembrane protein
1.18	KPNB1	karyopherin (importin) beta 1
1.18	ZNF777	zinc finger protein 777
1.18	DAOA	D-amino acid oxidase activator
1.18	TNPO1	transportin 1
1.18	TAS2R7	taste receptor. type 2. member 7
1.18	FUBP3	Transcript Identified by AceView. Entrez Gene ID(s) 8939
1.18	E2F6	E2F transcription factor 6
1.18	SPATA7	spermatogenesis associated 7
1.18	CD40LG	CD40 ligand
1.18	LCMT1	leucine carboxyl methyltransferase 1
1.18	FAM92A1	family with sequence similarity 92. member A1
1.18	LILRA1	leukocyte immunoglobulin-like receptor. subfamily A (with TM domain). member 1
1.18	PATL1	protein associated with topoisomerase II homolog 1 (yeast)
1.18	CD300LF	CD300 molecule-like family member f
1.18	LTV1	LTV1 ribosome biogenesis factor
1.18	DHX30	DEAH (Asp-Glu-Ala-His) box helicase 30
1.18	ANKRD61	ankyrin repeat domain 61
1.18	LCA5L	Leber congenital amaurosis 5-like
1.18	FLYWCH1	FLYWCH-type zinc finger 1
1.18	ITPA	inosine triphosphatase (nucleoside triphosphate pyrophosphatase)
1.18	RPS6KA1	ribosomal protein S6 kinase. 90kDa. polypeptide 1

1.18	NUDT17	nudix hydrolase 17
1.18	TPST1	tyrosylprotein sulfotransferase 1
1.18	TLR8	toll-like receptor 8
1.18	XPR1	xenotropic and polytropic retrovirus receptor 1
1.18	PSMD1	proteasome 26S subunit. non-ATPase 1
1.18	TRABD	Transcript Identified by AceView. Entrez Gene ID(s) 80305
1.18	THAP9	THAP domain containing 9
1.18	RDH12	retinol dehydrogenase 12 (all-trans/9-cis/11-cis)
1.18	DGCR6; LOC102724770	Homo sapiens DiGeorge syndrome critical region gene 6 (DGCR6). mRNA.; protein DGCR6; Homo sapiens DiGeorge syndrome critical region gene 6. mRNA (cDNA clone MGC:54086 IMAGE:5229172). complete cds.; Protein DGCR6 [Source:UniProtKB/Swiss-Prot;Acc:Q14129]
1.18	SLC6A12	solute carrier family 6 (neurotransmitter transporter). member 12
1.18	SLFN11	schlafen family member 11
1.18	USP9Y; TTTY15	ubiquitin specific peptidase 9. Y-linked; testis-specific transcript. Y-linked 15 (non-protein coding)
1.18	C14orf119	chromosome 14 open reading frame 119
1.18	MGAT4C	MGAT4 family. member C
1.18	RPL24	ribosomal protein L24
1.18	RPL39; SNORA69	ribosomal protein L39; small nucleolar RNA. H/ACA box 69
1.18	AVPR2	arginine vasopressin receptor 2
1.18	AHCTF1	AT hook containing transcription factor 1
1.18	SMS	spermine synthase
1.18	TRPV3	transient receptor potential cation channel. subfamily V. member 3
1.18	PRM2	protamine 2
1.18	LOX	lysyl oxidase
1.18	CARD6	caspase recruitment domain family. member 6

1.18	OSTC	oligosaccharyltransferase complex subunit (non-catalytic)
1.18	THAP4	THAP domain containing 4
1.18	TRPM8	transient receptor potential cation channel. subfamily M. member 8
1.18	RPL8; MIR6850	ribosomal protein L8; microRNA 6850
1.18	CCDC59	coiled-coil domain containing 59
1.18	CLDN8	claudin 8
1.18	SOX17	SRY box 17
1.18	C1orf50	chromosome 1 open reading frame 50
1.18	PRAMEF7	PRAME family member 7
1.18	CASK	calcium/calmodulin-dependent serine protein kinase (MAGUK family)
1.18	FAM179B	family with sequence similarity 179. member B
1.18	ATE1	arginyltransferase 1
1.18	ACP6	acid phosphatase 6. lysophosphatidic
1.18	TFEC	transcription factor EC
1.18	LCN2	lipocalin 2
1.18	CDH12	cadherin 12. type 2 (N-cadherin 2)
1.18	CRLF3	cytokine receptor-like factor 3
1.18	PURB; MIR4657	purine-rich element binding protein B; microRNA 4657
1.18	ODF2L	outer dense fiber of sperm tails 2-like
1.18	PLXNB3	plexin B3
1.18	MYT1L	Transcript Identified by AceView. Entrez Gene ID(s) 23040
1.18	SYK	spleen tyrosine kinase
1.18	TSSK2	testis-specific serine kinase 2
1.18	MAN2A1	mannosidase. alpha. class 2A. member 1
1.18	DLAT	dihydrolipoamide S-acetyltransferase
1.18	BCAS2	breast carcinoma amplified sequence 2

1.18	UBQLN4	ubiquilin 4
1.18	EPHB1	EPH receptor B1
1.18	RPL7A	ribosomal protein L7a
1.18	BTG2	BTG family. member 2
1.18	FAM69A	family with sequence similarity 69. member A
1.18	IDI2	isopentenyl-diphosphate delta isomerase 2
1.18	AHNAK2	AHNAK nucleoprotein 2
1.18	BCL9L	B-cell CLL/lymphoma 9-like
1.18	TLK1	tousled-like kinase 1
1.18	ZNF502	zinc finger protein 502
1.18	RPL17; SNORD58A; SNORD58B; SNORD58C	ribosomal protein L17; small nucleolar RNA. C/D box 58A; small nucleolar RNA. C/D box 58B; small nucleolar RNA. C/D box 58C
1.18	TBC1D21	TBC1 domain family. member 21
1.17	GREM1	gremlin 1. DAN family BMP antagonist [Source:HGNC Symbol;Acc:HGNC:2001]
1.17	LYSMD2	LysM. putative peptidoglycan-binding. domain containing 2
1.17	PLEKHG2	pleckstrin homology domain containing. family G (with RhoGef domain) member 2
1.17	DPCD	deleted in primary ciliary dyskinesia homolog (mouse)
1.17	OR2J1	olfactory receptor. family 2. subfamily J. member 1 (gene/pseudogene)
1.17	ZNF223	zinc finger protein 223
1.17	DENND4C	DENN/MADD domain containing 4C
1.17	FUT10	fucosyltransferase 10 (alpha (1.3) fucosyltransferase)
1.17	TBC1D13	TBC1 domain family. member 13
1.17	NSUN3	Transcript Identified by AceView. Entrez Gene ID(s) 63899
1.17	ECD	ecdysoneless homolog (Drosophila)
1.17	CALCB	calcitonin-related polypeptide beta

1.17	IGFBP5	insulin like growth factor binding protein 5
1.17	GPR180	G protein-coupled receptor 180
1.17	MRAP2	melanocortin 2 receptor accessory protein 2
1.17	PDLIM1	PDZ and LIM domain 1
1.17	DCST1	DC-STAMP domain containing 1
1.17	ARHGAP28	Rho GTPase activating protein 28
1.17	GLCE	glucuronic acid epimerase
1.17	CPM	carboxypeptidase M
1.17	KDM5D	lysine (K)-specific demethylase 5D
1.17	ZXDB	zinc finger. X-linked. duplicated B
1.17	POU2F2	POU class 2 homeobox 2
1.17	KRTAP19-6	keratin associated protein 19-6
1.17	IL1B	interleukin 1 beta
1.17	CARD9	caspase recruitment domain family. member 9
1.17	CYB5R1	cytochrome b5 reductase 1
1.17	FAM189B	family with sequence similarity 189. member B
1.17	STX16	syntaxin 16
1.17	AXDND1	axonemal dynein light chain domain containing 1
1.17	ATP6V1C2	ATPase. H+ transporting. lysosomal 42kDa. V1 subunit C2
1.17	PARG	poly (ADP-ribose) glycohydrolase
1.17	STAU2-AS1	STAU2 antisense RNA 1
1.17	ZNF835	zinc finger protein 835
1.17	OR52B6	olfactory receptor. family 52. subfamily B. member 6
1.17	MPP2	membrane protein. palmitoylated 2
1.17	HSPA4L	heat shock 70kDa protein 4-like
1.17	CDHR5	cadherin-related family member 5

1.17	SOCS5	suppressor of cytokine signaling 5
1.17	RHBDL3	Transcript Identified by AceView. Entrez Gene ID(s) 162494
1.17	RABGEF1	RAB guanine nucleotide exchange factor (GEF) 1
1.17	VEGFA	vascular endothelial growth factor A
1.17	PRODH	proline dehydrogenase (oxidase) 1
1.17	C16orf92	chromosome 16 open reading frame 92
1.17	THAP6	THAP domain containing 6
1.17	METTL24	methyltransferase like 24
1.17	RBX1	ring-box 1. E3 ubiquitin protein ligase
1.17	TRIM41	tripartite motif containing 41
1.17	BRD2	bromodomain containing 2
1.17	NACA2	nascent polypeptide-associated complex alpha subunit 2
1.17	CHST11	Transcript Identified by AceView. Entrez Gene ID(s) 50515
1.17	SESN3	Transcript Identified by AceView. Entrez Gene ID(s) 143686
1.17	CDKN2A	cyclin-dependent kinase inhibitor 2A
1.17	BMS1	BMS1 ribosome biogenesis factor
1.17	CEP104	centrosomal protein 104kDa
1.17	C9orf106	chromosome 9 open reading frame 106
1.17	FGD5	FYVE. RhoGEF and PH domain containing 5
1.17	FBXO10	F-box protein 10
1.17	RPL30	ribosomal protein L30
1.17	PLEKHH1	pleckstrin homology domain containing. family H (with MyTH4 domain) member 1
1.17	STEAP3	STEAP family member 3. metalloreductase
1.17	SLC15A5	solute carrier family 15. member 5
1.17	NDUFAF2	NADH dehydrogenase (ubiquinone) complex I. assembly factor 2

1.17	TARBP2	TAR (HIV-1) RNA binding protein 2
1.17	PYDC2	pyrin domain containing 2
1.17	TMEM156	transmembrane protein 156
1.17	TLE2	transducin-like enhancer of split 2
1.17	CUL4A	cullin 4A
1.17	OR5B3	olfactory receptor. family 5. subfamily B. member 3
1.17	SLFN5	schlafen family member 5
1.17	C1orf146	chromosome 1 open reading frame 146
1.17	CMIP	c-Maf inducing protein
1.17	ANXA4	Salzman2013 ANTISENSE. coding. INTERNAL. intronic best transcript NM_001153
1.17	AKR1C4	aldo-keto reductase family 1. member C4
1.17	GGT7	gamma-glutamyltransferase 7
1.17	PPIL1	peptidylprolyl isomerase (cyclophilin)-like 1
1.17	LRRC16B	leucine rich repeat containing 16B
1.17	EIF4B	eukaryotic translation initiation factor 4B
1.17	TULP3	tubby like protein 3
1.17	AP3S2; MIR5009	adaptor-related protein complex 3. sigma 2 subunit; microRNA 5009
1.17	RPP38	ribonuclease P/MRP 38kDa subunit
1.17	DNAJA1	DnaJ (Hsp40) homolog. subfamily A. member 1
1.17	POU3F4	POU class 3 homeobox 4
1.17	ZNF573	zinc finger protein 573
1.17	ARHGAP29	Rho GTPase activating protein 29
1.17	DERL1	derlin 1
1.17	GJB4	gap junction protein beta 4
1.17	ZSCAN22; MIR6806	zinc finger and SCAN domain containing 22; microRNA 6806

1.17	XK	X-linked Kx blood group
1.17	HSF1	heat shock transcription factor 1
1.17	ACTR3C	ARP3 actin-related protein 3 homolog C (yeast)
1.17	MKNK2	MAP kinase interacting serine/threonine kinase 2
1.17	RIF1	replication timing regulatory factor 1
1.17	TMLHE	trimethyllysine hydroxylase. epsilon
1.17	CACNA1A	calcium channel. voltage-dependent. P/Q type. alpha 1A subunit
1.17	ZNF35	zinc finger protein 35
1.17	KIZ	kizuna centrosomal protein
1.17	COIL	coilin
1.17	MED21	mediator complex subunit 21
1.17	IFI44L	interferon-induced protein 44-like
1.17	KCNK10	potassium channel. two pore domain subfamily K. member 10
1.17	TNNI3K	TNNI3 interacting kinase
1.17	DEFB125	defensin. beta 125
1.17	CAV3	caveolin 3
1.17	MYO1H	myosin IH
1.17	CHD8	chromodomain helicase DNA binding protein 8
1.17	NOB1	NIN1/RPN12 binding protein 1 homolog
1.17	ZC3H14	zinc finger CCCH-type containing 14
1.17	TFEB	transcription factor EB
1.17	RSPH9	radial spoke head 9 homolog (Chlamydomonas)
1.17	ZFYVE16	zinc finger. FYVE domain containing 16
1.17	SART3	squamous cell carcinoma antigen recognized by T-cells 3
1.17	FETUB	fetuin B
1.17	OR7D4	olfactory receptor. family 7. subfamily D. member 4

1.17	ZNF524	zinc finger protein 524
1.17	PLOD3	procollagen-lysine. 2-oxoglutarate 5-dioxygenase 3
1.17	RRP12	Memczak2013 ALT_ACCEPTOR. ALT_DONOR. coding. INTERNAL. intronic best transcript NM_001145114
1.17	PRDM1	PR domain containing 1. with ZNF domain
1.17	ARAP3	ArfGAP with RhoGAP domain. ankyrin repeat and PH domain 3
1.17	FGFBP2	fibroblast growth factor binding protein 2
1.17	TRIM33	tripartite motif containing 33
1.17	RPL11	ribosomal protein L11
1.17	SAMD8	sterile alpha motif domain containing 8
1.17	NT5M	5.3-nucleotidase. mitochondrial
1.17	C1orf74	chromosome 1 open reading frame 74
1.17	RAPGEF1	Rap guanine nucleotide exchange factor 1
1.17	CLCN4	chloride channel. voltage-sensitive 4
1.17	TFB1M	transcription factor B1. mitochondrial
1.17	GLI3	GLI family zinc finger 3
1.17	SLC25A40	solute carrier family 25. member 40
1.17	TMEM236	transmembrane protein 236
1.17	ITFG2	integrin alpha FG-GAP repeat containing 2
1.17	OR10A3	olfactory receptor. family 10. subfamily A. member 3
1.17	NPBWR1	neuropeptides B/W receptor 1
1.17	MSL3	male-specific lethal 3 homolog (Drosophila)
1.17	SEPN1	selenoprotein N. 1
1.17	MEIOC	meiosis specific with coiled-coil domain
1.17	ENOPH1	enolase-phosphatase 1
1.17	SUSD6	sushi domain containing 6

1.17	KERA	keratocan
1.17	ZNF780A	zinc finger protein 780A
1.17	SNX32	sorting nexin 32
1.17	FFAR1	free fatty acid receptor 1
1.17	OR4K15	olfactory receptor. family 4. subfamily K. member 15
1.17	RPEL1	ribulose-5-phosphate-3-epimerase-like 1
1.17	RIC1	RIC1 homolog. RAB6A GEF complex partner 1
1.17	GSE1	Gse1 coiled-coil protein
1.17	JAK1	Janus kinase 1
1.17	ZFR	zinc finger RNA binding protein
1.17	PTPN11	protein tyrosine phosphatase. non-receptor type 11
1.17	LINC00615	long intergenic non-protein coding RNA 615
1.17	SLC15A4	solute carrier family 15 (oligopeptide transporter). member 4
1.17	TSNAX	translin-associated factor X
1.17	YME1L1	YME1-like 1 ATPase
1.17	STK25	serine/threonine kinase 25
1.17	ANKRD63	ankyrin repeat domain 63
1.17	TEKT4	tektin 4
1.17	MRPL44	mitochondrial ribosomal protein L44
1.17	SLC25A6	solute carrier family 25 (mitochondrial carrier; adenine nucleotide translocator). member 6
1.17	GPR179	G protein-coupled receptor 179
1.17	CAP1	CAP. adenylate cyclase-associated protein 1 (yeast)
1.17	LAS1L	LAS1-like. ribosome biogenesis factor
1.17	MAP1LC3B2	microtubule-associated protein 1 light chain 3 beta 2
1.17	RFX2	regulatory factor X. 2 (influences HLA class II expression)

1.17	ADSL	adenylosuccinate lyase
1.17	TMEM38A	Transcript Identified by AceView. Entrez Gene ID(s) 79041
1.17	KMT2C	lysine (K)-specific methyltransferase 2C
1.17	GPR149	G protein-coupled receptor 149
1.17	TM4SF18	Transcript Identified by AceView. Entrez Gene ID(s) 116441
1.17	CTDSPL	CTD small phosphatase like
1.17	CCNDBP1	cyclin D-type binding-protein 1
1.17	GLI2	Jeck2013 ALT_ACCEPTOR. ALT_DONOR. coding. INTERNAL. intronic best transcript NM_005270
1.17	KLHL38	kelch-like family member 38
1.17	EIF2S2	Zhang2013 ALT_ACCEPTOR. ALT_DONOR. coding. INTERNAL. intronic best transcript NM_003908
1.17	PRMT6	protein arginine methyltransferase 6
1.17	RIMBP3C	RIMS binding protein 3C
1.17	FOXO1	forkhead box O1
1.17	FBXO3	F-box protein 3
1.17	C2orf78	chromosome 2 open reading frame 78
1.17	TBC1D31	TBC1 domain family. member 31
1.17	DCHS1	dachsous cadherin-related 1
1.17	LETM1	leucine zipper-EF-hand containing transmembrane protein 1
1.17	SPATS2L	spermatogenesis associated. serine-rich 2-like
1.17	RSPH1	radial spoke head 1 homolog (Chlamydomonas)
1.17	SF3B3	splicing factor 3b subunit 3
1.17	ADAM33	ADAM metallopeptidase domain 33
1.17	RIOK1	RIO kinase 1
1.17	TEX30	testis expressed 30
1.17	FMN1	formin 1

1.17	ATG16L1	autophagy related 16-like 1
1.17	NPTN	neuroplastin
1.17	SYNGR3	synaptogyrin 3
1.17	SDHAF1	succinate dehydrogenase complex assembly factor 1
1.17	DDX27	DEAD (Asp-Glu-Ala-Asp) box polypeptide 27
1.17	LAMA2	laminin. alpha 2
1.17	PTCH2	patched 2
1.17	TMED5	transmembrane p24 trafficking protein 5
1.17	RPS25	ribosomal protein S25
1.17	RHAG	Rh-associated glycoprotein
1.17	MYH2	myosin. heavy chain 2. skeletal muscle. adult
1.17	PSMB6	proteasome subunit beta 6
1.17	BMPR1A	bone morphogenetic protein receptor type IA
1.17	PAK2	p21 protein (Cdc42/Rac)-activated kinase 2
1.17	NFX1	nuclear transcription factor. X-box binding 1
1.17	PRND	prion protein 2 (dublet)
1.17	DNAJC7	DnaJ (Hsp40) homolog. subfamily C. member 7
1.16	RPL7A	ribosomal protein L7a
1.16	CECR2	cat eye syndrome chromosome region. candidate 2
1.16	LETMD1	LETM1 domain containing 1
1.16	CLRN1	clarin 1
1.16	ZNF532	zinc finger protein 532
1.16	PMFBP1	polyamine modulated factor 1 binding protein 1
1.16	SREK1	splicing regulatory glutamine/lysine-rich protein 1
1.16	TMX1	thioredoxin-related transmembrane protein 1
1.16	TTLL7	tubulin tyrosine ligase-like family member 7

1.16	RYK	receptor-like tyrosine kinase
1.16	VAPA	VAMP associated protein A
1.16	LRRC6	leucine rich repeat containing 6
1.16	ZFP82	ZFP82 zinc finger protein
1.16	PLXNA4	plexin A4
1.16	CCNH	cyclin H
1.16	RALBP1	ralA binding protein 1
1.16	RBP3	retinol binding protein 3. interstitial
1.16	ADSS	adenylosuccinate synthase
1.16	ZAP70	zeta chain of T cell receptor associated protein kinase 70kDa
1.16	SPTLC1	Transcript Identified by AceView. Entrez Gene ID(s) 10558
1.16	RERG	RAS-like. estrogen-regulated. growth inhibitor
1.16	TRIM22	tripartite motif containing 22
1.16	PHF1	PHD finger protein 1
1.16	GATAD2A; MIR640	GATA zinc finger domain containing 2A; microRNA 640
1.16	ZNF772	zinc finger protein 772
1.16	KIFC2	kinesin family member C2
1.16	BAG5	BCL2-associated athanogene 5
1.16	ZBTB43	zinc finger and BTB domain containing 43
1.16	AHR	aryl hydrocarbon receptor
1.16	sept-10	septin 10
1.16	DDX24	DEAD (Asp-Glu-Ala-Asp) box helicase 24
1.16	PITPNB	phosphatidylinositol transfer protein. beta
1.16	HSPD1	heat shock 60kDa protein 1 (chaperonin)
1.16	TMEM14C	transmembrane protein 14C
1.16	MDH1	malate dehydrogenase 1

1.16	COMMD8	COMM domain containing 8
1.16	C11orf91	chromosome 11 open reading frame 91
1.16	FAM199X	family with sequence similarity 199. X-linked
1.16	OR10A2	olfactory receptor. family 10. subfamily A. member 2
1.16	IL5	interleukin 5
1.16	KIDINS220	kinase D-interacting substrate 220kDa
1.16	FAM132B	family with sequence similarity 132. member B
1.16	UBE3C	ubiquitin protein ligase E3C
1.16	TC2N	tandem C2 domains. nuclear
1.16	ATL2	atlastin GTPase 2
1.16	CSGALNACT1	chondroitin sulfate N-acetylgalactosaminyltransferase 1
1.16	SLC4A1AP	solute carrier family 4 (anion exchanger). member 1. adaptor protein
1.16	VPS29	VPS29 retromer complex component
1.16	ASB1	ankyrin repeat and SOCS box containing 1
1.16	TMCO4	transmembrane and coiled-coil domains 4
1.16	SLC35A5	solute carrier family 35. member A5
1.16	AP1AR	adaptor-related protein complex 1 associated regulatory protein
1.16	ZNF207; MIR632	zinc finger protein 207; microRNA 632
1.16	UQCRB	ubiquinol-cytochrome c reductase binding protein
1.16	ZDHHC4	zinc finger. DHHC-type containing 4
1.16	ARIH2	ariadne RBR E3 ubiquitin protein ligase 2
1.16	FBXL21	F-box and leucine-rich repeat protein 21 (gene/pseudogene)
1.16	PROCR	protein C receptor. endothelial
1.16	ARHGAP21	Rho GTPase activating protein 21
1.16	HHAT	hedgehog acyltransferase
1.16	sept-07	septin 7

1.16	EXOC6	exocyst complex component 6
1.16	CPA6	carboxypeptidase A6
1.16	ATP6V1G3	ATPase. H+ transporting. lysosomal 13kDa. V1 subunit G3
1.16	FHAD1	forkhead-associated (FHA) phosphopeptide binding domain 1
1.16	STH	saitohin
1.16	SLC30A7	solute carrier family 30 (zinc transporter). member 7
1.16	CDC42SE2	CDC42 small effector 2
1.16	STK19	Homo sapiens serine/threonine kinase 19 (STK19). transcript variant 1. mRNA.; Homo sapiens serine/threonine kinase 19 (STK19). transcript variant 2. mRNA.; Homo sapiens serine/threonine kinase 19 (STK19). transcript variant 3. non-coding RNA.; Homo sapiens serine/threonine kinase 19. mRNA (cDNA clone MGC:117388 IMAGE:5165123). complete cds.
1.16	RBM45	RNA binding motif protein 45
1.16	CHRNA10; NUP98	cholinergic receptor. nicotinic alpha 10; nucleoporin 98kDa
1.16	IGSF21	immunoglobin superfamily. member 21
1.16	FRMPD3-AS1	FRMPD3 antisense RNA 1
1.16	ALG3	ALG3. alpha-1.3- mannosyltransferase
1.16	PQLC2L	PQ loop repeat containing 2-like
1.16	LAMTOR5	late endosomal/lysosomal adaptor. MAPK and MTOR activator 5
1.16	JSRP1	junctional sarcoplasmic reticulum protein 1
1.16	UNC13B	unc-13 homolog B (C. elegans)
1.16	TRIM67	tripartite motif containing 67
1.16	DEFB113	defensin. beta 113
1.16	GLT1D1	glycosyltransferase 1 domain containing 1
1.16	LPAR1	Jeck2013 ALT_ACCEPTOR. ALT_DONOR. coding. INTERNAL. intronic best transcript NM_057159; Salzman2013 ALT_ACCEPTOR. ALT_DONOR. coding. INTERNAL. intronic best transcript NM_057159
1.16	LHCGR	luteinizing hormone/choriogonadotropin receptor

1.16	DYRK3	dual specificity tyrosine-(Y)-phosphorylation regulated kinase 3
1.16	IGF2; INS-IGF2	insulin-like growth factor 2; INS-IGF2 readthrough
1.16	NES	nestin
1.16	GADD45A	growth arrest and DNA-damage-inducible. alpha
1.16	B4GALNT2	beta-1.4-N-acetyl-galactosaminyl transferase 2
1.16	HNRNPCL2	heterogeneous nuclear ribonucleoprotein C-like 2 [Source:HGNC Symbol;Acc:HGNC:48813]
1.16	BANF2	barrier to autointegration factor 2
1.16	PDK1	pyruvate dehydrogenase kinase. isozyme 1
1.16	ZSCAN12	zinc finger and SCAN domain containing 12
1.16	ASAH2B	N-acylsphingosine amidohydrolase (non-lysosomal ceramidase) 2B
1.16	HAS2	hyaluronan synthase 2
1.16	FBXO16	F-box protein 16
1.16	VPS11	VPS11. CORVET/HOPS core subunit [Source:HGNC Symbol;Acc:HGNC:14583]
1.16	CCNL2	cyclin L2
1.16	SLC35G3	solute carrier family 35. member G3
1.16	VPS4B	vacuolar protein sorting 4 homolog B (S. cerevisiae)
1.16	KLHL28	kelch-like family member 28
1.16	TCF23	transcription factor 23
1.16	KRT79	keratin 79. type II
1.16	IGF2BP3	insulin-like growth factor 2 mRNA binding protein 3
1.16	ENTPD4; LOXL2	ectonucleoside triphosphate diphosphohydrolase 4; lysyl oxidase-like 2
1.16	TMEM177	transmembrane protein 177
1.16	SLC37A3	solute carrier family 37. member 3
1.16	ZCCHC3	zinc finger. CCHC domain containing 3
1.16	OR6Q1	olfactory receptor. family 6. subfamily Q. member 1 (gene/pseudogene)

1.16	GCC1	GRIP and coiled-coil domain containing 1
1.16	BMX	BMX non-receptor tyrosine kinase
1.16	GIMAP1-GIMAP5; GIMAP5; GIMAP1	GIMAP1-GIMAP5 readthrough; GTPase. IMAP family member 5; GTPase. IMAP family member 1
1.16	COX7A1	cytochrome c oxidase subunit VIIa polypeptide 1 (muscle)
1.16	SPTBN2	spectrin. beta. non-erythrocytic 2
1.16	CFL2	cofilin 2 (muscle)
1.16	ERMN	ermin
1.16	CLCNKA	chloride channel. voltage-sensitive Ka
1.16	SYS1-DBNDD2	SYS1-DBNDD2 readthrough (NMD candidate)
1.16	GCGR	glucagon receptor
1.16	RPL31	ribosomal protein L31
1.16	TMEM131	transmembrane protein 131
1.16	CCDC93	coiled-coil domain containing 93
1.16	ACAP2; ACAP2-IT1; AC090018.3	Transcript Identified by AceView. Entrez Gene ID(s) 23527; ACAP2 intronic transcript 1 (non-protein coding) [Source:HGNC Symbol;Acc:HGNC:41426]; novel transcript; ACAP2 intronic transcript 1 [Source:HGNC Symbol;Acc:HGNC:41426]
1.16	IL7R	interleukin 7 receptor
1.16	DTX3L	deltex 3 like. E3 ubiquitin ligase
1.16	C12orf80	chromosome 12 open reading frame 80
1.16	USP24	ubiquitin specific peptidase 24
1.16	NDUFAF1	NADH dehydrogenase (ubiquinone) complex I. assembly factor 1
1.16	VCAM1	vascular cell adhesion molecule 1
1.16	S100A12	S100 calcium binding protein A12
1.16	CCDC151	coiled-coil domain containing 151
1.16	ZNF697	zinc finger protein 697

1.16	CLN8	ceroid-lipofuscinosis. neuronal 8
1.16	NR2F1	nuclear receptor subfamily 2. group F. member 1
1.16	AKR1C1	aldo-keto reductase family 1. member C1
1.16	OTUD1	OTU deubiquitinase 1
1.16	PLIN1	perilipin 1
1.16	PTPRF	protein tyrosine phosphatase. receptor type. F
1.16	RNF149	ring finger protein 149
1.16	ANHX	anomalous homeobox
1.16	SLC25A52	solute carrier family 25. member 52
1.16	WDYHV1	WDYHV motif containing 1
1.16	RPS5	ribosomal protein S5
1.16	RPS6KA1	ribosomal protein S6 kinase. 90kDa. polypeptide 1
1.16	TMEM170A	transmembrane protein 170A
1.16	ALS2	ALS2. alsin Rho guanine nucleotide exchange factor
1.16	ZNF502	zinc finger protein 502
1.16	CCNL1	cyclin L1
1.16	CT45A3; CT45A4; CT45A5	cancer/testis antigen family 45. member A3; cancer/testis antigen family 45. member A4; cancer/testis antigen family 45. member A5
1.16	WDR64	WD repeat domain 64
1.16	DNAJB4	DnaJ (Hsp40) homolog. subfamily B. member 4
1.16	GGTLC1	gamma-glutamyltransferase light chain 1
1.16	COPS5	COP9 signalosome subunit 5
1.16	C10orf142	chromosome 10 open reading frame 142
1.16	MYO9B	Memczak2013 ANTISENSE. CDS. coding. INTERNAL best transcript NM_004145
1.16	UBE2W	ubiquitin-conjugating enzyme E2W (putative)
1.16	HTR1B	5-hydroxytryptamine (serotonin) receptor 1B. G protein-coupled

1.16	PPP3R1	protein phosphatase 3. regulatory subunit B. alpha
1.16	INHBB	inhibin beta B
1.16	LINC00260	long intergenic non-protein coding RNA 260
1.16	NEUROG1	neurogenin 1
1.16	ATP1A1	ATPase. Na+/K+ transporting. alpha 1 polypeptide
1.16	METTL4	methyltransferase like 4
1.16	SNRNP35	small nuclear ribonucleoprotein. U11/U12 35kDa subunit
1.16	MAP3K1	mitogen-activated protein kinase kinase kinase 1. E3 ubiquitin protein ligase
1.16	OR1B1	olfactory receptor. family 1. subfamily B. member 1 (gene/pseudogene)
1.16	ACTG2	actin. gamma 2. smooth muscle. enteric
1.16	SNCAIP	synuclein alpha interacting protein
1.16	TPK1	thiamin pyrophosphokinase 1
1.16	SLC38A2	solute carrier family 38. member 2
1.16	PLD6	phospholipase D family. member 6
1.16	PSMG2	proteasome (prosome. macropain) assembly chaperone 2
1.16	CCDC91	coiled-coil domain containing 91
1.16	ILF2	Jeck2013 ALT_DONOR. coding. INTERNAL. intronic best transcript NM_004515
1.16	TMEM182	transmembrane protein 182
1.16	HSPH1	heat shock 105kDa/110kDa protein 1
1.16	GOLGA8DP	golgin A8 family. member D. pseudogene
1.16	PTGER2	prostaglandin E receptor 2
1.16	DNAJC9	DnaJ (Hsp40) homolog. subfamily C. member 9
1.16	UBE2Q1	ubiquitin-conjugating enzyme E2Q family member 1
1.16	TLR1	toll-like receptor 1
1.16	IQCF3	IQ motif containing F3

1.16	NDUFS4	NADH dehydrogenase (ubiquinone) Fe-S protein 4. 18kDa (NADH-coenzyme Q reductase)
1.16	ABL2	ABL proto-oncogene 2. non-receptor tyrosine kinase
1.16	LARS	leucyl-tRNA synthetase
1.16	ARHGEF38	Rho guanine nucleotide exchange factor 38
1.16	ZNF280A	zinc finger protein 280A
1.16	TAF1L	TAF1 RNA polymerase II. TATA box binding protein (TBP)-associated factor. 210kDa-like
1.16	MKS1	Meckel syndrome. type 1
1.16	ATXN1	Memczak2013 ALT_ACCEPTOR. ALT_DONOR. coding. INTERNAL. intronic best transcript NM_000332
1.16	ZNF780A	zinc finger protein 780A
1.16	DHX16	DEAH (Asp-Glu-Ala-His) box polypeptide 16
1.16	BORCS5	BLOC-1 related complex subunit 5
1.16	IKZF5	IKAROS family zinc finger 5
1.16	KNOP1	lysine-rich nucleolar protein 1
1.16	TFAP2D	transcription factor AP-2 delta (activating enhancer binding protein 2 delta)
1.16	VPS11	vacuolar protein sorting 11 homolog (S. cerevisiae)
1.16	PPM1A	protein phosphatase. Mg2+/Mn2+ dependent. 1A
1.16	DGKA	diacylglycerol kinase alpha
1.16	TMEM233	transmembrane protein 233
1.16	FOXK1	forkhead box K1
1.16	C10orf120	chromosome 10 open reading frame 120
1.16	POLR3D	polymerase (RNA) III (DNA directed) polypeptide D. 44kDa
1.16	ANAPC4	anaphase promoting complex subunit 4
1.16	NGF	nerve growth factor (beta polypeptide)
1.16	TMEM50A	transmembrane protein 50A

1.16	UNC5B	unc-5 netrin receptor B
1.16	NPIPB5	nuclear pore complex interacting protein family. member B5
1.16	ZNF713	zinc finger protein 713
1.16	FAM72D	Transcript Identified by AceView. Entrez Gene ID(s) 728833
1.16	C19orf48; SNORD88B; SNORD88C	chromosome 19 open reading frame 48; small nucleolar RNA. C/D box 88B; small nucleolar RNA. C/D box 88C
1.15	RBBP8NL	RBBP8 N-terminal like
1.15	POF1B	premature ovarian failure. 1B
1.15	MIR659; ANKRD54	microRNA 659; ankyrin repeat domain 54
1.15	AMOTL1	angiomotin like 1
1.15	CRYBG3	crystallin beta-gamma domain containing 3
1.15	CHCHD4	coiled-coil-helix-coiled-coil-helix domain containing 4
1.15	NAA10	N(alpha)-acetyltransferase 10. NatA catalytic subunit
1.15	BTF3L4	basic transcription factor 3-like 4
1.15	C9orf131	chromosome 9 open reading frame 131
1.15	OR5P2	olfactory receptor. family 5. subfamily P. member 2
1.15	MCMBP	minichromosome maintenance complex binding protein
1.15	MAP2K5	mitogen-activated protein kinase kinase 5
1.15	PPIAL4F	peptidylprolyl isomerase A (cyclophilin A)-like 4F
1.15	FEZ1	fasciculation and elongation protein zeta 1
1.15	CDADC1	cytidine and dCMP deaminase domain containing 1
1.15	NDUFA10	NADH dehydrogenase (ubiquinone) 1 alpha subcomplex. 10. 42kDa
1.15	BACH1	BTB and CNC homology 1. basic leucine zipper transcription factor 1
1.15	VANGL1	VANGL planar cell polarity protein 1
1.15	SLC12A7	solute carrier family 12 (potassium/chloride transporter). member 7

1.15	USP17L24; USP17L26; USP17L5; USP17L27; USP17L29; USP17L30	ubiquitin specific peptidase 17-like family member 24; ubiquitin specific peptidase 17-like family member 26; ubiquitin specific peptidase 17-like family member 5; ubiquitin specific peptidase 17-like family member 27; ubiquitin specific peptidase 17-like family member 29; ubiquitin specific peptidase 17-like family member 30
1.15	USP17L25; USP17L27	ubiquitin specific peptidase 17-like family member 25; ubiquitin specific peptidase 17-like family member 27
1.15	TRAPPC4	trafficking protein particle complex 4
1.15	TCF21	transcription factor 21
1.15	HYOU1	hypoxia up-regulated 1
1.15	MMP17	matrix metallopeptidase 17 (membrane-inserted)
1.15	PLEKHA7	pleckstrin homology domain containing. family A member 7
1.15	ADGRG4	adhesion G protein-coupled receptor G4
1.15	ADRBK1	adrenergic. beta. receptor kinase 1
1.15	PTPRG	protein tyrosine phosphatase. receptor type. G
1.15	SEC11A	SEC11 homolog A. signal peptidase complex subunit
1.15	S100A3	S100 calcium binding protein A3
1.15	ABCA6	ATP binding cassette subfamily A member 6
1.15	RAX	retina and anterior neural fold homeobox
1.15	ASIC3	acid sensing ion channel 3
1.15	RPL27	ribosomal protein L27
1.15	LOC283788; AL592183.1	FSHD region gene 1 pseudogene
1.15	TOMM20	translocase of outer mitochondrial membrane 20 homolog (yeast)
1.15	RCBTB2	regulator of chromosome condensation (RCC1) and BTB (POZ) domain containing protein 2
1.15	PIK3R6	phosphoinositide-3-kinase. regulatory subunit 6
1.15	CFH	complement factor H
1.15	MATN2	matrilin 2

1.15	CCNT2	cyclin T2
1.15	TMC6	transmembrane channel like 6
1.15	LOC441178; RP11-351J23.1	uncharacterized LOC441178; novel tanscript
1.15	MAP3K10	mitogen-activated protein kinase kinase kinase 10
1.15	ZNF202	zinc finger protein 202
1.15	ZFYVE27	zinc finger. FYVE domain containing 27
1.15	C16orf97	chromosome 16 open reading frame 97
1.15	SLC9A4	solute carrier family 9. subfamily A (NHE4. cation proton antiporter 4). member 4
1.15	TSTD2	thiosulfate sulfurtransferase (rhodanese)-like domain containing 2
1.15	SCGB2B2	secretoglobin. family 2B. member 2
1.15	ZCCHC7	zinc finger. CCHC domain containing 7
1.15	WBSCR17	Memczak2013 ALT_ACCEPTOR. ALT_DONOR. coding. INTERNAL. intronic best transcript NM_022479
1.15	DHX32	DEAH (Asp-Glu-Ala-His) box polypeptide 32
1.15	HMGN4	high mobility group nucleosomal binding domain 4
1.15	CDK5RAP2	CDK5 regulatory subunit associated protein 2
1.15	DCT	dopachrome tautomerase
1.15	SLC35G6	solute carrier family 35. member G6
1.15	LCOR	ligand dependent nuclear receptor corepressor
1.15	SPEF1	sperm flagellar 1
1.15	NLRP13	NLR family. pyrin domain containing 13
1.15	APIP	APAF1 interacting protein
1.15	MAD2L1BP	MAD2L1 binding protein
1.15	ENDOG	endonuclease G
1.15	NDUFA10	NADH dehydrogenase (ubiquinone) 1 alpha subcomplex. 10. 42kDa
1.15	LSP1P3	lymphocyte-specific protein 1 pseudogene 3

1.15	GBA2	glucosidase. beta (bile acid) 2
1.15	HEATR6	HEAT repeat containing 6
1.15	NEBL	nebulette
1.15	PPP2R5A	protein phosphatase 2. regulatory subunit B. alpha
1.15	TSSK1B	testis-specific serine kinase 1B
1.15	AZU1	azurocidin 1
1.15	MOB4	MOB family member 4. phocein
1.15	SFT2D1	SFT2 domain containing 1
1.15	CHN2	chimerin 2
1.15	TRPM2	transient receptor potential cation channel. subfamily M. member 2
1.15	MMP1	matrix metallopeptidase 1
1.15	ANAPC16	anaphase promoting complex subunit 16
1.15	FGD2	FYVE. RhoGEF and PH domain containing 2
1.15	LRRC37A2	leucine rich repeat containing 37. member A2
1.15	TMEM225	transmembrane protein 225
1.15	DNAJC18	DnaJ (Hsp40) homolog. subfamily C. member 18
1.15	ZNF22	zinc finger protein 22
1.15	SIL1	SIL1 nucleotide exchange factor
1.15	C1orf226	chromosome 1 open reading frame 226
1.15	EFCAB7; DLEU2L	EF-hand calcium binding domain 7; deleted in lymphocytic leukemia 2-like
1.15	C5orf67	chromosome 5 open reading frame 67
1.15	PARM1	prostate androgen-regulated mucin-like protein 1
1.15	PADI1	peptidyl arginine deiminase. type I
1.15	ARPP21	Transcript Identified by AceView. Entrez Gene ID(s) 10777
1.15	PDCD6IP	programmed cell death 6 interacting protein
1.15	GINS3	GINS complex subunit 3 (Psf3 homolog)

1.15	TRPM3	transient receptor potential cation channel. subfamily M. member 3
1.15	NOC4L	nucleolar complex associated 4 homolog
1.15	C8orf58	chromosome 8 open reading frame 58
1.15	RAD23A	RAD23 homolog A. nucleotide excision repair protein
1.15	ZFP64	ZFP64 zinc finger protein
1.15	GADD45GIP1	growth arrest and DNA-damage-inducible. gamma interacting protein 1
1.15	PQLC2	PQ loop repeat containing 2
1.15	TSSC4	tumor suppressing subtransferable candidate 4
1.15	GAN; MIR4720	gigaxonin; microRNA 4720
1.15	CLCN1	chloride channel. voltage-sensitive 1
1.15	CCDC36	coiled-coil domain containing 36
1.15	KLHDC2	kelch domain containing 2
1.15	AP2B1	adaptor-related protein complex 2. beta 1 subunit
1.15	CCDC129	coiled-coil domain containing 129
1.15	HDX	highly divergent homeobox
1.15	RTN3	reticulon 3
1.15	NEDD9	neural precursor cell expressed. developmentally down-regulated 9
1.15	H1FNT	H1 histone family. member N. testis-specific
1.15	SPRR2A	small proline-rich protein 2A
1.15	LOC149373; RP5-1097F14.3; kosimo	uncharacterized LOC149373; Transcript Identified by AceView; novel transcript
1.15	BTK	Bruton agammaglobulinemia tyrosine kinase
1.15	ZNF284	zinc finger protein 284
1.15	PLCL2; MIR3714	phospholipase C-like 2; microRNA 3714
1.15	HTR1A	5-hydroxytryptamine (serotonin) receptor 1A. G protein-coupled
1.15	MBLAC2	metallo-beta-lactamase domain containing 2

1.15	PRKRA	protein kinase. interferon-inducible double stranded RNA dependent activator
1.15	MEFV	Mediterranean fever
1.15	SCN2B	sodium channel. voltage gated. type II beta subunit
1.15	TTC5	tetratricopeptide repeat domain 5
1.15	GRK4	G protein-coupled receptor kinase 4
1.15	GAGE12H; GAGE12B; GAGE12C	G antigen 12H; G antigen 12B; G antigen 12C
1.15	GAGE12D	G antigen 12D
1.15	GAGE12G; GAGE12E	G antigen 12G; G antigen 12E
1.15	C16orf52	chromosome 16 open reading frame 52
1.15	CTNNA2	catenin (cadherin-associated protein). alpha 2
1.15	LRRC4B	leucine rich repeat containing 4B
1.15	RGPD5; RGPD8	RANBP2-like and GRIP domain containing 5; RANBP2-like and GRIP domain containing 8
1.15	ATP6AP1L	ATPase. H+ transporting. lysosomal accessory protein 1-like
1.15	FKBP6	FK506 binding protein 6
1.15	ZNF302	zinc finger protein 302
1.15	DZIP1L	DAZ interacting zinc finger protein 1-like
1.15	WNT3A	wingless-type MMTV integration site family. member 3A
1.15	PSMG3	proteasome (prosome. macropain) assembly chaperone 3
1.15	TSHZ3	teashirt zinc finger homeobox 3
1.15	ADAMTSL1	ADAMTS like 1
1.15	RUNX1	runt-related transcription factor 1
1.15	LSAMP	limbic system-associated membrane protein
1.15	ARRB2	arrestin. beta 2
1.15	ACVR2B	activin A receptor type IIB
1.15	LSM6	Transcript Identified by AceView. Entrez Gene ID(s) 11157

1.15	RNMT	RNA (guanine-7-) methyltransferase
1.15	PLEKHS1	pleckstrin homology domain containing. family S member 1
1.15	TOP1	topoisomerase (DNA) I
1.15	PRKAR2A	protein kinase. cAMP-dependent. regulatory. type II. alpha
1.15	AIDA	axin interactor. dorsalization associated
1.15	SYCE1; SPRNP1	synaptonemal complex central element protein 1; shadow of prion protein homolog (zebrafish) pseudogene 1
1.15	HIGD1B	HIG1 hypoxia inducible domain family. member 1B
1.15	CFTR	cystic fibrosis transmembrane conductance regulator
1.15	C10orf105	chromosome 10 open reading frame 105
1.15	HYOU1	hypoxia up-regulated 1
1.15	OOEP	oocyte expressed protein
1.15	LOC101928161; AC010890.1; NCKAP5; spawfa	uncharacterized LOC101928161; Salzman2013 ANTISENSE. coding. INTERNAL. intronic. OVERLAPTX best transcript NM_207363; novel transcript. antisense to NCKAP5; Transcript Identified by AceView
1.15	FAM110B	family with sequence similarity 110. member B
1.15	NAT9	N-acetyltransferase 9 (GCN5-related. putative)
1.15	COPG2; TSGA13	coatomer protein complex subunit gamma 2; testis specific 13
1.15	PLAGL2	pleiomorphic adenoma gene-like 2
1.15	MARCH10	membrane associated ring finger 10
1.15	INPP5D	inositol polyphosphate-5-phosphatase D
1.15	DACT1	Transcript Identified by AceView. Entrez Gene ID(s) 51339
1.15	BRMS1L	Transcript Identified by AceView. Entrez Gene ID(s) 84312
1.15	ANKRD30A	ankyrin repeat domain 30A
1.15	COX4I2	cytochrome c oxidase subunit IV isoform 2 (lung)
1.15	MYEOV	myeloma overexpressed
1.15	ESCO1	establishment of sister chromatid cohesion N-acetyltransferase 1

1.15	TRIM32	tripartite motif containing 32
1.15	ZBTB44	zinc finger and BTB domain containing 44
1.15	AFF3	AF4/FMR2 family. member 3
1.15	TRIM23	tripartite motif containing 23
1.15	UBE2D2	ubiquitin conjugating enzyme E2D 2
1.15	TRPS1	trichorhinophalangeal syndrome I
1.15	RASGRP3	RAS guanyl releasing protein 3 (calcium and DAG-regulated)
1.15	CTLA4	cytotoxic T-lymphocyte-associated protein 4
1.15	GFAP	glial fibrillary acidic protein
1.15	PTPN7	protein tyrosine phosphatase. non-receptor type 7
1.15	NUB1	negative regulator of ubiquitin-like proteins 1
1.15	DGCR8	Memczak2013 ALT_ACCEPTOR. ALT_DONOR. coding. INTERNAL. intronic best transcript NM_022720
1.15	C1RL	complement component 1. r subcomponent-like
1.15	ZNF512	Memczak2013 ANTISENSE. coding. INTERNAL. UTR3 best transcript NM_032434
1.15	LRRC37B	leucine rich repeat containing 37B
1.15	CHD4	chromodomain helicase DNA binding protein 4
1.15	TAS2R19	taste receptor. type 2. member 19
1.15	PLEKHA5	pleckstrin homology domain containing. family A member 5
1.15	RPL34	ribosomal protein L34
1.15	SMC1B	structural maintenance of chromosomes 1B
1.15	CHRDL1	chordin-like 1
1.15	ZNF347	zinc finger protein 347
1.15	LOC388242; LOC613038; RP11-347C12.3	Homo sapiens coiled-coil domain containing 101 pseudogene (LOC388242). non-coding RNA.; Homo sapiens SAGA complex associated factor 29 pseudogene (LOC613038). non-coding RNA.; Transcript Identified by AceView. Entrez Gene ID(s) 613038. RefSeq ID(s) NR_002557; Transcript

		Identified by AceView. Entrez Gene ID(s) 613038; Protein LOC101929849 [Source:UniProtKB/TrEMBL;Acc:H0YHN6]
1.15	PCYT1A	phosphate cytidylyltransferase 1. choline. alpha
1.15	MCEMP1	mast cell-expressed membrane protein 1
1.15	DOCK10	Transcript Identified by AceView. Entrez Gene ID(s) 55619
1.15	CAMK1D	calcium/calmodulin-dependent protein kinase ID
1.15	KCND3	potassium channel. voltage gated Shal related subfamily D. member 3
1.15	POMK	protein-O-mannose kinase
1.15	FBXO15	F-box protein 15
1.15	PSMA3	proteasome subunit alpha 3
1.15	RNASEH1	ribonuclease H1
1.15	GAS6	growth arrest-specific 6
1.15	RBM44	RNA binding motif protein 44
1.15	SUPT7L	SPT7-like STAGA complex gamma subunit
1.15	SFPQ	splicing factor proline/glutamine-rich
1.15	RASIP1	Ras interacting protein 1
1.15	GOLGA6L9	golgin A6 family-like 9
1.15	FCMR	Fc fragment of IgM receptor
1.15	PCSK1	proprotein convertase subtilisin/kexin type 1
1.15	NAALAD2	N-acetylated alpha-linked acidic dipeptidase 2
1.15	MANBA	mannosidase. beta A. lysosomal
1.15	STT3B	STT3B. subunit of the oligosaccharyltransferase complex (catalytic)
1.15	CBL	Memczak2013 ANTISENSE. coding. INTERNAL. UTR3 best transcript NM_005188
1.15	SCRIB	Zhang2013 ALT_ACCEPTOR. ALT_DONOR. coding. INTERNAL. intronic best transcript NM_182706

1.15	SUN1	Sad1 and UNC84 domain containing 1
1.15	TAF7	TAF7 RNA polymerase II. TATA box binding protein (TBP)-associated factor. 55kDa
1.15	GPR146	G protein-coupled receptor 146
1.15	GRAMD1B	GRAM domain containing 1B
1.15	ZNF600	zinc finger protein 600
1.15	SLC5A5	solute carrier family 5 (sodium/iodide cotransporter). member 5
1.15	M1AP	meiosis 1 associated protein
1.15	ARID5A	AT rich interactive domain 5A (MRF1-like)
1.15	ZNF878	zinc finger protein 878
1.15	MRPL33	mitochondrial ribosomal protein L33
1.15	CTCFL	CCCTC-binding factor (zinc finger protein)-like
1.15	ZNF106	zinc finger protein 106
1.15	ZDHHC8	zinc finger. DHHC-type containing 8
1.15	PDIK1L	PDLIM1 interacting kinase 1 like
1.15	SCT	secretin
1.15	PWP2	PWP2 periodic tryptophan protein homolog (yeast)
1.15	LUC7L	LUC7-like
1.15	CACNA1C	calcium channel. voltage-dependent. L type. alpha 1C subunit
1.15	IFNA16	interferon. alpha 16
1.15	TBC1D25	TBC1 domain family. member 25
1.15	SPANXA1	sperm protein associated with the nucleus. X-linked. family member A1
1.15	RRP1B	ribosomal RNA processing 1B
1.15	CYSRT1	cysteine-rich tail protein 1
1.15	PDE1A	Transcript Identified by AceView. Entrez Gene ID(s) 5136
1.15	TTLL11	tubulin tyrosine ligase-like family member 11

1.15	CRYGN	crystallin gamma N
1.15	CNTNAP1	contactin associated protein 1
1.15	GPCPD1	glycerophosphocholine phosphodiesterase 1
1.15	TAS2R4	taste receptor. type 2. member 4
1.15	ANKRD45	ankyrin repeat domain 45
1.15	CT45A5	cancer/testis antigen family 45. member A5
1.15	C1orf158	Transcript Identified by AceView. Entrez Gene ID(s) 93190
1.15	METTL9	methyltransferase like 9
1.15	CUL5	cullin 5
1.15	CT45A2	cancer/testis antigen family 45. member A2
1.15	KCNJ15	potassium channel. inwardly rectifying subfamily J. member 15
1.15	OR2B2	olfactory receptor. family 2. subfamily B. member 2
1.15	WBP4	WW domain binding protein 4
1.15	USP53	ubiquitin specific peptidase 53
1.15	PDE12	phosphodiesterase 12
1.15	MAMDC2	MAM domain containing 2
1.15	TXN	thioredoxin
1.15	CMTM4	CKLF-like MARVEL transmembrane domain containing 4
1.15	SLC8A3	solute carrier family 8 (sodium/calcium exchanger). member 3
1.14	EPB41L1	erythrocyte membrane protein band 4.1-like 1
1.14	TIPIN; RPL9P25	TIMELESS interacting protein; ribosomal protein L9 pseudogene 25
1.14	SLC9A7	solute carrier family 9. subfamily A (NHE7. cation proton antiporter 7). member 7
1.14	TGFBRAP1	transforming growth factor beta receptor associated protein 1
1.14	PIGL	phosphatidylinositol glycan anchor biosynthesis class L
1.14	RBM27	RNA binding motif protein 27

1.14	GNG10	guanine nucleotide binding protein (G protein). gamma 10
1.14	INO80C	INO80 complex subunit C
1.14	OPHN1	oligophrenin 1
1.14	TTC33	tetratricopeptide repeat domain 33
1.14	RHOU	ras homolog family member U
1.14	ICOSLG	inducible T-cell co-stimulator ligand
1.14	WFDC3	WAP four-disulfide core domain 3
1.14	FGF8	fibroblast growth factor 8 (androgen-induced)
1.14	PPP2R5C	protein phosphatase 2. regulatory subunit B. gamma
1.14	PPP1R26	protein phosphatase 1. regulatory subunit 26
1.14	WNK1	WNK lysine deficient protein kinase 1
1.14	EEFSEC	eukaryotic elongation factor. selenocysteine-tRNA-specific
1.14	BICD2	bicaudal D homolog 2 (Drosophila)
1.14	WDFY1	WD repeat and FYVE domain containing 1
1.14	KXD1	KxDL motif containing 1
1.14	EEF2; SNORD37	eukaryotic translation elongation factor 2; small nucleolar RNA. C/D box 37
1.14	KIAA1958	KIAA1958
1.14	MAPK1IP1L	mitogen-activated protein kinase 1 interacting protein 1-like
1.14	CYP2A6	cytochrome P450. family 2. subfamily A. polypeptide 6
1.14	EPB41L4B	erythrocyte membrane protein band 4.1 like 4B
1.14	C11orf57	chromosome 11 open reading frame 57
1.14	TGM5	transglutaminase 5
1.14	SPAM1	sperm adhesion molecule 1 (PH-20 hyaluronidase. zona pellucida binding)
1.14	ACHE	acetylcholinesterase (Yt blood group)
1.14	DDX52	DEAD (Asp-Glu-Ala-Asp) box polypeptide 52
1.14	SPATC1L	spermatogenesis and centriole associated 1-like

1.14	FAM3D	family with sequence similarity 3. member D
1.14	MMEL1	membrane metallo-endopeptidase-like 1
1.14	CAPSL	calcyphosine-like
1.14	SOAT2	sterol O-acyltransferase 2
1.14	CYP2J2	cytochrome P450. family 2. subfamily J. polypeptide 2
1.14	FER1L6	fer-1-like family member 6
1.14	COG1	component of oligomeric golgi complex 1
1.14	TBP	TATA box binding protein
1.14	IST1	increased sodium tolerance 1 homolog (yeast)
1.14	WNT4	wingless-type MMTV integration site family. member 4
1.14	USP17L2	ubiquitin specific peptidase 17-like family member 2
1.14	FAM198B	family with sequence similarity 198. member B
1.14	OR4N2	olfactory receptor. family 4. subfamily N. member 2
1.14	YIPF7	Yip1 domain family member 7
1.14	ZNF780B	zinc finger protein 780B
1.14	ALS2CR11	amyotrophic lateral sclerosis 2 chromosome region candidate 11
1.14	ZNF780B	zinc finger protein 780B
1.14	OPN1SW	opsin 1 (cone pigments). short-wave-sensitive
1.14	LRRIQ4	leucine-rich repeats and IQ motif containing 4
1.14	KCNU1	potassium channel. subfamily U. member 1
1.14	MTBP	MDM2 binding protein
1.14	GAGE4; GAGE7; GAGE12G	G antigen 4; G antigen 7; G antigen 12G
1.14	TOMM5	translocase of outer mitochondrial membrane 5 homolog (yeast)
1.14	DNAH17	dynein. axonemal. heavy chain 17
1.14	B4GALT2	UDP-Gal:betaGlcNAc beta 1.4- galactosyltransferase. polypeptide 2
1.14	TGFBR2	transforming growth factor beta receptor II

1.14	ZNF658	zinc finger protein 658
1.14	NRP1	neuropilin 1
1.14	SNX30	sorting nexin family member 30
1.14	HLX	H2.0-like homeobox
1.14	LTBP3	latent transforming growth factor beta binding protein 3
1.14	PLA2G10	phospholipase A2. group X
1.14	UBE2F	ubiquitin-conjugating enzyme E2F (putative)
1.14	GEMIN8	gem nuclear organelle associated protein 8
1.14	OR5L2	olfactory receptor. family 5. subfamily L. member 2
1.14	SNX5	sorting nexin 5
1.14	NOTCH2	notch 2
1.14	CTTN	cortactin
1.14	MANSC1	MANSC domain containing 1
1.14	FOPNL	FGFR1OP N-terminal like
1.14	SERTAD3	SERTA domain containing 3
1.14	GOLGA8T	golgin A8 family. member T
1.14	AATF	apoptosis antagonizing transcription factor
1.14	DEAF1	DEAF1 transcription factor
1.14	CT45A7; CT45A6	cancer/testis antigen family 45. member A7; cancer/testis antigen family 45. member A6
1.14	ANKRD20A3; ANKRD20A2	ankyrin repeat domain 20 family. member A3; ankyrin repeat domain 20 family. member A2
1.14	POGZ	Transcript Identified by AceView. Entrez Gene ID(s) 23126
1.14	ACTR3	ARP3 actin-related protein 3 homolog (yeast)
1.14	MLH1	mutL homolog 1
1.14	GFPT1	glutamine--fructose-6-phosphate transaminase 1
1.14	SCGB2A2	secretoglobin. family 2A. member 2

1.14	UNC13A	unc-13 homolog A (C. elegans)
1.14	TRIM43	tripartite motif containing 43
1.14	B3GNT5	UDP-GlcNAc:betaGal beta-1.3-N-acetylglucosaminyltransferase 5
1.14	TIGIT	T-cell immunoreceptor with Ig and ITIM domains
1.14	RNF175	ring finger protein 175
1.14	INPP4A	inositol polyphosphate-4-phosphatase type I A
1.14	EXOSC9	exosome component 9
1.14	C8orf48	chromosome 8 open reading frame 48
1.14	PLCG2	phospholipase C. gamma 2 (phosphatidylinositol-specific)
1.14	ITGA11	integrin alpha 11
1.14	SLC2A1	solute carrier family 2 (facilitated glucose transporter). member 1
1.14	TBX5	T-box 5
1.14	ZNF627	Memczak2013 ANTISENSE. CDS. coding. INTERNAL best transcript NM_145295
1.14	OOSP2	oocyte secreted protein 2
1.14	ELF2	E74-like factor 2 (ets domain transcription factor)
1.14	OR51I1	olfactory receptor. family 51. subfamily I. member 1
1.14	P2RY12	purinergic receptor P2Y. G-protein coupled. 12
1.14	LCT	lactase
1.14	TBL1Y	transducin (beta)-like 1. Y-linked
1.14	PBX3	pre-B-cell leukemia homeobox 3
1.14	KRT27	keratin 27. type I
1.14	ANKIB1	ankyrin repeat and IBR domain containing 1
1.14	PRR27	proline rich 27
1.14	FAM69C	family with sequence similarity 69. member C
1.14	FUT8	fucosyltransferase 8 (alpha (1.6) fucosyltransferase)

1.14	ISL1	ISL LIM homeobox 1
1.14	TRMT6	tRNA methyltransferase 6
1.14	SQSTM1	Transcript Identified by AceView. Entrez Gene ID(s) 8878
1.14	RP11-569G13.3; LOC158434; setora	uncharacterized protein LOC158434 [Source:RefSeq peptide;Acc:NP_001243337]; uncharacterized LOC158434; Protein LOC158434 [Source:UniProtKB/TrEMBL;Acc:A0A087X204]; novel transcript; Transcript Identified by AceView
1.14	GPRIN1	G protein regulated inducer of neurite outgrowth 1
1.14	SMYD1	SET and MYND domain containing 1
1.14	CWC27	CWC27 spliceosome-associated protein homolog
1.14	ZNF263	zinc finger protein 263
1.14	ZNF343	zinc finger protein 343
1.14	C9orf129	chromosome 9 open reading frame 129
1.14	ASH1L; MIR555	ash1 (absent. small. or homeotic)-like (Drosophila); microRNA 555
1.14	H1FOO	H1 histone family. member O. oocyte-specific
1.14	MGST1	microsomal glutathione S-transferase 1
1.14	GOLGA5	golgin A5
1.14	NGFRAP1	nerve growth factor receptor (TNFRSF16) associated protein 1
1.14	KARS	lysyl-tRNA synthetase
1.14	IL22RA2	interleukin 22 receptor. alpha 2
1.14	BRAP	BRCA1 associated protein
1.14	LRCOL1	leucine rich colipase-like 1
1.14	RAI14	Transcript Identified by AceView. Entrez Gene ID(s) 26064
1.14	IQCB1	IQ motif containing B1
1.14	SULT1E1	sulfotransferase family 1E member 1
1.14	PIK3CD	phosphatidylinositol-4.5-bisphosphate 3-kinase. catalytic subunit delta
1.14	SRRD	SRR1 domain containing

1.14	SLC7A6	solute carrier family 7 (amino acid transporter light chain. y+L system). member 6
1.14	ZNF485	zinc finger protein 485
1.14	COL4A2	collagen. type IV. alpha 2
1.14	OR13F1	olfactory receptor. family 13. subfamily F. member 1
1.14	C14orf132	chromosome 14 open reading frame 132
1.14	ATP11A	ATPase. class VI. type 11A
1.14	COQ10B	coenzyme Q10B
1.14	TGDS	TDP-glucose 4.6-dehydratase
1.14	KDM4D	lysine (K)-specific demethylase 4D
1.14	SCARF2	scavenger receptor class F. member 2
1.14	TSEN15	TSEN15 tRNA splicing endonuclease subunit
1.14	USP11	ubiquitin specific peptidase 11
1.14	BTF3	basic transcription factor 3
1.14	H1FX	H1 histone family. member X
1.14	ZNRF2	zinc and ring finger 2. E3 ubiquitin protein ligase
1.14	NUDT19	nudix hydrolase 19
1.14	UBE2S	ubiquitin-conjugating enzyme E2S
1.14	AKR1C3	aldo-keto reductase family 1. member C3
1.14	PLEKHG7	pleckstrin homology domain containing. family G (with RhoGef domain) member 7
1.14	RPS16	ribosomal protein S16
1.14	ZNF410	zinc finger protein 410
1.14	OR6K6	olfactory receptor. family 6. subfamily K. member 6
1.14	DNTT	DNA nucleotidylexotransferase
1.14	ARMS2	age-related maculopathy susceptibility 2
1.14	DEFB109P1B	defensin. beta 109. pseudogene 1B

1.14	KBTBD2	kelch repeat and BTB (POZ) domain containing 2
1.14	TIAL1	TIA1 cytotoxic granule-associated RNA binding protein-like 1
1.14	GNA11	guanine nucleotide binding protein (G protein). alpha 11 (Gq class)
1.14	DSCR3	Down syndrome critical region 3
1.14	AAGAB	alpha- and gamma-adaptin binding protein
1.14	CLEC12A	C-type lectin domain family 12. member A
1.14	LRRC37A	leucine rich repeat containing 37A
1.14	GHITM	growth hormone inducible transmembrane protein
1.14	TPSB2; TPSAB1	tryptase beta 2 (gene/pseudogene); tryptase alpha/beta 1
1.14	HEATR5A	HEAT repeat containing 5A
1.14	TPPP3	tubulin polymerization-promoting protein family member 3
1.14	PCSK2	proprotein convertase subtilisin/kexin type 2
1.14	MOB1B	MOB kinase activator 1B
1.14	RRP1	ribosomal RNA processing 1
1.14	CCSER1	coiled-coil serine rich protein 1
1.14	LPAR3	lysophosphatidic acid receptor 3
1.14	PPP2R2D	protein phosphatase 2. regulatory subunit B. delta
1.14	C14orf105	chromosome 14 open reading frame 105
1.14	TENM1	teneurin transmembrane protein 1
1.14	LRIG1	
1.14	SBK3	SH3 domain binding kinase family. member 3
1.14	CYP21A2	cytochrome P450. family 21. subfamily A. polypeptide 2
1.14	CLEC5A	C-type lectin domain family 5. member A
1.14	SERPINB11	serpin peptidase inhibitor. clade B (ovalbumin). member 11 (gene/pseudogene)
1.14	SNRPD3	small nuclear ribonucleoprotein D3 polypeptide

1.14	BYSL	bystin-like
1.14	DEFB115	defensin. beta 115
1.14	FNBP1	formin binding protein 1
1.14	MANSC1	MANSC domain containing 1
1.14	UBE2K	ubiquitin conjugating enzyme E2K
1.14	C4B	complement component 4B (Chido blood group)
1.14	C4A	complement component 4A (Rodgers blood group)
1.14	ZNF24	zinc finger protein 24
1.14	ATF1	activating transcription factor 1
1.14	PADI2	peptidyl arginine deiminase. type II
1.14	TAAR8	trace amine associated receptor 8
1.14	ZNF34	zinc finger protein 34
1.14	NOL10	nucleolar protein 10
1.14	C19orf68	chromosome 19 open reading frame 68
1.14	TRIM27	tripartite motif containing 27
1.14	TMEM186	transmembrane protein 186
1.14	BOLL	boule homolog. RNA binding protein
1.14	TTC28	tetratricopeptide repeat domain 28
1.14	DYNLRB2	dynein. light chain. roadblock-type 2
1.14	DSP	desmoplakin
1.14	WAC	WW domain containing adaptor with coiled-coil
1.14	PRPF38B	pre-mRNA processing factor 38B
1.14	EIF3I	eukaryotic translation initiation factor 3. subunit I
1.14	UBE2J2	Transcript Identified by AceView. Entrez Gene ID(s) 118424
1.14	SEMA3F	sema domain. immunoglobulin domain (Ig). short basic domain. secreted. (semaphorin) 3F

1.14	COL1A2	Jeck2013 ANTISENSE. CDS. coding. INTERNAL. intronic. OVCODE. OVEXON best transcript NM_000089
1.14	GRK5	G protein-coupled receptor kinase 5
1.14	OR10V1	olfactory receptor. family 10. subfamily V. member 1
1.14	FAM170A	family with sequence similarity 170. member A
1.14	ADGRL3	adhesion G protein-coupled receptor L3
1.14	CCL3L3; CCL3L1	chemokine (C-C motif) ligand 3-like 3; chemokine (C-C motif) ligand 3-like 1
1.14	FEM1A	fem-1 homolog a (C. elegans)
1.14	SLC22A4	solute carrier family 22 (organic cation/zwitterion transporter). member 4
1.14	EIF4E	eukaryotic translation initiation factor 4E
1.14	PPM1B	protein phosphatase. Mg2+/Mn2+ dependent. 1B
1.14	OR1L1	olfactory receptor. family 1. subfamily L. member 1
1.14	MAP1LC3C	microtubule associated protein 1 light chain 3 gamma
1.14	MRPL3	mitochondrial ribosomal protein L3
1.14	B4GALNT3	beta-1.4-N-acetyl-galactosaminyl transferase 3
1.14	CDKN1B	cyclin-dependent kinase inhibitor 1B (p27. Kip1)
1.14	KIDINS220	Transcript Identified by AceView. Entrez Gene ID(s) 57498; 100216337
1.14	DEFB112	defensin. beta 112
1.14	AMMECR1	Alport syndrome. mental retardation. midface hypoplasia and elliptocytosis chromosomal region gene 1
1.14	S100A10	S100 calcium binding protein A10
1.14	UBR5	ubiquitin protein ligase E3 component n-recognin 5
1.14	MARCH1	membrane associated ring finger 1
1.14	LOC102724279; AC004791.2; nimure	uncharacterized LOC102724279; Transcript Identified by AceView; novel transcript
1.14	SLC17A7	solute carrier family 17 (vesicular glutamate transporter). member 7
1.14	CASKIN1	CASK interacting protein 1

1.14	SUCLG1	succinate-CoA ligase. alpha subunit
1.14	RGS8	regulator of G-protein signaling 8
1.14	DYSF	dysferlin
1.14	PYDC1	PYD (pyrin domain) containing 1
1.14	LOC101927572; AC002116.7; CLIP3	uncharacterized LOC101927572; Memczak2013 ANTISENSE. CDS. coding. INTERNAL. intronic best transcript NM_015526; novel transcript. antisense to CLIP3 and THAP8
1.14	FAM155A	Jeck2013 ALT_ACCEPTOR. ALT_DONOR. coding. INTERNAL. intronic best transcript NM_001080396
1.14	NIM1K	NIM1 serine/threonine protein kinase
1.14	PRKDC	protein kinase. DNA-activated. catalytic polypeptide
1.14	ETV7	ets variant 7
1.14	LY6G6C	lymphocyte antigen 6 complex. locus G6C
1.14	SPACA1	sperm acrosome associated 1
1.14	OR2K2	olfactory receptor. family 2. subfamily K. member 2
1.14	P4HA2	prolyl 4-hydroxylase. alpha polypeptide II
1.14	BMP8A	bone morphogenetic protein 8a
1.14	KDM3A	lysine (K)-specific demethylase 3A
1.14	ZNF44	zinc finger protein 44
1.14	PRSS54	protease. serine. 54
1.14	TFAM	transcription factor A. mitochondrial
1.14	SLCO1C1	solute carrier organic anion transporter family. member 1C1
1.14	RTP4	receptor (chemosensory) transporter protein 4
1.14	MIER1	mesoderm induction early response 1. transcriptional regulator
1.14	ARPC5L	actin related protein 2/3 complex subunit 5-like
1.14	PHAX	phosphorylated adaptor for RNA export
1.14	OR13C7	olfactory receptor. family 13. subfamily C. member 7 (gene/pseudogene)

		[Source:HGNC Symbol;Acc:HGNC:15102]
1.14	EDEM3	ER degradation enhancer. mannosidase alpha-like 3
1.14	TCF24	transcription factor 24
1.14	KIF5C	kinesin family member 5C
1.14	CDK20	cyclin-dependent kinase 20
1.14	NLRP12	NLR family. pyrin domain containing 12
1.14	RALGPS2	Ral GEF with PH domain and SH3 binding motif 2
1.14	LRIG3	leucine-rich repeats and immunoglobulin-like domains 3
1.14	SNX3	sorting nexin 3
1.14	EDA	ectodysplasin A
1.14	ILKAP	ILK associated serine/threonine phosphatase
1.14	RBPMS	RNA binding protein with multiple splicing
1.14	SLC2A8	solute carrier family 2 (facilitated glucose transporter). member 8
1.14	MARCH7	membrane associated ring finger 7
1.13	FOXL2NB	FOXL2 neighbor
1.13	MORC1	MORC family CW-type zinc finger 1
1.13	SPCS3	signal peptidase complex subunit 3
1.13	LOC100287036; AC137932.6	uncharacterized LOC100287036; novel transcript. antisense to ANKRD11
1.13	KEL	Kell blood group. metallo-endopeptidase
1.13	MORC2	MORC family CW-type zinc finger 2
1.13	MAMLD1	mastermind-like domain containing 1
1.13	TPM2	tropomyosin 2 (beta)
1.13	USF2	upstream transcription factor 2. c-fos interacting
1.13	HUS1B	HUS1 checkpoint clamp component B
1.13	BEST2	bestrophin 2
1.13	OR52B2	olfactory receptor. family 52. subfamily B. member 2

1.13	MORC3	MORC family CW-type zinc finger 3
1.13	CCR1	chemokine (C-C motif) receptor 1
1.13	FNDC1	fibronectin type III domain containing 1
1.13	SLC7A13	solute carrier family 7 (anionic amino acid transporter). member 13
1.13	GPR45	G protein-coupled receptor 45
1.13	ENOSF1	enolase superfamily member 1
1.13	GTF3C5	Zhang2013 ALT_ACCEPTOR. ALT_DONOR. coding. INTERNAL. intronic best transcript NM_001122823
1.13	UBE3D	ubiquitin protein ligase E3D
1.13	ZNF684	zinc finger protein 684
1.13	OR5AK2	olfactory receptor. family 5. subfamily AK. member 2
1.13	LRFN5	leucine rich repeat and fibronectin type III domain containing 5
1.13	FSD1	fibronectin type III and SPRY domain containing 1
1.13	FBL	fibrillarin
1.13	KRT73	keratin 73. type II
1.13	COL22A1	collagen. type XXII. alpha 1
1.13	PGA3; PGA4	pepsinogen 3. group I (pepsinogen A); pepsinogen 4. group I (pepsinogen A)
1.13	EYA1	EYA transcriptional coactivator and phosphatase 1
1.13	PTF1A	pancreas specific transcription factor. 1a
1.13	LRP6	LDL receptor related protein 6
1.13	MGA	MGA. MAX dimerization protein
1.13	ICOSLG	inducible T-cell co-stimulator ligand
1.13	C4orf26	chromosome 4 open reading frame 26
1.13	ETFB	electron-transfer-flavoprotein. beta polypeptide
1.13	PDCD2	programmed cell death 2
1.13	OR1G1	olfactory receptor. family 1. subfamily G. member 1

1.13	ZNF280B	zinc finger protein 280B
1.13	SLC16A5	solute carrier family 16 (monocarboxylate transporter). member 5
1.13	KIAA2022	KIAA2022
1.13	HIST1H1D	histone cluster 1. H1d
1.13	C3orf49	chromosome 3 open reading frame 49
1.13	RHOB	ras homolog family member B
1.13	OR5K2	olfactory receptor. family 5. subfamily K. member 2
1.13	ANKRD65	ankyrin repeat domain 65
1.13	NOVA1	neuro-oncological ventral antigen 1
1.13	FIBIN	fin bud initiation factor homolog (zebrafish)
1.13	SPERT	spermatid associated
1.13	TMEM62	transmembrane protein 62
1.13	RBM4	RNA binding motif protein 4
1.13	TREML1	triggering receptor expressed on myeloid cells-like 1
1.13	LMTK2	lemur tyrosine kinase 2
1.13	HRASLS5	HRAS-like suppressor family. member 5
1.13	ANAPC5	anaphase promoting complex subunit 5
1.13	PABPC1L2A	poly(A) binding protein. cytoplasmic 1-like 2A
1.13	VWA5A	von Willebrand factor A domain containing 5A
1.13	FASLG	Fas ligand (TNF superfamily. member 6)
1.13	ZFPM2	zinc finger protein. FOG family member 2
1.13	PET117; CSRP2BP	PET117 homolog; CSRP2 binding protein
1.13	SLC6A2	solute carrier family 6 (neurotransmitter transporter). member 2
1.13	USMG5; MIR1307	up-regulated during skeletal muscle growth 5 homolog (mouse); microRNA 1307
1.13	TRPV5	transient receptor potential cation channel. subfamily V. member 5

1.13	MTX3	metaxin 3
1.13	BMF	Bcl2 modifying factor
1.13	NPAS3	neuronal PAS domain protein 3
1.13	OAF	out at first homolog
1.13	VPS11	VPS11. CORVET/HOPS core subunit [Source:HGNC Symbol;Acc:HGNC:14583]
1.13	TEX15	testis expressed 15
1.13	ACSL1	acyl-CoA synthetase long-chain family member 1
1.13	NKAIN3	Na+/K+ transporting ATPase interacting 3
1.13	LOC286238; RP13-60M5.2	uncharacterized LOC286238; Transcript Identified by AceView. Entrez Gene ID(s) 286238. RefSeq ID(s) NM_001100111; novel transcript
1.13	DDX20	DEAD (Asp-Glu-Ala-Asp) box polypeptide 20
1.13	ANKRD12	ankyrin repeat domain 12
1.13	FDXR	ferredoxin reductase
1.13	SF1	Memczak2013 ALT_ACCEPTOR. ALT_DONOR. coding. INTERNAL. intronic best transcript NM_201998
1.13	CDY1B; CDY1	chromodomain protein. Y-linked. 1B; chromodomain protein. Y-linked. 1
1.13	LCN8	lipocalin 8
1.13	CAPZA2	capping protein (actin filament) muscle Z-line. alpha 2
1.13	RPL5; SNORD21; SNORA66	ribosomal protein L5; small nucleolar RNA. C/D box 21; small nucleolar RNA. H/ACA box 66
1.13	LOC100130357; RP1-257A7.4	uncharacterized LOC100130357; novel transcript. antisense to PHACTR1
1.13	ADAM20	ADAM metallopeptidase domain 20
1.13	NDUFAB1	NADH dehydrogenase (ubiquinone) 1. alpha/beta subcomplex. 1. 8kDa
1.13	FTMT	ferritin mitochondrial
1.13	RPS23	ribosomal protein S23
1.13	GPR148	G protein-coupled receptor 148
1.13	C8orf89	chromosome 8 open reading frame 89

1.13	CRTC3	CREB regulated transcription coactivator 3
1.13	ZDHHC3	zinc finger. DHHC-type containing 3
1.13	RAB1A	RAB1A. member RAS oncogene family
1.13	TUSC1	tumor suppressor candidate 1
1.13	C10orf53	chromosome 10 open reading frame 53
1.13	TDO2	tryptophan 2.3-dioxygenase
1.13	ZNF823	zinc finger protein 823
1.13	HKR1	HKR1. GLI-Kruppel zinc finger family member
1.13	GPRC6A	G protein-coupled receptor. class C. group 6. member A
1.13	OR2A14; OR2A2	olfactory receptor. family 2. subfamily A. member 14; olfactory receptor. family 2. subfamily A. member 2
1.13	LIX1	Memczak2013 ANTISENSE. coding. INTERNAL. intronic best transcript NM_153234
1.13	RUVBL1	RuvB-like AAA ATPase 1
1.13	PAOX	polyamine oxidase (exo-N4-amino)
1.13	RAD51B	RAD51 paralog B
1.13	ADARB1	adenosine deaminase. RNA-specific. B1
1.13	PPP1R12C	protein phosphatase 1. regulatory subunit 12C
1.13	LRP2	LDL receptor related protein 2
1.13	LYAR	Ly1 antibody reactive
1.13	LAMB3; MIR4260	laminin. beta 3; microRNA 4260
1.13	PLEKHG4	pleckstrin homology domain containing. family G (with RhoGef domain) member 4
1.13	KRBA1	KRAB-A domain containing 1
1.13	MLXIP	MLX interacting protein
1.13	DZANK1	double zinc ribbon and ankyrin repeat domains 1
1.13	MYH14	myosin. heavy chain 14. non-muscle

1.13	TMEM33	transmembrane protein 33
1.13	BZW1	basic leucine zipper and W2 domains 1
1.13	ABRACL	ABRA C-terminal like
1.13	GAR1	GAR1 homolog. ribonucleoprotein
1.13	EHD4	EH domain containing 4
1.13	COL6A2	collagen. type VI. alpha 2
1.13	PSMG4	proteasome (prosome. macropain) assembly chaperone 4
1.13	SLC25A17	solute carrier family 25 (mitochondrial carrier; peroxisomal membrane protein. 34kDa). member 17
1.13	USP17L26; USP17L24	ubiquitin specific peptidase 17-like family member 26; ubiquitin specific peptidase 17-like family member 24
1.13	ZNF780B	zinc finger protein 780B
1.13	TRANK1	tetratricopeptide repeat and ankyrin repeat containing 1
1.13	TRIM56	tripartite motif containing 56
1.13	HERC2P3	hect domain and RLD 2 pseudogene 3
1.13	HECTD4; MIR6861	HECT domain containing E3 ubiquitin protein ligase 4; microRNA 6861
1.13	NRARP	NOTCH-regulated ankyrin repeat protein
1.13	HARS2	histidyl-tRNA synthetase 2. mitochondrial
1.13	INA	internexin neuronal intermediate filament protein. alpha
1.13	OXGR1	oxoglutarate (alpha-ketoglutarate) receptor 1
1.13	PTPRG	Jeck2013 ALT_ACCEPTOR. ALT_DONOR. coding. INTERNAL. intronic best transcript NM_002841
1.13	EXOC3L4	exocyst complex component 3-like 4
1.13	POLR1B	polymerase (RNA) I polypeptide B
1.13	TOPAZ1	testis and ovary specific PAZ domain containing 1
1.13	INPP5D	inositol polyphosphate-5-phosphatase D
1.13	SF1	splicing factor 1

1.13	ISL2	ISL LIM homeobox 2
1.13	ZNF277	zinc finger protein 277
1.13	RPL23; SNORA21	ribosomal protein L23; small nucleolar RNA. H/ACA box 21
1.13	FOXD1	forkhead box D1
1.13	PNPLA7	Transcript Identified by AceView. Entrez Gene ID(s) 375775
1.13	CCNB3	cyclin B3
1.13	BTBD7	BTB (POZ) domain containing 7
1.13	OR4P4	olfactory receptor. family 4. subfamily P. member 4
1.13	C16orf47	chromosome 16 open reading frame 47
1.13	MTMR6	Transcript Identified by AceView. Entrez Gene ID(s) 9107
1.13	PTPRC	protein tyrosine phosphatase. receptor type. C
1.13	DUPD1	dual specificity phosphatase and pro isomerase domain containing 1
1.13	CCL22	chemokine (C-C motif) ligand 22
1.13	NGB	neuroglobin
1.13	ZSCAN21	zinc finger and SCAN domain containing 21
1.13	FAM166B	family with sequence similarity 166. member B
1.13	C16orf86	chromosome 16 open reading frame 86
1.13	PLEKHB2	pleckstrin homology domain containing. family B (evectins) member 2
1.13	NDUFAF6	NADH dehydrogenase (ubiquinone) complex I. assembly factor 6
1.13	ATG16L1	autophagy related 16-like 1
1.13	WIF1	WNT inhibitory factor 1
1.13	GOT1L1	glutamic-oxaloacetic transaminase 1-like 1
1.13	CAPN14	calpain 14
1.13	ADAL	adenosine deaminase-like
1.13	SLC19A3	solute carrier family 19 (thiamine transporter). member 3
1.13	LCE3C	late cornified envelope 3C

1.13	NCKAP1L	NCK-associated protein 1-like
1.13	ABCA2	ATP binding cassette subfamily A member 2
1.13	FAM53A	family with sequence similarity 53. member A
1.13	SPINK9	serine peptidase inhibitor. Kazal type 9
1.13	MRGPRX1	MAS-related GPR. member X1
1.13	TMPRSS3	transmembrane protease. serine 3
1.13	MUC21	mucin 21. cell surface associated
1.13	RPS10-NUDT3	RPS10-NUDT3 readthrough
1.13	FIGN	fidgetin
1.13	SSBP1	single-stranded DNA binding protein 1. mitochondrial
1.13	KLHL28	kelch-like family member 28
1.13	MATK	megakaryocyte-associated tyrosine kinase
1.13	OR10J4	olfactory receptor. family 10. subfamily J. member 4 (gene/pseudogene) [Source:HGNC Symbol;Acc:HGNC:15408]
1.13	CCDC83	coiled-coil domain containing 83
1.13	ETV1	ets variant 1
1.13	LRRC25	leucine rich repeat containing 25
1.13	LRIF1	ligand dependent nuclear receptor interacting factor 1
1.13	KRT3	keratin 3. type II
1.13	EAF1	ELL associated factor 1
1.13	ACTL9	actin-like 9
1.13	CDR2	cerebellar degeneration related protein 2
1.13	RXRG	retinoid X receptor gamma
1.13	CAPS2	calcyphosine 2
1.13	HRNR	hornerin
1.13	USP17L28; USP17L5; USP17L24	ubiquitin specific peptidase 17-like family member 28; ubiquitin specific peptidase 17-like family member 5; ubiquitin specific peptidase 17-like

		family member 24
1.13	OR1S2	olfactory receptor. family 1. subfamily S. member 2
1.13	EBPL	emopamil binding protein-like
1.13	HORMAD1	HORMA domain containing 1
1.13	TFAP2E	transcription factor AP-2 epsilon (activating enhancer binding protein 2 epsilon)
1.13	TMEM75	transmembrane protein 75
1.13	ITGA9	integrin alpha 9
1.13	SCG2	secretogranin II
1.13	NDFIP1	Nedd4 family interacting protein 1
1.13	MRPS30	mitochondrial ribosomal protein S30
1.13	CTNNB1	catenin (cadherin-associated protein). beta 1
1.13	CCDC148	coiled-coil domain containing 148
1.13	GALE	UDP-galactose-4-epimerase
1.13	NAB1	NGFI-A binding protein 1
1.13	VSX2	visual system homeobox 2
1.13	FMNL3	formin like 3
1.13	ST3GAL5	ST3 beta-galactoside alpha-2.3-sialyltransferase 5
1.13	UGT3A1	UDP glycosyltransferase 3 family. polypeptide A1
1.13	IRF9	interferon regulatory factor 9
1.13	BRPF1	bromodomain and PHD finger containing 1
1.13	CDC42SE1	CDC42 small effector 1
1.13	C20orf194	chromosome 20 open reading frame 194
1.13	PMEPA1	prostate transmembrane protein. androgen induced 1
1.13	TAL2	T-cell acute lymphocytic leukemia 2
1.13	HRK	harakiri. BCL2 interacting protein

1.13	KCNAB1	Transcript Identified by AceView. Entrez Gene ID(s) 7881
1.13	TBCCD1	TBCC domain containing 1
1.13	POTEH	POTE ankyrin domain family. member H
1.13	GRIN3A	glutamate receptor. ionotropic. N-methyl-D-aspartate 3A
1.13	KPNA6	karyopherin alpha 6 (importin alpha 7)
1.13	OR1S1	olfactory receptor. family 1. subfamily S. member 1 (gene/pseudogene)
1.13	OLIG1	oligodendrocyte transcription factor 1
1.13	SH3TC2	SH3 domain and tetratricopeptide repeats 2
1.13	TRAPPC13	trafficking protein particle complex 13
1.13	MROH6	maestro heat-like repeat family member 6
1.13	TGM4	transglutaminase 4
1.13	DCAF16	DDB1 and CUL4 associated factor 16
1.13	NAA20	N(alpha)-acetyltransferase 20. NatB catalytic subunit
1.13	TBX22	T-box 22
1.13	BMP1	bone morphogenetic protein 1
1.13	HMBS	hydroxymethylbilane synthase
1.13	GON4L	gon-4-like (C. elegans)
1.13	ZZZ3	zinc finger. ZZ-type containing 3
1.13	KCNJ10	potassium channel. inwardly rectifying subfamily J. member 10
1.13	STAG2	stromal antigen 2
1.13	CXorf65	chromosome X open reading frame 65
1.13	ISLR2	immunoglobulin superfamily containing leucine-rich repeat 2
1.13	SSX4B; SSX4	synovial sarcoma. X breakpoint 4B; synovial sarcoma. X breakpoint 4
1.13	DUSP7	dual specificity phosphatase 7
1.13	C1orf52	chromosome 1 open reading frame 52
1.13	TRIM21	tripartite motif containing 21

1.13	CELA2B	chymotrypsin-like elastase family. member 2B
1.13	FYN	FYN proto-oncogene. Src family tyrosine kinase
1.13	GPR26	G protein-coupled receptor 26
1.13	OR4F4; OR4F17	olfactory receptor. family 4. subfamily F. member 4; olfactory receptor. family 4. subfamily F. member 17
1.13	NBN	Transcript Identified by AceView. Entrez Gene ID(s) 4683
1.13	CYLD	cylindromatosis (turban tumor syndrome)
1.13	ZNF501	zinc finger protein 501 [Source:HGNC Symbol;Acc:HGNC:23717]
1.13	TCEAL6	transcription elongation factor A (SII)-like 6
1.13	KRTAP19-8	keratin associated protein 19-8
1.13	EVPLL	envoplakin-like
1.13	TMX4	Memczak2013 ALT_ACCEPTOR. ALT_DONOR. coding. INTERNAL. intronic best transcript NM_021156
1.13	RBM39	RNA binding motif protein 39
1.13	TAF3	TATA box binding protein associated factor 3
1.13	RBFOX3	RNA binding protein. fox-1 homolog (C. elegans) 3
1.13	VCPIP1	valosin containing protein (p97)/p47 complex interacting protein 1
1.13	NMNAT2	nicotinamide nucleotide adenylyltransferase 2
1.13	AP3M2	adaptor-related protein complex 3. mu 2 subunit
1.13	IL20RA	interleukin 20 receptor. alpha
1.13	UCHL5	ubiquitin C-terminal hydrolase L5
1.13	HEBP1	heme binding protein 1
1.13	CALCR	calcitonin receptor
1.13	MEI4	meiotic double-stranded break formation protein 4
1.13	PHYKPL	5-phosphohydroxy-L-lysine phospho-lyase
1.13	SH3GL3	SH3-domain GRB2-like 3
1.13	SCN1B	sodium channel. voltage gated. type I beta subunit

1.13	ZBED4	zinc finger. BED-type containing 4
1.13	KRTAP20-4	keratin associated protein 20-4
1.13	PLPP6	phospholipid phosphatase 6
1.13	PASD1	PAS domain containing 1
1.13	TNFRSF8	tumor necrosis factor receptor superfamily. member 8
1.13	BRF2	BRF2. RNA polymerase III transcription initiation factor 50 kDa subunit
1.13	PPIC	peptidylprolyl isomerase C (cyclophilin C)
1.13	DCANP1	dendritic cell-associated nuclear protein
1.13	CXCL8	chemokine (C-X-C motif) ligand 8
1.13	TRIML2	tripartite motif family like 2
1.13	TWF1	twinfilin actin binding protein 1
1.13	IZUMO1R	IZUMO1 receptor. JUNO
1.13	RPL3; SNORD83B; SNORD43	ribosomal protein L3; small nucleolar RNA. C/D box 83B; small nucleolar RNA. C/D box 43
1.13	HSFY2; HSFY1	heat shock transcription factor. Y-linked 2; heat shock transcription factor. Y-linked 1
1.13	SYT2	synaptotagmin II
1.13	ZNF860	zinc finger protein 860
1.13	KRT28	keratin 28. type I
1.13	LRGUK	leucine-rich repeats and guanylate kinase domain containing
1.13	EMC1	ER membrane protein complex subunit 1
1.13	NT5C1A	5-nucleotidase. cytosolic IA
1.13	LCORL	ligand dependent nuclear receptor corepressor like
1.13	CCBE1	Jeck2013 ALT_ACCEPTOR. ALT_DONOR. coding. INTERNAL. intronic best transcript NM_133459
1.13	TAPT1	transmembrane anterior posterior transformation 1
1.13	MYPOP	Myb-related transcription factor. partner of profilin

1.13	MIPOL1	mirror-image polydactyly 1
1.13	FUBP1	far upstream element (FUSE) binding protein 1
1.13	RPL7A	ribosomal protein L7a
1.13	RFT1	RFT1 homolog
1.13	CLEC4C	C-type lectin domain family 4. member C
1.13	TMC1	transmembrane channel like 1
1.13	OR8D2	olfactory receptor. family 8. subfamily D. member 2 (gene/pseudogene)
1.13	TIE1	tyrosine kinase with immunoglobulin-like and EGF-like domains 1
1.13	CWF19L1; SNORA12	CWF19-like 1. cell cycle control (S. pombe); small nucleolar RNA. H/ACA box 12
1.13	C11orf94	chromosome 11 open reading frame 94
1.13	ARHGEF6	Rac/Cdc42 guanine nucleotide exchange factor 6
1.13	OTOS	otospiralin
1.13	STX16-NPEPL1	STX16-NPEPL1 readthrough (NMD candidate)
1.13	RPS6	ribosomal protein S6
1.13	OR52N5	olfactory receptor. family 52. subfamily N. member 5
1.12	LOC102724957; RP11-396O20.1	uncharacterized LOC102724957; novel transcript
1.12	NFATC3	nuclear factor of activated T-cells. cytoplasmic. calcineurin-dependent 3
1.12	GBX2	gastrulation brain homeobox 2
1.12	CELF4	CUGBP. Elav-like family member 4
1.12	MARVELD2	Transcript Identified by AceView. Entrez Gene ID(s) 153562
1.12	CRABP2	cellular retinoic acid binding protein 2
1.12	SERBP1	SERPINE1 mRNA binding protein 1
1.12	UHMK1	U2AF homology motif (UHM) kinase 1
1.12	NPR3	natriuretic peptide receptor 3
1.12	FGFR1OP2	FGFR1 oncogene partner 2

1.12	TP63	tumor protein p63
1.12	ASCL1	achaete-scute family bHLH transcription factor 1
1.12	COPS8	COP9 signalosome subunit 8
1.12	CYB561D1	cytochrome b561 family. member D1
1.12	PDCD4; MIR4680	programmed cell death 4 (neoplastic transformation inhibitor); microRNA 4680
1.12	TMEM14EP	transmembrane protein 14E. pseudogene
1.12	CSF3	colony stimulating factor 3
1.12	MS4A10	membrane-spanning 4-domains. subfamily A. member 10
1.12	OR7G2	olfactory receptor. family 7. subfamily G. member 2
1.12	GLTSCR2; SNORD23	glioma tumor suppressor candidate region gene 2; small nucleolar RNA. C/D box 23
1.12	ZNF786	zinc finger protein 786
1.12	SHH	sonic hedgehog
1.12	RANBP1	Zhang2013 ALT_ACCEPTOR. ALT_DONOR. coding. INTERNAL. intronic best transcript NM_002882
1.12	TAS2R60	taste receptor. type 2. member 60
1.12	OSTM1	osteopetrosis associated transmembrane protein 1
1.12	TBC1D27	TBC1 domain family. member 27
1.12	FBXL16	F-box and leucine-rich repeat protein 16
1.12	CHST14	carbohydrate (N-acetylgalactosamine 4-0) sulfotransferase 14
1.12	RPL7	ribosomal protein L7
1.12	HES3	hes family bHLH transcription factor 3
1.12	FAM205A	family with sequence similarity 205. member A
1.12	SH3BP5	SH3-domain binding protein 5 (BTK-associated)
1.12	KCNK13	potassium channel. two pore domain subfamily K. member 13
1.12	ZNF568	zinc finger protein 568

1.12	GOLGA8J	golgin A8 family. member J
1.12	CELA3A	chymotrypsin-like elastase family. member 3A
1.12	C11orf88	chromosome 11 open reading frame 88
1.12	TINAG	tubulointerstitial nephritis antigen
1.12	KIF4B	kinesin family member 4B
1.12	NDUFA10	NADH dehydrogenase (ubiquinone) 1 alpha subcomplex. 10. 42kDa
1.12	GUCY1A3	guanylate cyclase 1. soluble. alpha 3
1.12	THADA	thyroid adenoma associated
1.12	SYDE2	synapse defective 1. Rho GTPase. homolog 2 (C. elegans)
1.12	ACOT11	acyl-CoA thioesterase 11
1.12	NPM1	nucleophosmin (nucleolar phosphoprotein B23. numatrin)
1.12	OR8A1	olfactory receptor. family 8. subfamily A. member 1
1.12	TMEM41B	transmembrane protein 41B
1.12	FECH	ferrochelatase
1.12	NPIPB9	nuclear pore complex interacting protein family. member B9
1.12	SDR9C7	short chain dehydrogenase/reductase family 9C. member 7
1.12	VNN3	vanin 3
1.12	ZNF577	zinc finger protein 577
1.12	HSP90AB1	heat shock protein 90kDa alpha (cytosolic). class B member 1
1.12	FGF9	fibroblast growth factor 9
1.12	GNB1	guanine nucleotide binding protein (G protein). beta polypeptide 1
1.12	SCARB2	scavenger receptor class B. member 2
1.12	A3GALT2	alpha 1.3-galactosyltransferase 2
1.12	ADCYAP1	adenylate cyclase activating polypeptide 1 (pituitary)
1.12	TRIM58	tripartite motif containing 58
1.12	ZNF710	zinc finger protein 710

1.12	LINC00303	long intergenic non-protein coding RNA 303
1.12	CHIT1	chitinase 1 (chitotriosidase)
1.12	OTP	orthopedia homeobox
1.12	RPS14	ribosomal protein S14
1.12	WDR27	WD repeat domain 27
1.12	UBE2J2	ubiquitin-conjugating enzyme E2. J2
1.12	KIAA1522	KIAA1522
1.12	PTCH1	patched 1
1.12	LAD1	ladinin 1
1.12	PAX8	paired box 8
1.12	ENHO	energy homeostasis associated
1.12	RPS28	ribosomal protein S28
1.12	VNN2	vanin 2
1.12	ZKSCAN2	zinc finger with KRAB and SCAN domains 2
1.12	DPPA5	developmental pluripotency associated 5
1.12	TNFRSF10D	tumor necrosis factor receptor superfamily. member 10d. decoy with truncated death domain
1.12	IFT88	intraflagellar transport 88
1.12	SPINK6	serine peptidase inhibitor. Kazal type 6
1.12	VWA2	von Willebrand factor A domain containing 2
1.12	METTL6	methyltransferase like 6
1.12	EEF1G; MIR3654	eukaryotic translation elongation factor 1 gamma; microRNA 3654
1.12	PRODH; LOC102724788; AC007325.2	Homo sapiens proline dehydrogenase (oxidase) 1 (PRODH). transcript variant 2. mRNA.; Homo sapiens proline dehydrogenase (oxidase) 1 (PRODH). transcript variant 1. mRNA.; proline dehydrogenase 1. mitochondrial; Homo sapiens proline dehydrogenase (oxidase) 1. mRNA (cDNA clone MGC:148079 IMAGE:40108133). complete cds.
1.12	ADRA1A	adrenoceptor alpha 1A

1.12	PRPF19	pre-mRNA processing factor 19
1.12	PRAMEF4	PRAME family member 4
1.12	RUFY2	RUN and FYVE domain containing 2
1.12	DSG1	desmoglein 1
1.12	DDAH1	dimethylarginine dimethylaminohydrolase 1
1.12	TYW5	tRNA-yW synthesizing protein 5
1.12	DPYSL3	dihydropyrimidinase-like 3
1.12	TTC38	Memczak2013 ALT_ACCEPTOR. ALT_DONOR. coding. INTERNAL. intronic best transcript NM_017931
1.12	TP53BP2	tumor protein p53 binding protein 2
1.12	MS4A12	membrane-spanning 4-domains. subfamily A. member 12
1.12	NIT2	nitrilase family. member 2
1.12	TTLL9	tubulin tyrosine ligase-like family member 9
1.12	NBPF6; NBPF5P	neuroblastoma breakpoint family. member 6; neuroblastoma breakpoint family. member 5. pseudogene
1.12	DCUN1D1	DCN1. defective in cullin neddylation 1. domain containing 1
1.12	GRIP2	glutamate receptor interacting protein 2
1.12	SLC37A1	solute carrier family 37 (glucose-6-phosphate transporter). member 1
1.12	CREBBP	CREB binding protein
1.12	SNRNP40	small nuclear ribonucleoprotein. U5 40kDa subunit
1.12	TACC2	transforming. acidic coiled-coil containing protein 2
1.12	MFSD10	major facilitator superfamily domain containing 10
1.12	HIST1H2AC	histone cluster 1. H2ac
1.12	ACOT4	acyl-CoA thioesterase 4
1.12	EDIL3	EGF-like repeats and discoidin I-like domains 3
1.12	ARIH1; MIR630	ariadne RBR E3 ubiquitin protein ligase 1; microRNA 630
1.12	CHCHD3	coiled-coil-helix-coiled-coil-helix domain containing 3

1.12	ZNF227	zinc finger protein 227
1.12	MRPL19	mitochondrial ribosomal protein L19
1.12	CPE	carboxypeptidase E
1.12	OBP2A	odorant binding protein 2A
1.12	DSG3	desmoglein 3
1.12	NOP56; SNORD57; SNORD56; SNORA51; SNORD110; SNORD86; MIR1292	NOP56 ribonucleoprotein; small nucleolar RNA. C/D box 57; small nucleolar RNA. C/D box 56; small nucleolar RNA. H/ACA box 51; small nucleolar RNA. C/D box 110; small nucleolar RNA. C/D box 86; microRNA 1292
1.12	ARMC2	Memczak2013 ANTISENSE. coding. INTERNAL. intronic best transcript NM_032131
1.12	MAGEB3	MAGE family member B3
1.12	MDP1; NEDD8-MDP1; NEDD8; CHMP4A	magnesium-dependent phosphatase 1; NEDD8-MDP1 readthrough; neural precursor cell expressed. developmentally down-regulated 8; charged multivesicular body protein 4A
1.12	PHACTR1	phosphatase and actin regulator 1
1.12	TADA2B	Transcript Identified by AceView. Entrez Gene ID(s) 93624
1.12	ZNF566	zinc finger protein 566
1.12	SOWAHA	sosondowah ankyrin repeat domain family member A
1.12	DGKK	diacylglycerol kinase. kappa
1.12	CYTH4	cytohesin 4
1.12	SLC10A2	solute carrier family 10 (sodium/bile acid cotransporter). member 2
1.12	MID2	midline 2
1.12	NPIPB11	nuclear pore complex interacting protein family. member B11
1.12	VPS11	VPS11. CORVET/HOPS core subunit [Source:HGNC Symbol;Acc:HGNC:14583]
1.12	PTDSS2	phosphatidylserine synthase 2
1.12	RDH5	retinol dehydrogenase 5 (11-cis/9-cis)
1.12	DDX50	DEAD (Asp-Glu-Ala-Asp) box polypeptide 50
1.12	GORASP1	golgi reassembly stacking protein 1

1.12	UBE2A	ubiquitin conjugating enzyme E2A
1.12	PIGO	phosphatidylinositol glycan anchor biosynthesis class O
1.12	RNPEPL1	arginyl aminopeptidase (aminopeptidase B)-like 1
1.12	OR51H1	olfactory receptor. family 51. subfamily H. member 1
1.12	MSANTD2	Myb/SANT-like DNA-binding domain containing 2
1.12	CATSPER1	cation channel. sperm associated 1
1.12	ACTR5	ARP5 actin-related protein 5 homolog (yeast)
1.12	SAG	S-antigen; retina and pineal gland (arrestin)
1.12	NLRP7	NLR family. pyrin domain containing 7
1.12	GALNT12	polypeptide N-acetylgalactosaminyltransferase 12
1.12	ASIP	agouti signaling protein
1.12	PRR23D2; PRR23D1	proline rich 23 domain containing 2; proline rich 23 domain containing 1
1.12	OR1L3	olfactory receptor. family 1. subfamily L. member 3
1.12	UTP11L	UTP11-like. U3 small nucleolar ribonucleoprotein (yeast)
1.12	WBSCR22	Williams Beuren syndrome chromosome region 22
1.12	CORO1C	coronin. actin binding protein. 1C
1.12	NYX	nyctalopin
1.12	P2RY4	pyrimidinergic receptor P2Y. G-protein coupled. 4
1.12	SKP2	Transcript Identified by AceView. Entrez Gene ID(s) 6502
1.12	FXR2	fragile X mental retardation. autosomal homolog 2
1.12	GALNT8	polypeptide N-acetylgalactosaminyltransferase 8
1.12	B4GALT5	UDP-Gal:betaGlcNAc beta 1.4- galactosyltransferase. polypeptide 5
1.12	GFM1	G elongation factor. mitochondrial 1
1.12	TBC1D10C	TBC1 domain family. member 10C
1.12	VSTM2B	V-set and transmembrane domain containing 2B
1.12	FAM134B	family with sequence similarity 134. member B

1.12	C17orf80	chromosome 17 open reading frame 80
1.12	TBC1D32	TBC1 domain family. member 32
1.12	GOLGA6B	golgin A6 family. member B
1.12	NSL1	NSL1. MIS12 kinetochore complex component
1.12	APOBEC3A	apolipoprotein B mRNA editing enzyme. catalytic polypeptide-like 3A
1.12	TANC1	tetratricopeptide repeat. ankyrin repeat and coiled-coil containing 1
1.12	ZMAT2	zinc finger. matrin-type 2
1.12	OR8S1	olfactory receptor. family 8. subfamily S. member 1
1.12	CHCHD6	coiled-coil-helix-coiled-coil-helix domain containing 6
1.12	AGAP2	ArfGAP with GTPase domain. ankyrin repeat and PH domain 2
1.12	PSG3	pregnancy specific beta-1-glycoprotein 3
1.12	TIMM23B	translocase of inner mitochondrial membrane 23 homolog B (yeast)
1.12	KMT2E	lysine (K)-specific methyltransferase 2E
1.12	AANAT	aralkylamine N-acetyltransferase
1.12	IFNK	interferon. kappa
1.12	LRRTM3	leucine rich repeat transmembrane neuronal 3
1.12	SLC9A9	solute carrier family 9. subfamily A (NHE9. cation proton antiporter 9). member 9
1.12	ZNF35	zinc finger protein 35
1.12	FBXO24	F-box protein 24
1.12	TRAF5	TNF receptor-associated factor 5
1.12	SH2D4A	Transcript Identified by AceView. Entrez Gene ID(s) 63898
1.12	RASA3	RAS p21 protein activator 3
1.12	ZDHHC17	zinc finger. DHHC-type containing 17
1.12	SMARCAD1	SWI/SNF-related. matrix-associated actin-dependent regulator of chromatin. subfamily a. containing DEAD/H box 1
1.12	C12orf74	chromosome 12 open reading frame 74

1.12	HAL	histidine ammonia-lyase
1.12	SAMD14	sterile alpha motif domain containing 14
1.12	SGF29	SAGA complex associated factor 29
1.12	GPR151	G protein-coupled receptor 151
1.12	MAPKAPK3	mitogen-activated protein kinase-activated protein kinase 3
1.12	TAF1A	TATA box binding protein (TBP)-associated factor. RNA polymerase I. A. 48kDa
1.12	TMEM120B	transmembrane protein 120B
1.12	PIAS2	protein inhibitor of activated STAT 2
1.12	CT45A8	cancer/testis antigen family 45. member A8
1.12	CT45A9	cancer/testis antigen family 45. member A9
1.12	TNRC6C	trinucleotide repeat containing 6C
1.12	CECR1	cat eye syndrome chromosome region. candidate 1
1.12	RFPL1	ret finger protein-like 1
1.12	ZBTB48	zinc finger and BTB domain containing 48
1.12	TRAPPC4	trafficking protein particle complex 4
1.12	PISD; MIR7109	phosphatidylserine decarboxylase; microRNA 7109
1.12	FAM43A	family with sequence similarity 43. member A
1.12	PCDH17	protocadherin 17
1.12	SLC22A5	solute carrier family 22 (organic cation/carnitine transporter). member 5
1.12	PHF24	PHD finger protein 24
1.12	CXXC4	CXXC finger protein 4
1.12	TRAF2	TNF receptor-associated factor 2
1.12	THSD4	thrombospondin type 1 domain containing 4
1.12	HPS1; MIR4685	Hermansky-Pudlak syndrome 1; microRNA 4685
1.12	WDR17	WD repeat domain 17

1.12	SMARCC1	SWI/SNF related. matrix associated. actin dependent regulator of chromatin. subfamily c. member 1
1.12	MED21	mediator complex subunit 21
1.12	AKT1S1	Memczak2013 ANTISENSE. coding. INTERNAL. intronic best transcript NM_001098633
1.12	LOC105371242; AC243756.1	peptidyl-prolyl cis-trans isomerase A-like 4G; Peptidyl-prolyl cis-trans isomerase [Source:UniProtKB/TrEMBL;Acc:A0A075B767]
1.12	JADE1	jade family PHD finger 1
1.12	S100G	S100 calcium binding protein G
1.12	RAI2	retinoic acid induced 2
1.12	SUSD1	sushi domain containing 1
1.12	TDRD6	tudor domain containing 6
1.12	CSNK2A3	casein kinase 2. alpha 3 polypeptide
1.12	RRP12	ribosomal RNA processing 12 homolog
1.12	SEC31B	SEC31 homolog B. COPII coat complex component
1.12	CLEC4A	C-type lectin domain family 4. member A
1.12	SPIN3	spindlin family. member 3
1.12	PARK7	parkinson protein 7
1.12	STX1A	syntaxin 1A (brain)
1.12	SHFM1	split hand/foot malformation (ectrodactyly) type 1
1.12	DUSP15	dual specificity phosphatase 15
1.12	NOTCH3	notch 3
1.12	ZNF276	zinc finger protein 276
1.12	SLC35A4	solute carrier family 35. member A4
1.12	TBC1D23	TBC1 domain family. member 23
1.12	ZNF800	zinc finger protein 800
1.12	RUNX2	runt-related transcription factor 2

1.12	TDRD9	tudor domain containing 9
1.12	HDAC1	histone deacetylase 1
1.12	ATP11A	Transcript Identified by AceView. Entrez Gene ID(s) 23250
1.12	ANO10	anoctamin 10
1.12	LARP4	La ribonucleoprotein domain family. member 4
1.12	FAM207A	family with sequence similarity 207. member A
1.12	FILIP1	filamin A interacting protein 1
1.12	KLRC3	killer cell lectin-like receptor subfamily C. member 3
1.12	XAGE1E; XAGE1B	X antigen family. member 1E; X antigen family. member 1B
1.12	SPATA19	spermatogenesis associated 19
1.12	SAMM50	SAMM50 sorting and assembly machinery component
1.12	NOP58	NOP58 ribonucleoprotein
1.12	XKRY2; XKRY	X-linked Kx blood group related. Y-linked 2; X-linked Kx blood group related. Y-linked
1.12	SELP	selectin P (granule membrane protein 140kDa. antigen CD62)
1.12	KCNQ5	potassium channel. voltage gated KQT-like subfamily Q. member 5
1.12	PIGY; PYURF	phosphatidylinositol glycan anchor biosynthesis class Y; PIGY upstream reading frame
1.12	SLFN14	schlafen family member 14
1.12	FCGR1B	Fc fragment of IgG. high affinity Ib. receptor (CD64)
1.12	OR6B1	olfactory receptor. family 6. subfamily B. member 1
1.12	EIF5AL1	eukaryotic translation initiation factor 5A-like 1
1.12	TGFB2; TGFB2-OT1	transforming growth factor beta 2; TGFB2 overlapping transcript 1
1.12	ST20-MTHFS	ST20-MTHFS readthrough
1.12	ERC2	ELKS/RAB6-interacting/CAST family member 2
1.12	VPS45	vacuolar protein sorting 45 homolog (S. cerevisiae)
1.12	OR5P3	olfactory receptor. family 5. subfamily P. member 3

1.12	OASL	2-5-oligoadenylate synthetase-like
1.12	MIXL1	Mix paired-like homeobox
1.12	KRT36	keratin 36. type I
1.12	WFDC11	WAP four-disulfide core domain 11
1.12	ILDR2	immunoglobulin-like domain containing receptor 2
1.12	KIR3DX1	killer cell immunoglobulin-like receptor. three domains. X1
1.12	NFASC	neurofascin
1.12	SLPI	secretory leukocyte peptidase inhibitor
1.12	FAM186B	family with sequence similarity 186. member B
1.12	PIWIL4	piwi-like RNA-mediated gene silencing 4
1.12	NFXL1	nuclear transcription factor. X-box binding-like 1
1.12	NRXN3	neurexin 3
1.12	SNX14	sorting nexin 14
1.12	PTX3	pentraxin 3. long
1.12	C14orf169	chromosome 14 open reading frame 169
1.12	WDR3	WD repeat domain 3
1.12	KDELC1	KDEL (Lys-Asp-Glu-Leu) containing 1
1.12	ARRB1	arrestin. beta 1
1.12	RIN3	Ras and Rab interactor 3
1.12	NRP1	Transcript Identified by AceView. Entrez Gene ID(s) 8829
1.12	CABP4	calcium binding protein 4
1.12	MUC2	mucin 2. oligomeric mucus/gel-forming
1.12	ABI1	abl-interactor 1
1.12	PGPEP1L	pyroglutamyl-peptidase I-like
1.12	COL6A5	collagen. type VI. alpha 5
1.12	TOX2	Memczak2013 ALT_ACCEPTOR. ALT_DONOR. coding. INTERNAL. intronic

		best transcript NM_001098797
1.12	CASP7	caspase 7
1.12	PKNOX1	PBX/knotted 1 homeobox 1
1.12	PRR23D2	proline rich 23 domain containing 2
1.12	KLK7	kallikrein related peptidase 7
1.12	OR5M1	olfactory receptor. family 5. subfamily M. member 1
1.12	OR2H2	olfactory receptor. family 2. subfamily H. member 2
1.12	PRKRIR	protein-kinase. interferon-inducible double stranded RNA dependent inhibitor. repressor of (P58 repressor)
1.12	HTR5A-AS1	HTR5A antisense RNA 1
1.12	ZC3HAV1L	zinc finger CCCH-type. antiviral 1-like
1.12	SAMD3	sterile alpha motif domain containing 3
1.12	PRRC2B	proline-rich coiled-coil 2B
1.12	CSDE1	cold shock domain containing E1. RNA binding
1.12	SDAD1	SDA1 domain containing 1
1.12	EPS8L2	EPS8-like 2
1.12	TECRL	trans-2.3-enoyl-CoA reductase-like
1.12	ABCC9	Transcript Identified by AceView. Entrez Gene ID(s) 10060
1.12	MRPS25	mitochondrial ribosomal protein S25
1.12	ZNF17	zinc finger protein 17
1.12	CNIH3	cornichon family AMPA receptor auxiliary protein 3
1.12	ENO1	Memczak2013 ANTISENSE. coding. INTERNAL. UTR3 best transcript NM_001428
1.12	ASB13	ankyrin repeat and SOCS box containing 13
1.12	IL22	interleukin 22
1.12	PROS1	protein S (alpha)
1.12	AFF3	Memczak2013 ALT_ACCEPTOR. ALT_DONOR. coding. INTERNAL. intronic

		best transcript NM_001025108
1.12	AGR3	anterior gradient 3. protein disulphide isomerase family member
1.12	C1orf234	chromosome 1 open reading frame 234
1.12	CCDC136	coiled-coil domain containing 136
1.12	NIPA1	non imprinted in Prader-Willi/Angelman syndrome 1
1.12	RTCB	RNA 2.3-cyclic phosphate and 5-OH ligase
1.12	PHOSPHO1	phosphatase. orphan 1
1.12	NTRK2	neurotrophic tyrosine kinase. receptor. type 2
1.12	SLC14A1	solute carrier family 14 (urea transporter). member 1 (Kidd blood group)
1.11	SMG1	SMG1 phosphatidylinositol 3-kinase-related kinase
1.11	WNT8A	wingless-type MMTV integration site family. member 8A
1.11	DDHD1	DDHD domain containing 1
1.11	TDRD5	tudor domain containing 5
1.11	CCDC53	coiled-coil domain containing 53
1.11	AZIN1	antizyme inhibitor 1
1.11	EMC6	ER membrane protein complex subunit 6
1.11	ZDHHC22	zinc finger. DHHC-type containing 22
1.11	TMEM74	transmembrane protein 74
1.11	ZNF425	zinc finger protein 425
1.11	THSD4	Transcript Identified by AceView. Entrez Gene ID(s) 79875
1.11	VWDE	von Willebrand factor D and EGF domains
1.11	CATSPERD	catsper channel auxiliary subunit delta
1.11	KCNS2	potassium voltage-gated channel. modifier subfamily S. member 2
1.11	BCL2L14	BCL2-like 14 (apoptosis facilitator)
1.11	MRPL47	mitochondrial ribosomal protein L47
1.11	INSL5	insulin-like 5

1.11	PLAC8	placenta specific 8
1.11	ETV3L	ets variant 3-like
1.11	ICAM3	intercellular adhesion molecule 3
1.11	MATR3; SNORA74A; SNHG4	matrin 3; small nucleolar RNA. H/ACA box 74A; small nucleolar RNA host gene 4
1.11	C1orf123	chromosome 1 open reading frame 123
1.11	KAT2A	K(lysine) acetyltransferase 2A
1.11	CNTN6	contactin 6
1.11	POLR3K	polymerase (RNA) III (DNA directed) polypeptide K. 12.3 kDa
1.11	ZNF547; TRAPPC2B; TRAPPC2	zinc finger protein 547; trafficking protein particle complex 2B; trafficking protein particle complex 2
1.11	RNASET2	ribonuclease T2
1.11	PPA1	pyrophosphatase (inorganic) 1
1.11	RGS21	regulator of G-protein signaling 21
1.11	KLHL1	kelch-like family member 1
1.11	TMEM243	transmembrane protein 243. mitochondrial
1.11	FBXO11	F-box protein 11
1.11	CWC15	CWC15 spliceosome-associated protein
1.11	DNAH3	dynein. axonemal. heavy chain 3
1.11	TGM4	transglutaminase 4
1.11	CLC	Charcot-Leyden crystal galectin
1.11	LOC100129520	testis expressed sequence 13-like
1.11	TBC1D22A-AS1	TBC1D22A antisense RNA 1
1.11	ANAPC1	anaphase promoting complex subunit 1
1.11	LANCL2	LanC lantibiotic synthetase component C-like 2 (bacterial)
1.11	GNPDA2	glucosamine-6-phosphate deaminase 2
1.11	OR5K3	olfactory receptor. family 5. subfamily K. member 3

1.11	FAM221A	family with sequence similarity 221. member A
1.11	CYP4Z1	cytochrome P450. family 4. subfamily Z. polypeptide 1
1.11	AJUBA	ajuba LIM protein
1.11	SLAMF6	Memczak2013 ANTISENSE. coding. INTERNAL. intronic best transcript NM_052931
1.11	MAGEB5	MAGE family member B5
1.11	TRIM31	tripartite motif containing 31
1.11	B4GALNT1	beta-1.4-N-acetyl-galactosaminyl transferase 1
1.11	ADAT1	adenosine deaminase. tRNA-specific 1
1.11	HTATSF1	HIV-1 Tat specific factor 1
1.11	ASCC1	Transcript Identified by AceView. Entrez Gene ID(s) 51008
1.11	TTLL4	tubulin tyrosine ligase-like family member 4
1.11	OR4C13	olfactory receptor. family 4. subfamily C. member 13
1.11	TRIM52	tripartite motif containing 52
1.11	ZXDA	zinc finger. X-linked. duplicated A
1.11	WWP1	WW domain containing E3 ubiquitin protein ligase 1
1.11	MARCH1	Memczak2013 ANTISENSE. coding. INTERNAL. intronic best transcript NM_001166373
1.11	BMPR2	bone morphogenetic protein receptor type II
1.11	RAB15	RAB15. member RAS oncogene family
1.11	TSNAX-DISC1	TSNAX-DISC1 readthrough (NMD candidate)
1.11	DLC1	DLC1 Rho GTPase activating protein
1.11	CCDC89	coiled-coil domain containing 89
1.11	ADGRA1	adhesion G protein-coupled receptor A1
1.11	RPE	ribulose-5-phosphate-3-epimerase
1.11	GJB7	gap junction protein beta 7
1.11	KLF10	Kruppel-like factor 10

1.11	DNAJB12	DnaJ (Hsp40) homolog. subfamily B. member 12
1.11	FAM102A	Memczak2013 ANTISENSE. CDS. coding. INTERNAL. intronic. UTR3 best transcript NM_203305
1.11	ZNF354B	Zhang2013 ALT_ACCEPTOR. ALT_DONOR. coding. INTERNAL. intronic best transcript NM_058230
1.11	PPIP5K1	diphosphoinositol pentakisphosphate kinase 1
1.11	PRPF4B	pre-mRNA processing factor 4B
1.11	FAM101A	family with sequence similarity 101. member A
1.11	TSGA10IP	testis specific 10 interacting protein
1.11	C8orf31	chromosome 8 open reading frame 31
1.11	SUPV3L1	SUV3-like helicase
1.11	FAM78B	family with sequence similarity 78. member B
1.11	BOD1L2	biorientation of chromosomes in cell division 1-like 2
1.11	ATP6V1H	ATPase. H+ transporting. lysosomal 50/57kDa. V1 subunit H
1.11	ARF6	ADP-ribosylation factor 6
1.11	ATRX	alpha thalassemia/mental retardation syndrome X-linked
1.11	KBTBD7	kelch repeat and BTB (POZ) domain containing 7
1.11	CDKN2B	cyclin-dependent kinase inhibitor 2B (p15. inhibits CDK4)
1.11	PDE11A	phosphodiesterase 11A
1.11	EPB42	erythrocyte membrane protein band 4.2
1.11	ATG16L1	autophagy related 16-like 1
1.11	CAPZA1	capping protein (actin filament) muscle Z-line. alpha 1
1.11	NXNL1	nucleoredoxin-like 1
1.11	SLC5A12	solute carrier family 5 (sodium/monocarboxylate cotransporter). member 12
1.11	BBS12	Bardet-Biedl syndrome 12
1.11	SMCHD1	structural maintenance of chromosomes flexible hinge domain containing 1
1.11	UHRF1BP1L	UHRF1 binding protein 1-like

1.11	FBXO22	F-box protein 22
1.11	GPD1L	glycerol-3-phosphate dehydrogenase 1-like
1.11	EPN2; EPN2-IT1	epsin 2; EPN2 intronic transcript 1
1.11	SEMA4D	Memczak2013 ANTISENSE. coding. INTERNAL. UTR3 best transcript NM_006378
1.11	LINC00598	long intergenic non-protein coding RNA 598
1.11	WDR77	WD repeat domain 77
1.11	SIRPB1	signal-regulatory protein beta 1
1.11	GAS2L2	growth arrest-specific 2 like 2
1.11	RP11-307P5.1; SAMD5	novel transcript; Transcript Identified by AceView. Entrez Gene ID(s) 389432
1.11	ARPC2	actin related protein 2/3 complex subunit 2
1.11	CLEC2D	C-type lectin domain family 2. member D
1.11	C6orf163	chromosome 6 open reading frame 163
1.11	CLPX	caseinolytic mitochondrial matrix peptidase chaperone subunit
1.11	A4GALT	alpha 1.4-galactosyltransferase
1.11	CCL4L2; CCL4; CCL4L1	chemokine (C-C motif) ligand 4-like 2; chemokine (C-C motif) ligand 4; chemokine (C-C motif) ligand 4-like 1
1.11	AQP7	aquaporin 7
1.11	LHFPL4	lipoma HMGIC fusion partner-like 4
1.11	SMDT1	single-pass membrane protein with aspartate-rich tail 1
1.11	SNAP25	synaptosome associated protein 25kDa
1.11	OR2G2	olfactory receptor. family 2. subfamily G. member 2
1.11	CD163	CD163 molecule
1.11	RUFY1	RUN and FYVE domain containing 1
1.11	TXLNA	taxilin alpha
1.11	LOC79160; RP5-907D15.4	uncharacterized LOC79160; novel transcript
1.11	KCNT1	potassium channel. sodium activated subfamily T. member 1

1.11	RGL3	ral guanine nucleotide dissociation stimulator-like 3
1.11	UGT2B17	UDP glucuronosyltransferase 2 family. polypeptide B17
1.11	LOC100133669; RP11-273G15.2	Homo sapiens uncharacterized LOC100133669 (LOC100133669). long non-coding RNA.; Transcript Identified by AceView. Entrez Gene ID(s) 100133669; novel transcript
1.11	GID4	GID complex subunit 4 homolog
1.11	SPOPL	speckle-type POZ protein-like
1.11	WARS2	tryptophanyl tRNA synthetase 2. mitochondrial
1.11	RPL7A	ribosomal protein L7a
1.11	TMEM200B	transmembrane protein 200B
1.11	SPATA31C1	SPATA31 subfamily C. member 1
1.11	GLS2	glutaminase 2 (liver. mitochondrial)
1.11	TMEM181	transmembrane protein 181
1.11	ZNF429	zinc finger protein 429
1.11	CHCHD10	coiled-coil-helix-coiled-coil-helix domain containing 10
1.11	HYDIN	HYDIN. axonemal central pair apparatus protein
1.11	CLEC16A	C-type lectin domain family 16. member A
1.11	TAS2R3	taste receptor. type 2. member 3
1.11	RLBP1	retinaldehyde binding protein 1
1.11	TARBP1	TAR (HIV-1) RNA binding protein 1
1.11	LINC00493	long intergenic non-protein coding RNA 493
1.11	CYB5RL	cytochrome b5 reductase-like
1.11	FAM222B	family with sequence similarity 222. member B
1.11	B3GNT6	UDP-GlcNAc:betaGal beta-1.3-N-acetylglucosaminyltransferase 6
1.11	SCAF8; TIAM2	SR-related CTD-associated factor 8; T-cell lymphoma invasion and metastasis 2
1.11	GRIA1	Transcript Identified by AceView. Entrez Gene ID(s) 2890

1.11	MCMDC2	minichromosome maintenance domain containing 2
1.11	IL17D	interleukin 17D
1.11	HNRNPCL1	heterogeneous nuclear ribonucleoprotein C-like 1
1.11	IL17RE	interleukin 17 receptor E
1.11	DOK1	docking protein 1
1.11	NPC1	Niemann-Pick disease. type C1
1.11	CD3E	CD3e molecule. epsilon (CD3-TCR complex)
1.11	STK31	serine/threonine kinase 31
1.11	C2orf80	chromosome 2 open reading frame 80
1.11	TUBGCP4	tubulin. gamma complex associated protein 4
1.11	PRDM4	PR domain containing 4
1.11	HDAC8	histone deacetylase 8
1.11	LRIG1	
1.11	TMUB1	transmembrane and ubiquitin-like domain containing 1
1.11	ALDH1A1	aldehyde dehydrogenase 1 family. member A1
1.11	PRYP4	PTPN13-like. Y-linked pseudogene 4
1.11	IL18R1	interleukin 18 receptor 1
1.11	PROSER3	proline and serine rich 3
1.11	SLC22A8	solute carrier family 22 (organic anion transporter). member 8
1.11	F2RL2	coagulation factor II (thrombin) receptor-like 2
1.11	MYO1G	myosin IG
1.11	SFI1	SFI1 centrin binding protein
1.11	C21orf91; C21orf91-OT1	chromosome 21 open reading frame 91; C21orf91 overlapping transcript 1
1.11	MRPS17	mitochondrial ribosomal protein S17
1.11	RBL1	retinoblastoma-like 1
1.11	C7orf55-LUC7L2; LUC7L2;	C7orf55-LUC7L2 readthrough; LUC7-like 2 pre-mRNA splicing factor;

	C7orf55	chromosome 7 open reading frame 55
1.11	C1D	C1D nuclear receptor corepressor
1.11	UBXN7	UBX domain protein 7
1.11	OMD	osteomodulin
1.11	SEH1L	SEH1-like nucleoporin
1.11	TMPRSS11D	transmembrane protease. serine 11D
1.11	MYL3	myosin. light chain 3. alkali; ventricular. skeletal. slow
1.11	IFNA7	interferon. alpha 7
1.11	TNFSF15	tumor necrosis factor (ligand) superfamily. member 15
1.11	VAPB	VAMP (vesicle-associated membrane protein)-associated protein B and C
1.11	C3orf33	chromosome 3 open reading frame 33
1.11	TGIF2LY	TGFB-induced factor homeobox 2-like. Y-linked
1.11	CLDN20	claudin 20
1.11	IVL	involucrin
1.11	PTCHD4	patched domain containing 4
1.11	CMTM2	CKLF-like MARVEL transmembrane domain containing 2
1.11	FAM195A	family with sequence similarity 195. member A
1.11	SRPX	sushi-repeat containing protein. X-linked
1.11	ADAMTS13	ADAM metallopeptidase with thrombospondin type 1 motif 13
1.11	DZIP1	DAZ interacting zinc finger protein 1
1.11	KAT6B	K(lysine) acetyltransferase 6B
1.11	HES4	hes family bHLH transcription factor 4
1.11	DERL3	derlin 3
1.11	JMJD1C	jumonji domain containing 1C
1.11	RGS9BP	regulator of G protein signaling 9 binding protein
1.11	ARID1B	AT rich interactive domain 1B (SWI1-like)

1.11	PPP2R3A	protein phosphatase 2. regulatory subunit B. alpha
1.11	MEDAG	mesenteric estrogen-dependent adipogenesis
1.11	ZNF480	zinc finger protein 480
1.11	TRIM16	tripartite motif containing 16
1.11	ZBTB38	zinc finger and BTB domain containing 38
1.11	PPP2R2A	protein phosphatase 2. regulatory subunit B. alpha
1.11	OR8B8	olfactory receptor. family 8. subfamily B. member 8
1.11	VKORC1	vitamin K epoxide reductase complex subunit 1
1.11	RANGAP1	Ran GTPase activating protein 1
1.11	ATG101	autophagy related 101
1.11	ZNF740	zinc finger protein 740
1.11	C1QL2	complement component 1. q subcomponent-like 2
1.11	ETV3	ets variant 3
1.11	RNASE10	ribonuclease. RNase A family. 10 (non-active)
1.11	USP32	ubiquitin specific peptidase 32
1.11	PCGF1	polycomb group ring finger 1
1.11	ZNF830	zinc finger protein 830
1.11	CSMD3	CUB and Sushi multiple domains 3
1.11	GFER	growth factor. augmenter of liver regeneration
1.11	RUFY4	RUN and FYVE domain containing 4
1.11	OR2AG2	olfactory receptor. family 2. subfamily AG. member 2
1.11	SLC18A3	solute carrier family 18 (vesicular acetylcholine transporter). member 3
1.11	CLTA	clathrin. light chain A
1.11	WDR93	WD repeat domain 93
1.11	PLBD1	phospholipase B domain containing 1
1.11	C17orf74	chromosome 17 open reading frame 74

1.11	FAM24A	family with sequence similarity 24. member A
1.11	LRRC36	leucine rich repeat containing 36
1.11	JMY	junction mediating and regulatory protein. p53 cofactor
1.11	OR5H15; OR5H5P	olfactory receptor. family 5. subfamily H. member 15; olfactory receptor. family 5. subfamily H. member 5 pseudogene
1.11	GABRG2	gamma-aminobutyric acid (GABA) A receptor. gamma 2
1.11	DLC1	DLC1 Rho GTPase activating protein
1.11	CMTM5	CKLF-like MARVEL transmembrane domain containing 5
1.11	PABPC4L	poly(A) binding protein. cytoplasmic 4-like
1.11	RPH3A	rabphilin 3A
1.11	USP17L13	ubiquitin specific peptidase 17-like family member 13
1.11	ZYG11A	zyg-11 family member A. cell cycle regulator
1.11	GAGE10	G antigen 10
1.11	MIER2	Zhang2013 ALT_ACCEPTOR. ALT_DONOR. coding. INTERNAL. intronic. OVERLAPTX best transcript NM_017550
1.11	NAT2	N-acetyltransferase 2 (arylamine N-acetyltransferase)
1.11	AK3	adenylate kinase 3
1.11	C9orf153	chromosome 9 open reading frame 153
1.11	LOC101928327; AC007557.1	uncharacterized LOC101928327; novel transcript
1.11	PDZD9	PDZ domain containing 9
1.11	CEP63	centrosomal protein 63kDa
1.11	TARS	threonyl-tRNA synthetase
1.11	AKAP8L	A kinase (PRKA) anchor protein 8-like
1.11	FAM124A	family with sequence similarity 124 member A
1.11	BICD1	bicaudal D homolog 1 (Drosophila)
1.11	CDC42EP2	CDC42 effector protein (Rho GTPase binding) 2
1.11	RPS27A	ribosomal protein S27a

1.11	CARS	cysteinyl-tRNA synthetase
1.11	PKMYT1	protein kinase. membrane associated tyrosine/threonine 1
1.11	HSD17B11	hydroxysteroid (17-beta) dehydrogenase 11
1.11	MCHR2	melanin-concentrating hormone receptor 2
1.11	MUS81	MUS81 structure-specific endonuclease subunit
1.11	SLC35D1	solute carrier family 35 (UDP-GlcA/UDP-GalNAc transporter). member D1
1.11	CALHM3	calcium homeostasis modulator 3
1.11	CC2D2A	coiled-coil and C2 domain containing 2A
1.11	KCTD9	potassium channel tetramerization domain containing 9
1.11	CPSF2	cleavage and polyadenylation specific factor 2
1.11	UBE2M	ubiquitin-conjugating enzyme E2M
1.11	RRP8	Transcript Identified by AceView. Entrez Gene ID(s) 23378
1.11	SSMEM1	serine-rich single-pass membrane protein 1
1.11	COLCA1	colorectal cancer associated 1
1.11	PXMP4	peroxisomal membrane protein 4
1.11	C9	complement component 9
1.11	EXOSC5	exosome component 5
1.11	PIGU	Salzman2013 ALT_ACCEPTOR. ALT_DONOR. coding. INTERNAL. intronic best transcript NM_080476
1.11	DOCK4	dedicator of cytokinesis 4
1.11	PCNP	PEST proteolytic signal containing nuclear protein
1.11	IBTK	inhibitor of Bruton agammaglobulinemia tyrosine kinase
1.11	C7orf65	chromosome 7 open reading frame 65
1.11	FAM210B	family with sequence similarity 210. member B
1.11	COA4	cytochrome c oxidase assembly factor 4 homolog
1.11	CHST12	carbohydrate (chondroitin 4) sulfotransferase 12

1.11	NKAIN2	Na+/K+ transporting ATPase interacting 2
1.11	DCAF17	DDB1 and CUL4 associated factor 17
1.11	LIN7C	lin-7 homolog C (C. elegans)
1.11	CYP46A1	cytochrome P450. family 46. subfamily A. polypeptide 1
1.11	LRP6	LDL receptor related protein 6
1.11	RUVBL2	RuvB-like AAA ATPase 2
1.11	SPANXD	SPANX family. member D
1.11	DARS2	aspartyl-tRNA synthetase 2. mitochondrial
1.11	GPX6	glutathione peroxidase 6
1.11	HMGXB3	HMG box domain containing 3
1.11	TAF2	TAF2 RNA polymerase II. TATA box binding protein (TBP)-associated factor. 150kDa
1.11	C1QC	complement component 1. q subcomponent. C chain
1.11	C17orf98	chromosome 17 open reading frame 98
1.11	URGCP; URGCP-MRPS24	upregulator of cell proliferation; URGCP-MRPS24 readthrough
1.11	MFAP1	microfibrillar associated protein 1
1.11	IL6R	Memczak2013 ANTISENSE. coding. INTERNAL. UTR3 best transcript NM_000565
1.11	REST	RE1-silencing transcription factor
1.11	KLHL12	kelch-like family member 12
1.11	P4HA2	prolyl 4-hydroxylase. alpha polypeptide II
1.11	ZMYND11	zinc finger. MYND-type containing 11
1.11	MMP25	matrix metallopeptidase 25
1.11	ZNF507	zinc finger protein 507
1.11	WFDC13	WAP four-disulfide core domain 13
1.11	ISPD	isoprenoid synthase domain containing
1.11	DEFB123	defensin. beta 123

1.11	PDK4	pyruvate dehydrogenase kinase. isozyme 4
1.11	RFPL3S	RFPL3 antisense
1.11	DDX55	DEAD (Asp-Glu-Ala-Asp) box polypeptide 55
1.11	ZNF573	zinc finger protein 573
1.11	SEL1L2	sel-1 suppressor of lin-12-like 2 (C. elegans)
1.11	HMHB1	histocompatibility (minor) HB-1
1.11	ZFP91-CNTF	ZFP91-CNTF readthrough (NMD candidate)
1.11	LSG1	large 60S subunit nuclear export GTPase 1
1.11	C20orf173	chromosome 20 open reading frame 173
1.11	ZNF571	zinc finger protein 571
1.11	ATG16L1	autophagy related 16-like 1
1.11	CFL1	cofilin 1 (non-muscle)
1.11	C7orf26	chromosome 7 open reading frame 26
1.11	ATG9A	autophagy related 9A
1.11	ZNF589	zinc finger protein 589
1.11	RELT	RELT tumor necrosis factor receptor
1.11	ZNF780A	zinc finger protein 780A
1.11	PADI6	peptidyl arginine deiminase. type VI
1.11	STAR	steroidogenic acute regulatory protein
1.11	PRRC2A	proline-rich coiled-coil 2A
1.11	FAM205C	family with sequence similarity 205. member C
1.11	UFL1	UFM1-specific ligase 1
1.11	FOXP1; RP11-298C2.1	Transcript Identified by AceView. Entrez Gene ID(s) 27086; novel transcript. sense intronic to FOXP1
1.11	C6orf58	chromosome 6 open reading frame 58
1.11	BCL2L14	BCL2-like 14 (apoptosis facilitator)

1.10	ATP2C1	ATPase. Ca++ transporting. type 2C. member 1
1.10	ERMP1	endoplasmic reticulum metallopeptidase 1
1.10	PLA2G2E	phospholipase A2. group IIE
1.10	TMEM45B	transmembrane protein 45B
1.10	C12orf71	chromosome 12 open reading frame 71
1.10	LRRTM4	leucine rich repeat transmembrane neuronal 4
1.10	BAZ1B	bromodomain adjacent to zinc finger domain 1B
1.10	FOXI3	forkhead box I3
1.10	CRTAM	cytotoxic and regulatory T-cell molecule
1.10	ADAMTSL4	ADAMTS like 4
1.10	TSPAN6	tetraspanin 6
1.10	SERPINA7	serpin peptidase inhibitor. clade A (alpha-1 antiproteinase. antitrypsin). member 7
1.10	BCO2; RPS12P21	beta-carotene oxygenase 2; ribosomal protein S12 pseudogene 21
1.10	ZNF41	zinc finger protein 41
1.10	QSOX2	quiescin Q6 sulfhydryl oxidase 2
1.10	SIK1	salt-inducible kinase 1
1.10	SLC8A2	solute carrier family 8 (sodium/calcium exchanger). member 2
1.10	CCDC190	coiled-coil domain containing 190
1.10	CYB5R2	cytochrome b5 reductase 2
1.10	FGFBP1	fibroblast growth factor binding protein 1
1.10	OLFM1	olfactomedin 1
1.10	MAGOH	mago homolog. exon junction complex core component
1.10	SLC12A6	solute carrier family 12 (potassium/chloride transporter). member 6
1.10	NDUFA10	NADH dehydrogenase (ubiquinone) 1 alpha subcomplex. 10. 42kDa
1.10	TRIM55	tripartite motif containing 55

1.10	CDC42SE2	CDC42 small effector 2
1.10	PHF5A	PHD finger protein 5A
1.10	MGAT4A	mannosyl (alpha-1.3-)-glycoprotein beta-1.4-N-acetylglucosaminyltransferase. isozyme A
1.10	MPDZ	multiple PDZ domain protein
1.10	C17orf47	chromosome 17 open reading frame 47
1.10	WSCD1	WSC domain containing 1
1.10	LRRC4C	leucine rich repeat containing 4C
1.10	FARS2	phenylalanyl-tRNA synthetase 2. mitochondrial
1.10	TSPAN13	tetraspanin 13
1.10	CCT5	chaperonin containing TCP1. subunit 5 (epsilon)
1.10	C2orf50	chromosome 2 open reading frame 50
1.10	RAB6B	RAB6B. member RAS oncogene family
1.10	PTPN13	protein tyrosine phosphatase. non-receptor type 13 (APO-1/CD95 (Fas)-associated phosphatase)
1.10	TMEM185A	transmembrane protein 185A
1.10	OR5AC2	olfactory receptor. family 5. subfamily AC. member 2
1.10	OR10H1	olfactory receptor. family 10. subfamily H. member 1
1.10	ZNF197	zinc finger protein 197
1.10	FAM181B	family with sequence similarity 181. member B
1.10	C6orf132	chromosome 6 open reading frame 132
1.10	LDHB	lactate dehydrogenase B
1.10	GIF	gastric intrinsic factor (vitamin B synthesis)
1.10	TMCO2	transmembrane and coiled-coil domains 2
1.10	DICER1	dicer 1. ribonuclease type III
1.10	FAM102A	family with sequence similarity 102. member A
1.10	KLRG2	killer cell lectin-like receptor subfamily G. member 2

1.10	FAM76B	family with sequence similarity 76. member B
1.10	DCLRE1A	DNA cross-link repair 1A
1.10	HNRNPCL1	heterogeneous nuclear ribonucleoprotein C-like 1
1.10	OR8B3	olfactory receptor. family 8. subfamily B. member 3
1.10	TNKS2	tankyrase. TRF1-interacting ankyrin-related ADP-ribose polymerase 2
1.10	TERF2	telomeric repeat binding factor 2
1.10	CCDC6	coiled-coil domain containing 6
1.10	PRDX4	peroxiredoxin 4
1.10	TMLHE-AS1; LOC101927830; CLIC2; RP13-228J13.1	Homo sapiens TMLHE antisense RNA 1 (TMLHE-AS1). long non-coding RNA.; uncharacterized LOC101927830; Transcript Identified by AceView. Entrez Gene ID(s) 1193; novel transcript
1.10	KCNIP4	Kv channel interacting protein 4
1.10	KRTAP19-3	keratin associated protein 19-3
1.10	THSD7A	thrombospondin type 1 domain containing 7A
1.10	GDPD4	glycerophosphodiester phosphodiesterase domain containing 4
1.10	OR8J1	olfactory receptor. family 8. subfamily J. member 1
1.10	IFNGR2	Memczak2013 ALT_ACCEPTOR. ALT_DONOR. coding. INTERNAL. intronic best transcript NM_005534
1.10	COL6A1	collagen. type VI. alpha 1
1.10	PLA2G2F	phospholipase A2. group IIF
1.10	SRSF7	serine/arginine-rich splicing factor 7
1.10	DMBX1	diencephalon/mesencephalon homeobox 1
1.10	MMRN1	multimerin 1
1.10	VWC2	von Willebrand factor C domain containing 2
1.10	CD1C	CD1c molecule
1.10	ODF3B	outer dense fiber of sperm tails 3B
1.10	CLCA4	chloride channel accessory 4

1.10	FIGLA	folliculogenesis specific bHLH transcription factor
1.10	SYT5	synaptotagmin V
1.10	INTS6	integrator complex subunit 6
1.10	ARHGAP10	Transcript Identified by AceView. Entrez Gene ID(s) 79658
1.10	SSTR3	somatostatin receptor 3
1.10	STK32B	serine/threonine kinase 32B
1.10	ZC2HC1B	zinc finger. C2HC-type containing 1B
1.10	VAT1	vesicle amine transport 1
1.10	RPL18A; SNORA68	ribosomal protein L18a; small nucleolar RNA. H/ACA box 68
1.10	VPS13C	vacuolar protein sorting 13 homolog C (S. cerevisiae)
1.10	SETSIP	SET-like protein
1.10	DDX18	DEAD (Asp-Glu-Ala-Asp) box polypeptide 18
1.10	C9orf85	chromosome 9 open reading frame 85
1.10	CCDC67	coiled-coil domain containing 67
1.10	C12orf45	chromosome 12 open reading frame 45
1.10	AFF2	AF4/FMR2 family. member 2
1.10	TSKS	testis-specific serine kinase substrate
1.10	TMEM247	transmembrane protein 247
1.10	SH3BGRL2	SH3 domain binding glutamate-rich protein like 2
1.10	HTR1F	5-hydroxytryptamine (serotonin) receptor 1F. G protein-coupled
1.10	F3	coagulation factor III (thromboplastin. tissue factor)
1.10	CYTH2	cytohesin 2
1.10	ZNF200	Transcript Identified by AceView. Entrez Gene ID(s) 7752
1.10	GPR182	G protein-coupled receptor 182
1.10	SF3A1	splicing factor 3a. subunit 1. 120kDa
1.10	ZC3H15	zinc finger CCCH-type containing 15

1.10	LY9	lymphocyte antigen 9
1.10	WFIKKN2	WAP. follistatin/kazal. immunoglobulin. kunitz and netrin domain containing 2
1.10	GNG13	guanine nucleotide binding protein (G protein). gamma 13
1.10	CHAC2	ChaC. cation transport regulator homolog 2 (E. coli)
1.10	RASA3	RAS p21 protein activator 3
1.10	WDR44	WD repeat domain 44
1.10	GOLGA6L10	golgin A6 family-like 10
1.10	GTPBP1	GTP binding protein 1
1.10	MAP3K7	mitogen-activated protein kinase kinase kinase 7
1.10	FOXA3	forkhead box A3
1.10	USPL1	ubiquitin specific peptidase like 1
1.10	CXCR5	chemokine (C-X-C motif) receptor 5
1.10	XIRP1	xin actin binding repeat containing 1
1.10	OLFM2	olfactomedin 2
1.10	C1orf43	chromosome 1 open reading frame 43
1.10	TRIM64C	tripartite motif containing 64C
1.10	ZNF549	zinc finger protein 549
1.10	PRR9	proline rich 9
1.10	AGXT	alanine-glyoxylate aminotransferase
1.10	LARP4B	La ribonucleoprotein domain family. member 4B
1.10	SEMG1	semenogelin I
1.10	RNF144A	ring finger protein 144A
1.10	ZBTB41	zinc finger and BTB domain containing 41
1.10	SYNCRIP	synaptotagmin binding. cytoplasmic RNA interacting protein
1.10	MPRIP	myosin phosphatase Rho interacting protein

1.10	RPLP1	ribosomal protein. large. P1
1.10	SIDT1	SID1 transmembrane family. member 1
1.10	TRMT5	tRNA methyltransferase 5
1.10	PON1	paraoxonase 1
1.10	SMIM8	small integral membrane protein 8
1.10	RGL2	ral guanine nucleotide dissociation stimulator-like 2
1.10	LEKR1	leucine. glutamate and lysine rich 1
1.10	TCF15	transcription factor 15 (basic helix-loop-helix)
1.10	AGA	aspartylglucosaminidase
1.10	MAPK15	mitogen-activated protein kinase 15
1.10	C15orf39	chromosome 15 open reading frame 39
1.10	OR2A25	olfactory receptor. family 2. subfamily A. member 25
1.10	TBCB	tubulin folding cofactor B
1.10	P3H4	prolyl 3-hydroxylase family member 4 (non-enzymatic)
1.10	CABLES1	Cdk5 and Abl enzyme substrate 1
1.10	ZFP30	ZFP30 zinc finger protein
1.10	GNAI3	guanine nucleotide binding protein (G protein). alpha inhibiting activity polypeptide 3
1.10	MAP2K1	Transcript Identified by AceView. Entrez Gene ID(s) 5604
1.10	SLC35G2	solute carrier family 35. member G2
1.10	LINC00452	long intergenic non-protein coding RNA 452
1.10	DHX9	DEAH (Asp-Glu-Ala-His) box helicase 9
1.10	RAB31	Transcript Identified by AceView. Entrez Gene ID(s) 11031
1.10	RAB21	RAB21. member RAS oncogene family
1.10	RASL10B	RAS-like. family 10. member B
1.10	TSPAN5	tetraspanin 5

1.10	PRAME	preferentially expressed antigen in melanoma
1.10	ZNF35	zinc finger protein 35
1.10	PNP	purine nucleoside phosphorylase
1.10	ZBTB7C	zinc finger and BTB domain containing 7C
1.10	TCAF2	TRPM8 channel-associated factor 2
1.10	DTWD1	DTW domain containing 1
1.10	CWC22	CWC22 homolog. spliceosome-associated protein
1.10	CTH	cystathionine gamma-lyase
1.10	C12orf40	chromosome 12 open reading frame 40
1.10	ATP10B	ATPase. class V. type 10B
1.10	F13A1	coagulation factor XIII. A1 polypeptide
1.10	SLC9A5	solute carrier family 9. subfamily A (NHE5. cation proton antiporter 5). member 5
1.10	TMPRSS15	transmembrane protease. serine 15
1.10	TFPI2	tissue factor pathway inhibitor 2
1.10	KIRREL2	kin of IRRE like 2 (Drosophila)
1.10	OTOP3	otopetrin 3
1.10	TRIM6-TRIM34	TRIM6-TRIM34 readthrough
1.10	APH1B	APH1B gamma secretase subunit
1.10	DSCR8	Down syndrome critical region 8
1.10	GCM1	glial cells missing homolog 1 (Drosophila)
1.10	SLU7	SLU7 homolog. splicing factor
1.10	HMGXB4	HMG box domain containing 4
1.10	LOC643355; RP11-88H9.2; toyfawbu	uncharacterized LOC643355; putative novel transcript; Transcript Identified by AceView
1.10	RBMY1D; RBMY1B; RBMY1A1	RNA binding motif protein. Y-linked. family 1. member D; RNA binding motif protein. Y-linked. family 1. member B; RNA binding motif protein. Y-linked.

		family 1. member A1
1.10	NIP7	NIP7. nucleolar pre-rRNA processing protein
1.10	TMEM231	transmembrane protein 231
1.10	HNRNPCL3; HNRNPCL4	heterogeneous nuclear ribonucleoprotein C-like 3; heterogeneous nuclear ribonucleoprotein C-like 4
1.10	UGT2A2; UGT2A1	UDP glucuronosyltransferase 2 family. polypeptide A2; UDP glucuronosyltransferase 2 family. polypeptide A1. complex locus
1.10	OR4N4	olfactory receptor. family 4. subfamily N. member 4
1.10	EPPIN-WFDC6	EPPIN-WFDC6 readthrough
1.10	EMC3	ER membrane protein complex subunit 3
1.10	MCAT	malonyl-CoA-acyl carrier protein transacylase
1.10	CHM	choroideremia (Rab escort protein 1)
1.10	PRSS35	protease. serine 35
1.10	HOXB-AS3	HOXB cluster antisense RNA 3
1.10	NCOR2	nuclear receptor corepressor 2
1.10	VPS13B	vacuolar protein sorting 13 homolog B (yeast)
1.10	RPS21	ribosomal protein S21
1.10	RPL41P1	ribosomal protein L41 pseudogene 1
1.10	DAP3	death associated protein 3
1.10	TSPAN17	tetraspanin 17
1.10	EIF2S2	eukaryotic translation initiation factor 2. subunit 2 beta. 38kDa
1.10	DYNC1LI2	dynein. cytoplasmic 1. light intermediate chain 2
1.10	C19orf24	chromosome 19 open reading frame 24
1.10	PPIAL4G	peptidylprolyl isomerase A (cyclophilin A)-like 4G
1.10	SCAND2P	SCAN domain containing 2 pseudogene
1.10	EXOSC7	exosome component 7

1.10	BAK1	BCL2-antagonist/killer 1
1.10	OR2F1	olfactory receptor. family 2. subfamily F. member 1 (gene/pseudogene)
1.10	WASF2	WAS protein family. member 2
1.10	SRRM1	serine/arginine repetitive matrix 1
1.10	CTXN3	cortexin 3
1.10	C14orf93	chromosome 14 open reading frame 93
1.10	WDR53	WD repeat domain 53
1.10	STIP1	stress-induced phosphoprotein 1
1.10	NPIPB4	nuclear pore complex interacting protein family. member B4
1.10	PITPNM2	phosphatidylinositol transfer protein. membrane-associated 2
1.10	PIK3CD-AS1	PIK3CD antisense RNA 1
1.10	TBK1	TANK-binding kinase 1
1.10	CCDC168	coiled-coil domain containing 168
1.10	TBC1D9B	TBC1 domain family. member 9B (with GRAM domain)
1.10	HAVCR2	hepatitis A virus cellular receptor 2
1.10	OBP2B	odorant binding protein 2B
1.10	OSTF1	osteoclast stimulating factor 1
1.10	GINS3	GINS complex subunit 3 (Psf3 homolog)
1.10	C2orf61	chromosome 2 open reading frame 61
1.10	ACTN2	actinin. alpha 2
1.10	TERT	telomerase reverse transcriptase
1.10	MRPL18	mitochondrial ribosomal protein L18
1.10	HMSD	histocompatibility (minor) serpin domain containing
1.10	DNAJC19	DnaJ (Hsp40) homolog. subfamily C. member 19
1.10	FCHSD2	FCH and double SH3 domains 2
1.10	ADAMTS13	ADAM metallopeptidase with thrombospondin type 1 motif 13

1.10	FABP2	fatty acid binding protein 2. intestinal
1.10	STK32A	serine/threonine kinase 32A
1.10	SMARCC2	SWI/SNF related. matrix associated. actin dependent regulator of chromatin. subfamily c. member 2
1.10	PAIP1	poly(A) binding protein interacting protein 1
1.10	TNFSF8	tumor necrosis factor (ligand) superfamily. member 8
1.10	TCERG1	transcription elongation regulator 1
1.10	NRIP1	nuclear receptor interacting protein 1
1.10	TCEAL1	transcription elongation factor A (SII)-like 1
1.10	C7orf25	chromosome 7 open reading frame 25
1.10	ZNF506	Transcript Identified by AceView. Entrez Gene ID(s) 284440; 440515
1.10	GSX1	GS homeobox 1
1.10	TBL1X	transducin (beta)-like 1X-linked
1.10	NSF	N-ethylmaleimide-sensitive factor
1.10	HDDC3	HD domain containing 3
1.10	ARHGEF2	Rho/Rac guanine nucleotide exchange factor 2
1.10	FBXO47	F-box protein 47
1.10	CETN1	centrin 1
1.10	CCDC40	coiled-coil domain containing 40
1.10	ADAMTS13	ADAM metallopeptidase with thrombospondin type 1 motif 13
1.10	LINC00116	long intergenic non-protein coding RNA 116
1.10	FMN1	formin 1
1.10	EPB42	erythrocyte membrane protein band 4.2
1.10	METTL5	methyltransferase like 5
1.10	HCN4	hyperpolarization activated cyclic nucleotide gated potassium channel 4
1.10	CDKL1	cyclin-dependent kinase-like 1 (CDC2-related kinase)

1.10	OR2T6	olfactory receptor. family 2. subfamily T. member 6
1.10	HYAL4	hyaluronoglucosaminidase 4
1.10	MMADHC	methylmalonic aciduria (cobalamin deficiency) cblD type. with homocystinuria
1.10	SEMA3D	sema domain. immunoglobulin domain (Ig). short basic domain. secreted. (semaphorin) 3D
1.10	AP4E1	adaptor-related protein complex 4. epsilon 1 subunit
1.10	USP6	ubiquitin specific peptidase 6
1.10	SAG	S-antigen; retina and pineal gland (arrestin)
1.10	FGB	fibrinogen beta chain
1.10	HIBADH	3-hydroxyisobutyrate dehydrogenase
1.10	PMCH	pro-melanin-concentrating hormone
1.10	PXDNL	peroxidasin like
1.10	TAF4	TAF4 RNA polymerase II. TATA box binding protein (TBP)-associated factor. 135kDa
1.10	EML5	Transcript Identified by AceView. Entrez Gene ID(s) 161436
1.10	G6PC2	glucose-6-phosphatase. catalytic. 2
1.10	CPNE2	copine II
1.10	CATSPERB	catsper channel auxiliary subunit beta
1.10	GAPVD1	GTPase activating protein and VPS9 domains 1
1.10	SCLY	selenocysteine lyase
1.10	CD1E	CD1e molecule
1.10	SYTL5	synaptotagmin-like 5
1.10	TAS1R2	taste receptor. type 1. member 2
1.10	TAS2R13	taste receptor. type 2. member 13
1.10	SLC17A8	solute carrier family 17 (vesicular glutamate transporter). member 8
1.10	SPCS2	signal peptidase complex subunit 2

1.10	RAP1B	RAP1B. member of RAS oncogene family
1.10	ZNF461	zinc finger protein 461
1.10	CACNA1G	calcium channel. voltage-dependent. T type. alpha 1G subunit
1.10	THEG	theg spermatid protein
1.10	ELL	elongation factor RNA polymerase II
1.10	SNCG	synuclein gamma
1.10	CILP	cartilage intermediate layer protein. nucleotide pyrophosphohydrolase
1.10	MDH1B	malate dehydrogenase 1B
1.10	TP53TG3D; LOC102723655; TP53TG3; AC136612.1	Homo sapiens TP53 target 3D (TP53TG3D). transcript variant 2. non-coding RNA.; TP53-target gene 3 protein; Homo sapiens TP53 target 3. mRNA (cDNA clone MGC:119889 IMAGE:40015196). complete cds.; Homo sapiens TP53 target 3. mRNA (cDNA clone MGC:119888 IMAGE:40015195). complete cds.
1.10	HYOU1	hypoxia up-regulated 1
1.10	PPP5D1	PPP5 tetratricopeptide repeat domain containing 1
1.10	CMTR2	cap methyltransferase 2
1.10	GFRA3	GDNF family receptor alpha 3
1.10	EIF3F	Eukaryotic translation initiation factor 3 subunit F [Source:UniProtKB/Swiss-Prot;Acc:O00303]
1.10	RDM1	RAD52 motif containing 1
1.10	TNMD	tenomodulin
1.10	PWP2	PWP2 periodic tryptophan protein homolog (yeast)
1.10	LOC100652768; PCSK7	uncharacterized LOC100652768; Salzman2013 ANNOTATED. ncRNA. OVCODE. OVERLAPTX. OVEXON best transcript NR_045215; Transcript Identified by AceView. Entrez Gene ID(s) 9159
1.10	CEP350	centrosomal protein 350kDa
1.10	DHDH	dihydrodiol dehydrogenase (dimeric)
1.10	FANK1	fibronectin type III and ankyrin repeat domains 1
1.10	PCMT1	protein-L-isoaspartate (D-aspartate) O-methyltransferase

1.10	KLK6	kallikrein related peptidase 6
1.10	LOC105377348; RP11-10L12.4; UBE2D3; pugo	uncharacterized LOC105377348; Salzman2013 ANTISENSE. coding. INTERNAL. intronic. OVERLAPTX best transcript NM_181890; novel transcript. antisense UBE2D3; Transcript Identified by AceView
1.10	VMA21	VMA21 vacuolar H+-ATPase homolog (S. cerevisiae)
1.10	LOC653653; AC025048.1	adaptor-related protein complex 1. sigma 2 subunit pseudogene
1.10	C5orf63	chromosome 5 open reading frame 63
1.10	EFCAB10	EF-hand calcium binding domain 10
1.10	TBX18	T-box 18
1.10	UBR3	ubiquitin protein ligase E3 component n-recognin 3 (putative)
1.10	LOC389895	chromosome 16 open reading frame 72-like
1.10	MAS1L	MAS1 proto-oncogene like. G protein-coupled receptor
1.10	CD4	CD4 molecule
1.10	XRN2	5-3 exoribonuclease 2
1.10	GPR88	G protein-coupled receptor 88
1.10	GHRHR	growth hormone releasing hormone receptor
1.10	TAS2R38	taste receptor. type 2. member 38
1.10	ELN	elastin
1.10	KRTAP13-2	keratin associated protein 13-2
1.10	HERC2	HECT and RLD domain containing E3 ubiquitin protein ligase 2
1.10	HSFY2	heat shock transcription factor. Y-linked 2
1.10	XKR7	X-linked Kx blood group related 7
1.10	GLTSCR1L	GLTSCR1-like
1.10	GRASP	GRP1 (general receptor for phosphoinositides 1)-associated scaffold protein
1.10	LRRIQ1	leucine-rich repeats and IQ motif containing 1
1.10	FKBP11; ARF3	FK506 binding protein 11; ADP-ribosylation factor 3
1.10	MBD3L4	methyl-CpG binding domain protein 3-like 4

1.10	KLHDC3	kelch domain containing 3
1.10	SNIP1	Smad nuclear interacting protein 1
1.10	RHOBTB1	Rho-related BTB domain containing 1
1.10	AADACL4	arylacetamide deacetylase-like 4
1.10	MYPN	myopalladin
1.10	ADO	2-aminoethanethiol (cysteamine) dioxygenase
1.10	C1orf137	chromosome 1 open reading frame 137
1.10	RPL41P2	ribosomal protein L41 pseudogene 2
1.10	CBX3	chromobox homolog 3
1.10	TBC1D3P5	TBC1 domain family. member 3 pseudogene 5
1.10	TMEM8C	transmembrane protein 8C
1.10	CT45A10	cancer/testis antigen family 45. member A10
1.10	VSIG2	V-set and immunoglobulin domain containing 2
1.10	DYX1C1	dyslexia susceptibility 1 candidate 1
1.10	SNTN	sentan. cilia apical structure protein
1.10	TSPAN10	tetraspanin 10
1.10	HPSE	heparanase
1.10	KBTBD6	kelch repeat and BTB (POZ) domain containing 6
1.10	CCNT1	cyclin T1
1.10	MIR1247	microRNA 1247
1.10	NEXN	nexilin (F actin binding protein)
1.10	CNGB1	cyclic nucleotide gated channel beta 1
1.10	TTBK1	tau tubulin kinase 1
1.09	LRRC42	leucine rich repeat containing 42
1.09	CERS6	ceramide synthase 6
1.09	LCE1A	late cornified envelope 1A

1.09	ANKRD16	ankyrin repeat domain 16
1.09	OTUD6B	OTU domain containing 6B
1.09	ARFGEF3	ARFGEF family member 3
1.09	GSK3A	glycogen synthase kinase 3 alpha
1.09	NKX3-2	NK3 homeobox 2
1.09	DYDC1	DPY30 domain containing 1
1.09	OR4D5	olfactory receptor. family 4. subfamily D. member 5
1.09	FAM184B	family with sequence similarity 184. member B
1.09	P2RY1	purinergic receptor P2Y. G-protein coupled. 1
1.09	MALT1	MALT1 paracaspase
1.09	ZNF200	Transcript Identified by AceView. Entrez Gene ID(s) 7752
1.09	IRAK1BP1	interleukin 1 receptor associated kinase 1 binding protein 1
1.09	HHIPL2	HHIP-like 2
1.09	SLC24A1	solute carrier family 24 (sodium/potassium/calcium exchanger). member 1
1.09	KCNQ2	potassium channel. voltage gated KQT-like subfamily Q. member 2
1.09	IVNS1ABP	influenza virus NS1A binding protein
1.09	FRG1	FSHD region gene 1
1.09	SLC36A2	solute carrier family 36 (proton/amino acid symporter). member 2
1.09	RPL41	Synthetic construct Homo sapiens clone IMAGE:100063377. MGC:190767 ribosomal protein L41 (RPL41) mRNA. encodes complete protein.
1.09	LONP2	lon peptidase 2. peroxisomal
1.09	CUX1	cut-like homeobox 1
1.09	TSHZ2	teashirt zinc finger homeobox 2
1.09	SPATA13; C1QTNF9	spermatogenesis associated 13; C1q and tumor necrosis factor related protein 9
1.09	OSBPL8	oxysterol binding protein-like 8
1.09	RAB32	RAB32. member RAS oncogene family

1.09	C6orf132	Zhang2013 ALT_ACCEPTOR. ALT_DONOR. coding. INTERNAL. intronic best transcript NM_001164446
1.09	OR2G3	olfactory receptor. family 2. subfamily G. member 3
1.09	CRH	corticotropin releasing hormone
1.09	ENO1	enolase 1. (alpha)
1.09	C7orf34	chromosome 7 open reading frame 34
1.09	OR1J1	olfactory receptor. family 1. subfamily J. member 1
1.09	PIEZO1	piezo-type mechanosensitive ion channel component 1
1.09	KIFAP3	kinesin-associated protein 3
1.09	CHMP6	charged multivesicular body protein 6
1.09	PSME3	proteasome activator subunit 3
1.09	CC2D1A	coiled-coil and C2 domain containing 1A
1.09	ITGB2	Memczak2013 ANTISENSE. CDS. coding. INTERNAL best transcript NM_001127491
1.09	PRSS57	protease. serine. 57
1.09	KCNAB3	potassium channel. voltage gated subfamily A regulatory beta subunit 3
1.09	BCAR3	breast cancer anti-estrogen resistance 3
1.09	RPS10	ribosomal protein S10
1.09	MCM3AP	minichromosome maintenance complex component 3 associated protein
1.09	RNF32	ring finger protein 32
1.09	SNRNP200	small nuclear ribonucleoprotein. U5 200kDa subunit
1.09	SHOX2	short stature homeobox 2
1.09	DCAF12	DDB1 and CUL4 associated factor 12
1.09	THSD4	Transcript Identified by AceView. Entrez Gene ID(s) 79875
1.09	UST	uronyl-2-sulfotransferase
1.09	DUX4	double homeobox 4
1.09	NUP188	nucleoporin 188kDa

1.09	SERTAD4	SERTA domain containing 4
1.09	MTFR1	mitochondrial fission regulator 1
1.09	GABRB2	gamma-aminobutyric acid (GABA) A receptor. beta 2
1.09	LRFN4	leucine rich repeat and fibronectin type III domain containing 4
1.09	RASGRF1	Ras protein-specific guanine nucleotide-releasing factor 1
1.09	ZNF707	zinc finger protein 707
1.09	NACAD	NAC alpha domain containing
1.09	CASP5	caspase 5
1.09	MEGF6	multiple EGF-like-domains 6
1.09	ADAMTS17	Memczak2013 ANTISENSE. coding. INTERNAL. intronic best transcript NM_139057
1.09	LMNTD1	lamin tail domain containing 1
1.09	ADGRG6	adhesion G protein-coupled receptor G6
1.09	ELF1	E74-like factor 1 (ets domain transcription factor)
1.09	PHB	prohibitin
1.09	TMEM204	transmembrane protein 204
1.09	RASA4B; RASA4	RAS p21 protein activator 4B; RAS p21 protein activator 4
1.09	ZNF829	zinc finger protein 829
1.09	MYT1	myelin transcription factor 1
1.09	SPRYD3	SPRY domain containing 3
1.09	MBD3L2; CTB-25J19.1	Synthetic construct Homo sapiens clone IMAGE:100069019. MGC:199030 methyl-CpG binding domain protein 3-like 2 (MBD3L2) mRNA. encodes complete protein.; novel transcript
1.09	HIGD1A	HIG1 hypoxia inducible domain family. member 1A
1.09	GKN1	gastrokine 1
1.09	COL6A6	collagen. type VI. alpha 6
1.09	SYT3	synaptotagmin III

1.09	WAPL	WAPL cohesin release factor
1.09	PDZD3	PDZ domain containing 3
1.09	OR10H2	olfactory receptor. family 10. subfamily H. member 2
1.09	OR52J3	olfactory receptor. family 52. subfamily J. member 3
1.09	GAGE2D; GAGE13; GAGE2E; GAGE8; GAGE2B; GAGE2A; GAGE2C	G antigen 2D; G antigen 13; G antigen 2E; G antigen 8; G antigen 2B; G antigen 2A; G antigen 2C
1.09	ADAMTSL3	ADAMTS like 3
1.09	SRGN	Transcript Identified by AceView. Entrez Gene ID(s) 5552
1.09	SRL	sarcalumenin
1.09	ATG16L1	autophagy related 16-like 1
1.09	DCAF13	DDB1 and CUL4 associated factor 13
1.09	PLAT	plasminogen activator. tissue
1.09	ACIN1	Transcript Identified by AceView. Entrez Gene ID(s) 22985
1.09	RP5-1198O20.4; KLF17	Transcript Identified by AceView. Entrez Gene ID(s) 128209; novel transcript
1.09	MICAL3	Transcript Identified by AceView. Entrez Gene ID(s) 57553
1.09	FAM133B	family with sequence similarity 133. member B
1.09	STAT5B	signal transducer and activator of transcription 5B
1.09	ZNF281	zinc finger protein 281
1.09	TLCD2	TLC domain containing 2
1.09	GBP4	guanylate binding protein 4
1.09	POLR2D	polymerase (RNA) II (DNA directed) polypeptide D
1.09	RASGRP1	RAS guanyl releasing protein 1 (calcium and DAG-regulated)
1.09	PRR16	proline rich 16
1.09	YAF2	YY1 associated factor 2
1.09	ARHGAP25	Rho GTPase activating protein 25
1.09	TRNP1	TMF1-regulated nuclear protein 1

1.09	ZNF852	zinc finger protein 852
1.09	MEOX1	mesenchyme homeobox 1
1.09	MYOM2	myomesin 2
1.09	HYOU1	hypoxia up-regulated 1
1.09	NOBOX	NOBOX oogenesis homeobox
1.09	MYO9B	myosin IXB
1.09	ADGRF3	adhesion G protein-coupled receptor F3
1.09	CUL2	cullin 2
1.09	ACTL10	actin-like 10
1.09	POGZ	Transcript Identified by AceView. Entrez Gene ID(s) 23126
1.09	RGS22	regulator of G-protein signaling 22
1.09	ZSCAN29	zinc finger and SCAN domain containing 29
1.09	RPS17	ribosomal protein S17
1.09	DDX3X	DEAD (Asp-Glu-Ala-Asp) box helicase 3. X-linked
1.09	NUCKS1	nuclear casein kinase and cyclin-dependent kinase substrate 1
1.09	SOX10	SRY box 10
1.09	TMX3	thioredoxin-related transmembrane protein 3
1.09	IL1RAP	interleukin 1 receptor accessory protein
1.09	MAN1A2	mannosidase. alpha. class 1A. member 2
1.09	CIITA	class II. major histocompatibility complex. transactivator
1.09	CPSF3	cleavage and polyadenylation specific factor 3
1.09	SPRR2B	small proline-rich protein 2B
1.09	AOAH	acyloxyacyl hydrolase (neutrophil)
1.09	PVALB	parvalbumin
1.09	GIT1	G protein-coupled receptor kinase interacting ArfGAP 1
1.09	KRTAP11-1	keratin associated protein 11-1

1.09	SLCO1B3	solute carrier organic anion transporter family. member 1B3
1.09	ZNF749	zinc finger protein 749
1.09	NKX2-4	NK2 homeobox 4
1.09	VAMP7	Homo sapiens vesicle-associated membrane protein 7 (VAMP7). transcript variant 2. mRNA.; Homo sapiens vesicle-associated membrane protein 7 (VAMP7). transcript variant 3. mRNA.; Homo sapiens vesicle-associated membrane protein 7 (VAMP7). transcript variant 1. mRNA.; Homo sapiens vesicle-associated membrane protein 7 (VAMP7). transcript variant 4. non-coding RNA.; Homo sapiens vesicle-associated membrane protein 7 (VAMP7). transcript variant 5. non-coding RNA.; vesicle-associated membrane protein 7 [Source:HGNC Symbol;Acc:HGNC:11486]; Homo sapiens vesicle-associated membrane protein 7. mRNA (cDNA clone MGC:64832 IMAGE:6503665). complete cds.
1.09	GPSM3	G-protein signaling modulator 3
1.09	RXFP3	relaxin/insulin-like family peptide receptor 3
1.09	SLC14A2	solute carrier family 14 (urea transporter). member 2
1.09	ERICH5	glutamate rich 5
1.09	LHFPL5	lipoma HMGIC fusion partner-like 5
1.09	SMN1	survival of motor neuron 1. telomeric
1.09	ATG4D	autophagy related 4D. cysteine peptidase
1.09	MYBBP1A	MYB binding protein (P160) 1a
1.09	ZNF791	zinc finger protein 791
1.09	CEP170B	centrosomal protein 170B
1.09	EEFSEC	Memczak2013 ALT_ACCEPTOR. ALT_DONOR. coding. INTERNAL. intronic best transcript NM_021937
1.09	ZBTB24	zinc finger and BTB domain containing 24
1.09	PPARGC1A	peroxisome proliferator-activated receptor gamma. coactivator 1 alpha
1.09	ADAMTSL5	ADAMTS like 5
1.09	CYP4B1	cytochrome P450. family 4. subfamily B. polypeptide 1

1.09	SUCLG2	succinate-CoA ligase. GDP-forming. beta subunit
1.09	CD274	CD274 molecule
1.09	OR5AC1	olfactory receptor. family 5. subfamily AC. member 1 (gene/pseudogene)
1.09	HGSNAT	Transcript Identified by AceView. Entrez Gene ID(s) 138050
1.09	KDM4B	lysine (K)-specific demethylase 4B
1.09	SPDYE3	speedy/RINGO cell cycle regulator family member E3
1.09	THAP1	THAP domain containing. apoptosis associated protein 1
1.09	CFHR3	complement factor H-related 3
1.09	ZRANB1	zinc finger. RAN-binding domain containing 1
1.09	KIR2DS5; KIR3DL2; KIR2DL1; KIR3DL3; KIR3DL1; KIR2DS4; KIR2DL3; KIR3DS1; KIR2DS2; KIR2DP1; KIR3DP1	killer cell immunoglobulin-like receptor. two domains. short cytoplasmic tail. 5; killer cell immunoglobulin-like receptor. three domains. long cytoplasmic tail. 2; killer cell immunoglobulin-like receptor. two domains. long cytoplasmic tail. 1; killer cell immunoglobulin-like receptor. three domains. long cytoplasmic tail. 3; killer cell immunoglobulin-like receptor. three domains. long cytoplasmic tail. 1; killer cell immunoglobulin-like receptor. two domains. short cytoplasmic tail. 4; killer cell immunoglobulin-like receptor. two domains. long cytoplasmic tail. 3; killer cell immunoglobulin-like receptor. three domains. short cytoplasmic tail. 1; killer cell immunoglobulin-like receptor. two domains. short cytoplasmic tail. 2; killer cell immunoglobulin-like receptor. two domains. pseudogene 1; killer cell immunoglobulin-like receptor. three domains. pseudogene 1
1.09	KAZALD1	Kazal-type serine peptidase inhibitor domain 1
1.09	ZNF266	zinc finger protein 266
1.09	OR51Q1	olfactory receptor. family 51. subfamily Q. member 1 (gene/pseudogene)
1.09	OR2M4	olfactory receptor. family 2. subfamily M. member 4
1.09	CLDN18	claudin 18
1.09	RAB22A	RAB22A. member RAS oncogene family
1.09	AGTR2	angiotensin II receptor. type 2
1.09	TMEM51	transmembrane protein 51

1.09	CCDC178	coiled-coil domain containing 178
1.09	DLST	dihydrolipoamide S-succinyltransferase (E2 component of 2-oxo-glutarate complex)
1.09	UGT8	UDP glycosyltransferase 8
1.09	SMCP	sperm mitochondria-associated cysteine-rich protein
1.09	ARL4D	ADP-ribosylation factor like GTPase 4D
1.09	BEX2	brain expressed X-linked 2
1.09	SPINK5	serine peptidase inhibitor. Kazal type 5
1.09	TCEAL5	transcription elongation factor A (SII)-like 5
1.09	VIPR1	vasoactive intestinal peptide receptor 1
1.09	NUP98	Transcript Identified by AceView. Entrez Gene ID(s) 4928
1.09	ITLN1	intelectin 1 (galactofuranose binding)
1.09	OSBPL5	Memczak2013 ALT_ACCEPTOR. ALT_DONOR. coding. INTERNAL. intronic best transcript NM_020896
1.09	H2AFY	H2A histone family. member Y
1.09	TAF6	TAF6 RNA polymerase II. TATA box binding protein (TBP)-associated factor. 80kDa
1.09	DNAJC5B	DnaJ (Hsp40) homolog. subfamily C. member 5 beta
1.09	TMEM259	transmembrane protein 259
1.09	KRT86	keratin 86. type II
1.09	ARHGAP20	Rho GTPase activating protein 20
1.09	APOBR	apolipoprotein B receptor
1.09	MEIG1	meiosis/spermiogenesis associated 1
1.09	RPL41	Synthetic construct Homo sapiens clone IMAGE:100063377. MGC:190767 ribosomal protein L41 (RPL41) mRNA. encodes complete protein.
1.09	PPIL2	peptidylprolyl isomerase (cyclophilin)-like 2
1.09	SLC6A3	solute carrier family 6 (neurotransmitter transporter). member 3

1.09	AMFR	autocrine motility factor receptor. E3 ubiquitin protein ligase
1.09	NXF2B; NXF2	nuclear RNA export factor 2B; nuclear RNA export factor 2
1.09	RHD	Rh blood group. D antigen
1.09	STXBP3	syntaxin binding protein 3
1.09	AP5M1	adaptor-related protein complex 5. mu 1 subunit
1.09	TPR	translocated promoter region. nuclear basket protein
1.09	MLH3	mutL homolog 3
1.09	CPA4	carboxypeptidase A4
1.09	CTC-453G23.8; CARD8; nekera	Memczak2013 ANTISENSE. CDS. coding. INTERNAL best transcript NM_014959; Transcript Identified by AceView; novel transcript. antisense to CARD8
1.09	IFNAR1	interferon (alpha. beta and omega) receptor 1
1.09	MAP3K9	mitogen-activated protein kinase kinase kinase 9
1.09	SIAH2	siah E3 ubiquitin protein ligase 2
1.09	OR51B6	olfactory receptor. family 51. subfamily B. member 6
1.09	VMO1	vitelline membrane outer layer 1 homolog (chicken)
1.09	HPCAL4	hippocalcin like 4
1.09	TTBK2	tau tubulin kinase 2
1.09	SLC35E3	solute carrier family 35. member E3
1.09	REXO4	REX4 homolog. 3'-5' exonuclease [Source:HGNC Symbol;Acc:HGNC:12820]
1.09	ATF2	activating transcription factor 2
1.09	LOC100131496; RP4-569M23.5	uncharacterized LOC100131496; novel transcript. antisense to ZMYND8
1.09	TCEB3B	transcription elongation factor B polypeptide 3B (elongin A2)
1.09	C14orf2	chromosome 14 open reading frame 2
1.09	RAD21	Transcript Identified by AceView. Entrez Gene ID(s) 5885
1.09	NDUFA4L2	NADH dehydrogenase (ubiquinone) 1 alpha subcomplex. 4-like 2
1.09	TEFM	transcription elongation factor. mitochondrial

1.09	SHBG	sex hormone-binding globulin
1.09	GALNT15	polypeptide N-acetylgalactosaminyltransferase 15
1.09	SERTAD4	SERTA domain containing 4
1.09	ABCC9	ATP binding cassette subfamily C member 9
1.09	KDM6A	lysine (K)-specific demethylase 6A
1.09	TACR3	tachykinin receptor 3
1.09	MTHFSD	methenyltetrahydrofolate synthetase domain containing
1.09	MBD3L5	methyl-CpG binding domain protein 3-like 5
1.09	ASB3	ankyrin repeat and SOCS box containing 3
1.09	NRP2	neuropilin 2
1.09	BACH2	Memczak2013 ALT_ACCEPTOR. ALT_DONOR. coding. INTERNAL. intronic best transcript NM_001170794
1.09	OR4K14	olfactory receptor. family 4. subfamily K. member 14
1.09	TMEM25	transmembrane protein 25
1.09	CPO	carboxypeptidase O
1.09	MFSD2B	major facilitator superfamily domain containing 2B
1.09	FCN3	ficolin (collagen/fibrinogen domain containing) 3
1.09	CLHC1	clathrin heavy chain linker domain containing 1
1.09	CNIH3; RP11-449J1.1	Transcript Identified by AceView. Entrez Gene ID(s) 149111; novel transcript
1.09	CFAP53	cilia and flagella associated protein 53
1.09	TNFRSF25	tumor necrosis factor receptor superfamily. member 25
1.09	DRD3	dopamine receptor D3
1.09	MRVI1-AS1	MRVI1 antisense RNA 1
1.09	DUSP18	dual specificity phosphatase 18
1.09	MAGEA6	MAGE family member A6
1.09	NPIPA3	nuclear pore complex interacting protein family. member A3

1.09	GPR6	G protein-coupled receptor 6
1.09	PLXDC2	plexin domain containing 2
1.09	SLC7A4	solute carrier family 7. member 4
1.09	KRTAP16-1	keratin associated protein 16-1
1.09	GABARAP	GABA(A) receptor-associated protein
1.09	WBP4	Transcript Identified by AceView. Entrez Gene ID(s) 11193
1.09	DBF4	Transcript Identified by AceView. Entrez Gene ID(s) 10926
1.09	KIAA1211L	KIAA1211-like
1.09	ABT1	activator of basal transcription 1
1.09	CHRNB3	cholinergic receptor. nicotinic beta 3
1.09	FOXJ3	forkhead box J3
1.09	SLC18A1	solute carrier family 18 (vesicular monoamine transporter). member 1
1.09	SAG	S-antigen; retina and pineal gland (arrestin)
1.09	SHANK1	SH3 and multiple ankyrin repeat domains 1
1.09	GGTLC2	gamma-glutamyltransferase light chain 2
1.09	DDX23	DEAD (Asp-Glu-Ala-Asp) box polypeptide 23
1.09	RRAS	related RAS viral (r-ras) oncogene homolog
1.09	ARL11	ADP-ribosylation factor like GTPase 11
1.09	COPS4	COP9 signalosome subunit 4
1.09	TLR1	toll-like receptor 1
1.09	DOCK2	dedicator of cytokinesis 2
1.09	SH3BGRL3	SH3 domain binding glutamate-rich protein like 3
1.09	NOA1	nitric oxide associated 1
1.09	MMP15	matrix metallopeptidase 15 (membrane-inserted)
1.09	NUP93	nucleoporin 93kDa
1.09	ECSCR	endothelial cell surface expressed chemotaxis and apoptosis regulator

1.09	ZDHHC3	zinc finger. DHHC-type containing 3
1.09	KRT74	keratin 74. type II
1.09	OSBPL10	oxysterol binding protein-like 10
1.09	MYO16	myosin XVI
1.09	BPIFB4	BPI fold containing family B. member 4
1.09	CDH16	cadherin 16. KSP-cadherin
1.09	TAS2R14	taste receptor. type 2. member 14
1.09	HACL1	2-hydroxyacyl-CoA lyase 1
1.09	ATP13A4	ATPase type 13A4
1.09	OR52W1	olfactory receptor. family 52. subfamily W. member 1
1.09	ROBO4	roundabout guidance receptor 4
1.09	NRCAM	neuronal cell adhesion molecule
1.09	SPCS2	signal peptidase complex subunit 2
1.09	FJX1	four jointed box 1
1.09	PABPC1L2B	poly(A) binding protein. cytoplasmic 1-like 2B
1.09	TVP23A	trans-golgi network vesicle protein 23 homolog A (S. cerevisiae)
1.09	EIF5A2	eukaryotic translation initiation factor 5A2
1.09	RBMXL2	RNA binding motif protein. X-linked-like 2
1.09	C2CD2	C2 calcium-dependent domain containing 2
1.09	KRT8	keratin 8. type II
1.09	MAGEC2	MAGE family member C2
1.09	ST6GALNAC4	ST6 (alpha-N-acetyl-neuraminyl-2.3-beta-galactosyl-1.3)-N-acetylgalactosaminide alpha-2.6-sialyltransferase 4
1.09	GRIA2	Transcript Identified by AceView. Entrez Gene ID(s) 2891
1.09	CFAP54	cilia and flagella associated 54
1.09	HIC1	hypermethylated in cancer 1

1.09	HERC3	HECT and RLD domain containing E3 ubiquitin protein ligase 3
1.09	AHCYL2	adenosylhomocysteinase-like 2
1.09	NOV	nephroblastoma overexpressed
1.09	SEMA3E	sema domain. immunoglobulin domain (Ig). short basic domain. secreted. (semaphorin) 3E
1.09	REM2	RAS (RAD and GEM)-like GTP binding 2
1.09	IFNB1	interferon. beta 1. fibroblast
1.09	ORC3	origin recognition complex subunit 3
1.09	CXCR6	chemokine (C-X-C motif) receptor 6
1.09	NLRP2	NLR family. pyrin domain containing 2
1.09	RPL14	ribosomal protein L14
1.09	EFNA5	ephrin-A5
1.09	ATG3	autophagy related 3
1.09	HMBS	hydroxymethylbilane synthase
1.09	CBS	cystathionine-beta-synthase
1.09	TRABD2A	TraB domain containing 2A
1.09	ZNF780A	zinc finger protein 780A
1.09	AK1	adenylate kinase 1
1.09	OGFOD1	2-oxoglutarate and iron-dependent oxygenase domain containing 1
1.09	SPATA45	spermatogenesis associated 45
1.09	C7orf76; SHFM1	chromosome 7 open reading frame 76; split hand/foot malformation (ectrodactyly) type 1
1.09	ELP3	elongator acetyltransferase complex subunit 3
1.09	MLXIP	MLX interacting protein
1.09	BIRC2	baculoviral IAP repeat containing 2
1.09	ATP2A1	ATPase. Ca++ transporting. cardiac muscle. fast twitch 1
1.09	ZNF474	zinc finger protein 474

1.09	LCN6	lipocalin 6
1.09	C3orf14	chromosome 3 open reading frame 14
1.09	BDKRB1	bradykinin receptor B1
1.09	CDK15	cyclin-dependent kinase 15
1.09	CNTN4	contactin 4
1.09	SNX15	sorting nexin 15
1.09	PDS5B	Memczak2013 ALT_ACCEPTOR. ALT_DONOR. coding. INTERNAL. intronic best transcript NM_015032
1.09	DDIT4	DNA damage inducible transcript 4
1.09	NFIC	nuclear factor I/C (CCAAT-binding transcription factor)
1.09	OR1I1	olfactory receptor. family 1. subfamily I. member 1
1.09	GPR75-ASB3	GPR75-ASB3 readthrough
1.09	ZNF592	zinc finger protein 592
1.09	FAM217B	family with sequence similarity 217. member B
1.09	CIRBP	cold inducible RNA binding protein
1.09	CACFD1	calcium channel flower domain containing 1
1.09	SLC1A4	solute carrier family 1 (glutamate/neutral amino acid transporter). member 4
1.09	PTTG2	pituitary tumor-transforming 2
1.09	HCAR3	hydroxycarboxylic acid receptor 3
1.09	GRINA	glutamate receptor. ionotropic. N-methyl D-aspartate-associated protein 1 (glutamate binding)
1.09	PLAU	plasminogen activator. urokinase
1.09	NCK1	NCK adaptor protein 1
1.09	SPTB; MIR7855	spectrin. beta. erythrocytic; microRNA 7855
1.09	ZSCAN16	zinc finger and SCAN domain containing 16
1.09	MAML3	mastermind-like transcriptional coactivator 3

1.09	NSUN7	NOP2/Sun domain family. member 7
1.09	TM7SF3	transmembrane 7 superfamily member 3
1.09	PM20D2	peptidase M20 domain containing 2
1.09	POFUT2	protein O-fucosyltransferase 2
1.09	RPL7A; SNORD36C; SNORD36B; SNORD24; SNORD36A	ribosomal protein L7a; small nucleolar RNA. C/D box 36C; small nucleolar RNA. C/D box 36B; small nucleolar RNA. C/D box 24; small nucleolar RNA. C/D box 36A
1.09	PHC1	Homo sapiens polyhomeotic homolog 1 (Drosophila). mRNA (cDNA clone MGC:87926 IMAGE:5788132). complete cds.; Homo sapiens polyhomeotic homolog 1 (Drosophila). mRNA (cDNA clone MGC:189745 IMAGE:9057069). complete cds.
1.09	SLX1A-SULT1A3	SLX1A-SULT1A3 readthrough (NMD candidate)
1.09	GIMAP6	GTPase. IMAP family member 6
1.09	ERICH2	glutamate rich 2
1.09	PSMD9	proteasome 26S subunit. non-ATPase 9
1.09	C11orf63	chromosome 11 open reading frame 63
1.09	KCNMA1; swamu	Jeck2013 ALT_ACCEPTOR. ALT_DONOR. coding. INTERNAL. intronic best transcript NM_001014797; Transcript Identified by AceView
1.09	TBX15	T-box 15
1.09	SRI	sorcin
1.09	ADAMTS1	ADAM metallopeptidase with thrombospondin type 1 motif 1
1.09	COL3A1; MIR3606	collagen. type III. alpha 1; microRNA 3606
1.09	FAIM2	Fas apoptotic inhibitory molecule 2
1.09	TTC9C	tetratricopeptide repeat domain 9C
1.09	C1QBP	complement component 1. q subcomponent binding protein
1.09	UMODL1	uromodulin-like 1
1.09	NUTM2B	NUT family member 2B
1.09	WNT6	wingless-type MMTV integration site family. member 6

1.09	PANK2	pantothenate kinase 2
1.09	DFFA	DNA fragmentation factor. 45kDa. alpha polypeptide
1.09	SOST	sclerostin
1.09	DNAAF1	dynein. axonemal. assembly factor 1
1.09	GDF10	growth differentiation factor 10
1.09	ROBO3	roundabout guidance receptor 3
1.09	OR2H1	olfactory receptor. family 2. subfamily H. member 1
1.09	AGPAT5	1-acylglycerol-3-phosphate O-acyltransferase 5
1.09	IL27RA	interleukin 27 receptor. alpha
1.09	RGMA	repulsive guidance molecule family member a
1.09	PIH1D1	PIH1 domain containing 1
1.09	FBXO8	Transcript Identified by AceView. Entrez Gene ID(s) 26269
1.09	IFNA6	interferon. alpha 6
1.09	E2F7	E2F transcription factor 7
1.09	KCNMB2	potassium channel subfamily M regulatory beta subunit 2
1.09	DRC7	dynein regulatory complex subunit 7
1.09	VANGL2	VANGL planar cell polarity protein 2
1.09	RAP2A	RAP2A. member of RAS oncogene family
1.08	PTPRE	protein tyrosine phosphatase. receptor type. E
1.08	PI4K2A	phosphatidylinositol 4-kinase type 2 alpha
1.08	FLNC	filamin C. gamma
1.08	SMIM10L2A	small integral membrane protein 10 like 2A
1.08	PHF19	Transcript Identified by AceView. Entrez Gene ID(s) 26147
1.08	AQR	aquarius intron-binding spliceosomal factor
1.08	LSM12	Homo sapiens LSM12 homolog (S. cerevisiae). mRNA (cDNA clone MGC:57206 IMAGE:4794614). complete cds.

1.08	DDX43	DEAD (Asp-Glu-Ala-Asp) box polypeptide 43
1.08	SLC25A39	solute carrier family 25. member 39
1.08	MAPK12	mitogen-activated protein kinase 12
1.08	MAGEC1	MAGE family member C1
1.08	TMEM266	transmembrane protein 266
1.08	KLHL11	kelch-like family member 11
1.08	TCF20	transcription factor 20 (AR1)
1.08	TCF20	transcription factor 20 (AR1)
1.08	PSMF1	proteasome inhibitor subunit 1
1.08	XKRY2; XKRY	Homo sapiens XK. Kell blood group complex subunit-related. Y-linked 2 (XKRY2). mRNA.; Homo sapiens XK. Kell blood group complex subunit-related. Y-linked (XKRY). mRNA.; Homo sapiens XK. Kell blood group complex subunit-related. Y-linked. mRNA (cDNA clone MGC:164514 IMAGE:40146905). complete cds.; Homo sapiens XK. Kell blood group complex subunit-related. Y-linked. mRNA (cDNA clone MGC:164516 IMAGE:40146907). complete cds.; Homo sapiens XK. Kell blood group complex subunit-related. Y-linked 2. mRNA (cDNA clone MGC:164532 IMAGE:40146923). complete cds.; Homo sapiens XK. Kell blood group complex subunit-related. Y-linked 2. mRNA (cDNA clone MGC:164534 IMAGE:40146925). complete cds.; Synthetic construct Homo sapiens clone IMAGE:100015836. MGC:183189 XK. Kell blood group complex subunit-related. Y-linked 2 (XKRY2) mRNA. encodes complete protein.; Synthetic construct Homo sapiens clone IMAGE:100016528. MGC:184271 XK. Kell blood group complex subunit-related. Y-linked (XKRY) mRNA. encodes complete protein.; Transcript Identified by AceView. Entrez Gene ID(s) 353515. RefSeq ID(s) NM_004677. NM_001002906
1.08	GAGE12F; GAGE12J; GAGE12D; GAGE5; GAGE6; GAGE12B; GAGE4; GAGE2E	G antigen 12F; G antigen 12J; G antigen 12D; G antigen 5; G antigen 6; G antigen 12B; G antigen 4; G antigen 2E
1.08	FAM133B	Transcript Identified by AceView. Entrez Gene ID(s) 257415
1.08	PDS5A	PDS5 cohesin associated factor A
1.08	FBXL17	F-box and leucine-rich repeat protein 17

1.08	MAN2C1	mannosidase. alpha. class 2C. member 1
1.08	SULT1C3	sulfotransferase family 1C member 3
1.08	COQ5	coenzyme Q5. methyltransferase
1.08	BRMS1	breast cancer metastasis suppressor 1
1.08	GREM1	gremlin 1. DAN family BMP antagonist [Source:HGNC Symbol;Acc:HGNC:2001]
1.08	ADRBK2	adrenergic. beta. receptor kinase 2
1.08	CFAP157	cilia and flagella associated protein 157
1.08	ZNF7	zinc finger protein 7
1.08	KRTAP5-8	keratin associated protein 5-8
1.08	LCNL1	lipocalin-like 1
1.08	ERCC6L	excision repair cross-complementation group 6-like
1.08	CCDC172	coiled-coil domain containing 172
1.08	AFTPH	aftiphilin
1.08	A2BP1	Transcript Identified by AceView. Entrez Gene ID(s) 54715
1.08	OLA1	Obg-like ATPase 1
1.08	IDO1	indoleamine 2.3-dioxygenase 1
1.08	HOXA10; HOXA9; MIR196B	homeobox A10; homeobox A9; microRNA 196b
1.08	LGI3	leucine-rich repeat LGI family. member 3
1.08	TBCC	tubulin folding cofactor C
1.08	ORAI1	ORAI calcium release-activated calcium modulator 1
1.08	ST3GAL2	ST3 beta-galactoside alpha-2.3-sialyltransferase 2
1.08	CRTAP	cartilage associated protein
1.08	FLT1	fms-related tyrosine kinase 1
1.08	MLLT3	myeloid/lymphoid or mixed-lineage leukemia; translocated to. 3
1.08	MAGEA1	MAGE family member A1

1.08	FEM1C	Jeck2013 ALT_ACCEPTOR. ALT_DONOR. coding. INTERNAL. intronic best transcript NM_020177
1.08	RPL41	Synthetic construct Homo sapiens clone IMAGE:100063377. MGC:190767 ribosomal protein L41 (RPL41) mRNA. encodes complete protein.; 60S ribosomal protein L41 [Source:UniProtKB/Swiss-Prot;Acc:P62945]
1.08	KCNK12	potassium channel. two pore domain subfamily K. member 12
1.08	TRMT10C	tRNA methyltransferase 10C. mitochondrial RNase P subunit
1.08	SETMAR	SET domain and mariner transposase fusion gene
1.08	UBE2G2	ubiquitin conjugating enzyme E2G 2
1.08	CATSPER4	cation channel. sperm associated 4
1.08	JRK	Jrk helix-turn-helix protein
1.08	CCT3	chaperonin containing TCP1. subunit 3 (gamma)
1.08	PPY	pancreatic polypeptide
1.08	RPL10; SNORA70	ribosomal protein L10; small nucleolar RNA. H/ACA box 70
1.08	STXBP6	syntaxin binding protein 6 (amisyn)
1.08	LILRB2	leukocyte immunoglobulin-like receptor. subfamily B (with TM and ITIM domains). member 2
1.08	C12orf42	chromosome 12 open reading frame 42
1.08	SERPINB2	serpin peptidase inhibitor. clade B (ovalbumin). member 2
1.08	SMARCE1	SWI/SNF related. matrix associated. actin dependent regulator of chromatin. subfamily e. member 1
1.08	WNT7B	wingless-type MMTV integration site family. member 7B
1.08	SNX1	sorting nexin 1
1.08	GCSAML	germinal center-associated. signaling and motility-like
1.08	C4orf22	chromosome 4 open reading frame 22
1.08	LMNB2	Zhang2013 ALT_ACCEPTOR. ALT_DONOR. coding. INTERNAL. intronic best transcript NM_032737
1.08	TAP1	transporter 1. ATP-binding cassette. sub-family B (MDR/TAP)

1.08	SLC6A4	solute carrier family 6 (neurotransmitter transporter). member 4
1.08	PRY2; PRY	PTPN13-like. Y-linked 2; PTPN13-like. Y-linked
1.08	FMR1NB	fragile X mental retardation 1 neighbor
1.08	LEMD2	LEM domain containing 2
1.08	ZDHHC3	zinc finger. DHHC-type containing 3
1.08	SPINK13	serine peptidase inhibitor. Kazal type 13 (putative)
1.08	CPXCR1	CPX chromosome region. candidate 1
1.08	NPAS1	neuronal PAS domain protein 1
1.08	NRDC; MIR761	nardilysin convertase; microRNA 761
1.08	C12orf50	chromosome 12 open reading frame 50
1.08	VN1R1	vomeronasal 1 receptor 1
1.08	PLET1	placenta expressed transcript 1
1.08	CHIC1	cysteine rich hydrophobic domain 1
1.08	ZNF806	zinc finger protein 806
1.08	TAS2R39	taste receptor. type 2. member 39
1.08	SND1; SND1-IT1	staphylococcal nuclease and tudor domain containing 1; SND1 intronic transcript 1
1.08	CCDC85A	coiled-coil domain containing 85A
1.08	DSEL	dermatan sulfate epimerase-like
1.08	UMOD	uromodulin
1.08	USP17L22; USP17L20	ubiquitin specific peptidase 17-like family member 22; ubiquitin specific peptidase 17-like family member 20
1.08	COL8A2	collagen. type VIII. alpha 2
1.08	P2RY10	purinergic receptor P2Y. G-protein coupled. 10
1.08	TPM4	tropomyosin 4
1.08	SSPO	SCO-spondin
1.08	MTA3	Transcript Identified by AceView. Entrez Gene ID(s) 57504

1.08	TAS2R40	taste receptor. type 2. member 40
1.08	RNF141	ring finger protein 141
1.08	ZNF274	zinc finger protein 274
1.08	NPIPA2	nuclear pore complex interacting protein family. member A2
1.08	NDUFA10	NADH dehydrogenase (ubiquinone) 1 alpha subcomplex. 10. 42kDa
1.08	NXPH1	neurexophilin 1
1.08	OLR1	oxidized low density lipoprotein (lectin-like) receptor 1
1.08	ARHGEF33	Rho guanine nucleotide exchange factor 33
1.08	IL17REL	interleukin 17 receptor E-like
1.08	TMEM117	transmembrane protein 117
1.08	PTDSS1	phosphatidylserine synthase 1
1.08	ALG8	ALG8. alpha-1.3-glucosyltransferase
1.08	OR7A17	olfactory receptor. family 7. subfamily A. member 17
1.08	NFRKB	nuclear factor related to kappaB binding protein
1.08	EVA1A	eva-1 homolog A (C. elegans)
1.08	ARVCF	Zhang2013 ALT_ACCEPTOR. ALT_DONOR. coding. INTERNAL. intronic best transcript NM_001670
1.08	B4GALT3	UDP-Gal:betaGlcNAc beta 1.4- galactosyltransferase. polypeptide 3
1.08	MED8	mediator complex subunit 8
1.08	C20orf197	chromosome 20 open reading frame 197
1.08	PHF19	PHD finger protein 19
1.08	GPSM1	G-protein signaling modulator 1
1.08	HTN1	histatin 1
1.08	CA12	carbonic anhydrase XII
1.08	OR6N2	olfactory receptor. family 6. subfamily N. member 2
1.08	CFAP45	cilia and flagella associated protein 45

1.08	OGFOD2	2-oxoglutarate and iron-dependent oxygenase domain containing 2
1.08	FMN1	formin 1
1.08	ITPRIPL1	inositol 1.4.5-trisphosphate receptor interacting protein-like 1
1.08	SPTLC2	serine palmitoyltransferase. long chain base subunit 2
1.08	ADAMTS9	ADAM metallopeptidase with thrombospondin type 1 motif 9
1.08	C3orf84	chromosome 3 open reading frame 84
1.08	ITGB3BP	integrin beta 3 binding protein (beta3-endonexin)
1.08	ULK3	unc-51 like kinase 3
1.08	TMEM215	transmembrane protein 215
1.08	PRAMEF7; PRAMEF8	PRAME family member 7; PRAME family member 8
1.08	ANKRD34C	ankyrin repeat domain 34C
1.08	KMO	kynurenine 3-monooxygenase (kynurenine 3-hydroxylase)
1.08	CNR1	cannabinoid receptor 1 (brain)
1.08	SDCCAG8	serologically defined colon cancer antigen 8
1.08	ARMC10	armadillo repeat containing 10
1.08	TMEM176B	transmembrane protein 176B
1.08	USP32P2; FAM106A; CCDC144B	ubiquitin specific peptidase 32 pseudogene 2; family with sequence similarity 106. member A; coiled-coil domain containing 144B (pseudogene)
1.08	PLCB2	phospholipase C. beta 2
1.08	NR1H2	nuclear receptor subfamily 1. group H. member 2
1.08	ADCYAP1R1	adenylate cyclase activating polypeptide 1 (pituitary) receptor type I
1.08	STYXL1	serine/threonine/tyrosine interacting-like 1
1.08	SCGB1D2	secretoglobin. family 1D. member 2
1.08	LOC103611081; LOC100507431; RP11-890B15.3; RP11-890B15.2; klerglaw	uncharacterized LOC103611081; uncharacterized LOC100507431; Transcript Identified by AceView; novel transcript

1.08	MAGEA10	MAGE family member A10
1.08	TBR1	T-box. brain. 1
1.08	SHTN1	shootin 1
1.08	C10orf99	chromosome 10 open reading frame 99
1.08	FSCN3	fascin actin-bundling protein 3. testicular
1.08	SFTPC	surfactant protein C
1.08	MRGPRX2	MAS-related GPR. member X2
1.08	SLC35B4	solute carrier family 35 (UDP-xylose/UDP-N-acetylglucosamine transporter). member B4
1.08	NPBWR2	neuropeptides B/W receptor 2
1.08	TG	thyroglobulin
1.08	RPARP-AS1	RPARP antisense RNA 1
1.08	SLC25A21	solute carrier family 25 (mitochondrial oxoadipate carrier). member 21
1.08	THUMPD3	THUMP domain containing 3
1.08	RAB11A	RAB11A. member RAS oncogene family
1.08	NAMPT	nicotinamide phosphoribosyltransferase
1.08	CMTM6	CKLF-like MARVEL transmembrane domain containing 6
1.08	PIGK	phosphatidylinositol glycan anchor biosynthesis class K
1.08	TBCA	tubulin folding cofactor A
1.08	DNAH5	dynein. axonemal. heavy chain 5
1.08	KATNB1	katanin p80 (WD repeat containing) subunit B 1
1.08	ING3	inhibitor of growth family member 3
1.08	LOC101928694; RP11-355N15.1	uncharacterized LOC101928694; novel transcript. antisense to LIPC
1.08	CDKN2AIP	CDKN2A interacting protein
1.08	GJC1	gap junction protein gamma 1
1.08	GPRC5D	G protein-coupled receptor. class C. group 5. member D

1.08	AXIN2	axin 2
1.08	DCLK3	doublecortin-like kinase 3
1.08	SLC22A20	solute carrier family 22. member 20
1.08	PPP1R14C	protein phosphatase 1. regulatory (inhibitor) subunit 14C
1.08	VAMP2	vesicle associated membrane protein 2
1.08	FNDC3A	fibronectin type III domain containing 3A
1.08	GPATCH2L	G-patch domain containing 2 like
1.08	TTC14	tetratricopeptide repeat domain 14
1.08	BTBD18	BTB (POZ) domain containing 18
1.08	TFF1	trefoil factor 1
1.08	KIAA0226L	KIAA0226-like
1.08	PDGFB	platelet-derived growth factor beta polypeptide
1.08	TMEM200A	transmembrane protein 200A
1.08	KAAG1	kidney associated antigen 1
1.08	KDELC2	KDEL (Lys-Asp-Glu-Leu) containing 2
1.08	UHRF1BP1	UHRF1 binding protein 1
1.08	HGH1	HGH1 homolog
1.08	EP400; SNORA49	E1A binding protein p400; small nucleolar RNA. H/ACA box 49
1.08	ZNF333	zinc finger protein 333
1.08	NLRP14	NLR family. pyrin domain containing 14
1.08	PRAMEF4	PRAME family member 4
1.08	ZNF501	zinc finger protein 501 [Source:HGNC Symbol;Acc:HGNC:23717]
1.08	PCGF2	polycomb group ring finger 2
1.08	MPI	mannose phosphate isomerase
1.08	PIGC	phosphatidylinositol glycan anchor biosynthesis class C
1.08	BSPRY	B-box and SPRY domain containing

1.08	ANKRD46	ankyrin repeat domain 46
1.08	IGIP	IgA-inducing protein
1.08	PGS1	phosphatidylglycerophosphate synthase 1
1.08	SNAP47	synaptosome associated protein 47kDa
1.08	GAS1	growth arrest-specific 1
1.08	LOC79999; LOC388436; RP11-744A16.4	uncharacterized LOC79999; Homo sapiens uncharacterized protein ENSP00000382042 (LOC388436). mRNA.; novel transcript
1.08	PIAS3	protein inhibitor of activated STAT 3
1.08	PSMD10	proteasome 26S subunit. non-ATPase 10
1.08	RAB33B	RAB33B. member RAS oncogene family
1.08	HSPE1-MOB4	HSPE1-MOB4 readthrough
1.08	NCF1	neutrophil cytosolic factor 1
1.08	OR10A5	olfactory receptor. family 10. subfamily A. member 5
1.08	ANKAR	ankyrin and armadillo repeat containing
1.08	KAZN	kazrin. periplakin interacting protein
1.08	FADD	Fas (TNFRSF6)-associated via death domain
1.08	IAPP	islet amyloid polypeptide
1.08	ZNF527	zinc finger protein 527
1.08	HIST1H1T	histone cluster 1. H1t
1.08	ADH7	alcohol dehydrogenase 7 (class IV). mu or sigma polypeptide
1.08	SLC35D3	solute carrier family 35. member D3
1.08	C9orf142	chromosome 9 open reading frame 142
1.08	CCDC140	coiled-coil domain containing 140
1.08	OR6C65	olfactory receptor. family 6. subfamily C. member 65
1.08	GNE	glucosamine (UDP-N-acetyl)-2-epimerase/N-acetylmannosamine kinase
1.08	C19orf70	chromosome 19 open reading frame 70

1.08	PCBD2	pterin-4 alpha-carbinolamine dehydratase/dimerization cofactor of hepatocyte nuclear factor 1 alpha (TCF1) 2
1.08	PSMA5	proteasome subunit alpha 5
1.08	ZFP69B	ZFP69 zinc finger protein B
1.08	RFTN2	raftlin family member 2
1.08	CBS	cystathionine-beta-synthase
1.08	WDR19	WD repeat domain 19
1.08	C10orf67	chromosome 10 open reading frame 67
1.08	POU1F1	POU class 1 homeobox 1
1.08	LRRC37A5P	leucine rich repeat containing 37. member A5. pseudogene
1.08	S100A4	S100 calcium binding protein A4
1.08	GALNT9	polypeptide N-acetylgalactosaminyltransferase 9
1.08	TMEM184C	transmembrane protein 184C
1.08	ARL6IP6	ADP-ribosylation factor like GTPase 6 interacting protein 6
1.08	TMEM150A	transmembrane protein 150A
1.08	SLC16A1	solute carrier family 16 (monocarboxylate transporter). member 1
1.08	TRUB2	TruB pseudouridine (psi) synthase family member 2
1.08	NUP88	nucleoporin 88kDa
1.08	MIB2	mindbomb E3 ubiquitin protein ligase 2
1.08	DEFB136	defensin. beta 136
1.08	YIF1B	Yip1 interacting factor homolog B (S. cerevisiae)
1.08	C1R	complement component 1. r subcomponent
1.08	DEFB134	defensin. beta 134
1.08	MIR670HG; HSD17B12	MIR670 host gene; hydroxysteroid (17-beta) dehydrogenase 12
1.08	ANKRD13B	ankyrin repeat domain 13B
1.08	METRN	meteorin. glial cell differentiation regulator

1.08	ALS2CL	ALS2 C-terminal like
1.08	PTBP2	Transcript Identified by AceView. Entrez Gene ID(s) 58155
1.08	CTDSPL2	CTD small phosphatase like 2
1.08	DAZL	deleted in azoospermia-like
1.08	EIF4A1; SNORD10; SNORA67; SNORA48	eukaryotic translation initiation factor 4A1; small nucleolar RNA. C/D box 10; small nucleolar RNA. H/ACA box 67; small nucleolar RNA. H/ACA box 48
1.08	RSPH14	radial spoke head 14 homolog (Chlamydomonas)
1.08	CACNG5	calcium channel. voltage-dependent. gamma subunit 5
1.08	OR13C2	olfactory receptor. family 13. subfamily C. member 2
1.08	CA13	carbonic anhydrase XIII
1.08	CRYM	crystallin mu
1.08	ABCB5	ATP binding cassette subfamily B member 5
1.08	TBX1	T-box 1
1.08	C10orf71	chromosome 10 open reading frame 71
1.08	HTR4	5-hydroxytryptamine (serotonin) receptor 4. G protein-coupled
1.08	SETD1B	SET domain containing 1B
1.08	ZNF782	zinc finger protein 782
1.08	TMEM260	transmembrane protein 260
1.08	ZNF274	Memczak2013 ANTISENSE. CDS. coding. INTERNAL best transcript NM_133502
1.08	ARL13B	ADP-ribosylation factor like GTPase 13B
1.08	ZNF620	zinc finger protein 620
1.08	MCTS1	malignant T-cell amplified sequence 1
1.08	NDUFS6	NADH dehydrogenase (ubiquinone) Fe-S protein 6. 13kDa (NADH-coenzyme Q reductase)
1.08	C10orf76	chromosome 10 open reading frame 76

1.08	LOC388242; LOC613038; RP11-345J4.3	SAGA complex associated factor 29 pseudogene; Transcript Identified by AceView. Entrez Gene ID(s) 388242; Uncharacterized protein [Source:UniProtKB/TrEMBL;Acc:H0YHM3]
1.08	GNG12	guanine nucleotide binding protein (G protein). gamma 12
1.08	OR5H14	olfactory receptor. family 5. subfamily H. member 14
1.08	KLHL23	kelch-like family member 23
1.08	TMEM229A	transmembrane protein 229A
1.08	LAMP2	lysosomal-associated membrane protein 2
1.08	FOXF2	forkhead box F2
1.08	GRP	gastrin-releasing peptide
1.08	BCL2L14	BCL2-like 14 (apoptosis facilitator)
1.08	TACR2	tachykinin receptor 2
1.08	CCDC149	coiled-coil domain containing 149
1.08	KRTAP26-1	keratin associated protein 26-1
1.08	OR1L8	olfactory receptor. family 1. subfamily L. member 8
1.08	OR51A4	olfactory receptor. family 51. subfamily A. member 4
1.08	COL28A1	collagen. type XXVIII. alpha 1
1.08	HOXA6	homeobox A6
1.08	ZMIZ2	zinc finger. MIZ-type containing 2
1.08	RPS25	ribosomal protein S25
1.08	GFRAL	GDNF family receptor alpha like
1.08	MCOLN2	mucolipin 2
1.08	SH3PXD2B	SH3 and PX domains 2B
1.08	LINC00222	long intergenic non-protein coding RNA 222
1.08	DDR1; MIR4640	discoidin domain receptor tyrosine kinase 1; microRNA 4640
1.08	IMP4	IMP4 homolog. U3 small nucleolar ribonucleoprotein
1.08	HNRNPK; MIR7-1	heterogeneous nuclear ribonucleoprotein K; microRNA 7-1

1.08	MTHFS	5.10-methenyltetrahydrofolate synthetase (5-formyltetrahydrofolate cyclo-ligase)
1.08	VPS26A	VPS26 retromer complex component A
1.08	CARD10	caspase recruitment domain family. member 10
1.08	RAET1L	retinoic acid early transcript 1L
1.08	OR4Q3	olfactory receptor. family 4. subfamily Q. member 3
1.08	EGR2	early growth response 2
1.08	TMEM171	transmembrane protein 171
1.08	SURF6	surfeit 6
1.08	MEGF11	multiple EGF-like-domains 11
1.08	FAM160B1	family with sequence similarity 160. member B1
1.08	SERHL	serine hydrolase-like (pseudogene)
1.08	RPS25	ribosomal protein S25
1.08	OR1D4	olfactory receptor. family 1. subfamily D. member 4 (gene/pseudogene)
1.08	HDC	histidine decarboxylase
1.08	GRHL3	grainyhead-like transcription factor 3
1.08	ASTE1	asteroid homolog 1 (Drosophila)
1.08	PLEKHF2	pleckstrin homology domain containing. family F (with FYVE domain) member 2
1.08	LCE1B	late cornified envelope 1B
1.08	ZNF548	zinc finger protein 548
1.08	ZNF746	zinc finger protein 746
1.08	HCRP1	Transcript Identified by AceView. Entrez Gene ID(s) 387535
1.08	FPR3	formyl peptide receptor 3
1.08	SUPT4H1	SPT4 homolog. DSIF elongation factor subunit
1.08	RNF220	Memczak2013 ANTISENSE. coding. INTERNAL. intronic best transcript NM_018150

1.08	GTF2IRD2B	GTF2I repeat domain containing 2B
1.08	PLPPR4	phospholipid phosphatase related 4
1.08	MYO5B	myosin VB
1.08	KRT83	keratin 83. type II
1.08	GNL3L	guanine nucleotide binding protein-like 3 (nucleolar)-like
1.08	ANO5	anoctamin 5
1.08	FAM231C; FAM231A	family with sequence similarity 231. member C; family with sequence similarity 231. member A
1.08	FAM126A	family with sequence similarity 126. member A
1.08	COPS6	COP9 signalosome subunit 6
1.08	ZIC2	Zic family member 2
1.08	GRIA2	glutamate receptor. ionotropic. AMPA 2
1.08	HOXD3	homeobox D3
1.08	SSTR2	somatostatin receptor 2
1.08	SUMO2	small ubiquitin-like modifier 2
1.08	CBX4	chromobox homolog 4
1.08	GSPT1	G1 to S phase transition 1
1.08	GDPD2	glycerophosphodiester phosphodiesterase domain containing 2
1.08	C1orf174	chromosome 1 open reading frame 174
1.08	TRIM64	tripartite motif containing 64
1.08	ST3GAL6	ST3 beta-galactoside alpha-2.3-sialyltransferase 6
1.08	KDM5C	lysine (K)-specific demethylase 5C
1.08	C15orf38-AP3S2	C15orf38-AP3S2 readthrough
1.08	FAM131B	family with sequence similarity 131. member B
1.08	ARMC9	armadillo repeat containing 9
1.08	ZIC1	Zic family member 1

1.08	NKG7	natural killer cell granule protein 7
1.08	SMAD4	SMAD family member 4
1.08	SPG20	spastic paraplegia 20 (Troyer syndrome)
1.08	IGSF6	immunoglobulin superfamily. member 6
1.08	OLFM3	olfactomedin 3
1.08	MAB21L1	mab-21-like 1 (C. elegans)
1.08	OR6X1	olfactory receptor. family 6. subfamily X. member 1
1.08	NMRAL1	NmrA-like family domain containing 1
1.08	ZC3H6	zinc finger CCCH-type containing 6
1.08	ZNF48	zinc finger protein 48
1.08	ZNF30	zinc finger protein 30
1.08	C3orf67	chromosome 3 open reading frame 67
1.08	C8orf37	chromosome 8 open reading frame 37
1.08	LIPI	lipase. member I
1.08	CORO7; PAM16; CORO7-PAM16	coronin 7; presequence translocase-associated motor 16 homolog (S. cerevisiae); CORO7-PAM16 readthrough
1.08	REG1B	regenerating islet-derived 1 beta
1.08	ASB18	ankyrin repeat and SOCS box containing 18
1.08	PNPLA5	patatin-like phospholipase domain containing 5
1.08	GCK	glucokinase (hexokinase 4)
1.08	FXYD3; MIR6887	FXYD domain containing ion transport regulator 3; microRNA 6887
1.08	MARK4	MAP/microtubule affinity-regulating kinase 4
1.08	IFI16	interferon. gamma-inducible protein 16
1.08	KCNJ1	potassium channel. inwardly rectifying subfamily J. member 1
1.08	ZNF91	zinc finger protein 91
1.08	MAST2	microtubule associated serine/threonine kinase 2

1.08	CBX1	chromobox homolog 1
1.08	SLC25A26	solute carrier family 25 (S-adenosylmethionine carrier). member 26
1.08	OR5H2	olfactory receptor. family 5. subfamily H. member 2
1.08	CDK5RAP1	CDK5 regulatory subunit associated protein 1
1.08	BCR	breakpoint cluster region
1.08	DIABLO	diablo. IAP-binding mitochondrial protein
1.08	MYL12B	myosin light chain 12B
1.08	ACKR3	atypical chemokine receptor 3
1.08	SLITRK6	SLIT and NTRK-like family. member 6
1.08	IRAK1; MIR718	interleukin 1 receptor associated kinase 1; microRNA 718
1.08	MLNR	motilin receptor
1.08	TPD52L1	tumor protein D52-like 1
1.08	C16orf90	chromosome 16 open reading frame 90
1.08	ATP5G2	ATP synthase. H+ transporting. mitochondrial Fo complex subunit C2 (subunit 9)
1.08	IGFBP7	insulin like growth factor binding protein 7
1.08	UPK2	uroplakin 2
1.08	OR10P1	olfactory receptor. family 10. subfamily P. member 1
1.08	PDE6G	phosphodiesterase 6G. cGMP-specific. rod. gamma
1.08	GALP	galanin-like peptide
1.08	PHKB	phosphorylase kinase. beta
1.08	MAGEB10	MAGE family member B10
1.08	TCF20	transcription factor 20 (AR1)
1.08	TCF20	transcription factor 20 (AR1)
1.08	FAM185A	family with sequence similarity 185. member A
1.08	ORC5	origin recognition complex subunit 5

1.07	HOMER3	homer scaffolding protein 3
1.07	AQP12A	aquaporin 12A
1.07	PRKAR1A	protein kinase. cAMP-dependent. regulatory. type I. alpha
1.07	UBTF	upstream binding transcription factor. RNA polymerase I
1.07	TSPY10	testis specific protein. Y-linked 10
1.07	PJA1	praja ring finger 1. E3 ubiquitin protein ligase
1.07	GINM1	glycoprotein integral membrane 1
1.07	EEF2KMT	eukaryotic elongation factor 2 lysine methyltransferase
1.07	MFAP3	microfibrillar associated protein 3
1.07	HRC	histidine rich calcium binding protein
1.07	EIF3F	Eukaryotic translation initiation factor 3 subunit F [Source:UniProtKB/Swiss-Prot;Acc:O00303]
1.07	MEPE	matrix extracellular phosphoglycoprotein
1.07	MS4A8	membrane-spanning 4-domains. subfamily A. member 8
1.07	IGF2BP2	insulin-like growth factor 2 mRNA binding protein 2
1.07	NDUFB4	NADH dehydrogenase (ubiquinone) 1 beta subcomplex. 4. 15kDa
1.07	FLOT2	flotillin 2
1.07	TRMT61B	tRNA methyltransferase 61B
1.07	SPATA1	spermatogenesis associated 1
1.07	CCDC177	coiled-coil domain containing 177
1.07	ZBTB6	zinc finger and BTB domain containing 6
1.07	ZNF385D	zinc finger protein 385D
1.07	ZPBP	zona pellucida binding protein
1.07	DCC	DCC netrin 1 receptor
1.07	SLC35F3	solute carrier family 35. member F3
1.07	IL6	interleukin 6

1.07	TMEM199; MIR4723	transmembrane protein 199; microRNA 4723
1.07	JARID2	jumonji. AT rich interactive domain 2
1.07	EIF5A	eukaryotic translation initiation factor 5A
1.07	NEFH	neurofilament. heavy polypeptide
1.07	OR10A3	olfactory receptor. family 10. subfamily A. member 3
1.07	MIR17HG; MIR17; MIR18A; MIR19A; MIR19B1; MIR20A; MIR92A1	miR-17-92 cluster host gene; microRNA 17; microRNA 18a; microRNA 19a; microRNA 19b-1; microRNA 20a; microRNA 92a-1
1.07	TSPO	Memczak2013 ANTISENSE. CDS. coding. INTERNAL best transcript NM_000714
1.07	USP15; MIR6125	ubiquitin specific peptidase 15; microRNA 6125
1.07	OR13C5	olfactory receptor. family 13. subfamily C. member 5
1.07	ATL3	atlastin GTPase 3
1.07	DCST2	DC-STAMP domain containing 2
1.07	HAUS2	HAUS augmin like complex subunit 2
1.07	PACRGL	PARK2 co-regulated like
1.07	SNTA1	syntrophin. alpha 1
1.07	SCAMP4; ADAT3	secretory carrier membrane protein 4; adenosine deaminase. tRNA-specific 3
1.07	CHST8	carbohydrate (N-acetylgalactosamine 4-0) sulfotransferase 8
1.07	METTL1	methyltransferase like 1
1.07	PTPN13	Transcript Identified by AceView. Entrez Gene ID(s) 5783
1.07	MAPKAPK2	mitogen-activated protein kinase-activated protein kinase 2
1.07	ATG14	autophagy related 14
1.07	CCDC158	coiled-coil domain containing 158
1.07	STKLD1	serine/threonine kinase-like domain containing 1 [Source:HGNC Symbol;Acc:HGNC:28669]
1.07	MESP2	mesoderm posterior bHLH transcription factor 2

1.07	PRSS2	protease. serine. 2 (trypsin 2)
1.07	CA2	carbonic anhydrase II
1.07	USP17L11; USP17L18; USP17L20	ubiquitin specific peptidase 17-like family member 11; ubiquitin specific peptidase 17-like family member 18; ubiquitin specific peptidase 17-like family member 20
1.07	PRAMEF13	PRAME family member 13
1.07	PRR4	proline rich 4 (lacrimal)
1.07	JAK3	Janus kinase 3
1.07	ALOX5	arachidonate 5-lipoxygenase
1.07	DEFB108B	defensin. beta 108B
1.07	RERG	RAS-like. estrogen-regulated. growth inhibitor
1.07	STRA8	stimulated by retinoic acid 8
1.07	LINC00961	long intergenic non-protein coding RNA 961
1.07	MRPS9	mitochondrial ribosomal protein S9
1.07	CD33	CD33 molecule
1.07	CCDC22	coiled-coil domain containing 22
1.07	LOC101927668; AC005062.2; MACC1	uncharacterized LOC101927668; Transcript Identified by AceView. Entrez Gene ID(s) 346389; novel transcript. sense overlapping to MACC1
1.07	NSUN5	NOP2/Sun domain family. member 5
1.07	PMS1	PMS1 homolog 1. mismatch repair system component
1.07	SLF2	SMC5-SMC6 complex localization factor 2
1.07	USF1	upstream transcription factor 1
1.07	IARS2	isoleucyl-tRNA synthetase 2. mitochondrial
1.07	IL20RB	interleukin 20 receptor beta
1.07	C6orf118	chromosome 6 open reading frame 118
1.07	RBM46	RNA binding motif protein 46
1.07	ILF2	interleukin enhancer binding factor 2

1.07	MARVELD1	MARVEL domain containing 1
1.07	TSPAN18	tetraspanin 18
1.07	SUN3	Sad1 and UNC84 domain containing 3
1.07	GAP43	growth associated protein 43
1.07	G3BP2	GTPase activating protein (SH3 domain) binding protein 2
1.07	PHOX2A	paired-like homeobox 2a
1.07	XKR6	X-linked Kx blood group related 6
1.07	MSH2	mutS homolog 2
1.07	TAS2R50	taste receptor. type 2. member 50
1.07	TMEM184A	transmembrane protein 184A
1.07	JAZF1	Memczak2013 ANTISENSE. coding. INTERNAL. intronic best transcript NM_175061
1.07	HTR3A	5-hydroxytryptamine (serotonin) receptor 3A. ionotropic
1.07	PRCP	prolylcarboxypeptidase
1.07	AGAP1	ArfGAP with GTPase domain. ankyrin repeat and PH domain 1
1.07	OR10C1	olfactory receptor. family 10. subfamily C. member 1 (gene/pseudogene)
1.07	FAM180B	family with sequence similarity 180. member B
1.07	HYOU1	hypoxia up-regulated 1
1.07	COX5A	cytochrome c oxidase subunit Va
1.07	SEC61A1	Sec61 translocon alpha 1 subunit
1.07	DHRS4L1	dehydrogenase/reductase (SDR family) member 4 like 1
1.07	ZNF80	zinc finger protein 80
1.07	VIM	vimentin
1.07	SLITRK1	SLIT and NTRK-like family. member 1
1.07	DAW1	dynein assembly factor with WDR repeat domains 1
1.07	IFI27L2	interferon. alpha-inducible protein 27-like 2

1.07	RGS7	regulator of G-protein signaling 7
1.07	C7orf61	chromosome 7 open reading frame 61
1.07	WDR13	WD repeat domain 13
1.07	RP11-325B23.2; OR5H8	novel transcript; olfactory receptor. family 5. subfamily H. member 8 (gene/pseudogene) [Source:HGNC Symbol;Acc:HGNC:14773]
1.07	TEK	TEK tyrosine kinase. endothelial
1.07	C2orf71	chromosome 2 open reading frame 71
1.07	GTPBP3	GTP binding protein 3 (mitochondrial)
1.07	CFAP58	cilia and flagella associated protein 58
1.07	SPG21	spastic paraplegia 21 (autosomal recessive. Mast syndrome)
1.07	LDAH	lipid droplet associated hydrolase
1.07	RPL41	ribosomal protein L41
1.07	RPS24	ribosomal protein S24
1.07	KRTCAP3	keratinocyte associated protein 3
1.07	ZDHHC19	zinc finger. DHHC-type containing 19
1.07	MUSK	muscle. skeletal. receptor tyrosine kinase
1.07	POMP	proteasome maturation protein
1.07	PWWP2A	PWWP domain containing 2A
1.07	NIPAL1	NIPA-like domain containing 1
1.07	ARPP19	cAMP-regulated phosphoprotein 19kDa
1.07	C5orf24	chromosome 5 open reading frame 24
1.07	SPDYE6; SPDYE2	speedy/RINGO cell cycle regulator family member E6; speedy/RINGO cell cycle regulator family member E2
1.07	OCIAD1	Transcript Identified by AceView. Entrez Gene ID(s) 54940
1.07	SCAPER	S-phase cyclin A-associated protein in the ER
1.07	RNF145	ring finger protein 145
1.07	CCZ1B	CCZ1 homolog B. vacuolar protein trafficking and biogenesis associated

1.07	ANO2	anoctamin 2. calcium activated chloride channel
1.07	NOP14	NOP14 nucleolar protein
1.07	NWD2	NACHT and WD repeat domain containing 2
1.07	CSNK2A1	casein kinase 2. alpha 1 polypeptide
1.07	ZNF879	zinc finger protein 879
1.07	GGCT	gamma-glutamylcyclotransferase
1.07	DNAJC22	DnaJ (Hsp40) homolog. subfamily C. member 22
1.07	MPST	mercaptopyruvate sulfurtransferase
1.07	PPP1R21	protein phosphatase 1. regulatory subunit 21
1.07	PLEKHG4B	Zhang2013 ALT_ACCEPTOR. ALT_DONOR. coding. INTERNAL. intronic best transcript NM_052909
1.07	CDC42EP3	CDC42 effector protein (Rho GTPase binding) 3
1.07	PAIP2	poly(A) binding protein interacting protein 2
1.07	TMPRSS12	transmembrane (C-terminal) protease. serine 12
1.07	C8orf22	chromosome 8 open reading frame 22
1.07	MAP2K4; MIR744	mitogen-activated protein kinase kinase 4; microRNA 744
1.07	RORB	RAR-related orphan receptor B
1.07	GTF2H3	general transcription factor IIH subunit 3
1.07	AMD1	adenosylmethionine decarboxylase 1
1.07	SDF2	stromal cell-derived factor 2
1.07	SLC22A24	solute carrier family 22. member 24
1.07	OR10G9	olfactory receptor. family 10. subfamily G. member 9
1.07	KRTAP4-1	keratin associated protein 4-1
1.07	BCL2L14	BCL2-like 14 (apoptosis facilitator)
1.07	LHX4	LIM homeobox 4
1.07	PCP4L1	Purkinje cell protein 4 like 1

1.07	ADAM8	Memczak2013 ANTISENSE. CDS. coding. INTERNAL best transcript NM_001109
1.07	HS6ST1	heparan sulfate 6-O-sulfotransferase 1
1.07	MLC1	megalencephalic leukoencephalopathy with subcortical cysts 1
1.07	PLIN4	perilipin 4
1.07	KRBOX1	KRAB box domain containing 1
1.07	MTAP	methylthioadenosine phosphorylase
1.07	FAR2	fatty acyl-CoA reductase 2
1.07	SPPL2A	signal peptide peptidase like 2A
1.07	USH2A	Usher syndrome 2A (autosomal recessive. mild)
1.07	GBP2	guanylate binding protein 2. interferon-inducible
1.07	SCN3B	sodium channel. voltage gated. type III beta subunit
1.07	CEP131	centrosomal protein 131kDa
1.07	INTS10	integrator complex subunit 10
1.07	CPNE7	copine VII
1.07	INTS4P2	integrator complex subunit 4 pseudogene 2
1.07	SNTB1	syntrophin. beta 1 (dystrophin-associated protein A1. 59kDa. basic component 1)
1.07	DGKD	diacylglycerol kinase. delta 130kDa
1.07	ERMAP	erythroblast membrane-associated protein (Scianna blood group)
1.07	CHRNA3	cholinergic receptor. nicotinic alpha 3
1.07	RASGEF1A	RasGEF domain family member 1A
1.07	MSRA	methionine sulfoxide reductase A
1.07	MPPE1	metallophosphoesterase 1
1.07	POPDC3	popeye domain containing 3
1.07	PTP4A3	protein tyrosine phosphatase type IVA. member 3
1.07	ELF4	Memczak2013 ALT_DONOR. coding. INTERNAL. intronic best transcript

		NM_001421
1.07	LHX1	LIM homeobox 1
1.07	NCAN	neurocan
1.07	OR6V1	olfactory receptor. family 6. subfamily V. member 1
1.07	C12orf77	chromosome 12 open reading frame 77
1.07	SMPX	small muscle protein. X-linked
1.07	N4BP1	NEDD4 binding protein 1
1.07	BTBD1	BTB (POZ) domain containing 1
1.07	ZNF155	zinc finger protein 155
1.07	ANKRD22	ankyrin repeat domain 22
1.07	CPT1B	carnitine palmitoyltransferase 1B (muscle)
1.07	AMN1	antagonist of mitotic exit network 1 homolog
1.07	HMGB4	high mobility group box 4
1.07	NDUFA10	NADH dehydrogenase (ubiquinone) 1 alpha subcomplex. 10. 42kDa
1.07	SDHB	succinate dehydrogenase complex subunit B. iron sulfur (Ip)
1.07	BCL2L13	BCL2-like 13 (apoptosis facilitator)
1.07	ADAMTS19	ADAM metallopeptidase with thrombospondin type 1 motif 19
1.07	SDHA	succinate dehydrogenase complex subunit A. flavoprotein (Fp)
1.07	FAM57B	family with sequence similarity 57. member B
1.07	MICAL2	microtubule associated monooxygenase. calponin and LIM domain containing 2
1.07	PPP1R7	protein phosphatase 1. regulatory subunit 7
1.07	PSPN	persephin
1.07	SLC25A43	solute carrier family 25. member 43
1.07	RPL19	ribosomal protein L19
1.07	IPO4	importin 4

1.07	ZNF582	zinc finger protein 582
1.07	GABRG3	gamma-aminobutyric acid (GABA) A receptor. gamma 3
1.07	PLAA	phospholipase A2-activating protein
1.07	SMARCD1	SWI/SNF related. matrix associated. actin dependent regulator of chromatin. subfamily d. member 1
1.07	UBE3B	Transcript Identified by AceView. Entrez Gene ID(s) 89910
1.07	TRIM25; MIR3614	tripartite motif containing 25; microRNA 3614
1.07	PDHX	pyruvate dehydrogenase complex. component X
1.07	MAP6	microtubule associated protein 6
1.07	HSF5	heat shock transcription factor family member 5
1.07	MIR4738; H3F3B	microRNA 4738; H3 histone. family 3B (H3.3B)
1.07	CT47A7; CT47A5; CT47A6	cancer/testis antigen family 47. member A7; cancer/testis antigen family 47. member A5; cancer/testis antigen family 47. member A6
1.07	IGF1R	insulin-like growth factor 1 receptor
1.07	KIAA0355	KIAA0355
1.07	ZBED9	zinc finger. BED-type containing 9
1.07	UIMC1	ubiquitin interaction motif containing 1
1.07	KIAA1804; MLK4	mixed lineage kinase 4; Mitogen-activated protein kinase kinase kinase MLK4 [Source:UniProtKB/Swiss-Prot;Acc:Q5TCX8]; Salzman2013 ANNOTATED. CDS. coding. INTERNAL. OVCODE. OVEXON best transcript NM_032435
1.07	KHDRBS3	KH domain containing. RNA binding. signal transduction associated 3
1.07	ARHGEF38	Rho guanine nucleotide exchange factor 38
1.07	DYNLL2	Memczak2013 ANTISENSE. coding. INTERNAL. UTR3 best transcript NM_080677
1.07	OR56A5	olfactory receptor. family 56. subfamily A. member 5
1.07	PREX1	Memczak2013 ALT_ACCEPTOR. ALT_DONOR. coding. INTERNAL. intronic best transcript NM_020820
1.07	MFSD6	major facilitator superfamily domain containing 6

1.07	KIFC3	Jeck2013 ANTISENSE. coding. INTERNAL. intronic best transcript NM_001130100
1.07	FRG1BP	FSHD region gene 1 family member B. pseudogene
1.07	MAP4K2	mitogen-activated protein kinase kinase kinase kinase 2
1.07	CCDC13	coiled-coil domain containing 13
1.07	TCL1B; TCL6	T-cell leukemia/lymphoma 1B; T-cell leukemia/lymphoma 6 (non-protein coding)
1.07	BRS3	bombesin-like receptor 3
1.07	PLD5	phospholipase D family. member 5
1.07	REG4	regenerating islet-derived family. member 4
1.07	SNRK	SNF related kinase
1.07	ARL2-SNX15	ARL2-SNX15 readthrough (NMD candidate)
1.07	PRSS46	protease. serine 46
1.07	VAMP7	vesicle associated membrane protein 7
1.07	IZUMO2	IZUMO family member 2
1.07	SHROOM3	shroom family member 3
1.07	TTC29	tetratricopeptide repeat domain 29
1.07	PRAMEF19	PRAME family member 19
1.07	TLE3	transducin-like enhancer of split 3
1.07	RP11-281A20.2; TLR4	novel transcript; Transcript Identified by AceView. Entrez Gene ID(s) 7099
1.07	GREM1	gremlin 1. DAN family BMP antagonist [Source:HGNC Symbol;Acc:HGNC:2001]
1.07	PSMD11	proteasome 26S subunit. non-ATPase 11
1.07	MLLT6	myeloid/lymphoid or mixed-lineage leukemia; translocated to. 6
1.07	ZNF563	zinc finger protein 563
1.07	GNB2L1; SNORD95; SNORD96A	guanine nucleotide binding protein (G protein). beta polypeptide 2-like 1; small nucleolar RNA. C/D box 95; small nucleolar RNA. C/D box 96A

1.07	KIAA2026	KIAA2026
1.07	EPAS1	endothelial PAS domain protein 1
1.07	MBTPS1	membrane bound transcription factor peptidase. site 1
1.07	PUM2	pumilio RNA binding family member 2
1.07	OR51E2	olfactory receptor. family 51. subfamily E. member 2
1.07	ZDBF2	zinc finger. DBF-type containing 2
1.07	MYH9	Zhang2013 ALT_ACCEPTOR. ALT_DONOR. coding. INTERNAL. intronic best transcript NM_002473
1.07	GJD4	gap junction protein delta 4
1.07	ELL2	elongation factor. RNA polymerase II. 2
1.07	C2orf66	chromosome 2 open reading frame 66
1.07	PHKG1	phosphorylase kinase. gamma 1 (muscle)
1.07	BEND4	BEN domain containing 4
1.07	CRIPT	cysteine-rich PDZ-binding protein
1.07	ZNF708	zinc finger protein 708
1.07	OR11L1	olfactory receptor. family 11. subfamily L. member 1
1.07	USP17L21	ubiquitin specific peptidase 17-like family member 21
1.07	ZFP91	ZFP91 zinc finger protein
1.07	ZNF644	zinc finger protein 644
1.07	OR52A5	olfactory receptor. family 52. subfamily A. member 5
1.07	NDUFA10	NADH dehydrogenase (ubiquinone) 1 alpha subcomplex. 10. 42kDa
1.07	GYPB	glycophorin B (MNS blood group)
1.07	KATNAL1	katanin p60 subunit A-like 1
1.07	ZNF521	zinc finger protein 521
1.07	RNASEH2B	ribonuclease H2. subunit B
1.07	TM2D1	Transcript Identified by AceView. Entrez Gene ID(s) 83941

1.07	ARPC5	actin related protein 2/3 complex subunit 5
1.07	ERVH48-1	endogenous retrovirus group 48. member 1
1.07	GGNBP2	gametogenetin binding protein 2
1.07	FOLH1	folate hydrolase (prostate-specific membrane antigen) 1
1.07	FNDC9	fibronectin type III domain containing 9
1.07	SEZ6	seizure related 6 homolog (mouse)
1.07	RFPL4B	ret finger protein-like 4B
1.07	VCPKMT	valosin containing protein lysine (K) methyltransferase
1.07	RHEBL1	Ras homolog enriched in brain like 1
1.07	GTF2IRD2; GTF2IRD2B	GTF2I repeat domain containing 2; GTF2I repeat domain containing 2B
1.07	IFNL3	interferon. lambda 3
1.07	OR10K1	olfactory receptor. family 10. subfamily K. member 1
1.07	MKRN1	makorin ring finger protein 1
1.07	CLINT1	clathrin interactor 1
1.07	MLXIP	MLX interacting protein
1.07	GUCA2B	guanylate cyclase activator 2B (uroguanylin)
1.07	CPLX2	complexin 2
1.07	PDE3A	phosphodiesterase 3A. cGMP-inhibited
1.07	TMEM119	transmembrane protein 119
1.07	SOGA3; KIAA0408	SOGA family member 3; KIAA0408
1.07	KIAA0556	KIAA0556
1.07	SGK3	serum/glucocorticoid regulated kinase family. member 3
1.07	MEOX2	mesenchyme homeobox 2
1.07	ZNF541	zinc finger protein 541
1.07	BBS5	Bardet-Biedl syndrome 5
1.07	FAM229B	family with sequence similarity 229. member B

1.07	KCNQ4	potassium channel. voltage gated KQT-like subfamily Q. member 4
1.07	SMU1	smu-1 suppressor of mec-8 and unc-52 homolog (C. elegans)
1.07	VSIG10L	V-set and immunoglobulin domain containing 10 like
1.07	GPR31	G protein-coupled receptor 31
1.07	EVA1B	eva-1 homolog B (C. elegans)
1.07	OR52E2	olfactory receptor. family 52. subfamily E. member 2
1.07	SHMT2	serine hydroxymethyltransferase 2 (mitochondrial)
1.07	UPK1B	uroplakin 1B
1.07	TRPC3	transient receptor potential cation channel. subfamily C. member 3
1.07	SCUBE3	signal peptide. CUB domain. EGF-like 3
1.07	BMP10	bone morphogenetic protein 10
1.07	OR8G2	olfactory receptor. family 8. subfamily G. member 2
1.07	C18orf65	chromosome 18 open reading frame 65
1.07	GINS3	GINS complex subunit 3 (Psf3 homolog)
1.07	CSF3R	colony stimulating factor 3 receptor
1.07	ARFGEF2	ADP-ribosylation factor guanine nucleotide-exchange factor 2 (brefeldin A-inhibited)
1.07	NCAM2	neural cell adhesion molecule 2
1.07	CCDC152	coiled-coil domain containing 152
1.07	FMN1	formin 1
1.07	DMAP1	DNA methyltransferase 1 associated protein 1
1.07	MUC16	mucin 16. cell surface associated
1.07	DDX17	DEAD (Asp-Glu-Ala-Asp) box helicase 17
1.07	GPR55	G protein-coupled receptor 55
1.07	ZNF260	zinc finger protein 260
1.07	EMX2	empty spiracles homeobox 2

1.07	ZNF593	zinc finger protein 593
1.07	RFFL	ring finger and FYVE-like domain containing E3 ubiquitin protein ligase
1.07	COPS7A	COP9 signalosome subunit 7A
1.07	NONO	non-POU domain containing. octamer-binding
1.07	PPM1F	protein phosphatase. Mg2+/Mn2+ dependent. 1F
1.07	FAM47C	family with sequence similarity 47. member C
1.07	MYO19	myosin XIX
1.07	ZDHHC3	zinc finger. DHHC-type containing 3
1.07	RHOG	ras homolog family member G
1.07	POGZ	pogo transposable element with ZNF domain
1.07	SPOCK2	sparc/osteonectin. cwcv and kazal-like domains proteoglycan (testican) 2
1.07	NADK2	NAD kinase 2. mitochondrial
1.07	FMN1	formin 1
1.07	TRIM34	tripartite motif containing 34
1.07	TCF20	transcription factor 20 (AR1)
1.07	NFKBIE	nuclear factor of kappa light polypeptide gene enhancer in B-cells inhibitor. epsilon
1.07	CHCHD1	coiled-coil-helix-coiled-coil-helix domain containing 1
1.07	KIF21B	kinesin family member 21B
1.07	PRKCQ	protein kinase C. theta
1.07	TAC1	tachykinin. precursor 1
1.07	RABEPK	Rab9 effector protein with kelch motifs
1.07	SLC31A1	solute carrier family 31 (copper transporter). member 1
1.07	ANO9	anoctamin 9
1.07	PRR32	proline rich 32
1.07	TNIP2	TNFAIP3 interacting protein 2

1.07	PAICS	phosphoribosylaminoimidazole carboxylase. phosphoribosylaminoimidazole succinocarboxamide synthetase
1.07	PLLP	plasmolipin
1.07	CXCR1	chemokine (C-X-C motif) receptor 1
1.07	BCAS4	breast carcinoma amplified sequence 4
1.07	CUTC	cutC copper transporter
1.07	CDH22	cadherin 22. type 2
1.07	ANKRD35	ankyrin repeat domain 35
1.07	PITRM1	pitrilysin metallopeptidase 1
1.07	NUTF2	nuclear transport factor 2
1.07	NUP35	nucleoporin 35kDa
1.07	RPS6KA6	ribosomal protein S6 kinase. 90kDa. polypeptide 6
1.07	CCNC	cyclin C
1.07	EPHX4	epoxide hydrolase 4
1.07	TPD52	tumor protein D52
1.07	SPOP	speckle-type POZ protein
1.07	MAD2L2	MAD2 mitotic arrest deficient-like 2 (yeast)
1.07	KIF3B	kinesin family member 3B
1.07	GABPB2	GA binding protein transcription factor. beta subunit 2
1.07	SLC36A3	solute carrier family 36. member 3
1.07	CEBPZOS	CEBPZ opposite strand
1.07	ACTL6B	actin-like 6B
1.07	C21orf62	chromosome 21 open reading frame 62
1.07	MZT1	mitotic spindle organizing protein 1
1.07	GNPAT	glyceronephosphate O-acyltransferase
1.07	USP9X	ubiquitin specific peptidase 9. X-linked

1.07	MMP21	matrix metallopeptidase 21
1.07	PPP4R1	protein phosphatase 4. regulatory subunit 1
1.07	GGACT	gamma-glutamylamine cyclotransferase
1.07	SLC38A8	solute carrier family 38. member 8
1.07	SEMG2	semenogelin II
1.07	BCL7A	Memczak2013 ANTISENSE. coding. INTERNAL. intronic best transcript NM_001024808
1.07	WDR45B	WD repeat domain 45B
1.07	TRPA1	transient receptor potential cation channel. subfamily A. member 1
1.07	PARP8	Memczak2013 ANTISENSE. CDS. coding. INTERNAL. intronic best transcript NM_001178056
1.07	HIST3H3	histone cluster 3. H3
1.07	YDJC	YdjC homolog (bacterial)
1.07	UBAP1	ubiquitin associated protein 1
1.07	DPP10	dipeptidyl-peptidase 10 (inactive)
1.07	C5orf66	chromosome 5 open reading frame 66
1.07	NFATC4	nuclear factor of activated T-cells. cytoplasmic. calcineurin-dependent 4
1.07	ASXL2	additional sex combs like transcriptional regulator 2
1.07	LINC01235	long intergenic non-protein coding RNA 1235
1.07	KRT82	keratin 82. type II
1.07	TEX11	testis expressed 11
1.07	IL12A	interleukin 12A
1.07	LSMEM1	leucine-rich single-pass membrane protein 1
1.07	MAP1B	microtubule associated protein 1B
1.07	EIF2S3	eukaryotic translation initiation factor 2. subunit 3 gamma. 52kDa
1.07	EPC2	enhancer of polycomb homolog 2 (Drosophila)
1.07	SERINC3	serine incorporator 3

1.07	GHR	growth hormone receptor
1.07	GALNTL6	Salzman2013 ANTISENSE. coding. INTERNAL. intronic best transcript NM_001034845
1.07	OR56B4	olfactory receptor. family 56. subfamily B. member 4
1.07	DGCR8; MIR1306; MIR3618	DGCR8 microprocessor complex subunit; microRNA 1306; microRNA 3618
1.07	HOXA11	homeobox A11
1.07	DUX4L6	double homeobox 4 like 6
1.07	DUX4L5	double homeobox 4 like 5
1.07	DUX4L1	double homeobox 4 like 1
1.07	DUX4L3	double homeobox 4 like 3
1.07	DUX4L2	double homeobox 4 like 2
1.07	DEFB105B; DEFB105A	defensin. beta 105B; defensin. beta 105A
1.07	COX8C	cytochrome c oxidase subunit VIIIC
1.07	TIGD7	tigger transposable element derived 7
1.07	YEATS4	Transcript Identified by AceView. Entrez Gene ID(s) 8089
1.07	FBXO21	F-box protein 21
1.07	CRACR2B	calcium release activated channel regulator 2B
1.07	PLPPR3	phospholipid phosphatase related 3
1.07	SPARCL1	SPARC like 1
1.07	MBLAC1	metallo-beta-lactamase domain containing 1
1.07	KBTBD11	kelch repeat and BTB (POZ) domain containing 11
1.07	OR4A47	olfactory receptor. family 4. subfamily A. member 47
1.07	TCEANC	transcription elongation factor A (SII) N-terminal and central domain containing
1.07	PAGE2	P antigen family. member 2 (prostate associated)
1.06	ARAF	A-Raf proto-oncogene. serine/threonine kinase
1.06	WRAP73	WD repeat containing. antisense to TP73

1.06	PPP2R2C	protein phosphatase 2. regulatory subunit B. gamma
1.06	KRTAP22-2	keratin associated protein 22-2
1.06	C9orf84	chromosome 9 open reading frame 84
1.06	SNAP47	synaptosome associated protein 47kDa
1.06	IFIT1B	interferon-induced protein with tetratricopeptide repeats 1B
1.06	SUFU	Transcript Identified by AceView. Entrez Gene ID(s) 51684
1.06	LINC01590; SMIM8	long intergenic non-protein coding RNA 1590; small integral membrane protein 8
1.06	FBXO25	F-box protein 25
1.06	IRX2	Transcript Identified by AceView. Entrez Gene ID(s) 153572
1.06	UNC50	unc-50 homolog (C. elegans)
1.06	MPHOSPH8	M-phase phosphoprotein 8
1.06	ADCY2	Transcript Identified by AceView. Entrez Gene ID(s) 108
1.06	FCRL2	Fc receptor-like 2
1.06	LIPA	Transcript Identified by AceView. Entrez Gene ID(s) 3988
1.06	ZNF596	zinc finger protein 596
1.06	CTAGE8; CTAGE4	CTAGE family. member 8; CTAGE family. member 4
1.06	RNF224	ring finger protein 224
1.06	KIAA2026	KIAA2026
1.06	ASIC2	acid sensing ion channel 2
1.06	LITAF	lipopolysaccharide-induced TNF factor
1.06	C6orf15	chromosome 6 open reading frame 15
1.06	PHACTR3	Transcript Identified by AceView. Entrez Gene ID(s) 116154
1.06	ZNF629	zinc finger protein 629
1.06	CCDC58	coiled-coil domain containing 58
1.06	CLVS2	clavesin 2

1.06	ITGA7	integrin alpha 7
1.06	MAGEA12; CSAG4	MAGE family member A12; CSAG family. member 4 (pseudogene)
1.06	SLC25A26	solute carrier family 25 (S-adenosylmethionine carrier). member 26
1.06	USP17L23	ubiquitin specific peptidase 17-like family member 23 [Source:HGNC Symbol;Acc:HGNC:44451]
1.06	ZNF534	zinc finger protein 534
1.06	KLHL33	kelch-like family member 33
1.06	FAM122A	family with sequence similarity 122A
1.06	GGA2	golgi-associated. gamma adaptin ear containing. ARF binding protein 2
1.06	RNASE7	ribonuclease. RNase A family. 7
1.06	RBMS2	RNA binding motif. single stranded interacting protein 2
1.06	FUT6	fucosyltransferase 6 (alpha (1.3) fucosyltransferase)
1.06	CFAP43	cilia and flagella associated protein 43
1.06	OR2AP1	olfactory receptor. family 2. subfamily AP. member 1
1.06	DTD1	D-tyrosyl-tRNA deacylase 1
1.06	PAPOLB	poly(A) polymerase beta
1.06	PPM1E	Transcript Identified by AceView. Entrez Gene ID(s) 22843
1.06	PON3	paraoxonase 3
1.06	MN1	meningioma (disrupted in balanced translocation) 1
1.06	ABI3BP	ABI family. member 3 (NESH) binding protein
1.06	CFP	complement factor properdin
1.06	NAP1L2	Transcript Identified by AceView. Entrez Gene ID(s) 4674
1.06	BMS1P20; IGLV1-51; IGLV5-52	BMS1 ribosome biogenesis factor pseudogene 20; immunoglobulin lambda variable 1-51; immunoglobulin lambda variable 5-52
1.06	GPR139	G protein-coupled receptor 139
1.06	HHAT	hedgehog acyltransferase
1.06	TCEA2	transcription elongation factor A (SII). 2

1.06	DGKD	diacylglycerol kinase. delta 130kDa
1.06	OPN1LW	opsin 1 (cone pigments). long-wave-sensitive
1.06	SLC38A6	solute carrier family 38. member 6
1.06	PNLIPRP1	pancreatic lipase-related protein 1
1.06	SUDS3	SDS3 homolog. SIN3A corepressor complex component
1.06	NRG3	neuregulin 3
1.06	PIGR	polymeric immunoglobulin receptor
1.06	DOCK8	dedicator of cytokinesis 8
1.06	OAF	out at first homolog
1.06	ZNF780B	zinc finger protein 780B
1.06	HM13; MCTS2P	histocompatibility (minor) 13; malignant T-cell amplified sequence 2. pseudogene
1.06	ZNF292	zinc finger protein 292
1.06	ADCK2	aarF domain containing kinase 2
1.06	ADK	Memczak2013 ANTISENSE. coding. INTERNAL. intronic best transcript NM_006721
1.06	PIANP	PILR alpha associated neural protein
1.06	RAB38	RAB38. member RAS oncogene family
1.06	OR8H2	olfactory receptor. family 8. subfamily H. member 2
1.06	IL19	interleukin 19
1.06	MADD	MAP-kinase activating death domain
1.06	CCZ1; CCZ1B	CCZ1 homolog. vacuolar protein trafficking and biogenesis associated; CCZ1 homolog B. vacuolar protein trafficking and biogenesis associated
1.06	GGA3	golgi-associated. gamma adaptin ear containing. ARF binding protein 3
1.06	GPRASP1	G protein-coupled receptor associated sorting protein 1
1.06	SMR3A	submaxillary gland androgen regulated protein 3A
1.06	GOLGA8EP; GOLGA8DP	golgin A8 family. member E. pseudogene; golgin A8 family. member D.

		pseudogene
1.06	INO80E	INO80 complex subunit E
1.06	JMJD1C-AS1	JMJD1C antisense RNA 1
1.06	NR4A3	nuclear receptor subfamily 4. group A. member 3
1.06	BEND3	BEN domain containing 3
1.06	FASTK	Fas-activated serine/threonine kinase
1.06	FMNL1	formin like 1
1.06	LPCAT4	lysophosphatidylcholine acyltransferase 4
1.06	GGT2	gamma-glutamyltransferase 2
1.06	HLA-DQB1	major histocompatibility complex. class II. DQ beta 1
1.06	ARHGEF10	Jeck2013 ALT_ACCEPTOR. ALT_DONOR. coding. INTERNAL. intronic best transcript NM_014629
1.06	TRAPPC4; MIR3656	trafficking protein particle complex 4; microRNA 3656
1.06	PRKRIP1	PRKR interacting protein 1 (IL11 inducible)
1.06	TNFRSF4	tumor necrosis factor receptor superfamily. member 4
1.06	NKX2-1	NK2 homeobox 1
1.06	FAM149A	family with sequence similarity 149. member A
1.06	SLC25A41	solute carrier family 25. member 41
1.06	OR1C1	olfactory receptor. family 1. subfamily C. member 1
1.06	TAS2R1	taste receptor. type 2. member 1
1.06	HOXC11	homeobox C11
1.06	DCTN1	dynactin 1
1.06	OR51B5	olfactory receptor. family 51. subfamily B. member 5
1.06	GPR160	G protein-coupled receptor 160
1.06	LGALS13	lectin. galactoside-binding. soluble. 13
1.06	CSAG3	CSAG family. member 3

1.06	ZNF669	zinc finger protein 669
1.06	MDGA2	MAM domain containing glycosylphosphatidylinositol anchor 2
1.06	PFDN5	prefoldin subunit 5
1.06	MAGEB17	MAGE family member B17
1.06	LINC01549	long intergenic non-protein coding RNA 1549
1.06	TFF2	trefoil factor 2
1.06	SIGLEC12	sialic acid binding Ig-like lectin 12 (gene/pseudogene)
1.06	LRRTM4	leucine rich repeat transmembrane neuronal 4
1.06	RASSF3	Ras association (RalGDS/AF-6) domain family member 3
1.06	CAMK2A	calcium/calmodulin-dependent protein kinase II alpha
1.06	HDAC7	histone deacetylase 7
1.06	PTPRA; VPS16	protein tyrosine phosphatase. receptor type. A; vacuolar protein sorting 16 homolog (S. cerevisiae)
1.06	SIAH3	siah E3 ubiquitin protein ligase family member 3
1.06	TAOK1; MIR4523	TAO kinase 1; microRNA 4523
1.06	ZBTB45	zinc finger and BTB domain containing 45
1.06	IL16	interleukin 16
1.06	LNX1	ligand of numb-protein X 1. E3 ubiquitin protein ligase
1.06	C1orf228	chromosome 1 open reading frame 228
1.06	TXNRD2	Transcript Identified by AceView. Entrez Gene ID(s) 10587
1.06	UCN	urocortin
1.06	INTU	inturned planar cell polarity protein
1.06	CSRNP3	cysteine-serine-rich nuclear protein 3
1.06	RPL7A	ribosomal protein L7a
1.06	TMBIM1; MIR6513	transmembrane BAX inhibitor motif containing 1; microRNA 6513
1.06	SMARCA4	SWI/SNF related. matrix associated. actin dependent regulator of chromatin. subfamily a. member 4

1.06	PPP1R14D	protein phosphatase 1. regulatory (inhibitor) subunit 14D
1.06	FAM46D	family with sequence similarity 46. member D
1.06	PLCL1	phospholipase C-like 1
1.06	RAF1	Raf-1 proto-oncogene. serine/threonine kinase
1.06	RAB5A	RAB5A. member RAS oncogene family
1.06	EIF2D	eukaryotic translation initiation factor 2D
1.06	PHIP	pleckstrin homology domain interacting protein
1.06	CBLN2	cerebellin 2 precursor
1.06	PIK3C3	phosphatidylinositol 3-kinase. catalytic subunit type 3
1.06	SDK1	sidekick cell adhesion molecule 1
1.06	VAMP8	vesicle associated membrane protein 8
1.06	C1orf159	chromosome 1 open reading frame 159
1.06	DNAJB3	DnaJ (Hsp40) homolog. subfamily B. member 3
1.06	RANBP3	RAN binding protein 3
1.06	ESX1	ESX homeobox 1
1.06	CTSH	cathepsin H
1.06	EIF4EBP1	eukaryotic translation initiation factor 4E binding protein 1
1.06	ACTC1	actin. alpha. cardiac muscle 1
1.06	RNASE3	ribonuclease. RNase A family. 3
1.06	ANGPTL1	angiopoietin like 1
1.06	ASZ1	ankyrin repeat. SAM and basic leucine zipper domain containing 1
1.06	BPI	bactericidal/permeability-increasing protein
1.06	KAT6B	K(lysine) acetyltransferase 6B
1.06	HLF	hepatic leukemia factor
1.06	FH	fumarate hydratase
1.06	IL17A	interleukin 17A

1.06	TANK	TRAF family member-associated NFKB activator
1.06	LTBP4	Transcript Identified by AceView. Entrez Gene ID(s) 8425
1.06	TMEM214	transmembrane protein 214
1.06	OR8H1	olfactory receptor. family 8. subfamily H. member 1
1.06	EDN1	endothelin 1
1.06	THY1	Thy-1 cell surface antigen
1.06	OR5D14	olfactory receptor. family 5. subfamily D. member 14
1.06	VIP	vasoactive intestinal peptide
1.06	BRAT1	BRCA1-associated ATM activator 1
1.06	BCLAF1	BCL2-associated transcription factor 1
1.06	MFSD12	major facilitator superfamily domain containing 12
1.06	IARS	isoleucyl-tRNA synthetase
1.06	KIF5B	Memczak2013 ANTISENSE. CDS. coding. INTERNAL. UTR3 best transcript NM_004521
1.06	C1orf27	chromosome 1 open reading frame 27
1.06	MRFAP1	Morf4 family associated protein 1
1.06	OR5AS1	olfactory receptor. family 5. subfamily AS. member 1
1.06	PRPF8	pre-mRNA processing factor 8
1.06	C17orf78	chromosome 17 open reading frame 78
1.06	TXNDC15	thioredoxin domain containing 15
1.06	GALNT13	polypeptide N-acetylgalactosaminyltransferase 13
1.06	SH3BP1; PDXP	SH3-domain binding protein 1; pyridoxal (pyridoxine. vitamin B6) phosphatase
1.06	AKR1C8P	aldo-keto reductase family 1. member C8. pseudogene
1.06	LOC729159; RP11-457D20.2	UPF0607 protein ENSP00000381418-like; Putative UPF0607 protein ENSP00000381418 [Source:UniProtKB/Swiss-Prot;Acc:A8MU76]
1.06	CCL23	chemokine (C-C motif) ligand 23

1.06	CMC2	C-x(9)-C motif containing 2
1.06	ADIPOQ	adiponectin. C1Q and collagen domain containing
1.06	TAGLN2	transgelin 2
1.06	TFAP4	transcription factor AP-4 (activating enhancer binding protein 4)
1.06	RNF7	ring finger protein 7
1.06	COL21A1	collagen. type XXI. alpha 1
1.06	CHPT1	choline phosphotransferase 1
1.06	DPH3P1	diphthamide biosynthesis 3 pseudogene 1
1.06	ANXA13	annexin A13
1.06	WRB	tryptophan rich basic protein
1.06	GFRA1	GDNF family receptor alpha 1
1.06	CYLC1	cylicin. basic protein of sperm head cytoskeleton 1
1.06	IRF3	interferon regulatory factor 3
1.06	CCNB1IP1; SNORD126	cyclin B1 interacting protein 1. E3 ubiquitin protein ligase; small nucleolar RNA. C/D box 126
1.06	PSMC4	proteasome 26S subunit. ATPase 4 [Source:HGNC Symbol;Acc:HGNC:9551]
1.06	TBCEL	tubulin folding cofactor E-like
1.06	GZMK	granzyme K
1.06	CHST11	carbohydrate (chondroitin 4) sulfotransferase 11
1.06	RPS6KA1	ribosomal protein S6 kinase. 90kDa. polypeptide 1
1.06	SARAF	store-operated calcium entry-associated regulatory factor
1.06	FNDC5	fibronectin type III domain containing 5
1.06	TMEM14B	transmembrane protein 14B
1.06	CHD9	chromodomain helicase DNA binding protein 9
1.06	PTPN2	protein tyrosine phosphatase. non-receptor type 2
1.06	KCNJ6	potassium channel. inwardly rectifying subfamily J. member 6

1.06	RPS28	ribosomal protein S28
1.06	KCNK17	potassium channel. two pore domain subfamily K. member 17
1.06	UNC13D	unc-13 homolog D (C. elegans)
1.06	PCOLCE2	procollagen C-endopeptidase enhancer 2
1.06	PRSS3	protease. serine. 3
1.06	POU2AF1	POU class 2 associating factor 1
1.06	KDM1A; MIR3115	lysine (K)-specific demethylase 1A; microRNA 3115
1.06	BMP15	bone morphogenetic protein 15
1.06	KIAA0391	KIAA0391
1.06	GPR87	G protein-coupled receptor 87
1.06	ACVR1B	activin A receptor type IB
1.06	PLS3	plastin 3
1.06	ZFAND6	zinc finger. AN1-type domain 6
1.06	SAMD7	sterile alpha motif domain containing 7
1.06	IL13RA2	interleukin 13 receptor. alpha 2
1.06	RBM19	RNA binding motif protein 19
1.06	CARD18	caspase recruitment domain family. member 18
1.06	C11orf95	chromosome 11 open reading frame 95
1.06	GTF3C4	general transcription factor IIIC subunit 4
1.06	KLHL17	kelch-like family member 17
1.06	TUBA3C	tubulin. alpha 3c
1.06	NID2	nidogen 2 (osteonidogen)
1.06	WSB1	WD repeat and SOCS box containing 1
1.06	OR6C75	olfactory receptor. family 6. subfamily C. member 75
1.06	INPP5D	inositol polyphosphate-5-phosphatase D
1.06	NDUFA3	NADH dehydrogenase (ubiquinone) 1 alpha subcomplex. 3. 9kDa

1.06	MRRF	mitochondrial ribosome recycling factor
1.06	ZNF569	zinc finger protein 569
1.06	IFNL2	interferon. lambda 2
1.06	SH2D4A	SH2 domain containing 4A
1.06	LRRC8C	leucine rich repeat containing 8 family. member C
1.06	NDUFA6	NADH dehydrogenase (ubiquinone) 1 alpha subcomplex. 6. 14kDa
1.06	FUT9	fucosyltransferase 9 (alpha (1.3) fucosyltransferase)
1.06	PDE6C	phosphodiesterase 6C. cGMP-specific. cone. alpha prime
1.06	REG1A	regenerating islet-derived 1 alpha
1.06	CALN1	calneuron 1
1.06	COL6A2	Jeck2013 ALT_ACCEPTOR. ALT_DONOR. coding. INTERNAL. intronic best transcript NM_058175
1.06	RRP12	ribosomal RNA processing 12 homolog
1.06	RIF1	replication timing regulatory factor 1
1.06	ZDHHC9	zinc finger. DHHC-type containing 9
1.06	FRMPD2	FERM and PDZ domain containing 2
1.06	LILRA4	leukocyte immunoglobulin-like receptor. subfamily A (with TM domain). member 4
1.06	ZNF197	zinc finger protein 197
1.06	TMEM9B	TMEM9 domain family. member B
1.06	RNF165	ring finger protein 165
1.06	MST1R	macrophage stimulating 1 receptor
1.06	CCDC90B	coiled-coil domain containing 90B
1.06	MFSD14C	major facilitator superfamily domain containing 14C
1.06	SHOX	short stature homeobox
1.06	ANG; RNASE4	angiogenin. ribonuclease. RNase A family. 5; ribonuclease. RNase A family. 4
1.06	NCBP2	nuclear cap binding protein subunit 2

1.06	CLEC12B	C-type lectin domain family 12. member B
1.06	EIF5	eukaryotic translation initiation factor 5
1.06	REC114	REC114 meiotic recombination protein
1.06	RNF185	ring finger protein 185
1.06	RPS29	ribosomal protein S29
1.06	C11orf86	chromosome 11 open reading frame 86
1.06	DCTN5	dynactin 5 (p25)
1.06	ACADM	acyl-CoA dehydrogenase. C-4 to C-12 straight chain
1.06	MAP4	Transcript Identified by AceView. Entrez Gene ID(s) 4134
1.06	HIST1H4G	histone cluster 1. H4g
1.06	CCDC109B	coiled-coil domain containing 109B
1.06	NCOA1	nuclear receptor coactivator 1
1.06	SSB	Sjogren syndrome antigen B (autoantigen La)
1.06	ZNF774	zinc finger protein 774
1.06	MRGPRF	MAS-related GPR. member F
1.06	ALDH1A2	aldehyde dehydrogenase 1 family. member A2
1.06	OR9Q1	olfactory receptor. family 9. subfamily Q. member 1
1.06	FTH1	ferritin. heavy polypeptide 1
1.06	ZNF408	zinc finger protein 408
1.06	RBM33	RNA binding motif protein 33
1.06	KLHL3	kelch-like family member 3
1.06	ABCD2	ATP binding cassette subfamily D member 2
1.06	RGS16	regulator of G-protein signaling 16
1.06	AR	androgen receptor
1.06	SPO11	SPO11 meiotic protein covalently bound to DSB
1.06	UCHL1	ubiquitin C-terminal hydrolase L1

1.06	SLC25A16	solute carrier family 25 (mitochondrial carrier). member 16
1.06	C2orf44	chromosome 2 open reading frame 44
1.06	SLC23A2	solute carrier family 23 (ascorbic acid transporter). member 2
1.06	SIPA1L1	signal-induced proliferation-associated 1 like 1
1.06	TAGAP	T-cell activation RhoGTPase activating protein
1.06	UBXN8	UBX domain protein 8
1.06	LYPD6B	LY6/PLAUR domain containing 6B
1.06	ARHGAP15	Memczak2013 ALT_ACCEPTOR. ALT_DONOR. coding. INTERNAL. intronic best transcript NM_018460
1.06	USP17L3; USP17L8	ubiquitin specific peptidase 17-like family member 3; ubiquitin specific peptidase 17-like family member 8
1.06	UGT2A3	UDP glucuronosyltransferase 2 family. polypeptide A3
1.06	PSAT1	phosphoserine aminotransferase 1
1.06	CMTM7	CKLF-like MARVEL transmembrane domain containing 7
1.06	CLEC19A	C-type lectin domain family 19. member A
1.06	LSM1	LSM1 homolog. mRNA degradation associated
1.06	OR51A7	olfactory receptor. family 51. subfamily A. member 7
1.06	SAG	S-antigen; retina and pineal gland (arrestin)
1.06	GDAP1	ganglioside induced differentiation associated protein 1
1.06	HP09025; CTD-2116F7.1	uncharacterized LOC100652929; novel transcript; uncharacterized LOC100652929 [Source:EntrezGene;Acc:100652929]
1.06	C10orf126	chromosome 10 open reading frame 126
1.06	PIGA	phosphatidylinositol glycan anchor biosynthesis class A
1.06	ACTRT2	actin-related protein T2
1.06	TADA2A	transcriptional adaptor 2A
1.06	TMEM211	transmembrane protein 211
1.06	CRK	v-crk avian sarcoma virus CT10 oncogene homolog

1.06	PSMB1	proteasome subunit beta 1
1.06	TUFM; MIR4721	Tu translation elongation factor. mitochondrial; microRNA 4721
1.06	ZNF649	zinc finger protein 649
1.06	B3GLCT	beta 3-glucosyltransferase
1.06	DNAJC17	DnaJ (Hsp40) homolog. subfamily C. member 17
1.06	RAI1	retinoic acid induced 1
1.06	RCC1; SNORA73A; SNHG3; SNORA73B	regulator of chromosome condensation 1; small nucleolar RNA. H/ACA box 73A; small nucleolar RNA host gene 3; small nucleolar RNA. H/ACA box 73B
1.06	ANXA2	annexin A2
1.06	MAP2K1	mitogen-activated protein kinase kinase 1
1.06	FOLH1B	folate hydrolase 1B
1.06	ITPKB	inositol-trisphosphate 3-kinase B
1.06	ELAVL4	ELAV like neuron-specific RNA binding protein 4
1.06	MYL7	myosin light chain 7
1.06	OR1A2	olfactory receptor. family 1. subfamily A. member 2
1.06	SSPN	sarcospan
1.06	FAM8A1	family with sequence similarity 8. member A1
1.06	RTFDC1	replication termination factor 2 domain containing 1
1.06	TEX43	testis expressed 43
1.06	C22orf15	chromosome 22 open reading frame 15
1.06	FCAR	Fc fragment of IgA receptor
1.06	INPP5D	inositol polyphosphate-5-phosphatase D
1.06	SLC17A2	solute carrier family 17. member 2
1.06	EIF3F	Eukaryotic translation initiation factor 3 subunit F [Source:UniProtKB/Swiss-Prot;Acc:O00303]
1.06	HOXD3	homeobox D3
1.06	HESX1	HESX homeobox 1

1.06	TDP1	tyrosyl-DNA phosphodiesterase 1
1.06	COL20A1	collagen. type XX. alpha 1
1.06	YEATS2	YEATS domain containing 2
1.06	RHOF	ras homolog family member F (in filopodia)
1.06	GOLGA8S	golgin A8 family. member S
1.06	ALPL	alkaline phosphatase. liver/bone/kidney
1.06	STIM2	stromal interaction molecule 2
1.06	BCO1	beta-carotene oxygenase 1
1.06	HNRNPA1	heterogeneous nuclear ribonucleoprotein A1
1.06	SUGP2	SURP and G-patch domain containing 2
1.06	GPALPP1	GPALPP motifs containing 1
1.06	ARSK	arylsulfatase family. member K
1.06	ADGRF2	adhesion G protein-coupled receptor F2
1.06	TMEM26	transmembrane protein 26
1.06	ANKRD66	ankyrin repeat domain 66
1.06	C4orf51	chromosome 4 open reading frame 51
1.06	KDELR3	KDEL (Lys-Asp-Glu-Leu) endoplasmic reticulum protein retention receptor 3
1.06	HSD17B2	hydroxysteroid (17-beta) dehydrogenase 2
1.06	SLC25A26	solute carrier family 25 (S-adenosylmethionine carrier). member 26
1.06	KHDC3L	KH domain containing 3-like. subcortical maternal complex member
1.06	ZNF121	zinc finger protein 121
1.06	FBL	fibrillarin
1.06	TRIM37	Transcript Identified by AceView. Entrez Gene ID(s) 4591
1.06	APCDD1	adenomatosis polyposis coli down-regulated 1
1.06	CCDC157	coiled-coil domain containing 157
1.06	C22orf29; GNB1L	chromosome 22 open reading frame 29; guanine nucleotide binding protein

		(G protein). beta polypeptide 1-like
1.06	CD19	CD19 molecule
1.06	GCNT7	glucosaminyl (N-acetyl) transferase family member 7
1.06	MINA	MYC induced nuclear antigen
1.06	BIRC3	Memczak2013 ANTISENSE. CDS. coding. INTERNAL best transcript NM_182962
1.06	TVP23B	trans-golgi network vesicle protein 23 homolog B (S. cerevisiae)
1.06	CHRNA5	cholinergic receptor. nicotinic alpha 5
1.06	TRAPPC4	trafficking protein particle complex 4
1.06	CSK	c-src tyrosine kinase
1.06	MLF1	myeloid leukemia factor 1
1.06	FAM216B	family with sequence similarity 216. member B
1.06	RRAGC	Ras-related GTP binding C
1.06	ZDHHC14	zinc finger. DHHC-type containing 14
1.06	PTH	parathyroid hormone
1.06	DSTN	destrin (actin depolymerizing factor)
1.06	TRAPPC4	trafficking protein particle complex 4
1.06	TIMD4	T-cell immunoglobulin and mucin domain containing 4
1.06	ATP1A4	ATPase. Na+/K+ transporting. alpha 4 polypeptide
1.06	CTBP2	Transcript Identified by AceView. Entrez Gene ID(s) 1488
1.06	SMARCB1	SWI/SNF related. matrix associated. actin dependent regulator of chromatin. subfamily b. member 1
1.06	KIF1C	kinesin family member 1C
1.06	OR11H7	olfactory receptor. family 11. subfamily H. member 7 (gene/pseudogene)
1.06	AFF4	AF4/FMR2 family. member 4
1.06	CASKIN2	CASK interacting protein 2
1.06	TAP2	transporter 2. ATP-binding cassette. sub-family B (MDR/TAP)

1.06	HDAC6	Memczak2013 ANTISENSE. CDS. coding. INTERNAL best transcript NM_006044
1.06	PLRG1	pleiotropic regulator 1
1.06	L1TD1	LINE-1 type transposase domain containing 1
1.06	CYP11B1	cytochrome P450. family 11. subfamily B. polypeptide 1
1.06	TIRAP	toll-interleukin 1 receptor (TIR) domain containing adaptor protein
1.06	PRAMEF8	PRAME family member 8
1.06	CPLX4	complexin 4
1.06	DUSP3	dual specificity phosphatase 3
1.06	POU6F1	POU class 6 homeobox 1
1.06	MERTK	MER proto-oncogene. tyrosine kinase
1.06	HIST1H2BC	histone cluster 1. H2bc
1.06	GLYATL2	glycine-N-acyltransferase-like 2
1.06	DHX37	DEAH (Asp-Glu-Ala-His) box polypeptide 37
1.06	CPQ	carboxypeptidase Q
1.06	OR52I2	olfactory receptor. family 52. subfamily I. member 2
1.06	ZNF621	zinc finger protein 621
1.06	PGBD4	piggyBac transposable element derived 4
1.06	C10orf95	chromosome 10 open reading frame 95
1.06	STAT6	signal transducer and activator of transcription 6. interleukin-4 induced
1.06	CCDC43	coiled-coil domain containing 43
1.06	ZMYND10	zinc finger. MYND-type containing 10
1.06	MRPS21	mitochondrial ribosomal protein S21
1.06	OR2AG1	olfactory receptor. family 2. subfamily AG. member 1 (gene/pseudogene)
1.06	RLF	rearranged L-myc fusion
1.06	MMACHC	methylmalonic aciduria (cobalamin deficiency) cblC type. with homocystinuria

1.06	MEPCE	methylphosphate capping enzyme
1.06	C15orf41	chromosome 15 open reading frame 41
1.06	MFAP3L	microfibrillar associated protein 3 like
1.06	BAG2	BCL2-associated athanogene 2
1.06	SNORD89; RNF149	small nucleolar RNA. C/D box 89; ring finger protein 149
1.06	TMOD2	tropomodulin 2 (neuronal)
1.06	OR51M1	olfactory receptor. family 51. subfamily M. member 1
1.06	OR5D16	olfactory receptor. family 5. subfamily D. member 16
1.06	PP14571; AC110619.2	uncharacterized LOC100130449; novel transcript. antisense to GPC1; Transcript Identified by AceView. Entrez Gene ID(s) 100130449; uncharacterized LOC100130449 [Source:EntrezGene;Acc:100130449]
1.06	RRP8	ribosomal RNA processing 8. methyltransferase. homolog (yeast)
1.06	DNAJC11	DnaJ (Hsp40) homolog. subfamily C. member 11
1.06	FMO2	flavin containing monooxygenase 2
1.06	CCL18	chemokine (C-C motif) ligand 18
1.06	ABTB1	ankyrin repeat and BTB (POZ) domain containing 1
1.05	CDH26	cadherin 26
1.05	SPSB3	splA/ryanodine receptor domain and SOCS box containing 3
1.05	RAD50	RAD50 homolog. double strand break repair protein
1.05	FOXP2	forkhead box P2
1.05	DEFB135	defensin. beta 135
1.05	RAPGEF2	Rap guanine nucleotide exchange factor 2
1.05	CCNK	cyclin K
1.05	PDAP1	PDGFA associated protein 1
1.05	KRT75	keratin 75. type II
1.05	NUFIP2	nuclear fragile X mental retardation protein interacting protein 2
1.05	P4HA3	prolyl 4-hydroxylase. alpha polypeptide III

1.05	PSG6	pregnancy specific beta-1-glycoprotein 6
1.05	OR2T12	olfactory receptor. family 2. subfamily T. member 12
1.05	SLC17A3; HIST1H2APS2	solute carrier family 17 (organic anion transporter). member 3; histone cluster 1. H2a. pseudogene 2
1.05	NR1I3	nuclear receptor subfamily 1. group I. member 3
1.05	SMAP2	small ArfGAP2
1.05	PKM	pyruvate kinase. muscle
1.05	PGPEP1	pyroglutamyl-peptidase I
1.05	ABLIM2	actin binding LIM protein family. member 2
1.05	EIF4E1B	eukaryotic translation initiation factor 4E family member 1B
1.05	PER2	period circadian clock 2
1.05	KRTAP6-1	keratin associated protein 6-1
1.05	ATG2B	autophagy related 2B
1.05	DGKE	diacylglycerol kinase. epsilon 64kDa
1.05	C15orf61	chromosome 15 open reading frame 61
1.05	ITPKC	inositol-trisphosphate 3-kinase C
1.05	CARKD	carbohydrate kinase domain containing
1.05	ATG16L1	autophagy related 16-like 1
1.05	KIAA2012	KIAA2012
1.05	GOLGA6L2	golgin A6 family-like 2
1.05	RPS7	ribosomal protein S7
1.05	EIF4G2; SNORD97	eukaryotic translation initiation factor 4 gamma. 2; small nucleolar RNA. C/D box 97
1.05	SLC52A2	solute carrier family 52 (riboflavin transporter). member 2
1.05	HOXB7	homeobox B7
1.05	DNAJA4	DnaJ (Hsp40) homolog. subfamily A. member 4
1.05	OR10A6	olfactory receptor family 10 subfamily A member 6 (gene/pseudogene)

		[Source:HGNC Symbol;Acc:HGNC:15132]
1.05	POLR3GL	polymerase (RNA) III (DNA directed) polypeptide G (32kD)-like
1.05	TGM4	transglutaminase 4
1.05	LYPLA1	lysophospholipase I
1.05	SFMBT1	Scm-like with four mbt domains 1
1.05	NMT1	N-myristoyltransferase 1
1.05	DIP2C	disco-interacting protein 2 homolog C
1.05	POLR1E	polymerase (RNA) I polypeptide E
1.05	FLG	filaggrin
1.05	POTEM; POTEG	POTE ankyrin domain family. member M; POTE ankyrin domain family. member G
1.05	SGPL1	sphingosine-1-phosphate lyase 1
1.05	SCAF4	SR-related CTD-associated factor 4
1.05	GPR85	G protein-coupled receptor 85
1.05	VSTM1	V-set and transmembrane domain containing 1
1.05	OR51D1	olfactory receptor. family 51. subfamily D. member 1
1.05	NPLOC4	NPL4 homolog. ubiquitin recognition factor
1.05	PDE8A	phosphodiesterase 8A
1.05	FAM212B	family with sequence similarity 212. member B
1.05	KDR	kinase insert domain receptor
1.05	RNF151	ring finger protein 151
1.05	TMEM132C	transmembrane protein 132C
1.05	OR6S1	olfactory receptor. family 6. subfamily S. member 1
1.05	ZBED3	zinc finger. BED-type containing 3
1.05	THOC5	THO complex 5
1.05	SCGB1C1	secretoglobin. family 1C. member 1

1.05	PDE4D	Memczak2013 ANTISENSE. coding. INTERNAL. intronic best transcript NM_001165899
1.05	THSD4	Transcript Identified by AceView. Entrez Gene ID(s) 79875
1.05	OR1E2	olfactory receptor. family 1. subfamily E. member 2
1.05	NDUFA10	NADH dehydrogenase (ubiquinone) 1 alpha subcomplex. 10. 42kDa
1.05	HOXC6; HOXC4; HOXC5	homeobox C6; homeobox C4; homeobox C5
1.05	SAP30BP	SAP30 binding protein
1.05	SSR3	signal sequence receptor. gamma (translocon-associated protein gamma)
1.05	ENY2	enhancer of yellow 2 homolog (Drosophila)
1.05	OLFML2A	olfactomedin like 2A
1.05	DHX35	Memczak2013 ANTISENSE. coding. INTERNAL. intronic best transcript NM_021931
1.05	KRTAP3-3	keratin associated protein 3-3
1.05	GPNMB	glycoprotein (transmembrane) nmb
1.05	ALG6	ALG6. alpha-1.3-glucosyltransferase
1.05	MED16	mediator complex subunit 16
1.05	MGST2	microsomal glutathione S-transferase 2
1.05	TRIM35	tripartite motif containing 35
1.05	PLA2G7	phospholipase A2. group VII (platelet-activating factor acetylhydrolase. plasma)
1.05	OBP2B	odorant binding protein 2B
1.05	RAPGEF5	Rap guanine nucleotide exchange factor 5
1.05	DIRC2	disrupted in renal carcinoma 2
1.05	PABPN1L	poly(A) binding protein. nuclear 1-like (cytoplasmic)
1.05	KIF3A	kinesin family member 3A
1.05	FAM46A	family with sequence similarity 46. member A
1.05	ACIN1	apoptotic chromatin condensation inducer 1

1.05	ST8SIA2	ST8 alpha-N-acetyl-neuraminide alpha-2.8-sialyltransferase 2
1.05	ATG16L1	autophagy related 16-like 1
1.05	RPL41P5	ribosomal protein L41 pseudogene 5
1.05	RNF212B	ring finger protein 212B
1.05	CFAP70	cilia and flagella associated protein 70
1.05	TCFL5	transcription factor-like 5 (basic helix-loop-helix)
1.05	GPR17	G protein-coupled receptor 17
1.05	TRIM49	tripartite motif containing 49
1.05	AFG3L2	AFG3-like AAA ATPase 2
1.05	DUX4L8	double homeobox 4 like 8
1.05	VSIG8	V-set and immunoglobulin domain containing 8
1.05	FZD9	frizzled class receptor 9
1.05	FOXP3	forkhead box P3
1.05	NDUFA1	NADH dehydrogenase (ubiquinone) 1 alpha subcomplex. 1. 7.5kDa
1.05	F2RL3	coagulation factor II (thrombin) receptor-like 3
1.05	IMPACT	impact RWD domain protein
1.05	SMAD2	SMAD family member 2
1.05	SP7	Sp7 transcription factor
1.05	KIF26B	kinesin family member 26B
1.05	SSU72	SSU72 homolog. RNA polymerase II CTD phosphatase
1.05	ARHGAP15	Rho GTPase activating protein 15
1.05	LGALS16	lectin. galactoside-binding. soluble. 16
1.05	SCP2D1	SCP2 sterol-binding domain containing 1
1.05	PIK3R4	phosphoinositide-3-kinase. regulatory subunit 4
1.05	TAB2	TGF-beta activated kinase 1/MAP3K7 binding protein 2
1.05	GOLGA8N	golgin A8 family. member N

1.05	PSMD7	proteasome 26S subunit. non-ATPase 7
1.05	MPPED2	metallophosphoesterase domain containing 2
1.05	HERPUD2	HERPUD family member 2
1.05	HIST1H4E	histone cluster 1. H4e
1.05	FAM210A	family with sequence similarity 210. member A
1.05	TCERG1	Transcript Identified by AceView. Entrez Gene ID(s) 10915
1.05	PPP1R3A	protein phosphatase 1. regulatory subunit 3A
1.05	NFYA	nuclear transcription factor Y subunit alpha
1.05	HK2	hexokinase 2
1.05	NFU1	NFU1 iron-sulfur cluster scaffold
1.05	LMBRD1	LMBR1 domain containing 1
1.05	APCS	amyloid P component. serum
1.05	ZC3H13	Memczak2013 ANTISENSE. CDS. coding. INTERNAL best transcript NM_015070
1.05	CABYR	calcium binding tyrosine-(Y)-phosphorylation regulated
1.05	MEST	mesoderm specific transcript
1.05	CPAMD8	C3 and PZP-like. alpha-2-macroglobulin domain containing 8
1.05	TP53BP1	tumor protein p53 binding protein 1
1.05	KRTAP4-8	keratin associated protein 4-8
1.05	WNT5A	wingless-type MMTV integration site family. member 5A
1.05	LDLRAD2	low density lipoprotein receptor class A domain containing 2
1.05	FMO1	flavin containing monooxygenase 1
1.05	ELAC1	elaC ribonuclease Z 1
1.05	UBXN1	UBX domain protein 1
1.05	ACYP1	acylphosphatase 1. erythrocyte (common) type
1.05	UBE2G1	ubiquitin conjugating enzyme E2G 1

1.05	DEFB1	defensin. beta 1
1.05	GPR68	G protein-coupled receptor 68
1.05	SOX7	SRY box 7
1.05	C5orf56	chromosome 5 open reading frame 56
1.05	TRIL	TLR4 interactor with leucine-rich repeats
1.05	GRM1	glutamate receptor. metabotropic 1
1.05	NOL6	nucleolar protein 6 (RNA-associated)
1.05	PTPN20	protein tyrosine phosphatase. non-receptor type 20
1.05	DPH2	DPH2 homolog
1.05	FPGT	fucose-1-phosphate guanylyltransferase
1.05	RESP18	regulated endocrine-specific protein 18
1.05	TCTEX1D2	Tctex1 domain containing 2
1.05	FGF20	fibroblast growth factor 20
1.05	PATE1	prostate and testis expressed 1
1.05	SRD5A1	steroid-5-alpha-reductase. alpha polypeptide 1 (3-oxo-5 alpha-steroid delta 4-dehydrogenase alpha 1)
1.05	TRRAP	transformation/transcription domain-associated protein
1.05	GPRC5B	G protein-coupled receptor. class C. group 5. member B
1.05	CLEC4M	C-type lectin domain family 4. member M
1.05	CT55	cancer/testis antigen 55
1.05	B2M	beta-2-microglobulin
1.05	KDM1A	Transcript Identified by AceView. Entrez Gene ID(s) 23028
1.05	SALL3	spalt-like transcription factor 3
1.05	CLASRP	CLK4-associating serine/arginine rich protein
1.05	LKAAEAR1	LKAAEAR motif containing 1
1.05	TMEM65	transmembrane protein 65

1.05	EID2B	EP300 interacting inhibitor of differentiation 2B
1.05	ZNF174	zinc finger protein 174
1.05	ADK; MRPL35P3	adenosine kinase; mitochondrial ribosomal protein L35 pseudogene 3
1.05	GPR143	G protein-coupled receptor 143
1.05	ZNF20	zinc finger protein 20
1.05	GPR174	G protein-coupled receptor 174
1.05	UGT2B7	UDP glucuronosyltransferase 2 family. polypeptide B7
1.05	SLC35F5	solute carrier family 35. member F5
1.05	RIMBP3C; RIMBP3B	RIMS binding protein 3C; RIMS binding protein 3B
1.05	MORN1	MORN repeat containing 1
1.05	SLC35E4	solute carrier family 35. member E4
1.05	MBD2	methyl-CpG binding domain protein 2
1.05	FAM193A	family with sequence similarity 193. member A
1.05	CXCL13	chemokine (C-X-C motif) ligand 13
1.05	ATP5J2	ATP synthase. H+ transporting. mitochondrial Fo complex subunit F2
1.05	MAFF	v-maf avian musculoaponeurotic fibrosarcoma oncogene homolog F
1.05	CYB5D2	Transcript Identified by AceView. Entrez Gene ID(s) 124936
1.05	LOC55338; CTC-242N15.1	uncharacterized LOC55338; novel transcript
1.05	KAT8	K(lysine) acetyltransferase 8
1.05	SOD3	superoxide dismutase 3. extracellular
1.05	MFN2	mitofusin 2
1.05	SLC26A8	solute carrier family 26 (anion exchanger). member 8
1.05	JAM2	junctional adhesion molecule 2
1.05	LCE3A	late cornified envelope 3A
1.05	LOC401052; AC022007.5; TMEM111	uncharacterized LOC401052; Salzman2013 ANNOTATED. coding. INTERNAL. OVERLAPTX. OVEXON. UTR5 best transcript NM_001008737; novel transcript; Transcript Identified by AceView. Entrez Gene ID(s) 401052;

		55831
1.05	SLC25A15; MIR621	solute carrier family 25 (mitochondrial carrier; ornithine transporter) member 15; microRNA 621
1.05	NXT1	nuclear transport factor 2-like export factor 1
1.05	OR2A1	olfactory receptor. family 2. subfamily A. member 1
1.05	RIMS2	regulating synaptic membrane exocytosis 2
1.05	SPDYE4	speedy/RINGO cell cycle regulator family member E4
1.05	EPSTI1	epithelial stromal interaction 1 (breast)
1.05	ZFAND3	zinc finger. AN1-type domain 3
1.05	NTS	neurotensin
1.05	HEPH	hephaestin
1.05	VPS8	Transcript Identified by AceView. Entrez Gene ID(s) 23355
1.05	HSD17B8	hydroxysteroid (17-beta) dehydrogenase 8
1.05	ZNF79	zinc finger protein 79
1.05	GPSM2	G-protein signaling modulator 2
1.05	AKNA	AT-hook transcription factor
1.05	WFDC10A	WAP four-disulfide core domain 10A
1.05	SMIM18	small integral membrane protein 18
1.05	PLTP	phospholipid transfer protein
1.05	HACD2	3-hydroxyacyl-CoA dehydratase 2
1.05	FTHL17	ferritin. heavy polypeptide-like 17
1.05	CXCL11	chemokine (C-X-C motif) ligand 11
1.05	ZNF141	zinc finger protein 141
1.05	DNAH7	dynein. axonemal. heavy chain 7
1.05	AOC2	amine oxidase. copper containing 2 (retina-specific)
1.05	ZFHX4	zinc finger homeobox 4

1.05	PKP3	plakophilin 3
1.05	NKIRAS2	Memczak2013 ANTISENSE. coding. INTERNAL. UTR3 best transcript NM_001001349
1.05	CELA3B	chymotrypsin-like elastase family. member 3B
1.05	XRCC6	X-ray repair complementing defective repair in Chinese hamster cells 6
1.05	P2RX2	purinergic receptor P2X. ligand gated ion channel. 2
1.05	ABHD12B	abhydrolase domain containing 12B
1.05	EIF3F	eukaryotic translation initiation factor 3. subunit F
1.05	PGBD1	piggyBac transposable element derived 1
1.05	UTS2B	urotensin 2B
1.05	NFE2L1	nuclear factor. erythroid 2-like 1
1.05	RPL35	ribosomal protein L35
1.05	ADRA2C	adrenoceptor alpha 2C
1.05	ARHGEF40	Rho guanine nucleotide exchange factor (GEF) 40
1.05	SOX4	SRY box 4
1.05	LCN15	lipocalin 15
1.05	FANCE	Fanconi anemia complementation group E
1.05	SNX6	sorting nexin 6
1.05	ACTN1; HMGN1P3	actinin. alpha 1; high mobility group nucleosome binding domain 1 pseudogene 3
1.05	ICK	intestinal cell (MAK-like) kinase
1.05	FAM231A; FAM231C; RP5-1182A14.6	Homo sapiens family with sequence similarity 231. member A (FAM231A). mRNA.; Homo sapiens family with sequence similarity 231. member C (FAM231C). mRNA.; Protein FAM231A/C [Source:UniProtKB/Swiss-Prot;Acc:A6NEA5]
1.05	SMG6	SMG6 nonsense mediated mRNA decay factor
1.05	PCDHB5	protocadherin beta 5
1.05	CALML6	calmodulin-like 6

1.05	NCKAP1	NCK-associated protein 1
1.05	TP53	tumor protein p53
1.05	TIMM23	translocase of inner mitochondrial membrane 23 homolog (yeast)
1.05	LGALS7; LGALS7B	lectin. galactoside-binding. soluble. 7; lectin. galactoside-binding. soluble. 7B
1.05	USP3	Memczak2013 ALT_ACCEPTOR. ALT_DONOR. coding. INTERNAL. intronic best transcript NM_006537
1.05	TRIM59	tripartite motif containing 59
1.05	GLP2R	glucagon-like peptide 2 receptor
1.05	CCL25	chemokine (C-C motif) ligand 25
1.05	OR52E4	olfactory receptor. family 52. subfamily E. member 4
1.05	NPIPA1	nuclear pore complex interacting protein family. member A1
1.05	ZNF627	zinc finger protein 627
1.05	ZNF658B	zinc finger protein 658B. pseudogene
1.05	PRR23C	proline rich 23C
1.05	GTPBP10	GTP-binding protein 10 (putative)
1.05	CCAR1; SNORD98	cell division cycle and apoptosis regulator 1; small nucleolar RNA. C/D box 98
1.05	SMG7	SMG7 nonsense mediated mRNA decay factor
1.05	NDUFV2	NADH dehydrogenase (ubiquinone) flavoprotein 2. 24kDa
1.05	NCOA3	nuclear receptor coactivator 3
1.05	SCUBE2	signal peptide. CUB domain. EGF-like 2
1.05	SVOPL	SVOP-like
1.05	OBP2B	odorant binding protein 2B
1.05	TMEM147	transmembrane protein 147
1.05	SDHAF2	Memczak2013 ALT_ACCEPTOR. ALT_DONOR. coding. INTERNAL. intronic best transcript NM_017841
1.05	PRKG1	Memczak2013 ALT_ACCEPTOR. ALT_DONOR. coding. INTERNAL. intronic best transcript NM_001098512

1.05	PROL1	proline rich. lacrimal 1
1.05	MAP3K19	mitogen-activated protein kinase kinase kinase 19
1.05	TMEM229B	transmembrane protein 229B
1.05	JMJD7; PLA2G4B; JMJD7-PLA2G4B	jumonji domain containing 7; phospholipase A2. group IVB (cytosolic); JMJD7-PLA2G4B readthrough
1.05	CA7	carbonic anhydrase VII
1.05	FZD10	frizzled class receptor 10
1.05	CASP8	caspase 8. apoptosis-related cysteine peptidase
1.05	NOM1	nucleolar protein with MIF4G domain 1
1.05	MAP3K5	mitogen-activated protein kinase kinase kinase 5
1.05	FAM173B	Transcript Identified by AceView. Entrez Gene ID(s) 134145
1.05	SOD3	superoxide dismutase 3. extracellular
1.05	TRIM72	tripartite motif containing 72. E3 ubiquitin protein ligase
1.05	TEKT4P2	tektin 4 pseudogene 2
1.05	DYSF; kydor	Memczak2013 ANTISENSE. coding. INTERNAL. UTR5 best transcript NM_003494; Transcript Identified by AceView
1.05	CPEB1	cytoplasmic polyadenylation element binding protein 1
1.05	PSORS1C1	psoriasis susceptibility 1 candidate 1
1.05	OR1J4	olfactory receptor. family 1. subfamily J. member 4
1.05	HSD17B10	hydroxysteroid (17-beta) dehydrogenase 10
1.05	THUMPD1	THUMP domain containing 1
1.05	ADGRE3	adhesion G protein-coupled receptor E3
1.05	AKAP13; MIR7706	A kinase (PRKA) anchor protein 13; microRNA 7706
1.05	SYMPK	symplekin
1.05	VGLL1	vestigial-like family member 1
1.05	PAFAH1B2	platelet-activating factor acetylhydrolase 1b. catalytic subunit 2 (30kDa)
1.05	PNMT	phenylethanolamine N-methyltransferase

1.05	PDP1	pyruvate dehyrogenase phosphatase catalytic subunit 1
1.05	H2AFY2	H2A histone family. member Y2
1.05	PITPNA	phosphatidylinositol transfer protein. alpha
1.05	PKD1L1	polycystic kidney disease 1 like 1
1.05	NHLRC3	NHL repeat containing 3
1.05	GRM7	glutamate receptor. metabotropic 7
1.05	INS; INS-IGF2	insulin; INS-IGF2 readthrough
1.05	HPS4	Hermansky-Pudlak syndrome 4
1.05	SSX3	synovial sarcoma. X breakpoint 3
1.05	KIAA1429	KIAA1429
1.05	BHLHE22	basic helix-loop-helix family. member e22
1.05	SLC6A11	solute carrier family 6 (neurotransmitter transporter). member 11
1.05	EML3	echinoderm microtubule associated protein like 3
1.05	ZSWIM5	zinc finger. SWIM-type containing 5
1.05	KCNG1	potassium channel. voltage gated modifier subfamily G. member 1
1.05	POLH	polymerase (DNA directed). eta
1.05	OR4E1	olfactory receptor family 4 subfamily E member 1 (gene/pseudogene) [Source:HGNC Symbol;Acc:HGNC:8296]
1.05	JKAMP	JNK1/MAPK8-associated membrane protein
1.05	CAMK2B	Transcript Identified by AceView. Entrez Gene ID(s) 816
1.05	SHCBP1L	SHC SH2-domain binding protein 1-like
1.05	GORAB	golgin. RAB6-interacting
1.05	RUBCN; MIR922	RUN domain and cysteine-rich domain containing. Beclin 1-interacting protein; microRNA 922
1.05	CCDC105	coiled-coil domain containing 105
1.05	AC171558.1	Olfactory receptor 4N4 [Source:UniProtKB/Swiss-Prot;Acc:Q8N0Y3]
1.05	SLC7A8	solute carrier family 7 (amino acid transporter light chain. L system).

		member 8
1.05	AP1S1	adaptor-related protein complex 1 sigma 1 subunit
1.05	CAMK1G	calcium/calmodulin-dependent protein kinase IG
1.05	FBXO7	F-box protein 7
1.05	DNAJB8	DnaJ (Hsp40) homolog. subfamily B. member 8
1.05	DOCK6	dedicator of cytokinesis 6
1.05	IFFO2	intermediate filament family orphan 2
1.05	SEC23B	Sec23 homolog B. COPII coat complex component
1.05	ZKSCAN7	zinc finger with KRAB and SCAN domains 7
1.05	RASL10A	RAS-like. family 10. member A
1.05	CEACAM21	carcinoembryonic antigen-related cell adhesion molecule 21
1.05	ZNHIT2	zinc finger. HIT-type containing 2
1.05	C4orf50	chromosome 4 open reading frame 50
1.05	CRTC2	CREB regulated transcription coactivator 2
1.05	TTC36	tetratricopeptide repeat domain 36
1.05	ITGB6	integrin beta 6
1.05	SCGB1A1	secretoglobin. family 1A. member 1 (uteroglobin)
1.05	USP17L12	ubiquitin specific peptidase 17-like family member 12
1.05	OR1K1	olfactory receptor. family 1. subfamily K. member 1
1.05	MTNR1B	melatonin receptor 1B
1.05	LYPD6	LY6/PLAUR domain containing 6
1.05	CROCC	Zhang2013 ALT_ACCEPTOR. ALT_DONOR. coding. INTERNAL. intronic best transcript NM_014675
1.05	KIAA0100	KIAA0100
1.05	NDUFA10	NADH dehydrogenase (ubiquinone) 1 alpha subcomplex. 10. 42kDa
1.05	S100B	S100 calcium binding protein B

1.05	TP53TG3B	TP53 target 3B
1.05	CHD9	Transcript Identified by AceView. Entrez Gene ID(s) 80205
1.05	HSPA14	heat shock 70kDa protein 14
1.05	LPAL2	lipoprotein. Lp(a)-like 2. pseudogene
1.05	LINC00269	long intergenic non-protein coding RNA 269
1.05	TPT1; SNORA31	tumor protein. translationally-controlled 1; small nucleolar RNA. H/ACA box 31
1.05	PGRMC1	Memczak2013 ALT_ACCEPTOR. ALT_DONOR. coding. INTERNAL. intronic best transcript NM_006667
1.05	MAGEB1; MAGEB4	MAGE family member B1; MAGE family member B4
1.05	TTC7B	tetratricopeptide repeat domain 7B
1.05	SAG	S-antigen; retina and pineal gland (arrestin)
1.05	XRRA1	X-ray radiation resistance associated 1
1.05	MXRA7	matrix-remodelling associated 7
1.05	ANKRD20A1; ANKRD20A3	ankyrin repeat domain 20 family. member A1; ankyrin repeat domain 20 family. member A3
1.05	MPV17L	MPV17 mitochondrial membrane protein-like
1.05	GIMD1	GIMAP family P-loop NTPase domain containing 1
1.05	ATP6V0A2	ATPase. H+ transporting. lysosomal V0 subunit a2
1.05	COL11A1	collagen. type XI. alpha 1
1.05	NDUFB8	NADH dehydrogenase (ubiquinone) 1 beta subcomplex. 8. 19kDa
1.05	CBLN2	cerebellin 2 precursor
1.05	TNIP1	TNFAIP3 interacting protein 1
1.05	MAN1A1	mannosidase. alpha. class 1A. member 1
1.05	CLIC5	chloride intracellular channel 5
1.05	RRP7A	ribosomal RNA processing 7 homolog A
1.05	CYP4A11	cytochrome P450. family 4. subfamily A. polypeptide 11

1.05	VPS8	Transcript Identified by AceView. Entrez Gene ID(s) 23355
1.05	SLC45A3	solute carrier family 45. member 3
1.05	ZNF780A	zinc finger protein 780A
1.05	ARHGDIA	Rho GDP dissociation inhibitor (GDI) alpha
1.05	PRELID3B; ATP5E	PRELI domain containing 3B; ATP synthase. H+ transporting. mitochondrial F1 complex. epsilon subunit
1.05	FUT5	fucosyltransferase 5 (alpha (1.3) fucosyltransferase)
1.05	SCNM1; TNFAIP8L2	sodium channel modifier 1; tumor necrosis factor. alpha-induced protein 8-like 2
1.05	GORASP2	golgi reassembly stacking protein 2
1.05	EIF3C; EIF3CL	eukaryotic translation initiation factor 3. subunit C; eukaryotic translation initiation factor 3. subunit C-like
1.05	CABS1	calcium-binding protein. spermatid-specific 1
1.05	CUL7	cullin 7
1.05	ANO4	anoctamin 4
1.05	C10orf113	chromosome 10 open reading frame 113
1.05	PIP5K1A	phosphatidylinositol-4-phosphate 5-kinase. type I. alpha
1.05	HIST1H4H	histone cluster 1. H4h
1.05	CEMP1	cementum protein 1
1.05	KCNG4	potassium channel. voltage gated modifier subfamily G. member 4
1.05	ITGB4	integrin beta 4
1.05	HNRNPA1P33; LINC00842; ANXA8L1	heterogeneous nuclear ribonucleoprotein A1 pseudogene 33; long intergenic non-protein coding RNA 842; annexin A8-like 1
1.05	SLC4A2	solute carrier family 4 (anion exchanger). member 2
1.05	RGPD6; RGPD5	RANBP2-like and GRIP domain containing 6; RANBP2-like and GRIP domain containing 5
1.05	EIF4A3	eukaryotic translation initiation factor 4A3
1.05	PRPSAP1	phosphoribosyl pyrophosphate synthetase-associated protein 1

1.05	MYH8	myosin. heavy chain 8. skeletal muscle. perinatal
1.05	NDUFA10	Memczak2013 ANTISENSE. coding. INTERNAL. intronic best transcript NM_004544
1.05	NEUROD6	neuronal differentiation 6
1.05	HOXC9	homeobox C9
1.05	YPEL4	yippee like 4
1.05	BLOC1S5	biogenesis of lysosomal organelles complex-1. subunit 5. muted
1.05	PROB1	proline-rich basic protein 1
1.05	SPATA17	spermatogenesis associated 17
1.05	ATP6AP1L	ATPase. H+ transporting. lysosomal accessory protein 1-like
1.05	PRDX3	peroxiredoxin 3
1.05	IFNA8	interferon. alpha 8
1.05	RSPO1	R-spondin 1
1.05	SSR2	signal sequence receptor. beta (translocon-associated protein beta)
1.05	SIRPD	signal-regulatory protein delta
1.05	KRT6B	keratin 6B. type II
1.05	FNIP1	folliculin interacting protein 1
1.05	A4GNT	alpha-1.4-N-acetylglucosaminyltransferase
1.05	C1orf141	chromosome 1 open reading frame 141
1.05	ESR2	estrogen receptor 2 (ER beta)
1.05	RYBP	RING1 and YY1 binding protein
1.05	EDDM3A	epididymal protein 3A
1.05	GTF3C1	general transcription factor IIIC subunit 1
1.05	ALOX12B	arachidonate 12-lipoxygenase. 12R type
1.05	TLE3	Memczak2013 ANTISENSE. coding. INTERNAL. intronic best transcript NM_001105192
1.05	INMT	indolethylamine N-methyltransferase

1.05	RPRD1A	regulation of nuclear pre-mRNA domain containing 1A
1.05	NBPF4	neuroblastoma breakpoint family. member 4
1.05	NAA15	N(alpha)-acetyltransferase 15. NatA auxiliary subunit
1.05	GRHL2	grainyhead-like transcription factor 2
1.05	CFHR2	complement factor H-related 2
1.05	BEST3	bestrophin 3
1.05	RBBP7	retinoblastoma binding protein 7
1.05	EFR3A	EFR3 homolog A
1.05	PLP1	proteolipid protein 1
1.05	ABHD14B	abhydrolase domain containing 14B
1.05	ATCAY	ataxia. cerebellar. Cayman type
1.05	MON2	MON2 homolog. regulator of endosome-to-Golgi trafficking
1.05	FREM3	FRAS1 related extracellular matrix 3
1.05	PCDH8	protocadherin 8
1.05	CEP164	centrosomal protein 164kDa
1.05	EIF6	eukaryotic translation initiation factor 6
1.05	PTGFRN	prostaglandin F2 receptor inhibitor
1.05	SOWAHB	sosondowah ankyrin repeat domain family member B
1.05	BCL2L14	BCL2-like 14 (apoptosis facilitator)
1.05	SEL1L	sel-1 suppressor of lin-12-like (C. elegans)
1.05	SLC41A2	solute carrier family 41 (magnesium transporter). member 2
1.05	FOXO3	forkhead box O3
1.05	SHISA6	shisa family member 6
1.05	EIF3C; EIF3CL	eukaryotic translation initiation factor 3. subunit C; eukaryotic translation initiation factor 3. subunit C-like
1.05	C1orf64	chromosome 1 open reading frame 64

1.05	RAVER2	ribonucleoprotein. PTB-binding 2
1.05	PRB4	proline-rich protein BstNI subfamily 4
1.05	FAM124B	family with sequence similarity 124 member B
1.05	MYH3	myosin. heavy chain 3. skeletal muscle. embryonic
1.05	JUNB	jun B proto-oncogene
1.05	BCAN	brevican
1.05	ZMYM5	zinc finger. MYM-type 5
1.05	CCDC79	coiled-coil domain containing 79
1.05	C1orf111	chromosome 1 open reading frame 111
1.05	FAM25C	family with sequence similarity 25. member C
1.05	RRAGD	Ras-related GTP binding D
1.05	GTSCR1	Gilles de la Tourette syndrome chromosome region. candidate 1 (non-protein coding)
1.05	GIMAP2	GTPase. IMAP family member 2
1.05	TARDBP	TAR DNA binding protein
1.05	OR5B21	olfactory receptor. family 5. subfamily B. member 21
1.05	C3	complement component 3
1.05	OTOP1	otopetrin 1
1.05	RPL37A	ribosomal protein L37a
1.05	C17orf77	chromosome 17 open reading frame 77
1.05	NDUFA10	NADH dehydrogenase (ubiquinone) 1 alpha subcomplex. 10. 42kDa
1.05	DAZAP1	DAZ associated protein 1
1.05	OR14J1	olfactory receptor. family 14. subfamily J. member 1
1.05	NUBP2	nucleotide binding protein 2
1.05	OR56A1	olfactory receptor. family 56. subfamily A. member 1
1.05	EFNA1	ephrin-A1

1.05	AP5B1	adaptor-related protein complex 5. beta 1 subunit
1.05	TP53TG3B	TP53 target 3B
1.05	TP53TG3B; TP53TG3C; TP53TG3; LOC102723655; RP11-23E10.6	Homo sapiens TP53 target 3B (TP53TG3B). transcript variant 1. mRNA.; Homo sapiens TP53 target 3C (TP53TG3C). transcript variant 1. mRNA.; Homo sapiens TP53 target 3 (TP53TG3). transcript variant 1. mRNA.; Homo sapiens TP53 target 3 (TP53TG3). transcript variant 3. non-coding RNA.; Homo sapiens TP53 target 3B (TP53TG3B). transcript variant 3. non-coding RNA.; Homo sapiens TP53 target 3 (TP53TG3). transcript variant 4. non-coding RNA.; Homo sapiens TP53 target 3C (TP53TG3C). transcript variant 3. non-coding RNA.; TP53-target gene 3 protein; Homo sapiens TP53 target 3. mRNA (cDNA clone MGC:119889 IMAGE:40015196). complete cds.; Homo sapiens TP53 target 3. mRNA (cDNA clone MGC:119888 IMAGE:40015195). complete cds.; TP53-target gene 3 protein [Source:UniProtKB/Swiss-Prot;Acc:Q9ULZ0]
1.05	TP53TG3B; TP53TG3C; TP53TG3; LOC102723655; hotema; RP11-1277H1.5	Homo sapiens TP53 target 3B (TP53TG3B). transcript variant 1. mRNA.; Homo sapiens TP53 target 3C (TP53TG3C). transcript variant 1. mRNA.; Homo sapiens TP53 target 3 (TP53TG3). transcript variant 1. mRNA.; Homo sapiens TP53 target 3 (TP53TG3). transcript variant 3. non-coding RNA.; Homo sapiens TP53 target 3B (TP53TG3B). transcript variant 3. non-coding RNA.; Homo sapiens TP53 target 3 (TP53TG3). transcript variant 4. non-coding RNA.; Homo sapiens TP53 target 3C (TP53TG3C). transcript variant 3. non-coding RNA.; TP53-target gene 3 protein; Homo sapiens TP53 target 3. mRNA (cDNA clone MGC:119889 IMAGE:40015196). complete cds.; Homo sapiens TP53 target 3. mRNA (cDNA clone MGC:119888 IMAGE:40015195). complete cds.; Transcript Identified by AceView; TP53-target gene 3 protein [Source:UniProtKB/Swiss-Prot;Acc:Q9ULZ0]
1.05	BCL11A	B-cell CLL/lymphoma 11A (zinc finger protein)
1.05	ASB16	ankyrin repeat and SOCS box containing 16
1.05	PFN3	profilin 3
1.05	RAD54B	Transcript Identified by AceView. Entrez Gene ID(s) 25788
1.05	BMP2K	BMP2 inducible kinase
1.05	TMPRSS11E	transmembrane protease. serine 11E
1.05	GLDN	gliomedin

1.04	SNRNP27	small nuclear ribonucleoprotein. U4/U6.U5 27kDa subunit
1.04	RSRP1	arginine/serine-rich protein 1
1.04	RBFOX1	RNA binding protein. fox-1 homolog (C. elegans) 1
1.04	CHRNA1	cholinergic receptor. nicotinic alpha 1
1.04	CNOT10	CCR4-NOT transcription complex subunit 10
1.04	TNIK	TRAF2 and NCK interacting kinase
1.04	OR4F17	olfactory receptor. family 4. subfamily F. member 17
1.04	RRS1	ribosome biogenesis regulator homolog
1.04	OR10T2	olfactory receptor. family 10. subfamily T. member 2
1.04	C8orf44	chromosome 8 open reading frame 44
1.04	TRAPPC2	trafficking protein particle complex 2
1.04	GALNTL6	polypeptide N-acetylgalactosaminyltransferase-like 6
1.04	RASSF9	Ras association (RalGDS/AF-6) domain family (N-terminal) member 9
1.04	ZNF251	zinc finger protein 251
1.04	NAPB	N-ethylmaleimide-sensitive factor attachment protein. beta
1.04	RPL23A; SNORD4B; SNORD42B; SNORD42A	ribosomal protein L23a; small nucleolar RNA. C/D box 4B; small nucleolar RNA. C/D box 42B; small nucleolar RNA. C/D box 42A
1.04	TRABD	TraB domain containing
1.04	TNFSF11	tumor necrosis factor (ligand) superfamily. member 11
1.04	SLCO1A2	solute carrier organic anion transporter family. member 1A2
1.04	DESI2	desumoylating isopeptidase 2
1.04	ZNF501	zinc finger protein 501
1.04	RAPGEF2	Rap guanine nucleotide exchange factor 2
1.04	SLC47A1	solute carrier family 47 (multidrug and toxin extrusion). member 1
1.04	TAF1	TAF1 RNA polymerase II. TATA box binding protein (TBP)-associated factor. 250kDa
1.04	RHOU	ras homolog family member U

1.04	CCDC39	coiled-coil domain containing 39
1.04	NR1D2	nuclear receptor subfamily 1. group D. member 2
1.04	VSX1	visual system homeobox 1
1.04	HSFX2; HSFX1	heat shock transcription factor family. X-linked 2; heat shock transcription factor family. X-linked 1
1.04	WDR89	WD repeat domain 89
1.04	MTCL1	microtubule crosslinking factor 1
1.04	LINC00552	long intergenic non-protein coding RNA 552
1.04	DNAJC7	Zhang2013 ALT_ACCEPTOR. ALT_DONOR. coding. INTERNAL. intronic. OVERLAPTX. OVEXON best transcript NM_003315
1.04	POGZ	Transcript Identified by AceView. Entrez Gene ID(s) 23126
1.04	PLIN3	perilipin 3
1.04	KIF1B	kinesin family member 1B
1.04	OLIG2	oligodendrocyte lineage transcription factor 2
1.04	KRT24	keratin 24. type I
1.04	RRP36	ribosomal RNA processing 36
1.04	DIS3	DIS3 homolog. exosome endoribonuclease and 3-5 exoribonuclease
1.04	ERBB4	erb-b2 receptor tyrosine kinase 4
1.04	sept-03	septin 3
1.04	RP11-10L7.1; HERC6	Transcript Identified by AceView. Entrez Gene ID(s) 55008; novel transcript
1.04	INTS1	Memczak2013 ANTISENSE. CDS. coding. INTERNAL best transcript NM_001080453
1.04	FCGR2C	Fc fragment of IgG. low affinity IIc. receptor for (CD32) (gene/pseudogene)
1.04	BVES-AS1	BVES antisense RNA 1
1.04	TNNT2	troponin T type 2 (cardiac)
1.04	IZUMO1	izumo sperm-egg fusion 1
1.04	HCP5	HLA complex P5 (non-protein coding)

1.04	DEFB128	defensin. beta 128
1.04	LVRN	laeverin
1.04	MYH11	myosin. heavy chain 11. smooth muscle
1.04	USP17L27; USP17L28	ubiquitin specific peptidase 17-like family member 27; ubiquitin specific peptidase 17-like family member 28
1.04	USP17L28; USP17L30	ubiquitin specific peptidase 17-like family member 28; ubiquitin specific peptidase 17-like family member 30
1.04	USP17L29; USP17L5	ubiquitin specific peptidase 17-like family member 29; ubiquitin specific peptidase 17-like family member 5
1.04	USP17L25; USP17L30	ubiquitin specific peptidase 17-like family member 25; ubiquitin specific peptidase 17-like family member 30
1.04	HEMGN	hemogen
1.04	MICU1	mitochondrial calcium uptake 1
1.04	CHRNA6	cholinergic receptor. nicotinic alpha 6
1.04	CEP152	centrosomal protein 152kDa
1.04	NPVF	neuropeptide VF precursor
1.04	IK; MIR3655	IK cytokine. down-regulator of HLA II; microRNA 3655
1.04	IP6K3	inositol hexakisphosphate kinase 3
1.04	CAPN5	calpain 5
1.04	SCN4B	sodium channel. voltage gated. type IV beta subunit
1.04	GOLGA6D	golgin A6 family. member D
1.04	SMIM14	small integral membrane protein 14
1.04	ZNF510	zinc finger protein 510
1.04	TEX9	testis expressed 9
1.04	RLTPR	RGD motif. leucine rich repeats. tropomodulin domain and proline-rich containing
1.04	FDX1	ferredoxin 1
1.04	SETD5	SET domain containing 5

1.04	ZNF250	zinc finger protein 250
1.04	GRK4	G protein-coupled receptor kinase 4
1.04	CTDSP2	CTD small phosphatase 2
1.04	FMN1	formin 1
1.04	PRKAR1B	protein kinase. cAMP-dependent. regulatory. type I. beta
1.04	OR6Y1	olfactory receptor. family 6. subfamily Y. member 1
1.04	MTCH2	mitochondrial carrier 2
1.04	MMP16	matrix metallopeptidase 16 (membrane-inserted)
1.04	EIF3E	eukaryotic translation initiation factor 3. subunit E
1.04	STK32C	serine/threonine kinase 32C
1.04	KRTAP19-7	keratin associated protein 19-7
1.04	PLCXD3	phosphatidylinositol-specific phospholipase C. X domain containing 3
1.04	CDX1	caudal type homeobox 1
1.04	ADAMTS8	ADAM metallopeptidase with thrombospondin type 1 motif 8
1.04	SLC22A23	solute carrier family 22. member 23
1.04	RPS25	ribosomal protein S25
1.04	UBE2D3	ubiquitin conjugating enzyme E2D 3
1.04	GJB2	gap junction protein beta 2
1.04	CRYBA2	crystallin beta A2
1.04	LRCH3	leucine-rich repeats and calponin homology (CH) domain containing 3
1.04	STX18	syntaxin 18
1.04	RPS19	ribosomal protein S19
1.04	BIRC6	baculoviral IAP repeat containing 6
1.04	CACNA1B	calcium channel. voltage-dependent. N type. alpha 1B subunit
1.04	CUL4B	cullin 4B
1.04	PFDN6; MIR6834	prefoldin subunit 6; microRNA 6834

1.04	KRTAP9-7	keratin associated protein 9-7
1.04	ANKFN1	ankyrin-repeat and fibronectin type III domain containing 1
1.04	IDO2	indoleamine 2.3-dioxygenase 2
1.04	CNKSR1	connector enhancer of kinase suppressor of Ras 1
1.04	BIRC8	baculoviral IAP repeat containing 8
1.04	OR11H12	olfactory receptor. family 11. subfamily H. member 12
1.04	LRP5	LDL receptor related protein 5
1.04	IFNA10	interferon. alpha 10
1.04	HK3	hexokinase 3 (white cell)
1.04	SEMA6D	sema domain. transmembrane domain (TM). and cytoplasmic domain. (semaphorin) 6D
1.04	PRNT	prion protein (testis specific)
1.04	DLD	dihydrolipoamide dehydrogenase
1.04	USH1G	Usher syndrome 1G (autosomal recessive)
1.04	PLCE1	phospholipase C. epsilon 1
1.04	ZNF99	zinc finger protein 99
1.04	ZNF595	zinc finger protein 595
1.04	LINC01205	long intergenic non-protein coding RNA 1205
1.04	ATP1B2	ATPase. Na+/K+ transporting. beta 2 polypeptide
1.04	ZNF280C	zinc finger protein 280C
1.04	ZBED2	zinc finger. BED-type containing 2
1.04	RPF1	ribosome production factor 1 homolog
1.04	COX7B2	cytochrome c oxidase subunit VIIb2
1.04	NUP54	nucleoporin 54kDa
1.04	MYH4	myosin. heavy chain 4. skeletal muscle
1.04	EXOSC2	exosome component 2

1.04	LHFPL2	lipoma HMGIC fusion partner-like 2
1.04	CYS1	cystin 1
1.04	SLC6A13	solute carrier family 6 (neurotransmitter transporter). member 13
1.04	NLRC3	NLR family. CARD domain containing 3
1.04	ENPP5	ectonucleotide pyrophosphatase/phosphodiesterase 5 (putative)
1.04	RNF225	ring finger protein 225
1.04	RSAD1	radical S-adenosyl methionine domain containing 1
1.04	PHOX2B	paired-like homeobox 2b
1.04	MRPL32	mitochondrial ribosomal protein L32
1.04	RASSF8	Ras association (RalGDS/AF-6) domain family (N-terminal) member 8
1.04	NCK2	NCK adaptor protein 2
1.04	CCL19	chemokine (C-C motif) ligand 19
1.04	PLA2G15	phospholipase A2. group XV
1.04	ZNF134	zinc finger protein 134
1.04	TWIST1	twist family bHLH transcription factor 1
1.04	HEATR5B	HEAT repeat containing 5B
1.04	TNFAIP8L3	tumor necrosis factor. alpha-induced protein 8-like 3
1.04	C9orf57	chromosome 9 open reading frame 57
1.04	SCML1	sex comb on midleg-like 1 (Drosophila)
1.04	IPO13	importin 13
1.04	BCORL1	BCL6 corepressor-like 1
1.04	ZNF275	zinc finger protein 275
1.04	RPS15	ribosomal protein S15
1.04	ZNF827	zinc finger protein 827
1.04	NFE2L3	nuclear factor. erythroid 2-like 3
1.04	ZCCHC10	zinc finger. CCHC domain containing 10

1.04	TTC8	tetratricopeptide repeat domain 8
1.04	OR10H5	olfactory receptor. family 10. subfamily H. member 5
1.04	YIPF1	Yip1 domain family member 1
1.04	LGALS7B	lectin. galactoside-binding. soluble. 7B
1.04	NT5DC1	5-nucleotidase domain containing 1
1.04	BRMS1L	breast cancer metastasis-suppressor 1-like
1.04	PYHIN1	pyrin and HIN domain family. member 1
1.04	SIRT3	sirtuin 3
1.04	AQP1	aquaporin 1 (Colton blood group)
1.04	NPAT	nuclear protein. ataxia-telangiectasia locus
1.04	OR1E1	olfactory receptor. family 1. subfamily E. member 1
1.04	ADRA1B	adrenoceptor alpha 1B
1.04	EPHA6	EPH receptor A6
1.04	MRPL40	mitochondrial ribosomal protein L40
1.04	UXS1	UDP-glucuronate decarboxylase 1
1.04	LY6E	lymphocyte antigen 6 complex. locus E
1.04	C17orf67	chromosome 17 open reading frame 67
1.04	CERS2	ceramide synthase 2
1.04	TMEM42	transmembrane protein 42
1.04	FRAT1	frequently rearranged in advanced T-cell lymphomas 1
1.04	NSMF; MIR7114	NMDA receptor synaptonuclear signaling and neuronal migration factor; microRNA 7114
1.04	C10orf54	chromosome 10 open reading frame 54
1.04	TM9SF1	transmembrane 9 superfamily member 1
1.04	CAPZA3	capping protein (actin filament) muscle Z-line. alpha 3
1.04	RNPS1	RNA binding protein S1. serine-rich domain

1.04	EIF3F	Eukaryotic translation initiation factor 3 subunit F [Source:UniProtKB/Swiss-Prot;Acc:O00303]
1.04	URAD	ureidoimidazoline (2-oxo-4-hydroxy-4-carboxy-5-) decarboxylase
1.04	DUS2	dihydrouridine synthase 2
1.04	SUPT6H	SPT6 homolog. histone chaperone
1.04	LARS2	leucyl-tRNA synthetase 2. mitochondrial
1.04	ZDHHC15	zinc finger. DHHC-type containing 15
1.04	TMEM251	transmembrane protein 251
1.04	FCER2	Fc fragment of IgE. low affinity II. receptor for (CD23)
1.04	ARMCX4	armadillo repeat containing. X-linked 4
1.04	TMEM9	transmembrane protein 9
1.04	OBFC1	oligonucleotide/oligosaccharide-binding fold containing 1
1.04	UBE2N	ubiquitin conjugating enzyme E2N
1.04	ATP6V1C1	ATPase. H+ transporting. lysosomal 42kDa. V1 subunit C1
1.04	RCVRN	recoverin
1.04	DAB1	Dab. reelin signal transducer. homolog 1 (Drosophila)
1.04	KRTAP4-12	keratin associated protein 4-12
1.04	PAPD4	PAP associated domain containing 4
1.04	H3F3AP4; H3F3A; H3F3B	Homo sapiens H3 histone. family 3A. pseudogene 4 (H3F3AP4). non-coding RNA.; Homo sapiens H3 histone. family 3A. mRNA (cDNA clone MGC:47761 IMAGE:6014954). complete cds.; Homo sapiens H3 histone. family 3B (H3.3B). mRNA (cDNA clone MGC:87782 IMAGE:3884607). complete cds.; Homo sapiens H3 histone. family 3A. mRNA (cDNA clone MGC:87783 IMAGE:5162817). complete cds.
1.04	SBNO2	strawberry notch homolog 2 (Drosophila)
1.04	GCN1; MIR4498	GCN1 eIF2 alpha kinase activator homolog; microRNA 4498
1.04	SFTA2	surfactant associated 2
1.04	OR4C46	olfactory receptor. family 4. subfamily C. member 46

1.04	ZNF385D	zinc finger protein 385D
1.04	OR56B1	olfactory receptor. family 56. subfamily B. member 1
1.04	CLNK	cytokine-dependent hematopoietic cell linker
1.04	FAM160A2	family with sequence similarity 160. member A2
1.04	RWDD2B	RWD domain containing 2B
1.04	CTAG1B	cancer/testis antigen 1B
1.04	PHLDA3	pleckstrin homology-like domain. family A. member 3
1.04	CAPRIN2	caprin family member 2
1.04	NTMT1	N-terminal Xaa-Pro-Lys N-methyltransferase 1
1.04	IFT43	intraflagellar transport 43
1.04	MKX	mohawk homeobox
1.04	CBFA2T3	core-binding factor. runt domain. alpha subunit 2; translocated to. 3
1.04	HDGF	hepatoma-derived growth factor
1.04	CECR5	cat eye syndrome chromosome region. candidate 5
1.04	C7orf77	chromosome 7 open reading frame 77
1.04	SNRPG	small nuclear ribonucleoprotein polypeptide G
1.04	ADPGK	ADP-dependent glucokinase
1.04	RPL27A; SNORA3A; SNORA3B	ribosomal protein L27a; small nucleolar RNA. H/ACA box 3A; small nucleolar RNA. H/ACA box 3B
1.04	VPS16	vacuolar protein sorting 16 homolog (S. cerevisiae)
1.04	TMEM30B	transmembrane protein 30B
1.04	ENPP6	ectonucleotide pyrophosphatase/phosphodiesterase 6
1.04	ZNF132	zinc finger protein 132
1.04	VAX1	ventral anterior homeobox 1
1.04	GABRA4	gamma-aminobutyric acid (GABA) A receptor. alpha 4
1.04	NLRP8	NLR family. pyrin domain containing 8

1.04	TAF9B	TAF9B RNA polymerase II. TATA box binding protein (TBP)-associated factor. 31kDa
1.04	C20orf144	chromosome 20 open reading frame 144
1.04	POLB	polymerase (DNA directed). beta
1.04	PPBP	pro-platelet basic protein
1.04	CCDC184	coiled-coil domain containing 184
1.04	RERGL	RERG/RAS-like
1.04	KRTAP10-4	keratin associated protein 10-4
1.04	ZNF497; A1BG	zinc finger protein 497; alpha-1-B glycoprotein
1.04	TET2	tet methylcytosine dioxygenase 2
1.04	GAPDH	glyceraldehyde-3-phosphate dehydrogenase
1.04	IRG1	immunoresponsive 1 homolog (mouse)
1.04	TREML2	triggering receptor expressed on myeloid cells-like 2
1.04	BTBD10	BTB (POZ) domain containing 10
1.04	NUDT3	nudix hydrolase 3
1.04	LINGO3	leucine rich repeat and Ig domain containing 3
1.04	PRDM15	PR domain containing 15
1.04	OTOR	otoraplin
1.04	CDK5R1	cyclin-dependent kinase 5. regulatory subunit 1 (p35)
1.04	DEFB4B	defensin. beta 4B
1.04	TBX18	T-box 18
1.04	JADE2	jade family PHD finger 2
1.04	LTF	lactotransferrin
1.04	NPHP1	nephronophthisis 1 (juvenile)
1.04	RELL1	RELT-like 1
1.04	CYP2A13	cytochrome P450. family 2. subfamily A. polypeptide 13

1.04	THRAP3	thyroid hormone receptor associated protein 3
1.04	KIF15	kinesin family member 15
1.04	TSR1	TSR1. 20S rRNA accumulation. homolog (S. cerevisiae)
1.04	RHCG	Rh family. C glycoprotein
1.04	NPIPB6	nuclear pore complex interacting protein family. member B6
1.04	TREM1	triggering receptor expressed on myeloid cells 1
1.04	TMEM78	transmembrane protein 78
1.04	COG5	component of oligomeric golgi complex 5
1.04	DOLK	dolichol kinase
1.04	OIT3	oncoprotein induced transcript 3
1.04	MTNR1A	melatonin receptor 1A
1.04	CEP68	centrosomal protein 68kDa
1.04	C1orf100	chromosome 1 open reading frame 100
1.04	FUT2	fucosyltransferase 2 (secretor status included)
1.04	C20orf196	Transcript Identified by AceView. Entrez Gene ID(s) 149840
1.04	CLIC6	chloride intracellular channel 6
1.04	DEFB130	defensin. beta 130
1.04	DEFB130	defensin. beta 130
1.04	SIGLEC1	sialic acid binding Ig-like lectin 1. sialoadhesin
1.04	CCR9	chemokine (C-C motif) receptor 9
1.04	LRIG1	
1.04	KIF13B	kinesin family member 13B
1.04	ABHD3	Transcript Identified by AceView. Entrez Gene ID(s) 171586
1.04	MAP4K4	mitogen-activated protein kinase kinase kinase kinase 4
1.04	PF4V1	platelet factor 4 variant 1
1.04	LAIR2	leukocyte-associated immunoglobulin-like receptor 2

1.04	TGFBR1	transforming growth factor. beta receptor 1
1.04	SHISA5	shisa family member 5
1.04	DPPA4	developmental pluripotency associated 4
1.04	MCF2	MCF.2 cell line derived transforming sequence
1.04	CYP2C9	cytochrome P450. family 2. subfamily C. polypeptide 9
1.04	PSMC1	Homo sapiens proteasome (prosome. macropain) 26S subunit. ATPase. 1. mRNA (cDNA clone MGC:8541 IMAGE:2822718). complete cds.; Homo sapiens proteasome (prosome. macropain) 26S subunit. ATPase. 1. mRNA (cDNA clone MGC:24583 IMAGE:4133348). complete cds.; Homo sapiens proteasome (prosome. macropain) 26S subunit. ATPase. 1. mRNA (cDNA clone MGC:86994 IMAGE:5264945). complete cds.; Homo sapiens proteasome (prosome. macropain) 26S subunit. ATPase. 1. mRNA (cDNA clone MGC:88853 IMAGE:5456334). complete cds.
1.04	CELSR2	cadherin. EGF LAG seven-pass G-type receptor 2
1.04	CDC42BPG	CDC42 binding protein kinase gamma (DMPK-like)
1.04	PRICKLE3	prickle homolog 3
1.04	C9orf47; S1PR3	chromosome 9 open reading frame 47; sphingosine-1-phosphate receptor 3
1.04	CHRNA7	cholinergic receptor. nicotinic alpha 7
1.04	ANKRD2	ankyrin repeat domain 2 (stretch responsive muscle)
1.04	GPR142	G protein-coupled receptor 142
1.04	SLC26A9	solute carrier family 26 (anion exchanger). member 9
1.04	HAP1	huntingtin-associated protein 1
1.04	LARP6	La ribonucleoprotein domain family. member 6
1.04	RPS15A	ribosomal protein S15a
1.04	KRT38	keratin 38. type I
1.04	MC1R	melanocortin 1 receptor (alpha melanocyte stimulating hormone receptor)
1.04	OR8B4	olfactory receptor. family 8. subfamily B. member 4 (gene/pseudogene)
1.04	PPP1R12A	protein phosphatase 1. regulatory subunit 12A

1.04	HCN2	hyperpolarization activated cyclic nucleotide gated potassium channel 2
1.04	IKZF1	IKAROS family zinc finger 1
1.04	SLC9A3R2	solute carrier family 9. subfamily A (NHE3. cation proton antiporter 3). member 3 regulator 2
1.04	KAT6A	K(lysine) acetyltransferase 6A
1.04	HAPLN1	hyaluronan and proteoglycan link protein 1
1.04	GH2	growth hormone 2
1.04	CDKL2	cyclin-dependent kinase-like 2 (CDC2-related kinase)
1.04	SELT; SELT.1	selenoprotein T; SELT protein; Selenoprotein T; cDNA FLJ90525 fis. clone NT2RP4001001. highly similar to Selenoprotein T [Source:UniProtKB/TrEMBL;Acc:Q6IAK0]; Transcript Identified by AceView. Entrez Gene ID(s) 51714
1.04	PAWR	PRKC. apoptosis. WT1. regulator
1.04	ITIH5	inter-alpha-trypsin inhibitor heavy chain family. member 5
1.04	LY6E	lymphocyte antigen 6 complex. locus E
1.04	HDAC4	histone deacetylase 4
1.04	ALDH3A2	aldehyde dehydrogenase 3 family. member A2
1.04	ATP8A2	ATPase. aminophospholipid transporter. class I. type 8A. member 2
1.04	LAT2	linker for activation of T-cells family member 2
1.04	CTAGE8	CTAGE family. member 8
1.04	ZC3H18	zinc finger CCCH-type containing 18
1.04	MS4A13	membrane-spanning 4-domains. subfamily A. member 13
1.04	ADGRL1	adhesion G protein-coupled receptor L1
1.04	WASF2	Memczak2013 ANTISENSE. coding. INTERNAL. intronic best transcript NM_006990
1.04	EXOC4	exocyst complex component 4
1.04	PTGES3L-AARSD1; PTGES3L; AARSD1	PTGES3L-AARSD1 readthrough; prostaglandin E synthase 3 (cytosolic)-like; alanyl-tRNA synthetase domain containing 1

1.04	NDUFAF6	NADH dehydrogenase (ubiquinone) complex I. assembly factor 6
1.04	MORF4L1	mortality factor 4 like 1
1.04	ADCY10	adenylate cyclase 10 (soluble)
1.04	LRFN1	leucine rich repeat and fibronectin type III domain containing 1
1.04	ZNF677	zinc finger protein 677
1.04	COX6C	cytochrome c oxidase subunit VIc
1.04	PNMA5	paraneoplastic Ma antigen family member 5
1.04	DUPD1	dual specificity phosphatase and pro isomerase domain containing 1
1.04	ANXA1	annexin A1
1.04	BAALC	brain and acute leukemia. cytoplasmic
1.04	PRMT7	protein arginine methyltransferase 7
1.04	IPMK	inositol polyphosphate multikinase
1.04	ACVRL1	activin A receptor type IL
1.04	PLS1	plastin 1
1.04	MYH9	Zhang2013 ALT_ACCEPTOR. ALT_DONOR. coding. INTERNAL. intronic best transcript NM_002473
1.04	LARP1B	La ribonucleoprotein domain family. member 1B
1.04	B3GNT7	UDP-GlcNAc:betaGal beta-1.3-N-acetylglucosaminyltransferase 7
1.04	TSPAN16	tetraspanin 16
1.04	DYRK1B	dual specificity tyrosine-(Y)-phosphorylation regulated kinase 1B
1.04	LIPT2	lipoyl(octanoyl) transferase 2 (putative)
1.04	OR4S2	olfactory receptor. family 4. subfamily S. member 2
1.04	OR4K17	olfactory receptor. family 4. subfamily K. member 17
1.04	THEMIS	thymocyte selection associated
1.04	ELMOD1	ELMO/CED-12 domain containing 1
1.04	FAAP20	Fanconi anemia core complex associated protein 20

1.04	STARD5	StAR-related lipid transfer domain containing 5
1.04	TCTEX1D4	Tctex1 domain containing 4
1.04	BCL7C	B-cell CLL/lymphoma 7C
1.04	CNOT4	CCR4-NOT transcription complex subunit 4
1.04	TRMT44	tRNA methyltransferase 44 homolog (S. cerevisiae)
1.04	TRMT2A	tRNA methyltransferase 2 homolog A
1.04	WDR55	WD repeat domain 55
1.04	ADCY8	adenylate cyclase 8 (brain)
1.04	NXPH3	neurexophilin 3
1.04	ENAM	enamelin
1.04	KCP	kielin/chordin-like protein
1.04	ALDH3B2	aldehyde dehydrogenase 3 family. member B2
1.04	ERO1B	endoplasmic reticulum oxidoreductase beta
1.04	PPP4R2	protein phosphatase 4. regulatory subunit 2
1.04	LCE3E	late cornified envelope 3E
1.04	TARM1	T cell-interacting. activating receptor on myeloid cells 1
1.04	DNAJC24	DnaJ (Hsp40) homolog. subfamily C. member 24
1.04	SLC16A1	solute carrier family 16 (monocarboxylate transporter). member 1
1.04	ATXN2L	ataxin 2-like
1.04	CR1L	complement component (3b/4b) receptor 1-like
1.04	CCIN	calicin
1.04	ZPLD1	zona pellucida-like domain containing 1
1.04	RGPD8; RGPD6	RANBP2-like and GRIP domain containing 8; RANBP2-like and GRIP domain containing 6
1.04	SPANXN1	SPANX family. member N1
1.04	STAM	Memczak2013 ALT_ACCEPTOR. ALT_DONOR. INTERNAL. intronic. ncRNA best transcript NR_037774

1.04	FOXF1	forkhead box F1
1.04	ANKRD42	ankyrin repeat domain 42
1.04	C1orf54	chromosome 1 open reading frame 54
1.04	KRTAP9-1	keratin associated protein 9-1
1.04	TRIT1	tRNA isopentenyltransferase 1
1.04	OPRM1	opioid receptor. mu 1
1.04	AHSA1	AHA1. activator of heat shock 90kDa protein ATPase homolog 1 (yeast)
1.04	TNKS	tankyrase. TRF1-interacting ankyrin-related ADP-ribose polymerase
1.04	SLC25A13	solute carrier family 25 (aspartate/glutamate carrier). member 13
1.04	CIB2	calcium and integrin binding family member 2
1.04	MLANA	melan-A
1.04	AGAP9; BMS1P6	ArfGAP with GTPase domain. ankyrin repeat and PH domain 9; BMS1 ribosome biogenesis factor pseudogene 6
1.04	HIST1H4C	Jeck2013 ANTISENSE. CDS. coding. INTERNAL. OVCODE. OVEXON. UTR3 best transcript NM_003542
1.04	CCDC137	coiled-coil domain containing 137
1.04	NXPE1	neurexophilin and PC-esterase domain family. member 1
1.04	PPP1R3G	protein phosphatase 1. regulatory subunit 3G
1.04	ZNF385A	Memczak2013 ANTISENSE. CDS. coding. INTERNAL best transcript NM_001130967
1.04	MT4	metallothionein 4
1.04	MEI1	meiotic double-stranded break formation protein 1
1.04	ZNF645	zinc finger protein 645
1.04	CCDC102A	coiled-coil domain containing 102A
1.04	FAM134C	family with sequence similarity 134. member C
1.04	LOC389199; AC097381.1	uncharacterized LOC389199; Transcript Identified by AceView. Entrez Gene ID(s) 389199; novel transcript

1.04	CPA5	carboxypeptidase A5
1.04	GNG7	guanine nucleotide binding protein (G protein). gamma 7
1.04	CLDN24	claudin 24
1.04	ZNF451	zinc finger protein 451
1.04	PSMD12	proteasome 26S subunit. non-ATPase 12
1.04	CCSAP	centriole. cilia and spindle-associated protein
1.04	GLOD5	glyoxalase domain containing 5
1.04	HSF2	heat shock transcription factor 2
1.04	RBKS; BRE-AS1	ribokinase; BRE antisense RNA 1
1.04	MYDGF	myeloid-derived growth factor
1.04	RGS7BP	regulator of G-protein signaling 7 binding protein
1.04	ZNF517	zinc finger protein 517
1.04	GCOM1; MYZAP; POLR2M	GRINL1A complex locus 1; myocardial zonula adherens protein; polymerase (RNA) II (DNA directed) polypeptide M
1.04	CAMK4	calcium/calmodulin-dependent protein kinase IV
1.04	CXorf36	chromosome X open reading frame 36
1.04	PRPS2	phosphoribosyl pyrophosphate synthetase 2
1.04	PSG11	pregnancy specific beta-1-glycoprotein 11
1.04	RP11-998D10.7; FLJ10357	Transcript Identified by AceView. Entrez Gene ID(s) 55701
1.04	CTPS2	CTP synthase 2
1.04	RDH8	retinol dehydrogenase 8 (all-trans)
1.04	AARD	alanine and arginine rich domain containing protein
1.04	PQBP1	polyglutamine binding protein 1
1.04	MS4A4A	membrane-spanning 4-domains. subfamily A. member 4A
1.04	CTD-3032H12.2; IRX5	Transcript Identified by AceView. Entrez Gene ID(s) 10265; novel transcript
1.04	VPS11	VPS11. CORVET/HOPS core subunit [Source:HGNC Symbol;Acc:HGNC:14583]

1.04	TREML4	triggering receptor expressed on myeloid cells-like 4
1.04	ZNF585A	zinc finger protein 585A
1.04	DACT1	dishevelled-binding antagonist of beta-catenin 1
1.04	SSPN	sarcospan
1.04	RASSF6	Ras association (RalGDS/AF-6) domain family member 6
1.04	FCHSD1	FCH and double SH3 domains 1
1.04	ZSCAN5B	zinc finger and SCAN domain containing 5B
1.04	H2BFWT	H2B histone family. member W. testis-specific
1.04	FLOT2	Memczak2013 ANTISENSE. coding. INTERNAL. UTR3 best transcript NM_004475
1.04	XPNPEP1	X-prolyl aminopeptidase (aminopeptidase P) 1. soluble
1.04	MORN5	MORN repeat containing 5
1.04	METTL13	methyltransferase like 13
1.04	ZCCHC18	zinc finger. CCHC domain containing 18
1.04	OR51T1	olfactory receptor. family 51. subfamily T. member 1
1.04	GML	glycosylphosphatidylinositol anchored molecule like
1.04	SUPT3H	SPT3 homolog. SAGA and STAGA complex component
1.04	ADM5; CPT1C	adrenomedullin 5 (putative); carnitine palmitoyltransferase 1C
1.04	MBOAT1	membrane bound O-acyltransferase domain containing 1
1.04	PCNX	pecanex homolog (Drosophila)
1.04	IGSF3	immunoglobulin superfamily. member 3
1.04	SPATA41	spermatogenesis associated 41 (non-protein coding)
1.03	OR4L1	olfactory receptor. family 4. subfamily L. member 1
1.03	SART1	squamous cell carcinoma antigen recognized by T-cells 1
1.03	IFT81	intraflagellar transport 81
1.03	RPL37	ribosomal protein L37

1.03	ADIRF; AGAP11; BMS1P3	adipogenesis regulatory factor; ankyrin repeat and GTPase domain Arf GTPase activating protein 11; BMS1 ribosome biogenesis factor pseudogene 3
1.03	DEFA4	defensin. alpha 4. corticostatin
1.03	KIDINS220	kinase D-interacting substrate 220kDa
1.03	C11orf49	chromosome 11 open reading frame 49
1.03	PID1	phosphotyrosine interaction domain containing 1
1.03	TMEM64	transmembrane protein 64
1.03	ZNF780A	zinc finger protein 780A
1.03	ONECUT3	one cut homeobox 3
1.03	PACRG	PARK2 co-regulated
1.03	NDC1	NDC1 transmembrane nucleoporin
1.03	CD151	CD151 molecule (Raph blood group)
1.03	KAT6B	K(lysine) acetyltransferase 6B
1.03	SPNS3	spinster homolog 3 (Drosophila)
1.03	STK26	serine/threonine protein kinase 26
1.03	RAB41	RAB41. member RAS oncogene family
1.03	APITD1-CORT; CORT; APITD1	APITD1-CORT readthrough; cortistatin; apoptosis-inducing. TAF9-like domain 1
1.03	BTBD6	BTB (POZ) domain containing 6
1.03	ITIH2	inter-alpha-trypsin inhibitor heavy chain 2
1.03	PRAMEF27	PRAME family member 27
1.03	TGFBI	transforming growth factor. beta-induced. 68kDa
1.03	SSX1	synovial sarcoma. X breakpoint 1
1.03	CCDC24	coiled-coil domain containing 24
1.03	CHST1	carbohydrate (keratan sulfate Gal-6) sulfotransferase 1
1.03	MOG	myelin oligodendrocyte glycoprotein

1.03	COQ9	coenzyme Q9
1.03	GPBP1	GC-rich promoter binding protein 1
1.03	HECA	hdc homolog. cell cycle regulator
1.03	NMB	neuromedin B
1.03	C14orf80	chromosome 14 open reading frame 80
1.03	PGAM2	phosphoglycerate mutase 2 (muscle)
1.03	ZNF226	zinc finger protein 226
1.03	PTGDR2	prostaglandin D2 receptor 2
1.03	BAX	BCL2-associated X protein
1.03	BARX2	BARX homeobox 2
1.03	SNRPC	small nuclear ribonucleoprotein polypeptide C
1.03	ZNF598	zinc finger protein 598
1.03	ZNF543	zinc finger protein 543
1.03	CD52	CD52 molecule
1.03	PBX4	pre-B-cell leukemia homeobox 4
1.03	HTR2B	5-hydroxytryptamine (serotonin) receptor 2B. G protein-coupled
1.03	TDRP	testis development related protein
1.03	RPS9	Jeck2013 ANTISENSE. CDS. coding. INTERNAL. OVCODE. OVEXON. UTR3 best transcript NM_001013
1.03	PPAT	phosphoribosyl pyrophosphate amidotransferase
1.03	PEX5	peroxisomal biogenesis factor 5
1.03	KIAA0753	KIAA0753
1.03	FBRSL1	fibrosin-like 1
1.03	CRISPLD2	cysteine-rich secretory protein LCCL domain containing 2
1.03	BLVRB	biliverdin reductase B
1.03	ANXA4	annexin A4

1.03	RSC1A1; DDI2	regulatory solute carrier protein. family 1. member 1; DNA-damage inducible 1 homolog 2
1.03	HYPK	huntingtin interacting protein K
1.03	HPR	haptoglobin-related protein
1.03	ZNF726; ZNF92P3	zinc finger protein 726; zinc finger protein 92 pseudogene 3
1.03	EIF3B	eukaryotic translation initiation factor 3. subunit B
1.03	KIAA0930	KIAA0930
1.03	PRTN3	proteinase 3
1.03	TSTA3	tissue specific transplantation antigen P35B
1.03	P2RY2	purinergic receptor P2Y. G-protein coupled. 2
1.03	TP53I13	tumor protein p53 inducible protein 13
1.03	RAB4A; SPHAR	RAB4A. member RAS oncogene family; S-phase response (cyclin related)
1.03	USP17L17	ubiquitin specific peptidase 17-like family member 17
1.03	HAUS7; TREX2	HAUS augmin like complex subunit 7; three prime repair exonuclease 2
1.03	FAM89B	family with sequence similarity 89. member B
1.03	BPY2C	basic charge. Y-linked. 2C
1.03	MROH2B	maestro heat-like repeat family member 2B
1.03	ZP2	zona pellucida glycoprotein 2 (sperm receptor)
1.03	SUGCT	succinyl-CoA:glutarate-CoA transferase
1.03	CYP1B1	cytochrome P450. family 1. subfamily B. polypeptide 1
1.03	UBA6	ubiquitin-like modifier activating enzyme 6
1.03	TMEM232	transmembrane protein 232
1.03	ECSIT	ECSIT signalling integrator
1.03	GOLGA8J; GOLGA8IP	golgin A8 family. member J; golgin A8 family. member I. pseudogene
1.03	STAU1	staufen double-stranded RNA binding protein 1
1.03	KCNH7	potassium channel. voltage gated eag related subfamily H. member 7

1.03	UQCRH	ubiquinol-cytochrome c reductase hinge protein
1.03	RTTN	rotatin
1.03	PDZD8	PDZ domain containing 8
1.03	NDUFA10	NADH dehydrogenase (ubiquinone) 1 alpha subcomplex. 10. 42kDa
1.03	KRTAP5-6	keratin associated protein 5-6
1.03	ARSJ	arylsulfatase family. member J
1.03	ACPP	acid phosphatase. prostate
1.03	MBL2	mannose-binding lectin (protein C) 2. soluble
1.03	UBE2Q2L	ubiquitin conjugating enzyme E2Q family member 2-like
1.03	SDHAF4	succinate dehydrogenase complex assembly factor 4
1.03	CDKL4	cyclin-dependent kinase-like 4
1.03	MSI2	musashi RNA binding protein 2
1.03	PSG9	pregnancy specific beta-1-glycoprotein 9
1.03	CNOT8	CCR4-NOT transcription complex subunit 8
1.03	ERG	v-ets avian erythroblastosis virus E26 oncogene homolog
1.03	ABHD1	abhydrolase domain containing 1
1.03	SLMAP	sarcolemma associated protein
1.03	CADPS	Ca++-dependent secretion activator
1.03	CPPED1	calcineurin-like phosphoesterase domain containing 1
1.03	EFEMP1	EGF containing fibulin-like extracellular matrix protein 1
1.03	ACSBG1	acyl-CoA synthetase bubblegum family member 1
1.03	GAK	cyclin G associated kinase
1.03	NDUFA4	NDUFA4. mitochondrial complex associated
1.03	CTU2	cytosolic thiouridylase subunit 2 homolog (S. pombe)
1.03	RFX6	regulatory factor X. 6
1.03	C16orf87	chromosome 16 open reading frame 87

1.03	UBE2Q2	ubiquitin-conjugating enzyme E2Q family member 2
1.03	SLIT2	slit guidance ligand 2
1.03	C17orf89	chromosome 17 open reading frame 89
1.03	OR6C76	olfactory receptor. family 6. subfamily C. member 76
1.03	C1orf195	chromosome 1 open reading frame 195
1.03	MBP	myelin basic protein
1.03	PSMC5	proteasome 26S subunit. ATPase 5
1.03	IL12B	interleukin 12B
1.03	VPRBP	Vpr (HIV-1) binding protein
1.03	ZNF91	zinc finger protein 91
1.03	NRIP2	nuclear receptor interacting protein 2
1.03	PLEKHA6	pleckstrin homology domain containing. family A member 6
1.03	TRMU	tRNA 5-methylaminomethyl-2-thiouridylate methyltransferase
1.03	C2orf40	chromosome 2 open reading frame 40
1.03	LDHA	lactate dehydrogenase A
1.03	TFPI	tissue factor pathway inhibitor (lipoprotein-associated coagulation inhibitor)
1.03	ARHGAP40	Rho GTPase activating protein 40
1.03	AIM1	absent in melanoma 1
1.03	OBP2B	odorant binding protein 2B
1.03	GRID2IP	glutamate receptor. ionotropic. delta 2 (Grid2) interacting protein
1.03	MBOAT2	membrane bound O-acyltransferase domain containing 2
1.03	EGFL6	EGF-like-domain. multiple 6
1.03	MAP10	microtubule-associated protein 10
1.03	SLC6A8	solute carrier family 6 (neurotransmitter transporter). member 8
1.03	LGI4	leucine-rich repeat LGI family. member 4
1.03	L3MBTL2	l(3)mbt-like 2 (Drosophila)

1.03	ACTRT3	actin-related protein T3
1.03	NCOA6	nuclear receptor coactivator 6
1.03	PTPMT1	protein tyrosine phosphatase. mitochondrial 1
1.03	RTN1	reticulon 1
1.03	PALD1	phosphatase domain containing. paladin 1
1.03	FAM71F2	family with sequence similarity 71. member F2
1.03	SMTNL1	smoothelin-like 1
1.03	ZNF350	zinc finger protein 350
1.03	INO80B	INO80 complex subunit B
1.03	RFXAP	regulatory factor X-associated protein
1.03	DHRS13	dehydrogenase/reductase (SDR family) member 13
1.03	FAM209A	family with sequence similarity 209. member A
1.03	CNTN5	contactin 5
1.03	ELSPBP1	epididymal sperm binding protein 1
1.03	TEX36	testis expressed 36
1.03	PPFIA4	protein tyrosine phosphatase. receptor type. f polypeptide (PTPRF). interacting protein (liprin). alpha 4
1.03	CCNA1	cyclin A1
1.03	ZNF770	zinc finger protein 770
1.03	OBSCN	obscurin. cytoskeletal calmodulin and titin-interacting RhoGEF
1.03	OR52N4	olfactory receptor. family 52. subfamily N. member 4 (gene/pseudogene)
1.03	ZNF286B; FOXO3B	zinc finger protein 286B; forkhead box O3B pseudogene
1.03	GPR176	G protein-coupled receptor 176
1.03	SNX20	sorting nexin 20
1.03	CT45A1	cancer/testis antigen family 45. member A1
1.03	RAB34	RAB34. member RAS oncogene family

1.03	FOXE3	forkhead box E3
1.03	PIDD1	p53-induced death domain protein 1
1.03	TIGD2	tigger transposable element derived 2
1.03	OR52K2	olfactory receptor. family 52. subfamily K. member 2
1.03	UTRN	utrophin
1.03	PRB2	proline-rich protein BstNI subfamily 2
1.03	TULP1	tubby like protein 1
1.03	LRRC53	leucine rich repeat containing 53
1.03	CSTL1	cystatin-like 1
1.03	SORCS1	sortilin-related VPS10 domain containing receptor 1
1.03	CKAP5	Transcript Identified by AceView. Entrez Gene ID(s) 9793
1.03	KLF17	Kruppel-like factor 17
1.03	SMIM17	small integral membrane protein 17
1.03	PSMD14	proteasome 26S subunit. non-ATPase 14
1.03	CACUL1	CDK2-associated. cullin domain 1
1.03	DNASE2B	deoxyribonuclease II beta
1.03	NEK11	NIMA-related kinase 11
1.03	NPIPA5	nuclear pore complex interacting protein family. member A5
1.03	ZIC3	Zic family member 3
1.03	RPS6KA1	ribosomal protein S6 kinase. 90kDa. polypeptide 1
1.03	NEK9	NIMA-related kinase 9
1.03	PWWP2B	PWWP domain containing 2B
1.03	MYOZ1	myozenin 1
1.03	PRPF4	pre-mRNA processing factor 4
1.03	KCNE5; ACSL4	potassium channel. voltage gated subfamily E regulatory beta subunit 5; acyl-CoA synthetase long-chain family member 4

1.03	CEP85	centrosomal protein 85kDa
1.03	FBL	fibrillarin
1.03	TRAF1	TNF receptor-associated factor 1
1.03	RB1	retinoblastoma 1
1.03	ZNF683	zinc finger protein 683
1.03	TAS2R8	taste receptor. type 2. member 8
1.03	TMEM71	transmembrane protein 71
1.03	WNT8B	wingless-type MMTV integration site family. member 8B
1.03	GCFC2	GC-rich sequence DNA-binding factor 2
1.03	AMIGO1	adhesion molecule with Ig-like domain 1
1.03	SOX3	SRY box 3
1.03	ZP4	zona pellucida glycoprotein 4
1.03	PLEKHA8	pleckstrin homology domain containing. family A (phosphoinositide binding specific) member 8
1.03	ATP5F1	ATP synthase. H+ transporting. mitochondrial Fo complex subunit B1
1.03	REN	renin
1.03	HNRNPDL	heterogeneous nuclear ribonucleoprotein D like
1.03	RNF219	ring finger protein 219
1.03	MS4A7	membrane-spanning 4-domains. subfamily A. member 7
1.03	WNT2	wingless-type MMTV integration site family member 2
1.03	PTPRR	protein tyrosine phosphatase. receptor type. R
1.03	ZNF816-ZNF321P	ZNF816-ZNF321P readthrough
1.03	OTUB2	OTU deubiquitinase. ubiquitin aldehyde binding 2
1.03	TFRC	Transcript Identified by AceView. Entrez Gene ID(s) 7037
1.03	SLC25A22	solute carrier family 25 (mitochondrial carrier: glutamate). member 22
1.03	TMPRSS9	transmembrane protease. serine 9

1.03	HDDC2	HD domain containing 2
1.03	PARP8	poly(ADP-ribose) polymerase family member 8
1.03	PCDHB10	protocadherin beta 10
1.03	TESK1; MIR4667	testis-specific kinase 1; microRNA 4667
1.03	RALB	v-ral simian leukemia viral oncogene homolog B
1.03	TCP1; SNORA20; SNORA29	t-complex 1; small nucleolar RNA. H/ACA box 20; small nucleolar RNA. H/ACA box 29
1.03	IFIT5	interferon-induced protein with tetratricopeptide repeats 5
1.03	NFYB	nuclear transcription factor Y subunit beta
1.03	KRT32	keratin 32. type I
1.03	RAB3GAP2; AURKAPS1	RAB3 GTPase activating protein subunit 2 (non-catalytic); aurora kinase A pseudogene 1
1.03	SDE2	SDE2 telomere maintenance homolog (S. pombe)
1.03	TDRD10	tudor domain containing 10
1.03	MROH5	maestro heat-like repeat family member 5
1.03	OTUD3	OTU deubiquitinase 3
1.03	TOMM40	translocase of outer mitochondrial membrane 40 homolog (yeast)
1.03	SYT7	synaptotagmin VII
1.03	CYP11A1	cytochrome P450. family 11. subfamily A. polypeptide 1
1.03	HNRNPH2	heterogeneous nuclear ribonucleoprotein H2 (H)
1.03	ELANE	elastase. neutrophil expressed
1.03	BANK1	B-cell scaffold protein with ankyrin repeats 1
1.03	RPP14	ribonuclease P/MRP 14kDa subunit
1.03	ZNF775	zinc finger protein 775
1.03	FSCN1	fascin actin-bundling protein 1
1.03	AGXT2	alanine--glyoxylate aminotransferase 2
1.03	WDFY4	WDFY family member 4

1.03	SPINK8	serine peptidase inhibitor. Kazal type 8 (putative)
1.03	SDC1	syndecan 1
1.03	CCDC57	coiled-coil domain containing 57
1.03	ADGB	androglobin
1.03	CR1	complement component (3b/4b) receptor 1 (Knops blood group)
1.03	TAF5L	TAF5-like RNA polymerase II. p300/CBP-associated factor (PCAF)-associated factor. 65kDa
1.03	ARL5B	ADP-ribosylation factor like GTPase 5B
1.03	CMYA5	cardiomyopathy associated 5
1.03	EFCAB8	EF-hand calcium binding domain 8
1.03	CALCRL	calcitonin receptor like receptor
1.03	SVEP1	sushi. von Willebrand factor type A. EGF and pentraxin domain containing 1
1.03	SHC3	SHC (Src homology 2 domain containing) transforming protein 3
1.03	ELK1	ELK1. member of ETS oncogene family
1.03	TSC1	Transcript Identified by AceView. Entrez Gene ID(s) 7248
1.03	PLEKHO2	pleckstrin homology domain containing. family O member 2
1.03	MRPL54	mitochondrial ribosomal protein L54
1.03	KCNC2	potassium channel. voltage gated Shaw related subfamily C. member 2
1.03	CCDC78	coiled-coil domain containing 78
1.03	ANKRD62	ankyrin repeat domain 62
1.03	DNAJB7	DnaJ (Hsp40) homolog. subfamily B. member 7
1.03	ALDH1L1-AS1	ALDH1L1 antisense RNA 1
1.03	TMCO5A	transmembrane and coiled-coil domains 5A
1.03	GRIA4	glutamate receptor. ionotropic. AMPA 4
1.03	MYOD1	myogenic differentiation 1
1.03	TCTN1	tectonic family member 1

1.03	FUT1	fucosyltransferase 1 (galactoside 2-alpha-L-fucosyltransferase. H blood group)
1.03	MFAP3L	microfibrillar associated protein 3 like
1.03	FAM46C	family with sequence similarity 46. member C
1.03	VN1R2	vomeronasal 1 receptor 2
1.03	URB2	URB2 ribosome biogenesis 2 homolog (S. cerevisiae)
1.03	HLA-DOA	major histocompatibility complex. class II. DO alpha
1.03	SDR16C5	short chain dehydrogenase/reductase family 16C. member 5
1.03	CD300LD	CD300 molecule-like family member d
1.03	ZNF84	zinc finger protein 84
1.03	SSX5	synovial sarcoma. X breakpoint 5
1.03	OR13H1	olfactory receptor. family 13. subfamily H. member 1
1.03	NETO1	neuropilin (NRP) and tolloid (TLL)-like 1
1.03	PRDM9	PR domain containing 9
1.03	NTRK3	neurotrophic tyrosine kinase. receptor. type 3
1.03	CHFR	checkpoint with forkhead and ring finger domains. E3 ubiquitin protein ligase
1.03	TM4SF20	transmembrane 4 L six family member 20
1.03	ANK1	ankyrin 1. erythrocytic
1.03	PAX6	paired box 6
1.03	C4orf17	chromosome 4 open reading frame 17
1.03	DCDC1	doublecortin domain containing 1
1.03	BLK	BLK proto-oncogene. Src family tyrosine kinase
1.03	NAP1L1	Memczak2013 ALT_ACCEPTOR. ALT_DONOR. coding. INTERNAL. intronic best transcript NM_139207
1.03	COL4A4	collagen. type IV. alpha 4
1.03	DDX41	DEAD (Asp-Glu-Ala-Asp) box polypeptide 41
1.03	C5orf47	chromosome 5 open reading frame 47

1.03	TOX2	TOX high mobility group box family member 2
1.03	ZNF599	zinc finger protein 599
1.03	LSM14A	LSM14A mRNA processing body assembly factor
1.03	NPIPB8	nuclear pore complex interacting protein family. member B8
1.03	KRTAP9-8	keratin associated protein 9-8
1.03	HLA-G	major histocompatibility complex. class I. G
1.03	GAPT	GRB2-binding adaptor protein. transmembrane
1.03	SEC14L5	SEC14-like lipid binding 5
1.03	ETV6	ets variant 6
1.03	RCE1; C11orf80	Ras converting CAAX endopeptidase 1; chromosome 11 open reading frame 80
1.03	C22orf23	chromosome 22 open reading frame 23
1.03	CCDC84	coiled-coil domain containing 84
1.03	GADD45B	growth arrest and DNA-damage-inducible. beta
1.03	C15orf52	chromosome 15 open reading frame 52
1.03	CAPNS1	calpain. small subunit 1
1.03	PCBP1	poly(rC) binding protein 1
1.03	TBX20	T-box 20
1.03	CITED4	Cbp/p300-interacting transactivator. with Glu/Asp rich carboxy-terminal domain. 4
1.03	CACFD1	calcium channel flower domain containing 1
1.03	CRYGS	crystallin gamma S
1.03	MGP	matrix Gla protein
1.03	IFI44	interferon-induced protein 44
1.03	AHCY	adenosylhomocysteinase
1.03	ZNF148	zinc finger protein 148
1.03	METTL23	methyltransferase like 23

1.03	HTR7	5-hydroxytryptamine (serotonin) receptor 7. adenylate cyclase-coupled
1.03	OPRL1	opiate receptor-like 1
1.03	OR8K5	olfactory receptor. family 8. subfamily K. member 5
1.03	USP34	ubiquitin specific peptidase 34
1.03	GP9	glycoprotein IX (platelet)
1.03	TRIM14	tripartite motif containing 14
1.03	CNTLN	centlein. centrosomal protein
1.03	C19orf57	chromosome 19 open reading frame 57
1.03	TRIM54	tripartite motif containing 54
1.03	FAM150A	family with sequence similarity 150. member A
1.03	LPIN3	lipin 3
1.03	RPL6	ribosomal protein L6
1.03	HLA-DPA1	major histocompatibility complex. class II. DP alpha 1
1.03	EEF1A1P5	eukaryotic translation elongation factor 1 alpha 1 pseudogene 5
1.03	EGLN3	egl-9 family hypoxia-inducible factor 3
1.03	NCL	nucleolin
1.03	LOC339862; RP11-320G10.1; skerdorbo	uncharacterized LOC339862; novel transcript; Transcript Identified by AceView
1.03	XDH	xanthine dehydrogenase
1.03	ITLN2	intelectin 2
1.03	ZNF26	zinc finger protein 26
1.03	EBNA1BP2; MIR6733	EBNA1 binding protein 2; microRNA 6733
1.03	ZNF396	zinc finger protein 396
1.03	PLAC4	placenta specific 4
1.03	STK38	serine/threonine kinase 38
1.03	ADH4	alcohol dehydrogenase 4 (class II). pi polypeptide

1.03	CDH6	cadherin 6. type 2. K-cadherin (fetal kidney)
1.03	IL24	interleukin 24
1.03	ZNF529	zinc finger protein 529
1.03	SH3RF3	SH3 domain containing ring finger 3
1.03	C1orf127	chromosome 1 open reading frame 127
1.03	PRLHR	prolactin releasing hormone receptor
1.03	ZNF605	zinc finger protein 605
1.03	RPL13A; SNORD35A; SNORD34; SNORD33; SNORD32A	ribosomal protein L13a; small nucleolar RNA. C/D box 35A; small nucleolar RNA. C/D box 34; small nucleolar RNA. C/D box 33; small nucleolar RNA. C/D box 32A
1.03	LOC200726; AC010731.4	hCG1657980; Transcript Identified by AceView. Entrez Gene ID(s) 200726. RefSeq ID(s) NM_001102659; novel transcript
1.03	RPL32; SNORA7A	ribosomal protein L32; small nucleolar RNA. H/ACA box 7A
1.03	CALM1	calmodulin 1 (phosphorylase kinase. delta)
1.03	FOXI2	forkhead box I2
1.03	DCTD	dCMP deaminase
1.03	GOLGA8G	golgin A8 family. member G
1.03	GSTO1	glutathione S-transferase omega 1
1.03	ABCA13	ATP binding cassette subfamily A member 13
1.03	CYYR1	cysteine/tyrosine-rich 1
1.03	TUBAL3	tubulin. alpha-like 3
1.03	FOS	FBJ murine osteosarcoma viral oncogene homolog
1.03	STT3A	STT3A. subunit of the oligosaccharyltransferase complex (catalytic)
1.03	PKHD1L1	polycystic kidney and hepatic disease 1 (autosomal recessive)-like 1
1.03	JRKL	JRK-like
1.03	RPS6KL1	ribosomal protein S6 kinase-like 1
1.03	MTMR3	myotubularin related protein 3

1.03	DCAF8L1	DDB1 and CUL4 associated factor 8-like 1
1.03	FAM155A	Jeck2013 ALT_ACCEPTOR. ALT_DONOR. coding. INTERNAL. intronic best transcript NM_001080396
1.03	FAM53C	family with sequence similarity 53. member C
1.03	U2SURP	U2 snRNP-associated SURP domain containing
1.03	GREB1L	growth regulation by estrogen in breast cancer-like
1.03	WISP3	WNT1 inducible signaling pathway protein 3
1.03	OR4M2; AC171558.2	Homo sapiens olfactory receptor. family 4. subfamily M. member 2 (OR4M2). mRNA.; Homo sapiens olfactory receptor. family 4. subfamily M. member 2. mRNA (cDNA clone MGC:168600 IMAGE:9020977). complete cds.
1.03	PALM	paralemmin
1.03	ZC3HC1	zinc finger. C3HC-type containing 1
1.03	DHX34	DEAH (Asp-Glu-Ala-His) box polypeptide 34
1.03	RAB40C	RAB40C. member RAS oncogene family
1.03	LECT1	leukocyte cell derived chemotaxin 1
1.03	SAMD9	sterile alpha motif domain containing 9
1.03	SV2B	synaptic vesicle glycoprotein 2B
1.03	BCL2L14	BCL2-like 14 (apoptosis facilitator)
1.03	CLEC14A	C-type lectin domain family 14. member A
1.03	CCDC81	coiled-coil domain containing 81
1.03	PCBP3	poly(rC) binding protein 3
1.03	NEK5	NIMA-related kinase 5
1.03	CHAT	choline O-acetyltransferase
1.03	IFI27	interferon. alpha-inducible protein 27
1.03	LHX5	LIM homeobox 5
1.03	SNX18	sorting nexin 18
1.03	LYRM4	LYR motif containing 4

1.03	CCR6	chemokine (C-C motif) receptor 6
1.03	FAHD2B	fumarylacetoacetate hydrolase domain containing 2B
1.03	SMIM11A	small integral membrane protein 11A
1.03	LOC100130691; AC074286.1; NFE2L2	uncharacterized LOC100130691; Transcript Identified by AceView. Entrez Gene ID(s) 100130691; 4780. RefSeq ID(s) NR_026966; novel transcript
1.03	ATOH8	atonal bHLH transcription factor 8
1.03	WDR86-AS1	WDR86 antisense RNA 1
1.03	GPC1	glypican 1
1.03	SLAIN2	SLAIN motif family member 2
1.03	RAB3IP	RAB3A interacting protein
1.03	PARP1	poly(ADP-ribose) polymerase 1
1.03	CST7	cystatin F (leukocystatin)
1.03	TEX2	testis expressed 2
1.03	OXSR1	oxidative stress responsive 1
1.03	C5orf42	chromosome 5 open reading frame 42
1.03	FAM132A	family with sequence similarity 132. member A
1.03	LIX1	limb and CNS expressed 1
1.03	CDSN	corneodesmosin
1.03	NPIPA7; NPIPA8; PKD1P1	nuclear pore complex interacting protein family. member A7; nuclear pore complex interacting protein family. member A8; polycystic kidney disease 1 (autosomal dominant) pseudogene 1
1.03	GATAD2B	GATA zinc finger domain containing 2B
1.03	IER5L	immediate early response 5-like
1.03	KCNE4	potassium channel. voltage gated subfamily E regulatory beta subunit 4
1.03	ITGAM	Transcript Identified by AceView. Entrez Gene ID(s) 3684
1.03	KIAA0922	KIAA0922
1.03	OR2T5	olfactory receptor. family 2. subfamily T. member 5

1.03	ROR1-AS1	ROR1 antisense RNA 1
1.03	COL4A6	collagen. type IV. alpha 6
1.03	MYOZ2	myozenin 2
1.03	THBS2	thrombospondin 2
1.03	ANKK1	ankyrin repeat and kinase domain containing 1
1.03	VGLL4	vestigial-like family member 4
1.03	STARD8	StAR-related lipid transfer domain containing 8
1.03	RPS25	ribosomal protein S25
1.03	ABCG2	ATP binding cassette subfamily G member 2 (Junior blood group)
1.03	DLEU7	deleted in lymphocytic leukemia. 7
1.03	DHX57	DEAH (Asp-Glu-Ala-Asp/His) box polypeptide 57
1.03	CCDC84	coiled-coil domain containing 84
1.03	SPINK4	serine peptidase inhibitor. Kazal type 4
1.03	PHF10	PHD finger protein 10
1.03	ZNF530	zinc finger protein 530
1.03	TPD52	tumor protein D52
1.03	DGKD	diacylglycerol kinase. delta 130kDa
1.03	FBXL5	F-box and leucine-rich repeat protein 5
1.03	MRC2	mannose receptor. C type 2
1.03	SLC22A13	solute carrier family 22 (organic anion/urate transporter). member 13
1.03	OR51F2	olfactory receptor. family 51. subfamily F. member 2
1.03	SETD3	SET domain containing 3
1.03	ADAMTS3	ADAM metallopeptidase with thrombospondin type 1 motif 3
1.03	SLC5A6	solute carrier family 5 (sodium/multivitamin and iodide cotransporter). member 6
1.03	CARNS1	carnosine synthase 1

1.03	F2	coagulation factor II (thrombin)
1.03	EVI2A; EVI2B	ecotropic viral integration site 2A; ecotropic viral integration site 2B
1.03	INMT; FAM188B; INMT-FAM188B	indolethylamine N-methyltransferase; family with sequence similarity 188. member B; INMT-FAM188B readthrough (NMD candidate)
1.03	NOL6	Memczak2013 ANTISENSE. CDS. coding. INTERNAL. intronic best transcript NM_139235
1.03	SPATS2	spermatogenesis associated. serine-rich 2
1.03	ASB7	ankyrin repeat and SOCS box containing 7
1.03	RPL22	ribosomal protein L22
1.03	TGIF2LX	TGFB-induced factor homeobox 2-like. X-linked
1.03	FAT1	FAT atypical cadherin 1
1.03	VN1R4	vomeronasal 1 receptor 4
1.03	MAPKAPK5; ADAM1A	mitogen-activated protein kinase-activated protein kinase 5; ADAM metallopeptidase domain 1A (pseudogene)
1.03	ZNF729	zinc finger protein 729
1.03	TYMSOS	TYMS opposite strand
1.03	OLIG3	oligodendrocyte transcription factor 3
1.03	PDE4DIP	phosphodiesterase 4D interacting protein
1.03	CD101	CD101 molecule
1.03	FAM196A	family with sequence similarity 196. member A
1.03	MTG1	mitochondrial ribosome-associated GTPase 1
1.03	NUP62CL	nucleoporin 62kDa C-terminal like
1.03	CDK13	cyclin-dependent kinase 13
1.03	TCTE1	t-complex-associated-testis-expressed 1
1.03	U2AF1	U2 small nuclear RNA auxiliary factor 1
1.03	CNOT7	CCR4-NOT transcription complex subunit 7
1.03	ANKRD52	ankyrin repeat domain 52

1.03	CYP1A1	cytochrome P450. family 1. subfamily A. polypeptide 1
1.03	OR13C4	olfactory receptor. family 13. subfamily C. member 4
1.03	ZNF556	zinc finger protein 556
1.03	MTCH1	mitochondrial carrier 1
1.03	buskee; FAM25D; FAM25E; zawskaw	Transcript Identified by AceView; family with sequence similarity 25. member D [Source:HGNC Symbol;Acc:23588]; family with sequence similarity 25. member E [Source:HGNC Symbol;Acc:HGNC:23587]
1.03	PGLYRP3	peptidoglycan recognition protein 3
1.03	SCUBE1	signal peptide. CUB domain. EGF-like 1
1.03	NAA35	N(alpha)-acetyltransferase 35. NatC auxiliary subunit
1.03	SULF2	Memczak2013 ANTISENSE. coding. INTERNAL. intronic best transcript NM_001161841
1.03	KLHL18	kelch-like family member 18
1.03	CCR5	chemokine (C-C motif) receptor 5 (gene/pseudogene)
1.03	ZNF200	zinc finger protein 200
1.03	SEMA6B	sema domain. transmembrane domain (TM). and cytoplasmic domain. (semaphorin) 6B
1.03	ANKDD1A	ankyrin repeat and death domain containing 1A
1.03	LRRC58	Memczak2013 ANTISENSE. CDS. coding. INTERNAL. intronic. UTR3 best transcript NM_001099678
1.03	RUSC1-AS1	RUSC1 antisense RNA 1
1.03	ZHX1	zinc fingers and homeoboxes 1
1.03	PTP4A1	protein tyrosine phosphatase type IVA. member 1
1.03	PTGES2	prostaglandin E synthase 2
1.02	RBPJ	recombination signal binding protein for immunoglobulin kappa J region
1.02	SMCR5	Smith-Magenis syndrome chromosome region. candidate 5 (non-protein coding)
1.02	FRMD3	FERM domain containing 3

1.02	AMY2A	amylase. alpha 2A (pancreatic)
1.02	GTPBP3	Memczak2013 ANTISENSE. CDS. coding. INTERNAL best transcript NM_001195422
1.02	HERC6	Memczak2013 ANTISENSE. coding. INTERNAL. intronic best transcript NM_001165136
1.02	TXK	TXK tyrosine kinase
1.02	CD200R1L	CD200 receptor 1 like
1.02	CACNB2	calcium channel. voltage-dependent. beta 2 subunit
1.02	DPM1	dolichyl-phosphate mannosyltransferase polypeptide 1. catalytic subunit
1.02	TMTC1	transmembrane and tetratricopeptide repeat containing 1
1.02	CLOCK	clock circadian regulator
1.02	OR4K2	olfactory receptor. family 4. subfamily K. member 2
1.02	ILDR2	immunoglobulin-like domain containing receptor 2
1.02	INVS	inversin
1.02	ASTN2	astrotactin 2
1.02	OR10G4	olfactory receptor. family 10. subfamily G. member 4
1.02	CCL11	chemokine (C-C motif) ligand 11
1.02	C2orf91	chromosome 2 open reading frame 91
1.02	WFDC2	WAP four-disulfide core domain 2
1.02	FBXO4	F-box protein 4
1.02	PRSS45	protease. serine 45
1.02	TVP23C; CDRT4; TVP23C-CDRT4	trans-golgi network vesicle protein 23 homolog C (S. cerevisiae); CMT1A duplicated region transcript 4; TVP23C-CDRT4 readthrough
1.02	ASCL3	achaete-scute family bHLH transcription factor 3
1.02	PRAMEF25	PRAME family member 25
1.02	ALDH9A1	aldehyde dehydrogenase 9 family. member A1

1.02	PCDHA9; PCDHAC1; PCDHAC2; PCDHA1; PCDHA10; PCDHA11; PCDHA12; PCDHA13; PCDHA2; PCDHA3; PCDHA4; PCDHA5; PCDHA6; PCDHA7; PCDHA8; PCDHA14	protocadherin alpha 9; protocadherin alpha subfamily C. 1; protocadherin alpha subfamily C. 2; protocadherin alpha 1; protocadherin alpha 10; protocadherin alpha 11; protocadherin alpha 12; protocadherin alpha 13; protocadherin alpha 2; protocadherin alpha 3; protocadherin alpha 4; protocadherin alpha 5; protocadherin alpha 6; protocadherin alpha 7; protocadherin alpha 8; protocadherin alpha 14 pseudogene
1.02	ARHGAP27	Rho GTPase activating protein 27
1.02	RPL38	ribosomal protein L38
1.02	LRRK1	leucine-rich repeat kinase 1
1.02	MYO1C	myosin IC
1.02	ZNF692	zinc finger protein 692
1.02	UTP20	UTP20 small subunit (SSU) processome component
1.02	DDX39A	DEAD (Asp-Glu-Ala-Asp) box polypeptide 39A
1.02	FAM71C	family with sequence similarity 71. member C
1.02	LAMA4	laminin. alpha 4
1.02	BACE1	beta-site APP-cleaving enzyme 1
1.02	RPL13; SNORD68	ribosomal protein L13; small nucleolar RNA. C/D box 68
1.02	SH2B1	SH2B adaptor protein 1
1.02	RGS4	regulator of G-protein signaling 4
1.02	RIMKLB	ribosomal modification protein rimK-like family member B
1.02	LINC00272	long intergenic non-protein coding RNA 272
1.02	PYM1	PYM homolog 1. exon junction complex associated factor
1.02	E2F1	Memczak2013 ANTISENSE. coding. INTERNAL. intronic best transcript NM_005225
1.02	ARHGAP11B	Rho GTPase activating protein 11B
1.02	CCDC39	coiled-coil domain containing 39
1.02	PCBP3	poly(rC) binding protein 3
1.02	C3AR1	complement component 3a receptor 1

1.02	IL13RA1	interleukin 13 receptor. alpha 1
1.02	PEX1	peroxisomal biogenesis factor 1
1.02	RGSL1	regulator of G-protein signaling like 1
1.02	SECISBP2L	SECIS binding protein 2-like
1.02	ZSWIM2	zinc finger. SWIM-type containing 2
1.02	FLNB	filamin B. beta
1.02	LINC00243	long intergenic non-protein coding RNA 243
1.02	FAM105A	family with sequence similarity 105. member A
1.02	TRPM4	transient receptor potential cation channel. subfamily M. member 4
1.02	NOL11	nucleolar protein 11
1.02	CADM2	cell adhesion molecule 2
1.02	MSR1	macrophage scavenger receptor 1
1.02	TMOD4	tropomodulin 4 (muscle)
1.02	OR10A6	olfactory receptor. family 10. subfamily A. member 6 (gene/pseudogene)
1.02	COL1A1	collagen. type I. alpha 1
1.02	VCX3B; VCX	variable charge. X-linked 3B; variable charge. X-linked
1.02	TBCD	Transcript Identified by AceView. Entrez Gene ID(s) 6904
1.02	STARD13	StAR-related lipid transfer domain containing 13
1.02	SLC48A1	solute carrier family 48 (heme transporter). member 1
1.02	POLL	polymerase (DNA directed). lambda
1.02	PUS1	pseudouridylate synthase 1
1.02	PSMD5	proteasome 26S subunit. non-ATPase 5
1.02	SLC15A1	solute carrier family 15 (oligopeptide transporter). member 1
1.02	DDB2	damage-specific DNA binding protein 2
1.02	WNK4	WNK lysine deficient protein kinase 4
1.02	PAGE2B	P antigen family. member 2B

1.02	ELL3	elongation factor RNA polymerase II-like 3
1.02	RNF216	ring finger protein 216
1.02	IMMP2L	inner mitochondrial membrane peptidase subunit 2
1.02	PGLYRP1	peptidoglycan recognition protein 1
1.02	SLC26A10	solute carrier family 26. member 10
1.02	TTN	titin
1.02	ZBTB20; MIR568	zinc finger and BTB domain containing 20; microRNA 568
1.02	FBL	fibrillarin
1.02	RBMX2	RNA binding motif protein. X-linked 2
1.02	TCF7L2	transcription factor 7-like 2 (T-cell specific. HMG-box)
1.02	LMOD2	leiomodin 2 (cardiac)
1.02	ABCA9	ATP binding cassette subfamily A member 9
1.02	SIT1	signaling threshold regulating transmembrane adaptor 1
1.02	DCSTAMP	dendrocyte expressed seven transmembrane protein
1.02	ALCAM	activated leukocyte cell adhesion molecule
1.02	ATG16L1	autophagy related 16-like 1
1.02	NRBF2	nuclear receptor binding factor 2
1.02	HMGB3	high mobility group box 3
1.02	CPOX	coproporphyrinogen oxidase
1.02	MXRA5	matrix-remodelling associated 5
1.02	CSN1S1	casein alpha s1
1.02	IER3IP1	immediate early response 3 interacting protein 1
1.02	MGAM	maltase-glucoamylase
1.02	ADORA3; TMIGD3	adenosine A3 receptor; transmembrane and immunoglobulin domain containing 3
1.02	ADM	adrenomedullin

1.02	CASC1	cancer susceptibility candidate 1
1.02	SLC44A4	solute carrier family 44. member 4
1.02	CHMP5	charged multivesicular body protein 5
1.02	PADI3	peptidyl arginine deiminase. type III
1.02	TSFM	Ts translation elongation factor. mitochondrial
1.02	LINC00656	long intergenic non-protein coding RNA 656
1.02	GMIP	GEM interacting protein
1.02	AGL	amylo-alpha-1. 6-glucosidase. 4-alpha-glucanotransferase
1.02	KRTAP21-2	keratin associated protein 21-2
1.02	ACSL3	acyl-CoA synthetase long-chain family member 3
1.02	VPS8	vacuolar protein sorting 8 homolog (S. cerevisiae)
1.02	ANXA11	annexin A11
1.02	SP4	Sp4 transcription factor
1.02	TISP43; LOC646743; LOC150527	Homo sapiens uncharacterized LOC150527 (TISP43). transcript variant 1. non-coding RNA.; Homo sapiens uncharacterized LOC150527 (TISP43). transcript variant 2. non-coding RNA.; Homo sapiens uncharacterized LOC646743 (LOC646743). non-coding RNA.; Transcript Identified by AceView. Entrez Gene ID(s) 150527; Transcript Identified by AceView. Entrez Gene ID(s) 150527. RefSeq ID(s) NR_027313; uncharacterized LOC150527 [Source:EntrezGene;Acc:150527]
1.02	BTN2A1	butyrophilin. subfamily 2. member A1
1.02	PCBP2; PCBP2-OT1	poly(rC) binding protein 2; PCBP2 overlapping transcript 1
1.02	TEX29	testis expressed 29
1.02	KRTAP5-AS1	KRTAP5-1/KRTAP5-2 antisense RNA 1
1.02	HYOU1	hypoxia up-regulated 1
1.02	CRYGD	crystallin gamma D
1.02	DKK3	dickkopf WNT signaling pathway inhibitor 3
1.02	PRRX1	paired related homeobox 1

1.02	CFL1	cofilin 1 (non-muscle)
1.02	KCNH8	potassium channel. voltage gated eag related subfamily H. member 8
1.02	TGM4	transglutaminase 4
1.02	CCL7	chemokine (C-C motif) ligand 7
1.02	CHRNG	cholinergic receptor. nicotinic gamma
1.02	CINP	cyclin-dependent kinase 2 interacting protein
1.02	CELF1	CUGBP. Elav-like family member 1
1.02	PRICKLE2	prickle homolog 2
1.02	EIF2B4	eukaryotic translation initiation factor 2B. subunit 4 delta. 67kDa
1.02	PADI1	peptidyl arginine deiminase. type I
1.02	HECTD2	Transcript Identified by AceView. Entrez Gene ID(s) 143279
1.02	TXNDC17	thioredoxin domain containing 17
1.02	ZFP62	ZFP62 zinc finger protein
1.02	ST18	suppression of tumorigenicity 18. zinc finger
1.02	VIM	Jeck2013 ANTISENSE. coding. INTERNAL. intronic best transcript NM_003380
1.02	USP13	Memczak2013 ANTISENSE. coding. INTERNAL. intronic best transcript NM_003940
1.02	KLF9	Kruppel-like factor 9
1.02	RCN1	reticulocalbin 1. EF-hand calcium binding domain
1.02	RAP1GAP	RAP1 GTPase activating protein
1.02	ACSBG2	acyl-CoA synthetase bubblegum family member 2
1.02	HNRNPAB	heterogeneous nuclear ribonucleoprotein A/B
1.02	SBNO1; MIR8072	strawberry notch homolog 1 (Drosophila); microRNA 8072
1.02	CAST	calpastatin
1.02	TFE3	transcription factor binding to IGHM enhancer 3
1.02	FOXB1	forkhead box B1

1.02	FAR1	fatty acyl CoA reductase 1
1.02	SDHD	succinate dehydrogenase complex subunit D. integral membrane protein
1.02	KLHL24	kelch-like family member 24
1.02	CCL24	chemokine (C-C motif) ligand 24
1.02	ZMAT3	zinc finger. matrin-type 3
1.02	DGKD	diacylglycerol kinase. delta 130kDa
1.02	SLC16A1	solute carrier family 16 (monocarboxylate transporter). member 1
1.02	GRM3	glutamate receptor. metabotropic 3
1.02	NKX2-2	NK2 homeobox 2
1.02	C9orf78	chromosome 9 open reading frame 78
1.02	AKIP1	A kinase (PRKA) interacting protein 1
1.02	FRRS1L	ferric-chelate reductase 1-like
1.02	OSBPL1A	Transcript Identified by AceView. Entrez Gene ID(s) 114876
1.02	CERKL	ceramide kinase like
1.02	ago-03	argonaute RISC catalytic component 3
1.02	CEACAM8	carcinoembryonic antigen-related cell adhesion molecule 8
1.02	PRR23B	proline rich 23B
1.02	ZNF607	zinc finger protein 607
1.02	KCNIP3	Kv channel interacting protein 3. calsenilin
1.02	HTR1E	5-hydroxytryptamine (serotonin) receptor 1E. G protein-coupled
1.02	CLPB	ClpB homolog. mitochondrial AAA ATPase chaperonin
1.02	BCL2L14	BCL2-like 14 (apoptosis facilitator)
1.02	SLC41A3	solute carrier family 41. member 3
1.02	OR4D11	olfactory receptor. family 4. subfamily D. member 11
1.02	NYAP2	neuronal tyrosine-phosphorylated phosphoinositide-3-kinase adaptor 2
1.02	SLC27A2	solute carrier family 27 (fatty acid transporter). member 2

1.02	TMEM155	transmembrane protein 155
1.02	HACD3	3-hydroxyacyl-CoA dehydratase 3
1.02	LMBR1L	limb development membrane protein 1-like
1.02	OSTCP1	oligosaccharyltransferase complex subunit pseudogene 1
1.02	TTC9C	tetratricopeptide repeat domain 9C
1.02	TCF3	transcription factor 3
1.02	KLRF2	killer cell lectin-like receptor subfamily F. member 2
1.02	ZFYVE19	zinc finger. FYVE domain containing 19
1.02	CNTN3	contactin 3 (plasmacytoma associated)
1.02	OR10A6	olfactory receptor. family 10. subfamily A. member 6 (gene/pseudogene)
1.02	OR10A6	olfactory receptor family 10 subfamily A member 6 (gene/pseudogene) [Source:HGNC Symbol;Acc:HGNC:15132]
1.02	SENP5	SUMO1/sentrin specific peptidase 5
1.02	AFAP1L1	actin filament associated protein 1-like 1
1.02	GPT2	glutamic pyruvate transaminase (alanine aminotransferase) 2
1.02	GSK3B	glycogen synthase kinase 3 beta
1.02	UFSP1	UFM1-specific peptidase 1 (inactive)
1.02	GALNT3	polypeptide N-acetylgalactosaminyltransferase 3
1.02	APTX	aprataxin
1.02	PPME1	protein phosphatase methylesterase 1
1.02	LINC01559	long intergenic non-protein coding RNA 1559
1.02	RNMTL1	RNA methyltransferase like 1
1.02	POM121L2	POM121 transmembrane nucleoporin-like 2
1.02	RBMS1	Memczak2013 ALT_ACCEPTOR. ALT_DONOR. coding. INTERNAL. intronic best transcript NM_016836
1.02	NLGN1	neuroligin 1
1.02	SPEN	spen family transcriptional repressor

1.02	PPCDC	phosphopantothenoylcysteine decarboxylase
1.02	USP17L19	ubiquitin specific peptidase 17-like family member 19
1.02	ANKRD20A1	ankyrin repeat domain 20 family. member A1
1.02	CTTNBP2	cortactin binding protein 2
1.02	HNRNPU	heterogeneous nuclear ribonucleoprotein U (scaffold attachment factor A)
1.02	GPR158-AS1	GPR158 antisense RNA 1
1.02	CHKB-CPT1B	CHKB-CPT1B readthrough (NMD candidate)
1.02	ERCC3	excision repair cross-complementation group 3
1.02	PITX3	paired-like homeodomain 3
1.02	HYOU1	hypoxia up-regulated 1
1.02	AIF1L	allograft inflammatory factor 1-like
1.02	TMEM235	transmembrane protein 235
1.02	TSPY2	testis specific protein. Y-linked 2
1.02	UQCRHL	ubiquinol-cytochrome c reductase hinge protein like
1.02	ZFYVE1	zinc finger. FYVE domain containing 1
1.02	SYNPR	synaptoporin
1.02	PCMTD2	protein-L-isoaspartate (D-aspartate) O-methyltransferase domain containing 2
1.02	MAGI3	membrane associated guanylate kinase. WW and PDZ domain containing 3
1.02	OR10R2	olfactory receptor. family 10. subfamily R. member 2
1.02	OXSM	3-oxoacyl-ACP synthase. mitochondrial
1.02	FCGR3B	Fc fragment of IgG. low affinity IIIb. receptor (CD16b)
1.02	C4BPA	complement component 4 binding protein. alpha
1.02	UBB	ubiquitin B
1.02	HLA-DQB2	major histocompatibility complex. class II. DQ beta 2
1.02	TPM3	tropomyosin 3

1.02	LOC102724238; LOC554249; RP11-111F5.4	Homo sapiens uncharacterized LOC102724238 (LOC102724238). long non-coding RNA.; Homo sapiens uncharacterized LOC554249 (LOC554249). long non-coding RNA.; Homo sapiens hypothetical LOC554249. mRNA (cDNA clone MGC:99799 IMAGE:6378300). complete cds.; novel transcript
1.02	TMPRSS4	transmembrane protease. serine 4
1.02	NUDT5	nudix hydrolase 5
1.02	IL17RA	interleukin 17 receptor A
1.02	A2BP1	Transcript Identified by AceView. Entrez Gene ID(s) 54715
1.02	PRSS41	protease. serine 41
1.02	TAPBP	TAP binding protein (tapasin)
1.02	SLC30A9	solute carrier family 30 (zinc transporter). member 9
1.02	CCR3	chemokine (C-C motif) receptor 3
1.02	PLPP4	phospholipid phosphatase 4
1.02	TRPM5	transient receptor potential cation channel. subfamily M. member 5
1.02	PAGE1	P antigen family. member 1 (prostate associated)
1.02	LGI1	leucine-rich. glioma inactivated 1
1.02	KRTCAP2	keratinocyte associated protein 2
1.02	SPIN1	spindlin 1
1.02	HEG1	heart development protein with EGF-like domains 1
1.02	CXCL10	chemokine (C-X-C motif) ligand 10
1.02	HLTF	helicase-like transcription factor
1.02	LINC01565	long intergenic non-protein coding RNA 1565
1.02	HYOU1	hypoxia up-regulated 1
1.02	FAM231A	family with sequence similarity 231. member A
1.02	SHISA9	shisa family member 9
1.02	ACVR2A	activin A receptor type IIA
1.02	ATG16L1	autophagy related 16-like 1

1.02	MYOT	myotilin
1.02	USP48	ubiquitin specific peptidase 48
1.02	DOHH	deoxyhypusine hydroxylase/monooxygenase
1.02	ZMAT1	zinc finger. matrin-type 1
1.02	PKD1; MIR6511B2; MIR6511B1	polycystic kidney disease 1 (autosomal dominant); microRNA 6511b-2; microRNA 6511b-1
1.02	PTCD1	pentatricopeptide repeat domain 1
1.02	OTUD5	OTU deubiquitinase 5
1.02	MTIF3	mitochondrial translational initiation factor 3
1.02	THAP9-AS1	THAP9 antisense RNA 1
1.02	LOC388692; FAM231D; RP11-403I13.7; RP11-403I13.8	Homo sapiens uncharacterized LOC388692 (LOC388692). long non-coding RNA.; Homo sapiens family with sequence similarity 231. member D (FAM231D). long non-coding RNA.; novel transcript
1.02	C2CD4C	C2 calcium-dependent domain containing 4C
1.02	SLC22A12	solute carrier family 22 (organic anion/urate transporter). member 12
1.02	MTRNR2L4	MT-RNR2-like 4
1.02	DGKD	diacylglycerol kinase. delta 130kDa
1.02	MEIOC	meiosis specific with coiled-coil domain
1.02	NCOA4	nuclear receptor coactivator 4
1.02	GAPDHS	glyceraldehyde-3-phosphate dehydrogenase. spermatogenic
1.02	ALG13	Transcript Identified by AceView. Entrez Gene ID(s) 79868
1.02	PIH1D3	PIH1 domain containing 3
1.02	PRSS33	protease. serine. 33
1.02	SNRPA	small nuclear ribonucleoprotein polypeptide A
1.02	SLIT2	Jeck2013 ALT_ACCEPTOR. ALT_DONOR. coding. INTERNAL. intronic best transcript NM_004787
1.02	IFNE; MIR31HG	interferon. epsilon; MIR31 host gene

1.02	PPIL6	peptidylprolyl isomerase (cyclophilin)-like 6
1.02	GAL3ST2	galactose-3-O-sulfotransferase 2
1.02	MYCL	v-myc avian myelocytomatosis viral oncogene lung carcinoma derived homolog
1.02	C1GALT1C1L	C1GALT1-specific chaperone 1 like
1.02	IPCEF1	interaction protein for cytohesin exchange factors 1
1.02	SH3GL1	SH3-domain GRB2-like 1
1.02	CRLF2	cytokine receptor-like factor 2
1.02	NXF3	nuclear RNA export factor 3
1.02	CLDN23	claudin 23
1.02	NBN	nibrin
1.02	FLRT1	fibronectin leucine rich transmembrane protein 1
1.02	HSPA8; SNORD14C; SNORD14D	heat shock 70kDa protein 8; small nucleolar RNA. C/D box 14C; small nucleolar RNA. C/D box 14D
1.02	CCNY	cyclin Y
1.02	ORC2	origin recognition complex subunit 2
1.02	ODF4	outer dense fiber of sperm tails 4
1.02	PEX12	peroxisomal biogenesis factor 12
1.02	CXCL12	chemokine (C-X-C motif) ligand 12
1.02	RSBN1	round spermatid basic protein 1
1.02	ATG13	autophagy related 13
1.02	THOC6	THO complex 6
1.02	TXLNG	taxilin gamma
1.02	PCNXL4	pecanex-like 4 (Drosophila)
1.02	RALA	v-ral simian leukemia viral oncogene homolog A (ras related)
1.02	SIAH1	siah E3 ubiquitin protein ligase 1
1.02	HMBS	hydroxymethylbilane synthase

1.02	SCAP	SREBF chaperone
1.02	DHX35	DEAH (Asp-Glu-Ala-His) box polypeptide 35
1.02	TRIM49D1; TRIM49D2	tripartite motif containing 49D1; tripartite motif containing 49D2
1.02	TNIP3	TNFAIP3 interacting protein 3
1.02	XAGE5	X antigen family. member 5
1.02	SOX30	SRY box 30
1.02	CYP4B1	cytochrome P450. family 4. subfamily B. polypeptide 1
1.02	DALRD3	DALR anticodon binding domain containing 3
1.02	DBX1	developing brain homeobox 1
1.02	SLAMF6	SLAM family member 6
1.02	NACC1	nucleus accumbens associated 1. BEN and BTB (POZ) domain containing
1.02	ADIG	adipogenin
1.02	TTC39B	Transcript Identified by AceView. Entrez Gene ID(s) 158219
1.02	MAPK10	mitogen-activated protein kinase 10
1.02	VN1R10P	vomeronasal 1 receptor 10 pseudogene
1.02	IGF2BP1	insulin-like growth factor 2 mRNA binding protein 1
1.02	MSH6	mutS homolog 6
1.02	CIAPIN1	cytokine induced apoptosis inhibitor 1
1.02	CRMP1	collapsin response mediator protein 1
1.02	PDE5A	phosphodiesterase 5A. cGMP-specific
1.02	CYP51A1	cytochrome P450. family 51. subfamily A. polypeptide 1
1.02	SMIM11A	small integral membrane protein 11A
1.02	CNTLN	Transcript Identified by AceView. Entrez Gene ID(s) 54875
1.02	KRTAP25-1	keratin associated protein 25-1
1.02	SAMSN1	SAM domain. SH3 domain and nuclear localization signals 1
1.02	XIRP2	xin actin binding repeat containing 2

1.02	SAP18	Sin3A associated protein 18kDa
1.02	RRP9	ribosomal RNA processing 9. small subunit (SSU) processome component. homolog (yeast)
1.02	TADA2B	transcriptional adaptor 2B
1.02	HYOU1	hypoxia up-regulated 1
1.02	AIMP2	aminoacyl tRNA synthetase complex-interacting multifunctional protein 2
1.02	TNFSF12-TNFSF13	TNFSF12-TNFSF13 readthrough
1.02	NMS	neuromedin S
1.02	MAPRE1	microtubule-associated protein. RP/EB family. member 1
1.02	FAM65B	family with sequence similarity 65. member B
1.02	TGM1	transglutaminase 1
1.02	CERS3	ceramide synthase 3
1.02	CACFD1	calcium channel flower domain containing 1
1.02	MAP1A	microtubule associated protein 1A
1.02	DDIT4L	DNA-damage-inducible transcript 4-like
1.02	OR8K1	olfactory receptor. family 8. subfamily K. member 1
1.02	ETHE1	ethylmalonic encephalopathy 1
1.02	PRRG1	proline rich Gla (G-carboxyglutamic acid) 1
1.02	CHD1L	chromodomain helicase DNA binding protein 1-like
1.02	AACS	acetoacetyl-CoA synthetase
1.02	HDLBP	Transcript Identified by AceView. Entrez Gene ID(s) 3069
1.02	ALPK3	alpha kinase 3
1.02	ERICH4	glutamate rich 4
1.02	TNFRSF14	tumor necrosis factor receptor superfamily. member 14
1.02	SELV	selenoprotein V; selenoprotein V [Source:EntrezGene;Acc:348303]; Transcript Identified by AceView. Entrez Gene ID(s) 348303. RefSeq ID(s) NM_182704

1.02	CACNG2	calcium channel. voltage-dependent. gamma subunit 2
1.02	OR2W5	olfactory receptor. family 2. subfamily W. member 5 (gene/pseudogene)
1.02	ZXDC	ZXD family zinc finger C
1.02	GRIN2A	glutamate receptor. ionotropic. N-methyl D-aspartate 2A
1.02	TTC39A	tetratricopeptide repeat domain 39A
1.02	CFL1	cofilin 1 (non-muscle)
1.02	CD80	CD80 molecule
1.02	SI	sucrase-isomaltase (alpha-glucosidase)
1.02	SYPL2	synaptophysin-like 2
1.02	FOXN1	forkhead box N1
1.02	USP21	ubiquitin specific peptidase 21
1.02	ITPK1	inositol-tetrakisphosphate 1-kinase
1.02	SLC22A18	solute carrier family 22. member 18
1.02	IL3RA	interleukin 3 receptor. alpha (low affinity)
1.02	BRINP3	bone morphogenetic protein/retinoic acid inducible neural-specific 3
1.02	ZNF790	zinc finger protein 790
1.02	SLC2A12	solute carrier family 2 (facilitated glucose transporter). member 12
1.02	HPSE2	heparanase 2 (inactive)
1.02	DYNC1I1	dynein. cytoplasmic 1. intermediate chain 1
1.02	RNASEL	ribonuclease L (2.5-oligoisoadenylate synthetase-dependent)
1.02	TMEM167A	transmembrane protein 167A
1.02	IDH3A	isocitrate dehydrogenase 3 (NAD+) alpha
1.02	KRTAP4-6	keratin associated protein 4-6
1.02	ERGIC1	endoplasmic reticulum-golgi intermediate compartment 1
1.02	RBSN	rabenosyn. RAB effector
1.02	CD2BP2	CD2 (cytoplasmic tail) binding protein 2

1.02	PIP4K2C	phosphatidylinositol-5-phosphate 4-kinase. type II. gamma
1.02	FAM86B1	family with sequence similarity 86. member B1
1.02	RAB11FIP3	RAB11 family interacting protein 3 (class II)
1.02	INPP5D	inositol polyphosphate-5-phosphatase D
1.02	LRRC43	leucine rich repeat containing 43
1.02	IWS1	IWS1 homolog (S. cerevisiae)
1.02	FAM9B	family with sequence similarity 9. member B
1.02	ASPN	asporin
1.02	SLC16A1	solute carrier family 16 (monocarboxylate transporter). member 1
1.02	CEND1	cell cycle exit and neuronal differentiation 1
1.02	THSD7B	thrombospondin type 1 domain containing 7B
1.02	LOC388780; RP11-128M1.1	uncharacterized LOC388780; putative novel transcript
1.02	MROH9	maestro heat-like repeat family member 9
1.02	SLC27A3	solute carrier family 27 (fatty acid transporter). member 3
1.02	PAPSS2	3-phosphoadenosine 5-phosphosulfate synthase 2
1.02	ZCCHC13	zinc finger. CCHC domain containing 13
1.02	SLC12A1	solute carrier family 12 (sodium/potassium/chloride transporter). member 1
1.02	ARHGEF1	Rho guanine nucleotide exchange factor 1
1.02	RXRB	retinoid X receptor beta
1.02	IRX6	iroquois homeobox 6
1.02	LEP	leptin
1.02	EPB42	erythrocyte membrane protein band 4.2
1.02	CRISP1	cysteine-rich secretory protein 1
1.02	KCNK16	potassium channel. two pore domain subfamily K. member 16
1.02	SKOR2	SKI family transcriptional corepressor 2
1.02	NGRN; TTLL13P	neugrin. neurite outgrowth associated; tubulin tyrosine ligase-like family

		member 13. pseudogene
1.02	FAM150B	family with sequence similarity 150. member B
1.02	SLAMF7	SLAM family member 7
1.02	LOC100505478; RP11-402G3.3	uncharacterized LOC100505478; putative novel transcript
1.02	PCDH18	protocadherin 18
1.02	NTAN1	N-terminal asparagine amidase
1.02	PHC1	polyhomeotic homolog 1 (Drosophila)
1.02	PDS5B	PDS5 cohesin associated factor B
1.02	TGM4	transglutaminase 4
1.02	NOMO3; NOMO2	NODAL modulator 3; NODAL modulator 2
1.02	PLD1	phospholipase D1. phosphatidylcholine-specific
1.02	WNT1	wingless-type MMTV integration site family. member 1
1.02	GATC	glutamyl-tRNA(Gln) amidotransferase. subunit C
1.02	CEACAM4	carcinoembryonic antigen-related cell adhesion molecule 4
1.02	CSPG4P1Y	chondroitin sulfate proteoglycan 4 pseudogene 1. Y-linked
1.02	RGL1	ral guanine nucleotide dissociation stimulator-like 1
1.02	NASP	Zhang2013 ALT_DONOR. coding. INTERNAL. intronic best transcript NM_002482
1.02	FBXO31	Memczak2013 ANTISENSE. INTERNAL. intronic. ncRNA best transcript NR_024568
1.02	DUSP13	dual specificity phosphatase 13
1.02	RPS2; SNORA64; SNORA10	ribosomal protein S2; small nucleolar RNA. H/ACA box 64; small nucleolar RNA. H/ACA box 10
1.02	LOC654841; AC097662.2; COL4A3	uncharacterized LOC654841; Salzman2013 ANTISENSE. CDS. coding. INTERNAL. intronic. OVCODE. OVERLAPTX. OVEXON best transcript NM_000091; novel transcript. antisense to COL4A3
1.02	RHOQ	ras homolog family member Q
1.02	CAPN11	calpain 11

1.02	PMPCA	peptidase (mitochondrial processing) alpha
1.02	CBLB	Cbl proto-oncogene B. E3 ubiquitin protein ligase
1.02	C10orf91	chromosome 10 open reading frame 91
1.02	SYNPO	synaptopodin
1.02	MAGEA11	MAGE family member A11
1.02	C19orf53	chromosome 19 open reading frame 53
1.02	ITGAL	integrin alpha L
1.02	RABGGTB	Zhang2013 ALT_ACCEPTOR. ALT_DONOR. coding. INTERNAL. intronic best transcript NM_004582
1.02	SNX13	sorting nexin 13
1.02	GRIK4	glutamate receptor. ionotropic. kainate 4
1.02	STKLD1	serine/threonine kinase-like domain containing 1 [Source:HGNC Symbol;Acc:HGNC:28669]
1.02	TTC23	tetratricopeptide repeat domain 23
1.02	TNXA	tenascin XA (pseudogene)
1.02	HAPLN2	hyaluronan and proteoglycan link protein 2
1.02	INSRR	insulin receptor-related receptor
1.02	KCNA6; GALNT8	potassium channel. voltage gated shaker related subfamily A. member 6; polypeptide N-acetylgalactosaminyltransferase 8
1.02	CST4	cystatin S
1.02	OR2C1	olfactory receptor. family 2. subfamily C. member 1
1.02	HYOU1	hypoxia up-regulated 1
1.02	MLXIP	MLX interacting protein
1.02	RRAGB	Ras-related GTP binding B
1.02	SHE	Src homology 2 domain containing E
1.02	PSMD4	proteasome 26S subunit. non-ATPase 4
1.02	LRRC18	leucine rich repeat containing 18

1.02	BORCS8; MEF2B; MEF2BNB-MEF2B	BLOC-1 related complex subunit 8; myocyte enhancer factor 2B; MEF2BNB-MEF2B readthrough
1.02	PRAC2	prostate cancer susceptibility candidate 2
1.02	RAB39A	RAB39A. member RAS oncogene family
1.02	LY6G6D	lymphocyte antigen 6 complex. locus G6D
1.02	PMM1	phosphomannomutase 1
1.02	CTNNBL1	catenin. beta like 1
1.02	CNOT6L	CCR4-NOT transcription complex subunit 6-like
1.02	PPP2R3B	protein phosphatase 2. regulatory subunit B. beta
1.02	PPP2R3B	protein phosphatase 2. regulatory subunit B. beta
1.02	OVOL3	ovo-like zinc finger 3
1.02	POLR2A	polymerase (RNA) II (DNA directed) polypeptide A. 220kDa
1.02	NAE1	NEDD8 activating enzyme E1 subunit 1
1.02	ADCY4	adenylate cyclase 4
1.02	ALKBH7	alkB homolog 7
1.02	SIRPG	signal-regulatory protein gamma
1.02	C15orf43	chromosome 15 open reading frame 43
1.02	INAFM2	InaF-motif containing 2
1.02	MAN1B1-AS1	MAN1B1 antisense RNA 1 (head to head)
1.02	ZNF114	zinc finger protein 114
1.02	CGB1	chorionic gonadotropin. beta polypeptide 1
1.02	HACD4	3-hydroxyacyl-CoA dehydratase 4
1.02	CHRM2	cholinergic receptor. muscarinic 2
1.02	ULK1	unc-51 like autophagy activating kinase 1
1.02	NXN	nucleoredoxin
1.02	EIF3G	eukaryotic translation initiation factor 3. subunit G

1.02	CTBP1	C-terminal binding protein 1
1.02	SETDB2	SET domain. bifurcated 2
1.02	XPC	xeroderma pigmentosum. complementation group C
1.02	CINP	cyclin-dependent kinase 2 interacting protein
1.02	TCEB1	transcription elongation factor B (SIII). polypeptide 1 (15kDa. elongin C)
1.02	ZBTB5	zinc finger and BTB domain containing 5
1.02	NPAP1	nuclear pore associated protein 1
1.02	TRERF1	transcriptional regulating factor 1
1.02	CMTM1	CKLF-like MARVEL transmembrane domain containing 1
1.02	PPP2R4	protein phosphatase 2A activator. regulatory subunit 4
1.02	TOMM20L	translocase of outer mitochondrial membrane 20 homolog (yeast)-like
1.02	BAG6	BCL2 associated athanogene 6
1.02	SMIM2	small integral membrane protein 2
1.02	NUDT6	nudix hydrolase 6
1.02	KCTD8	potassium channel tetramerization domain containing 8
1.02	SYNE2	spectrin repeat containing. nuclear envelope 2
1.02	VAT1L	vesicle amine transport 1-like
1.01	RGL4	ral guanine nucleotide dissociation stimulator-like 4
1.01	CTAGE6	CTAGE family. member 6
1.01	EIF4H	eukaryotic translation initiation factor 4H
1.01	GABRA6	gamma-aminobutyric acid (GABA) A receptor. alpha 6
1.01	NPPB	natriuretic peptide B
1.01	ANKMY1	ankyrin repeat and MYND domain containing 1
1.01	ACRC	acidic repeat containing
1.01	CTSG	cathepsin G
1.01	NRTN	neurturin

1.01	HOXB3; HOXB4; MIR10A	homeobox B3; homeobox B4; microRNA 10a
1.01	MYRIP	myosin VIIA and Rab interacting protein
1.01	CASS4	Cas scaffolding protein family member 4
1.01	MIF	macrophage migration inhibitory factor (glycosylation-inhibiting factor)
1.01	LRBA	LPS-responsive vesicle trafficking. beach and anchor containing
1.01	COL12A1	collagen. type XII. alpha 1
1.01	MAK	male germ cell-associated kinase
1.01	ADCY5	adenylate cyclase 5
1.01	C1orf158	chromosome 1 open reading frame 158
1.01	SLC2A11	solute carrier family 2 (facilitated glucose transporter). member 11
1.01	DEXI	Dexi homolog (mouse)
1.01	ZNF679	zinc finger protein 679
1.01	VOPP1	vesicular. overexpressed in cancer. prosurvival protein 1
1.01	CDC14B	cell division cycle 14B
1.01	RXFP2	relaxin/insulin-like family peptide receptor 2
1.01	MTX2	metaxin 2
1.01	DMWD	dystrophia myotonica. WD repeat containing
1.01	DUS3L	dihydrouridine synthase 3-like
1.01	CRAMP1	cramped chromatin regulator homolog 1
1.01	RAB26	RAB26. member RAS oncogene family
1.01	DNAH8	dynein. axonemal. heavy chain 8
1.01	TSPY3; LOC728395	Homo sapiens testis specific protein. Y-linked 3. mRNA (cDNA clone MGC:103998 IMAGE:30915406). complete cds.; Homo sapiens similar to testis specific protein. Y-linked 1. mRNA (cDNA clone MGC:150413 IMAGE:40120433). complete cds.; Homo sapiens testis specific protein. Y-linked 3. mRNA (cDNA clone MGC:150414 IMAGE:40120435). complete cds.
1.01	LCE2D	late cornified envelope 2D

1.01	ATAD3B	ATPase family. AAA domain containing 3B
1.01	LOC388282; CTD-2600O9.1	uncharacterized LOC388282; novel transcript
1.01	COL6A2	Jeck2013 ANTISENSE. CDS. coding. INTERNAL. intronic. OVCODE. OVEXON best transcript NM_058175
1.01	TM4SF19-AS1	TM4SF19 antisense RNA 1
1.01	GCNT1	glucosaminyl (N-acetyl) transferase 1. core 2
1.01	SERPINB3	serpin peptidase inhibitor. clade B (ovalbumin). member 3
1.01	NUTM2G	NUT family member 2G
1.01	SCGB3A2	secretoglobin. family 3A. member 2
1.01	PLSCR5	phospholipid scramblase family. member 5
1.01	CUL2; RP11-297A16.2	Transcript Identified by AceView. Entrez Gene ID(s) 8453; novel transcript
1.01	SLC6A18	solute carrier family 6 (neutral amino acid transporter). member 18
1.01	MRGBP	MRG/MORF4L binding protein
1.01	ZFAND2B	zinc finger. AN1-type domain 2B
1.01	CFHR2	complement factor H-related 2
1.01	HINT1	histidine triad nucleotide binding protein 1
1.01	IL36A	interleukin 36. alpha
1.01	ACMSD	aminocarboxymuconate semialdehyde decarboxylase
1.01	ZNF83	zinc finger protein 83
1.01	NDUFAF7	NADH dehydrogenase (ubiquinone) complex I. assembly factor 7
1.01	FAM167A	family with sequence similarity 167. member A
1.01	SLC6A17	solute carrier family 6 (neutral amino acid transporter). member 17
1.01	MTM1	myotubularin 1
1.01	SMIM8	small integral membrane protein 8
1.01	PDCD7	programmed cell death 7
1.01	LCE6A	late cornified envelope 6A

1.01	SMIM15	small integral membrane protein 15
1.01	NGDN	neuroguidin. EIF4E binding protein
1.01	TRAPPC4	trafficking protein particle complex 4
1.01	PRMT8	protein arginine methyltransferase 8
1.01	CDV3	CDV3 homolog (mouse)
1.01	OR51L1	olfactory receptor. family 51. subfamily L. member 1
1.01	CSMD2	CUB and Sushi multiple domains 2
1.01	AP3D1	adaptor-related protein complex 3. delta 1 subunit
1.01	USP17L22	ubiquitin specific peptidase 17-like family member 22
1.01	STX11	syntaxin 11
1.01	EXOSC8	exosome component 8
1.01	MYSM1	Myb-like. SWIRM and MPN domains 1
1.01	TNN	tenascin N
1.01	LRIG1	
1.01	NEURL3	neuralized E3 ubiquitin protein ligase 3
1.01	IGSF22	immunoglobulin superfamily. member 22
1.01	CSTF2	cleavage stimulation factor. 3 pre-RNA. subunit 2
1.01	ARHGDIG	Rho GDP dissociation inhibitor (GDI) gamma
1.01	PDZRN3	PDZ domain containing ring finger 3
1.01	GTDC1	glycosyltransferase like domain containing 1
1.01	PKP1	plakophilin 1
1.01	CTNS	cystinosin. lysosomal cystine transporter
1.01	HDAC11	histone deacetylase 11
1.01	FBP1	fructose-1.6-bisphosphatase 1
1.01	C7orf62	chromosome 7 open reading frame 62
1.01	ETAA1	Ewing tumor-associated antigen 1

1.01	PARP3	poly(ADP-ribose) polymerase family member 3
1.01	STK4	serine/threonine kinase 4
1.01	ERICH1	glutamate rich 1
1.01	MUM1L1	melanoma associated antigen (mutated) 1-like 1
1.01	PSMB7	proteasome subunit beta 7
1.01	KCNJ9	potassium channel. inwardly rectifying subfamily J. member 9
1.01	PRKY	protein kinase. Y-linked. pseudogene
1.01	PSMB11	proteasome (prosome. macropain) subunit. beta type. 11
1.01	SMOC1	SPARC related modular calcium binding 1
1.01	USP29	ubiquitin specific peptidase 29
1.01	GRAMD4	GRAM domain containing 4
1.01	ASNSD1	asparagine synthetase domain containing 1
1.01	PHEX	phosphate regulating endopeptidase homolog. X-linked
1.01	RP11-732A19.2; TAF10	novel transcript. sense overlapping TAF10; Transcript Identified by AceView. Entrez Gene ID(s) 6881
1.01	DAZ2; DAZ4	deleted in azoospermia 2; deleted in azoospermia 4
1.01	B3GNT2	UDP-GlcNAc:betaGal beta-1.3-N-acetylglucosaminyltransferase 2
1.01	CIB4	calcium and integrin binding family member 4
1.01	FAM189A1	family with sequence similarity 189. member A1
1.01	GUCA1C	guanylate cyclase activator 1C
1.01	UBALD1	UBA-like domain containing 1
1.01	SOX14	SRY box 14
1.01	FRMPD3	FERM and PDZ domain containing 3
1.01	CCDC182	coiled-coil domain containing 182
1.01	POGZ	Transcript Identified by AceView. Entrez Gene ID(s) 23126
1.01	CAB39L	calcium binding protein 39-like

1.01	CWC25	CWC25 spliceosome-associated protein homolog
1.01	NBEA	neurobeachin
1.01	SURF4	surfeit 4
1.01	ZNF671	zinc finger protein 671
1.01	MYH7B	myosin. heavy chain 7B. cardiac muscle. beta
1.01	sept-04	septin 4
1.01	C16orf89	chromosome 16 open reading frame 89
1.01	RP5-1091N2.9; IL2RG	Transcript Identified by AceView. Entrez Gene ID(s) 3561; putative novel transcript
1.01	ENTHD2	ENTH domain containing 2
1.01	DNASE1L1	deoxyribonuclease I-like 1
1.01	PHF8	PHD finger protein 8
1.01	GABRA5	gamma-aminobutyric acid (GABA) A receptor. alpha 5
1.01	FMN1	formin 1
1.01	FSD2	fibronectin type III and SPRY domain containing 2
1.01	GPR42	G protein-coupled receptor 42 (gene/pseudogene)
1.01	LANCL1	LanC lantibiotic synthetase component C-like 1 (bacterial)
1.01	SLITRK2	SLIT and NTRK-like family. member 2
1.01	SLC25A3; SNORA53	solute carrier family 25 (mitochondrial carrier; phosphate carrier). member 3; small nucleolar RNA. H/ACA box 53
1.01	TRPC5	transient receptor potential cation channel. subfamily C. member 5
1.01	ROR2	receptor tyrosine kinase-like orphan receptor 2
1.01	LRTOMT	leucine rich transmembrane and O-methyltransferase domain containing
1.01	KHNYN	KH and NYN domain containing
1.01	ZNF775	zinc finger protein 775
1.01	SLC13A3; TP53RK	solute carrier family 13 (sodium-dependent dicarboxylate transporter). member 3; TP53 regulating kinase

1.01	UBE2O	ubiquitin-conjugating enzyme E2O
1.01	B3GALT2	UDP-Gal:betaGlcNAc beta 1.3-galactosyltransferase 2
1.01	PLAC8L1	PLAC8-like 1
1.01	ZNF320	zinc finger protein 320
1.01	KANSL1	KAT8 regulatory NSL complex subunit 1
1.01	FUT11	fucosyltransferase 11 (alpha (1.3) fucosyltransferase)
1.01	CDH19	cadherin 19. type 2
1.01	sept-12	septin 12
1.01	PSMG4	proteasome (prosome. macropain) assembly chaperone 4
1.01	DDX49	DEAD (Asp-Glu-Ala-Asp) box polypeptide 49
1.01	MSANTD4	Myb/SANT-like DNA-binding domain containing 4 with coiled-coils
1.01	PSMD13	proteasome 26S subunit. non-ATPase 13
1.01	RBM25	RNA binding motif protein 25
1.01	TJAP1	tight junction associated protein 1 (peripheral)
1.01	DNAJA3	DnaJ (Hsp40) homolog. subfamily A. member 3
1.01	RIMBP2	RIMS binding protein 2
1.01	MYH15	myosin. heavy chain 15
1.01	C22orf42	chromosome 22 open reading frame 42
1.01	ABHD10	abhydrolase domain containing 10
1.01	RGMB	repulsive guidance molecule family member b
1.01	HOXA13	homeobox A13
1.01	CLP1	cleavage and polyadenylation factor I subunit 1
1.01	UCN3	urocortin 3
1.01	CUX2	cut-like homeobox 2
1.01	SLC25A45	solute carrier family 25. member 45
1.01	KLHDC4	kelch domain containing 4

1.01	HNRNPD	heterogeneous nuclear ribonucleoprotein D
1.01	FAM71A	family with sequence similarity 71. member A
1.01	OR2J2	olfactory receptor. family 2. subfamily J. member 2
1.01	SLC4A1	solute carrier family 4 (anion exchanger). member 1 (Diego blood group)
1.01	NDUFC1	NADH dehydrogenase (ubiquinone) 1. subcomplex unknown. 1. 6kDa
1.01	RAD1	RAD1 checkpoint DNA exonuclease
1.01	PLCH2	phospholipase C. eta 2
1.01	KRTAP2-1	keratin associated protein 2-1
1.01	APOO	apolipoprotein O
1.01	DPH1; OVCA2	diphthamide biosynthesis 1; ovarian tumor suppressor candidate 2
1.01	COL17A1; MIR936	collagen. type XVII. alpha 1; microRNA 936
1.01	ALOX15B	arachidonate 15-lipoxygenase. type B
1.01	LONP1	Transcript Identified by AceView. Entrez Gene ID(s) 9361
1.01	LINGO4	leucine rich repeat and Ig domain containing 4
1.01	C17orf99	chromosome 17 open reading frame 99
1.01	SCYL2	SCY1-like. kinase-like 2
1.01	ZC4H2	zinc finger. C4H2 domain containing
1.01	C21orf59	chromosome 21 open reading frame 59
1.01	NUBPL	nucleotide binding protein-like
1.01	HSPB8	heat shock 22kDa protein 8
1.01	BTBD9	Memczak2013 ALT_ACCEPTOR. ALT_DONOR. coding. INTERNAL. intronic best transcript NM_001099272
1.01	TRAPPC4	trafficking protein particle complex 4
1.01	PBX2	pre-B-cell leukemia homeobox 2
1.01	LEUTX	leucine twenty homeobox
1.01	RPN2	ribophorin II

1.01	MRPL21	mitochondrial ribosomal protein L21
1.01	SPIN2B	spindlin family. member 2B
1.01	DCX	doublecortin
1.01	RGS11	regulator of G-protein signaling 11
1.01	ZNF641	zinc finger protein 641
1.01	HYOU1	hypoxia up-regulated 1
1.01	KCNK18	potassium channel. two pore domain subfamily K. member 18
1.01	EBF4	early B-cell factor 4
1.01	DEF6	DEF6 guanine nucleotide exchange factor
1.01	TRPC5OS	TRPC5 opposite strand
1.01	WDR5	WD repeat domain 5
1.01	CDY2A	chromodomain protein. Y-linked. 2A
1.01	DDC	dopa decarboxylase
1.01	MYH6	myosin. heavy chain 6. cardiac muscle. alpha
1.01	TANGO6	transport and golgi organization 6 homolog
1.01	OPN5	opsin 5
1.01	CHURC1-FNTB	CHURC1-FNTB readthrough
1.01	PCP4	Purkinje cell protein 4
1.01	PPWD1	peptidylprolyl isomerase domain and WD repeat containing 1
1.01	FAM173B	family with sequence similarity 173. member B
1.01	OR2AT4	olfactory receptor. family 2. subfamily AT. member 4
1.01	RP11-67L3.5; RP11-67L3.4; TMEM61	novel transcript; Transcript Identified by AceView. Entrez Gene ID(s) 199964
1.01	ISG20L2	interferon stimulated exonuclease gene 20kDa like 2
1.01	ESR1	estrogen receptor 1
1.01	MBD3	methyl-CpG binding domain protein 3

1.01	UBAP2; SNORD121A; SNORD121B	ubiquitin associated protein 2; small nucleolar RNA. C/D box 121A; small nucleolar RNA. C/D box 121B
1.01	TAAR2	trace amine associated receptor 2
1.01	ATP5J2-PTCD1; PTCD1	ATP5J2-PTCD1 readthrough; pentatricopeptide repeat domain 1
1.01	ARHGEF12	Rho guanine nucleotide exchange factor (GEF) 12
1.01	DIS3L	DIS3 like exosome 3-5 exoribonuclease
1.01	SLC5A3; MRPS6	solute carrier family 5 (sodium/myo-inositol cotransporter). member 3; mitochondrial ribosomal protein S6
1.01	TBL2	transducin (beta)-like 2
1.01	SLC25A31	solute carrier family 25 (mitochondrial carrier; adenine nucleotide translocator). member 31
1.01	TRAM2	translocation associated membrane protein 2
1.01	HN1L	hematological and neurological expressed 1-like
1.01	BACH2	BTB and CNC homology 1. basic leucine zipper transcription factor 2
1.01	PCDH15	protocadherin-related 15
1.01	IL10RA	interleukin 10 receptor. alpha
1.01	IQCF5	IQ motif containing F5
1.01	GEMIN4	gem nuclear organelle associated protein 4
1.01	B9D2	B9 protein domain 2
1.01	NRDE2	NRDE-2. necessary for RNA interference. domain containing
1.01	CASP3	caspase 3
1.01	OR5D18	olfactory receptor. family 5. subfamily D. member 18
1.01	SPDYA	speedy/RINGO cell cycle regulator family member A
1.01	EGR4	early growth response 4
1.01	NPTX1	neuronal pentraxin I
1.01	STXBP5L	syntaxin binding protein 5-like
1.01	ELF1	Transcript Identified by AceView. Entrez Gene ID(s) 1997; 100128628

1.01	MMP26	matrix metallopeptidase 26
1.01	SLC35F4	solute carrier family 35. member F4
1.01	OR4D10	olfactory receptor. family 4. subfamily D. member 10
1.01	PCYT1A	phosphate cytidylyltransferase 1. choline. alpha
1.01	KCNK1	Transcript Identified by AceView. Entrez Gene ID(s) 3775
1.01	NAALADL2	N-acetylated alpha-linked acidic dipeptidase-like 2
1.01	ZNF224	zinc finger protein 224
1.01	PPIL3	peptidylprolyl isomerase (cyclophilin)-like 3
1.01	OR2W3	olfactory receptor. family 2. subfamily W. member 3
1.01	EFS	embryonal Fyn-associated substrate
1.01	PADI6	peptidyl arginine deiminase. type VI
1.01	IPO9	importin 9
1.01	UBN1	ubinuclein 1
1.01	KIAA1143	KIAA1143
1.01	LSM12	LSM12 homolog
1.01	APOBEC3F	apolipoprotein B mRNA editing enzyme. catalytic polypeptide-like 3F
1.01	H3F3A; H3F3AP4	H3 histone. family 3A; H3 histone. family 3A. pseudogene 4
1.01	DPF2	D4. zinc and double PHD fingers family 2
1.01	BBS1	Bardet-Biedl syndrome 1
1.01	AAAS	achalasia. adrenocortical insufficiency. alacrimia
1.01	DUOXA1	dual oxidase maturation factor 1
1.01	ZNF32	zinc finger protein 32
1.01	RBM20	RNA binding motif protein 20
1.01	GP5	glycoprotein V (platelet)
1.01	KRT71	keratin 71. type II
1.01	ZSCAN25	zinc finger and SCAN domain containing 25

1.01	FOLR1	folate receptor 1 (adult)
1.01	RTL1	retrotransposon-like 1
1.01	AC008753.6; MYADM	Memczak2013 ANTISENSE. coding. INTERNAL. intronic best transcript NM_001020819; novel transcript. antisense to MYADM
1.01	PPP6R1	protein phosphatase 6. regulatory subunit 1
1.01	F8	coagulation factor VIII. procoagulant component
1.01	NANOS3	nanos homolog 3 (Drosophila)
1.01	PRR12	proline rich 12
1.01	PLEKHA4	pleckstrin homology domain containing. family A (phosphoinositide binding specific) member 4
1.01	SEMA7A	semaphorin 7A. GPI membrane anchor (John Milton Hagen blood group)
1.01	ZNF239	zinc finger protein 239
1.01	PHTF2	putative homeodomain transcription factor 2
1.01	SIX6	SIX homeobox 6
1.01	CCDC80; LINC01279	coiled-coil domain containing 80; long intergenic non-protein coding RNA 1279
1.01	ZNF678	Transcript Identified by AceView. Entrez Gene ID(s) 339500
1.01	RNF128	ring finger protein 128. E3 ubiquitin protein ligase
1.01	GLT8D2	glycosyltransferase 8 domain containing 2
1.01	MAP7D1	MAP7 domain containing 1
1.01	SPP2	secreted phosphoprotein 2
1.01	PPIAL4E	peptidylprolyl isomerase A (cyclophilin A)-like 4E
1.01	TRHDE	thyrotropin-releasing hormone degrading enzyme
1.01	MOAP1	modulator of apoptosis 1
1.01	OR4F6	olfactory receptor. family 4. subfamily F. member 6
1.01	RARS	arginyl-tRNA synthetase
1.01	ZBTB4	zinc finger and BTB domain containing 4

1.01	CIDEC	cell death-inducing DFFA-like effector c
1.01	PPP1R11	protein phosphatase 1. regulatory (inhibitor) subunit 11
1.01	GJB3	gap junction protein beta 3
1.01	ZNF831	zinc finger protein 831
1.01	OR4C5	olfactory receptor. family 4. subfamily C. member 5 (gene/pseudogene)
1.01	LRWD1; MIR4467; MIR5090	leucine-rich repeats and WD repeat domain containing 1; microRNA 4467; microRNA 5090
1.01	MC4R	melanocortin 4 receptor
1.01	DACH1	dachshund family transcription factor 1
1.01	POT1	protection of telomeres 1
1.01	TASP1	taspase 1
1.01	DNAJC21	DnaJ (Hsp40) homolog. subfamily C. member 21
1.01	STX7	syntaxin 7
1.01	RANBP10	RAN binding protein 10
1.01	PRR14L	proline rich 14-like
1.01	SPEF2	sperm flagellar 2
1.01	ROPN1	rhophilin associated tail protein 1
1.01	KRT80	keratin 80. type II
1.01	RBM38	RNA binding motif protein 38
1.01	DUSP22	dual specificity phosphatase 22
1.01	PANX3	pannexin 3
1.01	RGR	retinal G protein coupled receptor
1.01	OR56A3	olfactory receptor. family 56. subfamily A. member 3
1.01	CHGA	chromogranin A
1.01	KRT6A	keratin 6A. type II
1.01	PSMA8	proteasome subunit alpha 8

1.01	FOXK2	forkhead box K2
1.01	METAP1D	methionyl aminopeptidase type 1D (mitochondrial)
1.01	SIGLEC9	sialic acid binding Ig-like lectin 9
1.01	RSPO2	R-spondin 2
1.01	NAALADL2	N-acetylated alpha-linked acidic dipeptidase-like 2
1.01	CHRM1	cholinergic receptor. muscarinic 1
1.01	FLAD1	flavin adenine dinucleotide synthetase 1
1.01	TAPBPL	TAP binding protein-like
1.01	SERPINA2	serpin peptidase inhibitor. clade A (alpha-1 antiproteinase. antitrypsin). member 2 (gene/pseudogene)
1.01	NKD2	naked cuticle homolog 2 (Drosophila)
1.01	NPPC	natriuretic peptide C
1.01	KISS1	KiSS-1 metastasis-suppressor
1.01	TFAP2C	transcription factor AP-2 gamma (activating enhancer binding protein 2 gamma)
1.01	sept-09	septin 9
1.01	ATG9B	autophagy related 9B
1.01	PNPLA1	patatin-like phospholipase domain containing 1
1.01	ZNF23	zinc finger protein 23
1.01	RAB14	RAB14. member RAS oncogene family
1.01	FAM103A1	family with sequence similarity 103. member A1
1.01	HPS5	Hermansky-Pudlak syndrome 5
1.01	ZNF287	zinc finger protein 287
1.01	OR8K3	olfactory receptor. family 8. subfamily K. member 3 (gene/pseudogene)
1.01	EGLN3	egl-9 family hypoxia-inducible factor 3
1.01	MNDA	myeloid cell nuclear differentiation antigen
1.01	PRSS12	protease. serine. 12 (neurotrypsin. motopsin)

1.01	ST5	Transcript Identified by AceView. Entrez Gene ID(s) 6764
1.01	FGF5	fibroblast growth factor 5
1.01	AQP9	aquaporin 9
1.01	NECAP2	NECAP endocytosis associated 2
1.01	STK19	serine/threonine kinase 19
1.01	PIK3CG	phosphatidylinositol-4.5-bisphosphate 3-kinase. catalytic subunit gamma
1.01	PRR25	proline rich 25
1.01	FBXO36	F-box protein 36
1.01	UTS2R	urotensin 2 receptor
1.01	SYBU	syntabulin (syntaxin-interacting)
1.01	SAG	S-antigen; retina and pineal gland (arrestin)
1.01	ST3GAL4	ST3 beta-galactoside alpha-2.3-sialyltransferase 4
1.01	OR2M7	olfactory receptor. family 2. subfamily M. member 7
1.01	BCDIN3D	BCDIN3 domain containing
1.01	PTBP1; MIR4745	polypyrimidine tract binding protein 1; microRNA 4745
1.01	GPR62	G protein-coupled receptor 62
1.01	CYP4F11	cytochrome P450. family 4. subfamily F. polypeptide 11
1.01	SEPW1	selenoprotein W. 1
1.01	LRCH2	leucine-rich repeats and calponin homology (CH) domain containing 2
1.01	DYRK1B	dual specificity tyrosine-(Y)-phosphorylation regulated kinase 1B
1.01	CHL1	cell adhesion molecule L1-like
1.01	LDB2	LIM domain binding 2
1.01	FNBP4	formin binding protein 4
1.01	INADL	InaD-like (Drosophila)
1.01	NPHP3	nephronophthisis 3 (adolescent)
1.01	GPR158	G protein-coupled receptor 158

1.01	LINC01189	long intergenic non-protein coding RNA 1189
1.01	AP1G1	adaptor-related protein complex 1. gamma 1 subunit
1.01	CHRM5	cholinergic receptor. muscarinic 5
1.01	RHBDD2	rhomboid domain containing 2
1.01	FBL	fibrillarin
1.01	WDR49	WD repeat domain 49
1.01	RAD51B	Jeck2013 ALT_ACCEPTOR. ALT_DONOR. coding. INTERNAL. intronic best transcript NM_133509
1.01	KIF2B	kinesin family member 2B
1.01	GABRD	gamma-aminobutyric acid (GABA) A receptor. delta
1.01	EDF1	endothelial differentiation-related factor 1
1.01	SMAD5	SMAD family member 5
1.01	PPP1R36	protein phosphatase 1. regulatory subunit 36
1.01	PEX5L	Transcript Identified by AceView. Entrez Gene ID(s) 51555
1.01	TGFB3	transforming growth factor beta 3
1.01	RTN4R	reticulon 4 receptor
1.01	RABAC1	Rab acceptor 1 (prenylated)
1.01	ORAOV1	oral cancer overexpressed 1
1.01	ENKD1	enkurin domain containing 1
1.01	PHF13	PHD finger protein 13
1.01	OCSTAMP	osteoclast stimulatory transmembrane protein
1.01	CCNI2	cyclin I family. member 2
1.01	RTEL1; TNFRSF6B; RTEL1-TNFRSF6B	regulator of telomere elongation helicase 1; tumor necrosis factor receptor superfamily. member 6b. decoy; RTEL1-TNFRSF6B readthrough (NMD candidate)
1.01	MSX2	msh homeobox 2
1.01	CDIPT	CDP-diacylglycerol--inositol 3-phosphatidyltransferase

1.01	FAM181A	family with sequence similarity 181. member A
1.01	MFF	mitochondrial fission factor
1.01	LOC388813; bafor; AF165138.7	uncharacterized protein ENSP00000383407-like; Transcript Identified by AceView; HCG1818297; Protein LOC388813 [Source:UniProtKB/TrEMBL;Acc:A0A087WSY0]
1.01	CDCA5	Memczak2013 ANTISENSE. CDS. coding. INTERNAL. UTR3 best transcript NM_080668
1.01	PRAMEF25	PRAME family member 25
1.01	IAH1	isoamyl acetate-hydrolyzing esterase 1 homolog
1.01	DLGAP2	discs. large (Drosophila) homolog-associated protein 2
1.01	FLT3	fms-related tyrosine kinase 3
1.01	LINC00851	long intergenic non-protein coding RNA 851
1.01	CENPBD1	CENPB DNA-binding domain containing 1
1.01	ST6GALNAC2	ST6 (alpha-N-acetyl-neuraminyl-2.3-beta-galactosyl-1.3)-N-acetylgalactosaminide alpha-2.6-sialyltransferase 2
1.01	ANKRD34B	ankyrin repeat domain 34B
1.01	IQCA1	IQ motif containing with AAA domain 1
1.01	FAM169B	family with sequence similarity 169. member B
1.01	PITPNA	Memczak2013 ALT_ACCEPTOR. ALT_DONOR. coding. INTERNAL. intronic best transcript NM_006224
1.01	RASA3	RAS p21 protein activator 3
1.01	EXT2	exostosin glycosyltransferase 2
1.01	TBX6	T-box 6
1.01	OR10AG1	olfactory receptor. family 10. subfamily AG. member 1
1.01	C2orf48	chromosome 2 open reading frame 48
1.01	ANK3	Transcript Identified by AceView. Entrez Gene ID(s) 288
1.01	MED12	mediator complex subunit 12

1.01	BTN1A1	butyrophilin. subfamily 1. member A1
1.01	SNX2	sorting nexin 2
1.01	CHRDL2	chordin-like 2
1.01	PRAMEF18	PRAME family member 18
1.01	SPATA18	spermatogenesis associated 18
1.01	MLLT11	myeloid/lymphoid or mixed-lineage leukemia; translocated to. 11
1.01	PI4KA	phosphatidylinositol 4-kinase. catalytic. alpha
1.01	TNFSF12	tumor necrosis factor (ligand) superfamily. member 12
1.01	FOXD4L1	forkhead box D4-like 1
1.01	KRT17	keratin 17. type I
1.01	KHDC1	KH homology domain containing 1
1.01	RPUSD1	RNA pseudouridylate synthase domain containing 1
1.01	HSPA5	heat shock 70kDa protein 5 (glucose-regulated protein. 78kDa)
1.01	RAB39B	Transcript Identified by AceView. Entrez Gene ID(s) 116442
1.01	DDB1	damage-specific DNA binding protein 1
1.01	LOC100505549; RP11-35G9.3	uncharacterized LOC100505549; Salzman2013 ANNOTATED. CDS. coding. OVCODE. OVERLAPTX. OVEXON. UTR3. UTR5 best transcript NM_001242804; novel transcript. antisense to ATP8B1
1.01	ARHGAP31	Rho GTPase activating protein 31
1.01	LRRC58	leucine rich repeat containing 58
1.01	NOL3	nucleolar protein 3 (apoptosis repressor with CARD domain)
1.01	KRT40	keratin 40. type I
1.01	SLAMF9	SLAM family member 9
1.01	NFATC2IP; MIR4517	nuclear factor of activated T-cells. cytoplasmic. calcineurin-dependent 2 interacting protein; microRNA 4517
1.01	MROH1	maestro heat-like repeat family member 1
1.01	TMEM207	transmembrane protein 207

1.01	AGBL4	ATP/GTP binding protein-like 4
1.01	IL25	interleukin 25
1.01	ANO8	anoctamin 8
1.01	FAM153C	family with sequence similarity 153. member C. pseudogene
1.01	E4F1	E4F transcription factor 1
1.01	LOC101928034; S100A3	uncharacterized LOC101928034; Salzman2013 ANTISENSE. CDS. coding. INTERNAL. intronic. OVCODE. OVEXON. UTR5 best transcript NM_002960
1.01	GDAP2	ganglioside induced differentiation associated protein 2
1.01	MRPS22	mitochondrial ribosomal protein S22
1.01	LINC01359	long intergenic non-protein coding RNA 1359
1.01	SLC28A1	solute carrier family 28 (concentrative nucleoside transporter). member 1
1.01	SAA2; SAA2-SAA4; SAA4	serum amyloid A2; SAA2-SAA4 readthrough; serum amyloid A4. constitutive
1.01	ALDH1A3	aldehyde dehydrogenase 1 family. member A3
1.01	KIF5B	kinesin family member 5B
1.01	RAB35	RAB35. member RAS oncogene family
1.01	RAB11FIP5	RAB11 family interacting protein 5 (class I)
1.01	LRRTM2	leucine rich repeat transmembrane neuronal 2
1.01	AP2M1	adaptor-related protein complex 2. mu 1 subunit
1.01	GPR144; ADGRD2	Transcript Identified by AceView. Entrez Gene ID(s) 347088; adhesion G protein-coupled receptor D2 [Source:HGNC Symbol;Acc:HGNC:18651]
1.01	LIPN	lipase. family member N
1.01	CLEC18C	C-type lectin domain family 18. member C
1.01	MRPL20	mitochondrial ribosomal protein L20
1.01	TMEM123	transmembrane protein 123
1.01	NDN	necdin. MAGE family member
1.01	FPGT-TNNI3K; FPGT	FPGT-TNNI3K readthrough; fucose-1-phosphate guanylyltransferase
1.01	ZNF414	zinc finger protein 414

1.01	PHLDB3	pleckstrin homology-like domain. family B. member 3
1.01	DYRK1B	dual specificity tyrosine-(Y)-phosphorylation regulated kinase 1B
1.01	SOX18	SRY box 18
1.01	DPCR1	diffuse panbronchiolitis critical region 1
1.01	SLC27A6	solute carrier family 27 (fatty acid transporter). member 6
1.01	KLHL31	kelch-like family member 31
1.01	NDUFA10	NADH dehydrogenase (ubiquinone) 1 alpha subcomplex. 10. 42kDa
1.01	INTS4	integrator complex subunit 4
1.01	MMP13	matrix metallopeptidase 13
1.01	CFHR1	complement factor H-related 1
1.01	INSIG1	insulin induced gene 1
1.01	LOC401357; RP13-580B18.4	uncharacterized LOC401357; novel piRNA host transcript
1.01	OR5V1	olfactory receptor. family 5. subfamily V. member 1
1.01	SPPL3	signal peptide peptidase like 3
1.01	S100A7L2	S100 calcium binding protein A7 like 2
1.01	NTN5	netrin 5
1.01	HSPB7	heat shock 27kDa protein family. member 7 (cardiovascular)
1.01	PPCS; CCDC30	phosphopantothenoylcysteine synthetase; coiled-coil domain containing 30
1.01	CYP20A1	cytochrome P450. family 20. subfamily A. polypeptide 1
1.01	CD72	CD72 molecule
1.01	CKAP4	cytoskeleton-associated protein 4
1.01	NKD1	naked cuticle homolog 1 (Drosophila)
1.01	P4HTM	prolyl 4-hydroxylase. transmembrane (endoplasmic reticulum)
1.01	PSG4	pregnancy specific beta-1-glycoprotein 4
1.01	SRCIN1	SRC kinase signaling inhibitor 1
1.01	INTS9	integrator complex subunit 9

1.01	CRYAB	crystallin alpha B
1.01	LYPD5	LY6/PLAUR domain containing 5
1.01	PEBP4	phosphatidylethanolamine-binding protein 4
1.01	C1orf185	chromosome 1 open reading frame 185
1.01	LMLN	leishmanolysin-like (metallopeptidase M8 family)
1.01	ALPP	alkaline phosphatase. placental
1.01	SLC2A5	solute carrier family 2 (facilitated glucose/fructose transporter). member 5
1.01	CYP4X1	cytochrome P450. family 4. subfamily X. polypeptide 1
1.01	LCK	LCK proto-oncogene. Src family tyrosine kinase
1.01	RGS18	regulator of G-protein signaling 18
1.01	ZNF550	zinc finger protein 550
1.01	NUP43	nucleoporin 43kDa
1.01	MTA2	metastasis associated 1 family member 2
1.01	LEF1	lymphoid enhancer-binding factor 1
1.01	ARVCF	armadillo repeat gene deleted in velocardiofacial syndrome
1.01	MTF1	metal-regulatory transcription factor 1
1.01	C6orf229	chromosome 6 open reading frame 229
1.01	NEFM	neurofilament. medium polypeptide
1.01	KLHL41	kelch-like family member 41
1.01	RP11-93O14.2; VPS35	novel transcript; Transcript Identified by AceView. Entrez Gene ID(s) 55737
1.01	TBC1D10B	TBC1 domain family. member 10B
1.01	C17orf82	chromosome 17 open reading frame 82
1.01	TRGJ1; TRGC2; TRGJ2; TRGV9; TRGC1; TRGJP; TRGJP2	T cell receptor gamma joining 1; T cell receptor gamma constant 2; T cell receptor gamma joining 2; T cell receptor gamma variable 9; T cell receptor gamma constant 1; T cell receptor gamma joining P; T cell receptor gamma joining P2
1.01	DGKD	diacylglycerol kinase. delta 130kDa

1.01	SLC4A3	solute carrier family 4 (anion exchanger). member 3
1.01	KLK3	kallikrein related peptidase 3
1.00	MROH7; TTC4; MROH7-TTC4	maestro heat-like repeat family member 7; tetratricopeptide repeat domain 4; MROH7-TTC4 readthrough (NMD candidate)
1.00	FPR1	formyl peptide receptor 1
1.00	ALKBH2	alkB homolog 2. alpha-ketoglutarate-dependent dioxygenase
1.00	ABCG4	ATP binding cassette subfamily G member 4
1.00	CRB1	crumbs family member 1. photoreceptor morphogenesis associated
1.00	LAIR1	leukocyte-associated immunoglobulin-like receptor 1
1.00	FLG2	filaggrin family member 2
1.00	MRFAP1L1	Morf4 family associated protein 1-like 1
1.00	SNUPN	snurportin 1
1.00	HSD11B1	hydroxysteroid (11-beta) dehydrogenase 1
1.00	DDX26B	DEAD/H (Asp-Glu-Ala-Asp/His) box polypeptide 26B
1.00	SEMA3A	Transcript Identified by AceView. Entrez Gene ID(s) 10371
1.00	LRRTM1	leucine rich repeat transmembrane neuronal 1
1.00	FAM162A	family with sequence similarity 162. member A
1.00	OR4D9	olfactory receptor. family 4. subfamily D. member 9
1.00	RPRML	reprimo-like
1.00	FBL	fibrillarin
1.00	LRRC40	leucine rich repeat containing 40
1.00	PEX2	peroxisomal biogenesis factor 2
1.00	STX12	syntaxin 12
1.00	BSND	barttin CLCNK-type chloride channel accessory beta subunit
1.00	NCALD	neurocalcin delta
1.00	FABP12	fatty acid binding protein 12

1.00	SLC25A35	solute carrier family 25. member 35
1.00	IFNA14	interferon. alpha 14
1.00	ABCD3	Transcript Identified by AceView. Entrez Gene ID(s) 5825
1.00	DDX51	DEAD (Asp-Glu-Ala-Asp) box polypeptide 51
1.00	MZF1	myeloid zinc finger 1
1.00	IGSF9B	immunoglobulin superfamily. member 9B
1.00	ZNF296	zinc finger protein 296
1.00	NFKB1	nuclear factor of kappa light polypeptide gene enhancer in B-cells 1
1.00	NELFB	negative elongation factor complex member B
1.00	ZDHHC3	zinc finger. DHHC-type containing 3
1.00	BBOX1	butyrobetaine (gamma). 2-oxoglutarate dioxygenase (gamma-butyrobetaine hydroxylase) 1
1.00	HNRNPCL3	heterogeneous nuclear ribonucleoprotein C-like 3 [Source:HGNC Symbol;Acc:HGNC:51235]
1.00	MDM1	Transcript Identified by AceView. Entrez Gene ID(s) 56890
1.00	HTR3D	5-hydroxytryptamine (serotonin) receptor 3D. ionotropic
1.00	LOC100129083; CTD-2616J11.2	uncharacterized LOC100129083; Transcript Identified by AceView. Entrez Gene ID(s) 100129083; novel transcript. antisense to SIGLEC10
1.00	NOTCH4	notch 4
1.00	MRGPRX3	MAS-related GPR. member X3
1.00	RGPD3	RANBP2-like and GRIP domain containing 3
1.00	LRRC9	leucine rich repeat containing 9
1.00	EQTN	equatorin. sperm acrosome associated
1.00	SALL4	spalt-like transcription factor 4
1.00	PLCL1	phospholipase C-like 1
1.00	MB	myoglobin
1.00	NMU	neuromedin U

1.00	TCHH	trichohyalin
1.00	NHEJ1	nonhomologous end-joining factor 1
1.00	AADACL2	arylacetamide deacetylase-like 2
1.00	JPH3	junctophilin 3
1.00	ZNF469	zinc finger protein 469
1.00	VSIG1	V-set and immunoglobulin domain containing 1
1.00	SERPINB10	serpin peptidase inhibitor. clade B (ovalbumin). member 10
1.00	ATPAF2	ATP synthase mitochondrial F1 complex assembly factor 2
1.00	PRIMA1	proline rich membrane anchor 1
1.00	GSS	glutathione synthetase
1.00	CNBD1	cyclic nucleotide binding domain containing 1
1.00	NDUFA6	NADH dehydrogenase (ubiquinone) 1 alpha subcomplex. 6. 14kDa
1.00	MYRF	myelin regulatory factor
1.00	PNLDC1	poly(A)-specific ribonuclease (PARN)-like domain containing 1
1.00	LINGO1	leucine rich repeat and Ig domain containing 1
1.00	TP53TG3C	TP53 target 3C
1.00	CHRNB4	cholinergic receptor. nicotinic beta 4
1.00	PTPN23	Jeck2013 ANTISENSE. CDS. coding. INTERNAL. intronic. OVCODE. OVEXON best transcript NM_015466
1.00	CALML3	calmodulin-like 3
1.00	CSF2RA	colony stimulating factor 2 receptor. alpha. low-affinity (granulocyte-macrophage)
1.00	NCR2	natural cytotoxicity triggering receptor 2
1.00	CCNG1	cyclin G1
1.00	COPS3	COP9 signalosome subunit 3
1.00	DTHD1	death domain containing 1
1.00	CD200R1	CD200 receptor 1

1.00	ZNFX1	zinc finger. NFX1-type containing 1
1.00	TIPRL	TOR signaling pathway regulator
1.00	CRABP1	cellular retinoic acid binding protein 1
1.00	SLCO5A1	solute carrier organic anion transporter family. member 5A1
1.00	OR2T10	olfactory receptor. family 2. subfamily T. member 10
1.00	PLB1	phospholipase B1
1.00	BCHE	butyrylcholinesterase
1.00	FBXO28	F-box protein 28
1.00	SYT10	synaptotagmin X
1.00	LZTS1	leucine zipper. putative tumor suppressor 1
1.00	MED9	mediator complex subunit 9
1.00	DGCR8	Zhang2013 ALT_ACCEPTOR. ALT_DONOR. coding. INTERNAL. intronic best transcript NM_022720
1.00	SLC36A4	solute carrier family 36 (proton/amino acid symporter). member 4
1.00	GRAP2	GRB2-related adaptor protein 2
1.00	FAM103A1	Homo sapiens family with sequence similarity 103. member A1. mRNA (cDNA clone MGC:2560 IMAGE:2989772). complete cds.; Homo sapiens family with sequence similarity 103. member A1. mRNA (cDNA clone MGC:18029 IMAGE:3924570). complete cds.; Homo sapiens family with sequence similarity 103. member A1. mRNA (cDNA clone MGC:102778 IMAGE:5578103). complete cds.
1.00	PTPRQ	protein tyrosine phosphatase. receptor type. Q
1.00	HYOU1	hypoxia up-regulated 1
1.00	VCX2	variable charge. X-linked 2
1.00	LMO3	LIM domain only 3 (rhombotin-like 2)
1.00	SRSF9	serine/arginine-rich splicing factor 9
1.00	KRTAP20-1	keratin associated protein 20-1
1.00	DHRS4	dehydrogenase/reductase (SDR family) member 4

1.00	FABP5	fatty acid binding protein 5 (psoriasis-associated)
1.00	IKBKAP	inhibitor of kappa light polypeptide gene enhancer in B-cells. kinase complex-associated protein
1.00	OR6C3	olfactory receptor. family 6. subfamily C. member 3
1.00	CNDP2	CNDP dipeptidase 2 (metallopeptidase M20 family)
1.00	PPIL4	peptidylprolyl isomerase (cyclophilin)-like 4
1.00	TSEN34	TSEN34 tRNA splicing endonuclease subunit
1.00	CTSB	Jeck2013 ALT_DONOR. coding. INTERNAL. intronic best transcript NM_147780
1.00	MAP1LC3B2	microtubule-associated protein 1 light chain 3 beta 2
1.00	ZSCAN5A	zinc finger and SCAN domain containing 5A
1.00	GTF2E1	general transcription factor IIE subunit 1
1.00	TOMM6; PRICKLE4	translocase of outer mitochondrial membrane 6 homolog (yeast); prickle homolog 4
1.00	RNF19B	Memczak2013 ANTISENSE. CDS. coding. INTERNAL best transcript NM_153341
1.00	POLR2I	polymerase (RNA) II (DNA directed) polypeptide I. 14.5kDa
1.00	MTSS1L	metastasis suppressor 1-like
1.00	LOC101928841; RP11-102K13.5	collagen alpha-1(II) chain-like; novel transcript
1.00	RBMX; SNORD61	RNA binding motif protein. X-linked; small nucleolar RNA. C/D box 61
1.00	TRAK2	trafficking protein. kinesin binding 2
1.00	KLRF1	killer cell lectin-like receptor subfamily F. member 1
1.00	FAM213B	family with sequence similarity 213. member B
1.00	SRMS	src-related kinase lacking C-terminal regulatory tyrosine and N-terminal myristylation sites
1.00	PNLIP	pancreatic lipase
1.00	KAT2B	Transcript Identified by AceView. Entrez Gene ID(s) 8850
1.00	TPD52L3	tumor protein D52-like 3

1.00	CDY1	chromodomain protein. Y-linked. 1
1.00	FNDC4	fibronectin type III domain containing 4
1.00	PPP1R1B	protein phosphatase 1. regulatory (inhibitor) subunit 1B
1.00	NRSN2-AS1	NRSN2 antisense RNA 1
1.00	PSCA	prostate stem cell antigen
1.00	DMPK	dystrophia myotonica-protein kinase
1.00	LTN1	listerin E3 ubiquitin protein ligase 1
1.00	DSCR10	Down syndrome critical region 10 (non-protein coding)
1.00	LOC102724238; LOC554249	uncharacterized LOC102724238; uncharacterized LOC554249
1.00	FAM49B	family with sequence similarity 49. member B
1.00	VCAN	versican
1.00	GXYLT1	glucoside xylosyltransferase 1
1.00	MLLT10	myeloid/lymphoid or mixed-lineage leukemia; translocated to. 10
1.00	SLC25A26	solute carrier family 25 (S-adenosylmethionine carrier). member 26
1.00	CCSER2	coiled-coil serine rich protein 2
1.00	SLC30A5	solute carrier family 30 (zinc transporter). member 5
1.00	DUSP11	dual specificity phosphatase 11
1.00	IL13	interleukin 13
1.00	NPTX2	neuronal pentraxin II
1.00	OR1N2	olfactory receptor. family 1. subfamily N. member 2
1.00	ACTR3B	ARP3 actin-related protein 3 homolog B (yeast)
1.00	MYF6	myogenic factor 6 (herculin)
1.00	SLC27A5	solute carrier family 27 (fatty acid transporter). member 5
1.00	SCIMP	SLP adaptor and CSK interacting membrane protein
1.00	CCL27	chemokine (C-C motif) ligand 27
1.00	KRT7	keratin 7. type II

1.00	HYOU1	hypoxia up-regulated 1
1.00	FZD3	frizzled class receptor 3
1.00	PRPH2	peripherin 2 (retinal degeneration. slow)
1.00	KCNK7	potassium channel. two pore domain subfamily K. member 7
1.00	CCDC17	coiled-coil domain containing 17
1.00	METTL21C	methyltransferase like 21C
1.00	PLEKHA3	pleckstrin homology domain containing. family A (phosphoinositide binding specific) member 3
1.00	PRPF3	pre-mRNA processing factor 3
1.00	TMEM206	transmembrane protein 206
1.00	PODN	Transcript Identified by AceView. Entrez Gene ID(s) 127435
1.00	FABP3	fatty acid binding protein 3. muscle and heart
1.00	RPS6KA1	ribosomal protein S6 kinase. 90kDa. polypeptide 1
1.00	SLC52A3	solute carrier family 52 (riboflavin transporter). member 3
1.00	INSC	inscuteable homolog (Drosophila)
1.00	CLASP2	Transcript Identified by AceView. Entrez Gene ID(s) 23122
1.00	GP1BA	glycoprotein Ib (platelet). alpha polypeptide
1.00	RIPPLY3	ripply transcriptional repressor 3
1.00	OR52E6	olfactory receptor. family 52. subfamily E. member 6
1.00	CFAP77	cilia and flagella associated protein 77
1.00	MAU2	MAU2 sister chromatid cohesion factor
1.00	HAUS3; POLN	HAUS augmin like complex subunit 3; polymerase (DNA directed) nu
1.00	ADGRG1	adhesion G protein-coupled receptor G1
1.00	CLK2	CDC like kinase 2
1.00	GMEB1	glucocorticoid modulatory element binding protein 1
1.00	CSTB	cystatin B (stefin B)

1.00	C6orf99	chromosome 6 open reading frame 99
1.00	PRAMEF9	
1.00	CASP4	caspase 4
1.00	EXO5	exonuclease 5
1.00	PRMT9	protein arginine methyltransferase 9
1.00	CTAGE5	CTAGE family. member 5
1.00	FMN1	formin 1
1.00	FMO4	flavin containing monooxygenase 4
1.00	FRAT2	frequently rearranged in advanced T-cell lymphomas 2
1.00	RBFOX1	Memczak2013 ALT_ACCEPTOR. ALT_DONOR. coding. INTERNAL. intronic best transcript NM_001142333
1.00	RNF223	ring finger protein 223
1.00	CRELD1	cysteine rich with EGF-like domains 1
1.00	C6orf62	chromosome 6 open reading frame 62
1.00	OR5F1	olfactory receptor. family 5. subfamily F. member 1
1.00	OR4C11	olfactory receptor. family 4. subfamily C. member 11
1.00	FMN1	Transcript Identified by AceView. Entrez Gene ID(s) 342184
1.00	TRIM64B	tripartite motif containing 64B
1.00	STMN2	stathmin 2
1.00	SPTBN5	spectrin. beta. non-erythrocytic 5
1.00	NXF1	nuclear RNA export factor 1
1.00	SLC9C1	solute carrier family 9. subfamily C (Na+-transporting carboxylic acid decarboxylase). member 1
1.00	EML4	echinoderm microtubule associated protein like 4
1.00	ADAM32	ADAM metallopeptidase domain 32
1.00	NLRP1	NLR family. pyrin domain containing 1
1.00	PDHA2	pyruvate dehydrogenase (lipoamide) alpha 2

1.00	LDHAL6B	lactate dehydrogenase A-like 6B
1.00	LNPEP	leucyl/cystinyl aminopeptidase
1.00	FARSA	phenylalanyl-tRNA synthetase alpha subunit
1.00	FAM180A	family with sequence similarity 180. member A
1.00	SULT6B1	sulfotransferase family 6B member 1
1.00	CNST	consortin. connexin sorting protein
1.00	CDC42SE2	CDC42 small effector 2
1.00	SEC62	SEC62 homolog. preprotein translocation factor
1.00	MYL4	myosin light chain 4
1.00	MAGEA3	MAGE family member A3
1.00	SHROOM4	shroom family member 4
1.00	OR1D2	olfactory receptor. family 1. subfamily D. member 2
1.00	EIF3F	Eukaryotic translation initiation factor 3 subunit F [Source:UniProtKB/Swiss-Prot;Acc:O00303]
1.00	TSPAN4	tetraspanin 4
1.00	ZNF256	zinc finger protein 256
1.00	AMY1A; AMY1C	amylase. alpha 1A (salivary); amylase. alpha 1C (salivary)
1.00	FBL	fibrillarin
1.00	IMPG1	interphotoreceptor matrix proteoglycan 1
1.00	RXFP1	relaxin/insulin-like family peptide receptor 1
1.00	CCL5	chemokine (C-C motif) ligand 5
1.00	DPEP2	dipeptidase 2
1.00	OPN1MW2	opsin 1 (cone pigments). medium-wave-sensitive 2
1.00	IRF5	interferon regulatory factor 5
1.00	R3HDM1	R3H domain containing 1
1.00	RGS1	regulator of G-protein signaling 1

1.00	ATXN7L2	ataxin 7-like 2
1.00	HNRNPH3	heterogeneous nuclear ribonucleoprotein H3 (2H9)
1.00	ZBTB33	zinc finger and BTB domain containing 33
1.00	WLS	Jeck2013 ALT_ACCEPTOR. ALT_DONOR. coding. INTERNAL. intronic. OVERLAPTX best transcript NM_024911
1.00	C11orf42	chromosome 11 open reading frame 42
1.00	RAD51D	RAD51 paralog D
1.00	GUSB	glucuronidase. beta
1.00	ZPLD1	zona pellucida-like domain containing 1
1.00	MUC19	mucin 19. oligomeric
1.00	HYOU1	hypoxia up-regulated 1
1.00	NT5DC3	5-nucleotidase domain containing 3
1.00	CREB3L1	cAMP responsive element binding protein 3-like 1
1.00	CCDC85B	coiled-coil domain containing 85B
1.00	NDRG2; MIR6717	NDRG family member 2; microRNA 6717
1.00	SYCP1	synaptonemal complex protein 1
1.00	TMEM257	transmembrane protein 257
1.00	GATAD1	GATA zinc finger domain containing 1
1.00	DGKD	diacylglycerol kinase. delta 130kDa
1.00	CCDC174	coiled-coil domain containing 174
1.00	COG3	component of oligomeric golgi complex 3
1.00	IL26	interleukin 26
1.00	AWAT1	acyl-CoA wax alcohol acyltransferase 1
1.00	CYP2A7	cytochrome P450. family 2. subfamily A. polypeptide 7
1.00	TANGO2	transport and golgi organization 2 homolog
1.00	PDHA1	pyruvate dehydrogenase (lipoamide) alpha 1

1.00	NUTM1	NUT midline carcinoma. family member 1
1.00	GMDS	GDP-mannose 4.6-dehydratase
1.00	TRUB1	TruB pseudouridine (psi) synthase family member 1
1.00	SEC14L3	SEC14-like lipid binding 3
1.00	NUP160	nucleoporin 160kDa
1.00	PPP1CB	protein phosphatase 1. catalytic subunit. beta isozyme
1.00	VWA9	von Willebrand factor A domain containing 9
1.00	HIC2	hypermethylated in cancer 2
1.00	INPP5D	inositol polyphosphate-5-phosphatase D
1.00	C1orf68	chromosome 1 open reading frame 68
1.00	SLC39A6	solute carrier family 39 (zinc transporter). member 6
1.00	CCER1	coiled-coil glutamate rich protein 1
1.00	ST8SIA4	ST8 alpha-N-acetyl-neuraminide alpha-2.8-sialyltransferase 4
1.00	SIGLEC14; SIGLEC5	sialic acid binding Ig-like lectin 14; sialic acid binding Ig-like lectin 5
1.00	PHF20L1	Transcript Identified by AceView. Entrez Gene ID(s) 51105
1.00	ADA	adenosine deaminase
1.00	HDAC9	histone deacetylase 9
1.00	ST8SIA1	ST8 alpha-N-acetyl-neuraminide alpha-2.8-sialyltransferase 1
1.00	EXD1	exonuclease 3-5 domain containing 1
1.00	HEXDC	hexosaminidase (glycosyl hydrolase family 20. catalytic domain) containing
1.00	C9orf170	chromosome 9 open reading frame 170
1.00	SFTPA2; SFTPA1	surfactant protein A2; surfactant protein A1
1.00	TMEM165	transmembrane protein 165
1.00	KANSL3	KAT8 regulatory NSL complex subunit 3
1.00	LNPEP	Memczak2013 ANTISENSE. coding. INTERNAL. UTR3 best transcript NM_175920

1.00	MLX	MLX. MAX dimerization protein
1.00	CHST7	carbohydrate (N-acetylglucosamine 6-O) sulfotransferase 7
1.00	MMP9	matrix metallopeptidase 9
1.00	STAB2	stabilin 2
1.00	LOC100130451; AC079610.2; RP11-105N14.2	uncharacterized LOC100130451; novel transcript (LOC100130451); novel transcript
1.00	SNRNP70	small nuclear ribonucleoprotein. U1 70kDa subunit
1.00	DGAT2	diacylglycerol O-acyltransferase 2
1.00	CCDC127	coiled-coil domain containing 127
1.00	DCD	dermcidin
1.00	NRK	Nik related kinase
1.00	BCL2L2-PABPN1	BCL2L2-PABPN1 readthrough
1.00	AXIN1	axin 1
1.00	PHF3	PHD finger protein 3
1.00	TBC1D28	TBC1 domain family. member 28
1.00	ZGPAT; LIME1	zinc finger. CCCH-type with G-patch domain; Lck interacting transmembrane adaptor 1
1.00	AGGF1	angiogenic factor with G-patch and FHA domains 1
1.00	C2orf61	chromosome 2 open reading frame 61
1.00	ZFP28	ZFP28 zinc finger protein
1.00	HAS1	hyaluronan synthase 1
1.00	CCDC38	coiled-coil domain containing 38
1.00	LMBR1	Transcript Identified by AceView. Entrez Gene ID(s) 64327
1.00	CDK12	cyclin-dependent kinase 12
1.00	EDEM1	ER degradation enhancer. mannosidase alpha-like 1
1.00	ATXN7L3B	ataxin 7-like 3B
1.00	MALL	mal. T-cell differentiation protein-like

1.00	C10orf131	chromosome 10 open reading frame 131
1.00	ZNF443	zinc finger protein 443
1.00	SNRPB	small nuclear ribonucleoprotein polypeptides B and B1
1.00	CCDC27	coiled-coil domain containing 27
1.00	NOMO1	NODAL modulator 1
1.00	LACTB	lactamase. beta
1.00	PARN	poly(A)-specific ribonuclease
1.00	LRRC32	leucine rich repeat containing 32
1.00	C1QTNF6	C1q and tumor necrosis factor related protein 6
1.00	TOPORS	topoisomerase I binding. arginine/serine-rich. E3 ubiquitin protein ligase
1.00	ZCCHC9	zinc finger. CCHC domain containing 9
1.00	CLEC6A	C-type lectin domain family 6. member A
1.00	CFI	complement factor I
1.00	ANKRD18B	ankyrin repeat domain 18B
1.00	LHFP	lipoma HMGIC fusion partner
1.00	KCTD18	potassium channel tetramerization domain containing 18
1.00	MGC57346-CRHR1; RP11-105N13.4	Homo sapiens MGC57346-CRHR1 readthrough (MGC57346-CRHR1). transcript variant 5. mRNA.; Homo sapiens MGC57346-CRHR1 readthrough (MGC57346-CRHR1). transcript variant 6. mRNA.; novel transcript
1.00	ST6GALNAC1	ST6 (alpha-N-acetyl-neuraminyl-2.3-beta-galactosyl-1.3)-N-acetylgalactosaminide alpha-2.6-sialyltransferase 1
1.00	FAM153A	family with sequence similarity 153. member A
1.00	LRRN2	leucine rich repeat neuronal 2
1.00	LRRC31	leucine rich repeat containing 31
1.00	KRTAP2-2	keratin associated protein 2-2
1.00	APBB1IP	amyloid beta (A4) precursor protein-binding. family B. member 1 interacting protein

1.00	SLC39A12	solute carrier family 39 (zinc transporter). member 12
1.00	FOLR2	folate receptor 2 (fetal)
1.00	PRSS16	protease. serine. 16 (thymus)
1.00	OR1F1	olfactory receptor. family 1. subfamily F. member 1
1.00	PAPD7	PAP associated domain containing 7
1.00	MBD4	methyl-CpG binding domain 4 DNA glycosylase
1.00	GNG3	guanine nucleotide binding protein (G protein). gamma 3
1.00	LOC100506127; RP11-111M22.2; LOC387790	putative uncharacterized protein FLJ37770-like; Transcript Identified by AceView. Entrez Gene ID(s) 387790; novel transcript
1.00	OFD1	oral-facial-digital syndrome 1
1.00	FDXACB1	ferredoxin-fold anticodon binding domain containing 1
1.00	CHAMP1	chromosome alignment maintaining phosphoprotein 1
1.00	ZNF845	zinc finger protein 845
1.00	FBXO40	F-box protein 40
1.00	FGF1	fibroblast growth factor 1 (acidic)
1.00	LHX3	LIM homeobox 3
1.00	FKBP14	FK506 binding protein 14
1.00	CLRN2	clarin 2
1.00	ZNF57	zinc finger protein 57
1.00	PPP1R16B	Transcript Identified by AceView. Entrez Gene ID(s) 26051
1.00	HMBS	hydroxymethylbilane synthase
1.00	GSTA1	glutathione S-transferase alpha 1
1.00	IL4	interleukin 4
1.00	FAM83E	family with sequence similarity 83. member E
1.00	UNC5C	unc-5 netrin receptor C
1.00	MAP3K7CL	MAP3K7 C-terminal like

1.00	TMC2	transmembrane channel like 2
1.00	GPR173	G protein-coupled receptor 173
1.00	C9orf172	chromosome 9 open reading frame 172
1.00	IRF6	interferon regulatory factor 6
1.00	USP17L1	ubiquitin specific peptidase 17-like family member 1
1.00	PIK3CD-AS2	PIK3CD antisense RNA 2
1.00	PRKG2	protein kinase. cGMP-dependent. type II
1.00	MOB2	MOB kinase activator 2
1.00	FBXL5	F-box and leucine-rich repeat protein 5
1.00	RPL7A	ribosomal protein L7a
1.00	DRC3	dynein regulatory complex subunit 3
1.00	ZNF320	zinc finger protein 320
1.00	HHAT	hedgehog acyltransferase
1.00	RARS2	arginyl-tRNA synthetase 2. mitochondrial
1.00	PRSS36	protease. serine 36
1.00	USP2	ubiquitin specific peptidase 2
1.00	SELM	selenoprotein M; selenoprotein M [Source:EntrezGene;Acc:140606]; Salzman2013 ANNOTATED. CDS. coding. OVCODE. OVERLAPTX. OVEXON. UTR3 best transcript NM_080430; Salzman2013 ANNOTATED. CDS. coding. OVCODE. OVERLAPTX. OVEXON. UTR3. UTR5 best transcript NM_080430; Transcript Identified by AceView. Entrez Gene ID(s) 140606
1.00	FAXC	failed axon connections homolog
1.00	SMG5	SMG5 nonsense mediated mRNA decay factor
1.00	MRM1	mitochondrial rRNA methyltransferase 1
1.00	GUF1	GUF1 homolog. GTPase
1.00	C10orf90	chromosome 10 open reading frame 90
1.00	TMBIM6	transmembrane BAX inhibitor motif containing 6

1.00	DNAI2	dynein. axonemal. intermediate chain 2
1.00	HNMT	histamine N-methyltransferase
1.00	HIPK1	homeodomain interacting protein kinase 1
1.00	KIAA0907; SCARNA4	KIAA0907; small Cajal body-specific RNA 4
1.00	LILRB1	leukocyte immunoglobulin-like receptor. subfamily B (with TM and ITIM domains). member 1
1.00	TCF20	transcription factor 20 (AR1)
1.00	TCF20	transcription factor 20 (AR1)
1.00	MED13L	mediator complex subunit 13-like
1.00	MAF1	MAF1 homolog. negative regulator of RNA polymerase III
1.00	ZNF232	zinc finger protein 232
1.00	ZNF804A	zinc finger protein 804A
1.00	FLJ22763; RP11-59E19.1	uncharacterized LOC401081; uncharacterized LOC401081 [Source:EntrezGene;Acc:401081]; novel transcript
1.00	VOPP1	Jeck2013 ALT_ACCEPTOR. ALT_DONOR. coding. INTERNAL. intronic best transcript NM_030796
1.00	ATP6V1A	ATPase. H+ transporting. lysosomal 70kDa. V1 subunit A
1.00	TP53I11	Memczak2013 ALT_ACCEPTOR. ALT_DONOR. coding. INTERNAL. intronic best transcript NM_001076787
1.00	COL18A1-AS1	COL18A1 antisense RNA 1
1.00	BAGE; BAGE4; BAGE3; BAGE2; BAGE5	B melanoma antigen; B melanoma antigen family. member 4; B melanoma antigen family. member 3; B melanoma antigen family. member 2; B melanoma antigen family. member 5
1.00	SLC7A3	solute carrier family 7 (cationic amino acid transporter. y+ system). member 3
1.00	ARHGEF10	Rho guanine nucleotide exchange factor 10
1.00	FAM188A	family with sequence similarity 188. member A
1.00	EPB41L5	erythrocyte membrane protein band 4.1 like 5

1.00	ITGAD	integrin alpha D
1.00	LARP7	La ribonucleoprotein domain family. member 7
1.00	NASP	Zhang2013 ALT_ACCEPTOR. ALT_DONOR. coding. INTERNAL. intronic best transcript NM_002482
1.00	FBXL2	F-box and leucine-rich repeat protein 2
1.00	LRRC34	leucine rich repeat containing 34
1.00	NISCH	Zhang2013 ALT_DONOR. coding. INTERNAL. intronic best transcript NM_007184
1.00	TCAP	titin-cap
1.00	POM121	POM121 transmembrane nucleoporin
1.00	KLHL40	kelch-like family member 40
1.00	CAPN9	calpain 9
1.00	TBC1D10A	TBC1 domain family. member 10A
1.00	ATP5G3	ATP synthase. H+ transporting. mitochondrial Fo complex subunit C3 (subunit 9)
1.00	LINC00521	long intergenic non-protein coding RNA 521
1.00	DTX1	deltex 1. E3 ubiquitin ligase
1.00	MFAP4	microfibrillar associated protein 4
1.00	GTSF1L	gametocyte specific factor 1-like
1.00	LRPAP1	LDL receptor related protein associated protein 1
1.00	INSR	insulin receptor
1.00	C8orf88	chromosome 8 open reading frame 88
1.00	CALY	calcyon neuron-specific vesicular protein
1.00	ARPC1B	actin related protein 2/3 complex subunit 1B
1.00	LRRC47	leucine rich repeat containing 47
1.00	SIK3	SIK family kinase 3
1.00	LOC400558; RP11-46C24.3	uncharacterized LOC400558; Transcript Identified by AceView. Entrez Gene

		ID(s) 400558; novel transcript
1.00	SLC5A8	solute carrier family 5 (sodium/monocarboxylate cotransporter). member 8
1.00	RER1	retention in endoplasmic reticulum sorting receptor 1
1.00	WIPI2	WD repeat domain. phosphoinositide interacting 2
1.00	CXorf51A; CXorf51B; CXorf51	Homo sapiens chromosome X open reading frame 51A (CXorf51A). mRNA.; Homo sapiens chromosome X open reading frame 51B (CXorf51B). mRNA.; Transcript Identified by AceView. Entrez Gene ID(s) 100129239. RefSeq ID(s) NM_001144064; chromosome X open reading frame 51A [Source:HGNC Symbol;Acc:HGNC:30533]; chromosome X open reading frame 51
1.00	OR12D2	olfactory receptor. family 12. subfamily D. member 2 (gene/pseudogene)
1.00	HGF	hepatocyte growth factor (hepapoietin A; scatter factor)
1.00	NDP	Norrie disease (pseudoglioma)
1.00	RPS11; SNORD35B	ribosomal protein S11; small nucleolar RNA. C/D box 35B
1.00	IL5RA	interleukin 5 receptor. alpha
1.00	LPIN1	Transcript Identified by AceView. Entrez Gene ID(s) 23175
1.00	PIK3R5	phosphoinositide-3-kinase. regulatory subunit 5
1.00	SGIP1	SH3-domain GRB2-like (endophilin) interacting protein 1
1.00	ZFYVE28	zinc finger. FYVE domain containing 28
1.00	SLC12A9	solute carrier family 12. member 9
1.00	GTF2A1L; STON1-GTF2A1L; STON1	general transcription factor IIA 1-like; STON1-GTF2A1L readthrough; stonin 1
1.00	DUXA	double homeobox A
1.00	TMEM189	transmembrane protein 189
1.00	COQ3	coenzyme Q3 methyltransferase
1.00	NUDT21	nudix hydrolase 21
1.00	TMSB15B; RP11-722G7.1	Homo sapiens thymosin beta 15B (TMSB15B). mRNA.; Thymosin beta-15B [Source:UniProtKB/Swiss-Prot;Acc:P0CG35]
1.00	SEPHS1	selenophosphate synthetase 1

1.00	TSNAXIP1	translin-associated factor X interacting protein 1
1.00	GRPEL1	GrpE-like 1. mitochondrial (E. coli)
1.00	ZNF71	zinc finger protein 71
1.00	NXPH2	neurexophilin 2
1.00	KCNMA1	Jeck2013 ALT_ACCEPTOR. ALT_DONOR. coding. INTERNAL. intronic best transcript NM_001014797
1.00	BICC1	BicC family RNA binding protein 1
1.00	GRID2	glutamate receptor. ionotropic. delta 2
1.00	GALNT16	polypeptide N-acetylgalactosaminyltransferase 16
1.00	PDCL2	phosducin like 2
1.00	SPATA24	spermatogenesis associated 24
1.00	ZNF304	zinc finger protein 304
1.00	S100A8	S100 calcium binding protein A8
1.00	BCAP31	B-cell receptor-associated protein 31
1.00	JRK	Jrk helix-turn-helix protein
1.00	KRTAP10-5	keratin associated protein 10-5
1.00	PRKAR2B	protein kinase. cAMP-dependent. regulatory. type II. beta
1.00	GOLGA8N; GOLGA8H	golgin A8 family. member N; golgin A8 family. member H
1.00	OR5AP2	olfactory receptor. family 5. subfamily AP. member 2
1.00	AAMP	angio-associated migratory cell protein
1.00	GOLGA6L4	Homo sapiens golgin A6 family-like 4 (GOLGA6L4). mRNA.; golgin A6 family-like 4; golgin A6 family-like 4 [Source:HGNC Symbol;Acc:HGNC:27256]
1.00	BRD7	bromodomain containing 7
1.00	KRTAP13-3	keratin associated protein 13-3
1.00	CES5A	carboxylesterase 5A
1.00	PRR22	proline rich 22
1.00	TRPC6	transient receptor potential cation channel. subfamily C. member 6

1.00	CRHR1	corticotropin releasing hormone receptor 1
1.00	CETP	cholesteryl ester transfer protein. plasma
1.00	WNT10A	wingless-type MMTV integration site family. member 10A
1.00	SLC4A4	solute carrier family 4 (sodium bicarbonate cotransporter). member 4
1.00	LOC101928137; RP11-314D7.2	uncharacterized LOC101928137; novel transcript
1.00	EPHA7	EPH receptor A7
1.00	SCGB1D1	secretoglobin. family 1D. member 1
1.00	MUC1	mucin 1. cell surface associated
1.00	CD81	CD81 molecule
1.00	KRT10	keratin 10. type I
1.00	ZFP42	ZFP42 zinc finger protein
1.00	DYRK1A	dual specificity tyrosine-(Y)-phosphorylation regulated kinase 1A
1.00	RGS19	regulator of G-protein signaling 19
1.00	OR10X1	olfactory receptor. family 10. subfamily X. member 1 (gene/pseudogene)
1.00	FKBP8	FK506 binding protein 8
1.00	OR51J1	olfactory receptor. family 51. subfamily J. member 1 (gene/pseudogene)
1.00	CSPG4P1Y; DNM1P24	chondroitin sulfate proteoglycan 4 pseudogene 1. Y-linked; dynamin 1 pseudogene 24
1.00	OR5L1	olfactory receptor. family 5. subfamily L. member 1 (gene/pseudogene)
1.00	TUBA1C	tubulin. alpha 1c
1.00	PTK2	Transcript Identified by AceView. Entrez Gene ID(s) 5747
1.00	HYOU1	hypoxia up-regulated 1
1.00	TNFRSF13B	tumor necrosis factor receptor superfamily. member 13B
1.00	SLC11A2	solute carrier family 11 (proton-coupled divalent metal ion transporter). member 2
1.00	NRF1	nuclear respiratory factor 1
1.00	PHGDH	phosphoglycerate dehydrogenase

1.00	C2orf69	chromosome 2 open reading frame 69
1.00	KCNJ18	potassium channel. inwardly rectifying subfamily J. member 18
1.00	CEP57L1	centrosomal protein 57kDa-like 1
1.00	PRKAG2	protein kinase. AMP-activated. gamma 2 non-catalytic subunit
1.00	EDRF1	erythroid differentiation regulatory factor 1
1.00	AC006128.2; AKAP8L	Memczak2013 ANTISENSE. coding. INTERNAL. intronic best transcript NM_014371; TEC
1.00	EIF3K	eukaryotic translation initiation factor 3. subunit K
1.00	ZKSCAN3	zinc finger with KRAB and SCAN domains 3
1.00	OR13C9	olfactory receptor. family 13. subfamily C. member 9
1.00	SLC22A11	solute carrier family 22 (organic anion/urate transporter). member 11
1.00	EOMES	eomesodermin
1.00	KRTAP21-3	keratin associated protein 21-3
1.00	PDILT	protein disulfide isomerase-like. testis expressed
1.00	CCND1	cyclin D1
1.00	KIRREL3	Jeck2013 ALT_ACCEPTOR. ALT_DONOR. coding. INTERNAL. intronic best transcript NM_032531
1.00	PPP3R2	protein phosphatase 3. regulatory subunit B. beta
1.00	RPRD1B	regulation of nuclear pre-mRNA domain containing 1B
1.00	MIS18BP1	MIS18 binding protein 1
1.00	SMIM7; MED26	small integral membrane protein 7; mediator complex subunit 26
1.00	MAGEA2	MAGE family member A2
1.00	SPRR1A	small proline-rich protein 1A
1.00	CABP5	calcium binding protein 5
1.00	FCF1	FCF1 rRNA-processing protein
1.00	TRH	thyrotropin-releasing hormone
1.00	STEAP4	STEAP family member 4

1.00	POLR1A	polymerase (RNA) I polypeptide A
1.00	RP1L1	retinitis pigmentosa 1-like 1
0.99	MCIDAS	multiciliate differentiation and DNA synthesis associated cell cycle protein
0.99	MRPL51	mitochondrial ribosomal protein L51
0.99	DNASE1L3	deoxyribonuclease I-like 3
0.99	CPSF4L	cleavage and polyadenylation specific factor 4-like
0.99	SETDB1	SET domain. bifurcated 1
0.99	PPIF	peptidylprolyl isomerase F
0.99	ZNF334	zinc finger protein 334
0.99	KCNA7	potassium channel. voltage gated shaker related subfamily A. member 7
0.99	RAB3IL1	RAB3A interacting protein (rabin3)-like 1
0.99	C19orf66	chromosome 19 open reading frame 66
0.99	LINGO1-AS1	LINGO1 antisense RNA 1
0.99	TRIM77	tripartite motif containing 77
0.99	ATAD5	ATPase family. AAA domain containing 5
0.99	ASCC3	Memczak2013 ALT_ACCEPTOR. ALT_DONOR. coding. INTERNAL. intronic best transcript NM_006828
0.99	NLRP5	NLR family. pyrin domain containing 5
0.99	MARCH2	membrane associated ring finger 2
0.99	SUPT16H	SPT16 homolog. facilitates chromatin remodeling subunit
0.99	UBE2B	ubiquitin conjugating enzyme E2B
0.99	LY86	lymphocyte antigen 86
0.99	AGMO	alkylglycerol monooxygenase
0.99	MDFIC	MyoD family inhibitor domain containing
0.99	NKX2-5	NK2 homeobox 5
0.99	NTN1	netrin 1

0.99	C7orf57	chromosome 7 open reading frame 57
0.99	FHL5	four and a half LIM domains 5
0.99	BRWD3	bromodomain and WD repeat domain containing 3
0.99	ABCG1	ATP binding cassette subfamily G member 1
0.99	ZSCAN5A	zinc finger and SCAN domain containing 5A
0.99	OCM2	oncomodulin 2
0.99	GOSR1	golgi SNAP receptor complex member 1
0.99	DYRK1B	dual specificity tyrosine-(Y)-phosphorylation regulated kinase 1B
0.99	FAM83G	family with sequence similarity 83. member G
0.99	UBQLNL	ubiquilin-like
0.99	BRDT	bromodomain. testis-specific
0.99	C5AR1	complement component 5a receptor 1
0.99	OR2T33	olfactory receptor. family 2. subfamily T. member 33
0.99	NCS1	neuronal calcium sensor 1
0.99	TOR1A	torsin family 1. member A (torsin A)
0.99	EEF2K	eukaryotic elongation factor 2 kinase
0.99	GAST	gastrin
0.99	GALNTL5	polypeptide N-acetylgalactosaminyltransferase-like 5
0.99	UTP23	UTP23. small subunit (SSU) processome component. homolog (yeast)
0.99	CELF1	Memczak2013 ALT_ACCEPTOR. ALT_DONOR. coding. INTERNAL. intronic best transcript NM_001172639
0.99	KDF1	keratinocyte differentiation factor 1
0.99	PKD1L2	polycystic kidney disease 1-like 2 (gene/pseudogene)
0.99	FXYD4	FXYD domain containing ion transport regulator 4
0.99	OR7E24	olfactory receptor. family 7. subfamily E. member 24
0.99	SPRR2G	small proline-rich protein 2G

0.99	IL3	interleukin 3
0.99	HIST1H2AA	histone cluster 1. H2aa
0.99	SDCBP	syndecan binding protein
0.99	CDH5	cadherin 5. type 2 (vascular endothelium)
0.99	TMEM42	transmembrane protein 42
0.99	ZDHHC3	zinc finger. DHHC-type containing 3
0.99	HIF1A	hypoxia inducible factor 1. alpha subunit (basic helix-loop-helix transcription factor)
0.99	PPP1R9A	protein phosphatase 1. regulatory subunit 9A
0.99	ESYT2	extended synaptotagmin-like protein 2
0.99	PGAM1	Homo sapiens phosphoglycerate mutase 1 (brain). mRNA (cDNA clone MGC:8462 IMAGE:2821567). complete cds.; Homo sapiens phosphoglycerate mutase 1 (brain). mRNA (cDNA clone MGC:19732 IMAGE:3604026). complete cds.; Homo sapiens phosphoglycerate mutase 1 (brain). mRNA (cDNA clone MGC:15086 IMAGE:4299008). complete cds.; Homo sapiens phosphoglycerate mutase 1 (brain). mRNA (cDNA clone MGC:61519 IMAGE:5787678). complete cds.; Homo sapiens phosphoglycerate mutase 1 (brain). mRNA (cDNA clone MGC:71323 IMAGE:6584466). complete cds.; Homo sapiens phosphoglycerate mutase 1 (brain). mRNA (cDNA clone MGC:87445 IMAGE:5262935). complete cds.; Homo sapiens phosphoglycerate mutase 1 (brain). mRNA (cDNA clone MGC:88744 IMAGE:6297173). complete cds.; Homo sapiens phosphoglycerate mutase 1 (brain). mRNA (cDNA clone MGC:189702 IMAGE:8862699). complete cds.
0.99	DYNC1LI1	dynein. cytoplasmic 1. light intermediate chain 1
0.99	NPIPA7	nuclear pore complex interacting protein family. member A7
0.99	CD6	CD6 molecule
0.99	C17orf112	chromosome 17 open reading frame 112
0.99	PAX9	paired box 9
0.99	BLZF1	basic leucine zipper nuclear factor 1
0.99	FIZ1	FLT3-interacting zinc finger 1

0.99	ESYT1	extended synaptotagmin-like protein 1
0.99	WFDC1	WAP four-disulfide core domain 1
0.99	IQSEC3	IQ motif and Sec7 domain 3
0.99	HMCN1	hemicentin 1
0.99	COMTD1	catechol-O-methyltransferase domain containing 1
0.99	MAGEC3	MAGE family member C3
0.99	TRIM3	tripartite motif containing 3
0.99	VIT	vitrin
0.99	OR12D1	olfactory receptor. family 12. subfamily D. member 1 (gene/pseudogene) [Source:HGNC Symbol;Acc:HGNC:8177]
0.99	SRSF11	serine/arginine-rich splicing factor 11
0.99	IRF8	interferon regulatory factor 8
0.99	LONP1	lon peptidase 1. mitochondrial
0.99	GPR135	G protein-coupled receptor 135
0.99	SNRPA1	small nuclear ribonucleoprotein polypeptide A
0.99	XRCC5	Transcript Identified by AceView. Entrez Gene ID(s) 7520
0.99	SOX1	SRY box 1
0.99	CFAP221	cilia and flagella associated protein 221
0.99	TCEB2	transcription elongation factor B (SIII). polypeptide 2 (18kDa. elongin B)
0.99	LRCH4	leucine-rich repeats and calponin homology (CH) domain containing 4
0.99	TYRO3	TYRO3 protein tyrosine kinase
0.99	GRIA3	glutamate receptor. ionotropic. AMPA 3
0.99	CRYAA	crystallin alpha A
0.99	FBXW9	F-box and WD repeat domain containing 9
0.99	SMAD7	SMAD family member 7
0.99	HNRNPH1	heterogeneous nuclear ribonucleoprotein H1 (H)

0.99	KRTAP13-1	keratin associated protein 13-1
0.99	MTL5	metallothionein-like 5. testis-specific (tesmin)
0.99	GALNT18	polypeptide N-acetylgalactosaminyltransferase 18
0.99	PBDC1	polysaccharide biosynthesis domain containing 1
0.99	C4orf45	chromosome 4 open reading frame 45
0.99	SLAMF1	signaling lymphocytic activation molecule family member 1
0.99	CRHBP	corticotropin releasing hormone binding protein
0.99	ZNF573	Transcript Identified by AceView. Entrez Gene ID(s) 126231
0.99	C3orf80	chromosome 3 open reading frame 80
0.99	KIAA1257	KIAA1257
0.99	SEMA4B	sema domain. immunoglobulin domain (Ig). transmembrane domain (TM) and short cytoplasmic domain. (semaphorin) 4B
0.99	SUPT20H	SPT20 homolog. SAGA complex component
0.99	IL1F10	interleukin 1 family. member 10 (theta)
0.99	CCDC3	coiled-coil domain containing 3
0.99	STAP2	signal transducing adaptor family member 2
0.99	CHORDC1	cysteine and histidine rich domain containing 1
0.99	UBQLN2	ubiquilin 2
0.99	SPECC1L-ADORA2A	SPECC1L-ADORA2A readthrough (NMD candidate)
0.99	UBA5	ubiquitin-like modifier activating enzyme 5
0.99	TJP1	tight junction protein 1
0.99	VN1R5	vomeronasal 1 receptor 5 (gene/pseudogene)
0.99	PIWIL3	piwi-like RNA-mediated gene silencing 3
0.99	MED6	mediator complex subunit 6
0.99	ST6GALNAC3	ST6 (alpha-N-acetyl-neuraminyl-2.3-beta-galactosyl-1.3)-N-acetylgalactosaminide alpha-2.6-sialyltransferase 3

0.99	TOR4A	torsin family 4. member A
0.99	QRICH2	glutamine rich 2
0.99	DDX54	DEAD (Asp-Glu-Ala-Asp) box polypeptide 54
0.99	FANCM	Fanconi anemia complementation group M
0.99	ALX4	ALX homeobox 4
0.99	PARP12	poly(ADP-ribose) polymerase family member 12
0.99	XRCC5	X-ray repair complementing defective repair in Chinese hamster cells 5 (double-strand-break rejoining)
0.99	CASC10	cancer susceptibility candidate 10
0.99	ANKRD54; MIR658	ankyrin repeat domain 54; microRNA 658
0.99	ZFP3	ZFP3 zinc finger protein
0.99	AC018462.2; COMMD1	Salzman2013 ANTISENSE. coding. INTERNAL. intronic. OVERLAPTX best transcript NM_152516; novel transcript
0.99	DDX28	DEAD (Asp-Glu-Ala-Asp) box polypeptide 28
0.99	OR51G1	olfactory receptor. family 51. subfamily G. member 1 (gene/pseudogene)
0.99	OR9G4	olfactory receptor. family 9. subfamily G. member 4
0.99	TAF6L	TAF6-like RNA polymerase II. p300/CBP-associated factor (PCAF)-associated factor. 65kDa
0.99	LEPROTL1	leptin receptor overlapping transcript-like 1
0.99	LEFTY2	left-right determination factor 2
0.99	SFRP5	secreted frizzled-related protein 5
0.99	MAGEA10-MAGEA5; RP11-1007I13.4	MAGEA10-MAGEA5 readthrough; novel transcript
0.99	CD1B	CD1b molecule
0.99	DHRS11	dehydrogenase/reductase (SDR family) member 11
0.99	LAMB2	laminin. beta 2 (laminin S)
0.99	NBPF10	neuroblastoma breakpoint family. member 10

0.99	ZNF468	zinc finger protein 468
0.99	INPP5D	inositol polyphosphate-5-phosphatase D
0.99	NAA16	N(alpha)-acetyltransferase 16. NatA auxiliary subunit
0.99	PRC1-AS1	PRC1 antisense RNA 1
0.99	DHX29	DEAH (Asp-Glu-Ala-His) box polypeptide 29
0.99	RPS6KB1	ribosomal protein S6 kinase. 70kDa. polypeptide 1
0.99	CFAP36	cilia and flagella associated protein 36
0.99	UBR2	ubiquitin protein ligase E3 component n-recognin 2
0.99	LRMP	lymphoid-restricted membrane protein
0.99	CEACAM1	carcinoembryonic antigen-related cell adhesion molecule 1 (biliary glycoprotein)
0.99	HGSNAT	heparan-alpha-glucosaminide N-acetyltransferase
0.99	ADAM15	ADAM metallopeptidase domain 15
0.99	ESPNL	espin-like
0.99	B3GAT3	beta-1.3-glucuronyltransferase 3
0.99	PHKA2	phosphorylase kinase. alpha 2 (liver)
0.99	FAM63A	family with sequence similarity 63. member A
0.99	MAST1	microtubule associated serine/threonine kinase 1
0.99	BLID; MIR100HG	BH3-like motif containing. cell death inducer; mir-100-let-7a-2 cluster host gene
0.99	BIVM	basic. immunoglobulin-like variable motif containing
0.99	KRTAP2-3	keratin associated protein 2-3
0.99	BACH2	Memczak2013 ALT_ACCEPTOR. ALT_DONOR. coding. INTERNAL. intronic best transcript NM_001170794
0.99	TLR2	toll-like receptor 2
0.99	PLEKHD1	pleckstrin homology domain containing. family D (with coiled-coil domains) member 1

0.99	FAM24B	family with sequence similarity 24. member B
0.99	KLC3	kinesin light chain 3
0.99	CDH3	cadherin 3. type 1. P-cadherin (placental)
0.99	BCL2L2	BCL2-like 2
0.99	BATF3	basic leucine zipper transcription factor. ATF-like 3
0.99	MARC2	mitochondrial amidoxime reducing component 2
0.99	ERH	enhancer of rudimentary homolog (Drosophila)
0.99	PRR30	proline rich 30
0.99	BTBD17	BTB (POZ) domain containing 17
0.99	SCRIB; MIR937	scribbled planar cell polarity protein; microRNA 937
0.99	EXOSC1	exosome component 1
0.99	SURF6	surfeit 6
0.99	FKBP10	FK506 binding protein 10
0.99	RPL28; MIR6805	ribosomal protein L28; microRNA 6805
0.99	HNRNPUL1	heterogeneous nuclear ribonucleoprotein U-like 1
0.99	CCDC103	coiled-coil domain containing 103
0.99	RBMY1E; RBMY1B	RNA binding motif protein. Y-linked. family 1. member E; RNA binding motif protein. Y-linked. family 1. member B
0.99	GREM2	gremlin 2. DAN family BMP antagonist
0.99	MRPL57	mitochondrial ribosomal protein L57
0.99	KLF14	Kruppel-like factor 14
0.99	OR2A12	olfactory receptor. family 2. subfamily A. member 12
0.99	EVX2	even-skipped homeobox 2
0.99	PLEKHA8P1	pleckstrin homology domain containing. family A member 8 pseudogene 1
0.99	FAM110A	family with sequence similarity 110. member A
0.99	ARHGAP10	Rho GTPase activating protein 10

0.99	OR4K5	olfactory receptor. family 4. subfamily K. member 5
0.99	SLC43A1	solute carrier family 43 (amino acid system L transporter). member 1
0.99	FCRL5	Fc receptor-like 5
0.99	PLA2G4D	phospholipase A2. group IVD (cytosolic)
0.99	GHRL	ghrelin/obestatin prepropeptide
0.99	GNRH2	gonadotropin releasing hormone 2
0.99	SH2D2A	SH2 domain containing 2A
0.99	CNPPD1	cyclin Pas1/PHO80 domain containing 1
0.99	LINC01561	long intergenic non-protein coding RNA 1561
0.99	KLK10	kallikrein related peptidase 10
0.99	GRIFIN	galectin-related inter-fiber protein
0.99	HSPA12B	heat shock 70kD protein 12B
0.99	SDR42E1	short chain dehydrogenase/reductase family 42E. member 1
0.99	NOS2	nitric oxide synthase 2. inducible
0.99	CXorf49B; CXorf49	chromosome X open reading frame 49B; chromosome X open reading frame 49
0.99	C14orf79	chromosome 14 open reading frame 79
0.99	LOC100131107	putative UPF0607 protein ENSP00000383783
0.99	WNT7A	wingless-type MMTV integration site family. member 7A
0.99	REEP1	receptor accessory protein 1
0.99	KRT85	keratin 85. type II
0.99	ZNF300	zinc finger protein 300
0.99	NPY4R	neuropeptide Y receptor Y4
0.99	PLCD4	phospholipase C. delta 4
0.99	FAM109B	family with sequence similarity 109. member B
0.99	SLFN13	schlafen family member 13

0.99	HNF1A	HNF1 homeobox A
0.99	KAT6B	K(lysine) acetyltransferase 6B
0.99	PCDHB9	protocadherin beta 9
0.99	CD86	CD86 molecule
0.99	ZNF721	Salzman2013 ALT_ACCEPTOR. ALT_DONOR. coding. INTERNAL. intronic best transcript NM_133474
0.99	TPMT	thiopurine S-methyltransferase
0.99	COL6A3	collagen. type VI. alpha 3
0.99	C5orf60	chromosome 5 open reading frame 60
0.99	DOLPP1	dolichyldiphosphatase 1
0.99	PGM5	phosphoglucomutase 5
0.99	FPR2	formyl peptide receptor 2
0.99	GARNL3	GTPase activating Rap/RanGAP domain-like 3
0.99	UPK1A-AS1	UPK1A antisense RNA 1
0.99	OR2C3; GCSAML-AS1	olfactory receptor. family 2. subfamily C. member 3; GCSAML antisense RNA 1
0.99	ZNF586	zinc finger protein 586
0.99	OR10Q1	olfactory receptor. family 10. subfamily Q. member 1
0.99	UBE2I	ubiquitin conjugating enzyme E2I
0.99	TRAF3IP1	TNF receptor-associated factor 3 interacting protein 1
0.99	ADAMTS13	ADAM metallopeptidase with thrombospondin type 1 motif 13
0.99	DHRS9	dehydrogenase/reductase (SDR family) member 9
0.99	KLRC4-KLRK1; KLRK1; KLRC4	KLRC4-KLRK1 readthrough; killer cell lectin-like receptor subfamily K. member 1; killer cell lectin-like receptor subfamily C. member 4
0.99	ARHGAP1	Memczak2013 ANTISENSE. CDS. coding. INTERNAL. UTR3 best transcript NM_004308
0.99	PHYHIPL	phytanoyl-CoA 2-hydroxylase interacting protein-like

0.99	MUC17	mucin 17. cell surface associated
0.99	FOXB1	forkhead box B1
0.99	CHRFAM7A	CHRNA7 (cholinergic receptor. nicotinic. alpha 7. exons 5-10) and FAM7A (family with sequence similarity 7A. exons A-E) fusion
0.99	CCDC96	coiled-coil domain containing 96
0.99	REPIN1	replication initiator 1
0.99	FAF2	Fas associated factor family member 2
0.99	NYNRIN	NYN domain and retroviral integrase containing
0.99	TMEM202	transmembrane protein 202
0.99	RYR3	ryanodine receptor 3
0.99	NPHS1	nephrosis 1. congenital. Finnish type (nephrin)
0.99	BIK	BCL2-interacting killer (apoptosis-inducing)
0.99	PAIP2; CTB-43P18.1	poly(A) binding protein interacting protein 2 [Source:EntrezGene;Acc:51247]; novel transcript. antisense to PAIP2
0.99	ZKSCAN4	zinc finger with KRAB and SCAN domains 4
0.99	C14orf39	chromosome 14 open reading frame 39
0.99	LCTL	lactase-like
0.99	MIR7162; GOLGA2P6; MTPAP	microRNA 7162; golgin A2 pseudogene 6; mitochondrial poly(A) polymerase
0.99	PADI4	peptidyl arginine deiminase. type IV
0.99	PSG2	pregnancy specific beta-1-glycoprotein 2
0.99	URI1	URI1. prefoldin-like chaperone
0.99	IL20	interleukin 20
0.99	ADGRG2	adhesion G protein-coupled receptor G2
0.99	DACH1	Memczak2013 ANTISENSE. coding. INTERNAL. intronic best transcript NM_080760
0.99	OR2L3	olfactory receptor. family 2. subfamily L. member 3
0.99	NPY1R	neuropeptide Y receptor Y1

0.99	SRSF5	serine/arginine-rich splicing factor 5
0.99	NEDD4	neural precursor cell expressed. developmentally down-regulated 4. E3 ubiquitin protein ligase
0.99	SPANXN3	SPANX family. member N3
0.99	WIPF3	WAS/WASL interacting protein family. member 3
0.99	PDGFRA	platelet-derived growth factor receptor. alpha polypeptide
0.99	C1QTNF4	C1q and tumor necrosis factor related protein 4
0.99	OR8B12	olfactory receptor. family 8. subfamily B. member 12
0.99	ADCY9	adenylate cyclase 9
0.99	CTSW	cathepsin W
0.99	TSPYL5	TSPY-like 5
0.99	TMEM138	transmembrane protein 138
0.99	TXNRD3	thioredoxin reductase 3
0.99	NEU3	sialidase 3 (membrane sialidase)
0.99	PGGT1B	protein geranylgeranyltransferase type I. beta subunit
0.99	ADAM2	ADAM metallopeptidase domain 2
0.99	MC3R	melanocortin 3 receptor
0.99	FIBCD1	fibrinogen C domain containing 1
0.99	POM121C	POM121 transmembrane nucleoporin C
0.99	PRSS50	protease. serine 50
0.99	C8B	complement component 8. beta polypeptide
0.99	PKNOX2	PBX/knotted 1 homeobox 2
0.99	SCAP	Memczak2013 ANTISENSE. CDS. coding. INTERNAL best transcript NM_012235
0.99	IGFL2	IGF like family member 2
0.99	GPX1	glutathione peroxidase 1
0.99	NAV1	neuron navigator 1

0.99	RTBDN	retbindin
0.99	PRKCDBP	protein kinase C. delta binding protein
0.99	KHDC1	KH homology domain containing 1
0.99	ANKRD10	ankyrin repeat domain 10
0.99	ANAPC13	anaphase promoting complex subunit 13
0.99	ANP32C	acidic nuclear phosphoprotein 32 family member C
0.99	MICU3	mitochondrial calcium uptake family. member 3
0.99	LDLRAD3	low density lipoprotein receptor class A domain containing 3
0.99	CDS2	CDP-diacylglycerol synthase 2
0.99	NUP210L; MIR5698	nucleoporin 210kDa like; microRNA 5698
0.99	ZNF700	zinc finger protein 700
0.99	MAP2K7	mitogen-activated protein kinase kinase 7
0.99	ESRP2; MIR6773	epithelial splicing regulatory protein 2; microRNA 6773
0.99	PDZD4	PDZ domain containing 4
0.99	LHFPL1	lipoma HMGIC fusion partner-like 1
0.99	CSTF3	cleavage stimulation factor. 3 pre-RNA. subunit 3
0.99	OMG	oligodendrocyte myelin glycoprotein
0.99	SLC19A1	solute carrier family 19 (folate transporter). member 1
0.99	PARP11	poly(ADP-ribose) polymerase family member 11
0.99	INF2	inverted formin. FH2 and WH2 domain containing
0.99	TFAP2B	transcription factor AP-2 beta (activating enhancer binding protein 2 beta)
0.99	FBL	fibrillarin
0.99	PDDC1	Parkinson disease 7 domain containing 1
0.99	HHAT	hedgehog acyltransferase
0.99	ZNF788	zinc finger family member 788
0.99	SSX8	synovial sarcoma. X breakpoint 8

0.99	EPHB4	EPH receptor B4
0.99	GPR152	G protein-coupled receptor 152
0.99	RBM8A	RNA binding motif protein 8A
0.99	TNF	tumor necrosis factor
0.99	LINC01429	long intergenic non-protein coding RNA 1429
0.99	NANOG	Nanog homeobox
0.99	SPAG17	sperm associated antigen 17
0.99	LRMP	lymphoid-restricted membrane protein
0.99	ITGAX	integrin alpha X
0.99	RNF148	ring finger protein 148
0.99	PRUNE2	prune homolog 2 (Drosophila)
0.99	C1orf56	chromosome 1 open reading frame 56
0.99	EEF1A1	eukaryotic translation elongation factor 1 alpha 1
0.99	RAB8A	RAB8A. member RAS oncogene family
0.99	ARMC1	armadillo repeat containing 1
0.99	PRAMEF25	PRAME family member 25
0.99	MKL2; TVP23CP2	MKL/myocardin-like 2; TVP23C pseudogene 2
0.99	EBF1	early B-cell factor 1
0.99	GSTO2	glutathione S-transferase omega 2
0.99	SEBOX	SEBOX homeobox
0.99	MIA3	melanoma inhibitory activity family. member 3
0.99	COL4A1	collagen. type IV. alpha 1
0.99	NECAB3	N-terminal EF-hand calcium binding protein 3
0.99	COL7A1; MIR711	collagen. type VII. alpha 1; microRNA 711
0.99	ZNF273	zinc finger protein 273
0.99	SH3GLB2	SH3-domain GRB2-like endophilin B2

0.99	CFAP57	cilia and flagella associated protein 57
0.99	MAP3K14	mitogen-activated protein kinase kinase kinase 14
0.99	NBPF8	neuroblastoma breakpoint family. member 8
0.99	ZNF26	zinc finger protein 26
0.99	AFF4	Memczak2013 ANTISENSE. CDS. coding. INTERNAL best transcript NM_014423
0.99	ASIC1	acid sensing ion channel 1
0.99	RPS6KA1	ribosomal protein S6 kinase. 90kDa. polypeptide 1
0.99	GPR65	G protein-coupled receptor 65
0.99	CGB7	chorionic gonadotropin. beta polypeptide 7
0.99	TMEM95	transmembrane protein 95
0.99	DMRT2	doublesex and mab-3 related transcription factor 2
0.99	HAND2	heart and neural crest derivatives expressed 2
0.99	ZNF575	zinc finger protein 575
0.99	STAT5A	signal transducer and activator of transcription 5A
0.99	TLL2	tolloid like 2
0.99	KDM4C	Memczak2013 ANTISENSE. coding. INTERNAL. intronic best transcript NM_001146694
0.99	SCUBE1	signal peptide. CUB domain. EGF-like 1
0.99	YLPM1	YLP motif containing 1
0.99	OR5T3	olfactory receptor. family 5. subfamily T. member 3
0.99	KIAA1456	KIAA1456
0.99	PRR15	proline rich 15
0.99	OR2L5	olfactory receptor. family 2. subfamily L. member 5
0.99	ACSS2	Memczak2013 ANTISENSE. CDS. coding. INTERNAL best transcript NM_001076552
0.99	BRD8	bromodomain containing 8

0.99	NT5C2	5-nucleotidase. cytosolic II
0.99	CENPV	centromere protein V
0.99	HMBS	hydroxymethylbilane synthase
0.99	GATA3	GATA binding protein 3
0.99	AP2S1	adaptor-related protein complex 2 sigma 1 subunit
0.99	FCRLA	Fc receptor-like A
0.99	FAAH2	fatty acid amide hydrolase 2
0.99	ZNF391	zinc finger protein 391
0.99	ETF1	eukaryotic translation termination factor 1
0.99	DRG2	developmentally regulated GTP binding protein 2
0.99	HAS3	hyaluronan synthase 3
0.99	HSD3B2	hydroxy-delta-5-steroid dehydrogenase. 3 beta- and steroid delta-isomerase 2
0.99	ITGAE	integrin alpha E
0.99	DAZAP2	DAZ associated protein 2
0.99	LMOD3	leiomodin 3 (fetal)
0.99	DEFB129	defensin. beta 129
0.99	DNAJC15	DnaJ (Hsp40) homolog. subfamily C. member 15
0.99	C17orf107	chromosome 17 open reading frame 107
0.99	RBMY1J; RBMY1F	RNA binding motif protein. Y-linked. family 1. member J; RNA binding motif protein. Y-linked. family 1. member F
0.99	RP11-517A5.5; NOMO3; RP11-517A5.4	NODAL modulator 3 [Source:HGNC Symbol;Acc:HGNC:25242]; novel transcript. antisense to NOMO3; RP11-517A5.4 (from geneSymbol)
0.99	KCNK6	potassium channel. two pore domain subfamily K. member 6
0.99	RIPK1	receptor (TNFRSF)-interacting serine-threonine kinase 1
0.99	PIKFYVE	phosphoinositide kinase. FYVE finger containing
0.99	C7orf50	chromosome 7 open reading frame 50

0.99	INCA1	inhibitor of CDK. cyclin A1 interacting protein 1
0.99	UTS2	urotensin 2
0.99	RPL39L	ribosomal protein L39-like
0.99	PKN1	protein kinase N1
0.99	HTR2A	5-hydroxytryptamine (serotonin) receptor 2A. G protein-coupled
0.99	PPM1L	protein phosphatase. Mg2+/Mn2+ dependent. 1L
0.99	WTAP	Wilms tumor 1 associated protein
0.99	OR5D13	olfactory receptor. family 5. subfamily D. member 13 (gene/pseudogene)
0.99	USP8	ubiquitin specific peptidase 8
0.99	UQCRC1	ubiquinol-cytochrome c reductase core protein I
0.99	OTUD7B	OTU deubiquitinase 7B
0.99	QKI	QKI. KH domain containing. RNA binding
0.99	CCDC163P	coiled-coil domain containing 163. pseudogene
0.99	TCP10L2	t-complex 10-like 2
0.99	AMZ1	archaelysin family metallopeptidase 1
0.99	TAF1	Transcript Identified by AceView. Entrez Gene ID(s) 6872
0.99	ACACA	Transcript Identified by AceView. Entrez Gene ID(s) 31
0.99	FAM171A1	family with sequence similarity 171. member A1
0.99	FAM83A	family with sequence similarity 83. member A
0.99	CEP83	centrosomal protein 83kDa
0.99	OR4K3	olfactory receptor family 4 subfamily K member 3 (gene/pseudogene) [Source:HGNC Symbol;Acc:HGNC:14731]
0.99	ZNF585B	zinc finger protein 585B
0.99	CEACAM18	carcinoembryonic antigen-related cell adhesion molecule 18
0.99	ZBED5	zinc finger. BED-type containing 5
0.99	OR10J5	olfactory receptor. family 10. subfamily J. member 5

0.99	NOTO	notochord homeobox
0.99	SPATA8	spermatogenesis associated 8
0.99	PSKH2	protein serine kinase H2
0.99	KIAA1755	KIAA1755
0.99	PRKCH	protein kinase C. eta
0.99	SGK494; FLJ25006; KIAA0100andSGK494andSPAG5; RP11-192H23.4	uncharacterized serine/threonine-protein kinase SgK494; Synthetic construct Homo sapiens clone IMAGE:100064171. MGC:193254 uncharacterized serine/threonine-protein kinase SgK494 (FLJ25006) mRNA. encodes complete protein.; Salzman2013 ANNOTATED. CDS. coding. INTERNAL. OVCODE. OVERLAPTX. OVEXON best transcript NM_001174103; Transcript Identified by AceView. Entrez Gene ID(s) 124923; 9703; 645851; 10615; uncharacterized serine/threonine-protein kinase SgK494 [Source:EntrezGene;Acc:124923]; Uncharacterized protein [Source:UniProtKB/TrEMBL;Acc:J3KTE0]
0.99	IMPG2	interphotoreceptor matrix proteoglycan 2
0.99	SERPINB12	serpin peptidase inhibitor. clade B (ovalbumin). member 12
0.99	HOOK1	hook microtubule-tethering protein 1
0.99	LRRC59	leucine rich repeat containing 59
0.99	CRLF1	cytokine receptor-like factor 1
0.99	TEX13A	testis expressed 13A
0.99	RIMS1	regulating synaptic membrane exocytosis 1
0.99	PAX7	paired box 7
0.99	ERVFRD-1	endogenous retrovirus group FRD. member 1
0.99	ACTR1A	ARP1 actin-related protein 1 homolog A. centractin alpha (yeast)
0.99	LUZP2	leucine zipper protein 2
0.99	PDE8B	phosphodiesterase 8B
0.99	ATP2A2	ATPase. Ca++ transporting. cardiac muscle. slow twitch 2
0.99	PDSS2	prenyl (decaprenyl) diphosphate synthase. subunit 2
0.99	XPO4	exportin 4

0.99	FBLN2	fibulin 2
0.99	HSPA4	heat shock 70kDa protein 4
0.99	DENND2C	DENN/MADD domain containing 2C
0.99	ELAVL3	ELAV like neuron-specific RNA binding protein 3
0.99	CHD5	chromodomain helicase DNA binding protein 5
0.99	RPS26	Homo sapiens ribosomal protein S26. mRNA (cDNA clone MGC:27148 IMAGE:4807004). complete cds.; Homo sapiens ribosomal protein S26. mRNA (cDNA clone MGC:88201 IMAGE:6388128). complete cds.; Homo sapiens ribosomal protein S26. mRNA (cDNA clone MGC:104291 IMAGE:4287636). complete cds.; Homo sapiens ribosomal protein S26. mRNA (cDNA clone MGC:104292 IMAGE:6726218). complete cds.
0.99	KLHL5	kelch-like family member 5
0.99	PADI1	peptidyl arginine deiminase. type I
0.99	TXNL1	thioredoxin-like 1
0.99	CAMKMT	calmodulin-lysine N-methyltransferase
0.99	IRX3	iroquois homeobox 3
0.99	DGKB	diacylglycerol kinase. beta 90kDa
0.99	TOR1AIP1	torsin A interacting protein 1
0.99	OSTM1	osteopetrosis associated transmembrane protein 1
0.99	MYCBP; GJA9	MYC binding protein; gap junction protein alpha 9
0.99	CHP2	calcineurin-like EF-hand protein 2
0.99	TMEM109	transmembrane protein 109
0.99	GPN2	GPN-loop GTPase 2
0.99	SLC24A5	solute carrier family 24 (sodium/potassium/calcium exchanger). member 5
0.99	IKZF3	IKAROS family zinc finger 3
0.99	C1QTNF2	C1q and tumor necrosis factor related protein 2
0.99	EDEM1	Transcript Identified by AceView. Entrez Gene ID(s) 9695
0.99	FGF3	fibroblast growth factor 3

0.99	EYA4	EYA transcriptional coactivator and phosphatase 4
0.99	ATP13A5-AS1	ATP13A5 antisense RNA 1
0.99	MLF2	myeloid leukemia factor 2
0.99	CGB5; CGB8	chorionic gonadotropin. beta polypeptide 5; chorionic gonadotropin. beta polypeptide 8
0.99	ATP6V0E1	ATPase. H+ transporting. lysosomal 9kDa. V0 subunit e1
0.99	COL25A1	collagen. type XXV. alpha 1
0.99	RNF213	ring finger protein 213
0.99	LIPM	lipase. family member M
0.99	CARD17	caspase recruitment domain family. member 17
0.99	FOXD3	forkhead box D3
0.99	PRAMEF25	PRAME family member 25
0.99	PRAMEF25	PRAME family member 25
0.99	KCNJ12	potassium channel. inwardly rectifying subfamily J. member 12
0.99	DCDC2B	doublecortin domain containing 2B
0.99	GOLGA8K; ULK4P1	golgin A8 family. member K; ULK4 pseudogene 1
0.99	NT5C3B	5-nucleotidase. cytosolic IIIB
0.99	SPAG11A; SPAG11B	sperm associated antigen 11A; sperm associated antigen 11B
0.99	TMEM247	transmembrane protein 247
0.99	RNF114	ring finger protein 114
0.99	TBXA2R	thromboxane A2 receptor
0.99	SYT8	synaptotagmin VIII
0.99	DGKZ	diacylglycerol kinase. zeta
0.99	B3GALT5	UDP-Gal:betaGlcNAc beta 1.3-galactosyltransferase 5
0.99	PNKD; MIR6810	paroxysmal nonkinesigenic dyskinesia; microRNA 6810
0.99	flawmawbo; GBP3	Transcript Identified by AceView; Memczak2013 ANTISENSE. CDS. coding. INTERNAL. intronic. UTR5 best transcript NM_018284

0.99	C16orf72	chromosome 16 open reading frame 72
0.99	ADIPOR2	adiponectin receptor 2
0.99	YBX1	Y box binding protein 1
0.99	LRRK2	leucine-rich repeat kinase 2
0.99	LOC730159; RP11-360D2.1	uncharacterized LOC730159; putative novel transcript
0.99	RAB2A	Memczak2013 ALT_ACCEPTOR. ALT_DONOR. coding. INTERNAL. intronic best transcript NM_002865
0.99	FUBP3	far upstream element (FUSE) binding protein 3
0.99	BMPER	BMP binding endothelial regulator
0.99	FBXL4	F-box and leucine-rich repeat protein 4
0.99	SPINT3	serine peptidase inhibitor. Kunitz type. 3
0.99	RFX1	regulatory factor X. 1 (influences HLA class II expression)
0.99	PSMB5	proteasome subunit beta 5
0.99	ZNF799	zinc finger protein 799
0.99	PRR21	proline rich 21
0.99	CCDC66	coiled-coil domain containing 66
0.99	PIN4	peptidylprolyl cis/trans isomerase. NIMA-interacting 4
0.99	TMEM217	transmembrane protein 217
0.99	AKAP3	A kinase (PRKA) anchor protein 3
0.99	AGAP1; noygloy; torkeybo	Jeck2013 ALT_ACCEPTOR. ALT_DONOR. coding. INTERNAL. intronic best transcript NM_001037131; Transcript Identified by AceView
0.99	ATP1A2	ATPase. Na+/K+ transporting. alpha 2 polypeptide
0.99	KRR1	KRR1. small subunit (SSU) processome component. homolog (yeast)
0.99	CDRT15L2	CMT1A duplicated region transcript 15-like 2
0.99	SLIT3	slit guidance ligand 3
0.99	ZNF540; ZNF571-AS1	zinc finger protein 540; ZNF571 antisense RNA 1
0.99	SLC35G1	solute carrier family 35. member G1

0.99	GAS6-AS1	GAS6 antisense RNA 1
0.99	TMPRSS11B	transmembrane protease. serine 11B
0.99	ARHGEF11	Rho guanine nucleotide exchange factor 11
0.99	FER1L6-AS1	FER1L6 antisense RNA 1
0.99	KIAA1683	Memczak2013 ANTISENSE. CDS. coding. INTERNAL best transcript NM_001145304
0.99	C17orf58	chromosome 17 open reading frame 58
0.99	RNF121	ring finger protein 121
0.99	SLC5A10	solute carrier family 5 (sodium/sugar cotransporter). member 10
0.99	TERT	Zhang2013 ALT_ACCEPTOR. ALT_DONOR. coding. INTERNAL. intronic best transcript NM_198253
0.99	KRT78	keratin 78. type II
0.99	DHRS3; MIR6730	dehydrogenase/reductase (SDR family) member 3; microRNA 6730
0.99	FRK	fyn-related Src family tyrosine kinase
0.99	ENTPD3	ectonucleoside triphosphate diphosphohydrolase 3
0.99	FAM26E	family with sequence similarity 26. member E
0.99	USP38	ubiquitin specific peptidase 38
0.99	TMEM19	transmembrane protein 19
0.99	HS3ST3A1	Jeck2013 ALT_ACCEPTOR. ALT_DONOR. coding. INTERNAL. intronic best transcript NM_006042
0.99	TMEM105	transmembrane protein 105
0.99	C12orf43	chromosome 12 open reading frame 43
0.99	HCG27	HLA complex group 27 (non-protein coding)
0.99	GCKR	Transcript Identified by AceView. Entrez Gene ID(s) 2646
0.99	TIMM44	translocase of inner mitochondrial membrane 44 homolog (yeast)
0.98	CEP192	centrosomal protein 192kDa
0.98	SV2C	synaptic vesicle glycoprotein 2C

0.98	CUL9	cullin 9
0.98	CADM3	cell adhesion molecule 3
0.98	FFAR4	free fatty acid receptor 4
0.98	GPAA1	glycosylphosphatidylinositol anchor attachment 1
0.98	TRIM5	tripartite motif containing 5
0.98	IL33	interleukin 33
0.98	MINPP1	multiple inositol-polyphosphate phosphatase 1
0.98	YY1AP1	YY1 associated protein 1
0.98	SPATS2L	Transcript Identified by AceView. Entrez Gene ID(s) 26010
0.98	CCDC160	coiled-coil domain containing 160
0.98	SULF1	sulfatase 1
0.98	LMAN1	lectin. mannose-binding. 1
0.98	SLC30A2	solute carrier family 30 (zinc transporter). member 2
0.98	FOXK1	Transcript Identified by AceView. Entrez Gene ID(s) 221937
0.98	BATF2	basic leucine zipper transcription factor. ATF-like 2
0.98	CNRIP1	cannabinoid receptor interacting protein 1
0.98	TBRG1	transforming growth factor beta regulator 1
0.98	ARL9	ADP-ribosylation factor like GTPase 9
0.98	RAPGEF6	Rap guanine nucleotide exchange factor 6
0.98	RBMXL3	RNA binding motif protein. X-linked-like 3
0.98	TUBA8	tubulin. alpha 8
0.98	PRSS42	protease. serine 42
0.98	PRAMEF10	PRAME family member 10
0.98	HMBS	hydroxymethylbilane synthase
0.98	RAB9B	RAB9B. member RAS oncogene family
0.98	ATXN10	ataxin 10

0.98	KY	kyphoscoliosis peptidase
0.98	SKAP1	src kinase associated phosphoprotein 1
0.98	MAGEA5	MAGE family member A5
0.98	SPATA4	spermatogenesis associated 4
0.98	ZCCHC12	zinc finger. CCHC domain containing 12
0.98	NACA	nascent polypeptide-associated complex alpha subunit
0.98	OR4M1	olfactory receptor. family 4. subfamily M. member 1
0.98	GBP7	guanylate binding protein 7
0.98	CLECL1	C-type lectin-like 1
0.98	CYP2U1	cytochrome P450. family 2. subfamily U. polypeptide 1
0.98	MKL1	megakaryoblastic leukemia (translocation) 1
0.98	PNPLA2	patatin-like phospholipase domain containing 2
0.98	LCE1B	late cornified envelope 1B
0.98	C1QTNF9B	C1q and tumor necrosis factor related protein 9B
0.98	XAGE1B; XAGE1E	X antigen family. member 1B; X antigen family. member 1E
0.98	GLRA1	glycine receptor alpha 1
0.98	SMCO3	single-pass membrane protein with coiled-coil domains 3
0.98	SLC5A7	solute carrier family 5 (sodium/choline cotransporter). member 7
0.98	CFAP99	cilia and flagella associated protein 99
0.98	CTRB2	chymotrypsinogen B2
0.98	CLPSL1	colipase-like 1
0.98	CAMTA2	calmodulin binding transcription activator 2
0.98	CRKL	v-crk avian sarcoma virus CT10 oncogene homolog-like
0.98	SLC35C1	solute carrier family 35 (GDP-fucose transporter). member C1
0.98	KLK5	kallikrein related peptidase 5
0.98	FMN1	formin 1

0.98	RRAGA	Ras-related GTP binding A
0.98	SERF2	small EDRK-rich factor 2
0.98	TBC1D4	TBC1 domain family. member 4
0.98	APOL5	apolipoprotein L. 5
0.98	FMN1	formin 1
0.98	FLT4	fms-related tyrosine kinase 4
0.98	CAMK2G	calcium/calmodulin-dependent protein kinase II gamma
0.98	ARSH	arylsulfatase family. member H
0.98	BRK1	BRICK1. SCAR/WAVE actin-nucleating complex subunit
0.98	TRIM69	tripartite motif containing 69
0.98	SDHAF2	succinate dehydrogenase complex assembly factor 2
0.98	OAZ1	Memczak2013 ALT_ACCEPTOR. ALT_DONOR. coding. INTERNAL. intronic best transcript NM_004152
0.98	OMG	oligodendrocyte myelin glycoprotein
0.98	FGG	fibrinogen gamma chain
0.98	NAAA	N-acylethanolamine acid amidase
0.98	LRRC10	leucine rich repeat containing 10
0.98	SLC32A1	solute carrier family 32 (GABA vesicular transporter). member 1
0.98	SLX1A; SLX1B; SLX1A-SULT1A3; SLX1B-SULT1A4; SULT1A3	SLX1 homolog A. structure-specific endonuclease subunit; SLX1 homolog B. structure-specific endonuclease subunit; SLX1A-SULT1A3 readthrough (NMD candidate); SLX1B-SULT1A4 readthrough (NMD candidate); sulfotransferase family 1A member 3
0.98	DNAJC5; MIR941-1; MIR941-2; MIR941-3; MIR941-4; MIR941-5	DnaJ (Hsp40) homolog. subfamily C. member 5; microRNA 941-1; microRNA 941-2; microRNA 941-3; microRNA 941-4; microRNA 941-5
0.98	TAF8	TATA box binding protein associated factor 8
0.98	TOX3	TOX high mobility group box family member 3
0.98	MIER2	mesoderm induction early response 1. family member 2
0.98	PTPRQ	protein tyrosine phosphatase. receptor type. Q

0.98	TRAPPC4	trafficking protein particle complex 4
0.98	ZNF780A	zinc finger protein 780A
0.98	SPDYE1	speedy/RINGO cell cycle regulator family member E1
0.98	TMEM42; MIR564	transmembrane protein 42; microRNA 564
0.98	CYP24A1	cytochrome P450. family 24. subfamily A. polypeptide 1
0.98	SAE1	SUMO1 activating enzyme subunit 1
0.98	BABAM1	Zhang2013 ALT_ACCEPTOR. ALT_DONOR. coding. INTERNAL. intronic best transcript NM_001033549
0.98	FAM209B	family with sequence similarity 209. member B
0.98	MRPL27	mitochondrial ribosomal protein L27
0.98	AMPD1	adenosine monophosphate deaminase 1
0.98	GSN	gelsolin
0.98	RTP1	receptor (chemosensory) transporter protein 1
0.98	EIF1AY	eukaryotic translation initiation factor 1A. Y-linked
0.98	CBLL1	Cbl proto-oncogene-like 1. E3 ubiquitin protein ligase
0.98	FAM227B	family with sequence similarity 227. member B
0.98	OR5AR1	olfactory receptor. family 5. subfamily AR. member 1 (gene/pseudogene)
0.98	PFAS	phosphoribosylformylglycinamidine synthase
0.98	TIMM50	translocase of inner mitochondrial membrane 50 homolog (S. cerevisiae)
0.98	CNPY1	canopy FGF signaling regulator 1
0.98	SPOCK3	sparc/osteonectin. cwcv and kazal-like domains proteoglycan (testican) 3
0.98	XYLT1	xylosyltransferase I
0.98	APOD	apolipoprotein D
0.98	GYPA	glycophorin A (MNS blood group)
0.98	SCGB1D4	secretoglobin. family 1D. member 4
0.98	PTPRO	Transcript Identified by AceView. Entrez Gene ID(s) 5800

0.98	ATP5B	ATP synthase. H+ transporting. mitochondrial F1 complex. beta polypeptide
0.98	GPR82	G protein-coupled receptor 82
0.98	TNRC6B	Transcript Identified by AceView. Entrez Gene ID(s) 23112
0.98	TTPAL	tocopherol (alpha) transfer protein-like
0.98	NPY2R	neuropeptide Y receptor Y2
0.98	PARL	presenilin associated. rhomboid-like
0.98	MB21D1	Mab-21 domain containing 1
0.98	MNT	MAX network transcriptional repressor
0.98	LURAP1	leucine rich adaptor protein 1
0.98	ZIC1	Zic family member 1
0.98	GLRA2	glycine receptor alpha 2
0.98	GPR171	G protein-coupled receptor 171
0.98	ARMCX3	armadillo repeat containing. X-linked 3
0.98	PKP4	Transcript Identified by AceView. Entrez Gene ID(s) 8502
0.98	MAGEL2	MAGE family member L2
0.98	FBXW4	F-box and WD repeat domain containing 4
0.98	LBX2	ladybird homeobox 2
0.98	ARHGEF19	Rho guanine nucleotide exchange factor 19
0.98	LDOC1	leucine zipper. down-regulated in cancer 1
0.98	SERTAD4	SERTA domain containing 4
0.98	SLCO3A1	solute carrier organic anion transporter family. member 3A1
0.98	SNPH	syntaphilin
0.98	TEC	tec protein tyrosine kinase
0.98	HSH2D	hematopoietic SH2 domain containing
0.98	RAC3	ras-related C3 botulinum toxin substrate 3 (rho family. small GTP binding protein Rac3)

0.98	PARP14	poly(ADP-ribose) polymerase family member 14
0.98	OR2J3	olfactory receptor. family 2. subfamily J. member 3
0.98	ADORA1	adenosine A1 receptor
0.98	LOC100130880; AC022173.2	uncharacterized LOC100130880; novel transcript
0.98	MUSTN1	musculoskeletal. embryonic nuclear protein 1
0.98	C4orf47	chromosome 4 open reading frame 47
0.98	SLC38A10	solute carrier family 38. member 10
0.98	PRDX1	peroxiredoxin 1
0.98	SLC22A6	solute carrier family 22 (organic anion transporter). member 6
0.98	DHRSX	Jeck2013 ALT_ACCEPTOR. ALT_DONOR. coding. INTERNAL. intronic best transcript NM_145177
0.98	EXPH5	exophilin 5
0.98	IGSF10	immunoglobulin superfamily. member 10
0.98	MTOR	mechanistic target of rapamycin (serine/threonine kinase)
0.98	MIP	major intrinsic protein of lens fiber
0.98	RINL	Ras and Rab interactor like
0.98	GAD1	glutamate decarboxylase 1
0.98	TIMM13	translocase of inner mitochondrial membrane 13 homolog (yeast)
0.98	TTC1	tetratricopeptide repeat domain 1
0.98	SAG	S-antigen; retina and pineal gland (arrestin)
0.98	CAPN15; MIR5587	calpain 15; microRNA 5587
0.98	FGF6	fibroblast growth factor 6
0.98	MAP1S	microtubule-associated protein 1S
0.98	DDX10	DEAD (Asp-Glu-Ala-Asp) box polypeptide 10
0.98	ZNF136	zinc finger protein 136
0.98	CYTIP	cytohesin 1 interacting protein

0.98	IRF4	interferon regulatory factor 4
0.98	KAT6B	K(lysine) acetyltransferase 6B
0.98	RHOXF2; RHOXF2B	Rhox homeobox family. member 2; Rhox homeobox family. member 2B
0.98	RPS6KA1	ribosomal protein S6 kinase. 90kDa. polypeptide 1
0.98	C8orf46	chromosome 8 open reading frame 46
0.98	SCP2	sterol carrier protein 2
0.98	PTGDS	prostaglandin D2 synthase 21kDa (brain)
0.98	AGTRAP	angiotensin II receptor-associated protein
0.98	CLSTN1	calsyntenin 1
0.98	ZKSCAN7	zinc finger with KRAB and SCAN domains 7
0.98	CYP2C8	cytochrome P450. family 2. subfamily C. polypeptide 8
0.98	MPPED1	metallophosphoesterase domain containing 1
0.98	boyboy; RP4-630A11.3; LEPR	Transcript Identified by AceView; Jeck2013 ALT_ACCEPTOR. ALT_DONOR. coding. INTERNAL. intronic best transcript NM_001003679; putative novel transcript
0.98	APBA2	amyloid beta (A4) precursor protein-binding. family A. member 2
0.98	GABRA2	gamma-aminobutyric acid (GABA) A receptor. alpha 2
0.98	C8orf74	chromosome 8 open reading frame 74
0.98	SRRM4	serine/arginine repetitive matrix 4
0.98	GUCA1A	guanylate cyclase activator 1A (retina)
0.98	PCDH7	protocadherin 7
0.98	PDCL	phosducin like
0.98	AREL1	apoptosis resistant E3 ubiquitin protein ligase 1
0.98	PLCL2	Memczak2013 ANTISENSE. coding. INTERNAL. intronic best transcript NM_001144382
0.98	FAM53C	family with sequence similarity 53. member C
0.98	SPRYD7	SPRY domain containing 7

0.98	SYNE1	Memczak2013 ALT_ACCEPTOR. ALT_DONOR. coding. INTERNAL. intronic best transcript NM_033071
0.98	C3orf38	chromosome 3 open reading frame 38
0.98	AIM2	absent in melanoma 2
0.98	TNFAIP6	tumor necrosis factor. alpha-induced protein 6
0.98	ARL2	ADP-ribosylation factor like GTPase 2
0.98	MRGPRE	MAS-related GPR. member E
0.98	CLP1; AP000662.4	Transcript Identified by AceView. Entrez Gene ID(s) 10978; novel transcript. sense overlapping CLP1
0.98	IBSP	integrin-binding sialoprotein
0.98	RADIL	Ras association and DIL domains
0.98	NCAM2	Salzman2013 ALT_ACCEPTOR. ALT_DONOR. coding. INTERNAL. intronic best transcript NM_004540
0.98	SLC16A2	solute carrier family 16. member 2 (thyroid hormone transporter)
0.98	CD200; RP11-90K6.1	Transcript Identified by AceView. Entrez Gene ID(s) 4345; novel transcript
0.98	FAM26D	family with sequence similarity 26. member D
0.98	CAPN13	calpain 13
0.98	SEMA3C	sema domain. immunoglobulin domain (Ig). short basic domain. secreted. (semaphorin) 3C
0.98	CCDC34	Transcript Identified by AceView. Entrez Gene ID(s) 91057
0.98	PRYP3	PTPN13-like. Y-linked pseudogene 3
0.98	SPATA31A7; SPATA31A5	SPATA31 subfamily A. member 7; SPATA31 subfamily A. member 5
0.98	GZMH	granzyme H
0.98	HYOU1	hypoxia up-regulated 1
0.98	FBXO9	F-box protein 9
0.98	ADAMTS17	ADAM metallopeptidase with thrombospondin type 1 motif 17
0.98	OR6K3	olfactory receptor. family 6. subfamily K. member 3

0.98	SPIC	Spi-C transcription factor (Spi-1/PU.1 related)
0.98	NR5A1	nuclear receptor subfamily 5. group A. member 1
0.98	TMC3	transmembrane channel like 3
0.98	SGSM2	small G protein signaling modulator 2
0.98	TP53INP1	tumor protein p53 inducible nuclear protein 1
0.98	RPL7A	ribosomal protein L7a
0.98	SMARCA5	SWI/SNF related. matrix associated. actin dependent regulator of chromatin. subfamily a. member 5
0.98	ZNF766; MIR643	zinc finger protein 766; microRNA 643
0.98	RAB43	RAB43. member RAS oncogene family
0.98	FTH1P18	ferritin. heavy polypeptide 1 pseudogene 18
0.98	ABI3	ABI family. member 3
0.98	DTNA	Transcript Identified by AceView. Entrez Gene ID(s) 1837
0.98	ELOVL7	ELOVL fatty acid elongase 7
0.98	STRA13	stimulated by retinoic acid 13
0.98	SPTAN1	spectrin. alpha. non-erythrocytic 1
0.98	ZNF732	zinc finger protein 732
0.98	SLC16A12	solute carrier family 16. member 12
0.98	KIAA1024L	KIAA1024-like
0.98	FBXO38	F-box protein 38
0.98	LINC01276	long intergenic non-protein coding RNA 1276
0.98	C6orf183	chromosome 6 open reading frame 183
0.98	SYT6	synaptotagmin VI
0.98	GSG1L2	GSG1-like 2
0.98	PPP2R2B	protein phosphatase 2. regulatory subunit B. beta
0.98	HNRNPF	heterogeneous nuclear ribonucleoprotein F

0.98	ELFN2	Transcript Identified by AceView. Entrez Gene ID(s) 114794
0.98	CDH8	cadherin 8. type 2
0.98	NWD1	NACHT and WD repeat domain containing 1
0.98	NDUFA2	NADH dehydrogenase (ubiquinone) 1 alpha subcomplex. 2. 8kDa
0.98	CPSF3L; MIR6727	cleavage and polyadenylation specific factor 3-like; microRNA 6727
0.98	RAPGEFL1	Rap guanine nucleotide exchange factor like 1
0.98	CAGE1	cancer antigen 1
0.98	CHST6	carbohydrate (N-acetylglucosamine 6-O) sulfotransferase 6
0.98	SNW1	SNW domain containing 1
0.98	SLC2A13	solute carrier family 2 (facilitated glucose transporter). member 13
0.98	BBS10	Bardet-Biedl syndrome 10
0.98	PIAS1	protein inhibitor of activated STAT 1
0.98	OR2W1	olfactory receptor. family 2. subfamily W. member 1
0.98	C11orf53	chromosome 11 open reading frame 53
0.98	GAS8	Memczak2013 ALT_ACCEPTOR. ALT_DONOR. INTERNAL. intronic. ncRNA best transcript NR_023348
0.98	OR2V2	olfactory receptor. family 2. subfamily V. member 2
0.98	PLEKHF1	pleckstrin homology domain containing. family F (with FYVE domain) member 1
0.98	DNLZ	DNL-type zinc finger
0.98	IGSF5	immunoglobulin superfamily. member 5
0.98	KL	klotho
0.98	TNC	tenascin C
0.98	OR3A2	olfactory receptor. family 3. subfamily A. member 2
0.98	PRAMEF6; PRAMEF5	PRAME family member 6; PRAME family member 5
0.98	RNLS	renalase. FAD-dependent amine oxidase
0.98	GP6	glycoprotein VI (platelet)

0.98	FOXH1	forkhead box H1
0.98	RRAD	Ras-related associated with diabetes
0.98	SLC23A3	solute carrier family 23. member 3
0.98	IMMT	inner membrane protein. mitochondrial
0.98	DCN	decorin
0.98	PCDHB4	protocadherin beta 4
0.98	MYL12A	myosin light chain 12A
0.98	MARK1	MAP/microtubule affinity-regulating kinase 1
0.98	GDF3	growth differentiation factor 3
0.98	CLEC4E	C-type lectin domain family 4. member E
0.98	MRLN	myoregulin
0.98	METTL25	methyltransferase like 25
0.98	CXorf56	chromosome X open reading frame 56
0.98	MOB3B	MOB kinase activator 3B
0.98	FTL	ferritin. light polypeptide
0.98	PRSS53	protease. serine 53
0.98	TRMT61A	tRNA methyltransferase 61A
0.98	FAM86C1	family with sequence similarity 86. member C1
0.98	ZNF444	zinc finger protein 444
0.98	PTPN6	protein tyrosine phosphatase. non-receptor type 6
0.98	ZNF470	zinc finger protein 470
0.98	LIMS3L	LIM and senescent cell antigen-like domains 3-like
0.98	KLF4	Kruppel-like factor 4 (gut)
0.98	USP26	ubiquitin specific peptidase 26
0.98	ATP1A1	ATPase. Na+/K+ transporting. alpha 1 polypeptide
0.98	ATXN7L3	ataxin 7-like 3

0.98	EPHA5	EPH receptor A5
0.98	MEGF10	multiple EGF-like-domains 10
0.98	NPIPB15	nuclear pore complex interacting protein family. member B15
0.98	PTPN2	Transcript Identified by AceView. Entrez Gene ID(s) 5771
0.98	MYH9	Memczak2013 ANTISENSE. CDS. coding. INTERNAL best transcript NM_002473
0.98	CFAP73	cilia and flagella associated protein 73
0.98	RPLP0	ribosomal protein. large. P0
0.98	RGPD1; RGPD2	RANBP2-like and GRIP domain containing 1; RANBP2-like and GRIP domain containing 2
0.98	KDM4E	lysine (K)-specific demethylase 4E
0.98	AADACL3	arylacetamide deacetylase-like 3
0.98	OR5T2	olfactory receptor. family 5. subfamily T. member 2
0.98	TAAR6	trace amine associated receptor 6
0.98	MYCBP2	MYC binding protein 2. E3 ubiquitin protein ligase
0.98	AIFM2	Memczak2013 ANTISENSE. coding. INTERNAL. intronic best transcript NM_001198696
0.98	KRTAP13-4	keratin associated protein 13-4
0.98	HMBS	hydroxymethylbilane synthase
0.98	SLC9A3	solute carrier family 9. subfamily A (NHE3. cation proton antiporter 3). member 3
0.98	ATP5EP2	ATP synthase. H+ transporting. mitochondrial F1 complex. epsilon subunit pseudogene 2
0.98	TTC16	tetratricopeptide repeat domain 16
0.98	ODF1	outer dense fiber of sperm tails 1
0.98	LRRC39	leucine rich repeat containing 39
0.98	PLA2R1	phospholipase A2 receptor 1
0.98	DGKD	diacylglycerol kinase. delta 130kDa

0.98	MAOA	monoamine oxidase A
0.98	ZNF3	zinc finger protein 3
0.98	SLITRK3	SLIT and NTRK-like family. member 3
0.98	C4orf36	chromosome 4 open reading frame 36
0.98	GTPBP1	GTP binding protein 1
0.98	STOML2	stomatin (EPB72)-like 2
0.98	CPS1	carbamoyl-phosphate synthase 1
0.98	PDCD6	programmed cell death 6
0.98	GPR34	G protein-coupled receptor 34
0.98	NRSN1	neurensin 1
0.98	FICD	FIC domain containing
0.98	CREBZF	CREB/ATF bZIP transcription factor
0.98	HPDL	4-hydroxyphenylpyruvate dioxygenase-like
0.98	INPP5D	inositol polyphosphate-5-phosphatase D
0.98	RASL12	RAS-like. family 12
0.98	MATN4	matrilin 4
0.98	ASCC2	activating signal cointegrator 1 complex subunit 2
0.98	ATP11A	ATPase. class VI. type 11A
0.98	ATG16L2	autophagy related 16-like 2
0.98	PPM1E	protein phosphatase. Mg2+/Mn2+ dependent. 1E
0.98	CPD	carboxypeptidase D
0.98	BCL2L14	BCL2-like 14 (apoptosis facilitator)
0.98	KLHL7	kelch-like family member 7
0.98	UQCRFS1	ubiquinol-cytochrome c reductase. Rieske iron-sulfur polypeptide 1
0.98	EPHA4	EPH receptor A4
0.98	PDCD1	programmed cell death 1

0.98	SERPINI2	serpin peptidase inhibitor. clade I (pancpin). member 2
0.98	LIPH	lipase. member H
0.98	WDR86	WD repeat domain 86
0.98	BMP3	bone morphogenetic protein 3
0.98	ZKSCAN7	zinc finger with KRAB and SCAN domains 7
0.98	CACNA1H	calcium channel. voltage-dependent. T type. alpha 1H subunit
0.98	IGDCC4	immunoglobulin superfamily. DCC subclass. member 4
0.98	TPO	thyroid peroxidase
0.98	TSPY10	testis specific protein. Y-linked 10
0.98	WDR60	WD repeat domain 60
0.98	CEACAM6	carcinoembryonic antigen-related cell adhesion molecule 6 (non-specific cross reacting antigen)
0.98	PSTPIP2	proline-serine-threonine phosphatase interacting protein 2
0.98	ZNF668	zinc finger protein 668
0.98	RFX8	RFX family member 8. lacking RFX DNA binding domain
0.98	NBEAL2	neurobeachin like 2
0.98	GPR141	G protein-coupled receptor 141
0.98	GLRA3	glycine receptor alpha 3
0.98	MARCH4	membrane associated ring finger 4
0.98	MAGT1	magnesium transporter 1
0.98	FGF11	fibroblast growth factor 11
0.98	NTPCR	nucleoside-triphosphatase. cancer-related
0.98	OR11H4	olfactory receptor. family 11. subfamily H. member 4
0.98	ZNF385C	zinc finger protein 385C
0.98	TMEM59L	transmembrane protein 59-like
0.98	CTCF	CCCTC-binding factor (zinc finger protein)

0.98	RANBP17	RAN binding protein 17
0.98	OR8D4	olfactory receptor. family 8. subfamily D. member 4
0.98	PPP1R12C	Memczak2013 ANTISENSE. CDS. coding. INTERNAL best transcript NM_017607
0.98	COPZ2	coatomer protein complex subunit zeta 2
0.98	C7orf43; MIR4658	chromosome 7 open reading frame 43; microRNA 4658
0.98	T	T brachyury transcription factor
0.98	LIN28A	lin-28 homolog A (C. elegans)
0.98	ADAD1	adenosine deaminase domain containing 1
0.98	ZMYND15	zinc finger. MYND-type containing 15
0.98	SPTY2D1	SPT2 chromatin protein domain containing 1
0.98	ZNF614	zinc finger protein 614
0.98	LOC441155	zinc finger CCCH-type domain-containing-like
0.98	KCNG2	potassium channel. voltage gated modifier subfamily G. member 2
0.98	ASAH2	N-acylsphingosine amidohydrolase (non-lysosomal ceramidase) 2
0.98	HYOU1	hypoxia up-regulated 1
0.98	DIDO1	death inducer-obliterator 1
0.98	GRM6	glutamate receptor. metabotropic 6
0.98	ALDH3A1	aldehyde dehydrogenase 3 family. member A1
0.98	TXLNB	taxilin beta
0.98	ELP5	elongator acetyltransferase complex subunit 5
0.98	FAM71E1	family with sequence similarity 71. member E1
0.98	RPS27	ribosomal protein S27
0.98	CACNA1A	calcium channel. voltage-dependent. P/Q type. alpha 1A subunit
0.98	MYLK2	myosin light chain kinase 2
0.98	KCNA4	potassium channel. voltage gated shaker related subfamily A. member 4

0.98	FERD3L	Fer3-like bHLH transcription factor
0.98	WIPF1	WAS/WASL interacting protein family. member 1
0.98	LRRC19	leucine rich repeat containing 19
0.98	PSMA4	proteasome subunit alpha 4
0.98	IP6K1	inositol hexakisphosphate kinase 1
0.98	TCAIM	T cell activation inhibitor. mitochondrial
0.98	CSH2	chorionic somatomammotropin hormone 2
0.98	TRAPPC4	trafficking protein particle complex 4
0.98	TMEM74B	transmembrane protein 74B
0.98	MMP14	matrix metallopeptidase 14 (membrane-inserted)
0.98	KIAA1328	KIAA1328
0.98	C9orf135-AS1	C9orf135 antisense RNA 1 (head to head)
0.98	SLC25A26	solute carrier family 25 (S-adenosylmethionine carrier). member 26
0.98	EPB41L4A-AS2	EPB41L4A antisense RNA 2 (head to head)
0.98	DDX59	DEAD (Asp-Glu-Ala-Asp) box polypeptide 59
0.98	COL23A1	collagen. type XXIII. alpha 1
0.98	PATZ1	POZ (BTB) and AT hook containing zinc finger 1
0.98	VPS9D1	VPS9 domain containing 1
0.98	NANOS2	nanos homolog 2 (Drosophila)
0.98	NR0B1	nuclear receptor subfamily 0. group B. member 1
0.98	ACCSL	1-aminocyclopropane-1-carboxylate synthase (inactive)-like
0.98	TRIM62	tripartite motif containing 62
0.98	DUX4L14; DUX4L15	double homeobox 4 like 14; double homeobox 4 like 15
0.98	SAP25	Sin3A associated protein 25kDa
0.98	ST6GAL1	ST6 beta-galactosamide alpha-2.6-sialyltranferase 1
0.98	BRE	brain and reproductive organ-expressed (TNFRSF1A modulator)

0.98	EXOSC6	exosome component 6
0.98	MAPK9	mitogen-activated protein kinase 9
0.98	ZNF615	zinc finger protein 615
0.98	CD93	CD93 molecule
0.98	C1QL4	complement component 1. q subcomponent-like 4
0.98	S100A9	S100 calcium binding protein A9
0.98	MIR1199	microRNA 1199
0.98	IPO8	importin 8
0.98	POMC	proopiomelanocortin
0.98	CACNG3	calcium channel. voltage-dependent. gamma subunit 3
0.98	VASH2	vasohibin 2
0.98	C15orf57; MRPL42P5	chromosome 15 open reading frame 57; mitochondrial ribosomal protein L42 pseudogene 5
0.98	PIAS4	protein inhibitor of activated STAT 4
0.98	DNAJC9	DnaJ (Hsp40) homolog. subfamily C. member 9
0.98	MT3	metallothionein 3
0.98	PYGM	phosphorylase. glycogen. muscle
0.98	FAM183A	family with sequence similarity 183. member A
0.98	TMEM210	transmembrane protein 210
0.98	SYT12	synaptotagmin XII
0.98	TADA1	transcriptional adaptor 1
0.98	RBCK1	RanBP-type and C3HC4-type zinc finger containing 1
0.98	METTL22	methyltransferase like 22
0.98	FAM168A	family with sequence similarity 168. member A
0.98	VARS2	valyl-tRNA synthetase 2. mitochondrial
0.98	ADAM19	ADAM metallopeptidase domain 19

0.98	DAGLB	diacylglycerol lipase. beta
0.98	RAC2	ras-related C3 botulinum toxin substrate 2 (rho family. small GTP binding protein Rac2)
0.98	KIF2C	kinesin family member 2C
0.98	RFWD3	ring finger and WD repeat domain 3
0.98	TMEM252	transmembrane protein 252
0.98	ROPN1B	rhophilin associated tail protein 1B
0.98	C16orf91	chromosome 16 open reading frame 91
0.98	KANK3	KN motif and ankyrin repeat domains 3
0.98	ERICH3	glutamate rich 3
0.98	PHF20L1	PHD finger protein 20-like 1
0.98	IL23R	interleukin 23 receptor
0.98	SLFN12	schlafen family member 12
0.98	KLHL35	kelch-like family member 35
0.98	HMOX2	heme oxygenase 2
0.98	TCF7	transcription factor 7 (T-cell specific. HMG-box)
0.98	CLEC4D	C-type lectin domain family 4. member D
0.98	MKNK1	MAP kinase interacting serine/threonine kinase 1
0.98	TNNI3	troponin I type 3 (cardiac)
0.98	GHRH	growth hormone releasing hormone
0.98	BCL2L14	BCL2-like 14 (apoptosis facilitator)
0.98	GAL	galanin/GMAP prepropeptide
0.98	FAM109A	family with sequence similarity 109. member A
0.98	PAK6; BUB1B	p21 protein (Cdc42/Rac)-activated kinase 6; BUB1 mitotic checkpoint serine/threonine kinase B
0.98	DPAGT1	dolichyl-phosphate (UDP-N-acetylglucosamine) N-acetylglucosaminephosphotransferase 1 (GlcNAc-1-P transferase)

0.98	EIF5B	eukaryotic translation initiation factor 5B
0.98	GRM6	glutamate receptor. metabotropic 6
0.98	KCNA3	potassium channel. voltage gated shaker related subfamily A. member 3
0.98	EYA2	EYA transcriptional coactivator and phosphatase 2
0.98	ZNF736	zinc finger protein 736
0.98	PTX4	pentraxin 4. long
0.98	KIAA0196	KIAA0196
0.98	GPATCH8	G-patch domain containing 8
0.98	ELAVL2	ELAV like neuron-specific RNA binding protein 2
0.98	TMEM17	transmembrane protein 17
0.98	CNTROB	centrobin. centrosomal BRCA2 interacting protein
0.98	TRIM9	tripartite motif containing 9
0.98	HOXC12	homeobox C12
0.98	CXCL17	chemokine (C-X-C motif) ligand 17
0.98	ZBED6; ZC3H11A	zinc finger. BED-type containing 6; zinc finger CCCH-type containing 11A
0.98	DSCAML1	Down syndrome cell adhesion molecule like 1
0.98	SNX7	sorting nexin 7
0.98	RIT2	Ras-like without CAAX 2
0.98	PLXNA3	plexin A3
0.98	OR4K1	olfactory receptor. family 4. subfamily K. member 1
0.98	ARHGEF35	Rho guanine nucleotide exchange factor 35
0.98	WIPF2	WAS/WASL interacting protein family. member 2
0.98	TCF4	Memczak2013 ANTISENSE. coding. INTERNAL. intronic best transcript NM_001243234
0.98	UBE2NL	ubiquitin conjugating enzyme E2N-like (gene/pseudogene)
0.98	DUSP16	dual specificity phosphatase 16

0.98	UBA52	ubiquitin A-52 residue ribosomal protein fusion product 1
0.98	BRD4	bromodomain containing 4
0.98	ZNF286A	zinc finger protein 286A
0.98	MEP1B	meprin A. beta
0.98	NCMAP	noncompact myelin associated protein
0.98	MIR1-1HG; MIR133A2	MIR1-1 host gene; microRNA 133a-2
0.98	MED28	mediator complex subunit 28
0.98	B4GALT2	Memczak2013 ANTISENSE. CDS. coding. INTERNAL best transcript NM_001005417
0.98	VAMP4	vesicle associated membrane protein 4
0.98	MTDH	metadherin
0.98	MTBP	MDM2 binding protein
0.98	ASPSCR1	alveolar soft part sarcoma chromosome region. candidate 1
0.98	PRTFDC1	phosphoribosyl transferase domain containing 1
0.98	ABHD6	abhydrolase domain containing 6
0.98	DCP1A	decapping mRNA 1A
0.98	PAPOLG	Transcript Identified by AceView. Entrez Gene ID(s) 64895
0.98	RNF34	ring finger protein 34. E3 ubiquitin protein ligase
0.98	MSS51	MSS51 mitochondrial translational activator
0.98	FGF7	fibroblast growth factor 7
0.98	MOCS1	molybdenum cofactor synthesis 1
0.98	LYZL6	lysozyme-like 6
0.98	C11orf21	chromosome 11 open reading frame 21
0.98	BBS2	Bardet-Biedl syndrome 2
0.98	RAB18	RAB18. member RAS oncogene family
0.98	KRT7	keratin 7. type II

0.98	AIFM1	apoptosis-inducing factor. mitochondrion-associated. 1
0.98	TRAT1	T cell receptor associated transmembrane adaptor 1
0.98	PAX1	paired box 1
0.98	NUS1	NUS1 dehydrodolichyl diphosphate synthase subunit
0.98	PPP2R5B	protein phosphatase 2. regulatory subunit B. beta
0.98	ZNF35	zinc finger protein 35
0.98	DENR	density-regulated protein
0.98	ECT2L	epithelial cell transforming 2 like
0.98	OR6M1	olfactory receptor. family 6. subfamily M. member 1
0.98	NECAB1	N-terminal EF-hand calcium binding protein 1
0.98	FIP1L1	factor interacting with PAPOLA and CPSF1
0.98	PNLIPRP2	pancreatic lipase-related protein 2
0.98	WDR33	WD repeat domain 33
0.98	CCDC54	coiled-coil domain containing 54
0.98	POU3F3	POU class 3 homeobox 3
0.98	ADNP	activity-dependent neuroprotector homeobox
0.98	BTC	betacellulin
0.98	TMEM230	transmembrane protein 230
0.98	TULP2	tubby like protein 2
0.98	IFNLR1	interferon. lambda receptor 1
0.98	OR2F2	olfactory receptor. family 2. subfamily F. member 2
0.98	ZZEF1	zinc finger. ZZ-type with EF-hand domain 1
0.98	KCNB1	potassium channel. voltage gated Shab related subfamily B. member 1
0.98	U2AF2	Memczak2013 ALT_ACCEPTOR. ALT_DONOR. coding. INTERNAL. intronic best transcript NM_001012478
0.98	DNAH6	dynein. axonemal. heavy chain 6

0.98	TACSTD2	tumor-associated calcium signal transducer 2
0.98	ZNF688	zinc finger protein 688
0.98	KMT2B	lysine (K)-specific methyltransferase 2B
0.98	PRSS23	protease. serine. 23
0.98	PPAN-P2RY11; P2RY11; PPAN	PPAN-P2RY11 readthrough; purinergic receptor P2Y. G-protein coupled. 11; peter pan homolog (Drosophila)
0.98	HMBS	hydroxymethylbilane synthase
0.98	FAM53B	Memczak2013 ALT_ACCEPTOR. ALT_DONOR. coding. INTERNAL. intronic best transcript NM_014661
0.98	EPN3	Transcript Identified by AceView. Entrez Gene ID(s) 55040
0.98	TM6SF2	transmembrane 6 superfamily member 2
0.98	RGN	regucalcin
0.98	CCDC124	coiled-coil domain containing 124
0.98	ARPC3	actin related protein 2/3 complex subunit 3
0.97	PIP4K2A	phosphatidylinositol-5-phosphate 4-kinase. type II. alpha
0.97	DEFB121	defensin. beta 121
0.97	SLC39A1	solute carrier family 39 (zinc transporter). member 1
0.97	IRS4	insulin receptor substrate 4
0.97	TK2	thymidine kinase 2. mitochondrial
0.97	PLA2G2D	phospholipase A2. group IID
0.97	FAM221B	family with sequence similarity 221. member B
0.97	CCND3	cyclin D3
0.97	LGR6	leucine-rich repeat containing G protein-coupled receptor 6
0.97	CACNB3	calcium channel. voltage-dependent. beta 3 subunit
0.97	LSMEM2	leucine-rich single-pass membrane protein 2
0.97	RAB39B	RAB39B. member RAS oncogene family
0.97	PLAC1	placenta specific 1

0.97	RNF43	ring finger protein 43
0.97	RRP36	ribosomal RNA processing 36
0.97	PPP1R14B	protein phosphatase 1. regulatory (inhibitor) subunit 14B
0.97	CRNN	cornulin
0.97	RSBN1L	round spermatid basic protein 1-like
0.97	IQCA1	Transcript Identified by AceView. Entrez Gene ID(s) 79781
0.97	RPS6KA1	ribosomal protein S6 kinase. 90kDa. polypeptide 1
0.97	OR6C68	olfactory receptor. family 6. subfamily C. member 68
0.97	S100A5	S100 calcium binding protein A5
0.97	NDUFS1; GCSHP3	NADH dehydrogenase (ubiquinone) Fe-S protein 1. 75kDa (NADH-coenzyme Q reductase); glycine cleavage system protein H (aminomethyl carrier) pseudogene 3
0.97	LRRIQ3	leucine-rich repeats and IQ motif containing 3
0.97	AKIRIN2	akirin 2
0.97	HBP1	HMG-box transcription factor 1
0.97	SNX8; MIR6836	sorting nexin 8; microRNA 6836
0.97	PITX1	paired-like homeodomain 1
0.97	FOXE1	forkhead box E1
0.97	DCAF4L2	DDB1 and CUL4 associated factor 4-like 2
0.97	TMEM229B	transmembrane protein 229B
0.97	LOC400661; AC034110.1	uncharacterized LOC400661; novel transcript
0.97	ZBED6CL	ZBED6 C-terminal like
0.97	GPR150	G protein-coupled receptor 150
0.97	CD1A	CD1a molecule
0.97	GRK5	Memczak2013 ANTISENSE. coding. INTERNAL. intronic best transcript NM_005308
0.97	FGF2	fibroblast growth factor 2 (basic)

0.97	SV2A	synaptic vesicle glycoprotein 2A
0.97	SOCS3	suppressor of cytokine signaling 3
0.97	KCNA1	potassium channel. voltage gated shaker related subfamily A. member 1
0.97	KRT16	keratin 16. type I
0.97	OR6C74	olfactory receptor. family 6. subfamily C. member 74
0.97	GRAMD2	GRAM domain containing 2
0.97	YTHDC2	YTH domain containing 2
0.97	KLHL20	kelch-like family member 20
0.97	GPR63	G protein-coupled receptor 63
0.97	NKAPL	NFKB activating protein-like
0.97	SPATA31A6	SPATA31 subfamily A. member 6
0.97	OR1Q1	olfactory receptor. family 1. subfamily Q. member 1
0.97	ZFP2	ZFP2 zinc finger protein
0.97	CEACAM3	carcinoembryonic antigen-related cell adhesion molecule 3
0.97	DCHS2	dachsous cadherin-related 2
0.97	OR4D6	olfactory receptor. family 4. subfamily D. member 6
0.97	ZNF623	zinc finger protein 623
0.97	FAM160B2	family with sequence similarity 160. member B2
0.97	OR4N4; OR4N3P; OR4M2	olfactory receptor. family 4. subfamily N. member 4; olfactory receptor. family 4. subfamily N. member 3 pseudogene; olfactory receptor. family 4. subfamily M. member 2
0.97	SERINC5	serine incorporator 5
0.97	CNOT6	CCR4-NOT transcription complex subunit 6
0.97	OR9K2	olfactory receptor. family 9. subfamily K. member 2
0.97	NOP10	NOP10 ribonucleoprotein
0.97	C3orf18	chromosome 3 open reading frame 18
0.97	HMX2	H6 family homeobox 2

0.97	C11orf87	chromosome 11 open reading frame 87
0.97	AMOTL1	Memczak2013 ANTISENSE. coding. INTERNAL. UTR3 best transcript NM_130847
0.97	SLN	sarcolipin
0.97	UBTFL1	upstream binding transcription factor. RNA polymerase I-like 1
0.97	WDFY3	WD repeat and FYVE domain containing 3
0.97	NOX5; SPESP1	NADPH oxidase. EF-hand calcium binding domain 5; sperm equatorial segment protein 1
0.97	RAD51C	RAD51 paralog C
0.97	PROCA1	protein interacting with cyclin A1
0.97	SNRNP48	small nuclear ribonucleoprotein. U11/U12 48KDa subunit
0.97	CATSPERG	catsper channel auxiliary subunit gamma
0.97	CAMSAP3	calmodulin regulated spectrin-associated protein family. member 3
0.97	TRIP6; MIR6875	thyroid hormone receptor interactor 6; microRNA 6875
0.97	LPCAT1	lysophosphatidylcholine acyltransferase 1
0.97	CDC23	cell division cycle 23
0.97	GJD3	gap junction protein delta 3
0.97	RBM12B	RNA binding motif protein 12B
0.97	ABHD16A	abhydrolase domain containing 16A
0.97	NPIPA8	nuclear pore complex interacting protein family. member A8
0.97	CLCA1	chloride channel accessory 1
0.97	ABCA4	ATP binding cassette subfamily A member 4
0.97	TBC1D15	TBC1 domain family. member 15
0.97	CGGBP1	CGG triplet repeat binding protein 1
0.97	GATAD2A	Jeck2013 ALT_ACCEPTOR. ALT_DONOR. coding. INTERNAL. intronic best transcript NM_017660
0.97	RNF182	ring finger protein 182

0.97	FBXO46	F-box protein 46
0.97	DUX4L7	double homeobox 4 like 7
0.97	KRI1	KRI1 homolog
0.97	MKRN2OS	MKRN2 opposite strand
0.97	USP39	ubiquitin specific peptidase 39
0.97	HDGFL1	hepatoma derived growth factor-like 1
0.97	VAMP7	Transcript Identified by AceView. Entrez Gene ID(s) 6845
0.97	TFDP3	transcription factor Dp family. member 3
0.97	ASRGL1	asparaginase like 1
0.97	CHST2	carbohydrate (N-acetylglucosamine-6-O) sulfotransferase 2
0.97	TMSB15B	thymosin beta 15B
0.97	CDH23	cadherin-related 23
0.97	NDUFC2-KCTD14; NDUFC2; KCTD14	NDUFC2-KCTD14 readthrough; NADH dehydrogenase (ubiquinone) 1. subcomplex unknown. 2. 14.5kDa; potassium channel tetramerization domain containing 14
0.97	IKZF2	IKAROS family zinc finger 2
0.97	YTHDF1	YTH N(6)-methyladenosine RNA binding protein 1
0.97	FMN1	formin 1
0.97	LUZP6; MTPN	leucine zipper protein 6; myotrophin
0.97	EVC	Ellis van Creveld protein
0.97	KRT37	keratin 37. type I
0.97	SETD7	SET domain containing (lysine methyltransferase) 7
0.97	ANKRD36B	ankyrin repeat domain 36B
0.97	ZBTB16	zinc finger and BTB domain containing 16
0.97	TNKS	tankyrase. TRF1-interacting ankyrin-related ADP-ribose polymerase
0.97	BIN2	bridging integrator 2
0.97	NUDT14	nudix hydrolase 14

0.97	MFNG	MFNG O-fucosylpeptide 3-beta-N-acetylglucosaminyltransferase
0.97	ST3GAL3; MIR6079	ST3 beta-galactoside alpha-2.3-sialyltransferase 3; microRNA 6079
0.97	ZNF445	zinc finger protein 445
0.97	TAAR3	trace amine associated receptor 3 (gene/pseudogene)
0.97	RNF212	ring finger protein 212
0.97	TRPS1	Transcript Identified by AceView. Entrez Gene ID(s) 7227
0.97	NIPAL3	NIPA-like domain containing 3
0.97	BST1	bone marrow stromal cell antigen 1
0.97	BORCS7-ASMT	BORCS7-ASMT readthrough (NMD candidate)
0.97	ZBED1	zinc finger. BED-type containing 1
0.97	COMP	cartilage oligomeric matrix protein
0.97	OR52N1	olfactory receptor. family 52. subfamily N. member 1
0.97	TSPAN11	tetraspanin 11
0.97	NCOR1	nuclear receptor corepressor 1
0.97	MITF	microphthalmia-associated transcription factor
0.97	CRLS1	cardiolipin synthase 1
0.97	LIPJ	lipase. family member J
0.97	TMEM39A	transmembrane protein 39A
0.97	HAGHL	hydroxyacylglutathione hydrolase-like
0.97	LOXL3	lysyl oxidase-like 3
0.97	CCNO	cyclin O
0.97	RABIF	RAB interacting factor
0.97	TTLL6	tubulin tyrosine ligase-like family member 6
0.97	ADIRF-AS1; RP11-96C23.15; MMRN2	ADIRF antisense RNA 1 [Source:HGNC Symbol;Acc:HGNC:45127]; Transcript Identified by AceView. Entrez Gene ID(s) 79812; ADIRF antisense RNA 1; novel transcript. antisense to ADIRF
0.97	ATG5	autophagy related 5

0.97	HMBS	hydroxymethylbilane synthase
0.97	CD209	CD209 molecule
0.97	CDK14	cyclin-dependent kinase 14
0.97	RAB2A	RAB2A. member RAS oncogene family
0.97	STX19	syntaxin 19
0.97	OR2T34	olfactory receptor. family 2. subfamily T. member 34
0.97	ZNF630	zinc finger protein 630
0.97	ZC3H12A; MIR6732	zinc finger CCCH-type containing 12A; microRNA 6732
0.97	MOK	MOK protein kinase
0.97	HS6ST3	heparan sulfate 6-O-sulfotransferase 3
0.97	GLIPR2	GLI pathogenesis-related 2
0.97	C11orf97	chromosome 11 open reading frame 97
0.97	OR10Z1	olfactory receptor. family 10. subfamily Z. member 1
0.97	SLC26A11	solute carrier family 26 (anion exchanger). member 11
0.97	STAM2	signal transducing adaptor molecule (SH3 domain and ITAM motif) 2
0.97	WDHD1	WD repeat and HMG-box DNA binding protein 1
0.97	EFR3B	EFR3 homolog B
0.97	SERPINE3	serpin peptidase inhibitor. clade E (nexin. plasminogen activator inhibitor type 1). member 3
0.97	WASH1	WAS protein family homolog 1
0.97	BCL11B	B-cell CLL/lymphoma 11B (zinc finger protein)
0.97	PRAMEF12	PRAME family member 12
0.97	SREK1IP1	SREK1-interacting protein 1
0.97	IGLON5	IgLON family member 5
0.97	ARMCX6	armadillo repeat containing. X-linked 6
0.97	ANKRD33B	ankyrin repeat domain 33B

0.97	CES3; CES2	carboxylesterase 3; carboxylesterase 2
0.97	OR52L1	olfactory receptor. family 52. subfamily L. member 1
0.97	RPS6KC1	ribosomal protein S6 kinase. 52kDa. polypeptide 1
0.97	ARPP21	Transcript Identified by AceView. Entrez Gene ID(s) 10777
0.97	IGSF9	immunoglobulin superfamily. member 9
0.97	ZMAT4	zinc finger. matrin-type 4
0.97	THAP11	THAP domain containing 11
0.97	NRG1	neuregulin 1
0.97	CYP21A1P; CYP21A2	cytochrome P450. family 21. subfamily A. polypeptide 1 pseudogene; cytochrome P450. family 21. subfamily A. polypeptide 2
0.97	CTDNEP1	CTD nuclear envelope phosphatase 1
0.97	CCDC169	coiled-coil domain containing 169
0.97	PPHLN1	periphilin 1
0.97	TAAR1	trace amine associated receptor 1
0.97	CYP2C19	cytochrome P450. family 2. subfamily C. polypeptide 19
0.97	C2orf16	chromosome 2 open reading frame 16
0.97	OR52H1	olfactory receptor. family 52. subfamily H. member 1
0.97	POGZ	Transcript Identified by AceView. Entrez Gene ID(s) 23126
0.97	ZCCHC24	zinc finger. CCHC domain containing 24
0.97	GNG2	guanine nucleotide binding protein (G protein). gamma 2
0.97	GALR3	galanin receptor 3
0.97	NARS2	asparaginyl-tRNA synthetase 2. mitochondrial (putative)
0.97	ZNF514	zinc finger protein 514
0.97	MUC13	mucin 13. cell surface associated
0.97	RBBP9	retinoblastoma binding protein 9
0.97	STK11	serine/threonine kinase 11

0.97	OTOF	otoferlin
0.97	MUC5B	mucin 5B. oligomeric mucus/gel-forming
0.97	PLEKHG1	pleckstrin homology domain containing. family G (with RhoGef domain) member 1
0.97	IP6K2	inositol hexakisphosphate kinase 2
0.97	CRLF2	cytokine receptor-like factor 2
0.97	FOXI1	forkhead box I1
0.97	B3GAT2	beta-1.3-glucuronyltransferase 2
0.97	CNGA3	cyclic nucleotide gated channel alpha 3
0.97	LRFN3	leucine rich repeat and fibronectin type III domain containing 3
0.97	TRAPPC3	trafficking protein particle complex 3
0.97	LAMTOR2	late endosomal/lysosomal adaptor. MAPK and MTOR activator 2
0.97	CAPZB	capping protein (actin filament) muscle Z-line. beta
0.97	STRN4	striatin. calmodulin binding protein 4
0.97	IL12RB2	interleukin 12 receptor. beta 2
0.97	PACSIN1	protein kinase C and casein kinase substrate in neurons 1
0.97	ZNF546	zinc finger protein 546
0.97	SLC35F4	solute carrier family 35. member F4
0.97	TBL3	transducin (beta)-like 3
0.97	FTO	fat mass and obesity associated
0.97	TSPAN32	tetraspanin 32
0.97	PLEKHN1	pleckstrin homology domain containing. family N member 1
0.97	SYNC	syncoilin. intermediate filament protein
0.97	ZBTB2	zinc finger and BTB domain containing 2
0.97	P2RX4	purinergic receptor P2X. ligand gated ion channel. 4
0.97	C1orf95	chromosome 1 open reading frame 95

0.97	DIRAS1	DIRAS family. GTP-binding RAS-like 1
0.97	OR14A16	olfactory receptor. family 14. subfamily A. member 16
0.97	UPF3A	UPF3 regulator of nonsense transcripts homolog A (yeast)
0.97	RAB6A	RAB6A. member RAS oncogene family
0.97	ARR3	arrestin 3. retinal (X-arrestin)
0.97	TEX101	testis expressed 101
0.97	PSMD6	proteasome 26S subunit. non-ATPase 6
0.97	MPO	myeloperoxidase
0.97	POGZ	Transcript Identified by AceView. Entrez Gene ID(s) 23126
0.97	GC	group-specific component (vitamin D binding protein)
0.97	OR5AN1	olfactory receptor. family 5. subfamily AN. member 1
0.97	CDR1	cerebellar degeneration related protein 1
0.97	DOC2A	double C2-like domains. alpha
0.97	RPS6KA1	ribosomal protein S6 kinase. 90kDa. polypeptide 1
0.97	ASXL1	additional sex combs like transcriptional regulator 1
0.97	HOXD10	homeobox D10
0.97	SURF4	surfeit 4
0.97	GPR161	G protein-coupled receptor 161
0.97	MIIP	Transcript Identified by AceView. Entrez Gene ID(s) 60672
0.97	EMX1	empty spiracles homeobox 1
0.97	KCNK1	potassium channel. two pore domain subfamily K. member 1
0.97	MAPK4	mitogen-activated protein kinase 4
0.97	CFAP61	cilia and flagella associated protein 61
0.97	XKR4	X-linked Kx blood group related 4
0.97	SLC4A9	solute carrier family 4. sodium bicarbonate cotransporter. member 9
0.97	DAPK2	death-associated protein kinase 2

0.97	SLC9B1	solute carrier family 9. subfamily B (NHA1. cation proton antiporter 1). member 1
0.97	DERA	deoxyribose-phosphate aldolase (putative)
0.97	MANBAL	mannosidase. beta A. lysosomal-like
0.97	AMELX	amelogenin. X-linked
0.97	NEK10	NIMA-related kinase 10
0.97	C6orf223	chromosome 6 open reading frame 223
0.97	PCDHB12	protocadherin beta 12
0.97	RAB3D	RAB3D. member RAS oncogene family
0.97	HEATR3	HEAT repeat containing 3
0.97	LINC00473	long intergenic non-protein coding RNA 473
0.97	FAM168B	family with sequence similarity 168. member B
0.97	DSG4	desmoglein 4
0.97	IL1A	interleukin 1 alpha
0.97	CARD14	caspase recruitment domain family. member 14
0.97	EPYC	epiphycan
0.97	ZBTB25	zinc finger and BTB domain containing 25
0.97	LYRM5	Memczak2013 ANTISENSE. coding. INTERNAL. intronic best transcript NM_001001660
0.97	GRIA1	glutamate receptor. ionotropic. AMPA 1
0.97	HERPUD1	homocysteine-inducible. endoplasmic reticulum stress-inducible. ubiquitin-like domain member 1
0.97	NDUFS2	NADH dehydrogenase (ubiquinone) Fe-S protein 2. 49kDa (NADH-coenzyme Q reductase)
0.97	GLMP	glycosylated lysosomal membrane protein
0.97	MIS12	MIS12 kinetochore complex component
0.97	IKBKE	inhibitor of kappa light polypeptide gene enhancer in B-cells. kinase epsilon

0.97	TMEM176A	transmembrane protein 176A
0.97	KRT25	keratin 25. type I
0.97	ZDHHC3	zinc finger. DHHC-type containing 3
0.97	WISP2	WNT1 inducible signaling pathway protein 2
0.97	AMER3	APC membrane recruitment protein 3
0.97	MYO3B	myosin IIIB
0.97	FKBP1A; MIR6869	FK506 binding protein 1A; microRNA 6869
0.97	LAMTOR3	late endosomal/lysosomal adaptor. MAPK and MTOR activator 3
0.97	OR6B3	olfactory receptor. family 6. subfamily B. member 3
0.97	SPATA9	spermatogenesis associated 9
0.97	IFNL1	interferon. lambda 1
0.97	RNF222	ring finger protein 222
0.97	GRB2	growth factor receptor bound protein 2
0.97	P3H2	prolyl 3-hydroxylase 2
0.97	RPS6KA1	ribosomal protein S6 kinase. 90kDa. polypeptide 1
0.97	NEU4	sialidase 4
0.97	NSUN6	NOP2/Sun domain family. member 6
0.97	PTS	6-pyruvoyltetrahydropterin synthase
0.97	DHX38	DEAH (Asp-Glu-Ala-His) box polypeptide 38
0.97	ADGRG3	adhesion G protein-coupled receptor G3
0.97	SLC17A9	solute carrier family 17 (vesicular nucleotide transporter). member 9
0.97	SPACA7	sperm acrosome associated 7
0.97	CASP16P	caspase 16. pseudogene
0.97	SMIM9	small integral membrane protein 9
0.97	KIAA1324	KIAA1324
0.97	HMX3	H6 family homeobox 3

0.97	GAD2	glutamate decarboxylase 2
0.97	IREB2	iron responsive element binding protein 2
0.97	RIMKLA	ribosomal modification protein rimK-like family member A
0.97	UBA7	Memczak2013 ANTISENSE. coding. INTERNAL. intronic best transcript NM_003335
0.97	OR52K1	olfactory receptor. family 52. subfamily K. member 1
0.97	ANKRD27	ankyrin repeat domain 27 (VPS9 domain)
0.97	HMGCLL1	3-hydroxymethyl-3-methylglutaryl-CoA lyase-like 1
0.97	ST8SIA1	ST8 alpha-N-acetyl-neuraminide alpha-2.8-sialyltransferase 1
0.97	CCDC167	coiled-coil domain containing 167
0.97	ADAM10	ADAM metallopeptidase domain 10
0.97	TRIM17	tripartite motif containing 17
0.97	HS6ST2	heparan sulfate 6-O-sulfotransferase 2
0.97	HLA-L	major histocompatibility complex. class I. L (pseudogene)
0.97	RAD51L3-RFFL	RAD51L3-RFFL readthrough; HCG2039718. isoform CRA_g; Uncharacterized protein [Source:UniProtKB/TrEMBL;Acc:K7EN88]
0.97	SCGB1C2	secretoglobin. family 1C. member 2
0.97	NINJ1	ninjurin 1
0.97	CKM	creatine kinase. muscle
0.97	HIRIP3	HIRA interacting protein 3
0.97	VAC14	Vac14 homolog (S. cerevisiae)
0.97	MIB1	mindbomb E3 ubiquitin protein ligase 1
0.97	VEPH1	ventricular zone expressed PH domain containing 1
0.97	TSSC1	tumor suppressing subtransferable candidate 1
0.97	MICAL1	Jeck2013 ANTISENSE. coding. INTERNAL. intronic best transcript NM_022765
0.97	SLAMF8	SLAM family member 8
0.97	MICU2	mitochondrial calcium uptake 2

0.97	RPS6KA1	ribosomal protein S6 kinase. 90kDa. polypeptide 1
0.97	ELOF1	ELF1 homolog. elongation factor 1
0.97	ANKRD20A5P; RHOT1P1	ankyrin repeat domain 20 family. member A5. pseudogene; ras homolog family member T1 pseudogene 1
0.97	TFR2	transferrin receptor 2
0.97	IFI27L1	interferon. alpha-inducible protein 27-like 1
0.97	OR5C1	olfactory receptor. family 5. subfamily C. member 1
0.97	AC009133.17; C16orf54	Memczak2013 ANTISENSE. coding. INTERNAL. intronic best transcript NM_175900; novel transcript. antisense to C16orf54
0.97	ISCA1	iron-sulfur cluster assembly 1
0.97	LCE1C	late cornified envelope 1C
0.97	ITM2A	integral membrane protein 2A
0.97	DCTN6	dynactin 6
0.97	CAPN6	calpain 6
0.97	GPANK1	G-patch domain and ankyrin repeats 1
0.97	ADGRA2	adhesion G protein-coupled receptor A2
0.97	ALK	Transcript Identified by AceView. Entrez Gene ID(s) 238
0.97	FAM71F1	family with sequence similarity 71. member F1
0.97	CHADL	chondroadherin-like
0.97	AMER2	APC membrane recruitment protein 2
0.97	RAP2C	RAP2C. member of RAS oncogene family
0.97	RPUSD2	RNA pseudouridylate synthase domain containing 2
0.97	CAB39	calcium binding protein 39
0.97	GANC	glucosidase. alpha; neutral C
0.97	PDLIM5	PDZ and LIM domain 5
0.97	SLC4A10	solute carrier family 4. sodium bicarbonate transporter. member 10
0.97	MUL1	mitochondrial E3 ubiquitin protein ligase 1

0.97	IGFALS	insulin-like growth factor binding protein. acid labile subunit
0.97	DOK5	docking protein 5
0.97	C2orf47	chromosome 2 open reading frame 47
0.97	COL9A2	collagen. type IX. alpha 2
0.97	DCAF4	DDB1 and CUL4 associated factor 4
0.97	ZNF780B	zinc finger protein 780B
0.97	ZNF780B	zinc finger protein 780B
0.97	CDK5R2	cyclin-dependent kinase 5. regulatory subunit 2 (p39)
0.97	ANKS1B	ankyrin repeat and sterile alpha motif domain containing 1B
0.97	CORO1B	coronin. actin binding protein. 1B
0.97	ADAMTS13	ADAM metallopeptidase with thrombospondin type 1 motif 13
0.97	AQP5	aquaporin 5
0.97	PPM1M	protein phosphatase. Mg2+/Mn2+ dependent. 1M
0.97	ANKRD52	Zhang2013 ALT_ACCEPTOR. ALT_DONOR. coding. INTERNAL. intronic best transcript NM_173595
0.97	BTLA	B and T lymphocyte associated
0.97	PCDHB15	protocadherin beta 15
0.97	KCNJ14	potassium channel. inwardly rectifying subfamily J. member 14
0.97	SGSM1	small G protein signaling modulator 1
0.97	ARID1A	AT rich interactive domain 1A (SWI-like)
0.97	BRSK1	BR serine/threonine kinase 1
0.97	ZNF689	zinc finger protein 689
0.97	TNPO2	transportin 2
0.97	ELF1	Memczak2013 ALT_ACCEPTOR. ALT_DONOR. coding. INTERNAL. intronic best transcript NM_172373
0.97	DUSP27	dual specificity phosphatase 27 (putative)
0.97	RFWD2	ring finger and WD repeat domain 2. E3 ubiquitin protein ligase

0.97	DGCR5; DGCR9	DiGeorge syndrome critical region gene 5 (non-protein coding); DiGeorge syndrome critical region gene 9 (non-protein coding)
0.97	TOMM70A	translocase of outer mitochondrial membrane 70 homolog A (S. cerevisiae)
0.97	DNAJC8	DnaJ (Hsp40) homolog. subfamily C. member 8
0.97	MPG	N-methylpurine DNA glycosylase
0.97	BHMT2	betaine--homocysteine S-methyltransferase 2
0.97	TSPAN3	tetraspanin 3
0.97	CHMP4B	charged multivesicular body protein 4B
0.97	TRMU	tRNA 5-methylaminomethyl-2-thiouridylate methyltransferase
0.97	CALML5	calmodulin-like 5
0.97	CD14	CD14 molecule
0.97	NCBP2-AS2	NCBP2 antisense RNA 2 (head to head)
0.97	MECP2	methyl-CpG binding protein 2
0.97	RELB	v-rel avian reticuloendotheliosis viral oncogene homolog B
0.97	HMBS	hydroxymethylbilane synthase
0.97	PPP6R1	Memczak2013 ANTISENSE. CDS. coding. INTERNAL best transcript NM_014931
0.97	ENTPD1	ectonucleoside triphosphate diphosphohydrolase 1
0.97	DENND3	DENN/MADD domain containing 3
0.97	WFDC5	WAP four-disulfide core domain 5
0.97	MARK3	MAP/microtubule affinity-regulating kinase 3
0.97	ATOH1	atonal bHLH transcription factor 1
0.97	PSMG3-AS1	PSMG3 antisense RNA 1 (head to head)
0.97	TMEM200C	transmembrane protein 200C
0.97	UPB1	ureidopropionase. beta
0.97	CTSL3P	cathepsin L family member 3. pseudogene
0.97	C10orf128	chromosome 10 open reading frame 128

0.97	PLBD1	Memczak2013 ANTISENSE. coding. INTERNAL. intronic best transcript NM_024829
0.97	LDHC	lactate dehydrogenase C
0.97	ZPBP	zona pellucida binding protein
0.97	GSTA3	glutathione S-transferase alpha 3
0.97	MED7	mediator complex subunit 7
0.97	KIF25-AS1	KIF25 antisense RNA 1
0.97	ZNF233	zinc finger protein 233
0.97	C8orf34	chromosome 8 open reading frame 34
0.97	TMEM11	transmembrane protein 11
0.97	KCNAB1	potassium channel. voltage gated subfamily A regulatory beta subunit 1
0.97	GHRHR	growth hormone releasing hormone receptor
0.97	TLL1	tolloid like 1
0.97	JAM3	junctional adhesion molecule 3
0.97	OR5T1	olfactory receptor. family 5. subfamily T. member 1
0.97	CFHR4	complement factor H-related 4
0.97	NAPRT	nicotinate phosphoribosyltransferase
0.97	FREM2	FRAS1 related extracellular matrix protein 2
0.97	SLC45A4	solute carrier family 45. member 4
0.97	HNRNPA3; MIR4444-2; MIR4444-1	heterogeneous nuclear ribonucleoprotein A3; microRNA 4444-2; microRNA 4444-1
0.97	KAT2A	Memczak2013 ALT_DONOR. coding. INTERNAL. intronic best transcript NM_021078
0.97	ADAMTS20	ADAM metallopeptidase with thrombospondin type 1 motif 20
0.97	PSMA6	proteasome subunit alpha 6
0.97	GNG7	Memczak2013 ALT_ACCEPTOR. ALT_DONOR. coding. INTERNAL. intronic best transcript NM_052847

0.97	NUTM2A	NUT family member 2A
0.97	CXorf58	chromosome X open reading frame 58
0.97	HSF2BP	heat shock transcription factor 2 binding protein
0.97	OR2G6	olfactory receptor. family 2. subfamily G. member 6
0.97	C2orf82	chromosome 2 open reading frame 82
0.97	TTC19	tetratricopeptide repeat domain 19
0.97	EPGN	epithelial mitogen
0.97	PORCN	porcupine homolog (Drosophila)
0.97	ARHGDIB	Rho GDP dissociation inhibitor (GDI) beta
0.97	MMP20	matrix metallopeptidase 20
0.97	LCE3B	late cornified envelope 3B
0.97	DKK2	dickkopf WNT signaling pathway inhibitor 2
0.97	LRRC57	leucine rich repeat containing 57
0.97	SNRPD2	small nuclear ribonucleoprotein D2 polypeptide
0.97	PPP4R4	protein phosphatase 4. regulatory subunit 4
0.97	KRTAP4-3	keratin associated protein 4-3
0.97	CRTAC1	cartilage acidic protein 1
0.97	NMBR	neuromedin B receptor
0.97	GK2	glycerol kinase 2
0.97	TMEM30A	transmembrane protein 30A
0.97	NPB	neuropeptide B
0.97	PDE2A	phosphodiesterase 2A. cGMP-stimulated
0.97	LAGE3	L antigen family. member 3
0.97	TNFAIP2	tumor necrosis factor. alpha-induced protein 2
0.97	CHST11	Transcript Identified by AceView. Entrez Gene ID(s) 50515
0.97	NUBP1	nucleotide binding protein 1

0.97	CTSL	cathepsin L
0.97	OR5I1	olfactory receptor. family 5. subfamily I. member 1
0.97	RAB6C	RAB6C. member RAS oncogene family
0.97	LOC283731; RP11-247C2.2	uncharacterized LOC283731; novel transcript
0.97	CCL4L2; CCL4L1	chemokine (C-C motif) ligand 4-like 2; chemokine (C-C motif) ligand 4-like 1
0.97	IDH3B	isocitrate dehydrogenase 3 (NAD+) beta
0.97	ZNF761; TPM3P9	zinc finger protein 761; tropomyosin 3 pseudogene 9
0.97	ASAP3	ArfGAP with SH3 domain. ankyrin repeat and PH domain 3
0.97	GDI2	GDP dissociation inhibitor 2
0.97	TMSB4Y	thymosin beta 4. Y-linked
0.97	OR2T8	olfactory receptor. family 2. subfamily T. member 8
0.97	USB1	U6 snRNA biogenesis 1
0.97	KCNN1	potassium channel. calcium activated intermediate/small conductance subfamily N alpha. member 1
0.97	HELQ	helicase. POLQ-like
0.97	PCDH12	protocadherin 12
0.97	FGF19	fibroblast growth factor 19
0.97	OR5AL1	olfactory receptor. family 5. subfamily AL. member 1 (gene/pseudogene)
0.97	MFSD6L	major facilitator superfamily domain containing 6-like
0.97	GZMM	granzyme M
0.97	XCL1	chemokine (C motif) ligand 1
0.97	VMP1; MIR21	vacuole membrane protein 1; microRNA 21
0.97	RNF25	ring finger protein 25
0.97	COL1A1	Jeck2013 ANTISENSE. coding. INTERNAL. intronic best transcript NM_000088
0.97	PRSS55	protease. serine. 55
0.97	TMEM154	transmembrane protein 154

0.97	DMRTB1	DMRT-like family B with proline-rich C-terminal. 1
0.97	SLC12A2	solute carrier family 12 (sodium/potassium/chloride transporter). member 2
0.97	ACSF2	acyl-CoA synthetase family member 2
0.97	FTSJ2	FtsJ RNA methyltransferase homolog 2 (E. coli)
0.97	KDM5A	lysine (K)-specific demethylase 5A
0.97	AAMDC	adipogenesis associated. Mth938 domain containing
0.97	FAM179A	family with sequence similarity 179. member A
0.97	CACFD1	calcium channel flower domain containing 1
0.97	TTLL10	tubulin tyrosine ligase-like family member 10
0.97	PTGFR	prostaglandin F receptor (FP)
0.97	CYP2R1	cytochrome P450. family 2. subfamily R. polypeptide 1
0.97	TCIRG1	T-cell. immune regulator 1. ATPase. H+ transporting. lysosomal V0 subunit A3
0.97	SLC37A1	solute carrier family 37 (glucose-6-phosphate transporter). member 1
0.97	TMEM240	transmembrane protein 240
0.97	CCR4	chemokine (C-C motif) receptor 4
0.97	OGN	osteoglycin
0.97	SMNDC1	survival motor neuron domain containing 1
0.97	SERPINB4	serpin peptidase inhibitor. clade B (ovalbumin). member 4
0.97	MFI2	antigen p97 (melanoma associated) identified by monoclonal antibodies 133.2 and 96.5
0.97	XCR1	chemokine (C motif) receptor 1
0.97	ANAPC11	anaphase promoting complex subunit 11
0.97	ADGRL3	adhesion G protein-coupled receptor L3
0.97	KCNK9	potassium channel. two pore domain subfamily K. member 9
0.97	SERF1B; SERF1A	small EDRK-rich factor 1B (centromeric); small EDRK-rich factor 1A (telomeric)

0.97	SERF1A	small EDRK-rich factor 1A (telomeric)
0.97	ITGAV	integrin alpha V
0.97	AQP12B	aquaporin 12B
0.97	MYOZ3	myozenin 3
0.97	LOXL1	lysyl oxidase-like 1
0.97	DLL4	delta-like 4 (Drosophila)
0.97	MCCD1	mitochondrial coiled-coil domain 1
0.97	ELMO3	engulfment and cell motility 3
0.97	IQCF4; IQCF3	IQ motif containing F5 pseudogene; Transcript Identified by AceView. Entrez Gene ID(s) 401067
0.97	EVI5	Transcript Identified by AceView. Entrez Gene ID(s) 7813
0.97	PRAMEF33P	PRAME family member 33. pseudogene
0.97	U2AF1	U2 small nuclear RNA auxiliary factor 1
0.97	C16orf70	chromosome 16 open reading frame 70
0.97	EHMT1	euchromatic histone-lysine N-methyltransferase 1
0.97	ST20	suppressor of tumorigenicity 20
0.97	TXNDC12; KTI12	thioredoxin domain containing 12 (endoplasmic reticulum); KTI12 chromatin associated homolog
0.97	INPP4B	inositol polyphosphate-4-phosphatase type II B
0.97	API5L1	Transcript Identified by AceView. Entrez Gene ID(s) 642812
0.97	ATP2B1	ATPase. Ca++ transporting. plasma membrane 1
0.97	SMCO1	single-pass membrane protein with coiled-coil domains 1
0.97	LEMD3	LEM domain containing 3
0.97	DYNLRB1	dynein. light chain. roadblock-type 1
0.97	CNKSR3	CNKSR family member 3
0.97	SEZ6L	seizure related 6 homolog (mouse)-like
0.97	CCDC94	coiled-coil domain containing 94

0.97	LOC100129697; RP11-830F9.6	uncharacterized LOC100129697; novel transcript. antisense to CBFA2T3
0.97	LOC100631378; AC016582.2; LOC728853	uncharacterized 100631378; Transcript Identified by AceView. Entrez Gene ID(s) 728853; novel transcript
0.97	TRAPPC4	trafficking protein particle complex 4
0.97	CWH43	cell wall biogenesis 43 C-terminal homolog
0.97	DDX4	DEAD (Asp-Glu-Ala-Asp) box polypeptide 4
0.97	RMND5A	required for meiotic nuclear division 5 homolog A
0.97	DPM2	dolichyl-phosphate mannosyltransferase polypeptide 2. regulatory subunit
0.97	TTYH2	tweety family member 2
0.97	SYT9	synaptotagmin IX
0.97	FBXO2	F-box protein 2
0.97	LENG9	leukocyte receptor cluster (LRC) member 9
0.97	EMILIN1	elastin microfibril interfacer 1
0.97	ZNF518B	zinc finger protein 518B
0.97	SERTM1	serine-rich and transmembrane domain containing 1
0.97	C3orf30	chromosome 3 open reading frame 30
0.97	ABO	ABO blood group (transferase A. alpha 1-3-N-acetylgalactosaminyltransferase; transferase B. alpha 1-3-galactosyltransferase) [Source:HGNC Symbol;Acc:HGNC:79]
0.97	JSRP1	junctional sarcoplasmic reticulum protein 1
0.97	CTNND1	catenin (cadherin-associated protein). delta 1
0.97	APLP2	amyloid beta (A4) precursor-like protein 2
0.97	ADAM18	ADAM metallopeptidase domain 18
0.97	PTH1R	parathyroid hormone 1 receptor
0.97	GPR22	G protein-coupled receptor 22
0.97	INTS8	integrator complex subunit 8
0.97	MCF2L2	MCF.2 cell line derived transforming sequence-like 2

0.97	COX17	COX17 cytochrome c oxidase copper chaperone
0.97	CCDC82	coiled-coil domain containing 82
0.97	FBL	fibrillarin
0.97	PLVAP	plasmalemma vesicle associated protein
0.97	OSTN	osteocrin
0.97	SYTL3	synaptotagmin-like 3
0.97	DNAJC7	DnaJ (Hsp40) homolog. subfamily C. member 7
0.97	DENND1C	DENN/MADD domain containing 1C
0.96	TNFSF9	tumor necrosis factor (ligand) superfamily. member 9
0.96	ZNF264	zinc finger protein 264
0.96	NAA25	N(alpha)-acetyltransferase 25. NatB auxiliary subunit
0.96	SYT16	synaptotagmin XVI
0.96	A2ML1	alpha-2-macroglobulin-like 1
0.96	TMEM98	transmembrane protein 98
0.96	EFCAB8	EF-hand calcium binding domain 8
0.96	NTRK1	neurotrophic tyrosine kinase. receptor. type 1
0.96	SH3TC1	SH3 domain and tetratricopeptide repeats 1
0.96	NCBP2L	nuclear cap binding protein subunit 2-like
0.96	FAM83B	family with sequence similarity 83. member B
0.96	PUF60	poly-U binding splicing factor 60KDa
0.96	CCDC12	coiled-coil domain containing 12
0.96	AP2A2	adaptor-related protein complex 2. alpha 2 subunit
0.96	PHKB	Transcript Identified by AceView. Entrez Gene ID(s) 5257
0.96	CXorf49B	chromosome X open reading frame 49B
0.96	CHST11	Transcript Identified by AceView. Entrez Gene ID(s) 50515
0.96	C1orf53	chromosome 1 open reading frame 53

0.96	ADORA2A	adenosine A2a receptor
0.96	DOCK3	dedicator of cytokinesis 3
0.96	DOK3	docking protein 3
0.96	GOLGA6L6	golgin A6 family-like 6
0.96	OR2D3	olfactory receptor. family 2. subfamily D. member 3
0.96	NUP155	nucleoporin 155kDa
0.96	C9orf9	chromosome 9 open reading frame 9
0.96	ETFA	electron-transfer-flavoprotein. alpha polypeptide
0.96	SPNS2	spinster homolog 2 (Drosophila)
0.96	LY6G6E	lymphocyte antigen 6 complex. locus G6E (pseudogene)
0.96	PRAMEF11	PRAME family member 11
0.96	MBTD1	mbt domain containing 1
0.96	ATHL1	ATH1. acid trehalase-like 1 (yeast)
0.96	CD46	CD46 molecule. complement regulatory protein
0.96	FAM71E2	family with sequence similarity 71. member E2
0.96	ATXN7L1	ataxin 7-like 1
0.96	NPY4R; CH17-360D5.1; PPYR1	Homo sapiens neuropeptide Y receptor Y4 (NPY4R). transcript variant 2. mRNA.; neuropeptide Y receptor type 4-like; Homo sapiens pancreatic polypeptide receptor 1. mRNA (cDNA clone MGC:116895 IMAGE:40005502). complete cds.; Homo sapiens pancreatic polypeptide receptor 1. mRNA (cDNA clone MGC:116897 IMAGE:40005506). complete cds.; novel transcript
0.96	COL5A1	collagen. type V. alpha 1
0.96	XKR8	X-linked Kx blood group related 8
0.96	NIPSNAP3A; NIPSNAP3B	nipsnap homolog 3A (C. elegans); nipsnap homolog 3B (C. elegans)
0.96	SPRR2D	small proline-rich protein 2D
0.96	ARX	aristaless related homeobox
0.96	C10orf82	chromosome 10 open reading frame 82

0.96	CCR10	chemokine (C-C motif) receptor 10
0.96	CRISP2	cysteine-rich secretory protein 2
0.96	BCL3; MIR8085	B-cell CLL/lymphoma 3; microRNA 8085
0.96	SLC25A26	solute carrier family 25 (S-adenosylmethionine carrier). member 26
0.96	OSBPL6	oxysterol binding protein-like 6
0.96	KRTAP10-9	keratin associated protein 10-9
0.96	HCK	HCK proto-oncogene. Src family tyrosine kinase
0.96	FOLR3	folate receptor 3 (gamma)
0.96	ZNF576	zinc finger protein 576
0.96	RNF103	ring finger protein 103
0.96	ZNF594	zinc finger protein 594
0.96	KCTD5	potassium channel tetramerization domain containing 5
0.96	PRPH	peripherin
0.96	ATP2B3	ATPase. Ca++ transporting. plasma membrane 3
0.96	VSIG4	V-set and immunoglobulin domain containing 4
0.96	MARCH8	membrane associated ring finger 8
0.96	DNHD1	dynein heavy chain domain 1
0.96	TLDC1	TBC/LysM-associated domain containing 1
0.96	NSD1	nuclear receptor binding SET domain protein 1
0.96	KLF16	Kruppel-like factor 16
0.96	ZNF787	zinc finger protein 787
0.96	SERTAD4	SERTA domain containing 4
0.96	TSPAN8	tetraspanin 8
0.96	ZNF14	zinc finger protein 14
0.96	CHERP	calcium homeostasis endoplasmic reticulum protein
0.96	YWHAZ	tyrosine 3-monooxygenase/tryptophan 5-monooxygenase activation

		protein. zeta
0.96	ANO3	anoctamin 3
0.96	OSBPL1A	oxysterol binding protein-like 1A
0.96	MED19	mediator complex subunit 19
0.96	FERMT3	fermitin family member 3
0.96	CPVL	carboxypeptidase. vitellogenic-like
0.96	FBL	fibrillarin
0.96	POGZ	Transcript Identified by AceView. Entrez Gene ID(s) 23126
0.96	ZNF880	zinc finger protein 880
0.96	RP11-295P9.3; PRPF18	novel transcript; Transcript Identified by AceView. Entrez Gene ID(s) 8559; RP11-295P9.3 (from geneSymbol)
0.96	C12orf57; RNU7-1	chromosome 12 open reading frame 57; RNA. U7 small nuclear 1
0.96	LINC00550	long intergenic non-protein coding RNA 550
0.96	KCTD10	potassium channel tetramerization domain containing 10
0.96	KRTAP5-4	keratin associated protein 5-4
0.96	TIAM1	T-cell lymphoma invasion and metastasis 1
0.96	TULP4	tubby like protein 4
0.96	MID1IP1	MID1 interacting protein 1
0.96	PACS2	phosphofurin acidic cluster sorting protein 2
0.96	DMRTA2	DMRT-like family A2
0.96	SLC39A9	solute carrier family 39. member 9
0.96	DUT	deoxyuridine triphosphatase
0.96	AP3B1	adaptor-related protein complex 3. beta 1 subunit
0.96	RSU1	Ras suppressor protein 1
0.96	HCST	hematopoietic cell signal transducer
0.96	SYCP2	synaptonemal complex protein 2

0.96	QRSL1	Transcript Identified by AceView. Entrez Gene ID(s) 55278
0.96	ANXA9	annexin A9
0.96	CTRB1	chymotrypsinogen B1
0.96	PDE1B	phosphodiesterase 1B. calmodulin-dependent
0.96	ZNF773	zinc finger protein 773
0.96	IL36G	interleukin 36. gamma
0.96	ANGEL1	angel homolog 1 (Drosophila)
0.96	HMBS	hydroxymethylbilane synthase
0.96	RP3-370M22.8; GRAP2	Transcript Identified by AceView. Entrez Gene ID(s) 9402; putative novel transcript
0.96	APPBP2	amyloid beta precursor protein (cytoplasmic tail) binding protein 2
0.96	C19orf54	chromosome 19 open reading frame 54
0.96	YARS	Transcript Identified by AceView. Entrez Gene ID(s) 8565
0.96	CSRP1; markobu	Memczak2013 ALT_ACCEPTOR. ALT_DONOR. coding. INTERNAL. intronic best transcript NM_001193571; Transcript Identified by AceView
0.96	FAM228B	family with sequence similarity 228. member B
0.96	PSPC1	paraspeckle component 1
0.96	SP3	Sp3 transcription factor
0.96	LRRFIP1	leucine rich repeat (in FLII) interacting protein 1
0.96	PRPF18	pre-mRNA processing factor 18
0.96	DUOXA2	dual oxidase maturation factor 2
0.96	AFF1	AF4/FMR2 family. member 1
0.96	PHF11	PHD finger protein 11
0.96	GPR132	G protein-coupled receptor 132
0.96	LOC100130370; RP11-1055B8.3	uncharacterized LOC100130370; novel transcript
0.96	CLUL1	clusterin-like 1 (retinal)
0.96	BZRAP1	benzodiazepine receptor (peripheral) associated protein 1

0.96	CSNK1G2	casein kinase 1. gamma 2
0.96	LRRC46	leucine rich repeat containing 46
0.96	CDC5L	cell division cycle 5-like
0.96	LOC101929319; TNFAIP6	uncharacterized LOC101929319; Salzman2013 ANTISENSE. CDS. coding. INTERNAL. intronic. OVCODE. OVEXON best transcript NM_007115
0.96	TSPY8	testis specific protein. Y-linked 8
0.96	RASGRP4	RAS guanyl releasing protein 4
0.96	CREG1	cellular repressor of E1A-stimulated genes 1
0.96	PIK3AP1	phosphoinositide-3-kinase adaptor protein 1
0.96	GRK1	G protein-coupled receptor kinase 1
0.96	LBH	limb bud and heart development
0.96	ZDHHC5	zinc finger. DHHC-type containing 5
0.96	CDHR4	cadherin-related family member 4
0.96	MSC	musculin
0.96	SAT2	spermidine/spermine N1-acetyltransferase family member 2
0.96	AADAT	aminoadipate aminotransferase
0.96	MPV17	MpV17 mitochondrial inner membrane protein
0.96	FLJ33534; AC062028.1	uncharacterized LOC285150; uncharacterized LOC285150 [Source:EntrezGene;Acc:285150]; novel transcript
0.96	KLK12	kallikrein related peptidase 12
0.96	TM2D3	TM2 domain containing 3
0.96	SHANK2	SH3 and multiple ankyrin repeat domains 2
0.96	RBM26	RNA binding motif protein 26
0.96	SLC25A28	solute carrier family 25 (mitochondrial iron transporter). member 28
0.96	GPAT2	glycerol-3-phosphate acyltransferase 2. mitochondrial
0.96	GOLPH3	golgi phosphoprotein 3 (coat-protein)
0.96	FAM177A1	family with sequence similarity 177. member A1

0.96	ESM1	endothelial cell-specific molecule 1
0.96	RNF146	ring finger protein 146
0.96	C14orf159	chromosome 14 open reading frame 159
0.96	C17orf105	chromosome 17 open reading frame 105
0.96	CASP12	caspase 12 (gene/pseudogene)
0.96	RFPL2	ret finger protein-like 2
0.96	ZNF471	zinc finger protein 471
0.96	LRRC2	leucine rich repeat containing 2
0.96	HERC3	HECT and RLD domain containing E3 ubiquitin protein ligase 3
0.96	PHC3	polyhomeotic homolog 3 (Drosophila)
0.96	AURKC	aurora kinase C
0.96	FBXW8	F-box and WD repeat domain containing 8
0.96	PRAMEF14	PRAME family member 14
0.96	LYZL4	lysozyme-like 4
0.96	CDH3	cadherin 3. type 1. P-cadherin (placental)
0.96	CHTF18	chromosome transmission fidelity factor 18
0.96	TUBGCP2	tubulin. gamma complex associated protein 2
0.96	PIGW	phosphatidylinositol glycan anchor biosynthesis class W
0.96	KCTD2	potassium channel tetramerization domain containing 2
0.96	DRAP1	DR1-associated protein 1 (negative cofactor 2 alpha)
0.96	NDUFV1	NADH dehydrogenase (ubiquinone) flavoprotein 1. 51kDa
0.96	UBE4A	ubiquitination factor E4A
0.96	MFSD2A	major facilitator superfamily domain containing 2A
0.96	TPRG1	tumor protein p63 regulated 1
0.96	THUMPD3-AS1	THUMPD3 antisense RNA 1
0.96	RCOR2	REST corepressor 2

0.96	USHBP1	Usher syndrome 1C binding protein 1
0.96	DCTN2	dynactin 2 (p50)
0.96	CDO1	cysteine dioxygenase type 1
0.96	PICALM	phosphatidylinositol binding clathrin assembly protein
0.96	CACFD1	calcium channel flower domain containing 1
0.96	CFAP206	cilia and flagella associated protein 206
0.96	ULK2	unc-51 like autophagy activating kinase 2
0.96	NUCB1	Memczak2013 ANTISENSE. CDS. coding. INTERNAL best transcript NM_006184
0.96	BAHCC1	BAH domain and coiled-coil containing 1
0.96	COPG1	coatomer protein complex subunit gamma 1
0.96	GLT6D1	glycosyltransferase 6 domain containing 1
0.96	KRTAP9-6	keratin associated protein 9-6
0.96	POSTN	periostin. osteoblast specific factor
0.96	H2AFB3	H2A histone family. member B3
0.96	LIPK	lipase. family member K
0.96	CACFD1	calcium channel flower domain containing 1
0.96	LTC4S	leukotriene C4 synthase
0.96	JAGN1	jagunal homolog 1
0.96	SH3BP5L	SH3-binding domain protein 5-like
0.96	LCP2	lymphocyte cytosolic protein 2
0.96	SLC26A4	solute carrier family 26 (anion exchanger). member 4
0.96	ZBTB7B	zinc finger and BTB domain containing 7B
0.96	RAMP1	receptor (G protein-coupled) activity modifying protein 1
0.96	STAC	SH3 and cysteine rich domain
0.96	SLC38A7	solute carrier family 38. member 7

0.96	EIF2AK2	eukaryotic translation initiation factor 2-alpha kinase 2
0.96	TRAPPC11	trafficking protein particle complex 11
0.96	TRIM43B	tripartite motif containing 43B
0.96	GPR101	G protein-coupled receptor 101
0.96	PNMAL1	paraneoplastic Ma antigen family-like 1
0.96	ABRA	actin binding Rho activating protein
0.96	FOXL2	forkhead box L2
0.96	OR1D5	olfactory receptor. family 1. subfamily D. member 5
0.96	ZSCAN4	zinc finger and SCAN domain containing 4
0.96	SNX4	sorting nexin 4
0.96	BRSK2	BR serine/threonine kinase 2
0.96	CASP6	caspase 6
0.96	MUC4	mucin 4. cell surface associated
0.96	FTCDNL1	formiminotransferase cyclodeaminase N-terminal like
0.96	FATE1	fetal and adult testis expressed 1
0.96	RPLP2; SNORA52	ribosomal protein. large. P2; small nucleolar RNA. H/ACA box 52
0.96	LINC00471	long intergenic non-protein coding RNA 471
0.96	EDDM3B	epididymal protein 3B
0.96	USP17L15	ubiquitin specific peptidase 17-like family member 15
0.96	KRTAP10-1	keratin associated protein 10-1
0.96	TIGD5	tigger transposable element derived 5
0.96	USP34	Transcript Identified by AceView. Entrez Gene ID(s) 9736
0.96	NDST4	N-deacetylase/N-sulfotransferase (heparan glucosaminyl) 4
0.96	DMKN	dermokine
0.96	HRH4	histamine receptor H4
0.96	TM9SF3	transmembrane 9 superfamily member 3

0.96	SAPCD2	suppressor APC domain containing 2
0.96	ARAP2	ArfGAP with RhoGAP domain. ankyrin repeat and PH domain 2
0.96	KRTAP1-3	keratin associated protein 1-3
0.96	GUCY1A2	guanylate cyclase 1. soluble. alpha 2
0.96	TPH1	tryptophan hydroxylase 1
0.96	TEDDM1	transmembrane epididymal protein 1
0.96	CRB2	crumbs family member 2
0.96	FUT4	fucosyltransferase 4 (alpha (1.3) fucosyltransferase. myeloid-specific)
0.96	CRP	C-reactive protein. pentraxin-related
0.96	FBXW12	F-box and WD repeat domain containing 12
0.96	OR5P3	olfactory receptor. family 5. subfamily P. member 3
0.96	TXNL4A	thioredoxin-like 4A
0.96	BDH2	3-hydroxybutyrate dehydrogenase. type 2
0.96	EDC3	enhancer of mRNA decapping 3
0.96	RGS3	regulator of G-protein signaling 3
0.96	OR10W1	olfactory receptor. family 10. subfamily W. member 1
0.96	RALY	RALY heterogeneous nuclear ribonucleoprotein
0.96	SLC17A5	solute carrier family 17 (acidic sugar transporter). member 5
0.96	PKIA	protein kinase (cAMP-dependent. catalytic) inhibitor alpha
0.96	SPATA31A3	SPATA31 subfamily A. member 3
0.96	TRIM6	tripartite motif containing 6
0.96	TPBG	trophoblast glycoprotein
0.96	NLGN2	neuroligin 2
0.96	EEF1E1-BLOC1S5	EEF1E1-BLOC1S5 readthrough (NMD candidate)
0.96	DYRK1B	dual specificity tyrosine-(Y)-phosphorylation regulated kinase 1B
0.96	THEM5	thioesterase superfamily member 5

0.96	LRRC3C	leucine rich repeat containing 3C
0.96	TSHB	thyroid stimulating hormone. beta
0.96	TPP2	tripeptidyl peptidase II
0.96	TSPY3; TSPY4; TSPY8; TSPY1	testis specific protein. Y-linked 3; testis specific protein. Y-linked 4; testis specific protein. Y-linked 8; testis specific protein. Y-linked 1
0.96	HPN-AS1	HPN antisense RNA 1
0.96	ZDHHC3	zinc finger. DHHC-type containing 3
0.96	NBPF1	neuroblastoma breakpoint family. member 1
0.96	SRM	spermidine synthase
0.96	PCDH9	Memczak2013 ALT_ACCEPTOR. ALT_DONOR. coding. INTERNAL. intronic best transcript NM_203487
0.96	DLX2	distal-less homeobox 2
0.96	TRPC7	transient receptor potential cation channel. subfamily C. member 7
0.96	ADAMTS13	ADAM metallopeptidase with thrombospondin type 1 motif 13
0.96	TTYH1	tweety family member 1
0.96	EFCAB6	EF-hand calcium binding domain 6
0.96	ZNF160	zinc finger protein 160
0.96	IL9R	interleukin 9 receptor
0.96	IL9R	Homo sapiens interleukin 9 receptor (IL9R). transcript variant 1. mRNA.; Homo sapiens interleukin 9 receptor (IL9R). transcript variant 2. mRNA.; interleukin 9 receptor [Source:HGNC Symbol;Acc:HGNC:6030]; Homo sapiens interleukin 9 receptor. mRNA (cDNA clone MGC:59852 IMAGE:6304829). complete cds.; Transcript Identified by AceView. Entrez Gene ID(s) 3581. RefSeq ID(s) NM_002186; Transcript Identified by AceView. Entrez Gene ID(s) 3581
0.96	OR2L13	olfactory receptor. family 2. subfamily L. member 13
0.96	TMOD1	tropomodulin 1
0.96	AMY1B; AMY1A	amylase. alpha 1B (salivary); amylase. alpha 1A (salivary)
0.96	FAM110C	family with sequence similarity 110. member C

0.96	SP100	SP100 nuclear antigen
0.96	BRF1	BRF1. RNA polymerase III transcription initiation factor 90 kDa subunit
0.96	CCDC141	coiled-coil domain containing 141
0.96	OTOL1	otolin 1
0.96	TLR9	toll-like receptor 9
0.96	RNF170; MIR4469	ring finger protein 170; microRNA 4469
0.96	SOHLH1	spermatogenesis and oogenesis specific basic helix-loop-helix 1
0.96	PADI1	peptidyl arginine deiminase. type I
0.96	LOC100133091	uncharacterized LOC100133091; Salzman2013 ANNOTATED. INTERNAL. ncRNA. OVEXON best transcript NR_029411; Transcript Identified by AceView. Entrez Gene ID(s) 554248; 100133091. RefSeq ID(s) NR_029411
0.96	HSD17B13	hydroxysteroid (17-beta) dehydrogenase 13
0.96	PCDH11X	Salzman2013 ANTISENSE. coding. INTERNAL. intronic best transcript NM_032968
0.96	GPR37	G protein-coupled receptor 37 (endothelin receptor type B-like)
0.96	NARS	asparaginyl-tRNA synthetase
0.96	SUSD4	sushi domain containing 4
0.96	C22orf24	chromosome 22 open reading frame 24
0.96	ANK2	Transcript Identified by AceView. Entrez Gene ID(s) 287
0.96	HOXB13	homeobox B13
0.96	ATP4B	ATPase. H+/K+ exchanging. beta polypeptide
0.96	JAKMIP1	janus kinase and microtubule interacting protein 1
0.96	CACNA1E	calcium channel. voltage-dependent. R type. alpha 1E subunit
0.96	PPP1R37	protein phosphatase 1. regulatory subunit 37
0.96	CSH1	chorionic somatomammotropin hormone 1 (placental lactogen)
0.96	COX6B2	cytochrome c oxidase subunit VIb polypeptide 2 (testis)
0.96	STIM1; MIR4687	stromal interaction molecule 1; microRNA 4687

0.96	GPR1	G protein-coupled receptor 1
0.96	ABL1	ABL proto-oncogene 1. non-receptor tyrosine kinase
0.96	ATRNL1	attractin-like 1
0.96	MYO1F	Jeck2013 ANTISENSE. CDS. coding. INTERNAL. intronic. OVCODE. OVEXON best transcript NM_012335
0.96	ZNF816	zinc finger protein 816
0.96	NDUFA8	NADH dehydrogenase (ubiquinone) 1 alpha subcomplex. 8. 19kDa
0.96	DNAJC13	DnaJ (Hsp40) homolog. subfamily C. member 13
0.96	NOL4L	nucleolar protein 4-like
0.96	HHLA2	HERV-H LTR-associating 2
0.96	NR2F6	nuclear receptor subfamily 2. group F. member 6
0.96	C2orf88	chromosome 2 open reading frame 88
0.96	AMBN	ameloblastin
0.96	UFSP2	UFM1-specific peptidase 2
0.96	PRKACG	protein kinase. cAMP-dependent. catalytic. gamma
0.96	ALX3	ALX homeobox 3
0.96	EVX1	even-skipped homeobox 1
0.96	PRR7	proline rich 7 (synaptic)
0.96	CARM1	coactivator-associated arginine methyltransferase 1
0.96	NPR1	natriuretic peptide receptor 1
0.96	ELMSAN1	ELM2 and Myb/SANT-like domain containing 1
0.96	MAGI1	membrane associated guanylate kinase. WW and PDZ domain containing 1
0.96	VPS13A	vacuolar protein sorting 13 homolog A (S. cerevisiae)
0.96	KCNQ1	potassium channel. voltage gated KQT-like subfamily Q. member 1
0.96	MDH2	malate dehydrogenase 2
0.96	MRAS	muscle RAS oncogene homolog

0.96	GALNT6	polypeptide N-acetylgalactosaminyltransferase 6
0.96	DNER	delta/notch like EGF repeat containing
0.96	KNCN	kinocilin
0.96	AZGP1	alpha-2-glycoprotein 1. zinc-binding
0.96	VPS39	vacuolar protein sorting 39 homolog (S. cerevisiae)
0.96	NBPF15	neuroblastoma breakpoint family. member 15
0.96	THEMIS2	thymocyte selection associated family member 2
0.96	ENO4	enolase family member 4
0.96	CBWD2	COBW domain containing 2
0.96	SERPINC1	serpin peptidase inhibitor. clade C (antithrombin). member 1
0.96	HSD3B1	hydroxy-delta-5-steroid dehydrogenase. 3 beta- and steroid delta-isomerase 1
0.96	M6PR	mannose-6-phosphate receptor (cation dependent)
0.96	GSTZ1	glutathione S-transferase zeta 1
0.96	SPEG	SPEG complex locus
0.96	WDR87	WD repeat domain 87
0.96	PPIA	peptidylprolyl isomerase A (cyclophilin A)
0.96	GCC1	GRIP and coiled-coil domain containing 1
0.96	FLJ30679; RP11-463O9.8	uncharacterized protein FLJ30679; uncharacterized protein FLJ30679 [Source:EntrezGene;Acc:146512]; novel transcript
0.96	CLDND2	claudin domain containing 2
0.96	MOCS3	molybdenum cofactor synthesis 3
0.96	ARRDC1-AS1	ARRDC1 antisense RNA 1
0.96	UACA	uveal autoantigen with coiled-coil domains and ankyrin repeats
0.96	CLASP1	cytoplasmic linker associated protein 1
0.96	KRTAP17-1	keratin associated protein 17-1
0.96	FOXN3	forkhead box N3

0.96	LRAT	lecithin retinol acyltransferase (phosphatidylcholine--retinol O-acyltransferase)
0.96	HMBS	hydroxymethylbilane synthase
0.96	C22orf34	chromosome 22 open reading frame 34
0.96	ZNF319	zinc finger protein 319
0.96	TMEM246	transmembrane protein 246
0.96	RPSAP58; RPSA	ribosomal protein SA pseudogene 58; ribosomal protein SA
0.96	LRIT2	leucine-rich repeat. immunoglobulin-like and transmembrane domains 2
0.96	DNAAF3	dynein. axonemal. assembly factor 3
0.96	ABCF1	ATP binding cassette subfamily F member 1
0.96	PRR3	proline rich 3
0.96	KCNJ11	potassium channel. inwardly rectifying subfamily J. member 11
0.96	ZNF180	zinc finger protein 180
0.96	GDF2	growth differentiation factor 2
0.96	GALNT14	Memczak2013 ALT_ACCEPTOR. ALT_DONOR. INTERNAL. intronic. ncRNA best transcript NR_045602
0.96	ARHGEF3	Rho guanine nucleotide exchange factor 3
0.96	DOCK11	dedicator of cytokinesis 11
0.96	TOR1B	torsin family 1. member B (torsin B)
0.96	SS18L2	synovial sarcoma translocation gene on chromosome 18-like 2
0.96	PTPRM	protein tyrosine phosphatase. receptor type. M
0.96	TM9SF4	transmembrane 9 superfamily protein member 4
0.96	WDR1	Jeck2013 ANTISENSE. CDS. coding. INTERNAL. intronic. OVCODE. OVEXON best transcript NM_017491
0.96	RLN1	relaxin 1
0.96	CPB1	carboxypeptidase B1 (tissue)
0.96	TGFBR3L	transforming growth factor beta receptor III like

0.96	FSCB	fibrous sheath CABYR binding protein
0.96	MOBP	myelin-associated oligodendrocyte basic protein
0.96	TLN2	talin 2
0.96	CCDC114	coiled-coil domain containing 114
0.96	STARD7	StAR-related lipid transfer domain containing 7
0.96	JDP2	Jun dimerization protein 2
0.96	NHS	Nance-Horan syndrome (congenital cataracts and dental anomalies)
0.96	NAP1L2	nucleosome assembly protein 1-like 2
0.96	HEPACAM	hepatic and glial cell adhesion molecule
0.96	OR5M9	olfactory receptor. family 5. subfamily M. member 9
0.96	DNALI1	dynein. axonemal. light intermediate chain 1
0.96	TMEM173	transmembrane protein 173
0.96	LINC01343	long intergenic non-protein coding RNA 1343
0.96	EIF3F	Eukaryotic translation initiation factor 3 subunit F [Source:UniProtKB/Swiss-Prot;Acc:O00303]
0.96	PRAMEF7	PRAME family member 7
0.96	TIA1	TIA1 cytotoxic granule-associated RNA binding protein
0.96	IFNL4	interferon. lambda 4 (gene/pseudogene)
0.96	OR9A4	olfactory receptor. family 9. subfamily A. member 4
0.96	SEMA5A	sema domain. seven thrombospondin repeats (type 1 and type 1-like). transmembrane domain (TM) and short cytoplasmic domain. (semaphorin) 5A
0.96	DGCR14	Transcript Identified by AceView. Entrez Gene ID(s) 8220
0.96	ANKRD20A4	ankyrin repeat domain 20 family. member A4
0.96	KRT13	keratin 13. type I
0.96	NNAT	neuronatin
0.96	R3HCC1L	R3H domain and coiled-coil containing 1-like

0.96	CDC42	Transcript Identified by AceView. Entrez Gene ID(s) 998
0.96	NAP1L4	nucleosome assembly protein 1-like 4
0.96	FA2H	fatty acid 2-hydroxylase
0.96	GNA15	guanine nucleotide binding protein (G protein). alpha 15 (Gq class)
0.96	PYY	peptide YY
0.96	NEK4	NIMA-related kinase 4
0.96	EML5	echinoderm microtubule associated protein like 5
0.96	TMEFF2	transmembrane protein with EGF-like and two follistatin-like domains 2
0.96	RPS3A	Zhang2013 ALT_DONOR. coding. INTERNAL. intronic best transcript NM_001006
0.96	FAH	fumarylacetoacetate hydrolase (fumarylacetoacetase)
0.96	API5	apoptosis inhibitor 5
0.96	ASNA1	arsA arsenite transporter. ATP-binding. homolog 1 (bacterial)
0.96	RASSF1	Ras association (RalGDS/AF-6) domain family member 1
0.96	UBR4	ubiquitin protein ligase E3 component n-recognin 4
0.96	GTSF1	gametocyte specific factor 1
0.96	CCDC84	coiled-coil domain containing 84
0.96	ZNF546	zinc finger protein 546
0.96	PRDM5	PR domain containing 5
0.96	C19orf67	chromosome 19 open reading frame 67
0.96	PGK2	phosphoglycerate kinase 2
0.96	KCNK15	potassium channel. two pore domain subfamily K. member 15
0.96	STRC	stereocilin
0.96	ZNF195	zinc finger protein 195
0.96	ST8SIA1	ST8 alpha-N-acetyl-neuraminide alpha-2.8-sialyltransferase 1
0.96	LRRC8D	leucine rich repeat containing 8 family. member D

0.96	UBLCP1	ubiquitin-like domain containing CTD phosphatase 1
0.96	TAL1	T-cell acute lymphocytic leukemia 1
0.96	SLC2A7	solute carrier family 2 (facilitated glucose transporter). member 7
0.96	PRRG3	proline rich Gla (G-carboxyglutamic acid) 3 (transmembrane)
0.96	MLXIP	MLX interacting protein
0.96	MAN2C1	mannosidase. alpha. class 2C. member 1
0.96	FFAR2	free fatty acid receptor 2
0.96	IL3RA	Homo sapiens interleukin 3 receptor. alpha (low affinity) (IL3RA). transcript variant 2. mRNA.; Homo sapiens interleukin 3 receptor. alpha (low affinity) (IL3RA). transcript variant 1. mRNA.; interleukin 3 receptor. alpha (low affinity) [Source:HGNC Symbol;Acc:HGNC:6012]; Homo sapiens interleukin 3 receptor. alpha (low affinity). mRNA (cDNA clone MGC:34174 IMAGE:5167281). complete cds.; Salzman2013 ANNOTATED. CDS. coding. INTERNAL. OVCODE. OVERLAPTX. OVEXON best transcript NM_002183
0.96	LTBP1	latent transforming growth factor beta binding protein 1
0.96	TMEM222	transmembrane protein 222
0.96	VWA5B1	von Willebrand factor A domain containing 5B1
0.96	CSF2	colony stimulating factor 2 (granulocyte-macrophage)
0.96	GNB3	guanine nucleotide binding protein (G protein). beta polypeptide 3
0.96	CXorf21	chromosome X open reading frame 21
0.96	DKK1	Jeck2013 ANTISENSE. CDS. coding. INTERNAL. intronic. OVCODE. OVEXON best transcript NM_012242
0.96	AGRP	agouti related neuropeptide
0.96	JAG2	jagged 2
0.96	ZNF835	Transcript Identified by AceView. Entrez Gene ID(s) 90485
0.96	VDAC1	voltage-dependent anion channel 1
0.96	RASEF	RAS and EF-hand domain containing
0.96	UGT2B28	UDP glucuronosyltransferase 2 family. polypeptide B28

0.96	TMEM63C	transmembrane protein 63C
0.96	MORN3	MORN repeat containing 3
0.96	CBX8	chromobox homolog 8
0.96	ADNP2	ADNP homeobox 2
0.96	ZNF804B	zinc finger protein 804B
0.96	GK5	glycerol kinase 5 (putative)
0.96	ANKZF1	ankyrin repeat and zinc finger domain containing 1
0.96	XPO1	exportin 1
0.96	OR6C4	olfactory receptor. family 6. subfamily C. member 4
0.96	OR7G3	olfactory receptor. family 7. subfamily G. member 3
0.96	ALOX15	arachidonate 15-lipoxygenase
0.96	PTRH1	peptidyl-tRNA hydrolase 1 homolog
0.96	LILRA5	leukocyte immunoglobulin-like receptor. subfamily A (with TM domain). member 5
0.96	NANP	N-acetylneuraminic acid phosphatase
0.96	TMED2	transmembrane p24 trafficking protein 2
0.96	DGKD	diacylglycerol kinase. delta 130kDa
0.96	MRPL14	mitochondrial ribosomal protein L14
0.96	GLIS1	GLIS family zinc finger 1
0.96	GPR33	G protein-coupled receptor 33 (gene/pseudogene)
0.96	FABP6	fatty acid binding protein 6. ileal
0.96	TREH	trehalase
0.96	LUZP4	leucine zipper protein 4
0.96	THEG5; AC011525.4; pleysey	testis highly expressed protein 5; novel transcript; Transcript Identified by AceView
0.96	C9orf114	chromosome 9 open reading frame 114
0.96	GABRR3	gamma-aminobutyric acid (GABA) A receptor. rho 3 (gene/pseudogene)

0.96	APOBEC3B	apolipoprotein B mRNA editing enzyme. catalytic polypeptide-like 3B
0.96	ADCY7	adenylate cyclase 7
0.96	CAPG	capping protein (actin filament). gelsolin-like
0.96	DEFB110	defensin. beta 110
0.96	BLOC1S4	biogenesis of lysosomal organelles complex-1. subunit 4. cappuccino
0.96	MYO1A	myosin IA
0.96	SCNN1D	sodium channel. non voltage gated 1 delta subunit
0.96	ZNF705A; FAM66C	zinc finger protein 705A; family with sequence similarity 66. member C
0.96	NIPAL1	NIPA-like domain containing 1
0.96	ZEB2	zinc finger E-box binding homeobox 2
0.96	CORO6	coronin 6
0.96	FAM120B	family with sequence similarity 120B
0.96	ULK4	unc-51 like kinase 4
0.96	CLCC1	chloride channel CLIC-like 1
0.96	TBC1D8B	TBC1 domain family. member 8B (with GRAM domain)
0.96	GSTA4	glutathione S-transferase alpha 4
0.96	CPSF1; MIR939; MIR1234; MIR6849	cleavage and polyadenylation specific factor 1; microRNA 939; microRNA 1234; microRNA 6849
0.96	OR6J1	olfactory receptor. family 6. subfamily J. member 1 (gene/pseudogene)
0.96	KRT2	keratin 2. type II
0.96	PGR	progesterone receptor
0.96	USP27X	ubiquitin specific peptidase 27. X-linked
0.96	ARHGAP32	Rho GTPase activating protein 32
0.96	TBC1D8	TBC1 domain family. member 8 (with GRAM domain)
0.96	CH25H	cholesterol 25-hydroxylase
0.96	PPM1D	protein phosphatase. Mg2+/Mn2+ dependent. 1D

0.96	SPATA2L	spermatogenesis associated 2-like
0.96	BPIFA3	BPI fold containing family A. member 3
0.96	UGT2B15	UDP glucuronosyltransferase 2 family. polypeptide B15
0.96	NOP2	NOP2 nucleolar protein
0.96	OR5H6	olfactory receptor. family 5. subfamily H. member 6 (gene/pseudogene)
0.96	PFKFB3	6-phosphofructo-2-kinase/fructose-2.6-biphosphatase 3
0.96	CENPL	centromere protein L
0.96	CTAGE9	CTAGE family. member 9
0.96	CLDN22	claudin 22
0.96	TRIP12	Transcript Identified by AceView. Entrez Gene ID(s) 9320
0.96	CLN6	ceroid-lipofuscinosis. neuronal 6. late infantile. variant
0.96	MDN1	midasin AAA ATPase 1
0.96	KIR2DL4	killer cell immunoglobulin-like receptor. two domains. long cytoplasmic tail. 4
0.96	GET4	golgi to ER traffic protein 4
0.96	PPP2CA	protein phosphatase 2. catalytic subunit. alpha isozyme
0.96	USP17L4	ubiquitin specific peptidase 17-like family member 4
0.96	IRS1	insulin receptor substrate 1
0.96	MACC1	metastasis associated in colon cancer 1
0.96	LRRC74A	leucine rich repeat containing 74A
0.96	WFDC6	WAP four-disulfide core domain 6
0.96	SSX7	synovial sarcoma. X breakpoint 7
0.96	AGPAT2	1-acylglycerol-3-phosphate O-acyltransferase 2
0.96	PRAMEF25	PRAME family member 25
0.96	HYOU1	hypoxia up-regulated 1
0.96	ECE2	endothelin converting enzyme 2

0.96	ASCL4	achaete-scute family bHLH transcription factor 4
0.96	BCL2L14	BCL2-like 14 (apoptosis facilitator)
0.96	DEFB126	defensin. beta 126
0.96	SIAE	sialic acid acetylesterase
0.96	MRPL45	mitochondrial ribosomal protein L45
0.96	OR56A4	olfactory receptor. family 56. subfamily A. member 4
0.96	TEX12	testis expressed 12
0.96	OR2AK2	olfactory receptor. family 2. subfamily AK. member 2
0.96	PDE3B	phosphodiesterase 3B. cGMP-inhibited
0.96	SFTPC	surfactant protein C
0.96	SMKR1	small lysine-rich protein 1
0.96	RTN2	reticulon 2
0.96	OR51F1	olfactory receptor. family 51. subfamily F. member 1 (gene/pseudogene)
0.96	NHLH2	nescient helix-loop-helix 2
0.96	AGT	angiotensinogen (serpin peptidase inhibitor. clade A. member 8)
0.96	CCBL2; RBMXL1	cysteine conjugate-beta lyase 2; RNA binding motif protein. X-linked-like 1
0.96	PELI2	pellino E3 ubiquitin protein ligase family member 2
0.96	COQ6	coenzyme Q6 monooxygenase
0.96	ORAOV1	oral cancer overexpressed 1
0.96	LYRM1	LYR motif containing 1
0.96	CD53	Memczak2013 ALT_ACCEPTOR. ALT_DONOR. coding. INTERNAL. intronic best transcript NM_001040033
0.96	OR6C1	olfactory receptor. family 6. subfamily C. member 1
0.96	RYR1	ryanodine receptor 1 (skeletal)
0.96	UBE2L6	Transcript Identified by AceView. Entrez Gene ID(s) 9246
0.96	MXD4	Memczak2013 ALT_ACCEPTOR. ALT_DONOR. coding. INTERNAL. intronic best transcript NM_006454

0.96	APOL6	apolipoprotein L. 6
0.96	SMIM23	small integral membrane protein 23
0.96	MCTP2	multiple C2 domains. transmembrane 2
0.96	TP53TG3B; TP53TG3; TP53TG3C	TP53 target 3B; TP53 target 3; TP53 target 3C
0.96	LOC399886; RP11-430H10.2; FLJ41423	uncharacterized LOC399886; Transcript Identified by AceView. Entrez Gene ID(s) 399886; novel transcript
0.96	CHTF8	chromosome transmission fidelity factor 8
0.96	SNTB2	syntrophin. beta 2 (dystrophin-associated protein A1. 59kDa. basic component 2)
0.96	PSAP	prosaposin
0.96	NAP1L6	nucleosome assembly protein 1-like 6
0.96	NDST2	N-deacetylase/N-sulfotransferase (heparan glucosaminyl) 2
0.96	RREB1	ras responsive element binding protein 1
0.96	STKLD1	serine/threonine kinase-like domain containing 1
0.96	S1PR4	sphingosine-1-phosphate receptor 4
0.96	SPATA31D3	SPATA31 subfamily D. member 3
0.96	PFDN1	prefoldin subunit 1
0.96	HOXA5	homeobox A5
0.96	CCKAR	cholecystokinin A receptor
0.96	ARL6IP5	ADP-ribosylation factor like GTPase 6 interacting protein 5
0.95	DHPS	deoxyhypusine synthase
0.95	LOC728485; CTD-2162K18.4	uncharacterized LOC728485; novel transcript
0.95	RNF168	ring finger protein 168. E3 ubiquitin protein ligase
0.95	PCDH1	protocadherin 1
0.95	ZNF337	zinc finger protein 337
0.95	AP3S1	adaptor-related protein complex 3. sigma 1 subunit
0.95	TRIM28	tripartite motif containing 28

0.95	TMEM30C	transmembrane protein 30C
0.95	PPIAL4A	peptidylprolyl isomerase A (cyclophilin A)-like 4A
0.95	APOBEC4	apolipoprotein B mRNA editing enzyme. catalytic polypeptide-like 4 (putative)
0.95	FAM173A	family with sequence similarity 173. member A
0.95	RP11-9L18.3; FAM78B	Transcript Identified by AceView. Entrez Gene ID(s) 149297; novel transcript
0.95	BROX	BRO1 domain and CAAX motif containing
0.95	BHLHA15	basic helix-loop-helix family. member a15
0.95	TAS2R31	taste receptor. type 2. member 31
0.95	ST13	suppression of tumorigenicity 13 (colon carcinoma) (Hsp70 interacting protein)
0.95	ZBTB8B	zinc finger and BTB domain containing 8B
0.95	FCGR3A	Fc fragment of IgG. low affinity IIIa. receptor (CD16a)
0.95	PLPP7	phospholipid phosphatase 7 (inactive)
0.95	C6orf141	chromosome 6 open reading frame 141
0.95	SRSF3	serine/arginine-rich splicing factor 3
0.95	DAPK3; MIR637	death-associated protein kinase 3; microRNA 637
0.95	NAT1	N-acetyltransferase 1 (arylamine N-acetyltransferase)
0.95	PRKAR1A; ARSG	protein kinase. cAMP-dependent. regulatory. type I. alpha; arylsulfatase G
0.95	ARHGEF5	Rho guanine nucleotide exchange factor 5
0.95	PEX26	peroxisomal biogenesis factor 26
0.95	OR2T3	olfactory receptor. family 2. subfamily T. member 3
0.95	PRAMEF11	PRAME family member 11
0.95	CD1D	CD1d molecule
0.95	GTF2F1	general transcription factor IIF subunit 1
0.95	ANK3	ankyrin 3. node of Ranvier (ankyrin G)
0.95	CRLF3	Memczak2013 ALT_ACCEPTOR. ALT_DONOR. coding. INTERNAL. intronic

		best transcript NM_015986
0.95	PKP4	plakophilin 4
0.95	MRGPRG-AS1	MRGPRG antisense RNA 1
0.95	C17orf50	chromosome 17 open reading frame 50
0.95	TENM3	teneurin transmembrane protein 3
0.95	PRKCB	protein kinase C. beta
0.95	POLM; MIR6838	polymerase (DNA directed). mu; microRNA 6838
0.95	BCL2L14	BCL2-like 14 (apoptosis facilitator)
0.95	WDR4	WD repeat domain 4
0.95	NR2E3	nuclear receptor subfamily 2. group E. member 3
0.95	ZC3H12B	zinc finger CCCH-type containing 12B
0.95	MZB1	marginal zone B and B1 cell-specific protein
0.95	SNCB	synuclein beta
0.95	ZNF442	zinc finger protein 442
0.95	NUDT11	nudix hydrolase 11
0.95	TDG	thymine DNA glycosylase
0.95	RETN	resistin
0.95	PHACTR2	Jeck2013 ALT_ACCEPTOR. ALT_DONOR. coding. INTERNAL. intronic best transcript NM_001100164
0.95	RTN4RL1	reticulon 4 receptor-like 1
0.95	ENKUR	enkurin. TRPC channel interacting protein
0.95	SNAP23	synaptosome associated protein 23kDa
0.95	THUMPD2	THUMP domain containing 2
0.95	PGAM4	phosphoglycerate mutase family member 4
0.95	TDRD12	tudor domain containing 12
0.95	PMVK	phosphomevalonate kinase

0.95	HCLS1	hematopoietic cell-specific Lyn substrate 1
0.95	C9orf16	chromosome 9 open reading frame 16
0.95	ATXN2	ataxin 2
0.95	YTHDF2	YTH N(6)-methyladenosine RNA binding protein 2
0.95	TPRXL	tetra-peptide repeat homeobox-like
0.95	STX5	syntaxin 5
0.95	RNF40	ring finger protein 40. E3 ubiquitin protein ligase
0.95	PPM1N	protein phosphatase. Mg2+/Mn2+ dependent. 1N (putative)
0.95	TXNIP	thioredoxin interacting protein
0.95	GOLGA8B	golgin A8 family. member B
0.95	FAM50B	family with sequence similarity 50. member B
0.95	FNTB	farnesyltransferase. CAAX box. beta
0.95	CLDN5	claudin 5
0.95	DNM2	dynamin 2
0.95	LAPTM4A	Memczak2013 ALT_ACCEPTOR. ALT_DONOR. coding. INTERNAL. intronic best transcript NM_014713
0.95	GRIK1	glutamate receptor. ionotropic. kainate 1
0.95	FUNDC1	FUN14 domain containing 1
0.95	PRAMEF9	
0.95	WDR45; PRAF2	WD repeat domain 45; PRA1 domain family. member 2
0.95	TRIM65	tripartite motif containing 65
0.95	MUC15	mucin 15. cell surface associated
0.95	REM1	RAS (RAD and GEM)-like GTP-binding 1
0.95	MADCAM1	mucosal vascular addressin cell adhesion molecule 1
0.95	ATG4C	autophagy related 4C. cysteine peptidase
0.95	SAMD15	sterile alpha motif domain containing 15

0.95	GLYR1	glyoxylate reductase 1 homolog (Arabidopsis)
0.95	DPH7	diphthamide biosynthesis 7
0.95	TXN2	thioredoxin 2
0.95	CTF1	cardiotrophin 1
0.95	MED20	mediator complex subunit 20
0.95	CSF2RA	colony stimulating factor 2 receptor. alpha. low-affinity (granulocyte-macrophage)
0.95	CAMP	cathelicidin antimicrobial peptide
0.95	KRTAP4-5	keratin associated protein 4-5
0.95	C6orf1	chromosome 6 open reading frame 1
0.95	ITGA4	integrin alpha 4
0.95	DPYSL4	dihydropyrimidinase-like 4
0.95	RASGEF1C	RasGEF domain family member 1C
0.95	RPS20; SNORD54	ribosomal protein S20; small nucleolar RNA. C/D box 54
0.95	SLC24A4	solute carrier family 24 (sodium/potassium/calcium exchanger). member 4
0.95	TET1	tet methylcytosine dioxygenase 1
0.95	SGK2	serum/glucocorticoid regulated kinase 2
0.95	AMH; MIR4321	anti-Mullerian hormone; microRNA 4321
0.95	NLRP3	NLR family. pyrin domain containing 3
0.95	ZCCHC2	zinc finger. CCHC domain containing 2
0.95	TTC30B	tetratricopeptide repeat domain 30B
0.95	BOC	BOC cell adhesion associated. oncogene regulated
0.95	OR2D2	olfactory receptor. family 2. subfamily D. member 2
0.95	DUSP26	dual specificity phosphatase 26 (putative)
0.95	DLGAP1	Memczak2013 ANTISENSE. coding. INTERNAL. intronic best transcript NM_001242765
0.95	CCDC65	coiled-coil domain containing 65

0.95	NRSN2	neurensin 2
0.95	LONRF2	LON peptidase N-terminal domain and ring finger 2
0.95	FSIP1	fibrous sheath interacting protein 1
0.95	DHH	desert hedgehog
0.95	GHDC	GH3 domain containing
0.95	LINC01387	long intergenic non-protein coding RNA 1387
0.95	C6orf47	chromosome 6 open reading frame 47
0.95	TCEAL2	transcription elongation factor A (SII)-like 2
0.95	APC	adenomatous polyposis coli
0.95	TUBA3E	tubulin. alpha 3e
0.95	B3GNT8	UDP-GlcNAc:betaGal beta-1.3-N-acetylglucosaminyltransferase 8
0.95	HSPA2	heat shock 70kDa protein 2
0.95	PPIG	peptidylprolyl isomerase G (cyclophilin G)
0.95	PKD1L3	polycystic kidney disease 1-like 3
0.95	BACH2	Memczak2013 ANTISENSE. coding. INTERNAL. intronic best transcript NM_001170794
0.95	CRNDE	colorectal neoplasia differentially expressed (non-protein coding)
0.95	AC009133.12; PAGR1	novel transcript. antisense to C16orf53 and MVP; PAXIP1 associated glutamate-rich protein 1 [Source:HGNC Symbol;Acc:HGNC:28707]
0.95	GRHPR	glyoxylate reductase/hydroxypyruvate reductase
0.95	TIGD6	tigger transposable element derived 6
0.95	PPM1G	protein phosphatase. Mg2+/Mn2+ dependent. 1G
0.95	CSTF2T	cleavage stimulation factor. 3 pre-RNA. subunit 2. tau variant
0.95	CDAN1	codanin 1
0.95	PSMB3	proteasome subunit beta 3
0.95	AKAP4	A kinase (PRKA) anchor protein 4
0.95	PIGF	phosphatidylinositol glycan anchor biosynthesis class F

0.95	C1orf189	chromosome 1 open reading frame 189
0.95	GPC3	glypican 3
0.95	EXOSC4; MIR6847	exosome component 4; microRNA 6847
0.95	PI4KAP1	phosphatidylinositol 4-kinase. catalytic. alpha pseudogene 1
0.95	MAFB	v-maf avian musculoaponeurotic fibrosarcoma oncogene homolog B
0.95	TMEM203	transmembrane protein 203
0.95	CCL28	chemokine (C-C motif) ligand 28
0.95	SLC31A2	solute carrier family 31 (copper transporter). member 2
0.95	ANKUB1	ankyrin repeat and ubiquitin domain containing 1
0.95	ZNF273	zinc finger protein 273
0.95	IMPAD1	inositol monophosphatase domain containing 1
0.95	ABHD12	abhydrolase domain containing 12
0.95	HHAT	hedgehog acyltransferase
0.95	SLC10A4	solute carrier family 10. member 4
0.95	BFSP2	beaded filament structural protein 2. phakinin
0.95	SPTLC1	serine palmitoyltransferase. long chain base subunit 1
0.95	ALPPL2	alkaline phosphatase. placental like 2
0.95	SORCS3	sortilin-related VPS10 domain containing receptor 3
0.95	NAA50	N(alpha)-acetyltransferase 50. NatE catalytic subunit
0.95	TMEM163	transmembrane protein 163
0.95	BCCIP	BRCA2 and CDKN1A interacting protein
0.95	RP11-499E18.1; SLC39A8	novel transcript; Transcript Identified by AceView. Entrez Gene ID(s) 64116
0.95	SSX2; SSX2B	synovial sarcoma. X breakpoint 2; synovial sarcoma. X breakpoint 2B
0.95	ARHGAP1	Rho GTPase activating protein 1
0.95	DPY19L2	dpy-19-like 2 (C. elegans)
0.95	PRR29	proline rich 29

0.95	PLN	phospholamban
0.95	AIPL1	aryl hydrocarbon receptor interacting protein-like 1
0.95	GLB1L3	galactosidase beta 1 like 3
0.95	RIBC2	RIB43A domain with coiled-coils 2
0.95	FMN1	Jeck2013 ANTISENSE. coding. INTERNAL. intronic best transcript NM_001103184
0.95	SULT1A4; SULT1A3	sulfotransferase family 1A member 4; sulfotransferase family 1A member 3
0.95	RNF125	ring finger protein 125. E3 ubiquitin protein ligase
0.95	LUC7L3	LUC7-like 3 pre-mRNA splicing factor
0.95	AEBP1; MIR4649	AE binding protein 1; microRNA 4649
0.95	EEF1D	eukaryotic translation elongation factor 1 delta (guanine nucleotide exchange protein)
0.95	NCR3	natural cytotoxicity triggering receptor 3
0.95	TCEAL3	transcription elongation factor A (SII)-like 3
0.95	PRDM13	PR domain containing 13
0.95	SERPINA13P	serpin peptidase inhibitor. clade A (alpha-1 antiproteinase. antitrypsin). member 13. pseudogene
0.95	NUP153	nucleoporin 153kDa
0.95	SP140L	SP140 nuclear body protein-like
0.95	ERBB2	erb-b2 receptor tyrosine kinase 2
0.95	FBXO6	F-box protein 6
0.95	CRYAA	crystallin alpha A
0.95	EMC4	ER membrane protein complex subunit 4
0.95	NOTUM	notum pectinacetylesterase homolog (Drosophila)
0.95	GIMAP4	GTPase. IMAP family member 4
0.95	MATN1	matrilin 1. cartilage matrix protein
0.95	SLTM	SAFB-like. transcription modulator

0.95	LDB1	LIM domain binding 1
0.95	MTMR8	myotubularin related protein 8
0.95	LECT2	leukocyte cell-derived chemotaxin 2
0.95	RTP3	receptor (chemosensory) transporter protein 3
0.95	CCDC63	coiled-coil domain containing 63
0.95	FUCA2	fucosidase. alpha-L- 2. plasma
0.95	ANKRD36C	ankyrin repeat domain 36C
0.95	FUT7	fucosyltransferase 7 (alpha (1.3) fucosyltransferase)
0.95	CFC1	cripto. FRL-1. cryptic family 1
0.95	EFHC1	EF-hand domain (C-terminal) containing 1
0.95	HNRNPR	heterogeneous nuclear ribonucleoprotein R
0.95	GCSH	glycine cleavage system protein H (aminomethyl carrier)
0.95	GID8	GID complex subunit 8
0.95	ANXA2R	annexin A2 receptor
0.95	SPINT4	serine peptidase inhibitor. Kunitz type 4
0.95	AP2A1	adaptor-related protein complex 2. alpha 1 subunit
0.95	LINC00092	long intergenic non-protein coding RNA 92
0.95	PLXDC1	plexin domain containing 1
0.95	CA11	carbonic anhydrase XI
0.95	FMN1	formin 1
0.95	RPS6KB2	ribosomal protein S6 kinase. 70kDa. polypeptide 2
0.95	OR51V1	olfactory receptor. family 51. subfamily V. member 1
0.95	HOXB5	homeobox B5
0.95	POU3F1	POU class 3 homeobox 1
0.95	MAPK8IP3	mitogen-activated protein kinase 8 interacting protein 3
0.95	NTM	neurotrimin

0.95	LHX6	LIM homeobox 6
0.95	ISOC1	isochorismatase domain containing 1
0.95	UNC45B	unc-45 myosin chaperone B
0.95	PRG1	p53-responsive gene 1
0.95	OR4C12	olfactory receptor. family 4. subfamily C. member 12
0.95	CALD1	caldesmon 1
0.95	NDUFA6	NADH dehydrogenase (ubiquinone) 1 alpha subcomplex. 6. 14kDa
0.95	ZC3HAV1	zinc finger CCCH-type. antiviral 1
0.95	PEX10	peroxisomal biogenesis factor 10
0.95	WBSCR16	Williams-Beuren syndrome chromosome region 16
0.95	TP53TG5	TP53 target 5
0.95	CACNG6	calcium channel. voltage-dependent. gamma subunit 6
0.95	ARG1	arginase 1
0.95	EFCAB14	EF-hand calcium binding domain 14
0.95	CHRNA4	cholinergic receptor. nicotinic alpha 4
0.95	ATG7	autophagy related 7
0.95	TIMM50	translocase of inner mitochondrial membrane 50 homolog (S. cerevisiae)
0.95	LOC101928093; RP11-779O18.1	uncharacterized LOC101928093; novel transcript
0.95	MON1A	MON1 secretory trafficking family member A
0.95	ITIH4	inter-alpha-trypsin inhibitor heavy chain family. member 4
0.95	SCAF11	SR-related CTD-associated factor 11
0.95	GPR153	G protein-coupled receptor 153
0.95	PC	pyruvate carboxylase
0.95	SELL	selectin L
0.95	LACTBL1	lactamase. beta-like 1
0.95	RPS25	ribosomal protein S25

0.95	CBWD6; CBWD5; CBWD7	COBW domain containing 6; COBW domain containing 5; COBW domain containing 7
0.95	NRAP	nebulin-related anchoring protein
0.95	CCDC8	coiled-coil domain containing 8
0.95	CD99L2	CD99 molecule-like 2
0.95	LOC400553; RP11-863P13.4	uncharacterized LOC400553; novel transcript
0.95	SYT1	synaptotagmin I
0.95	DNTT	Transcript Identified by AceView. Entrez Gene ID(s) 1791
0.95	TMX4	thioredoxin-related transmembrane protein 4
0.95	PIK3C2B	Transcript Identified by AceView. Entrez Gene ID(s) 5287
0.95	C17orf96	chromosome 17 open reading frame 96
0.95	OVOL2	ovo-like zinc finger 2
0.95	ZRANB2	zinc finger. RAN-binding domain containing 2
0.95	AKAP9	A kinase (PRKA) anchor protein 9
0.95	UBR3	Transcript Identified by AceView. Entrez Gene ID(s) 130507
0.95	POLD2	polymerase (DNA directed). delta 2. accessory subunit
0.95	POLE2	polymerase (DNA directed). epsilon 2. accessory subunit
0.95	COL4A5	collagen. type IV. alpha 5
0.95	AHSP	alpha hemoglobin stabilizing protein
0.95	MMP23B; MMP23A	matrix metallopeptidase 23B; matrix metallopeptidase 23A (pseudogene)
0.95	DEFB104A; DEFB104B	defensin. beta 104A; defensin. beta 104B
0.95	TBC1D7	TBC1 domain family. member 7
0.95	SATB1	SATB homeobox 1
0.95	HFM1	HFM1. ATP-dependent DNA helicase homolog
0.95	RPL36AL	ribosomal protein L36a-like
0.95	NANOS1	nanos homolog 1 (Drosophila)

0.95	C2	complement component 2
0.95	TMEM221	transmembrane protein 221
0.95	ANTXRL	anthrax toxin receptor-like
0.95	PCDHB7	protocadherin beta 7
0.95	ISM1	isthmin 1. angiogenesis inhibitor
0.95	ZNF583	zinc finger protein 583
0.95	CSRP3	cysteine and glycine-rich protein 3 (cardiac LIM protein)
0.95	PIF1	PIF1 5-to-3 DNA helicase
0.95	NOMO2	NODAL modulator 2
0.95	sept-01	septin 1
0.95	BIVM-ERCC5	BIVM-ERCC5 readthrough
0.95	VSTM2L	V-set and transmembrane domain containing 2 like
0.95	KRTAP27-1	keratin associated protein 27-1
0.95	APC2	adenomatosis polyposis coli 2
0.95	OR8I2	olfactory receptor. family 8. subfamily I. member 2
0.95	CNGA1	cyclic nucleotide gated channel alpha 1
0.95	PLA2G4C	phospholipase A2. group IVC (cytosolic. calcium-independent)
0.95	EMCN	endomucin
0.95	ZNF219	zinc finger protein 219
0.95	BHMG1	basic helix-loop-helix and HMG box domain containing 1
0.95	BIN3	bridging integrator 3
0.95	CARHSP1	Jeck2013 ANTISENSE. CDS. coding. INTERNAL. OVCODE. OVEXON best transcript NM_001042476
0.95	SOWAHD	sosondowah ankyrin repeat domain family member D
0.95	PDIA2	protein disulfide isomerase family A. member 2
0.95	LOC100129940; flanu; RP11-366L20.2	uncharacterized LOC100129940; Transcript Identified by AceView; novel transcript. antisense to HMGA2

0.95	ERAP1	Transcript Identified by AceView. Entrez Gene ID(s) 51752
0.95	DUOX1	dual oxidase 1
0.95	SCN5A	sodium channel. voltage gated. type V alpha subunit
0.95	CST1	cystatin SN
0.95	TMEM237	transmembrane protein 237
0.95	RPS26	ribosomal protein S26
0.95	CHI3L1	chitinase 3-like 1 (cartilage glycoprotein-39)
0.95	SYCE1L	synaptonemal complex central element protein 1-like
0.95	CD247	CD247 molecule
0.95	CXADR	coxsackie virus and adenovirus receptor
0.95	SLFNL1	schlafen like 1
0.95	HMCES	5-hydroxymethylcytosine (hmC) binding. ES cell-specific
0.95	PCNA	proliferating cell nuclear antigen
0.95	SHQ1	SHQ1. H/ACA ribonucleoprotein assembly factor
0.95	MUCL1	mucin-like 1
0.95	ENTPD6	ectonucleoside triphosphate diphosphohydrolase 6 (putative)
0.95	RBMS3	RNA binding motif. single stranded interacting protein 3
0.95	NXF2B; TCP11X2	nuclear RNA export factor 2B; t-complex 11 family. X-linked 2
0.95	SP110	SP110 nuclear body protein
0.95	CCDC116	coiled-coil domain containing 116
0.95	WDR97	WD repeat domain 97
0.95	MED25; MIR6800	mediator complex subunit 25; microRNA 6800
0.95	UGGT1	UDP-glucose glycoprotein glucosyltransferase 1
0.95	SRPR	signal recognition particle receptor (docking protein)
0.95	USP17L7	ubiquitin specific peptidase 17-like family member 7
0.95	GMPR	Zhang2013 ALT_ACCEPTOR. ALT_DONOR. coding. INTERNAL. intronic best

		transcript NM_006877
0.95	WDR92	WD repeat domain 92
0.95	BMP7	bone morphogenetic protein 7
0.95	F12	coagulation factor XII (Hageman factor)
0.95	FAM49A	family with sequence similarity 49. member A
0.95	ITM2C	integral membrane protein 2C
0.95	WBP2NL	WBP2 N-terminal like
0.95	CTAG1B; CTAG1A	cancer/testis antigen 1B; cancer/testis antigen 1A
0.95	STRBP; MIR600HG; MIR600	spermatid perinuclear RNA binding protein; MIR600 host gene; microRNA 600
0.95	OR8J2	olfactory receptor. family 8. subfamily J. member 2 (gene/pseudogene)
0.95	EVC2	Ellis van Creveld syndrome 2
0.95	PRRX2	paired related homeobox 2
0.95	FLCN	folliculin
0.95	GPRASP2	G protein-coupled receptor associated sorting protein 2
0.95	PGBD5	piggyBac transposable element derived 5
0.95	GABRA1	gamma-aminobutyric acid (GABA) A receptor. alpha 1
0.95	MAGEA8	MAGE family member A8
0.95	OR2T1	olfactory receptor. family 2. subfamily T. member 1
0.95	RASD2	RASD family. member 2
0.95	LCE5A	late cornified envelope 5A
0.95	VWA1	von Willebrand factor A domain containing 1
0.95	SGCE	sarcoglycan epsilon
0.95	MORF4L2	mortality factor 4 like 2
0.95	PGAM5	PGAM family member 5. serine/threonine protein phosphatase. mitochondrial
0.95	PIWIL1	piwi-like RNA-mediated gene silencing 1

0.95	ADAMDEC1	ADAM-like. decysin 1
0.95	ZNF705G	zinc finger protein 705G
0.95	LENG1	leukocyte receptor cluster (LRC) member 1
0.95	CAPN10	calpain 10
0.95	PITPNM1	phosphatidylinositol transfer protein. membrane-associated 1
0.95	VASH1	vasohibin 1
0.95	PI16	peptidase inhibitor 16
0.95	HYOU1	hypoxia up-regulated 1
0.95	NDUFS3	NADH dehydrogenase (ubiquinone) Fe-S protein 3. 30kDa (NADH-coenzyme Q reductase)
0.95	ABCB11	ATP binding cassette subfamily B member 11
0.95	FLOT2	Memczak2013 ANTISENSE. CDS. coding. INTERNAL. intronic best transcript NM_004475
0.95	PARS2	prolyl-tRNA synthetase 2. mitochondrial (putative)
0.95	PLXNA1	plexin A1
0.95	GOLGA8F; GOLGA8G	golgin A8 family. member F; golgin A8 family. member G
0.95	CSNK1G2-AS1	CSNK1G2 antisense RNA 1
0.95	COL13A1	collagen. type XIII. alpha 1
0.95	UMPS	uridine monophosphate synthetase
0.95	THBS4	thrombospondin 4
0.95	GPM6A	glycoprotein M6A
0.95	IFNA1	interferon. alpha 1
0.95	HORMAD2	HORMA domain containing 2
0.95	DCAF5	DDB1 and CUL4 associated factor 5
0.95	RSPRY1	ring finger and SPRY domain containing 1
0.95	PODXL	podocalyxin-like
0.95	PPP6C	protein phosphatase 6. catalytic subunit

0.95	TNS3	tensin 3
0.95	RASSF10	Ras association (RalGDS/AF-6) domain family (N-terminal) member 10
0.95	FBXO48	F-box protein 48
0.95	TXNDC9	thioredoxin domain containing 9
0.95	SS18	synovial sarcoma translocation. chromosome 18
0.95	FAM186A	family with sequence similarity 186. member A
0.95	G2E3	G2/M-phase specific E3 ubiquitin protein ligase
0.95	RABGAP1L	RAB GTPase activating protein 1-like
0.95	SUPT20HL2	SPT20 homolog. SAGA complex component-like 2
0.95	ATP5O	ATP synthase. H+ transporting. mitochondrial F1 complex. O subunit
0.95	SYT15; ABC7-42404400C24.1	Homo sapiens synaptotagmin XV (SYT15). transcript variant a. mRNA.; Homo sapiens synaptotagmin XV (SYT15). transcript variant b. mRNA.; Homo sapiens synaptotagmin XV. mRNA (cDNA clone MGC:164845 IMAGE:40147906). complete cds.; Synaptotagmin-15 [Source:UniProtKB/Swiss-Prot;Acc:Q9BQS2]
0.95	PRAMEF19	PRAME family member 19 [Source:EntrezGene;Acc:645414]
0.95	EML1	echinoderm microtubule associated protein like 1
0.95	LY6G5B; CSNK2B	lymphocyte antigen 6 complex. locus G5B; casein kinase 2. beta polypeptide
0.95	DAAM2	dishevelled associated activator of morphogenesis 2
0.95	EHBP1L1	EH domain binding protein 1 like 1
0.95	CCDC144NL-AS1	CCDC144NL antisense RNA 1
0.95	RIC3	RIC3 acetylcholine receptor chaperone
0.95	SMIM10L2B	small integral membrane protein 10 like 2B
0.95	FAM114A1	family with sequence similarity 114. member A1
0.95	CALHM2	calcium homeostasis modulator 2
0.95	GSDMA	gasdermin A
0.95	TOM1	target of myb1 membrane trafficking protein

0.95	DTX2	deltex 2. E3 ubiquitin ligase
0.95	PHOSPHO2	phosphatase. orphan 2
0.95	GALNT2	polypeptide N-acetylgalactosaminyltransferase 2
0.95	RNF185	Transcript Identified by AceView. Entrez Gene ID(s) 91445
0.95	RTN4RL2	reticulon 4 receptor-like 2
0.95	NOX4	NADPH oxidase 4
0.95	RUNDC3B	RUN domain containing 3B
0.95	SBSPON	somatomedin B and thrombospondin type 1 domain containing
0.95	FOXR2	forkhead box R2
0.95	KRTAP4-7	keratin associated protein 4-7
0.95	ITPKB	Transcript Identified by AceView. Entrez Gene ID(s) 3707
0.95	RGS6	regulator of G-protein signaling 6
0.95	HCRT	hypocretin (orexin) neuropeptide precursor
0.95	PSMC4	proteasome 26S subunit. ATPase 4 [Source:HGNC Symbol;Acc:HGNC:9551]
0.95	MFAP3	microfibrillar associated protein 3
0.95	SST	somatostatin
0.95	HMGB1	high mobility group box 1
0.95	CREG2	cellular repressor of E1A-stimulated genes 2
0.95	ARFGAP3	ADP-ribosylation factor GTPase activating protein 3
0.95	GPR37L1	G protein-coupled receptor 37 like 1
0.95	DIP2B	disco-interacting protein 2 homolog B
0.95	TMEM220	transmembrane protein 220
0.95	CLEC2B	C-type lectin domain family 2. member B
0.95	FOXS1	forkhead box S1
0.95	CDRT15	CMT1A duplicated region transcript 15
0.95	RPS25	ribosomal protein S25

0.95	RAD23B	RAD23 homolog B. nucleotide excision repair protein
0.95	UNK	unkempt family zinc finger
0.95	FZR1	Jeck2013 ANTISENSE. coding. INTERNAL. intronic best transcript NM_016263
0.95	RPAP2	RNA polymerase II associated protein 2
0.95	GTSE1	G-2 and S-phase expressed 1
0.95	NDST3	N-deacetylase/N-sulfotransferase (heparan glucosaminyl) 3
0.95	MROH2A	maestro heat-like repeat family member 2A
0.95	MMP12	matrix metallopeptidase 12
0.95	FBXW2	F-box and WD repeat domain containing 2
0.95	LHX8	LIM homeobox 8
0.95	BAAT	bile acid-CoA:amino acid N-acyltransferase
0.95	CDH18	cadherin 18. type 2
0.95	CDC42EP3	CDC42 effector protein (Rho GTPase binding) 3
0.95	ZNF35	zinc finger protein 35
0.95	OR2AE1	olfactory receptor. family 2. subfamily AE. member 1
0.95	PRPS1L1	phosphoribosyl pyrophosphate synthetase 1-like 1
0.95	ACTR2	ARP2 actin-related protein 2 homolog (yeast)
0.95	ARAP1	ArfGAP with RhoGAP domain. ankyrin repeat and PH domain 1
0.95	EXTL3	exostosin-like glycosyltransferase 3
0.95	TTC13	tetratricopeptide repeat domain 13
0.95	DBR1	debranching RNA lariats 1
0.95	INPP5B	inositol polyphosphate-5-phosphatase B
0.95	MUC7	mucin 7. secreted
0.95	CPXM1	carboxypeptidase X (M14 family). member 1
0.95	APLNR	apelin receptor
0.95	PALM3	paralemmin 3

0.95	CRIPAK	cysteine-rich PAK1 inhibitor
0.95	RNF180	ring finger protein 180
0.95	KRTAP5-9	keratin associated protein 5-9
0.95	TRIM27	Transcript Identified by AceView. Entrez Gene ID(s) 5987
0.95	GYS2	glycogen synthase 2 (liver)
0.95	FZR1	fizzy/cell division cycle 20 related 1
0.95	PLGRKT	plasminogen receptor. C-terminal lysine transmembrane protein
0.95	PCDHB1	protocadherin beta 1
0.95	CLPP	caseinolytic mitochondrial matrix peptidase proteolytic subunit
0.95	OR2T27	olfactory receptor. family 2. subfamily T. member 27
0.95	FBXO36	Transcript Identified by AceView. Entrez Gene ID(s) 130888
0.95	SNN	stannin
0.95	OGDHL	oxoglutarate dehydrogenase-like
0.95	SPDYE5	speedy/RINGO cell cycle regulator family member E5
0.95	HDAC9	Jeck2013 ALT_ACCEPTOR. ALT_DONOR. coding. INTERNAL. intronic best transcript NM_178423
0.95	CTU1	cytosolic thiouridylase subunit 1
0.95	CPNE3	copine III
0.95	KRBA2	KRAB-A domain containing 2
0.95	RNF13	ring finger protein 13
0.95	XYLB	xylulokinase homolog (H. influenzae)
0.95	SF3B4	splicing factor 3b. subunit 4. 49kDa
0.95	ZFPM1	zinc finger protein. FOG family member 1
0.95	PIGS	phosphatidylinositol glycan anchor biosynthesis class S
0.95	DLG2	discs. large homolog 2 (Drosophila)
0.95	PGAM1	phosphoglycerate mutase 1 (brain)

0.95	ZNF10	zinc finger protein 10
0.95	SLC4A8	solute carrier family 4. sodium bicarbonate cotransporter. member 8
0.95	RPS6KA1	Zhang2013 ALT_ACCEPTOR. ALT_DONOR. coding. INTERNAL. intronic best transcript NM_002953
0.95	PPP1CA	protein phosphatase 1. catalytic subunit. alpha isozyme
0.95	TRIM40	tripartite motif containing 40
0.95	LIN7A	lin-7 homolog A (C. elegans)
0.95	CAND2	cullin-associated and neddylation-dissociated 2 (putative)
0.95	ZNF516	zinc finger protein 516
0.95	ARPP21	Transcript Identified by AceView. Entrez Gene ID(s) 10777
0.95	PPIAL4F; PPIAL4D	peptidylprolyl isomerase A (cyclophilin A)-like 4F; peptidylprolyl isomerase A (cyclophilin A)-like 4D
0.95	ZNF704	zinc finger protein 704
0.95	UGT2B10	UDP glucuronosyltransferase 2 family. polypeptide B10
0.95	SSR4	signal sequence receptor. delta
0.95	OR8J3	olfactory receptor. family 8. subfamily J. member 3
0.95	GZMA	granzyme A
0.95	SLC50A1	solute carrier family 50 (sugar efflux transporter). member 1
0.95	TMEM216	transmembrane protein 216
0.95	OPN4	opsin 4
0.95	ADAM28	Memczak2013 ANTISENSE. coding. INTERNAL. intronic best transcript NM_014265
0.95	KLK4	kallikrein related peptidase 4
0.95	KRTAP3-2	keratin associated protein 3-2
0.95	PNOC	prepronociceptin
0.95	GYPC	Memczak2013 ANTISENSE. coding. INTERNAL. UTR3 best transcript NM_016815

0.95	TMEM169	Transcript Identified by AceView. Entrez Gene ID(s) 92691
0.95	RBFA; RBFADN	ribosome binding factor A (putative); RBFA downstream neighbor (non-protein coding)
0.95	ARCN1	archain 1
0.95	RUNX1	runt-related transcription factor 1
0.95	NELFA; MIR943	negative elongation factor complex member A; microRNA 943
0.95	NDUFB9	NADH dehydrogenase (ubiquinone) 1 beta subcomplex. 9. 22kDa
0.95	THOP1	thimet oligopeptidase 1
0.95	RNF38	ring finger protein 38
0.95	ACVRL1	Memczak2013 ANTISENSE. CDS. coding. INTERNAL. intronic best transcript NM_001077401
0.95	MTMR14	myotubularin related protein 14
0.95	PRR5	proline rich 5 (renal)
0.95	PRSS27	protease. serine 27
0.95	IL21	interleukin 21
0.95	GSTM2	glutathione S-transferase mu 2 (muscle)
0.95	AVPI1	arginine vasopressin-induced 1
0.95	TUBA4B	tubulin. alpha 4b
0.95	CLEC18A	C-type lectin domain family 18. member A
0.95	B3GALNT1	beta-1.3-N-acetylgalactosaminyltransferase 1 (globoside blood group)
0.95	SNX19	sorting nexin 19
0.95	FNDC7	fibronectin type III domain containing 7
0.95	C6orf222	chromosome 6 open reading frame 222
0.95	GPR21; RABGAP1	G protein-coupled receptor 21; RAB GTPase activating protein 1
0.95	C9orf135	chromosome 9 open reading frame 135
0.95	RFX4	regulatory factor X. 4 (influences HLA class II expression)
0.95	SLC35C2	solute carrier family 35 (GDP-fucose transporter). member C2

0.95	RGS6	regulator of G-protein signaling 6
0.95	PPP1R9B	protein phosphatase 1. regulatory subunit 9B
0.95	NLRP10	NLR family. pyrin domain containing 10
0.95	TMIE	transmembrane inner ear
0.95	KCNJ16	potassium channel. inwardly rectifying subfamily J. member 16
0.95	PSMC4	proteasome 26S subunit. ATPase 4 [Source:HGNC Symbol;Acc:HGNC:9551]
0.95	EBAG9	estrogen receptor binding site associated. antigen. 9
0.95	TANC2	tetratricopeptide repeat. ankyrin repeat and coiled-coil containing 2
0.95	MYH9	myosin. heavy chain 9. non-muscle
0.95	LZIC	leucine zipper and CTNNBIP1 domain containing
0.95	IKBIP	IKBKB interacting protein
0.95	PCED1B	PC-esterase domain containing 1B
0.95	LSM5	LSM5 homolog. U6 small nuclear RNA and mRNA degradation associated
0.95	PNMA2	paraneoplastic Ma antigen 2
0.95	GUCD1	guanylyl cyclase domain containing 1
0.95	R3HDM1	Transcript Identified by AceView. Entrez Gene ID(s) 23518
0.95	EDNRB	endothelin receptor type B
0.95	MAN1B1	mannosidase. alpha. class 1B. member 1
0.95	OR51B2	olfactory receptor. family 51. subfamily B. member 2 (gene/pseudogene)
0.95	MMP27	matrix metallopeptidase 27
0.95	CARS2	cysteinyl-tRNA synthetase 2. mitochondrial (putative)
0.95	RS1	retinoschisin 1
0.95	SLC19A2	solute carrier family 19 (thiamine transporter). member 2
0.95	PAPPA	pregnancy-associated plasma protein A. pappalysin 1
0.95	KIF9	kinesin family member 9
0.95	ARID1B	Memczak2013 ANTISENSE. CDS. coding. INTERNAL best transcript

		NM_017519
0.95	ZNF440	zinc finger protein 440
0.95	TIMM10	translocase of inner mitochondrial membrane 10 homolog (yeast)
0.95	PPP1R16A	protein phosphatase 1. regulatory subunit 16A
0.95	CD2	CD2 molecule
0.94	MPL	MPL proto-oncogene. thrombopoietin receptor
0.94	MANSC1	MANSC domain containing 1
0.94	ZNF235	zinc finger protein 235
0.94	LXN	latexin
0.94	LRRC73	leucine rich repeat containing 73
0.94	SSSCA1	Sjogren syndrome/scleroderma autoantigen 1
0.94	SIMC1	SUMO-interacting motifs containing 1
0.94	JAKMIP3	Janus kinase and microtubule interacting protein 3
0.94	PROX2	prospero homeobox 2
0.94	HMP19	HMP19 protein; Neuron-specific protein family member 2 [Source:UniProtKB/Swiss-Prot;Acc:Q9Y328]; Transcript Identified by AceView. Entrez Gene ID(s) 51617
0.94	STS	steroid sulfatase (microsomal). isozyme S
0.94	EPS8L1	EPS8-like 1
0.94	CARNMT1	carnosine N-methyltransferase 1
0.94	ARL16	ADP-ribosylation factor like GTPase 16
0.94	ISLR	immunoglobulin superfamily containing leucine-rich repeat
0.94	KRTAP8-1	keratin associated protein 8-1
0.94	UBXN6	UBX domain protein 6
0.94	TROAP	trophinin associated protein
0.94	ECI1	enoyl-CoA delta isomerase 1
0.94	INO80D	INO80 complex subunit D

0.94	CLEC4G	C-type lectin domain family 4. member G
0.94	TBC1D3L	TBC1 domain family. member 3L
0.94	C8orf76	chromosome 8 open reading frame 76
0.94	TMEM115	transmembrane protein 115
0.94	DKFZp779M0652; CTD-2210P24.4	uncharacterized DKFZp779M0652; Transcript Identified by AceView. Entrez Gene ID(s) 374387. RefSeq ID(s) NR_027134; novel transcript; uncharacterized DKFZp779M0652 [Source:EntrezGene;Acc:374387]
0.94	MPRIP	myosin phosphatase Rho interacting protein
0.94	C1GALT1	core 1 synthase. glycoprotein-N-acetylgalactosamine 3-beta-galactosyltransferase 1
0.94	MPEG1	macrophage expressed 1
0.94	GPR156	G protein-coupled receptor 156
0.94	PPP1R42	protein phosphatase 1. regulatory subunit 42
0.94	RP1-122P22.2; RIN2	novel transcript; Transcript Identified by AceView. Entrez Gene ID(s) 54453
0.94	C17orf102	chromosome 17 open reading frame 102
0.94	KRTAP5-1	keratin associated protein 5-1
0.94	PDYN	prodynorphin
0.94	C8A	complement component 8. alpha polypeptide
0.94	CDR2L	cerebellar degeneration-related protein 2-like
0.94	TUBB6	tubulin. beta 6 class V
0.94	MOV10L1	Mov10 RISC complex RNA helicase like 1
0.94	TOMM22	translocase of outer mitochondrial membrane 22 homolog (yeast)
0.94	KRTAP9-9	keratin associated protein 9-9
0.94	LFNG; MIR4648	LFNG O-fucosylpeptide 3-beta-N-acetylglucosaminyltransferase; microRNA 4648
0.94	ACAP3	ArfGAP with coiled-coil. ankyrin repeat and PH domains 3
0.94	SP8	Sp8 transcription factor

0.94	IFT20	intraflagellar transport 20
0.94	CRYZ	crystallin zeta
0.94	SS18L1	synovial sarcoma translocation gene on chromosome 18-like 1
0.94	HYAL1	hyaluronoglucosaminidase 1
0.94	DONSON; CRYZL1	downstream neighbor of SON; crystallin zeta like 1
0.94	IGLL1	immunoglobulin lambda-like polypeptide 1
0.94	PXT1	peroxisomal. testis specific 1
0.94	ALB	albumin
0.94	ZBTB11	zinc finger and BTB domain containing 11
0.94	PLIN2	perilipin 2
0.94	C19orf33	chromosome 19 open reading frame 33
0.94	C16orf62	chromosome 16 open reading frame 62
0.94	FAM25G; FAM25C; FAM25BP	family with sequence similarity 25. member G; family with sequence similarity 25. member C; protein FAM25
0.94	SUMF1	sulfatase modifying factor 1
0.94	GABRA3	gamma-aminobutyric acid (GABA) A receptor. alpha 3
0.94	CA5B	carbonic anhydrase VB. mitochondrial
0.94	ATRAID	all-trans retinoic acid-induced differentiation factor
0.94	GSDMD	gasdermin D
0.94	LIMK1	Memczak2013 ANTISENSE. CDS. coding. INTERNAL best transcript NM_001204426
0.94	NUP214	nucleoporin 214kDa
0.94	LIPE	lipase. hormone-sensitive
0.94	SF3A2	splicing factor 3a subunit 2
0.94	C15orf32	chromosome 15 open reading frame 32
0.94	ZBTB47	zinc finger and BTB domain containing 47
0.94	INPP5A	inositol polyphosphate-5-phosphatase A

0.94	THAP3	THAP domain containing. apoptosis associated protein 3
0.94	WASL	Wiskott-Aldrich syndrome-like
0.94	PNPLA8	patatin-like phospholipase domain containing 8
0.94	PLA2G4E	phospholipase A2. group IVE
0.94	THRB	thyroid hormone receptor. beta
0.94	KCNH1	potassium channel. voltage gated eag related subfamily H. member 1
0.94	PZP	pregnancy-zone protein
0.94	UBN2	ubinuclein 2
0.94	PPP4C	protein phosphatase 4. catalytic subunit
0.94	SDHC	succinate dehydrogenase complex. subunit C. integral membrane protein. 15kDa
0.94	CHSY3	chondroitin sulfate synthase 3
0.94	BLNK	Memczak2013 ALT_ACCEPTOR. ALT_DONOR. coding. INTERNAL. intronic best transcript NM_013314
0.94	ING3	Transcript Identified by AceView. Entrez Gene ID(s) 54556
0.94	SLC3A1	solute carrier family 3 (amino acid transporter heavy chain). member 1
0.94	ATXN3L	ataxin 3-like
0.94	PLA2G5	phospholipase A2. group V
0.94	KRT6C	keratin 6C. type II
0.94	YY1; MIR6764	YY1 transcription factor; microRNA 6764
0.94	EXOC3	exocyst complex component 3
0.94	SNAPC2	small nuclear RNA activating complex polypeptide 2
0.94	ELK4	ELK4. ETS-domain protein (SRF accessory protein 1)
0.94	CD164	CD164 molecule. sialomucin
0.94	ETNK1	ethanolamine kinase 1
0.94	EXD3	exonuclease 3-5 domain containing 3
0.94	N6AMT1	N-6 adenine-specific DNA methyltransferase 1 (putative)

0.94	ASMT; AKAP17A	acetylserotonin O-methyltransferase; A kinase (PRKA) anchor protein 17A
0.94	ASMT; SFRS17AandASMT	Homo sapiens acetylserotonin O-methyltransferase (ASMT). transcript variant 1. mRNA.; Homo sapiens acetylserotonin O-methyltransferase (ASMT). transcript variant 3. mRNA.; acetylserotonin O-methyltransferase [Source:HGNC Symbol;Acc:HGNC:750]; Homo sapiens acetylserotonin O-methyltransferase. mRNA (cDNA clone MGC:2145 IMAGE:3356470). complete cds.; Transcript Identified by AceView. Entrez Gene ID(s) 438; 8227. RefSeq ID(s) NM_001171038
0.94	GP2	glycoprotein 2 (zymogen granule membrane)
0.94	FAM177B	family with sequence similarity 177. member B
0.94	ZNF213	zinc finger protein 213
0.94	ACSS1	acyl-CoA synthetase short-chain family member 1
0.94	TWSG1	twisted gastrulation BMP signaling modulator 1
0.94	CELF2	Transcript Identified by AceView. Entrez Gene ID(s) 10659
0.94	C2orf27B	chromosome 2 open reading frame 27B
0.94	OVOL1	ovo-like zinc finger 1
0.94	DLG1	discs. large homolog 1 (Drosophila)
0.94	CST2	cystatin SA
0.94	DNMT3L	DNA (cytosine-5-)-methyltransferase 3-like
0.94	VPS11	VPS11. CORVET/HOPS core subunit [Source:HGNC Symbol;Acc:HGNC:14583]
0.94	LRRC66	leucine rich repeat containing 66
0.94	IL18BP	interleukin 18 binding protein
0.94	HOXC10	homeobox C10
0.94	RBM3	RNA binding motif (RNP1. RRM) protein 3
0.94	IQGAP3	IQ motif containing GTPase activating protein 3
0.94	WDR91	WD repeat domain 91
0.94	KRT19	keratin 19. type I
0.94	TMEM100	transmembrane protein 100

0.94	CACNG7	calcium channel. voltage-dependent. gamma subunit 7
0.94	DEFB106A	defensin. beta 106A
0.94	C1orf109	chromosome 1 open reading frame 109
0.94	C1QTNF1	C1q and tumor necrosis factor related protein 1
0.94	TMEM151B	transmembrane protein 151B
0.94	HECTD1	HECT domain containing E3 ubiquitin protein ligase 1
0.94	PDC	phosducin
0.94	AUH	AU RNA binding protein/enoyl-CoA hydratase
0.94	DEFB119	defensin. beta 119
0.94	TLE6	transducin-like enhancer of split 6
0.94	ARHGAP35	Rho GTPase activating protein 35
0.94	SPRYD4	SPRY domain containing 4
0.94	ACKR4	atypical chemokine receptor 4
0.94	MRPL36	mitochondrial ribosomal protein L36
0.94	NARF	nuclear prelamin A recognition factor
0.94	TBC1D22B	TBC1 domain family. member 22B
0.94	BSDC1	BSD domain containing 1
0.94	INPP5K	inositol polyphosphate-5-phosphatase K
0.94	TNFSF13B	Transcript Identified by AceView. Entrez Gene ID(s) 10673
0.94	RBM7	RNA binding motif protein 7
0.94	RP11-362J17.1; PACRGL	novel transcript. antisense to KCNIP4; Transcript Identified by AceView. Entrez Gene ID(s) 133015
0.94	SAXO1	stabilizer of axonemal microtubules 1
0.94	GRIN2C	glutamate receptor. ionotropic. N-methyl D-aspartate 2C
0.94	F13B	coagulation factor XIII. B polypeptide
0.94	TMEM110	transmembrane protein 110

0.94	ADCY3	adenylate cyclase 3
0.94	WDR48	WD repeat domain 48
0.94	DRP2	dystrophin related protein 2
0.94	KIAA0319	KIAA0319
0.94	POMZP3	POM121 and ZP3 fusion
0.94	RERE	arginine-glutamic acid dipeptide (RE) repeats
0.94	ACOT6	acyl-CoA thioesterase 6
0.94	FBL	fibrillarin
0.94	PAPD5	PAP associated domain containing 5
0.94	ASGR1	asialoglycoprotein receptor 1
0.94	RPE65	retinal pigment epithelium-specific protein 65kDa
0.94	LARP1	La ribonucleoprotein domain family. member 1
0.94	LNP1	leukemia NUP98 fusion partner 1
0.94	ZNF676	zinc finger protein 676
0.94	OR8G5	olfactory receptor. family 8. subfamily G. member 5
0.94	SCPEP1	serine carboxypeptidase 1
0.94	HSPB9	heat shock protein. alpha-crystallin-related. B9
0.94	CELSR1	cadherin. EGF LAG seven-pass G-type receptor 1
0.94	GLYATL3	glycine-N-acyltransferase-like 3
0.94	ASCC1	activating signal cointegrator 1 complex subunit 1
0.94	TCEAL7	transcription elongation factor A (SII)-like 7
0.94	TSPAN19	tetraspanin 19
0.94	SPRR2F	small proline-rich protein 2F
0.94	MYOCD	myocardin
0.94	SSC4D	scavenger receptor cysteine rich family. 4 domains
0.94	SIDT2	SID1 transmembrane family. member 2

0.94	SLC25A38	solute carrier family 25. member 38
0.94	UBE2S	Zhang2013 ALT_ACCEPTOR. ALT_DONOR. coding. INTERNAL. intronic. OVERLAPTX best transcript NM_014501
0.94	IRGM	immunity-related GTPase family. M
0.94	KCTD1	potassium channel tetramerization domain containing 1
0.94	LOC101929372; AC004019.13	uncharacterized LOC101929372; novel transcript
0.94	FLJ33360; CTD-2324F15.2	FLJ33360 protein; FLJ33360 protein [Source:EntrezGene;Acc:401172]; putative novel transcript
0.94	OAZ3	ornithine decarboxylase antizyme 3
0.94	IL31	interleukin 31
0.94	RNF220	ring finger protein 220
0.94	BCL9	B-cell CLL/lymphoma 9
0.94	GFI1B	growth factor independent 1B transcription repressor
0.94	ZNF484	zinc finger protein 484
0.94	CDH20	cadherin 20. type 2
0.94	ZFP1	ZFP1 zinc finger protein
0.94	SORBS1	Transcript Identified by AceView. Entrez Gene ID(s) 10580
0.94	RAD21L1	RAD21 cohesin complex component like 1
0.94	ZNF546	zinc finger protein 546
0.94	KBTBD3	kelch repeat and BTB (POZ) domain containing 3
0.94	ZAR1	zygote arrest 1
0.94	LY75-CD302; CD302; LY75	LY75-CD302 readthrough; CD302 molecule; lymphocyte antigen 75
0.94	CAD	carbamoyl-phosphate synthetase 2. aspartate transcarbamylase. and dihydroorotase
0.94	ITGA8	integrin alpha 8
0.94	EFHD1	EF-hand domain family member D1
0.94	LBX1	ladybird homeobox 1

0.94	LOR	loricrin
0.94	PKMYT1	Transcript Identified by AceView. Entrez Gene ID(s) 9088
0.94	SAG	S-antigen; retina and pineal gland (arrestin)
0.94	COPZ1	coatomer protein complex subunit zeta 1
0.94	CASC4	cancer susceptibility candidate 4
0.94	TPSG1	tryptase gamma 1
0.94	CORO2A	coronin. actin binding protein. 2A
0.94	FAM153A	Transcript Identified by AceView. Entrez Gene ID(s) 285596
0.94	SLC12A3	solute carrier family 12 (sodium/chloride transporter). member 3
0.94	OXT	oxytocin/neurophysin I prepropeptide
0.94	DVL3	dishevelled segment polarity protein 3
0.94	CNFN	cornifelin
0.94	VWF	von Willebrand factor
0.94	FAM217A	family with sequence similarity 217. member A
0.94	SCIN	scinderin
0.94	PANO1	proapoptotic nucleolar protein 1
0.94	VTI1A	vesicle transport through interaction with t-SNAREs 1A
0.94	DOCK2	Memczak2013 ANTISENSE. CDS. coding. INTERNAL best transcript NM_004946
0.94	LSM8	LSM8 homolog. U6 small nuclear RNA associated
0.94	FEZF2	FEZ family zinc finger 2
0.94	OR51B4	olfactory receptor. family 51. subfamily B. member 4
0.94	EPB41	erythrocyte membrane protein band 4.1
0.94	EXTL1	exostosin-like glycosyltransferase 1
0.94	EVI5L	ecotropic viral integration site 5-like
0.94	NDUFS7	NADH dehydrogenase (ubiquinone) Fe-S protein 7. 20kDa (NADH-coenzyme Q reductase)

0.94	UCMA	upper zone of growth plate and cartilage matrix associated
0.94	OFCC1	orofacial cleft 1 candidate 1
0.94	GNAI1	guanine nucleotide binding protein (G protein). alpha inhibiting activity polypeptide 1
0.94	NFRKB	Transcript Identified by AceView. Entrez Gene ID(s) 4798
0.94	FDCSP	follicular dendritic cell secreted protein
0.94	SLC6A20	solute carrier family 6 (proline IMINO transporter). member 20
0.94	NNMT	nicotinamide N-methyltransferase
0.94	ZNF703	zinc finger protein 703
0.94	FAM83H	family with sequence similarity 83. member H
0.94	TRIP12	thyroid hormone receptor interactor 12
0.94	OR10H4	olfactory receptor. family 10. subfamily H. member 4
0.94	TRMT1L	tRNA methyltransferase 1 like
0.94	CDH4	cadherin 4. type 1. R-cadherin (retinal)
0.94	PCM1	Transcript Identified by AceView. Entrez Gene ID(s) 5108
0.94	MYLK4	myosin light chain kinase family member 4
0.94	GLIS2	GLIS family zinc finger 2
0.94	GCNT2	glucosaminyl (N-acetyl) transferase 2. I-branching enzyme (I blood group)
0.94	TRIM49D2; TRIM49D1	tripartite motif containing 49D2; tripartite motif containing 49D1
0.94	HNRNPF	Memczak2013 ALT_ACCEPTOR. ALT_DONOR. coding. INTERNAL. intronic best transcript NM_001098207
0.94	NRBP1	nuclear receptor binding protein 1
0.94	LOC100505841	zinc finger protein 474-like
0.94	PDCD1LG2	programmed cell death 1 ligand 2
0.94	PON2	paraoxonase 2
0.94	RNGTT	RNA guanylyltransferase and 5-phosphatase
0.94	SSBP3	single stranded DNA binding protein 3

0.94	RPUSD3	RNA pseudouridylate synthase domain containing 3
0.94	AP4M1	adaptor-related protein complex 4. mu 1 subunit
0.94	OR52N2	olfactory receptor. family 52. subfamily N. member 2
0.94	OR2T4	olfactory receptor. family 2. subfamily T. member 4
0.94	ADM2	adrenomedullin 2
0.94	FAM184A	family with sequence similarity 184. member A
0.94	PYROXD2; MIR1287	pyridine nucleotide-disulphide oxidoreductase domain 2; microRNA 1287
0.94	CCDC153	coiled-coil domain containing 153
0.94	GNRH1	gonadotropin releasing hormone 1
0.94	CYTL1	cytokine like 1
0.94	ZNF638	Transcript Identified by AceView. Entrez Gene ID(s) 27332
0.94	PRR35	proline rich 35
0.94	CHRNA9	cholinergic receptor. nicotinic alpha 9
0.94	SERTAD4	SERTA domain containing 4
0.94	SNX31	sorting nexin 31
0.94	TFAP2A	transcription factor AP-2 alpha (activating enhancer binding protein 2 alpha)
0.94	RTP2	receptor (chemosensory) transporter protein 2
0.94	FGF12	fibroblast growth factor 12
0.94	TAF13	TAF13 RNA polymerase II. TATA box binding protein (TBP)-associated factor. 18kDa
0.94	SURF4	surfeit 4
0.94	TNFSF18	tumor necrosis factor (ligand) superfamily. member 18
0.94	ABHD14A	abhydrolase domain containing 14A
0.94	UGT3A2	UDP glycosyltransferase 3 family. polypeptide A2
0.94	RAB27B	RAB27B. member RAS oncogene family
0.94	FAM178B	family with sequence similarity 178. member B

0.94	NDUFA6	NADH dehydrogenase (ubiquinone) 1 alpha subcomplex. 6. 14kDa
0.94	RXFP4	relaxin/insulin-like family peptide receptor 4
0.94	DCTN4	dynactin 4 (p62)
0.94	DARS	aspartyl-tRNA synthetase
0.94	GLG1	golgi glycoprotein 1
0.94	FANCB	Fanconi anemia complementation group B
0.94	KLHDC9	kelch domain containing 9
0.94	MEI1	Salzman2013 ALT_ACCEPTOR. ALT_DONOR. coding. INTERNAL. intronic best transcript NM_152513
0.94	STK36	serine/threonine kinase 36
0.94	ZNF613	zinc finger protein 613
0.94	FABP9	fatty acid binding protein 9. testis
0.94	AK5	adenylate kinase 5
0.94	CLEC10A	C-type lectin domain family 10. member A
0.94	B3GNT4	UDP-GlcNAc:betaGal beta-1.3-N-acetylglucosaminyltransferase 4
0.94	ALKBH1	alkB homolog 1. histone H2A dioxygenase
0.94	GNS	glucosamine (N-acetyl)-6-sulfatase
0.94	SURF1	surfeit 1
0.94	SRSF8	serine/arginine-rich splicing factor 8
0.94	TGIF2	TGFB-induced factor homeobox 2
0.94	GFOD1	glucose-fructose oxidoreductase domain containing 1
0.94	HIST1H4F	histone cluster 1. H4f
0.94	TUT1	terminal uridylyl transferase 1. U6 snRNA-specific
0.94	KIAA1143	KIAA1143 [Source:HGNC Symbol;Acc:HGNC:29198]
0.94	LOC101928436; RP11-153F1.1; RP11-347K2.2; teyfawbu	uncharacterized LOC101928436; novel transcript; Transcript Identified by AceView
0.94	PRPF40A	PRP40 pre-mRNA processing factor 40 homolog A

0.94	CLDN16	claudin 16
0.94	NOTCH2NL	notch 2 N-terminal like
0.94	USP17L18; USP17L11	ubiquitin specific peptidase 17-like family member 18; ubiquitin specific peptidase 17-like family member 11
0.94	GPC4	glypican 4
0.94	ANAPC2	anaphase promoting complex subunit 2
0.94	FSHR	follicle stimulating hormone receptor
0.94	NSMCE4A	NSE4 homolog A. SMC5-SMC6 complex component
0.94	C18orf63	chromosome 18 open reading frame 63
0.94	C10orf107	chromosome 10 open reading frame 107
0.94	ZNHIT3	zinc finger. HIT-type containing 3
0.94	SLC22A3	solute carrier family 22 (organic cation transporter). member 3
0.94	FAM171A2	family with sequence similarity 171. member A2
0.94	C19orf18	chromosome 19 open reading frame 18
0.94	POLG	polymerase (DNA directed). gamma
0.94	ATG16L1	autophagy related 16-like 1
0.94	SURF4	surfeit 4
0.94	GNPDA1	glucosamine-6-phosphate deaminase 1
0.94	NPS	neuropeptide S
0.94	PRR13	proline rich 13
0.94	TMEM158	transmembrane protein 158 (gene/pseudogene)
0.94	RBMS1	Jeck2013 ALT_ACCEPTOR. ALT_DONOR. coding. INTERNAL. intronic best transcript NM_016836
0.94	TMEM190	transmembrane protein 190
0.94	PRAMEF15; PRAMEF9	PRAME family member 15; PRAME family member 9
0.94	DMP1	dentin matrix acidic phosphoprotein 1
0.94	NDUFA13	NADH dehydrogenase (ubiquinone) 1 alpha subcomplex. 13

0.94	USP40	ubiquitin specific peptidase 40
0.94	GRAP	GRB2-related adaptor protein
0.94	MAGEB3	Transcript Identified by AceView. Entrez Gene ID(s) 4114
0.94	ABCC1	Jeck2013 ALT_ACCEPTOR. ALT_DONOR. coding. INTERNAL. intronic best transcript NM_004996
0.94	SLC25A18	solute carrier family 25 (glutamate carrier). member 18
0.94	DUSP28	dual specificity phosphatase 28
0.94	GLTPD2	glycolipid transfer protein domain containing 2
0.94	MRS2	MRS2 magnesium transporter
0.94	GRAMD1B	GRAM domain containing 1B
0.94	LOC100131303; CTC-479C5.6	uncharacterized LOC100131303; novel transcript. antisense to DUS2L
0.94	ARHGEF7	Rho guanine nucleotide exchange factor 7
0.94	MAP4K2	Memczak2013 ANTISENSE. CDS. coding. INTERNAL. intronic best transcript NM_004579
0.94	NR1I2	nuclear receptor subfamily 1. group I. member 2
0.94	ACOT12	acyl-CoA thioesterase 12
0.94	SGPP2	sphingosine-1-phosphate phosphatase 2
0.94	QPRT	quinolinate phosphoribosyltransferase
0.94	CCDC47	coiled-coil domain containing 47
0.94	C5orf46	chromosome 5 open reading frame 46
0.94	PRR23A	proline rich 23A
0.94	C20orf85	chromosome 20 open reading frame 85
0.94	LYPLAL1	lysophospholipase-like 1
0.94	SLC38A9	Transcript Identified by AceView. Entrez Gene ID(s) 153129
0.94	ATP6V0D1	ATPase. H+ transporting. lysosomal 38kDa. V0 subunit d1
0.94	HHAT	hedgehog acyltransferase
0.94	TCP11	t-complex 11. testis-specific

0.94	C11orf65	chromosome 11 open reading frame 65
0.94	HPS3	Hermansky-Pudlak syndrome 3
0.94	GLCCI1	glucocorticoid induced 1
0.94	GTF2A2	general transcription factor IIA 2
0.94	BOD1	biorientation of chromosomes in cell division 1
0.94	NOS1AP	nitric oxide synthase 1 (neuronal) adaptor protein
0.94	CYBB	cytochrome b-245. beta polypeptide
0.94	GBGT1	globoside alpha-1.3-N-acetylgalactosaminyltransferase 1
0.94	PABPC5	poly(A) binding protein. cytoplasmic 5
0.94	ICOS	inducible T-cell co-stimulator
0.94	PROP1	PROP paired-like homeobox 1
0.94	LRP12	LDL receptor related protein 12
0.94	TIPIN	TIMELESS interacting protein
0.94	AGRN	agrin
0.94	APEH	acylaminoacyl-peptide hydrolase
0.94	APOL4	apolipoprotein L. 4
0.94	ZNF35	zinc finger protein 35
0.94	FGF10	fibroblast growth factor 10
0.94	C9orf64	chromosome 9 open reading frame 64
0.94	UTF1	undifferentiated embryonic cell transcription factor 1
0.94	TOR2A	torsin family 2. member A
0.94	TBC1D3H; TBC1D3G; TBC1D3I; LOC101060389; TBC1D3C; TBC1D3	Homo sapiens TBC1 domain family. member 3H (TBC1D3H). mRNA.; Homo sapiens TBC1 domain family. member 3G (TBC1D3G). mRNA.; Homo sapiens TBC1 domain family. member 3I (TBC1D3I). mRNA.; Homo sapiens TBC1 domain family member-like (LOC101060389). mRNA.; Homo sapiens TBC1 domain family. member 3C. mRNA (cDNA clone MGC:87891 IMAGE:5165385). complete cds.; Homo sapiens TBC1 domain family. member 3. mRNA (cDNA clone MGC:87893 IMAGE:5742850). complete cds.

0.94	SPATA16	spermatogenesis associated 16
0.94	EZH2	Transcript Identified by AceView. Entrez Gene ID(s) 2146
0.94	GOLGA8A; GOLGA8B	golgin A8 family. member A; golgin A8 family. member B
0.94	UBE2J1	ubiquitin-conjugating enzyme E2. J1
0.94	CD160	CD160 molecule
0.94	ERVW-1	endogenous retrovirus group W. member 1
0.94	SLC25A48	solute carrier family 25. member 48
0.94	PPTC7	PTC7 protein phosphatase homolog
0.94	ZBTB46	zinc finger and BTB domain containing 46
0.94	DUX4L24; DUX4L23	double homeobox 4 like 24; double homeobox 4 like 23
0.94	DUX4L22; DUX4L21	double homeobox 4 like 22; double homeobox 4 like 21
0.94	DUX4L10; DUX4L11	double homeobox 4 like 10; double homeobox 4 like 11
0.94	GRXCR1	glutaredoxin. cysteine rich 1
0.94	NXNL2	nucleoredoxin-like 2
0.94	NOG	noggin
0.94	LILRB5	leukocyte immunoglobulin-like receptor. subfamily B (with TM and ITIM domains). member 5
0.94	POLR2M	polymerase (RNA) II (DNA directed) polypeptide M
0.94	OSBPL11	oxysterol binding protein-like 11
0.94	ASIC5	acid sensing ion channel family member 5
0.94	SPINK2	serine peptidase inhibitor. Kazal type 2 (acrosin-trypsin inhibitor)
0.94	SPATC1	spermatogenesis and centriole associated 1
0.94	RBMY1E; RBMY1D; RBMY1B	RNA binding motif protein. Y-linked. family 1. member E; RNA binding motif protein. Y-linked. family 1. member D; RNA binding motif protein. Y-linked. family 1. member B
0.94	SYNM	synemin. intermediate filament protein
0.94	ZNF717	zinc finger protein 717

0.94	POP1	POP1 homolog. ribonuclease P/MRP subunit
0.94	NPW	neuropeptide W
0.94	ZNF705E	zinc finger protein 705E
0.94	FAM98C	family with sequence similarity 98. member C
0.94	LRRD1	leucine-rich repeats and death domain containing 1
0.94	SRF	Jeck2013 ANTISENSE. CDS. coding. INTERNAL. intronic. OVCODE. OVEXON best transcript NM_003131
0.94	RBBP4	retinoblastoma binding protein 4
0.94	CDH15	cadherin 15. type 1. M-cadherin (myotubule)
0.94	REXO1L2P; REXO1L1; blarbeyby	Homo sapiens REX1. RNA exonuclease 1 homolog-like 2. pseudogene (REXO1L2P). non-coding RNA.; Synthetic construct Homo sapiens clone IMAGE:100068172. MGC:195789 REX1. RNA exonuclease 1 homolog (S. cerevisiae)-like 1 (REXO1L1) mRNA. encodes complete protein.; Transcript Identified by AceView. RefSeq ID(s) NR_003594
0.94	HMBS	hydroxymethylbilane synthase
0.94	SYNPO2	synaptopodin 2
0.94	C19orf25	chromosome 19 open reading frame 25
0.94	TNS2	tensin 2
0.94	OR2Z1	olfactory receptor. family 2. subfamily Z. member 1
0.94	PYGO1	pygopus family PHD finger 1
0.94	VPS35	VPS35 retromer complex component
0.94	SLF1	SMC5-SMC6 complex localization factor 1
0.94	USP36	ubiquitin specific peptidase 36
0.94	GIMAP7	GTPase. IMAP family member 7
0.94	TCL1A	T-cell leukemia/lymphoma 1A
0.94	FOXR1	forkhead box R1
0.94	PTCD3	pentatricopeptide repeat domain 3
0.94	XBP1	X-box binding protein 1

0.94	GNGT2	guanine nucleotide binding protein (G protein). gamma transducing activity polypeptide 2
0.94	SERAC1	serine active site containing 1
0.94	LAPTM4A	lysosomal protein transmembrane 4 alpha
0.94	DEFB133	defensin. beta 133
0.94	NHLRC1	NHL repeat containing E3 ubiquitin protein ligase 1
0.94	HYOU1	hypoxia up-regulated 1
0.94	CRYBA4	crystallin beta A4
0.94	BRD9	Transcript Identified by AceView. Entrez Gene ID(s) 65980
0.94	PEX16	peroxisomal biogenesis factor 16
0.94	MED14	mediator complex subunit 14
0.94	MAZ	MYC-associated zinc finger protein (purine-binding transcription factor)
0.94	C12orf65	chromosome 12 open reading frame 65
0.94	HSPB6	heat shock protein. alpha-crystallin-related. B6
0.94	PPP1R2	protein phosphatase 1. regulatory (inhibitor) subunit 2
0.94	SURF4	surfeit 4
0.94	AADAC	arylacetamide deacetylase
0.94	TMEM8A	transmembrane protein 8A
0.94	IRX4	iroquois homeobox 4
0.94	MDS2	myelodysplastic syndrome 2 translocation associated
0.94	QRICH1	glutamine-rich 1
0.94	LARGE	like-glycosyltransferase
0.94	ATF7IP	activating transcription factor 7 interacting protein
0.94	COG7	component of oligomeric golgi complex 7
0.94	C5orf49	chromosome 5 open reading frame 49
0.94	SPRED3	sprouty-related. EVH1 domain containing 3

0.94	KIAA1147	KIAA1147
0.94	HEPACAM2	HEPACAM family member 2
0.94	POLD1	polymerase (DNA directed). delta 1. catalytic subunit
0.94	PTK2	Transcript Identified by AceView. Entrez Gene ID(s) 5747
0.94	MRPL50	mitochondrial ribosomal protein L50
0.94	TMEM127	transmembrane protein 127
0.94	ZNF33A	zinc finger protein 33A
0.94	SYN3	synapsin III
0.94	GYPE	glycophorin E (MNS blood group)
0.94	CSF3R	Memczak2013 ANTISENSE. CDS. coding. INTERNAL best transcript NM_156039
0.94	GABBR1	gamma-aminobutyric acid (GABA) B receptor. 1
0.94	KU-MEL-3; RP4-529N6.1	uncharacterized LOC497048; uncharacterized LOC497048 [Source:EntrezGene;Acc:497048]; putative novel transcript
0.94	RPL26L1	ribosomal protein L26-like 1
0.94	C20orf203	chromosome 20 open reading frame 203
0.94	CLEC11A	C-type lectin domain family 11. member A
0.94	LRRC14	leucine rich repeat containing 14
0.94	CES4A	carboxylesterase 4A
0.94	PNRC2	proline-rich nuclear receptor coactivator 2
0.94	ZNF175	zinc finger protein 175
0.94	NUCB2	nucleobindin 2
0.94	ARHGEF17	Rho guanine nucleotide exchange factor 17
0.94	VPS25	vacuolar protein sorting 25 homolog (S. cerevisiae)
0.94	IL10	interleukin 10
0.94	WNK1	Transcript Identified by AceView. Entrez Gene ID(s) 378465; 65125
0.94	EPN3	epsin 3

0.94	PROZ	protein Z. vitamin K-dependent plasma glycoprotein
0.94	C16orf96	chromosome 16 open reading frame 96
0.94	GDPD3	glycerophosphodiester phosphodiesterase domain containing 3
0.94	MC5R	melanocortin 5 receptor
0.94	DROSHA	drosha. ribonuclease type III
0.94	CKLF-CMTM1	CKLF-CMTM1 readthrough
0.94	AUTS2	autism susceptibility candidate 2
0.94	TBCD	tubulin folding cofactor D
0.94	MTRNR2L5	MT-RNR2-like 5
0.94	CARD19	caspase recruitment domain family. member 19
0.94	C11orf70	chromosome 11 open reading frame 70
0.94	PTPRB	protein tyrosine phosphatase. receptor type. B
0.94	BHLHB9	basic helix-loop-helix domain containing. class B. 9
0.94	NADSYN1	NAD synthetase 1
0.94	C9orf24	chromosome 9 open reading frame 24
0.94	KIT	v-kit Hardy-Zuckerman 4 feline sarcoma viral oncogene homolog
0.94	MBOAT7	membrane bound O-acyltransferase domain containing 7
0.94	MAGEA4	MAGE family member A4
0.94	GALC	galactosylceramidase
0.94	TMED10	transmembrane p24 trafficking protein 10
0.94	ATG2A	autophagy related 2A
0.94	NEK5	NIMA-related kinase 5
0.94	RPS6KA1	ribosomal protein S6 kinase. 90kDa. polypeptide 1
0.94	ERBB3	erb-b2 receptor tyrosine kinase 3
0.94	COL1A2	collagen. type I. alpha 2
0.94	DYRK2	dual specificity tyrosine-(Y)-phosphorylation regulated kinase 2

0.94	CLEC2A	C-type lectin domain family 2. member A
0.94	DLX1	distal-less homeobox 1
0.94	CUZD1	CUB and zona pellucida-like domains 1
0.94	COL15A1	collagen. type XV. alpha 1
0.94	PEX19	peroxisomal biogenesis factor 19
0.94	ZFP57	ZFP57 zinc finger protein
0.94	CAMK1	calcium/calmodulin-dependent protein kinase I
0.94	IFNW1	interferon. omega 1
0.94	LYL1	lymphoblastic leukemia associated hematopoiesis regulator 1
0.94	ITGB2	integrin. beta 2 (complement component 3 receptor 3 and 4 subunit)
0.94	ZCRB1; MIR7851	zinc finger CCHC-type and RNA binding motif 1; microRNA 7851
0.94	RAB19	RAB19. member RAS oncogene family
0.94	GTF3C2	general transcription factor IIIC subunit 2
0.94	OR13C3	olfactory receptor. family 13. subfamily C. member 3
0.94	SCRT2	scratch family zinc finger 2
0.94	PSMC4	proteasome 26S subunit. ATPase 4 [Source:HGNC Symbol;Acc:HGNC:9551]
0.94	C11orf68	chromosome 11 open reading frame 68
0.93	NODAL	nodal growth differentiation factor
0.93	SLC25A53	solute carrier family 25. member 53
0.93	ZSCAN32	zinc finger and SCAN domain containing 32
0.93	LRRC26	leucine rich repeat containing 26
0.93	LYG1	lysozyme G-like 1
0.93	CRADD	CASP2 and RIPK1 domain containing adaptor with death domain
0.93	SEC22B	SEC22 homolog B. vesicle trafficking protein (gene/pseudogene)
0.93	ZNF852	zinc finger protein 852
0.93	SIGLEC15	sialic acid binding Ig-like lectin 15

0.93	BBIP1	BBSome interacting protein 1
0.93	DEFB103A	defensin. beta 103A
0.93	MEIS1	Transcript Identified by AceView. Entrez Gene ID(s) 4211
0.93	TTF1	transcription termination factor. RNA polymerase I
0.93	CMAS	cytidine monophosphate N-acetylneuraminic acid synthetase
0.93	FBXO44	F-box protein 44
0.93	AHSG	alpha-2-HS-glycoprotein
0.93	VCL	vinculin
0.93	DENND6A	DENN/MADD domain containing 6A
0.93	DNAH12	dynein. axonemal. heavy chain 12
0.93	ART3	ADP-ribosyltransferase 3
0.93	OR10J1	olfactory receptor. family 10. subfamily J. member 1
0.93	HHAT	hedgehog acyltransferase
0.93	JPH4	junctophilin 4
0.93	POGZ	Transcript Identified by AceView. Entrez Gene ID(s) 23126
0.93	LRRC74B	leucine rich repeat containing 74B
0.93	CHRD	chordin
0.93	HNRNPCL2	heterogeneous nuclear ribonucleoprotein C-like 2
0.93	CNGB3	cyclic nucleotide gated channel beta 3
0.93	CA6	carbonic anhydrase VI
0.93	DPF1	D4. zinc and double PHD fingers family 1
0.93	ZMPSTE24	zinc metallopeptidase STE24
0.93	SAMD13	sterile alpha motif domain containing 13
0.93	PSPH	phosphoserine phosphatase
0.93	FGF17	fibroblast growth factor 17
0.93	MFSD4	major facilitator superfamily domain containing 4

0.93	NDNL2	necdin-like 2. SMC5-SMC6 complex component
0.93	GABRP	gamma-aminobutyric acid (GABA) A receptor. pi
0.93	C9orf3; MIR24-1; MIR23B; MIR27B	chromosome 9 open reading frame 3; microRNA 24-1; microRNA 23b; microRNA 27b
0.93	FKBP1B	FK506 binding protein 1B
0.93	OR11H2	olfactory receptor. family 11. subfamily H. member 2
0.93	PPP1R35	protein phosphatase 1. regulatory subunit 35
0.93	PILRA	paired immunoglobin-like type 2 receptor alpha
0.93	TSPY1	testis specific protein. Y-linked 1
0.93	ALG2	ALG2. alpha-1.3/1.6-mannosyltransferase
0.93	HERC5	HECT and RLD domain containing E3 ubiquitin protein ligase 5
0.93	GLRA3	glycine receptor alpha 3
0.93	TTC31	tetratricopeptide repeat domain 31
0.93	GAA	glucosidase. alpha; acid
0.93	FBP2	fructose-1.6-bisphosphatase 2
0.93	SECISBP2	SECIS binding protein 2
0.93	SAC3D1	SAC3 domain containing 1
0.93	HRAS	Harvey rat sarcoma viral oncogene homolog
0.93	XRCC6BP1	XRCC6 binding protein 1
0.93	LINC00346	long intergenic non-protein coding RNA 346
0.93	TMEM189-UBE2V1	TMEM189-UBE2V1 readthrough
0.93	B4GALT1	UDP-Gal:betaGlcNAc beta 1.4- galactosyltransferase. polypeptide 1
0.93	HEATR4	HEAT repeat containing 4
0.93	TRIML1	tripartite motif family like 1
0.93	ZNF780B	zinc finger protein 780B
0.93	MTUS2	microtubule associated tumor suppressor candidate 2

0.93	CCDC185	coiled-coil domain containing 185
0.93	TRIM29	tripartite motif containing 29
0.93	CACFD1	calcium channel flower domain containing 1
0.93	SPATA22	spermatogenesis associated 22
0.93	ATAD3A	ATPase family. AAA domain containing 3A
0.93	IZUMO3	IZUMO family member 3
0.93	COMMD6	COMM domain containing 6
0.93	ETV5	ets variant 5
0.93	HEMK1	HemK methyltransferase family member 1
0.93	ASAP1	Memczak2013 ALT_ACCEPTOR. ALT_DONOR. coding. INTERNAL. intronic best transcript NM_018482
0.93	NBPF14	neuroblastoma breakpoint family. member 14
0.93	DUSP15	dual specificity phosphatase 15
0.93	EIF4E2	eukaryotic translation initiation factor 4E family member 2
0.93	PRPSAP2	phosphoribosyl pyrophosphate synthetase-associated protein 2
0.93	ARFGAP1; MIR4326	ADP-ribosylation factor GTPase activating protein 1; microRNA 4326
0.93	DACT3	dishevelled-binding antagonist of beta-catenin 3
0.93	APOBEC3A	apolipoprotein B mRNA editing enzyme. catalytic polypeptide-like 3A
0.93	SNTG2	syntrophin. gamma 2
0.93	CNTNAP3	contactin associated protein-like 3
0.93	HYOU1	hypoxia up-regulated 1
0.93	IQCK	IQ motif containing K
0.93	ADAM30	ADAM metallopeptidase domain 30
0.93	FGFRL1	fibroblast growth factor receptor-like 1
0.93	VGLL2	vestigial-like family member 2
0.93	C7orf31	chromosome 7 open reading frame 31

0.93	DNAJC1	DnaJ (Hsp40) homolog. subfamily C. member 1
0.93	PLPP3	phospholipid phosphatase 3
0.93	TMEM147-AS1	TMEM147 antisense RNA 1
0.93	RILP	Rab interacting lysosomal protein
0.93	IQCJ; SCHIP1; IQCJ-SCHIP1	IQ motif containing J; schwannomin interacting protein 1; IQCJ-SCHIP1 readthrough
0.93	AKT3	v-akt murine thymoma viral oncogene homolog 3
0.93	TMEM52	transmembrane protein 52
0.93	UBE2D3	Memczak2013 ANTISENSE. coding. INTERNAL. intronic best transcript NM_181893
0.93	LINC00885	long intergenic non-protein coding RNA 885
0.93	CCM2L	cerebral cavernous malformation 2-like
0.93	ACSM4	acyl-CoA synthetase medium-chain family member 4
0.93	ZNF420	zinc finger protein 420
0.93	TEX40	testis expressed 40
0.93	DRD2	dopamine receptor D2
0.93	CYHR1	cysteine/histidine-rich 1
0.93	MC2R	melanocortin 2 receptor (adrenocorticotropic hormone)
0.93	RPS6KA5	ribosomal protein S6 kinase. 90kDa. polypeptide 5
0.93	ZNF346	zinc finger protein 346
0.93	PPFIA1	protein tyrosine phosphatase. receptor type. f polypeptide (PTPRF). interacting protein (liprin). alpha 1
0.93	PAX4	paired box 4
0.93	RAB33A	RAB33A. member RAS oncogene family
0.93	TSHR	thyroid stimulating hormone receptor
0.93	MANSC1	MANSC domain containing 1
0.93	NUDT6	nudix hydrolase 6

0.93	ZNF707	zinc finger protein 707
0.93	MYADML2	myeloid-associated differentiation marker-like 2
0.93	IGFBP4	insulin like growth factor binding protein 4
0.93	ZSCAN20	zinc finger and SCAN domain containing 20
0.93	MIR22HG	MIR22 host gene
0.93	SPON1	spondin 1. extracellular matrix protein
0.93	YIPF6	Yip1 domain family member 6
0.93	IGFN1	immunoglobulin-like and fibronectin type III domain containing 1
0.93	SKIV2L	SKI2 homolog. superkiller viralicidic activity 2-like
0.93	ARID4B	AT rich interactive domain 4B (RBP1-like)
0.93	NR0B2	nuclear receptor subfamily 0. group B. member 2
0.93	PCSK7	proprotein convertase subtilisin/kexin type 7
0.93	DPYSL5	dihydropyrimidinase-like 5
0.93	SRP72	Memczak2013 ANTISENSE. coding. INTERNAL. intronic best transcript NM_006947
0.93	TAS1R1	taste receptor. type 1. member 1
0.93	NDEL1	nudE neurodevelopment protein 1-like 1
0.93	C12orf54	chromosome 12 open reading frame 54
0.93	PREX1	phosphatidylinositol-3.4.5-trisphosphate-dependent Rac exchange factor 1
0.93	ING4	inhibitor of growth family member 4
0.93	PRKCSH	protein kinase C substrate 80K-H
0.93	MAML3	Memczak2013 ALT_ACCEPTOR. ALT_DONOR. coding. INTERNAL. intronic best transcript NM_018717
0.93	SDCCAG3	serologically defined colon cancer antigen 3
0.93	SLC13A4	solute carrier family 13 (sodium/sulfate symporter). member 4
0.93	PACSIN3	protein kinase C and casein kinase substrate in neurons 3
0.93	CMPK1	cytidine monophosphate (UMP-CMP) kinase 1. cytosolic

0.93	CAMK2N2	calcium/calmodulin-dependent protein kinase II inhibitor 2
0.93	TPRN	taperin
0.93	TRPC4AP	transient receptor potential cation channel. subfamily C. member 4 associated protein
0.93	ZNF780A	zinc finger protein 780A
0.93	MCRS1	microspherule protein 1
0.93	FBXL6	F-box and leucine-rich repeat protein 6
0.93	RIN1	Ras and Rab interactor 1
0.93	IFNA4	interferon. alpha 4
0.93	DENND1A	DENN/MADD domain containing 1A
0.93	LINC01578	long intergenic non-protein coding RNA 1578
0.93	C22orf39; HIRA	chromosome 22 open reading frame 39; histone cell cycle regulator
0.93	TMEM258; MIR611	transmembrane protein 258; microRNA 611
0.93	FAM218A	family with sequence similarity 218. member A
0.93	TMEM132E	transmembrane protein 132E
0.93	FAT2	FAT atypical cadherin 2
0.93	LINC01600	long intergenic non-protein coding RNA 1600
0.93	NT5C	5. 3-nucleotidase. cytosolic
0.93	REXO1L4P	REX1. RNA exonuclease 1 homolog-like 4. pseudogene
0.93	CCDC113	coiled-coil domain containing 113
0.93	TUBA3D	tubulin. alpha 3d
0.93	REP15	RAB15 effector protein
0.93	PARVA	parvin. alpha
0.93	ZNF852	zinc finger protein 852
0.93	RPGRIP1	retinitis pigmentosa GTPase regulator interacting protein 1
0.93	FAM83F	family with sequence similarity 83. member F

0.93	ATXN3	ataxin 3
0.93	KRTAP19-2	keratin associated protein 19-2
0.93	CPNE9	copine family member IX
0.93	IL16	interleukin 16
0.93	LYZ	lysozyme
0.93	ARHGAP6	Rho GTPase activating protein 6
0.93	RHNO1	RAD9-HUS1-RAD1 interacting nuclear orphan 1
0.93	ERICH6B	glutamate rich 6B
0.93	PMP22	peripheral myelin protein 22
0.93	BEAN1	brain expressed. associated with NEDD4. 1
0.93	GLT8D1	glycosyltransferase 8 domain containing 1
0.93	N4BP3	NEDD4 binding protein 3
0.93	IFI35	interferon-induced protein 35
0.93	TELO2	telomere maintenance 2
0.93	HEY2	hes-related family bHLH transcription factor with YRPW motif 2
0.93	VTCN1	V-set domain containing T cell activation inhibitor 1
0.93	SGCZ	sarcoglycan zeta
0.93	TTYH3	tweety family member 3
0.93	NKX6-2	NK6 homeobox 2
0.93	VWC2L	von Willebrand factor C domain containing protein 2-like
0.93	PRDM2	PR domain containing 2. with ZNF domain
0.93	GIN1	gypsy retrotransposon integrase 1
0.93	TMEM88	transmembrane protein 88
0.93	ALPK1	alpha kinase 1
0.93	CA10	carbonic anhydrase X
0.93	MTFP1	mitochondrial fission process 1

0.93	OR52I1	olfactory receptor. family 52. subfamily I. member 1
0.93	LMO1	LIM domain only 1 (rhombotin 1)
0.93	HDHD2	haloacid dehalogenase-like hydrolase domain containing 2
0.93	PDZRN4	PDZ domain containing ring finger 4
0.93	ATP6V1B1	ATPase. H+ transporting. lysosomal 56/58kDa. V1 subunit B1
0.93	SOCS7	suppressor of cytokine signaling 7
0.93	SIGLEC8	sialic acid binding Ig-like lectin 8
0.93	C15orf53	chromosome 15 open reading frame 53
0.93	PRMT2	protein arginine methyltransferase 2
0.93	FGF13; LINC00889	fibroblast growth factor 13; long intergenic non-protein coding RNA 889
0.93	TCERG1L	transcription elongation regulator 1-like
0.93	IGF1	insulin-like growth factor 1 (somatomedin C)
0.93	DNAH11	dynein. axonemal. heavy chain 11
0.93	GFY	golgi-associated. olfactory signaling regulator
0.93	LINC01547	long intergenic non-protein coding RNA 1547
0.93	MGRN1	mahogunin ring finger 1. E3 ubiquitin protein ligase
0.93	PYGL	phosphorylase. glycogen. liver
0.93	ZMYND8	zinc finger. MYND-type containing 8
0.93	SKIV2L2	superkiller viralicidic activity 2-like 2 (S. cerevisiae)
0.93	CMA1	chymase 1. mast cell
0.93	TBC1D14	TBC1 domain family. member 14
0.93	FKBP15	FK506 binding protein 15
0.93	MACROD2; RPS3P1	MACRO domain containing 2; ribosomal protein S3 pseudogene 1
0.93	ZNF718	zinc finger protein 718
0.93	CABP7	calcium binding protein 7
0.93	NKX1-1	NK1 homeobox 1

0.93	PSMC1	proteasome 26S subunit. ATPase 1
0.93	LACRT	lacritin
0.93	RASL11B	RAS-like. family 11. member B
0.93	DND1	DND microRNA-mediated repression inhibitor 1
0.93	MAP7D3	MAP7 domain containing 3
0.93	USP35	ubiquitin specific peptidase 35
0.93	HINT3	histidine triad nucleotide binding protein 3
0.93	ME3	malic enzyme 3. NADP(+)-dependent. mitochondrial
0.93	DPP3	dipeptidyl-peptidase 3
0.93	TSPY3	testis specific protein. Y-linked 3
0.93	PBX1	pre-B-cell leukemia homeobox 1
0.93	ZDHHC3	zinc finger. DHHC-type containing 3
0.93	MARCH9	membrane associated ring finger 9
0.93	MAP2	microtubule associated protein 2
0.93	CDH24	cadherin 24. type 2
0.93	GPR27	G protein-coupled receptor 27
0.93	PSORS1C2	psoriasis susceptibility 1 candidate 2
0.93	ZNF587B	zinc finger protein 587B
0.93	CDK16	cyclin-dependent kinase 16
0.93	AP5Z1	adaptor-related protein complex 5. zeta 1 subunit
0.93	CNTF	ciliary neurotrophic factor
0.93	ZKSCAN5	zinc finger with KRAB and SCAN domains 5
0.93	DPP4	Transcript Identified by AceView. Entrez Gene ID(s) 1803
0.93	MGAT5	mannosyl (alpha-1.6-)-glycoprotein beta-1.6-N-acetyl-glucosaminyltransferase
0.93	AMIGO3	adhesion molecule with Ig-like domain 3

0.93	TNRC6A	trinucleotide repeat containing 6A
0.93	PPIAP30	peptidylprolyl isomerase A (cyclophilin A) pseudogene 30
0.93	DDRGK1	DDRGK domain containing 1
0.93	ZNF254	zinc finger protein 254
0.93	NUGGC	nuclear GTPase. germinal center associated
0.93	EZH1	enhancer of zeste 1 polycomb repressive complex 2 subunit
0.93	MGAM2	maltase-glucoamylase 2 (putative)
0.93	PNN	pinin. desmosome associated protein
0.93	IFNA5	interferon. alpha 5
0.93	ZBTB21	zinc finger and BTB domain containing 21
0.93	SNED1	Memczak2013 ALT_DONOR. coding. INTERNAL. intronic best transcript NM_001080437
0.93	SRF	serum response factor
0.93	FAM86B2	family with sequence similarity 86. member B2
0.93	ALX1	ALX homeobox 1
0.93	DBH	dopamine beta-hydroxylase (dopamine beta-monooxygenase)
0.93	TEAD3	TEA domain family member 3
0.93	TAS2R46	taste receptor. type 2. member 46
0.93	ARF1; MIR3620	ADP-ribosylation factor 1; microRNA 3620
0.93	KCTD6	potassium channel tetramerization domain containing 6
0.93	ATP5A1	ATP synthase. H+ transporting. mitochondrial F1 complex. alpha subunit 1. cardiac muscle
0.93	C9orf40	chromosome 9 open reading frame 40
0.93	HOXD13	homeobox D13
0.93	PLA2G1B	phospholipase A2. group IB (pancreas)
0.93	KRTAP12-1	keratin associated protein 12-1
0.93	ZSCAN23	zinc finger and SCAN domain containing 23

0.93	ANKRD36	ankyrin repeat domain 36
0.93	EPHB1	EPH receptor B1
0.93	NAA11	N(alpha)-acetyltransferase 11. NatA catalytic subunit
0.93	TCP10	t-complex 10
0.93	STOML3	stomatin (EPB72)-like 3
0.93	OR4M2	olfactory receptor. family 4. subfamily M. member 2
0.93	FANCC	Fanconi anemia complementation group C
0.93	HPS6	Hermansky-Pudlak syndrome 6
0.93	DRG1	developmentally regulated GTP binding protein 1
0.93	MGAT4D	MGAT4 family. member D
0.93	KLK13	kallikrein related peptidase 13
0.93	KIAA1468	KIAA1468
0.93	PRKCA	Jeck2013 ALT_ACCEPTOR. ALT_DONOR. coding. INTERNAL. intronic best transcript NM_002737
0.93	PRY; PRY2	PTPN13-like. Y-linked; PTPN13-like. Y-linked 2
0.93	PLEKHA3	Transcript Identified by AceView. Entrez Gene ID(s) 65977
0.93	RETSAT	retinol saturase (all-trans-retinol 13.14-reductase)
0.93	CANX	calnexin
0.93	NHSL2	NHS-like 2
0.93	RBMY1F; RBMY2FP; RBMY1J	RNA binding motif protein. Y-linked. family 1. member F; RNA binding motif protein. Y-linked. family 2. member F pseudogene; RNA binding motif protein. Y-linked. family 1. member J
0.93	TTC21A; MIR6822	tetratricopeptide repeat domain 21A; microRNA 6822
0.93	AGBL1	ATP/GTP binding protein-like 1
0.93	sept-08	septin 8
0.93	PDE4C	phosphodiesterase 4C. cAMP-specific
0.93	FIG4	FIG4 phosphoinositide 5-phosphatase

0.93	MAP6D1	MAP6 domain containing 1
0.93	SLC6A16	solute carrier family 6. member 16
0.93	ALK	anaplastic lymphoma receptor tyrosine kinase
0.93	INSIG2	insulin induced gene 2
0.93	KHDRBS2	KH domain containing. RNA binding. signal transduction associated 2
0.93	CCL8	chemokine (C-C motif) ligand 8
0.93	FBXL14	F-box and leucine-rich repeat protein 14
0.93	C2orf83	chromosome 2 open reading frame 83
0.93	RCHY1	ring finger and CHY zinc finger domain containing 1. E3 ubiquitin protein ligase
0.93	REPS2	RALBP1 associated Eps domain containing 2
0.93	ZNF460	zinc finger protein 460
0.93	GNAT2	guanine nucleotide binding protein (G protein). alpha transducing activity polypeptide 2
0.93	KCTD15	potassium channel tetramerization domain containing 15
0.93	DYNLL2	dynein. light chain. LC8-type 2
0.93	NUFIP1	nuclear fragile X mental retardation protein interacting protein 1
0.93	CHEK2	checkpoint kinase 2
0.93	ARL14EPL	ADP-ribosylation factor like GTPase 14 effector protein like
0.93	ZIM3	zinc finger. imprinted 3
0.93	ZNF638	zinc finger protein 638
0.93	CLCN5	chloride channel. voltage-sensitive 5
0.93	MAML2	mastermind-like transcriptional coactivator 2
0.93	BTN3A1	butyrophilin. subfamily 3. member A1
0.93	MCHR1	melanin-concentrating hormone receptor 1
0.93	CADM4	cell adhesion molecule 4
0.93	ZNF197	zinc finger protein 197

0.93	MEX3A	mex-3 RNA binding family member A
0.93	TRIM51	tripartite motif-containing 51
0.93	IL17RC	interleukin 17 receptor C
0.93	LHX2	LIM homeobox 2
0.93	HAUS6	HAUS augmin like complex subunit 6
0.93	GSX2	GS homeobox 2
0.93	IRF2	interferon regulatory factor 2
0.93	URB1	URB1 ribosome biogenesis 1 homolog (S. cerevisiae)
0.93	R3HDML	R3H domain containing-like
0.93	TBX10	T-box 10
0.93	PPP1R14A	protein phosphatase 1. regulatory (inhibitor) subunit 14A
0.93	GRM4	glutamate receptor. metabotropic 4
0.93	LRTM1	leucine-rich repeats and transmembrane domains 1
0.93	PRAMEF6	PRAME family member 6 [Source:HGNC Symbol;Acc:HGNC:30583]
0.93	PRAMEF6	PRAME family member 6 [Source:HGNC Symbol;Acc:HGNC:30583]
0.93	LIG4	ligase IV. DNA. ATP-dependent
0.93	SKA2	spindle and kinetochore associated complex subunit 2
0.93	POLA1	polymerase (DNA directed). alpha 1. catalytic subunit
0.93	CAMK2B	calcium/calmodulin-dependent protein kinase II beta
0.93	ADGRB2	adhesion G protein-coupled receptor B2
0.93	TMEM47	transmembrane protein 47
0.93	ELOVL6	ELOVL fatty acid elongase 6
0.93	SKI	SKI proto-oncogene
0.93	MMP19	matrix metallopeptidase 19
0.93	EGFLAM	EGF-like. fibronectin type III and laminin G domains
0.93	FFAR3	free fatty acid receptor 3

0.93	PI4KAP2	phosphatidylinositol 4-kinase. catalytic. alpha pseudogene 2
0.93	SOHLH2	spermatogenesis and oogenesis specific basic helix-loop-helix 2
0.93	MYO3A	myosin IIIA
0.93	SLC29A3	Transcript Identified by AceView. Entrez Gene ID(s) 55315
0.93	PCF11	PCF11 cleavage and polyadenylation factor subunit
0.93	ARV1	ARV1 homolog. fatty acid homeostasis modulator
0.93	SGCA	sarcoglycan alpha
0.93	SERGEF	secretion regulating guanine nucleotide exchange factor
0.93	CPXM2	carboxypeptidase X (M14 family). member 2
0.93	BRI3	brain protein I3
0.93	DIO2	deiodinase. iodothyronine. type II
0.93	SZRD1	SUZ RNA binding domain containing 1
0.93	CORO2B	Memczak2013 ALT_ACCEPTOR. ALT_DONOR. coding. INTERNAL. intronic best transcript NM_001190457
0.93	MICA	MHC class I polypeptide-related sequence A
0.93	VBP1	von Hippel-Lindau binding protein 1
0.93	SCAF1	SR-related CTD-associated factor 1
0.93	PRSS58	protease. serine. 58
0.93	PSD	pleckstrin and Sec7 domain containing
0.93	AKIRIN1	akirin 1
0.93	IGSF23	immunoglobulin superfamily. member 23
0.93	INO80	INO80 complex subunit
0.93	EPB41	Memczak2013 ALT_ACCEPTOR. ALT_DONOR. coding. INTERNAL. intronic best transcript NM_001166007
0.93	CDK9	cyclin-dependent kinase 9
0.93	C3orf36	chromosome 3 open reading frame 36
0.93	MBD1	methyl-CpG binding domain protein 1

0.93	LRRC61	leucine rich repeat containing 61
0.93	C7orf69	chromosome 7 open reading frame 69
0.93	MAP3K2	mitogen-activated protein kinase kinase kinase 2
0.93	CRHR2	corticotropin releasing hormone receptor 2
0.93	DHX40	DEAH (Asp-Glu-Ala-His) box polypeptide 40
0.93	SERPINF1	serpin peptidase inhibitor. clade F (alpha-2 antiplasmin. pigment epithelium derived factor). member 1
0.93	PRB1	proline-rich protein BstNI subfamily 1
0.93	FCN1	ficolin (collagen/fibrinogen domain containing) 1
0.93	LOC101927844; serfawbu	uncharacterized LOC101927844; Transcript Identified by AceView
0.93	TRIM26	tripartite motif containing 26
0.93	FCRL3	Fc receptor-like 3
0.93	TMEM191B	transmembrane protein 191B
0.93	PCDHB16	protocadherin beta 16
0.93	YWHAQ	tyrosine 3-monooxygenase/tryptophan 5-monooxygenase activation protein. theta
0.93	RABGGTA	Rab geranylgeranyltransferase. alpha subunit
0.93	EIF2AK4	eukaryotic translation initiation factor 2 alpha kinase 4
0.93	GZF1	GDNF-inducible zinc finger protein 1
0.93	DYNC2LI1	dynein. cytoplasmic 2. light intermediate chain 1
0.93	OR8U1; OR8U8	olfactory receptor. family 8. subfamily U. member 1; olfactory receptor. family 8. subfamily U. member 8
0.93	HSPA1L	heat shock 70kDa protein 1-like
0.93	AGPAT3	1-acylglycerol-3-phosphate O-acyltransferase 3
0.93	TYMS	thymidylate synthetase
0.93	VPREB3	pre-B lymphocyte 3
0.93	AVP	arginine vasopressin

0.93	ATP13A1	ATPase type 13A1
0.93	ANKRD33	ankyrin repeat domain 33
0.93	AGPS	alkylglycerone phosphate synthase
0.93	FAM229A	family with sequence similarity 229. member A
0.93	G0S2	G0/G1 switch 2
0.93	GRID1	glutamate receptor. ionotropic. delta 1
0.93	MLXIP	MLX interacting protein
0.93	IFT22	intraflagellar transport 22
0.93	TBC1D9	Memczak2013 ALT_ACCEPTOR. ALT_DONOR. coding. INTERNAL. intronic best transcript NM_015130
0.93	C2CD2L	C2CD2-like
0.93	PARVG	parvin. gamma
0.93	NTF3	neurotrophin 3
0.93	DFNB31	deafness. autosomal recessive 31
0.93	ZNF419	zinc finger protein 419
0.93	RPS6KA1	ribosomal protein S6 kinase. 90kDa. polypeptide 1
0.93	HSFX1	heat shock transcription factor family. X-linked 1
0.93	KDM3B	lysine (K)-specific demethylase 3B
0.93	AGBL5	ATP/GTP binding protein-like 5
0.93	NOTCH2NL	notch 2 N-terminal like
0.93	KRT35	keratin 35. type I
0.93	LEMD1	LEM domain containing 1
0.93	TIMP4	TIMP metallopeptidase inhibitor 4
0.93	TYROBP	TYRO protein tyrosine kinase binding protein
0.93	RASGRF2	Jeck2013 ALT_ACCEPTOR. ALT_DONOR. coding. INTERNAL. intronic best transcript NM_006909
0.93	ZDHHC3	zinc finger. DHHC-type containing 3

0.93	TMEM245	transmembrane protein 245
0.93	DPPA2	developmental pluripotency associated 2
0.93	PTMS	parathymosin
0.93	RNF133	ring finger protein 133
0.93	ZNF197	zinc finger protein 197
0.93	USH1C	Usher syndrome 1C
0.93	URM1	ubiquitin related modifier 1
0.93	SURF4	surfeit 4
0.93	NBPF12	neuroblastoma breakpoint family. member 12
0.93	CCNYL1; MIR4775	cyclin Y like 1; microRNA 4775
0.93	SPIB	Spi-B transcription factor (Spi-1/PU.1 related)
0.93	ASCL2	achaete-scute family bHLH transcription factor 2
0.93	FARP2	FERM. ARH/RhoGEF and pleckstrin domain protein 2
0.93	CXCL14	chemokine (C-X-C motif) ligand 14
0.93	MGARP	mitochondria localized glutamic acid rich protein
0.93	FAM19A5	family with sequence similarity 19 (chemokine (C-C motif)-like). member A5
0.93	TYW1B	tRNA-yW synthesizing protein 1 homolog B (S. cerevisiae)
0.93	OR7A5	olfactory receptor. family 7. subfamily A. member 5
0.93	FAM46B	family with sequence similarity 46. member B
0.93	PRMT1	protein arginine methyltransferase 1
0.93	MYCN	v-myc avian myelocytomatosis viral oncogene neuroblastoma derived homolog
0.93	PMP2	peripheral myelin protein 2
0.93	POLR2C	polymerase (RNA) II (DNA directed) polypeptide C. 33kDa
0.93	KLHL32	kelch-like family member 32
0.93	HDAC3	histone deacetylase 3

0.93	ATM	ATM serine/threonine kinase
0.93	SNAPC4	small nuclear RNA activating complex polypeptide 4
0.93	ATP8A1	ATPase. aminophospholipid transporter (APLT). class I. type 8A. member 1
0.93	NDUFA11	NADH dehydrogenase (ubiquinone) 1 alpha subcomplex. 11. 14.7kDa
0.93	C19orf35	chromosome 19 open reading frame 35
0.93	SLC16A8	solute carrier family 16 (monocarboxylate transporter). member 8
0.93	LIFR	leukemia inhibitory factor receptor alpha
0.93	CELA2A	chymotrypsin-like elastase family. member 2A
0.93	EIF2AK1	eukaryotic translation initiation factor 2-alpha kinase 1
0.93	KIRREL3-AS3	KIRREL3 antisense RNA 3
0.93	SSX2B	synovial sarcoma. X breakpoint 2B
0.93	ZNF780A	zinc finger protein 780A
0.93	TTI1	TELO2 interacting protein 1
0.93	ZNF35	zinc finger protein 35
0.93	ST7-AS1	ST7 antisense RNA 1
0.93	NXPE2	neurexophilin and PC-esterase domain family. member 2
0.93	SAA1	serum amyloid A1
0.93	FOXA2	forkhead box A2
0.93	COLEC12	collectin sub-family member 12
0.93	ATRIP; TREX1	ATR interacting protein; three prime repair exonuclease 1
0.93	C2orf73	chromosome 2 open reading frame 73
0.93	POU4F2	POU class 4 homeobox 2
0.93	LINC00337	long intergenic non-protein coding RNA 337
0.93	GAB3	GRB2-associated binding protein 3
0.93	TAS2R10	taste receptor. type 2. member 10
0.93	NSRP1; MIR423	nuclear speckle splicing regulatory protein 1; microRNA 423

0.93	ELMOD3	Transcript Identified by AceView. Entrez Gene ID(s) 84173
0.93	KRT1	keratin 1. type II
0.93	GNA14	guanine nucleotide binding protein (G protein). alpha 14
0.93	AKNAD1	AKNA domain containing 1
0.93	OR10G3	olfactory receptor. family 10. subfamily G. member 3
0.93	KRTAP6-3	keratin associated protein 6-3
0.93	RNF181	ring finger protein 181
0.93	sept-14	septin 14
0.93	PADI4	peptidyl arginine deiminase. type IV
0.93	SLC39A13	solute carrier family 39 (zinc transporter). member 13
0.93	ZNRF3	zinc and ring finger 3
0.93	KCTD12	potassium channel tetramerization domain containing 12
0.93	ZBED1; DHRSX	zinc finger. BED-type containing 1; dehydrogenase/reductase (SDR family) X-linked
0.93	DIAPH1	diaphanous-related formin 1
0.93	SLC6A1	solute carrier family 6 (neurotransmitter transporter). member 1
0.93	OAS2	2-5-oligoadenylate synthetase 2
0.93	ARC	activity-regulated cytoskeleton-associated protein
0.93	SEMA4D	sema domain. immunoglobulin domain (Ig). transmembrane domain (TM) and short cytoplasmic domain. (semaphorin) 4D
0.93	AASDH	aminoadipate-semialdehyde dehydrogenase
0.93	TPRG1L	tumor protein p63 regulated 1-like
0.93	LINC01225	long intergenic non-protein coding RNA 1225
0.93	TMTC4	transmembrane and tetratricopeptide repeat containing 4
0.93	OR5P2	olfactory receptor. family 5. subfamily P. member 2
0.93	PROK2	prokineticin 2
0.93	LGALSL	lectin. galactoside-binding-like

0.93	IGHMBP2	immunoglobulin mu binding protein 2
0.93	VCX3A; VCX	variable charge. X-linked 3A; variable charge. X-linked
0.93	TPSAB1; TPSB2	tryptase alpha/beta 1; tryptase beta 2 (gene/pseudogene)
0.93	HMBS	hydroxymethylbilane synthase
0.93	TMCO6	transmembrane and coiled-coil domains 6
0.93	SNRPB2	small nuclear ribonucleoprotein polypeptide B
0.93	NIPBL	Nipped-B homolog (Drosophila)
0.93	CLEC1B	C-type lectin domain family 1. member B
0.93	UBE4B	ubiquitination factor E4B
0.93	CSPG4	chondroitin sulfate proteoglycan 4
0.93	GSG1	germ cell associated 1
0.93	ATP6V1B2	ATPase. H+ transporting. lysosomal 56/58kDa. V1 subunit B2
0.93	TLE4	transducin-like enhancer of split 4
0.93	LIPF	lipase. gastric
0.93	NUPR2	nuclear protein 2. transcriptional regulator
0.93	PDE4B	phosphodiesterase 4B. cAMP-specific
0.93	CPLX1	complexin 1
0.93	LHX9	LIM homeobox 9
0.93	COPB1	coatomer protein complex subunit beta 1
0.93	CYP4F8	cytochrome P450. family 4. subfamily F. polypeptide 8
0.93	TDRD7	tudor domain containing 7
0.93	PTPN22	protein tyrosine phosphatase. non-receptor type 22 (lymphoid)
0.93	PPP3CA	protein phosphatase 3. catalytic subunit. alpha isozyme
0.93	VHLL	von Hippel-Lindau tumor suppressor-like
0.93	SERINC4	serine incorporator 4
0.93	P2RY14	purinergic receptor P2Y. G-protein coupled. 14

0.93	PRL	prolactin
0.93	NEGR1	neuronal growth regulator 1
0.93	ECM1	extracellular matrix protein 1
0.93	TRIM71	tripartite motif containing 71. E3 ubiquitin protein ligase
0.93	HLTF	Transcript Identified by AceView. Entrez Gene ID(s) 6596
0.93	ZNF841	zinc finger protein 841
0.92	RABEP2	rabaptin. RAB GTPase binding effector protein 2
0.92	DYTN	dystrotelin
0.92	VN1R3	vomeronasal 1 receptor 3 (gene/pseudogene)
0.92	CNOT1; SNORA50A	CCR4-NOT transcription complex subunit 1; small nucleolar RNA. H/ACA box 50A
0.92	LOC388882; AP000345.1	uncharacterized LOC388882; novel transcript
0.92	U2AF2	U2 small nuclear RNA auxiliary factor 2
0.92	PLSCR2	phospholipid scramblase 2
0.92	TNFRSF1B; MIR4632; MIR7846	tumor necrosis factor receptor superfamily. member 1B; microRNA 4632; microRNA 7846
0.92	SPSB4	splA/ryanodine receptor domain and SOCS box containing 4
0.92	VCX; VCX3A	variable charge. X-linked; variable charge. X-linked 3A
0.92	C1orf87	chromosome 1 open reading frame 87
0.92	CALB1	calbindin 1
0.92	FAP	fibroblast activation protein alpha
0.92	BANP	BTG3 associated nuclear protein
0.92	LOC340074; CTC-321K16.4	uncharacterized LOC340074; Transcript Identified by AceView. Entrez Gene ID(s) 340074. RefSeq ID(s) NR_027127; novel transcript
0.92	MPZL3	myelin protein zero-like 3
0.92	WNT10B	wingless-type MMTV integration site family. member 10B
0.92	DPP6	dipeptidyl-peptidase 6

0.92	NOD1	nucleotide-binding oligomerization domain containing 1
0.92	RND3	Rho family GTPase 3
0.92	XXbac-BPG154L12.4; RNF5	putative novel transcript; Transcript Identified by AceView. Entrez Gene ID(s) 6048
0.92	PBX1	pre-B-cell leukemia homeobox 1
0.92	UBC	ubiquitin C
0.92	ATP6V0B	ATPase. H+ transporting. lysosomal 21kDa. V0 subunit b
0.92	AMT; NICN1	aminomethyltransferase; nicolin 1
0.92	REXO1L2P	REX1. RNA exonuclease 1 homolog-like 2. pseudogene
0.92	SSX9	synovial sarcoma. X breakpoint 9
0.92	GAREM2	GRB2 associated regulator of MAPK1 2
0.92	UEVLD	UEV and lactate/malate dehyrogenase domains
0.92	TMEM174	transmembrane protein 174
0.92	TRY2P	trypsinogen-like pseudogene
0.92	SRSF4	serine/arginine-rich splicing factor 4
0.92	ITGA5	integrin alpha 5
0.92	GSG1L	GSG1-like
0.92	SLC6A15	solute carrier family 6 (neutral amino acid transporter). member 15
0.92	LENG8	leukocyte receptor cluster (LRC) member 8
0.92	NHLRC4; PIGQ	NHL repeat containing 4; phosphatidylinositol glycan anchor biosynthesis class Q
0.92	SLC26A3	solute carrier family 26 (anion exchanger). member 3
0.92	BTG4	B-cell translocation gene 4
0.92	TRDN	triadin
0.92	TNRC18	trinucleotide repeat containing 18
0.92	POU5F1B	POU class 5 homeobox 1B
0.92	ME1	malic enzyme 1. NADP(+)-dependent. cytosolic

0.92	KLHL25	Memczak2013 ALT_ACCEPTOR. ALT_DONOR. coding. INTERNAL. intronic best transcript NM_022480
0.92	CD34	CD34 molecule
0.92	BTNL9	butyrophilin-like 9
0.92	GRIK3	glutamate receptor. ionotropic. kainate 3
0.92	OSM	oncostatin M
0.92	ZNF808; RPL39P34	zinc finger protein 808; ribosomal protein L39 pseudogene 34
0.92	C1QTNF8	C1q and tumor necrosis factor related protein 8
0.92	SLC35G5	solute carrier family 35. member G5
0.92	C1orf220	chromosome 1 open reading frame 220
0.92	RPS6KA1	ribosomal protein S6 kinase. 90kDa. polypeptide 1
0.92	LSP1; MIR7847	lymphocyte-specific protein 1; microRNA 7847
0.92	TMEM192	transmembrane protein 192
0.92	SMTN	smoothelin
0.92	SYNPO2L	synaptopodin 2-like
0.92	CSE1L	CSE1 chromosome segregation 1-like (yeast)
0.92	MYO15A	myosin XVA
0.92	ACTRT1	actin-related protein T1
0.92	IQCF2	IQ motif containing F2
0.92	MIR650; IGLV3-9; IGLV2-8	microRNA 650; immunoglobulin lambda variable 3-9 (gene/pseudogene); immunoglobulin lambda variable 2-8
0.92	PAQR7	progestin and adipoQ receptor family member VII
0.92	OR2T35	olfactory receptor. family 2. subfamily T. member 35
0.92	WEE2	WEE1 homolog 2 (S. pombe)
0.92	CBWD1	COBW domain containing 1
0.92	RPL23AP5	ribosomal protein L23a pseudogene 5
0.92	KDM5B	lysine (K)-specific demethylase 5B

0.92	CISD1	CDGSH iron sulfur domain 1
0.92	UBOX5; FASTKD5	U-box domain containing 5; FAST kinase domains 5
0.92	TGM7	transglutaminase 7
0.92	ZNF416	zinc finger protein 416
0.92	LINC00173	long intergenic non-protein coding RNA 173
0.92	PCDHB13	protocadherin beta 13
0.92	TMEM43	transmembrane protein 43
0.92	SPRR2E	small proline-rich protein 2E
0.92	CDH10	cadherin 10. type 2 (T2-cadherin)
0.92	COL9A1	collagen. type IX. alpha 1
0.92	DTD2	D-tyrosyl-tRNA deacylase 2 (putative)
0.92	LMNA	lamin A/C
0.92	CLEC9A	C-type lectin domain family 9. member A
0.92	HBQ1	hemoglobin. theta 1
0.92	USP42	ubiquitin specific peptidase 42
0.92	ADAM12	ADAM metallopeptidase domain 12
0.92	FCAMR	Fc receptor. IgA. IgM. high affinity
0.92	SLC22A10	solute carrier family 22. member 10
0.92	BCL2L10	BCL2-like 10 (apoptosis facilitator)
0.92	MRPL49	mitochondrial ribosomal protein L49
0.92	PHGDH	Memczak2013 ANTISENSE. CDS. coding. INTERNAL best transcript NM_006623
0.92	CAPN7	calpain 7
0.92	CCDC169-SOHLH2	CCDC169-SOHLH2 readthrough
0.92	TRAM2	Transcript Identified by AceView. Entrez Gene ID(s) 9697
0.92	CC2D1B	coiled-coil and C2 domain containing 1B

0.92	TOMM7	translocase of outer mitochondrial membrane 7 homolog (yeast)
0.92	TCHHL1	trichohyalin like 1
0.92	POTEC	POTE ankyrin domain family. member C
0.92	RNF112	ring finger protein 112
0.92	TMBIM6	transmembrane BAX inhibitor motif containing 6
0.92	USP22	ubiquitin specific peptidase 22
0.92	SPDYE16	speedy/RINGO cell cycle regulator family member E16 [Source:HGNC Symbol;Acc:HGNC:51512]
0.92	COA6	cytochrome c oxidase assembly factor 6
0.92	PUM1	pumilio RNA binding family member 1
0.92	NELL2	neural EGFL like 2
0.92	ASTN1	astrotactin 1
0.92	ATL1	atlastin GTPase 1
0.92	PPIP5K2	diphosphoinositol pentakisphosphate kinase 2
0.92	FHOD3	formin homology 2 domain containing 3
0.92	PBOV1	prostate and breast cancer overexpressed 1
0.92	SUMF2	sulfatase modifying factor 2
0.92	COLCA2	colorectal cancer associated 2
0.92	ZMYM3	zinc finger. MYM-type 3
0.92	ELAVL1	ELAV like RNA binding protein 1
0.92	HMX1	H6 family homeobox 1
0.92	TMEM70; RPS20P21	transmembrane protein 70; ribosomal protein S20 pseudogene 21
0.92	TMEM88B	transmembrane protein 88B
0.92	SMC1A	structural maintenance of chromosomes 1A
0.92	BOLA1	bolA family member 1
0.92	HTN3	histatin 3

0.92	CILP2	cartilage intermediate layer protein 2
0.92	POLR3A	polymerase (RNA) III (DNA directed) polypeptide A. 155kDa
0.92	ATP13A3	Transcript Identified by AceView. Entrez Gene ID(s) 79572
0.92	TRIM47	tripartite motif containing 47
0.92	CKAP5; SNORD67	cytoskeleton associated protein 5; small nucleolar RNA. C/D box 67
0.92	ITGAM	integrin. alpha M (complement component 3 receptor 3 subunit)
0.92	ABCA3	ATP binding cassette subfamily A member 3
0.92	PLA2G2C	phospholipase A2. group IIC
0.92	KLK11	kallikrein related peptidase 11
0.92	SLC34A2	solute carrier family 34 (type II sodium/phosphate cotransporter). member 2
0.92	VPS18	VPS18 CORVET/HOPS core subunit
0.92	JAKMIP2	janus kinase and microtubule interacting protein 2
0.92	C9orf139	chromosome 9 open reading frame 139
0.92	ZNF157	zinc finger protein 157
0.92	GBP6	guanylate binding protein family. member 6
0.92	NEUROD1	neuronal differentiation 1
0.92	HNRNPH1	heterogeneous nuclear ribonucleoprotein H1 (H)
0.92	EMILIN3	elastin microfibril interfacer 3
0.92	KCNIP1	Kv channel interacting protein 1
0.92	KRTAP9-4	keratin associated protein 9-4
0.92	KRCC1	lysine-rich coiled-coil 1
0.92	ABCD1	Transcript Identified by AceView. Entrez Gene ID(s) 215
0.92	CTSO	cathepsin O
0.92	BTBD2	BTB (POZ) domain containing 2
0.92	ADGRE1	adhesion G protein-coupled receptor E1
0.92	EMB	embigin

0.92	FOXO6	forkhead box O6
0.92	RDH13	retinol dehydrogenase 13 (all-trans/9-cis)
0.92	PIN1	peptidylprolyl cis/trans isomerase. NIMA-interacting 1
0.92	EPHB3	EPH receptor B3
0.92	GJA8	gap junction protein alpha 8
0.92	CST8	cystatin 8 (cystatin-related epididymal specific)
0.92	SETX	senataxin
0.92	BOK	BCL2-related ovarian killer
0.92	APRT	adenine phosphoribosyltransferase
0.92	PCIF1	PDX1 C-terminal inhibiting factor 1
0.92	ZC3H12D	zinc finger CCCH-type containing 12D
0.92	TBC1D3B; TBC1D3H; TBC1D3I; TBC1D3F; TBC1D3G	TBC1 domain family. member 3B; TBC1 domain family. member 3H; TBC1 domain family. member 3I; TBC1 domain family. member 3F; TBC1 domain family. member 3G
0.92	HLA-DPB1	major histocompatibility complex. class II. DP beta 1
0.92	RP11-351M8.2; MESDC2; RP11-351M8.1	Transcript Identified by AceView. Entrez Gene ID(s) 23184; novel transcript. antisense to CEMIP
0.92	C9orf66	chromosome 9 open reading frame 66
0.92	TIMM8A	translocase of inner mitochondrial membrane 8 homolog A (yeast)
0.92	VPS37D	vacuolar protein sorting 37 homolog D (S. cerevisiae)
0.92	PRSS48	protease. serine. 48
0.92	ZHX1-C8orf76	ZHX1-C8orf76 readthrough
0.92	TAC3	tachykinin 3
0.92	PARP16	poly(ADP-ribose) polymerase family member 16
0.92	HMGA2	high mobility group AT-hook 2
0.92	ALPK2	alpha kinase 2
0.92	EIF2B1	eukaryotic translation initiation factor 2B. subunit 1 alpha. 26kDa

0.92	LAYN	layilin
0.92	ZNF135	zinc finger protein 135
0.92	NEMP1	nuclear envelope integral membrane protein 1
0.92	FAM177A1	family with sequence similarity 177. member A1
0.92	FICD	FIC domain containing
0.92	NTF4	neurotrophin 4
0.92	SLC38A8	Transcript Identified by AceView. Entrez Gene ID(s) 146167
0.92	C9orf64	Transcript Identified by AceView. Entrez Gene ID(s) 84267
0.92	CDK18	cyclin-dependent kinase 18
0.92	OR2L2	olfactory receptor. family 2. subfamily L. member 2
0.92	C10orf11	chromosome 10 open reading frame 11
0.92	LILRB4	leukocyte immunoglobulin-like receptor. subfamily B (with TM and ITIM domains). member 4
0.92	CCDC144A	coiled-coil domain containing 144A
0.92	CACNG4	calcium channel. voltage-dependent. gamma subunit 4
0.92	GLYATL1	glycine-N-acyltransferase-like 1
0.92	LOC100288748; RP11-22C11.2	uncharacterized LOC100288748; novel transcript. antisense to ESRP1
0.92	RCSD1	RCSD domain containing 1
0.92	PODN	podocan
0.92	RPS6KA1	ribosomal protein S6 kinase. 90kDa. polypeptide 1
0.92	SPHKAP	SPHK1 interactor. AKAP domain containing
0.92	NIPAL4	NIPA-like domain containing 4
0.92	LINC00174	long intergenic non-protein coding RNA 174
0.92	C10orf55	chromosome 10 open reading frame 55
0.92	OR7C1	olfactory receptor. family 7. subfamily C. member 1
0.92	ANXA10	annexin A10

0.92	CLEC4F	C-type lectin domain family 4. member F
0.92	RMND1	required for meiotic nuclear division 1 homolog
0.92	NBPF11	neuroblastoma breakpoint family. member 11
0.92	DEPDC1B	DEP domain containing 1B
0.92	PLA2G4A	phospholipase A2. group IVA (cytosolic. calcium-dependent)
0.92	PRAMEF6	PRAME family member 6 [Source:HGNC Symbol;Acc:HGNC:30583]
0.92	EN1	engrailed homeobox 1
0.92	RING1	ring finger protein 1
0.92	RAB24	RAB24. member RAS oncogene family
0.92	MIEN1	migration and invasion enhancer 1
0.92	ARFIP1	ADP-ribosylation factor interacting protein 1
0.92	FSTL1; MIR198	follistatin like 1; microRNA 198
0.92	BLM	Bloom syndrome. RecQ helicase-like
0.92	TMEM205	transmembrane protein 205
0.92	PSMC4	proteasome 26S subunit. ATPase 4 [Source:HGNC Symbol;Acc:HGNC:9551]
0.92	WDR6	Memczak2013 ANTISENSE. CDS. coding. INTERNAL best transcript NM_018031
0.92	TPTE	transmembrane phosphatase with tensin homology
0.92	PGBD2	piggyBac transposable element derived 2
0.92	ERGIC3	ERGIC and golgi 3
0.92	BBS4	Memczak2013 ALT_ACCEPTOR. ALT_DONOR. coding. INTERNAL. intronic best transcript NM_033028
0.92	PNMA3	paraneoplastic Ma antigen 3
0.92	P2RX3	purinergic receptor P2X. ligand gated ion channel. 3
0.92	DMC1	DNA meiotic recombinase 1
0.92	PHGR1	proline/histidine/glycine-rich 1
0.92	REXO1L1P; REXO1L3P	REX1. RNA exonuclease 1 homolog-like 1. pseudogene; REX1. RNA

		exonuclease 1 homolog-like 3. pseudogene
0.92	LOC100506422; RP11-80I3.1; tayamu	putative deoxyuridine 5-triphosphate nucleotidohydrolase-like protein FLJ16323; novel transcript; Transcript Identified by AceView
0.92	MBD5	methyl-CpG binding domain protein 5
0.92	ZNF559-ZNF177; ZNF177; ZNF559	ZNF559-ZNF177 readthrough; zinc finger protein 177; zinc finger protein 559
0.92	SEC23A	Sec23 homolog A. COPII coat complex component
0.92	DDX25	DEAD (Asp-Glu-Ala-Asp) box helicase 25
0.92	ALMS1	Alstrom syndrome protein 1
0.92	ESRRG	estrogen-related receptor gamma
0.92	CACTIN	cactin. spliceosome C complex subunit
0.92	CYP2D6	cytochrome P450. family 2. subfamily D. polypeptide 6
0.92	PCBD1	pterin-4 alpha-carbinolamine dehydratase/dimerization cofactor of hepatocyte nuclear factor 1 alpha
0.92	ACR	acrosin
0.92	OLFM4	olfactomedin 4
0.92	GABRB1	gamma-aminobutyric acid (GABA) A receptor. beta 1
0.92	RGS13	regulator of G-protein signaling 13
0.92	TCTA	T-cell leukemia translocation altered
0.92	C19orf84	chromosome 19 open reading frame 84
0.92	PTPRT	protein tyrosine phosphatase. receptor type. T
0.92	PLCG1; RPL23AP81	phospholipase C. gamma 1; ribosomal protein L23a pseudogene 81
0.92	MAGEH1	MAGE family member H1
0.92	AHCYL1	adenosylhomocysteinase like 1
0.92	KIN	Kin17 DNA and RNA binding protein
0.92	OR14I1	olfactory receptor. family 14. subfamily I. member 1
0.92	SCEL	sciellin

0.92	CYB5R4	cytochrome b5 reductase 4
0.92	ARPP21	Transcript Identified by AceView. Entrez Gene ID(s) 10777
0.92	RARRES3	retinoic acid receptor responder (tazarotene induced) 3
0.92	FGFR4	fibroblast growth factor receptor 4
0.92	BCKDHA	branched chain keto acid dehydrogenase E1. alpha polypeptide
0.92	NKX6-3	NK6 homeobox 3
0.92	TFB2M	transcription factor B2. mitochondrial
0.92	ANOS1	anosmin 1
0.92	TBC1D3E	TBC1 domain family. member 3E
0.92	CDCP2	CUB domain containing protein 2
0.92	C5orf38	chromosome 5 open reading frame 38
0.92	KLHDC4	kelch domain containing 4
0.92	PSAPL1	prosaposin-like 1 (gene/pseudogene)
0.92	OR1E3	olfactory receptor. family 1. subfamily E. member 3 (gene/pseudogene)
0.92	TM4SF18	transmembrane 4 L six family member 18
0.92	SKIDA1	SKI/DACH domain containing 1
0.92	HIST1H3I	histone cluster 1. H3i
0.92	CCL14; CCL15; CCL15-CCL14	chemokine (C-C motif) ligand 14; chemokine (C-C motif) ligand 15; CCL15-CCL14 readthrough (NMD candidate)
0.92	LRIG1	
0.92	HIST1H2BA	histone cluster 1. H2ba
0.92	HTRA3	HtrA serine peptidase 3
0.92	TAS2R9	taste receptor. type 2. member 9
0.92	KAT7	K(lysine) acetyltransferase 7
0.92	SLC26A7	solute carrier family 26 (anion exchanger). member 7
0.92	LGALS9	lectin. galactoside-binding. soluble. 9

0.92	OR2A7; ARHGEF34P	olfactory receptor. family 2. subfamily A. member 7; Rho guanine nucleotide exchange factor 34. pseudogene
0.92	ZFPM2	zinc finger protein. FOG family member 2
0.92	LINC00525	long intergenic non-protein coding RNA 525
0.92	OMA1	OMA1 zinc metallopeptidase
0.92	CNTNAP4	contactin associated protein-like 4
0.92	OPN1MW; OPN1MW2; OPN1MW3	opsin 1 (cone pigments). medium-wave-sensitive; opsin 1 (cone pigments). medium-wave-sensitive 2; opsin 1 (cone pigments). medium-wave-sensitive 3
0.92	MED4	mediator complex subunit 4
0.92	ELFN2	extracellular leucine-rich repeat and fibronectin type III domain containing 2
0.92	PGM1	phosphoglucomutase 1
0.92	TAS1R3	taste receptor. type 1. member 3
0.92	KCNMB4	potassium channel subfamily M regulatory beta subunit 4
0.92	COLEC10	collectin sub-family member 10 (C-type lectin)
0.92	TUBB4A	tubulin. beta 4A class IVa
0.92	POLR2F; MIR4534; MIR6820	polymerase (RNA) II (DNA directed) polypeptide F; microRNA 4534; microRNA 6820
0.92	PAM	peptidylglycine alpha-amidating monooxygenase
0.92	SPECC1L	sperm antigen with calponin homology and coiled-coil domains 1-like
0.92	COL11A2	collagen. type XI. alpha 2
0.92	RPS6KA1	ribosomal protein S6 kinase. 90kDa. polypeptide 1
0.92	SORBS2	Transcript Identified by AceView. Entrez Gene ID(s) 8470
0.92	KLK9	kallikrein related peptidase 9
0.92	WNT16	wingless-type MMTV integration site family. member 16
0.92	XAF1	XIAP associated factor 1
0.92	SHD	Src homology 2 domain containing transforming protein D

0.92	ANKRD55	ankyrin repeat domain 55
0.92	AGMAT	agmatinase
0.92	GABRR2	gamma-aminobutyric acid (GABA) A receptor. rho 2
0.92	TIAL1	Transcript Identified by AceView. Entrez Gene ID(s) 7073
0.92	ILVBL	ilvB (bacterial acetolactate synthase)-like
0.92	TTLL8	tubulin tyrosine ligase-like family member 8
0.92	TAS2R41	taste receptor. type 2. member 41
0.92	KLRC1	killer cell lectin-like receptor subfamily C. member 1
0.92	SPIDR	scaffolding protein involved in DNA repair
0.92	CD69	CD69 molecule
0.92	SNX17	sorting nexin 17
0.92	MED12L	mediator complex subunit 12 like
0.92	AGAP6	ArfGAP with GTPase domain. ankyrin repeat and PH domain 6
0.92	ASB17	ankyrin repeat and SOCS box containing 17
0.92	ALDH1L1	aldehyde dehydrogenase 1 family. member L1
0.92	VWA3A	von Willebrand factor A domain containing 3A
0.92	COX15	cytochrome c oxidase assembly homolog 15 (yeast)
0.92	HDAC2	histone deacetylase 2
0.92	THOC1	THO complex 1
0.92	OR1J2	olfactory receptor. family 1. subfamily J. member 2
0.92	KIRREL	kin of IRRE like (Drosophila)
0.92	CSAG1	chondrosarcoma associated gene 1
0.92	ANO6	Transcript Identified by AceView. Entrez Gene ID(s) 196527
0.92	CHRNA4	cholinergic receptor. nicotinic alpha 4
0.92	ERLEC1	endoplasmic reticulum lectin 1
0.92	SIX2	SIX homeobox 2

0.92	DNAH9	dynein. axonemal. heavy chain 9
0.92	MYBPC2	myosin binding protein C. fast type
0.92	TTC1	Transcript Identified by AceView. Entrez Gene ID(s) 7265
0.92	MSGN1	mesogenin 1
0.92	HYLS1	hydrolethalus syndrome 1
0.92	ZNF398	zinc finger protein 398
0.92	AKAP11	A kinase (PRKA) anchor protein 11
0.92	NBEAL1	neurobeachin like 1
0.92	A2M	alpha-2-macroglobulin
0.92	ZNF154	zinc finger protein 154
0.92	GPX5	glutathione peroxidase 5
0.92	CCK	cholecystokinin
0.92	EPX	eosinophil peroxidase
0.92	H6PD	hexose-6-phosphate dehydrogenase (glucose 1-dehydrogenase)
0.92	NELL1	neural EGFL like 1
0.92	WNT9B	wingless-type MMTV integration site family. member 9B
0.92	KRT5	keratin 5. type II
0.92	LCE2C	late cornified envelope 2C
0.92	MS4A4E	membrane-spanning 4-domains. subfamily A. member 4E
0.92	KCNT2	potassium channel. sodium activated subfamily T. member 2
0.92	NOXRED1	NADP-dependent oxidoreductase domain containing 1
0.92	PARP15	poly(ADP-ribose) polymerase family member 15
0.92	MAD1L1	MAD1 mitotic arrest deficient-like 1 (yeast)
0.92	ITGB8	integrin beta 8
0.92	USP24	Transcript Identified by AceView. Entrez Gene ID(s) 23358
0.92	EFHB	EF-hand domain family. member B

0.92	HNRNPM	heterogeneous nuclear ribonucleoprotein M
0.92	FAM157A	family with sequence similarity 157. member A
0.92	MS4A14	membrane-spanning 4-domains. subfamily A. member 14
0.92	ZNF610	zinc finger protein 610
0.92	FAM134A	family with sequence similarity 134. member A
0.92	FBF1	Fas (TNFRSF6) binding factor 1
0.92	GCC2	Jeck2013 ANTISENSE. CDS. coding. INTERNAL. intronic. OVCODE. OVEXON best transcript NM_181453
0.92	DDX42	DEAD (Asp-Glu-Ala-Asp) box helicase 42
0.92	CISD2	CDGSH iron sulfur domain 2
0.92	TEX13B	testis expressed 13B
0.92	EVA1C	eva-1 homolog C (C. elegans)
0.92	SLFN12L	schlafen family member 12-like
0.92	ARPC3	actin related protein 2/3 complex subunit 3
0.92	NUFIP2; rerdy	Memczak2013 ALT_ACCEPTOR. ALT_DONOR. coding. INTERNAL. intronic best transcript NM_020772; Transcript Identified by AceView
0.92	ZNF655	zinc finger protein 655
0.92	ZNF267	zinc finger protein 267
0.92	CHURC1	churchill domain containing 1
0.92	SAMD12	sterile alpha motif domain containing 12
0.92	NDUFA6	NADH dehydrogenase (ubiquinone) 1 alpha subcomplex. 6. 14kDa
0.92	SULT4A1	sulfotransferase family 4A member 1
0.92	MRPL22	mitochondrial ribosomal protein L22
0.92	HHIPL1	HHIP-like 1
0.92	SPN	Memczak2013 ANTISENSE. CDS. coding. INTERNAL best transcript NM_003123
0.92	CHMP3	charged multivesicular body protein 3

0.92	ARHGAP39	Rho GTPase activating protein 39
0.92	MARCKS	myristoylated alanine-rich protein kinase C substrate
0.92	PDE4DIP	Transcript Identified by AceView. Entrez Gene ID(s) 9659
0.92	TRPM1	transient receptor potential cation channel. subfamily M. member 1
0.92	LINC01599	long intergenic non-protein coding RNA 1599
0.92	RPL7A	ribosomal protein L7a
0.92	APELA	apelin receptor early endogenous ligand
0.92	SYDE1	synapse defective 1. Rho GTPase. homolog 1 (C. elegans)
0.92	CTXN1	cortexin 1
0.92	SCN8A	Jeck2013 ANTISENSE. coding. INTERNAL. intronic best transcript NM_014191
0.92	AGPAT4	Transcript Identified by AceView. Entrez Gene ID(s) 56895
0.92	KCTD17	potassium channel tetramerization domain containing 17
0.92	BPIFA2	BPI fold containing family A. member 2
0.92	FAM129C	family with sequence similarity 129. member C
0.92	SPPL2B	signal peptide peptidase like 2B
0.92	IRS2	Transcript Identified by AceView. Entrez Gene ID(s) 8660
0.92	CNTN2	contactin 2 (axonal)
0.92	CBWD5	COBW domain containing 5
0.92	C4orf19	chromosome 4 open reading frame 19
0.92	ADGRD1	adhesion G protein-coupled receptor D1
0.92	YWHAE	tyrosine 3-monooxygenase/tryptophan 5-monooxygenase activation protein. epsilon
0.92	CDS1	CDP-diacylglycerol synthase 1
0.92	KRTAP2-4	keratin associated protein 2-4
0.92	WDR38	WD repeat domain 38
0.92	KLHL4	kelch-like family member 4

0.92	LCE1E	late cornified envelope 1E
0.92	LIMD2	LIM domain containing 2
0.92	GIPR	gastric inhibitory polypeptide receptor
0.92	SEMA4F	sema domain. immunoglobulin domain (Ig). transmembrane domain (TM) and short cytoplasmic domain. (semaphorin) 4F
0.92	MS4A5	membrane-spanning 4-domains. subfamily A. member 5
0.92	PRAMEF8	PRAME family member 8
0.92	CDPF1	cysteine rich. DPF motif domain containing 1
0.92	ZNF503-AS2	ZNF503 antisense RNA 2
0.92	KCNF1	potassium channel. voltage gated modifier subfamily F. member 1
0.92	FAM102B	family with sequence similarity 102. member B
0.92	LRRC7	leucine rich repeat containing 7
0.92	HMG20B	high mobility group 20B
0.92	ZNF358	zinc finger protein 358
0.92	CXCL3	chemokine (C-X-C motif) ligand 3
0.92	CBLN1	cerebellin 1 precursor
0.92	DENND5B	DENN/MADD domain containing 5B
0.92	PDZD11	PDZ domain containing 11
0.92	SAG	S-antigen; retina and pineal gland (arrestin)
0.92	ISM2	isthmin 2
0.92	GNAT3	guanine nucleotide binding protein. alpha transducing 3
0.92	PURA	purine-rich element binding protein A
0.92	PPP1R10	protein phosphatase 1. regulatory subunit 10
0.92	TMEM196	transmembrane protein 196
0.92	REG3G	regenerating islet-derived 3 gamma
0.92	C7orf49	chromosome 7 open reading frame 49

0.92	ADCY2	adenylate cyclase 2 (brain)
0.92	ADGRF1	adhesion G protein-coupled receptor F1
0.92	EPHA10	EPH receptor A10
0.92	C18orf32	chromosome 18 open reading frame 32
0.92	UNC119B	Memczak2013 ANTISENSE. CDS. coding. INTERNAL. UTR3 best transcript NM_001080533
0.92	DET1	de-etiolated homolog 1 (Arabidopsis)
0.92	sept-02	septin 2
0.92	ZFHX2	zinc finger homeobox 2
0.92	CD164L2	CD164 sialomucin-like 2
0.92	DNAJB9	DnaJ (Hsp40) homolog. subfamily B. member 9
0.92	CABP2	calcium binding protein 2
0.92	TMPRSS5	transmembrane protease. serine 5
0.92	DCAF12L2	DDB1 and CUL4 associated factor 12-like 2
0.92	RNASE12; RNASE11	ribonuclease. RNase A family. 12 (non-active); ribonuclease. RNase A family. 11 (non-active)
0.92	AMZ2	archaelysin family metallopeptidase 2
0.92	ZNF384	zinc finger protein 384
0.92	C9orf163	chromosome 9 open reading frame 163
0.92	GIMAP8	GTPase. IMAP family member 8
0.92	PRIM2	Salzman2013 ANNOTATED. CDS. coding. OVCODE. OVEXON. UTR3 best transcript NM_000947
0.92	SRPK3	SRSF protein kinase 3
0.92	SPINT2	serine peptidase inhibitor. Kunitz type. 2
0.92	MANSC4	MANSC domain containing 4
0.92	NDFIP2	Nedd4 family interacting protein 2
0.92	APPL2	adaptor protein. phosphotyrosine interaction. PH domain and leucine zipper

		containing 2
0.92	EPB41L4A	erythrocyte membrane protein band 4.1 like 4A
0.92	TMEM263	transmembrane protein 263
0.92	MRPS18A	mitochondrial ribosomal protein S18A
0.92	CACNG2	calcium channel. voltage-dependent. gamma subunit 2
0.92	CHPT1	choline phosphotransferase 1
0.92	SIRPA	signal-regulatory protein alpha
0.92	C5orf22	chromosome 5 open reading frame 22
0.92	HLA-DQA2	major histocompatibility complex. class II. DQ alpha 2
0.92	GALNT1	polypeptide N-acetylgalactosaminyltransferase 1
0.92	XYLT2	xylosyltransferase II
0.92	POU5F1	POU class 5 homeobox 1
0.92	RNH1	ribonuclease/angiogenin inhibitor 1
0.92	PPA2	pyrophosphatase (inorganic) 2
0.92	KLLN	killin. p53-regulated DNA replication inhibitor
0.92	BNIP3L	BCL2/adenovirus E1B 19kDa interacting protein 3-like
0.92	NBPF19	neuroblastoma breakpoint family. member 19
0.92	RAB7A	RAB7A. member RAS oncogene family
0.92	AC007292.7; MPND	Transcript Identified by AceView. Entrez Gene ID(s) 84954; novel transcript. antisense to STAP2
0.92	SENP7	SUMO1/sentrin specific peptidase 7
0.92	DMRTC2	DMRT-like family C2
0.92	TRAPPC4	trafficking protein particle complex 4
0.92	TBC1D1	TBC1 (tre-2/USP6. BUB2. cdc16) domain family. member 1
0.92	SHISA3	shisa family member 3
0.92	CLMP	CXADR-like membrane protein

0.92	TRABD2B	TraB domain containing 2B
0.92	INIP	INTS3 and NABP interacting protein
0.92	SAXO2	stabilizer of axonemal microtubules 2
0.92	PLD1	phospholipase D1. phosphatidylcholine-specific
0.92	ATF7; NPFF	activating transcription factor 7; neuropeptide FF-amide peptide precursor
0.92	PVRL2	poliovirus receptor-related 2 (herpesvirus entry mediator B)
0.92	MSL2	male-specific lethal 2 homolog (Drosophila)
0.92	LOC643802	u3 small nucleolar ribonucleoprotein protein MPP10-like
0.92	PPP1R3C	protein phosphatase 1. regulatory subunit 3C
0.92	ZFR2	zinc finger RNA binding protein 2
0.92	ATP8B2	ATPase. aminophospholipid transporter. class I. type 8B. member 2
0.92	EPB41L2	erythrocyte membrane protein band 4.1-like 2
0.92	ZNF646	zinc finger protein 646
0.92	SPEG	SPEG complex locus
0.92	COASY	CoA synthase
0.92	ZNF699	zinc finger protein 699
0.92	GNL1	guanine nucleotide binding protein-like 1
0.92	NLRX1	NLR family member X1
0.92	HOXA3	homeobox A3
0.92	OR5B17	olfactory receptor. family 5. subfamily B. member 17
0.92	PSMC2	proteasome 26S subunit. ATPase 2
0.92	CNKSR2	connector enhancer of kinase suppressor of Ras 2
0.92	IMP3	IMP3. U3 small nucleolar ribonucleoprotein
0.92	NOC2L	NOC2-like nucleolar associated transcriptional repressor
0.91	FAM234B	family with sequence similarity 234. member B
0.91	MMP11	matrix metallopeptidase 11

0.91	HES2	hes family bHLH transcription factor 2
0.91	ALOX5	Transcript Identified by AceView. Entrez Gene ID(s) 240
0.91	APBB3; MIR6831	amyloid beta (A4) precursor protein-binding. family B. member 3; microRNA 6831
0.91	ZNF397	zinc finger protein 397
0.91	ZNF19	zinc finger protein 19
0.91	CX3CL1	chemokine (C-X3-C motif) ligand 1
0.91	PSTPIP1	proline-serine-threonine phosphatase interacting protein 1
0.91	POP5	POP5 homolog. ribonuclease P/MRP subunit
0.91	NLRP10	NLR family. pyrin domain containing 10
0.91	BIN3-IT1	BIN3 intronic transcript 1
0.91	MIF4GD	MIF4G domain containing
0.91	ISCA2	iron-sulfur cluster assembly 2
0.91	SHKBP1	SH3KBP1 binding protein 1
0.91	NRL	neural retina leucine zipper
0.91	YEATS2	Memczak2013 ANTISENSE. coding. INTERNAL. intronic best transcript NM_018023
0.91	CCDC173	coiled-coil domain containing 173
0.91	ADGRG7	adhesion G protein-coupled receptor G7
0.91	DPYS	dihydropyrimidinase
0.91	PTK2	Transcript Identified by AceView. Entrez Gene ID(s) 5747
0.91	FOXN4	forkhead box N4
0.91	SERPINB5	serpin peptidase inhibitor. clade B (ovalbumin). member 5
0.91	COMMD5	COMM domain containing 5
0.91	POM121L12	POM121 transmembrane nucleoporin-like 12
0.91	TMED7-TICAM2; TICAM2; TMED7	TMED7-TICAM2 readthrough; toll-like receptor adaptor molecule 2; transmembrane p24 trafficking protein 7

0.91	PRSS38	protease. serine 38
0.91	CCDC159	coiled-coil domain containing 159
0.91	STXBP1	syntaxin binding protein 1
0.91	FAM73B	family with sequence similarity 73. member B
0.91	SUB1	SUB1 homolog. transcriptional regulator
0.91	SARNP	SAP domain containing ribonucleoprotein
0.91	NOP9	NOP9 nucleolar protein
0.91	RGAG1	retrotransposon gag domain containing 1
0.91	MTFMT	mitochondrial methionyl-tRNA formyltransferase
0.91	TRNAU1AP	tRNA selenocysteine 1 associated protein 1
0.91	PEAK1	pseudopodium-enriched atypical kinase 1
0.91	PTTG1IP	pituitary tumor-transforming 1 interacting protein
0.91	FSIP2	fibrous sheath interacting protein 2
0.91	MAGOHB	mago homolog B. exon junction complex core component
0.91	RPP25	ribonuclease P/MRP 25kDa subunit
0.91	MED29	mediator complex subunit 29
0.91	MLXIP	Memczak2013 ALT_ACCEPTOR. ALT_DONOR. coding. INTERNAL. intronic best transcript NM_014938
0.91	TRIM10	tripartite motif containing 10
0.91	DMRT3	doublesex and mab-3 related transcription factor 3
0.91	MAP1LC3A	microtubule-associated protein 1 light chain 3 alpha
0.91	TSHZ1	teashirt zinc finger homeobox 1
0.91	ACSL6	acyl-CoA synthetase long-chain family member 6
0.91	THBS3	Salzman2013 ALT_ACCEPTOR. ALT_DONOR. coding. INTERNAL. intronic best transcript NM_007112
0.91	ZNF467	zinc finger protein 467
0.91	RAB7B	RAB7B. member RAS oncogene family

0.91	BTBD16	BTB (POZ) domain containing 16
0.91	PYROXD1	pyridine nucleotide-disulphide oxidoreductase domain 1
0.91	RHOA	ras homolog family member A
0.91	ZNRF1	zinc and ring finger 1. E3 ubiquitin protein ligase
0.91	IRF2BP1	interferon regulatory factor 2 binding protein 1
0.91	HDLBP	high density lipoprotein binding protein
0.91	ZNF197	zinc finger protein 197
0.91	DDI1	DNA-damage inducible 1 homolog 1
0.91	AMTN	amelotin
0.91	ATP8B1	Salzman2013 ANTISENSE. CDS. coding. INTERNAL. intronic. OVCODE. OVERLAPTX. OVEXON. UTR5 best transcript NM_005603
0.91	ABCD4	ATP binding cassette subfamily D member 4
0.91	ZFP92	ZFP92 zinc finger protein
0.91	CYP2F1	cytochrome P450. family 2. subfamily F. polypeptide 1
0.91	SMC6	Transcript Identified by AceView. Entrez Gene ID(s) 79677
0.91	FBXO34	F-box protein 34
0.91	TMX2-CTNND1	TMX2-CTNND1 readthrough (NMD candidate)
0.91	GNA12	guanine nucleotide binding protein (G protein) alpha 12
0.91	NEUROG3	neurogenin 3
0.91	POR; MIR4651	P450 (cytochrome) oxidoreductase; microRNA 4651
0.91	SETD4	SET domain containing 4
0.91	BASP1	brain abundant. membrane attached signal protein 1
0.91	CCDC84	coiled-coil domain containing 84
0.91	KIAA1143	KIAA1143 [Source:HGNC Symbol;Acc:HGNC:29198]
0.91	BLACE	B-cell acute lymphoblastic leukemia expressed
0.91	KRTAP10-3	keratin associated protein 10-3

0.91	C1QTNF7	C1q and tumor necrosis factor related protein 7
0.91	CXCL6	chemokine (C-X-C motif) ligand 6
0.91	KRT84	keratin 84. type II
0.91	MND1	meiotic nuclear divisions 1
0.91	SAG	S-antigen; retina and pineal gland (arrestin)
0.91	TMPRSS11F	transmembrane protease. serine 11F
0.91	CYP2D6	cytochrome P450. family 2. subfamily D. polypeptide 6
0.91	OR2V1	olfactory receptor. family 2. subfamily V. member 1
0.91	ELP6	elongator acetyltransferase complex subunit 6
0.91	SEC31A	SEC31 homolog A. COPII coat complex component
0.91	CDY2B; CDY2A	chromodomain protein. Y-linked. 2B; chromodomain protein. Y-linked. 2A
0.91	STOML1	stomatin (EPB72)-like 1
0.91	SERPINA9	serpin peptidase inhibitor. clade A (alpha-1 antiproteinase. antitrypsin). member 9
0.91	ECI2	enoyl-CoA delta isomerase 2
0.91	ADAMTS5	ADAM metallopeptidase with thrombospondin type 1 motif 5
0.91	ZNF433	zinc finger protein 433
0.91	RNF150	ring finger protein 150
0.91	BTNL8	butyrophilin-like 8
0.91	VWA3B	von Willebrand factor A domain containing 3B
0.91	NT5DC4	5-nucleotidase domain containing 4
0.91	LGALS4	lectin. galactoside-binding. soluble. 4
0.91	SPIN4	spindlin family. member 4
0.91	FAM9A	family with sequence similarity 9. member A
0.91	MAGEF1	MAGE family member F1
0.91	PTAR1	protein prenyltransferase alpha subunit repeat containing 1

0.91	ANO7	anoctamin 7
0.91	FGL2	fibrinogen-like 2
0.91	CCDC106	coiled-coil domain containing 106
0.91	PIK3C2G	phosphatidylinositol-4-phosphate 3-kinase. catalytic subunit type 2 gamma
0.91	MRPS18B	mitochondrial ribosomal protein S18B
0.91	SFRP1	secreted frizzled-related protein 1
0.91	MED10	mediator complex subunit 10
0.91	PRKD1	protein kinase D1
0.91	PRDM7	PR domain containing 7
0.91	GOLT1B	golgi transport 1B
0.91	TNFRSF9	tumor necrosis factor receptor superfamily. member 9
0.91	TSPYL6	TSPY-like 6
0.91	PCGF3	polycomb group ring finger 3
0.91	SYNGR4	synaptogyrin 4
0.91	CCDC166	coiled-coil domain containing 166
0.91	LGR5	leucine-rich repeat containing G protein-coupled receptor 5
0.91	FADS2	fatty acid desaturase 2
0.91	THAP10	THAP domain containing 10
0.91	FOXL1	forkhead box L1
0.91	PILRB	Jeck2013 ANTISENSE. INTERNAL. intronic. ncRNA. OVEXON best transcript NR_036569
0.91	CLCNKA	chloride channel. voltage-sensitive Ka
0.91	PNPLA7	patatin-like phospholipase domain containing 7
0.91	HMBS	hydroxymethylbilane synthase
0.91	MYO9A	Memczak2013 ANTISENSE. CDS. coding. INTERNAL best transcript NM_006901
0.91	ZNF182	zinc finger protein 182

0.91	PJA2	praja ring finger 2. E3 ubiquitin protein ligase
0.91	KRIT1	KRIT1. ankyrin repeat containing
0.91	AFP	alpha-fetoprotein
0.91	SIX1	SIX homeobox 1
0.91	HEPN1	hepatocellular carcinoma. down-regulated 1
0.91	BEGAIN	brain-enriched guanylate kinase-associated
0.91	ATP4A	ATPase. H+/K+ exchanging. alpha polypeptide
0.91	HHAT	hedgehog acyltransferase
0.91	OCLM	oculomedin
0.91	YWHAB	tyrosine 3-monooxygenase/tryptophan 5-monooxygenase activation protein. beta
0.91	JMJD8	jumonji domain containing 8
0.91	OPN1MW	opsin 1 (cone pigments). medium-wave-sensitive
0.91	FCHO1	FCH domain only 1
0.91	KCNS1	potassium voltage-gated channel. modifier subfamily S. member 1
0.91	PAPL	iron/zinc purple acid phosphatase-like protein; Iron/zinc purple acid phosphatase-like protein [Source:UniProtKB/Swiss-Prot;Acc:Q6ZNF0]; Transcript Identified by AceView. Entrez Gene ID(s) 390928
0.91	TTC25	tetratricopeptide repeat domain 25
0.91	IL32	interleukin 32
0.91	OR6F1; OR14A2	olfactory receptor. family 6. subfamily F. member 1; olfactory receptor. family 14. subfamily A. member 2
0.91	TEX38	testis expressed 38
0.91	AKAP17A; SFRS17A	Homo sapiens A kinase (PRKA) anchor protein 17A (AKAP17A). transcript variant 1. mRNA.; Homo sapiens A kinase (PRKA) anchor protein 17A (AKAP17A). transcript variant 2. non-coding RNA.; A kinase (PRKA) anchor protein 17A [Source:HGNC Symbol;Acc:HGNC:18783]; Homo sapiens splicing factor. arginine/serine-rich 17A. mRNA (cDNA clone MGC:39904 IMAGE:5217247). complete cds.; Homo sapiens splicing factor.

		arginine/serine-rich 17A. mRNA (cDNA clone MGC:125365 IMAGE:40007662). complete cds.; Homo sapiens splicing factor. arginine/serine-rich 17A. mRNA (cDNA clone MGC:125366 IMAGE:40007663). complete cds.; Jeck2013 ANNOTATED. INTERNAL. ncRNA. OVCODE. OVEXON best transcript NR_027383
0.91	SLC47A2	solute carrier family 47 (multidrug and toxin extrusion). member 2
0.91	PATL2	protein associated with topoisomerase II homolog 2 (yeast)
0.91	CGB2	chorionic gonadotropin. beta polypeptide 2
0.91	HDGFRP2	hepatoma-derived growth factor-related protein 2; Hepatoma-derived growth factor-related protein 2 [Source:UniProtKB/Swiss-Prot;Acc:Q7Z4V5]; Salzman2013 ANNOTATED. CDS. coding. OVCODE. OVEXON. UTR5 best transcript NM_001001520; Salzman2013 ANNOTATED. CDS. coding. OVCODE. OVERLAPTX. OVEXON. UTR3. UTR5 best transcript NM_001001520; Salzman2013 ANNOTATED. CDS. coding. INTERNAL. OVCODE. OVEXON best transcript NM_001001520; Salzman2013 ALT_ACCEPTOR. CDS. coding. INTERNAL. intronic. OVCODE. OVEXON best transcript NM_001001520; Salzman2013 ANNOTATED. CDS. coding. OVCODE. OVERLAPTX. OVEXON. UTR3 best transcript NM_001001520; Salzman2013 ALT_DONOR. CDS. coding. INTERNAL. OVCODE. OVERLAPTX. OVEXON. UTR3 best transcript NM_001001520
0.91	CST5	cystatin D
0.91	PTGER3	prostaglandin E receptor 3 (subtype EP3)
0.91	ARHGEF15	Rho guanine nucleotide exchange factor 15
0.91	MTA3	metastasis associated 1 family member 3
0.91	CDHR3	cadherin-related family member 3
0.91	CCNG2	cyclin G2
0.91	PTPN4	protein tyrosine phosphatase. non-receptor type 4 (megakaryocyte)
0.91	AOC1	amine oxidase. copper containing 1
0.91	TMEM144	transmembrane protein 144
0.91	CCNJL	cyclin J-like

0.91	DES	desmin
0.91	PLEKHG3	pleckstrin homology domain containing. family G (with RhoGef domain) member 3
0.91	FAM32A	family with sequence similarity 32. member A
0.91	EPM2AIP1	EPM2A (laforin) interacting protein 1
0.91	RALY	Jeck2013 ALT_DONOR. coding. INTERNAL. intronic best transcript NM_016732
0.91	NRXN2	neurexin 2
0.91	NDUFB1	NADH dehydrogenase (ubiquinone) 1 beta subcomplex. 1. 7kDa
0.91	HMBS	hydroxymethylbilane synthase
0.91	WRB	tryptophan rich basic protein
0.91	POU3F2	POU class 3 homeobox 2
0.91	MANF	mesencephalic astrocyte-derived neurotrophic factor
0.91	C11orf96	chromosome 11 open reading frame 96
0.91	IL17F	interleukin 17F
0.91	MAGEA9B; MAGEA9	MAGE family member A9B; MAGE family member A9
0.91	DISP1	dispatched homolog 1 (Drosophila)
0.91	PSD4	pleckstrin and Sec7 domain containing 4
0.91	BRD3	bromodomain containing 3
0.91	IL37	interleukin 37
0.91	CYP26B1	cytochrome P450. family 26. subfamily B. polypeptide 1
0.91	MACF1; KIAA0754	microtubule-actin crosslinking factor 1; KIAA0754
0.91	PCSK4	proprotein convertase subtilisin/kexin type 4
0.91	CAPRIN1	cell cycle associated protein 1
0.91	ATMIN	ATM interactor
0.91	PROK1	prokineticin 1
0.91	PRSS1	protease. serine. 1 (trypsin 1)

0.91	USP20	ubiquitin specific peptidase 20
0.91	RUFY1	Transcript Identified by AceView. Entrez Gene ID(s) 80230
0.91	FAM98A	family with sequence similarity 98. member A
0.91	CD37	CD37 molecule
0.91	CNNM3	Zhang2013 ALT_ACCEPTOR. ALT_DONOR. coding. INTERNAL. intronic. OVERLAPTX best transcript NM_017623
0.91	ZNF709	zinc finger protein 709
0.91	MMP7	matrix metallopeptidase 7
0.91	KCNA5	potassium channel. voltage gated shaker related subfamily A. member 5
0.91	SLC24A2	solute carrier family 24 (sodium/potassium/calcium exchanger). member 2
0.91	RPIA	ribose 5-phosphate isomerase A
0.91	KHDRBS1	KH domain containing. RNA binding. signal transduction associated 1
0.91	PTMA	prothymosin. alpha
0.91	NOL9	nucleolar protein 9
0.91	CCL1	chemokine (C-C motif) ligand 1
0.91	DENND1B	DENN/MADD domain containing 1B
0.91	CNR2	cannabinoid receptor 2
0.91	SUMO1	small ubiquitin-like modifier 1
0.91	TEAD1	TEA domain family member 1 (SV40 transcriptional enhancer factor)
0.91	ANAPC10	anaphase promoting complex subunit 10
0.91	KPNA3	karyopherin alpha 3 (importin alpha 4)
0.91	BPTF	bromodomain PHD finger transcription factor
0.91	YWHAG	tyrosine 3-monooxygenase/tryptophan 5-monooxygenase activation protein. gamma
0.91	FBXW5	F-box and WD repeat domain containing 5
0.91	GPC6	Transcript Identified by AceView. Entrez Gene ID(s) 10082
0.91	EXT1	exostosin glycosyltransferase 1

0.91	PACRG-AS3	PACRG antisense RNA 3
0.91	TNP1	transition protein 1 (during histone to protamine replacement)
0.91	UGT2B11	UDP glucuronosyltransferase 2 family. polypeptide B11
0.91	KATNA1	katanin p60 (ATPase containing) subunit A 1
0.91	ABHD17B	abhydrolase domain containing 17B
0.91	PIGL	phosphatidylinositol glycan anchor biosynthesis class L
0.91	DNM2	Transcript Identified by AceView. Entrez Gene ID(s) 1785
0.91	STKLD1	serine/threonine kinase-like domain containing 1 [Source:HGNC Symbol;Acc:HGNC:28669]
0.91	PLSCR1	phospholipid scramblase 1
0.91	ZFYVE26	zinc finger. FYVE domain containing 26
0.91	FAM101B	family with sequence similarity 101. member B
0.91	GPR84	G protein-coupled receptor 84
0.91	LOC729970; RP4-639F20.1	hCG2028352-like; novel transcript
0.91	SLITRK5	SLIT and NTRK-like family. member 5
0.91	DEK	DEK proto-oncogene
0.91	SKAP2	src kinase associated phosphoprotein 2
0.91	MOCS2	molybdenum cofactor synthesis 2
0.91	YPEL5	yippee like 5
0.91	BCL7B	B-cell CLL/lymphoma 7B
0.91	TLDC2	TBC/LysM-associated domain containing 2
0.91	NUDT16L1	nudix hydrolase 16 like 1
0.91	MYEOV2	myeloma overexpressed 2
0.91	UBE2L6	ubiquitin-conjugating enzyme E2L 6
0.91	MEF2C	myocyte enhancer factor 2C
0.91	PRDM14	PR domain containing 14

0.91	MARCO	macrophage receptor with collagenous structure
0.91	SMCR8	Smith-Magenis syndrome chromosome region. candidate 8
0.91	DOK6	docking protein 6
0.91	ZNF502	zinc finger protein 502
0.91	FAM156A	family with sequence similarity 156. member A
0.91	PAF1	PAF1 homolog. Paf1/RNA polymerase II complex component
0.91	CLDN17	claudin 17
0.91	OR2B11	olfactory receptor. family 2. subfamily B. member 11
0.91	SDR42E2	short chain dehydrogenase/reductase family 42E. member 2
0.91	DDX39B; SNORD84	DEAD (Asp-Glu-Ala-Asp) box polypeptide 39B; small nucleolar RNA. C/D box 84
0.91	CLTC	clathrin. heavy chain (Hc)
0.91	SPATA31E1	SPATA31 subfamily E. member 1
0.91	TLE1	transducin-like enhancer of split 1 (E(sp1) homolog. Drosophila)
0.91	ZNF625	zinc finger protein 625
0.91	CFAP65	cilia and flagella associated protein 65
0.91	AJAP1	adherens junctions associated protein 1
0.91	ADGRL4	adhesion G protein-coupled receptor L4
0.91	SENP1	SUMO1/sentrin specific peptidase 1
0.91	SLC35F6	solute carrier family 35. member F6
0.91	ZCCHC6	zinc finger. CCHC domain containing 6
0.91	CYP27B1	cytochrome P450. family 27. subfamily B. polypeptide 1
0.91	KLK8	kallikrein related peptidase 8
0.91	UBE2R2	ubiquitin-conjugating enzyme E2R 2
0.91	JPH1	junctophilin 1
0.91	FAM213B	Memczak2013 ANTISENSE. coding. INTERNAL. UTR3 best transcript NM_001195737

0.91	DCAF15	DDB1 and CUL4 associated factor 15
0.91	MRPS14	mitochondrial ribosomal protein S14
0.91	CRYGB	crystallin gamma B
0.91	SDC3	syndecan 3
0.91	APOL3	apolipoprotein L. 3
0.91	SGK3; C8orf44-SGK3	serum/glucocorticoid regulated kinase family. member 3; C8orf44-SGK3 readthrough
0.91	CCER2	coiled-coil glutamate rich protein 2
0.91	PSMC4	proteasome 26S subunit. ATPase 4
0.91	KCNK5	potassium channel. two pore domain subfamily K. member 5
0.91	MFSD14A	major facilitator superfamily domain containing 14A
0.91	GSTM5	glutathione S-transferase mu 5
0.91	TTC21B	tetratricopeptide repeat domain 21B
0.91	ANGPT2	angiopoietin 2
0.91	TAF15	Transcript Identified by AceView. Entrez Gene ID(s) 8148
0.91	CAPS	calcyphosine
0.91	MFSD3	major facilitator superfamily domain containing 3
0.91	ZNF546	zinc finger protein 546
0.91	HIST1H4L	histone cluster 1. H4l
0.91	HIPK4	homeodomain interacting protein kinase 4
0.91	KRT33B	keratin 33B. type I
0.91	MIOX	myo-inositol oxygenase
0.91	SNRPE	small nuclear ribonucleoprotein polypeptide E
0.91	ZNF619	zinc finger protein 619
0.91	ZW10	zw10 kinetochore protein
0.91	MYEF2	Transcript Identified by AceView. Entrez Gene ID(s) 50804

0.91	NCCRP1	non-specific cytotoxic cell receptor protein 1 homolog (zebrafish)
0.91	SLC22A18AS	solute carrier family 22 (organic cation transporter). member 18 antisense
0.91	RNASEK; C17orf49; RNASEK-C17orf49	ribonuclease. RNase K; chromosome 17 open reading frame 49; RNASEK-C17orf49 readthrough
0.91	ZNF354C	zinc finger protein 354C
0.91	MAGEA9; MAGEA9B	MAGE family member A9; MAGE family member A9B
0.91	OR52A1	olfactory receptor. family 52. subfamily A. member 1
0.91	SPN	sialophorin
0.91	L3MBTL4	Memczak2013 ANTISENSE. coding. INTERNAL. intronic best transcript NM_173464
0.91	PIGB	phosphatidylinositol glycan anchor biosynthesis class B
0.91	CDON	cell adhesion associated. oncogene regulated
0.91	ARHGEF25	Rho guanine nucleotide exchange factor 25
0.91	FSTL3	follistatin-like 3 (secreted glycoprotein)
0.91	KRT39	keratin 39. type I
0.91	AES	amino-terminal enhancer of split
0.91	ANKRD11	ankyrin repeat domain 11
0.91	CDR1; CDR1-AS	Memczak2013 ANTISENSE. CDS. coding. downstream_end. upstream_start. UTR3. UTR5 best transcript NM_004065; CDR1 antisense RNA; CDR1 antisense RNA [Source:HGNC Symbol;Acc:HGNC:48926]
0.91	LINC00934	long intergenic non-protein coding RNA 934
0.91	OR52Z1	olfactory receptor. family 52. subfamily Z. member 1 (gene/pseudogene)
0.91	ZNF316	zinc finger protein 316
0.91	AMY1C; AMY1A	amylase. alpha 1C (salivary); amylase. alpha 1A (salivary)
0.91	CCDC180	coiled-coil domain containing 180
0.91	RPS19BP1	ribosomal protein S19 binding protein 1
0.91	GLOD4	glyoxalase domain containing 4

0.91	CD180	CD180 molecule
0.91	DDX19A	DEAD (Asp-Glu-Ala-Asp) box polypeptide 19A
0.91	NF2	neurofibromin 2 (merlin)
0.91	RGS5	regulator of G-protein signaling 5
0.91	GALM	galactose mutarotase (aldose 1-epimerase)
0.91	CTDP1	CTD phosphatase subunit 1
0.91	AICDA	activation-induced cytidine deaminase
0.91	EBLN2	endogenous Bornavirus-like nucleoprotein 2
0.91	FAM214B	family with sequence similarity 214. member B
0.91	HAT1	histone acetyltransferase 1
0.91	OR52E1	olfactory receptor. family 52. subfamily E. member 1 (gene/pseudogene)
0.91	MARK2	MAP/microtubule affinity-regulating kinase 2
0.91	MECOM	MDS1 and EVI1 complex locus
0.91	ATP1B1	ATPase. Na+/K+ transporting. beta 1 polypeptide
0.91	COPA	coatomer protein complex subunit alpha
0.91	GRAMD3	GRAM domain containing 3
0.91	UNC5D	unc-5 netrin receptor D
0.91	LINC01620	long intergenic non-protein coding RNA 1620
0.91	CCR2	chemokine (C-C motif) receptor 2
0.91	CTSF	cathepsin F
0.91	SLC5A1	solute carrier family 5 (sodium/glucose cotransporter). member 1
0.91	CERK	ceramide kinase
0.91	ZNF365	zinc finger protein 365
0.91	HLA-E	major histocompatibility complex. class I. E
0.91	CLDN15	claudin 15
0.91	MBIP	MAP3K12 binding inhibitory protein 1

0.91	dorsnarby; IGF2BP2	Transcript Identified by AceView; Zhang2013 ALT_ACCEPTOR. ALT_DONOR. coding. INTERNAL. intronic best transcript NM_006548
0.91	GUCA1B	guanylate cyclase activator 1B (retina)
0.91	CCS	copper chaperone for superoxide dismutase
0.91	OR10G2	olfactory receptor. family 10. subfamily G. member 2
0.91	HTRA1	HtrA serine peptidase 1
0.91	OR5M3	olfactory receptor. family 5. subfamily M. member 3
0.91	MSH5; MSH5-SAPCD1; SAPCD1	mutS homolog 5; MSH5-SAPCD1 readthrough (NMD candidate); suppressor APC domain containing 1
0.91	TRMT112	tRNA methyltransferase 11-2 homolog (S. cerevisiae)
0.91	TMEM241	transmembrane protein 241
0.91	PRPF38A	pre-mRNA processing factor 38A
0.91	ZNF124	zinc finger protein 124
0.91	ELP4	elongator acetyltransferase complex subunit 4
0.91	GPR20	G protein-coupled receptor 20
0.91	BMP2	bone morphogenetic protein 2
0.91	MED12	Memczak2013 ANTISENSE. CDS. coding. INTERNAL best transcript NM_005120
0.91	CBWD3	COBW domain containing 3
0.91	EID3	EP300 interacting inhibitor of differentiation 3
0.91	RNF126	ring finger protein 126
0.91	PHYH	phytanoyl-CoA 2-hydroxylase
0.91	PDGFRL	platelet-derived growth factor receptor-like
0.91	RPRM	reprimo. TP53 dependent G2 arrest mediator candidate
0.91	NLRP6	NLR family. pyrin domain containing 6
0.91	RAB29	RAB29. member RAS oncogene family
0.91	TCF7L1	transcription factor 7-like 1 (T-cell specific. HMG-box)

0.91	EXOC6B	exocyst complex component 6B
0.91	TSPAN2	tetraspanin 2
0.91	SPAG8	sperm associated antigen 8
0.91	UBE2V1	ubiquitin conjugating enzyme E2 variant 1
0.91	SHROOM2	shroom family member 2
0.91	C16orf82	chromosome 16 open reading frame 82
0.91	C2orf57	chromosome 2 open reading frame 57
0.91	TISP43; LOC646743; AC013269.5	uncharacterized LOC150527; uncharacterized LOC646743; Transcript Identified by AceView. Entrez Gene ID(s) 646743. RefSeq ID(s) NR_033930; Uncharacterized protein [Source:UniProtKB/TrEMBL;Acc:B9A039]
0.91	OR9I1	olfactory receptor. family 9. subfamily I. member 1
0.91	GRWD1	glutamate-rich WD repeat containing 1
0.91	SAFB2	scaffold attachment factor B2
0.91	TBC1D17; MIR4750	TBC1 domain family. member 17; microRNA 4750
0.91	HRASLS2	HRAS-like suppressor 2
0.91	POLR2B	polymerase (RNA) II (DNA directed) polypeptide B. 140kDa
0.91	LYRM5	LYR motif containing 5
0.91	CFAP46	cilia and flagella associated protein 46
0.91	PRPF19	Memczak2013 ALT_ACCEPTOR. ALT_DONOR. coding. INTERNAL. intronic best transcript NM_014502
0.91	PSEN1	presenilin 1
0.91	MSRB2	methionine sulfoxide reductase B2
0.91	SMARCA1	SWI/SNF related. matrix associated. actin dependent regulator of chromatin. subfamily a. member 1
0.91	SET	SET nuclear proto-oncogene
0.91	APEX2	APEX nuclease (apurinic/apyrimidinic endonuclease) 2
0.91	LDHAL6A	lactate dehydrogenase A-like 6A

0.91	AP1B1	adaptor-related protein complex 1. beta 1 subunit
0.91	goyborbu; MCM2	Transcript Identified by AceView; Memczak2013 ANTISENSE. CDS. coding. INTERNAL. UTR3 best transcript NM_004526
0.91	RC3H2	ring finger and CCCH-type domains 2
0.91	C12orf56	chromosome 12 open reading frame 56
0.91	CARD11	caspase recruitment domain family. member 11
0.91	CDK11A	cyclin-dependent kinase 11A
0.91	USP44	ubiquitin specific peptidase 44
0.91	PPP1R13L	protein phosphatase 1. regulatory subunit 13 like
0.91	TMEM87A	transmembrane protein 87A
0.91	AMOT; MIR4329	angiomotin; microRNA 4329
0.91	LRIT1	leucine-rich repeat. immunoglobulin-like and transmembrane domains 1
0.91	DAZ4; DAZ1	deleted in azoospermia 4; deleted in azoospermia 1
0.91	TH	tyrosine hydroxylase
0.91	DMRTC1B; DMRTC1	DMRT-like family C1B; DMRT-like family C1
0.91	CYP26C1	cytochrome P450. family 26. subfamily C. polypeptide 1
0.91	OXTR	oxytocin receptor
0.91	SLC30A10	solute carrier family 30. member 10
0.91	RNF135	ring finger protein 135
0.91	KIFC3	kinesin family member C3
0.91	PLA2G12A	phospholipase A2. group XIIA
0.91	RNF113B	ring finger protein 113B
0.91	MRPS26	mitochondrial ribosomal protein S26
0.91	INHA	inhibin alpha
0.91	TBPL2	TATA box binding protein like 2
0.91	TMTC2	transmembrane and tetratricopeptide repeat containing 2

0.91	PMF1; PMF1-BGLAP; BGLAP	polyamine-modulated factor 1; PMF1-BGLAP readthrough; bone gamma-carboxyglutamate (gla) protein
0.91	TSPAN9	tetraspanin 9
0.91	LEFTY1	left-right determination factor 1
0.91	FGF21	fibroblast growth factor 21
0.91	NT5C1B; NT5C1B-RDH14; RDH14	5-nucleotidase. cytosolic IB; NT5C1B-RDH14 readthrough; retinol dehydrogenase 14 (all-trans/9-cis/11-cis)
0.91	UBXN2B	UBX domain protein 2B
0.91	RHOXF1	Rhox homeobox family. member 1
0.91	DOK7	docking protein 7
0.91	GLMN	glomulin. FKBP associated protein
0.91	C16orf46	chromosome 16 open reading frame 46
0.91	DXO	decapping exoribonuclease
0.91	MRO	maestro
0.91	C19orf12	chromosome 19 open reading frame 12
0.91	USP37	ubiquitin specific peptidase 37
0.91	ISOC2	isochorismatase domain containing 2
0.91	CCDC9	coiled-coil domain containing 9
0.91	SPINK7	serine peptidase inhibitor. Kazal type 7 (putative)
0.91	EP300; MIR1281	E1A binding protein p300; microRNA 1281
0.91	DIS3L2	DIS3 like 3-5 exoribonuclease 2
0.91	TRIM66	tripartite motif containing 66
0.91	MYBPHL	myosin binding protein H-like
0.91	FSHB	follicle stimulating hormone. beta polypeptide
0.91	FAM84B	family with sequence similarity 84. member B
0.91	AMHR2	anti-Mullerian hormone receptor. type II
0.91	SFRP4	secreted frizzled-related protein 4

0.91	SMCO4	single-pass membrane protein with coiled-coil domains 4
0.91	DDX6	DEAD (Asp-Glu-Ala-Asp) box helicase 6
0.91	BLCAP	bladder cancer associated protein
0.91	FXYD7	FXYD domain containing ion transport regulator 7
0.91	BDKRB2	bradykinin receptor B2
0.91	DHODH	dihydroorotate dehydrogenase (quinone)
0.91	FXYD1	FXYD domain containing ion transport regulator 1
0.91	RFESD	Rieske (Fe-S) domain containing
0.91	MAST4	microtubule associated serine/threonine kinase family member 4
0.91	BANK1	B-cell scaffold protein with ankyrin repeats 1
0.91	NIPA2	non imprinted in Prader-Willi/Angelman syndrome 2
0.91	C7orf33	chromosome 7 open reading frame 33
0.91	USP10	ubiquitin specific peptidase 10
0.91	HLA-F	major histocompatibility complex. class I. F
0.91	C1QL3	complement component 1. q subcomponent-like 3
0.91	HIST3H2A	histone cluster 3. H2a
0.91	CD36	CD36 molecule (thrombospondin receptor)
0.90	ALG1L2	ALG1. chitobiosyldiphosphodolichol beta-mannosyltransferase-like 2
0.90	TTC9B	tetratricopeptide repeat domain 9B
0.90	CNPY3	Memczak2013 ALT_ACCEPTOR. ALT_DONOR. coding. INTERNAL. intronic best transcript NM_006586
0.90	FIGF	c-fos induced growth factor (vascular endothelial growth factor D)
0.90	NFKBIZ	nuclear factor of kappa light polypeptide gene enhancer in B-cells inhibitor. zeta
0.90	DAXX	death-domain associated protein
0.90	FBXL19	F-box and leucine-rich repeat protein 19
0.90	NFATC1	nuclear factor of activated T-cells. cytoplasmic. calcineurin-dependent 1

0.90	C1orf168	chromosome 1 open reading frame 168
0.90	OPLAH	5-oxoprolinase (ATP-hydrolysing)
0.90	SULT1C4	sulfotransferase family 1C member 4
0.90	INHBA	inhibin beta A
0.90	LCN1	lipocalin 1
0.90	C20orf166-AS1	C20orf166 antisense RNA 1
0.90	CA14	carbonic anhydrase XIV
0.90	TM4SF4	transmembrane 4 L six family member 4
0.90	TRIM7	tripartite motif containing 7
0.90	USP1	ubiquitin specific peptidase 1
0.90	FITM2	fat storage-inducing transmembrane protein 2
0.90	EPHA4	Transcript Identified by AceView. Entrez Gene ID(s) 2043
0.90	VAX2	ventral anterior homeobox 2
0.90	PSMA7	proteasome subunit alpha 7
0.90	MYO1D	myosin ID
0.90	MRPL23	mitochondrial ribosomal protein L23
0.90	MEX3B	mex-3 RNA binding family member B
0.90	RALGAPA1	Ral GTPase activating protein. alpha subunit 1 (catalytic)
0.90	TEPP	testis. prostate and placenta expressed
0.90	IL7	interleukin 7
0.90	FAM19A1	family with sequence similarity 19 (chemokine (C-C motif)-like). member A1
0.90	SBF1	SET binding factor 1
0.90	UBL4A	ubiquitin-like 4A
0.90	ZC3H8	zinc finger CCCH-type containing 8
0.90	DNAH10	dynein. axonemal. heavy chain 10
0.90	FGA	fibrinogen alpha chain

0.90	CD48	CD48 molecule
0.90	ADGRB1	adhesion G protein-coupled receptor B1
0.90	C1orf204	chromosome 1 open reading frame 204
0.90	BRCC3	BRCA1/BRCA2-containing complex subunit 3
0.90	ULBP1	UL16 binding protein 1
0.90	FAM217A	family with sequence similarity 217. member A
0.90	FAM170B	family with sequence similarity 170. member B
0.90	ADGRF5	adhesion G protein-coupled receptor F5
0.90	ARHGAP23	Rho GTPase activating protein 23
0.90	CACYBP	calcyclin binding protein
0.90	GBX1	gastrulation brain homeobox 1
0.90	QARS; MIR6890	glutaminyl-tRNA synthetase; microRNA 6890
0.90	STK17B	serine/threonine kinase 17b
0.90	SPINK14	serine peptidase inhibitor. Kazal type 14 (putative)
0.90	LIN52	lin-52 DREAM MuvB core complex component
0.90	UBE2S; UBE2SP1	ubiquitin-conjugating enzyme E2S; ubiquitin conjugating enzyme E2S pseudogene 1
0.90	RPA1	replication protein A1
0.90	CEACAM16	carcinoembryonic antigen-related cell adhesion molecule 16
0.90	AK8	adenylate kinase 8
0.90	DNM1L	dynamin 1-like
0.90	CCNG2	cyclin G2
0.90	ARNT2; MIR5572	aryl-hydrocarbon receptor nuclear translocator 2; microRNA 5572
0.90	VPS11	VPS11. CORVET/HOPS core subunit [Source:HGNC Symbol;Acc:HGNC:14583]
0.90	SPATA31A1	SPATA31 subfamily A. member 1
0.90	GNAS	GNAS complex locus

0.90	GLIS3	GLIS family zinc finger 3
0.90	NAT14	N-acetyltransferase 14 (GCN5-related. putative)
0.90	CYSLTR2	cysteinyl leukotriene receptor 2
0.90	CFTR	cystic fibrosis transmembrane conductance regulator
0.90	OSBPL2	oxysterol binding protein-like 2
0.90	SMOC2	SPARC related modular calcium binding 2
0.90	LARGE	Transcript Identified by AceView. Entrez Gene ID(s) 9215
0.90	ZNF606	zinc finger protein 606
0.90	CDK11B	cyclin-dependent kinase 11B
0.90	KRT34	keratin 34. type I
0.90	PLXNA2	plexin A2
0.90	VIM-AS1	VIM antisense RNA 1
0.90	METTL17	methyltransferase like 17
0.90	TLX3	T-cell leukemia homeobox 3
0.90	ADRB2	adrenoceptor beta 2. surface
0.90	DDO	D-aspartate oxidase
0.90	CDC123	cell division cycle 123
0.90	ARHGEF37	Rho guanine nucleotide exchange factor 37
0.90	AKR1A1	aldo-keto reductase family 1. member A1 (aldehyde reductase)
0.90	ZSWIM1	zinc finger. SWIM-type containing 1
0.90	KRT18P55	keratin 18 pseudogene 55
0.90	CDIP1	cell death-inducing p53 target 1
0.90	SPG11	spastic paraplegia 11 (autosomal recessive)
0.90	TMED8	transmembrane p24 trafficking protein family member 8
0.90	CXCL9	chemokine (C-X-C motif) ligand 9
0.90	PARP2	poly(ADP-ribose) polymerase 2

0.90	CRISP3	cysteine-rich secretory protein 3
0.90	OPCML	opioid binding protein/cell adhesion molecule-like
0.90	OR2T2	olfactory receptor. family 2. subfamily T. member 2
0.90	ZNF836	zinc finger protein 836
0.90	EAPP	E2F-associated phosphoprotein
0.90	SLC29A2	solute carrier family 29 (equilibrative nucleoside transporter). member 2
0.90	DEFB104A; DEFB104B	defensin. beta 104A; defensin. beta 104B
0.90	CDH22	Transcript Identified by AceView. Entrez Gene ID(s) 64405
0.90	BPHL	biphenyl hydrolase-like (serine hydrolase)
0.90	FUS	FUS RNA binding protein
0.90	CMKLR1	chemerin chemokine-like receptor 1
0.90	EFNB3	ephrin-B3
0.90	ATP11AUN	ATP11A upstream neighbor
0.90	CD79B	CD79b molecule. immunoglobulin-associated beta
0.90	MAFA	v-maf avian musculoaponeurotic fibrosarcoma oncogene homolog A
0.90	LINC01089	long intergenic non-protein coding RNA 1089
0.90	ZFP37	ZFP37 zinc finger protein
0.90	MYO1G	Memczak2013 ANTISENSE. CDS. coding. INTERNAL best transcript NM_033054
0.90	NDUFA10	NADH dehydrogenase (ubiquinone) 1 alpha subcomplex. 10. 42kDa
0.90	LRP6	LDL receptor related protein 6
0.90	UBE2L3	ubiquitin conjugating enzyme E2L 3
0.90	BRI3BP	BRI3 binding protein
0.90	NAIP	NLR family. apoptosis inhibitory protein
0.90	DCLK2	doublecortin-like kinase 2
0.90	DNAJB13	DnaJ (Hsp40) homolog. subfamily B. member 13

0.90	SPR	sepiapterin reductase (7.8-dihydrobiopterin:NADP+ oxidoreductase)
0.90	CNIH3	cornichon family AMPA receptor auxiliary protein 3
0.90	CFD	complement factor D (adipsin)
0.90	PPP6R2	protein phosphatase 6. regulatory subunit 2
0.90	SMIM5	small integral membrane protein 5
0.90	MINOS1	mitochondrial inner membrane organizing system 1
0.90	METTL20	methyltransferase like 20
0.90	AP1G2	adaptor-related protein complex 1. gamma 2 subunit
0.90	RMDN2	regulator of microtubule dynamics 2
0.90	RAMP3	receptor (G protein-coupled) activity modifying protein 3
0.90	ZCCHC16	zinc finger. CCHC domain containing 16
0.90	PSME3	proteasome activator subunit 3
0.90	CARF	calcium responsive transcription factor
0.90	NOS1	nitric oxide synthase 1 (neuronal)
0.90	UPP2	uridine phosphorylase 2
0.90	ALG1L9P	asparagine-linked glycosylation 1-like 9. pseudogene
0.90	C1QB	complement component 1. q subcomponent. B chain
0.90	POPDC2	popeye domain containing 2
0.90	UNC119B; MIR4700	unc-119 lipid binding chaperone B; microRNA 4700
0.90	ADAM29	ADAM metallopeptidase domain 29
0.90	PDE3B	phosphodiesterase 3B. cGMP-inhibited
0.90	HMBS	hydroxymethylbilane synthase
0.90	ADCY1	adenylate cyclase 1 (brain)
0.90	TEKT1	tektin 1
0.90	IL15RA	interleukin 15 receptor. alpha
0.90	TUBB2A	tubulin. beta 2A class IIa

0.90	CXXC5	CXXC finger protein 5
0.90	HERC2P2	hect domain and RLD 2 pseudogene 2
0.90	SERPINA12	serpin peptidase inhibitor. clade A (alpha-1 antiproteinase. antitrypsin). member 12
0.90	ADAM28	ADAM metallopeptidase domain 28
0.90	SLC16A10	solute carrier family 16 (aromatic amino acid transporter). member 10
0.90	HR	hair growth associated
0.90	MAL2	mal. T-cell differentiation protein 2 (gene/pseudogene)
0.90	C9orf92	chromosome 9 open reading frame 92
0.90	SYNRG	synergin. gamma
0.90	FOXD4	forkhead box D4
0.90	LEUTX	leucine twenty homeobox
0.90	ALOXE3	arachidonate lipoxygenase 3
0.90	OR4D2	olfactory receptor. family 4. subfamily D. member 2
0.90	CIPC	CLOCK-interacting pacemaker
0.90	ERCC6L2	excision repair cross-complementation group 6-like 2
0.90	SLC10A3	solute carrier family 10. member 3
0.90	IQGAP2	IQ motif containing GTPase activating protein 2
0.90	ZNF783	zinc finger family member 783
0.90	SHISA7	shisa family member 7
0.90	UBE2U	ubiquitin-conjugating enzyme E2U (putative)
0.90	HOXA7	homeobox A7
0.90	FCGR2B	Fc fragment of IgG. low affinity IIb. receptor (CD32)
0.90	HIST1H4C	histone cluster 1. H4c
0.90	CBR4	carbonyl reductase 4
0.90	PGD	phosphogluconate dehydrogenase

0.90	LUZP1	leucine zipper protein 1
0.90	OR4C45	olfactory receptor. family 4. subfamily C. member 45
0.90	SYCE3	synaptonemal complex central element protein 3
0.90	MRPS28	mitochondrial ribosomal protein S28
0.90	SMR3B	submaxillary gland androgen regulated protein 3B
0.90	FBXW10	F-box and WD repeat domain containing 10
0.90	LATS2	large tumor suppressor kinase 2
0.90	UXT	ubiquitously expressed prefoldin like chaperone
0.90	RAET1G	retinoic acid early transcript 1G
0.90	NENF	neudesin neurotrophic factor
0.90	TDRD3	tudor domain containing 3
0.90	ADSSL1	adenylosuccinate synthase like 1
0.90	NFE2L2	nuclear factor. erythroid 2-like 2
0.90	AQP6	aquaporin 6. kidney specific
0.90	VILL	villin-like
0.90	GFRA2	GDNF family receptor alpha 2
0.90	TMEM248	transmembrane protein 248
0.90	GDF5	growth differentiation factor 5
0.90	HKR1	HKR1. GLI-Kruppel zinc finger family member
0.90	RET	ret proto-oncogene
0.90	CISH	cytokine inducible SH2-containing protein
0.90	STATH	statherin
0.90	CCDC188	coiled-coil domain containing 188
0.90	TBATA	thymus. brain and testes associated
0.90	MFSD7	major facilitator superfamily domain containing 7
0.90	APOC4	apolipoprotein C-IV

0.90	C20orf96	chromosome 20 open reading frame 96
0.90	FXYD2	FXYD domain containing ion transport regulator 2
0.90	CDC27	cell division cycle 27
0.90	NUP88	nucleoporin 88kDa
0.90	PEX5L	peroxisomal biogenesis factor 5-like
0.90	RAB44	RAB44. member RAS oncogene family
0.90	CBARP	calcium channel. voltage-dependent. beta subunit associated regulatory protein
0.90	ELF3	E74-like factor 3 (ets domain transcription factor. epithelial-specific)
0.90	ATP6AP2	ATPase. H+ transporting. lysosomal accessory protein 2
0.90	DBP	D site of albumin promoter (albumin D-box) binding protein
0.90	C15orf65	chromosome 15 open reading frame 65
0.90	ATP5SL	ATP5S-like
0.90	CELF2	CUGBP. Elav-like family member 2
0.90	CCR8	chemokine (C-C motif) receptor 8
0.90	SIGLEC11	sialic acid binding Ig-like lectin 11
0.90	BAG4	BCL2-associated athanogene 4
0.90	OR4B1	olfactory receptor. family 4. subfamily B. member 1
0.90	OR2L8	olfactory receptor. family 2. subfamily L. member 8 (gene/pseudogene)
0.90	CCDC154	coiled-coil domain containing 154
0.90	LPP	LIM domain containing preferred translocation partner in lipoma
0.90	POP4	POP4 homolog. ribonuclease P/MRP subunit
0.90	LOC100506142; RP11-417F21.1; RHOQ	uncharacterized LOC100506142; Memczak2013 ANTISENSE. coding. INTERNAL. intronic best transcript NM_012249; novel transcript
0.90	DLG3	discs. large homolog 3 (Drosophila)
0.90	TRAM1	translocation associated membrane protein 1
0.90	NTF3	neurotrophin 3

0.90	SGEF	Transcript Identified by AceView. Entrez Gene ID(s) 26084
0.90	CFAP69	cilia and flagella associated protein 69
0.90	PNRC1	proline-rich nuclear receptor coactivator 1
0.90	GLRB	glycine receptor beta
0.90	CCDC85A	Transcript Identified by AceView. Entrez Gene ID(s) 114800
0.90	REXO1L11P	REX1. RNA exonuclease 1 homolog-like 11. pseudogene
0.90	MKRN3	makorin ring finger protein 3
0.90	B3GALT6	UDP-Gal:betaGal beta 1.3-galactosyltransferase 6
0.90	PTCHD2	patched domain containing 2
0.90	FAM120C	family with sequence similarity 120C
0.90	RIC8B	RIC8 guanine nucleotide exchange factor B
0.90	PLEKHG6	pleckstrin homology domain containing. family G (with RhoGef domain) member 6
0.90	ACTBL2	actin. beta-like 2
0.90	FRMPD4	FERM and PDZ domain containing 4
0.90	SMIM22	small integral membrane protein 22
0.90	VAV1	vav 1 guanine nucleotide exchange factor
0.90	EEF1A2	eukaryotic translation elongation factor 1 alpha 2
0.90	RCCD1	RCC1 domain containing 1
0.90	POU2F1	POU class 2 homeobox 1
0.90	CYB5R3	cytochrome b5 reductase 3
0.90	PLPPR5	phospholipid phosphatase related 5
0.90	SH3BGRL2	Transcript Identified by AceView. Entrez Gene ID(s) 83699
0.90	ART5	ADP-ribosyltransferase 5
0.90	UFC1	ubiquitin-fold modifier conjugating enzyme 1
0.90	TIGD1; MIR5001	tigger transposable element derived 1; microRNA 5001

0.90	PAG1	phosphoprotein membrane anchor with glycosphingolipid microdomains 1
0.90	KLHDC1	kelch domain containing 1
0.90	ADD3	adducin 3 (gamma)
0.90	SORCS2	sortilin-related VPS10 domain containing receptor 2
0.90	DCTN3	dynactin 3 (p22)
0.90	SETD3	Transcript Identified by AceView. Entrez Gene ID(s) 84193
0.90	CDYL2	chromodomain protein. Y-like 2
0.90	GLI4	GLI family zinc finger 4
0.90	TPRG1	tumor protein p63 regulated 1
0.90	TMIGD2	transmembrane and immunoglobulin domain containing 2
0.90	HNRNPK	Zhang2013 ALT_ACCEPTOR. ALT_DONOR. coding. INTERNAL. intronic. OVERLAPTX best transcript NM_002140
0.90	MAT2B	methionine adenosyltransferase II. beta
0.90	CCDC85C	coiled-coil domain containing 85C
0.90	ZNF705CP	zinc finger protein 705C. pseudogene
0.90	NAGA	N-acetylgalactosaminidase. alpha-
0.90	PLCD1	phospholipase C. delta 1
0.90	FDX1L	ferredoxin 1-like
0.90	IQCC	IQ motif containing C
0.90	NEIL1	nei-like DNA glycosylase 1
0.90	DAB2	Dab. mitogen-responsive phosphoprotein. homolog 2 (Drosophila)
0.90	B4GAT1	beta-1.4-glucuronyltransferase 1
0.90	OR6N1	olfactory receptor. family 6. subfamily N. member 1
0.90	TMEM132A	transmembrane protein 132A
0.90	COX19	COX19 cytochrome c oxidase assembly factor
0.90	FRG2B	FSHD region gene 2 family. member B

0.90	DPP7	dipeptidyl-peptidase 7
0.90	COX11	COX11 cytochrome c oxidase copper chaperone
0.90	PLXNB2	plexin B2
0.90	EPHA8	EPH receptor A8
0.90	MMP8	matrix metallopeptidase 8
0.90	H2AFV	H2A histone family. member V
0.90	F5	coagulation factor V (proaccelerin. labile factor)
0.90	MYO15B	Memczak2013 ANTISENSE. INTERNAL. ncRNA best transcript NR_003587
0.90	SLC52A1	solute carrier family 52 (riboflavin transporter). member 1
0.90	HLA-DOB	major histocompatibility complex. class II. DO beta
0.90	ZNF44	zinc finger protein 44
0.90	XPO7	exportin 7
0.90	SMC5	structural maintenance of chromosomes 5
0.90	RPA4	replication protein A4
0.90	BLK	Transcript Identified by AceView. Entrez Gene ID(s) 640
0.90	FKBP9	FK506 binding protein 9
0.90	SLC16A11	solute carrier family 16. member 11
0.90	CIC	capicua transcriptional repressor
0.90	BVES	blood vessel epicardial substance
0.90	ARL10	ADP-ribosylation factor like GTPase 10
0.90	CMC1	C-x(9)-C motif containing 1
0.90	PIBF1	progesterone immunomodulatory binding factor 1
0.90	ZNF395	zinc finger protein 395
0.90	PSD3	pleckstrin and Sec7 domain containing 3
0.90	PATE4	prostate and testis expressed 4
0.90	AKT1	v-akt murine thymoma viral oncogene homolog 1

0.90	GADD45G	growth arrest and DNA-damage-inducible. gamma
0.90	LINC00649	long intergenic non-protein coding RNA 649
0.90	C1QL1	complement component 1. q subcomponent-like 1
0.90	CXorf23	chromosome X open reading frame 23
0.90	BHLHA9	basic helix-loop-helix family. member a9
0.90	NAP1L3	nucleosome assembly protein 1-like 3
0.90	SEC14L6	SEC14-like lipid binding 6
0.90	ENOX2	ecto-NOX disulfide-thiol exchanger 2
0.90	DUSP2	dual specificity phosphatase 2
0.90	EGF	epidermal growth factor
0.90	FAM3A	family with sequence similarity 3. member A
0.90	SPATA5L1	spermatogenesis associated 5-like 1
0.90	MAGEE1	MAGE family member E1
0.90	PLPP2	phospholipid phosphatase 2
0.90	BPI	bactericidal/permeability-increasing protein
0.90	TGOLN2	trans-golgi network protein 2
0.90	MRPL37	mitochondrial ribosomal protein L37
0.90	OAZ1	ornithine decarboxylase antizyme 1
0.90	METTL11B	methyltransferase like 11B
0.90	RASSF5	Ras association (RalGDS/AF-6) domain family member 5
0.90	CAMKK2	calcium/calmodulin-dependent protein kinase kinase 2. beta
0.90	SCAMP2	secretory carrier membrane protein 2
0.90	TTLL1	tubulin tyrosine ligase-like family member 1
0.90	METTL12; SNORA57	methyltransferase like 12; small nucleolar RNA. H/ACA box 57
0.90	LIMS2	LIM and senescent cell antigen-like domains 2
0.90	CBX7	chromobox homolog 7

0.90	ANKS3	ankyrin repeat and sterile alpha motif domain containing 3
0.90	MTRF1L	mitochondrial translational release factor 1-like
0.90	AMBP	alpha-1-microglobulin/bikunin precursor
0.90	TRAPPC4	trafficking protein particle complex 4
0.90	CBLC	Cbl proto-oncogene C. E3 ubiquitin protein ligase
0.90	PPEF2	protein phosphatase. EF-hand calcium binding domain 2
0.90	C9orf147; HSDL2	Transcript Identified by AceView. Entrez Gene ID(s) 100133204; Salzman2013 ANTISENSE. CDS. coding. downstream_end. intronic. OVCODE. OVERLAPTX. OVEXON. UTR3 best transcript NM_032303; chromosome 9 open reading frame 147; chromosome 9 open reading frame 147 [Source:HGNC Symbol;Acc:HGNC:31438]
0.90	ZNF814	zinc finger protein 814
0.90	C19orf47	chromosome 19 open reading frame 47
0.90	PSMB4	proteasome subunit beta 4
0.90	ARL6IP1	ADP-ribosylation factor like GTPase 6 interacting protein 1
0.90	ZNF18	zinc finger protein 18
0.90	VAMP3	vesicle associated membrane protein 3
0.90	ZKSCAN7	zinc finger with KRAB and SCAN domains 7
0.90	KRTAP5-3	keratin associated protein 5-3
0.90	ISL1	Transcript Identified by AceView. Entrez Gene ID(s) 3670
0.90	BEST4	bestrophin 4
0.90	PSG7	pregnancy specific beta-1-glycoprotein 7 (gene/pseudogene)
0.90	CASP1	caspase 1
0.90	FOCAD	focadhesin
0.90	MAGEB18	MAGE family member B18
0.90	RGP1	RGP1 homolog. RAB6A GEF complex partner 1
0.90	RCN2	reticulocalbin 2. EF-hand calcium binding domain

0.90	CCDC117	coiled-coil domain containing 117
0.90	ERVMER34-1	endogenous retrovirus group MER34. member 1
0.90	CACNA1S	calcium channel. voltage-dependent. L type. alpha 1S subunit
0.90	PRKAR1B	protein kinase. cAMP-dependent. regulatory. type I. beta
0.90	SIGLEC16	sialic acid binding Ig-like lectin 16 (gene/pseudogene)
0.90	THBS3	thrombospondin 3
0.90	B3GAT1	beta-1.3-glucuronyltransferase 1
0.90	SDPR	serum deprivation response
0.90	C20orf78	chromosome 20 open reading frame 78
0.90	SDCBP2	syndecan binding protein (syntenin) 2
0.90	ASMT	Homo sapiens acetylserotonin O-methyltransferase (ASMT). transcript variant 2. mRNA.
0.90	PERM1	PPARGC1 and ESRR induced regulator. muscle 1
0.90	FANCF	Fanconi anemia complementation group F
0.90	POU6F2-AS2; POU6F2; AC011292.2	POU6F2 antisense RNA 2 [Source:HGNC Symbol;Acc:HGNC:21887]; Salzman2013 ANTISENSE. coding. INTERNAL. intronic. OVERLAPTX best transcript NM_007252; novel transcript. antisense to POU6F2
0.90	GALNT5	polypeptide N-acetylgalactosaminyltransferase 5
0.90	ICA1	islet cell autoantigen 1
0.90	MEGF8	multiple EGF-like-domains 8
0.90	PRKAB2	protein kinase. AMP-activated. beta 2 non-catalytic subunit
0.90	CWF19L2	CWF19-like 2. cell cycle control (S. pombe)
0.90	LMNB2; MIR7108	lamin B2; microRNA 7108
0.90	RLN3	relaxin 3
0.90	KIAA0141	KIAA0141
0.90	HFE2	hemochromatosis type 2 (juvenile)
0.90	CLUH	clustered mitochondria (cluA/CLU1) homolog

0.90	WFDC10B	WAP four-disulfide core domain 10B
0.90	DLL1	delta-like 1 (Drosophila)
0.90	FAM212A	family with sequence similarity 212. member A
0.90	NDUFB2	NADH dehydrogenase (ubiquinone) 1 beta subcomplex. 2. 8kDa
0.90	HCG9	HLA complex group 9 (non-protein coding)
0.90	SCD5	stearoyl-CoA desaturase 5
0.90	ATP6V0A1; MIR5010	ATPase. H+ transporting. lysosomal V0 subunit a1; microRNA 5010
0.90	BCL2	B-cell CLL/lymphoma 2
0.90	UMAD1	UBAP1-MVB12-associated (UMA) domain containing 1
0.90	RNASEH2C	ribonuclease H2. subunit C
0.90	LRIG1	
0.90	RBM15B	RNA binding motif protein 15B
0.90	RRBP1	ribosome binding protein 1
0.90	TAS2R16	taste receptor. type 2. member 16
0.90	ZNF812P	zinc finger protein 812. pseudogene
0.90	ADGRG5	adhesion G protein-coupled receptor G5
0.90	UBR1	ubiquitin protein ligase E3 component n-recognin 1
0.90	HN1	hematological and neurological expressed 1
0.90	C7orf72	chromosome 7 open reading frame 72
0.90	MALRD1	MAM and LDL receptor class A domain containing 1
0.90	SLCO2A1	solute carrier organic anion transporter family. member 2A1
0.90	PARVB	parvin. beta
0.90	RIMS3	regulating synaptic membrane exocytosis 3
0.90	ACP1	acid phosphatase 1. soluble
0.90	EXD2	exonuclease 3-5 domain containing 2
0.90	PDE6H	phosphodiesterase 6H. cGMP-specific. cone. gamma

0.90	CST11	cystatin 11
0.90	POLE	Transcript Identified by AceView. Entrez Gene ID(s) 5426
0.90	RAD51AP2	RAD51 associated protein 2
0.90	KIF19	kinesin family member 19
0.90	RBP1	retinol binding protein 1. cellular
0.90	P2RY13	purinergic receptor P2Y. G-protein coupled. 13
0.90	RHCE	Rh blood group. CcEe antigens
0.90	BARHL2	BarH-like homeobox 2
0.90	ADGRV1	adhesion G protein-coupled receptor V1
0.90	TRAPPC4	trafficking protein particle complex 4
0.90	ZBTB18	zinc finger and BTB domain containing 18
0.90	ROS1	ROS proto-oncogene 1 . receptor tyrosine kinase
0.90	NSUN6	NOP2/Sun domain family. member 6
0.90	EVI5	ecotropic viral integration site 5
0.90	PTPN23	protein tyrosine phosphatase. non-receptor type 23
0.90	PXDN	Transcript Identified by AceView. Entrez Gene ID(s) 7837
0.90	TRIP12	Transcript Identified by AceView. Entrez Gene ID(s) 9320
0.90	H3F3B	H3 histone. family 3B (H3.3B)
0.90	AIFM3	apoptosis-inducing factor. mitochondrion-associated. 3
0.90	CCDC62	coiled-coil domain containing 62
0.90	C15orf54	chromosome 15 open reading frame 54
0.90	BRINP2	bone morphogenetic protein/retinoic acid inducible neural-specific 2
0.90	RNASE2	ribonuclease. RNase A family. 2 (liver. eosinophil-derived neurotoxin)
0.90	RFC3	Memczak2013 ALT_ACCEPTOR. ALT_DONOR. coding. INTERNAL. intronic best transcript NM_181558
0.90	EBLN1	endogenous Bornavirus-like nucleoprotein 1

0.90	LYPD2	LY6/PLAUR domain containing 2
0.90	C9orf173	chromosome 9 open reading frame 173
0.90	ZNF525	zinc finger protein 525
0.90	P3H3	prolyl 3-hydroxylase 3
0.90	RAB11FIP1	RAB11 family interacting protein 1 (class I)
0.90	FOXP4; MIR4641	forkhead box P4; microRNA 4641
0.90	ANKRD60	ankyrin repeat domain 60
0.90	SCNN1B	sodium channel. non voltage gated 1 beta subunit
0.90	CSPP1	centrosome and spindle pole associated protein 1
0.90	TMEM234	transmembrane protein 234
0.90	TMX2; C11orf31	thioredoxin-related transmembrane protein 2; chromosome 11 open reading frame 31
0.90	LHFPL3	lipoma HMGIC fusion partner-like 3
0.90	CATSPER2	cation channel. sperm associated 2
0.90	UBAC2	UBA domain containing 2
0.90	KRT9	keratin 9. type I
0.90	LMCD1; LINC00312	LIM and cysteine-rich domains 1; long intergenic non-protein coding RNA 312
0.90	C11orf73	chromosome 11 open reading frame 73
0.90	CMTM8	CKLF-like MARVEL transmembrane domain containing 8
0.90	ZNF197	zinc finger protein 197
0.90	KRTAP5-5	keratin associated protein 5-5
0.90	ARMC4	armadillo repeat containing 4
0.90	MMP28	matrix metallopeptidase 28
0.90	APOBEC1	apolipoprotein B mRNA editing enzyme. catalytic polypeptide 1
0.90	BAP1	BRCA1 associated protein 1
0.90	TXNDC11	thioredoxin domain containing 11

0.90	HAPLN3	hyaluronan and proteoglycan link protein 3
0.90	ARHGEF9; ARHGEF9-IT1	Cdc42 guanine nucleotide exchange factor 9; ARHGEF9 intronic transcript 1
0.90	PTGIS	prostaglandin I2 (prostacyclin) synthase
0.90	MIEF1	mitochondrial elongation factor 1
0.90	PCDHB8	protocadherin beta 8
0.90	COL16A1	collagen. type XVI. alpha 1
0.90	TBC1D30	TBC1 domain family. member 30
0.90	CEACAM5	carcinoembryonic antigen-related cell adhesion molecule 5
0.90	OR9G1; OR9G9	olfactory receptor. family 9. subfamily G. member 1; olfactory receptor. family 9. subfamily G. member 9
0.90	MYO18B	myosin XVIIIB
0.90	OR5J2	olfactory receptor. family 5. subfamily J. member 2
0.90	ELMO2	engulfment and cell motility 2
0.89	VTA1	vesicle (multivesicular body) trafficking 1
0.89	EIF4G1	eukaryotic translation initiation factor 4 gamma. 1
0.89	APAF1	apoptotic peptidase activating factor 1
0.89	BCKDK	branched chain ketoacid dehydrogenase kinase
0.89	SEC23IP	SEC23 interacting protein
0.89	KLRD1	killer cell lectin-like receptor subfamily D. member 1
0.89	TCP10L	t-complex 10-like
0.89	DGKI	diacylglycerol kinase. iota
0.89	GMNC	geminin coiled-coil domain containing
0.89	KLHDC8B	kelch domain containing 8B
0.89	CHRNB4	cholinergic receptor. nicotinic beta 4
0.89	HSBP1L1	heat shock factor binding protein 1-like 1
0.89	PRAMEF18	PRAME family member 18

0.89	KREMEN2	kringle containing transmembrane protein 2
0.89	PRICKLE1	prickle homolog 1
0.89	KIAA0430	Transcript Identified by AceView. Entrez Gene ID(s) 9665
0.89	ITGB3	integrin beta 3
0.89	TFCP2	transcription factor CP2
0.89	SCNN1G	sodium channel. non voltage gated 1 gamma subunit
0.89	HSPA1B; HSPA1A	heat shock 70kDa protein 1B; heat shock 70kDa protein 1A
0.89	LOC101928877; RP11-386B13.3; SLC25A4	uncharacterized LOC101928877; novel transcript; Transcript Identified by AceView. Entrez Gene ID(s) 291
0.89	TCF20	transcription factor 20 (AR1)
0.89	TCF20	transcription factor 20 (AR1)
0.89	YAE1D1	Yae1 domain containing 1
0.89	SAMD11	sterile alpha motif domain containing 11
0.89	WDR78	WD repeat domain 78
0.89	DRD1	dopamine receptor D1
0.89	BIRC6	Transcript Identified by AceView. Entrez Gene ID(s) 57448
0.89	AWAT2	acyl-CoA wax alcohol acyltransferase 2
0.89	FGFR2	fibroblast growth factor receptor 2
0.89	AP3B1	Memczak2013 ALT_ACCEPTOR. ALT_DONOR. coding. INTERNAL. intronic best transcript NM_003664
0.89	DPP9	dipeptidyl-peptidase 9
0.89	ZSCAN10	zinc finger and SCAN domain containing 10
0.89	NFKBIL1	nuclear factor of kappa light polypeptide gene enhancer in B-cells inhibitor-like 1
0.89	ZNF888	zinc finger protein 888
0.89	DHRS7B	dehydrogenase/reductase (SDR family) member 7B
0.89	LAMC2	laminin. gamma 2

0.89	SCG3	secretogranin III
0.89	CRYGC	crystallin gamma C
0.89	COMT; MIR4761	catechol-O-methyltransferase; microRNA 4761
0.89	WDR20	WD repeat domain 20
0.89	FBXL18	F-box and leucine-rich repeat protein 18
0.89	ZNF493	zinc finger protein 493
0.89	GLB1L2	galactosidase beta 1 like 2
0.89	FGF14; FGF14-IT1	fibroblast growth factor 14; FGF14 intronic transcript 1
0.89	ASCL5	achaete-scute family bHLH transcription factor 5
0.89	ARNT	aryl hydrocarbon receptor nuclear translocator
0.89	RPN1	ribophorin I
0.89	MAGEB6	MAGE family member B6
0.89	OTOGL	otogelin-like
0.89	TXNDC5	thioredoxin domain containing 5 (endoplasmic reticulum)
0.89	PDRG1	p53 and DNA-damage regulated 1
0.89	YKT6	YKT6 v-SNARE homolog (S. cerevisiae)
0.89	ARMC12	armadillo repeat containing 12
0.89	NXF5	nuclear RNA export factor 5
0.89	GZMB	granzyme B
0.89	LOC442028; ANKRD20B	uncharacterized LOC442028; Transcript Identified by AceView. Entrez Gene ID(s) 729171
0.89	CNDP1	carnosine dipeptidase 1 (metallopeptidase M20 family)
0.89	ACKR1	atypical chemokine receptor 1 (Duffy blood group)
0.89	C1QTNF6	C1q and tumor necrosis factor related protein 6
0.89	YIPF5	Yip1 domain family member 5
0.89	BUB3	BUB3 mitotic checkpoint protein

0.89	RFXANK	regulatory factor X-associated ankyrin-containing protein
0.89	CRYBB3	crystallin beta B3
0.89	TEKT3	tektin 3
0.89	SPSB2	splA/ryanodine receptor domain and SOCS box containing 2
0.89	OR2T11	olfactory receptor. family 2. subfamily T. member 11 (gene/pseudogene)
0.89	CCDC88C	coiled-coil domain containing 88C
0.89	KIRREL3	Jeck2013 ALT_ACCEPTOR. ALT_DONOR. coding. INTERNAL. intronic best transcript NM_032531
0.89	FAM96B	family with sequence similarity 96. member B
0.89	COL4A3	collagen. type IV. alpha 3 (Goodpasture antigen)
0.89	SAG	S-antigen; retina and pineal gland (arrestin)
0.89	MYRFL	myelin regulatory factor-like
0.89	POMGNT1	protein O-linked mannose N-acetylglucosaminyltransferase 1 (beta 1.2-)
0.89	FAM231D; LINC00869	family with sequence similarity 231. member D; long intergenic non-protein coding RNA 869
0.89	NUTM2D	NUT family member 2D
0.89	LRIG1	
0.89	APOH	apolipoprotein H (beta-2-glycoprotein I)
0.89	SFPQ	splicing factor proline/glutamine-rich
0.89	CD177	CD177 molecule
0.89	RFTN1	raftlin. lipid raft linker 1
0.89	PYCR2; MIR6741	pyrroline-5-carboxylate reductase family. member 2; microRNA 6741
0.89	OR1M1	olfactory receptor. family 1. subfamily M. member 1
0.89	ATAD3C	ATPase family. AAA domain containing 3C
0.89	EXOC3L2	exocyst complex component 3-like 2
0.89	ALKBH5	alkB homolog 5. RNA demethylase
0.89	NDOR1	NADPH dependent diflavin oxidoreductase 1

0.89	UBR7	ubiquitin protein ligase E3 component n-recognin 7 (putative)
0.89	CCND3	Memczak2013 ALT_ACCEPTOR. ALT_DONOR. coding. INTERNAL. intronic best transcript NM_001136017
0.89	DNMT3A	DNA (cytosine-5-)-methyltransferase 3 alpha
0.89	CXorf40A	chromosome X open reading frame 40A
0.89	PPP1R13B	protein phosphatase 1. regulatory subunit 13B
0.89	SCRG1	stimulator of chondrogenesis 1
0.89	KCNC4	potassium channel. voltage gated Shaw related subfamily C. member 4
0.89	RHOJ	ras homolog family member J
0.89	AMZ1	archaelysin family metallopeptidase 1
0.89	HRH2	histamine receptor H2
0.89	OR4C16	olfactory receptor. family 4. subfamily C. member 16 (gene/pseudogene)
0.89	CXorf66	chromosome X open reading frame 66
0.89	ARHGEF1	Rho guanine nucleotide exchange factor 1
0.89	PNPLA6	patatin-like phospholipase domain containing 6
0.89	SPATA31D4	SPATA31 subfamily D. member 4
0.89	APOA4	apolipoprotein A-IV
0.89	PDIA6	protein disulfide isomerase family A. member 6
0.89	C6orf89	chromosome 6 open reading frame 89
0.89	C4orf33	chromosome 4 open reading frame 33
0.89	RAMP2	receptor (G protein-coupled) activity modifying protein 2
0.89	POGZ	Transcript Identified by AceView. Entrez Gene ID(s) 23126
0.89	CLK3	CDC like kinase 3
0.89	F11R	F11 receptor
0.89	TXNDC8	thioredoxin domain containing 8 (spermatozoa)
0.89	HSD17B3	hydroxysteroid (17-beta) dehydrogenase 3

0.89	RIC8A; MIR6743	RIC8 guanine nucleotide exchange factor A; microRNA 6743
0.89	CRX	cone-rod homeobox
0.89	CHRNA2	cholinergic receptor. nicotinic alpha 2
0.89	ARHGAP11B	Rho GTPase activating protein 11B
0.89	ATP5J	ATP synthase. H+ transporting. mitochondrial Fo complex subunit F6
0.89	MITD1	microtubule interacting and trafficking domain containing 1
0.89	NKPD1	NTPase. KAP family P-loop domain containing 1
0.89	ZNF212	zinc finger protein 212
0.89	SIGLECL1	SIGLEC family like 1
0.89	GS1-259H13.2; TCONS_l2_00026125; TCONS_l2_00026126; ZNF655	transmembrane protein 225-like; Jeck2013 ALT_ACCEPTOR. ALT_DONOR. ncRNA. OVCODE. OVEXON. upstream_start best transcript TCONS_l2_00026125; Salzman2013 ANNOTATED. INTERNAL. ncRNA. OVCODE. OVEXON best transcript TCONS_l2_00026126; novel transcript; Transcript Identified by AceView. Entrez Gene ID(s) 79027
0.89	RNASE9	ribonuclease. RNase A family. 9 (non-active)
0.89	OR3A3	olfactory receptor. family 3. subfamily A. member 3
0.89	LOXHD1	lipoxygenase homology domains 1
0.89	ZSCAN31	zinc finger and SCAN domain containing 31
0.89	NNT	nicotinamide nucleotide transhydrogenase
0.89	FGFR1OP	FGFR1 oncogene partner
0.89	NR4A2	nuclear receptor subfamily 4. group A. member 2
0.89	HAVCR1	hepatitis A virus cellular receptor 1
0.89	ZNF667	zinc finger protein 667
0.89	IFNA17	interferon. alpha 17
0.89	ATAD2	ATPase family. AAA domain containing 2
0.89	WRN	Werner syndrome. RecQ helicase-like
0.89	CCDC97	coiled-coil domain containing 97

0.89	TJP2	tight junction protein 2
0.89	OR4D1	olfactory receptor. family 4. subfamily D. member 1
0.89	DNAJB11	DnaJ (Hsp40) homolog. subfamily B. member 11
0.89	TNFSF14	tumor necrosis factor (ligand) superfamily. member 14
0.89	HHAT	hedgehog acyltransferase
0.89	BPY2B	basic charge. Y-linked. 2B
0.89	ADGRE2	adhesion G protein-coupled receptor E2
0.89	ANXA8	annexin A8
0.89	EMC9	ER membrane protein complex subunit 9
0.89	NKIRAS1	NFKB inhibitor interacting Ras-like 1
0.89	RORC	RAR-related orphan receptor C
0.89	CAPN1	calpain 1. (mu/I) large subunit
0.89	IGF2R	insulin-like growth factor 2 receptor
0.89	FAM81B	family with sequence similarity 81. member B
0.89	ROBO2	roundabout guidance receptor 2
0.89	FZD7	frizzled class receptor 7
0.89	NSUN5P2; NSUN5P1	NOP2/Sun domain family. member 5 pseudogene 2; NOP2/Sun domain family. member 5 pseudogene 1
0.89	MTHFD1	methylenetetrahydrofolate dehydrogenase (NADP+ dependent) 1. methenyltetrahydrofolate cyclohydrolase. formyltetrahydrofolate synthetase
0.89	GAB1	GRB2-associated binding protein 1
0.89	ECHDC2	enoyl-CoA hydratase domain containing 2
0.89	HKR1	HKR1. GLI-Kruppel zinc finger family member
0.89	OR2M5	olfactory receptor. family 2. subfamily M. member 5
0.89	CSNK1A1L	casein kinase 1. alpha 1-like
0.89	HOXD11	homeobox D11

0.89	SYT15	synaptotagmin XV
0.89	OR52B4	olfactory receptor. family 52. subfamily B. member 4 (gene/pseudogene)
0.89	TMCC1	transmembrane and coiled-coil domain family 1
0.89	INSM1	insulinoma-associated 1
0.89	MBL1P; BMS1P21	mannose-binding lectin (protein A) 1. pseudogene; BMS1 ribosome biogenesis factor pseudogene 21
0.89	MRPS11	mitochondrial ribosomal protein S11
0.89	RPL10L	ribosomal protein L10-like
0.89	MAGEB2	MAGE family member B2
0.89	BTBD8	BTB (POZ) domain containing 8
0.89	CSTF1	cleavage stimulation factor. 3 pre-RNA. subunit 1
0.89	DEFB132	defensin. beta 132
0.89	DISP2	dispatched homolog 2 (Drosophila)
0.89	ALDH4A1	aldehyde dehydrogenase 4 family. member A1
0.89	DACT2	dishevelled-binding antagonist of beta-catenin 2
0.89	HS3ST2	heparan sulfate (glucosamine) 3-O-sulfotransferase 2
0.89	LRRFIP2	leucine rich repeat (in FLII) interacting protein 2
0.89	TMEM89	transmembrane protein 89
0.89	TTC30A	tetratricopeptide repeat domain 30A
0.89	CEP128	centrosomal protein 128kDa
0.89	ARMCX1	armadillo repeat containing. X-linked 1
0.89	SIPA1L2	signal-induced proliferation-associated 1 like 2
0.89	ZNF805	zinc finger protein 805
0.89	UGP2	UDP-glucose pyrophosphorylase 2
0.89	SLC10A7	solute carrier family 10. member 7
0.89	TNNT3	troponin T type 3 (skeletal. fast)

0.89	SLC15A2	solute carrier family 15 (oligopeptide transporter). member 2
0.89	PRPF40B	PRP40 homolog. pre-mRNA processing factor B
0.89	CHPF2; MIR671	chondroitin polymerizing factor 2; microRNA 671
0.89	TRIM42	tripartite motif containing 42
0.89	ADRA2B	adrenoceptor alpha 2B
0.89	PRR18	proline rich 18
0.89	ZCWPW1	zinc finger. CW type with PWWP domain 1
0.89	SLC30A1	solute carrier family 30 (zinc transporter). member 1
0.89	SLC34A1	solute carrier family 34 (type II sodium/phosphate cotransporter). member 1
0.89	C16orf59	chromosome 16 open reading frame 59
0.89	C20orf195	chromosome 20 open reading frame 195
0.89	CC2D2B	coiled-coil and C2 domain containing 2B
0.89	ROBO1	roundabout guidance receptor 1
0.89	PRRT4	proline-rich transmembrane protein 4
0.89	CNTNAP5	contactin associated protein-like 5
0.89	RANBP2	RAN binding protein 2
0.89	TRAK2	Jeck2013 ALT_ACCEPTOR. ALT_DONOR. coding. INTERNAL. intronic best transcript NM_015049
0.89	KCNJ13	potassium channel. inwardly rectifying subfamily J. member 13
0.89	CCP110	centriolar coiled coil protein 110kDa
0.89	HYOU1	hypoxia up-regulated 1
0.89	CYP11B2	cytochrome P450. family 11. subfamily B. polypeptide 2
0.89	CD8B	CD8b molecule
0.89	HSP90B1; MIR3652	heat shock protein 90kDa beta (Grp94). member 1; microRNA 3652
0.89	TXNRD3NB; TXNRD3	thioredoxin reductase 3 neighbor; thioredoxin reductase 3
0.89	PSMD8	proteasome 26S subunit. non-ATPase 8

0.89	FGF23	fibroblast growth factor 23
0.89	CD320	CD320 molecule
0.89	LINC00663	long intergenic non-protein coding RNA 663
0.89	CHIA	chitinase. acidic
0.89	PDE6A	phosphodiesterase 6A. cGMP-specific. rod. alpha
0.89	PLPP5	phospholipid phosphatase 5
0.89	ADRM1	adhesion regulating molecule 1
0.89	PRKX	protein kinase. X-linked
0.89	WNT5B	wingless-type MMTV integration site family. member 5B
0.89	DRAXIN	dorsal inhibitory axon guidance protein
0.89	PFN4	profilin family. member 4
0.89	EFCAB13	EF-hand calcium binding domain 13
0.89	PADI4	peptidyl arginine deiminase. type IV
0.89	NEMP2	nuclear envelope integral membrane protein 2
0.89	DPEP3	dipeptidase 3
0.89	MSN	moesin
0.89	RETNLB	resistin like beta
0.89	CEP112	centrosomal protein 112kDa
0.89	CACNA2D3	calcium channel. voltage-dependent. alpha 2/delta subunit 3
0.89	AHDC1	AT hook. DNA binding motif. containing 1
0.89	NPAS4	neuronal PAS domain protein 4
0.89	C9orf152	chromosome 9 open reading frame 152
0.89	SOBP	sine oculis binding protein homolog
0.89	DPP9-AS1	DPP9 antisense RNA 1
0.89	USP19	ubiquitin specific peptidase 19
0.89	UBAP1L	ubiquitin associated protein 1 like

0.89	CDK5	cyclin-dependent kinase 5
0.89	GABPA	GA binding protein transcription factor alpha subunit
0.89	IL2RB	interleukin 2 receptor. beta
0.89	MMS19	MMS19 homolog. cytosolic iron-sulfur assembly component
0.89	HHAT	hedgehog acyltransferase
0.89	TSNARE1	t-SNARE domain containing 1
0.89	CYSLTR1	cysteinyl leukotriene receptor 1
0.89	DOCK1	dedicator of cytokinesis 1
0.89	WDR83	WD repeat domain 83
0.89	ZNF441	zinc finger protein 441
0.89	PODXL2	podocalyxin-like 2
0.89	WWTR1	WW domain containing transcription regulator 1
0.89	TMEM213	transmembrane protein 213
0.89	ATP6V1E1	ATPase. H+ transporting. lysosomal 31kDa. V1 subunit E1
0.89	GOLGA6A	golgin A6 family. member A
0.89	WNT9A	wingless-type MMTV integration site family. member 9A
0.89	KIF26A	kinesin family member 26A
0.89	RFX2	regulatory factor X. 2 (influences HLA class II expression)
0.89	SLC6A7	solute carrier family 6 (neurotransmitter transporter). member 7
0.89	MZT2B	mitotic spindle organizing protein 2B
0.89	FMO3	flavin containing monooxygenase 3
0.89	MMD2	monocyte to macrophage differentiation-associated 2
0.89	ZNF479	zinc finger protein 479
0.89	CDHR2	cadherin-related family member 2
0.89	ACVR1C	activin A receptor type IC
0.89	ZNF664	zinc finger protein 664

0.89	ACPT	acid phosphatase. testicular
0.89	LIX1L	limb and CNS expressed 1 like
0.89	SP9	Sp9 transcription factor
0.89	RPL14	Transcript Identified by AceView. Entrez Gene ID(s) 9045
0.89	HIST1H2BE	Memczak2013 ANTISENSE. CDS. coding. upstream_start. UTR3. UTR5 best transcript NM_003523
0.89	ZNF646	Memczak2013 ANTISENSE. coding. INTERNAL. intronic best transcript NM_014699
0.89	OR6C70	olfactory receptor. family 6. subfamily C. member 70
0.89	HMHA1	histocompatibility (minor) HA-1
0.89	IL36B	interleukin 36. beta
0.89	CCDC88B	coiled-coil domain containing 88B
0.89	ARHGAP19-SLIT1	ARHGAP19-SLIT1 readthrough (NMD candidate)
0.89	IL1RL1	interleukin 1 receptor-like 1
0.89	DAZ3	deleted in azoospermia 3
0.89	ADRB1	adrenoceptor beta 1
0.89	MARVELD3	MARVEL domain containing 3
0.89	NME8	NME/NM23 family member 8
0.89	PRH2	proline-rich protein HaeIII subfamily 2
0.89	TMEM184B	transmembrane protein 184B
0.89	FAM19A3	family with sequence similarity 19 (chemokine (C-C motif)-like). member A3
0.89	RBM12; CPNE1	RNA binding motif protein 12; copine I
0.89	SIRPB2	signal-regulatory protein beta 2
0.89	SPTSSA	serine palmitoyltransferase. small subunit A
0.89	LCE3D	late cornified envelope 3D
0.89	COL8A1	collagen. type VIII. alpha 1
0.89	CCKBR	cholecystokinin B receptor

0.89	FCN2	ficolin (collagen/fibrinogen domain containing lectin) 2
0.89	ZNF776	zinc finger protein 776
0.89	CCDC84	coiled-coil domain containing 84
0.89	ND5	NADH dehydrogenase. subunit 5 (complex I)
0.89	ANGPT4	angiopoietin 4
0.89	PRAMEF16; PRAMEF17	PRAME family member 16; PRAME family member 17
0.89	LSM6	LSM6 homolog. U6 small nuclear RNA and mRNA degradation associated
0.89	IL34	interleukin 34
0.89	BARX1	BARX homeobox 1
0.89	TLK2	tousled-like kinase 2
0.89	LRFN3	leucine rich repeat and fibronectin type III domain containing 3
0.89	RAB36	RAB36. member RAS oncogene family
0.89	DCDC2C	doublecortin domain containing 2C
0.89	CDKAL1	CDK5 regulatory subunit associated protein 1-like 1
0.89	PTPN1	protein tyrosine phosphatase. non-receptor type 1
0.89	SPARC	secreted protein. acidic. cysteine-rich (osteonectin)
0.89	TRDMT1	tRNA aspartic acid methyltransferase 1
0.89	ASB15	ankyrin repeat and SOCS box containing 15
0.89	CASD1	CAS1 domain containing 1
0.89	MGAT1	mannosyl (alpha-1.3-)-glycoprotein beta-1.2-N-acetylglucosaminyltransferase
0.89	SMIM10L1	small integral membrane protein 10 like 1
0.89	RFC1	replication factor C subunit 1
0.89	PSMA2	proteasome subunit alpha 2
0.89	ZNF70	zinc finger protein 70
0.89	ATP6V1F	ATPase. H+ transporting. lysosomal 14kDa. V1 subunit F

0.89	CACNA2D1	calcium channel. voltage-dependent. alpha 2/delta subunit 1
0.89	CMTR1	cap methyltransferase 1
0.89	EIF3F	Eukaryotic translation initiation factor 3 subunit F [Source:UniProtKB/Swiss-Prot;Acc:O00303]
0.89	APOBEC2	apolipoprotein B mRNA editing enzyme. catalytic polypeptide-like 2
0.89	TOB1	transducer of ERBB2. 1
0.89	TCEAL4	transcription elongation factor A (SII)-like 4
0.89	PHPT1	phosphohistidine phosphatase 1
0.89	NDUFV3	NADH dehydrogenase (ubiquinone) flavoprotein 3. 10kDa
0.89	SCO1	SCO1 cytochrome c oxidase assembly protein
0.89	CCDC186; MIR2110	coiled-coil domain containing 186; microRNA 2110
0.89	DBF4B	DBF4 zinc finger B
0.89	GRIK1-AS2; BACH1	GRIK1 antisense RNA 2; BTB and CNC homology 1. basic leucine zipper transcription factor 1
0.89	TRAPPC9	trafficking protein particle complex 9
0.89	ZNF780B	zinc finger protein 780B
0.89	ZNF160	Transcript Identified by AceView. Entrez Gene ID(s) 90338
0.89	MAMSTR	MEF2 activating motif and SAP domain containing transcriptional regulator
0.89	FGFBP3	fibroblast growth factor binding protein 3
0.89	PHB2; SCARNA12	prohibitin 2; small Cajal body-specific RNA 12
0.89	SAG	S-antigen; retina and pineal gland (arrestin)
0.89	OR11A1	olfactory receptor. family 11. subfamily A. member 1
0.89	ADCK4	aarF domain containing kinase 4
0.89	BHLHE23	basic helix-loop-helix family. member e23
0.89	CENPP	centromere protein P
0.89	FAM9C	family with sequence similarity 9. member C
0.89	IL1RL2	interleukin 1 receptor-like 2

0.89	NANS	N-acetylneuraminic acid synthase
0.89	APPL1	adaptor protein. phosphotyrosine interaction. PH domain and leucine zipper containing 1
0.89	PML	promyelocytic leukemia
0.89	WDR76	WD repeat domain 76
0.89	NLRP11	NLR family. pyrin domain containing 11
0.89	NXPE3	neurexophilin and PC-esterase domain family. member 3
0.89	ROPN1L	rhophilin associated tail protein 1-like
0.89	GPR18	G protein-coupled receptor 18
0.89	ASPG	asparaginase
0.89	NFKBID	nuclear factor of kappa light polypeptide gene enhancer in B-cells inhibitor. delta
0.89	UCP1	uncoupling protein 1 (mitochondrial. proton carrier)
0.89	KRTAP23-1	keratin associated protein 23-1
0.89	KRTAP6-2	keratin associated protein 6-2
0.89	DLGAP1	discs. large (Drosophila) homolog-associated protein 1
0.89	SLC43A2	solute carrier family 43 (amino acid system L transporter). member 2
0.89	TDRD15	tudor domain containing 15
0.89	CTAGE15	CTAGE family. member 15
0.89	TMPRSS11A	transmembrane protease. serine 11A
0.89	REC8	REC8 meiotic recombination protein
0.89	ACAA2	acetyl-CoA acyltransferase 2
0.89	LRRC29	leucine rich repeat containing 29
0.89	VAMP1	vesicle associated membrane protein 1
0.89	MSI1	musashi RNA binding protein 1
0.89	PIWIL2	piwi-like RNA-mediated gene silencing 2
0.89	SIGLEC10	sialic acid binding Ig-like lectin 10

0.89	FAM107B	Memczak2013 ALT_ACCEPTOR. ALT_DONOR. coding. INTERNAL. intronic best transcript NM_031453
0.89	ENDOU	endonuclease. polyU-specific
0.89	ZNF512B	zinc finger protein 512B
0.89	LTA	lymphotoxin alpha
0.89	CPNE5	copine V
0.89	DDA1	DET1 and DDB1 associated 1
0.89	RNASE1	ribonuclease. RNase A family. 1 (pancreatic)
0.89	ARL13A	ADP-ribosylation factor like GTPase 13A
0.89	BPIFB2	BPI fold containing family B. member 2
0.89	ZNF140	zinc finger protein 140
0.89	ABCD3	Transcript Identified by AceView. Entrez Gene ID(s) 5825
0.89	PPT2; EGFL8; PPT2-EGFL8	palmitoyl-protein thioesterase 2; EGF-like-domain. multiple 8; PPT2-EGFL8 readthrough (NMD candidate)
0.89	COL10A1	collagen. type X. alpha 1
0.89	ADAMTS13	ADAM metallopeptidase with thrombospondin type 1 motif 13
0.89	GOLGA2	golgin A2
0.89	FBXL7	F-box and leucine-rich repeat protein 7
0.89	SVOP	SV2 related protein
0.89	THPO	thrombopoietin
0.89	CDC7	cell division cycle 7
0.89	DRD5	dopamine receptor D5
0.89	SUCLA2	succinate-CoA ligase. ADP-forming. beta subunit
0.89	LAMP1	lysosomal-associated membrane protein 1
0.89	USP33	ubiquitin specific peptidase 33
0.89	FAM182B	family with sequence similarity 182. member B
0.89	GLP1R	glucagon-like peptide 1 receptor

0.89	ASB14	ankyrin repeat and SOCS box containing 14
0.89	PTCRA	pre T-cell antigen receptor alpha
0.89	SMPD4	sphingomyelin phosphodiesterase 4. neutral membrane (neutral sphingomyelinase-3)
0.89	PDE6B	phosphodiesterase 6B. cGMP-specific. rod. beta
0.89	HIST4H4	histone cluster 4. H4
0.89	ZNF394	zinc finger protein 394
0.89	SALL1	spalt-like transcription factor 1
0.89	PCLO	piccolo presynaptic cytomatrix protein
0.89	GAS2	growth arrest-specific 2
0.89	UBXN11	UBX domain protein 11
0.89	CLVS1	clavesin 1
0.89	SASH3	SAM and SH3 domain containing 3
0.89	HAUS1	HAUS augmin like complex subunit 1
0.89	PLEKHG5	pleckstrin homology domain containing. family G (with RhoGef domain) member 5
0.89	CLDN14	claudin 14
0.89	FBXO27	F-box protein 27
0.89	SFT2D3	SFT2 domain containing 3
0.89	SOX15	SRY box 15
0.89	MESP1	mesoderm posterior bHLH transcription factor 1
0.89	MYB-AS1; MYB; RP1-32B1.3; rawjer	MYB antisense RNA 1 [Source:HGNC Symbol;Acc:HGNC:37457]; Memczak2013 ANTISENSE. coding. INTERNAL. intronic best transcript NM_005375; putative novel transcript; Transcript Identified by AceView
0.89	PYCARD	PYD and CARD domain containing
0.89	AKAP2	A kinase (PRKA) anchor protein 2
0.89	BPIFA1	BPI fold containing family A. member 1

0.89	IVD	isovaleryl-CoA dehydrogenase
0.89	SPDEF	SAM pointed domain containing ETS transcription factor
0.89	ERMARD	ER membrane-associated RNA degradation
0.89	PM20D1	peptidase M20 domain containing 1
0.89	LSM2	LSM2 homolog. U6 small nuclear RNA and mRNA degradation associated
0.89	ODF3	outer dense fiber of sperm tails 3
0.89	OR5K1	olfactory receptor. family 5. subfamily K. member 1
0.89	ST20-AS1	ST20 antisense RNA 1
0.89	PINK1; MIR6084	PTEN induced putative kinase 1; microRNA 6084
0.89	OR6T1	olfactory receptor. family 6. subfamily T. member 1
0.89	CRYGA	crystallin gamma A
0.89	GNPNAT1	glucosamine-phosphate N-acetyltransferase 1
0.89	OR4X1	olfactory receptor. family 4. subfamily X. member 1 (gene/pseudogene)
0.89	MAP3K11	mitogen-activated protein kinase kinase kinase 11
0.89	OR1P1	olfactory receptor. family 1. subfamily P. member 1 (gene/pseudogene)
0.89	TBX2	T-box 2
0.89	FAAH	fatty acid amide hydrolase
0.89	SEC61G	Sec61 translocon gamma subunit
0.89	TRAPPC5	trafficking protein particle complex 5
0.89	STRIP2	striatin interacting protein 2
0.89	KRTAP5-2	keratin associated protein 5-2
0.89	ALG10B	ALG10B. alpha-1.2-glucosyltransferase
0.89	SUMO4	small ubiquitin-like modifier 4
0.89	SLC1A7	solute carrier family 1 (glutamate transporter). member 7
0.89	NAT16	N-acetyltransferase 16 (GCN5-related. putative)
0.89	ACOXL	acyl-CoA oxidase-like

0.89	PTPRK	protein tyrosine phosphatase. receptor type. K
0.89	TLR6	toll-like receptor 6
0.89	TGFA	transforming growth factor alpha
0.89	AKR7L	aldo-keto reductase family 7-like (gene/pseudogene)
0.89	WDR59	WD repeat domain 59
0.89	TYR	tyrosinase
0.89	SNED1	sushi. nidogen and EGF-like domains 1
0.89	WNT9B	wingless-type MMTV integration site family. member 9B
0.89	LINC00518	long intergenic non-protein coding RNA 518
0.89	HTRA4	HtrA serine peptidase 4
0.89	LIMA1	LIM domain and actin binding 1
0.89	SH3GL2	SH3-domain GRB2-like 2
0.89	RP11-466A19.5; MYO1D	Transcript Identified by AceView. Entrez Gene ID(s) 4642; novel transcript. sense intronic MYO1D
0.89	RAB28	Jeck2013 ALT_ACCEPTOR. ALT_DONOR. coding. INTERNAL. intronic best transcript NM_004249
0.89	DYRK1B	dual specificity tyrosine-(Y)-phosphorylation regulated kinase 1B
0.89	TSSK3	testis-specific serine kinase 3
0.89	ZNF217	zinc finger protein 217
0.89	CNOT2	CCR4-NOT transcription complex subunit 2
0.89	GLRA4	glycine receptor alpha 4
0.89	APTX	aprataxin
0.89	GATA2	GATA binding protein 2
0.89	ODF3L1	outer dense fiber of sperm tails 3-like 1
0.89	UBXN4	UBX domain protein 4
0.89	NDUFAF3	NADH dehydrogenase (ubiquinone) complex I. assembly factor 3
0.89	OR13D1	olfactory receptor. family 13. subfamily D. member 1

0.88	PDIA5; MIR7110	protein disulfide isomerase family A. member 5; microRNA 7110
0.88	SMPDL3B	sphingomyelin phosphodiesterase. acid-like 3B
0.88	PTPRCAP	protein tyrosine phosphatase. receptor type. C-associated protein
0.88	SCFD2	sec1 family domain containing 2
0.88	LRTM2	leucine-rich repeats and transmembrane domains 2
0.88	HABP4	hyaluronan binding protein 4
0.88	TRIM49C	tripartite motif containing 49C
0.88	CHRM3	cholinergic receptor. muscarinic 3
0.88	CTRC	chymotrypsin C (caldecrin)
0.88	DNAJB6	DnaJ (Hsp40) homolog. subfamily B. member 6
0.88	RHBDF1	rhomboid 5 homolog 1 (Drosophila)
0.88	G3BP1	GTPase activating protein (SH3 domain) binding protein 1
0.88	TAS2R20	taste receptor. type 2. member 20
0.88	CENPT	centromere protein T
0.88	RAD21; MIR3610	RAD21 cohesin complex component; microRNA 3610
0.88	AP1M2	adaptor-related protein complex 1. mu 2 subunit
0.88	PENK	proenkephalin
0.88	UBA1; tydee	Memczak2013 ANTISENSE. CDS. coding. INTERNAL best transcript NM_153280; Transcript Identified by AceView
0.88	ILDR1	immunoglobulin-like domain containing receptor 1
0.88	USP14	ubiquitin specific peptidase 14 (tRNA-guanine transglycosylase)
0.88	ANKRD44	Memczak2013 ANTISENSE. coding. INTERNAL. intronic best transcript NM_001195144
0.88	MYO1E	Transcript Identified by AceView. Entrez Gene ID(s) 4643
0.88	TADA3	transcriptional adaptor 3
0.88	CBLN4	cerebellin 4 precursor
0.88	ELOVL3	ELOVL fatty acid elongase 3

0.88	PDLIM2	PDZ and LIM domain 2 (mystique)
0.88	ZKSCAN7	zinc finger with KRAB and SCAN domains 7
0.88	OR5R1	olfactory receptor. family 5. subfamily R. member 1 (gene/pseudogene)
0.88	COL19A1	collagen. type XIX. alpha 1
0.88	ABHD16B	abhydrolase domain containing 16B
0.88	CYBA	cytochrome b-245. alpha polypeptide
0.88	GIT2	G protein-coupled receptor kinase interacting ArfGAP 2
0.88	WNT3	wingless-type MMTV integration site family. member 3
0.88	LPCAT1	Memczak2013 ANTISENSE. coding. INTERNAL. intronic best transcript NM_024830
0.88	SRRM5	serine/arginine repetitive matrix 5
0.88	EGFL7	EGF-like-domain. multiple 7
0.88	PSMC3	proteasome 26S subunit. ATPase 3
0.88	PC	Memczak2013 ALT_ACCEPTOR. ALT_DONOR. coding. INTERNAL. intronic best transcript NM_022172
0.88	TMEM94; MIR6785	transmembrane protein 94; microRNA 6785
0.88	UNKL	unkempt family zinc finger-like
0.88	KRTAP10-11; KRTAP12-3; KRTAP10-12	keratin associated protein 10-11; keratin associated protein 12-3; keratin associated protein 10-12
0.88	TDGF1	teratocarcinoma-derived growth factor 1
0.88	CHRM3	cholinergic receptor. muscarinic 3
0.88	NAT8	N-acetyltransferase 8 (GCN5-related. putative)
0.88	SAR1A	secretion associated. Ras related GTPase 1A
0.88	PRR5-ARHGAP8	PRR5-ARHGAP8 readthrough
0.88	BTNL3	butyrophilin-like 3
0.88	PCSK6	proprotein convertase subtilisin/kexin type 6
0.88	ABHD2	abhydrolase domain containing 2

0.88	GAGE12C; GAGE12E; GAGE12H	G antigen 12C; G antigen 12E; G antigen 12H
0.88	WDR25	WD repeat domain 25
0.88	SCFD1	sec1 family domain containing 1
0.88	ABO	ABO blood group (transferase A. alpha 1-3-N-acetylgalactosaminyltransferase; transferase B. alpha 1-3-galactosyltransferase)
0.88	C7orf66	chromosome 7 open reading frame 66
0.88	PADI3	peptidyl arginine deiminase. type III
0.88	PGAP2	post-GPI attachment to proteins 2
0.88	TYW1	tRNA-yW synthesizing protein 1 homolog (S. cerevisiae)
0.88	ATP6V1G2-DDX39B	ATP6V1G2-DDX39B readthrough (NMD candidate)
0.88	WIZ	widely interspaced zinc finger motifs
0.88	WHSC1	Wolf-Hirschhorn syndrome candidate 1
0.88	NMUR1	neuromedin U receptor 1
0.88	KPNA3	Transcript Identified by AceView. Entrez Gene ID(s) 3839
0.88	KRTAP1-5	keratin associated protein 1-5
0.88	AFAP1	actin filament associated protein 1
0.88	SYT14	synaptotagmin XIV
0.88	TPRKB	TP53RK binding protein
0.88	PQLC3	PQ loop repeat containing 3
0.88	ERVV-2	endogenous retrovirus group V. member 2
0.88	HMBOX1	homeobox containing 1
0.88	FAM153B	family with sequence similarity 153. member B
0.88	ZNF618	Zhang2013 ALT_ACCEPTOR. ALT_DONOR. coding. INTERNAL. intronic best transcript NM_133374
0.88	ACHE	acetylcholinesterase (Yt blood group)
0.88	DAD1	defender against cell death 1

0.88	DGKD	diacylglycerol kinase. delta 130kDa
0.88	ARPC4	actin related protein 2/3 complex subunit 4
0.88	HP1BP3	heterochromatin protein 1. binding protein 3
0.88	NEURL1	neuralized E3 ubiquitin protein ligase 1
0.88	S1PR1	sphingosine-1-phosphate receptor 1
0.88	GNG11	guanine nucleotide binding protein (G protein). gamma 11
0.88	SERP1	stress-associated endoplasmic reticulum protein 1
0.88	GJA3	gap junction protein alpha 3
0.88	SKOR1	SKI family transcriptional corepressor 1
0.88	THRA	thyroid hormone receptor. alpha
0.88	C19orf52	chromosome 19 open reading frame 52
0.88	TMPRSS13	transmembrane protease. serine 13
0.88	GPC6	glypican 6
0.88	CYP4F22	cytochrome P450. family 4. subfamily F. polypeptide 22
0.88	RD3	retinal degeneration 3
0.88	TRIB2	tribbles pseudokinase 2
0.88	EZH2	enhancer of zeste 2 polycomb repressive complex 2 subunit
0.88	EYA3	EYA transcriptional coactivator and phosphatase 3
0.88	LINGO2	leucine rich repeat and Ig domain containing 2
0.88	SRP9	signal recognition particle 9kDa
0.88	WWC2-AS2	WWC2 antisense RNA 2
0.88	TXNDC2	thioredoxin domain containing 2 (spermatozoa)
0.88	PPP2R1B	protein phosphatase 2. regulatory subunit A. beta
0.88	LOC100129307	putative UPF0607 protein ENSP00000383144
0.88	TRAM1L1	translocation associated membrane protein 1-like 1
0.88	ZNF324	zinc finger protein 324

0.88	FAM215A	family with sequence similarity 215. member A (non-protein coding)
0.88	SIM2	single-minded family bHLH transcription factor 2
0.88	PANX2	pannexin 2
0.88	GPR108; MIR6791	G protein-coupled receptor 108; microRNA 6791
0.88	SLC45A1	solute carrier family 45. member 1
0.88	MYOG	myogenin (myogenic factor 4)
0.88	ISG20	interferon stimulated exonuclease gene 20kDa
0.88	BMPR1B	bone morphogenetic protein receptor type IB
0.88	OR2M3	olfactory receptor. family 2. subfamily M. member 3
0.88	GUCY2C	guanylate cyclase 2C
0.88	NPSR1	neuropeptide S receptor 1
0.88	NBPF9	neuroblastoma breakpoint family. member 9
0.88	RPL3L	ribosomal protein L3-like
0.88	CCDC121	coiled-coil domain containing 121
0.88	HOXB9	homeobox B9
0.88	UBE2V2	ubiquitin conjugating enzyme E2 variant 2
0.88	HTT	huntingtin
0.88	SLC17A4	solute carrier family 17. member 4
0.88	NMUR2	neuromedin U receptor 2
0.88	ZNF536	zinc finger protein 536
0.88	SYVN1	synovial apoptosis inhibitor 1. synoviolin
0.88	MS4A1	membrane-spanning 4-domains. subfamily A. member 1
0.88	CALHM1	calcium homeostasis modulator 1
0.88	EHMT2	euchromatic histone-lysine N-methyltransferase 2
0.88	PLEKHB1	pleckstrin homology domain containing. family B (evectins) member 1
0.88	MICAL2	microtubule associated monooxygenase. calponin and LIM domain

		containing 2
0.88	SLC25A11	solute carrier family 25 (mitochondrial carrier; oxoglutarate carrier). member 11
0.88	CKMT1B; CKMT1A	creatine kinase. mitochondrial 1B; creatine kinase. mitochondrial 1A
0.88	OBP2B	odorant binding protein 2B
0.88	TBX19	T-box 19
0.88	LY6G5C	lymphocyte antigen 6 complex. locus G5C
0.88	CD5L	CD5 molecule-like
0.88	FAM153B	family with sequence similarity 153. member B
0.88	KDM4C	lysine (K)-specific demethylase 4C
0.88	FTSJ3	FtsJ homolog 3 (E. coli)
0.88	VPREB1	pre-B lymphocyte 1
0.88	SYCE2	synaptonemal complex central element protein 2
0.88	ND4L; ND4	NADH dehydrogenase. subunit 4L (complex I); NADH dehydrogenase. subunit 4 (complex I)
0.88	NFE4	nuclear factor. erythroid 4
0.88	WWC2	WW and C2 domain containing 2
0.88	CEP76	centrosomal protein 76kDa
0.88	TPSD1	tryptase delta 1
0.88	SLC12A4	solute carrier family 12 (potassium/chloride transporter). member 4
0.88	ANKRD13A	ankyrin repeat domain 13A
0.88	C6orf25	chromosome 6 open reading frame 25
0.88	LRRC1	leucine rich repeat containing 1
0.88	OR6B2	olfactory receptor. family 6. subfamily B. member 2
0.88	MRPS24	mitochondrial ribosomal protein S24
0.88	SSX4B	synovial sarcoma. X breakpoint 4B
0.88	JMJD4	jumonji domain containing 4

0.88	TUBA4A	tubulin. alpha 4a
0.88	RHOV	ras homolog family member V
0.88	PFN1	profilin 1
0.88	DCAF4L1	DDB1 and CUL4 associated factor 4-like 1
0.88	TRIM68	tripartite motif containing 68
0.88	METTL14	methyltransferase like 14
0.88	RAB5C	RAB5C. member RAS oncogene family
0.88	LPA	lipoprotein. Lp(a)
0.88	TLX1NB	TLX1 neighbor
0.88	ZNF765	zinc finger protein 765
0.88	LALBA	lactalbumin. alpha-
0.88	RYR2	ryanodine receptor 2 (cardiac)
0.88	RP11-484D2.4; TTC17	novel transcript. sense overlapping TTC17; Transcript Identified by AceView. Entrez Gene ID(s) 55761
0.88	SPRR1B	small proline-rich protein 1B
0.88	GTF2I	general transcription factor IIi
0.88	NUDCD3	NudC domain containing 3
0.88	IL36RN	interleukin 36 receptor antagonist
0.88	ZNF32-AS3	ZNF32 antisense RNA 3
0.88	NRGN	neurogranin (protein kinase C substrate. RC3)
0.88	RPS15	Memczak2013 ANTISENSE. coding. INTERNAL. intronic best transcript NM_001018
0.88	ARMC6	armadillo repeat containing 6
0.88	ACTL7B	actin-like 7B
0.88	PPP2R5D	protein phosphatase 2. regulatory subunit B. delta
0.88	SLIT1; ARHGAP19-SLIT1	slit guidance ligand 1; ARHGAP19-SLIT1 readthrough (NMD candidate)
0.88	FGR	FGR proto-oncogene. Src family tyrosine kinase

0.88	SLC16A14	solute carrier family 16. member 14
0.88	ZNF436-AS1	ZNF436 antisense RNA 1
0.88	HHIP	hedgehog interacting protein
0.88	SPINK1	serine peptidase inhibitor. Kazal type 1
0.88	AP1M1	adaptor-related protein complex 1. mu 1 subunit
0.88	MED31	mediator complex subunit 31
0.88	PPIB	peptidylprolyl isomerase B (cyclophilin B)
0.88	IL11RA	interleukin 11 receptor. alpha
0.88	SDF4	stromal cell derived factor 4
0.88	SERPINA1	serpin peptidase inhibitor. clade A (alpha-1 antiproteinase. antitrypsin). member 1
0.88	UVRAG	Memczak2013 ALT_ACCEPTOR. ALT_DONOR. coding. INTERNAL. intronic best transcript NM_003369
0.88	FAM71D	family with sequence similarity 71. member D
0.88	INTS1	integrator complex subunit 1
0.88	NFIX	nuclear factor I/X (CCAAT-binding transcription factor)
0.88	RLN2	relaxin 2
0.88	F11-AS1	F11 antisense RNA 1
0.88	AIM1L	absent in melanoma 1-like
0.88	CD200	CD200 molecule
0.88	FCHO1	Memczak2013 ANTISENSE. coding. intronic. upstream_start. UTR5 best transcript NM_001161357
0.88	CCDC28A	coiled-coil domain containing 28A
0.88	NRN1L	neuritin 1-like
0.88	RPS6KA1	ribosomal protein S6 kinase. 90kDa. polypeptide 1
0.88	VAPA	Memczak2013 ALT_ACCEPTOR. ALT_DONOR. coding. INTERNAL. intronic best transcript NM_003574

0.88	YLPM1	YLP motif containing 1
0.88	LYPD4	LY6/PLAUR domain containing 4
0.88	LRRC37B	leucine rich repeat containing 37B
0.88	REXO1L10P	REX1. RNA exonuclease 1 homolog-like 10. pseudogene
0.88	ILF3	interleukin enhancer binding factor 3
0.88	HLA-DRB1	major histocompatibility complex. class II. DR beta 1
0.88	HMBS	hydroxymethylbilane synthase
0.88	PARP9	poly(ADP-ribose) polymerase family member 9
0.88	H2AFZ	H2A histone family. member Z
0.88	ADD1	adducin 1 (alpha)
0.88	SLCO4A1-AS1	SLCO4A1 antisense RNA 1
0.88	KALRN	kalirin. RhoGEF kinase
0.88	TCP11X2	t-complex 11 family. X-linked 2
0.88	EIF3F	Eukaryotic translation initiation factor 3 subunit F [Source:UniProtKB/Swiss-Prot;Acc:O00303]
0.88	AMOTL2; MIR6827; RPL39P5	angiomotin like 2; microRNA 6827; ribosomal protein L39 pseudogene 5
0.88	CFAP74	cilia and flagella associated protein 74
0.88	KLRAP1	killer cell lectin-like receptor subfamily A pseudogene 1
0.88	ZFAT	zinc finger and AT hook domain containing
0.88	BLOC1S5-TXNDC5	BLOC1S5-TXNDC5 readthrough (NMD candidate)
0.88	HID1	HID1 domain containing
0.88	ARHGAP17	Rho GTPase activating protein 17
0.88	CCAR2	cell cycle and apoptosis regulator 2
0.88	TRIM60	tripartite motif containing 60

0.88	HDGFRP3	hepatoma-derived growth factor. related protein 3; Hepatoma-derived growth factor-related protein 3 [Source:UniProtKB/Swiss-Prot;Acc:Q9Y3E1]; Transcript Identified by AceView. Entrez Gene ID(s) 50810; Jeck2013. Memczak2013. Salzman2013 ANNOTATED. CDS. coding. INTERNAL. OVCODE. OVEXON best transcript NM_016073; Jeck2013 ALT_ACCEPTOR. ALT_DONOR. coding. INTERNAL. intronic best transcript NM_016073; Salzman2013 ANNOTATED. CDS. coding. INTERNAL. OVCODE. OVEXON best transcript NM_016073
0.88	HSF4; FBXL8	heat shock transcription factor 4; F-box and leucine-rich repeat protein 8
0.88	DYNC1H1	dynein. cytoplasmic 1. heavy chain 1
0.88	APOA2	apolipoprotein A-II
0.88	FLT3LG	fms-related tyrosine kinase 3 ligand
0.88	TOE1	target of EGR1. member 1 (nuclear)
0.88	NUAK2	NUAK family. SNF1-like kinase. 2
0.88	MYH10	myosin. heavy chain 10. non-muscle
0.88	HLCS	holocarboxylase synthetase (biotin-(proprionyl-CoA-carboxylase (ATP-hydrolysing)) ligase)
0.88	NBPF19	neuroblastoma breakpoint family. member 19
0.88	ST8SIA1	ST8 alpha-N-acetyl-neuraminide alpha-2.8-sialyltransferase 1
0.88	GTF2A1	general transcription factor IIA 1
0.88	PTPRJ	protein tyrosine phosphatase. receptor type. J
0.88	BTBD19	BTB (POZ) domain containing 19
0.88	TRAPPC6A	trafficking protein particle complex 6A
0.88	IFT52	intraflagellar transport 52
0.88	MRPL23; LOC102724828; AC004556.1	Homo sapiens mitochondrial ribosomal protein L23 (MRPL23). mRNA.; 39S ribosomal protein L23. mitochondrial-like; Homo sapiens mitochondrial ribosomal protein L23. mRNA (cDNA clone MGC:12795 IMAGE:4298000). complete cds.
0.88	ANKRD17	ankyrin repeat domain 17

0.88	KRTAP5-11	keratin associated protein 5-11
0.88	BPY2C; BPY2	basic charge. Y-linked. 2C; basic charge. Y-linked. 2
0.88	MUC12	mucin 12. cell surface associated
0.88	MARCH11	membrane associated ring finger 11
0.88	ERF	Ets2 repressor factor
0.88	AKAP8	A kinase (PRKA) anchor protein 8
0.88	CAMK1D	Memczak2013 ALT_ACCEPTOR. ALT_DONOR. coding. INTERNAL. intronic best transcript NM_020397
0.88	PMM2	phosphomannomutase 2
0.88	ARHGAP8	Rho GTPase activating protein 8
0.88	RASSF7	Ras association (RalGDS/AF-6) domain family (N-terminal) member 7
0.88	GOSR2	golgi SNAP receptor complex member 2
0.88	ACAT1	acetyl-CoA acetyltransferase 1
0.88	RPS6KA1	ribosomal protein S6 kinase. 90kDa. polypeptide 1
0.88	MFAP5	microfibrillar associated protein 5
0.88	NCOA2	nuclear receptor coactivator 2
0.88	EFCAB1	EF-hand calcium binding domain 1
0.88	LCN10	lipocalin 10
0.88	ATG4B	autophagy related 4B. cysteine peptidase
0.88	RAB25	RAB25. member RAS oncogene family
0.88	TRHDE-AS1	TRHDE antisense RNA 1
0.88	PUS7L	pseudouridylate synthase 7-like
0.88	CCNE1	cyclin E1
0.88	NDUFB5	NADH dehydrogenase (ubiquinone) 1 beta subcomplex. 5. 16kDa
0.88	HEYL	Transcript Identified by AceView. Entrez Gene ID(s) 26508
0.88	ZNF730	zinc finger protein 730

0.88	WLS	wntless Wnt ligand secretion mediator
0.88	HSPE1	heat shock 10kDa protein 1
0.88	ATP6V1G2	ATPase. H+ transporting. lysosomal 13kDa. V1 subunit G2
0.88	EPHB6	EPH receptor B6
0.88	C10orf25	chromosome 10 open reading frame 25
0.88	SRGAP2	SLIT-ROBO Rho GTPase activating protein 2
0.88	C3orf22	chromosome 3 open reading frame 22
0.88	DET1	de-etiolated homolog 1 (Arabidopsis)
0.88	THNSL2	threonine synthase-like 2
0.88	SLC25A46	solute carrier family 25. member 46
0.88	RHEB	Ras homolog enriched in brain
0.88	MAPK8IP1	mitogen-activated protein kinase 8 interacting protein 1
0.88	CLEC2L	C-type lectin domain family 2. member L
0.88	HCG22	HLA complex group 22
0.88	ATP5C1	ATP synthase. H+ transporting. mitochondrial F1 complex. gamma polypeptide 1
0.88	TPGS1	tubulin polyglutamylase complex subunit 1
0.88	ANKUB1	ankyrin repeat and ubiquitin domain containing 1
0.88	ITGA10	integrin alpha 10
0.88	CHAF1B	chromatin assembly factor 1. subunit B (p60)
0.88	TPBGL	trophoblast glycoprotein-like
0.88	KCNJ4	potassium channel. inwardly rectifying subfamily J. member 4
0.88	RDH13	retinol dehydrogenase 13 (all-trans/9-cis)
0.88	PSMB2	proteasome subunit beta 2
0.88	RP2	retinitis pigmentosa 2 (X-linked recessive)
0.88	MMS22L	MMS22-like. DNA repair protein

0.88	ERAP1	endoplasmic reticulum aminopeptidase 1
0.88	C19orf71	chromosome 19 open reading frame 71
0.88	FAM91A1	family with sequence similarity 91. member A1
0.88	TRAPPC4	trafficking protein particle complex 4
0.88	OGDH	oxoglutarate (alpha-ketoglutarate) dehydrogenase (lipoamide)
0.88	GATA5	GATA binding protein 5
0.88	GAS8-AS1	GAS8 antisense RNA 1
0.88	PRRC2C	proline-rich coiled-coil 2C
0.88	SERPIND1	serpin peptidase inhibitor. clade D (heparin cofactor). member 1
0.88	KIAA2013	KIAA2013
0.88	ARL6IP4	ADP-ribosylation factor like GTPase 6 interacting protein 4
0.88	PRAMEF19	PRAME family member 19 [Source:EntrezGene;Acc:645414]
0.88	DRD4	dopamine receptor D4
0.88	TAF15	TATA box binding protein associated factor 15
0.88	SLC9B2	solute carrier family 9. subfamily B (NHA2. cation proton antiporter 2). member 2
0.88	ANKRD30B	ankyrin repeat domain 30B
0.88	KIAA0513	KIAA0513
0.88	YBX2	Y box binding protein 2
0.88	SLC4A5	solute carrier family 4 (sodium bicarbonate cotransporter). member 5
0.88	NR6A1	nuclear receptor subfamily 6. group A. member 1
0.88	RCAN2	regulator of calcineurin 2
0.88	AVIL	advillin
0.88	GLB1; TMPPE	galactosidase beta 1; transmembrane protein with metallophosphoesterase domain
0.88	GOLPH3L	golgi phosphoprotein 3-like
0.88	NUDT1	nudix hydrolase 1

0.88	R3HDM2	R3H domain containing 2
0.88	KCNK4	potassium channel. two pore domain subfamily K. member 4
0.88	VAV2	vav 2 guanine nucleotide exchange factor
0.88	MEIS3	Meis homeobox 3
0.88	OS9	osteosarcoma amplified 9. endoplasmic reticulum lectin
0.88	TLN1; MIR6852	talin 1; microRNA 6852
0.88	BECN1	beclin 1. autophagy related
0.88	KCNC1	potassium channel. voltage gated Shaw related subfamily C. member 1
0.88	FANCD2	Fanconi anemia complementation group D2
0.88	ARHGAP30	Rho GTPase activating protein 30
0.88	KIF1A	kinesin family member 1A
0.88	MPC1L	mitochondrial pyruvate carrier 1-like
0.88	TUBGCP3	Memczak2013 ANTISENSE. coding. INTERNAL. intronic best transcript NM_006322
0.88	SAYSD1	SAYSVFN motif domain containing 1
0.88	LOC728395; TSPY3	Homo sapiens similar to testis specific protein. Y-linked 1. mRNA (cDNA clone MGC:150413 IMAGE:40120433). complete cds.; Homo sapiens testis specific protein. Y-linked 3. mRNA (cDNA clone MGC:150414 IMAGE:40120435). complete cds.
0.88	ESPL1	extra spindle pole bodies like 1. separase
0.88	SAMD10	sterile alpha motif domain containing 10
0.88	FAM192A	family with sequence similarity 192. member A
0.88	SRBD1	S1 RNA binding domain 1
0.88	C16orf95	chromosome 16 open reading frame 95
0.88	ECM2	extracellular matrix protein 2. female organ and adipocyte specific
0.88	SENP2	SUMO1/sentrin/SMT3 specific peptidase 2
0.88	SKI; kerlabu	Memczak2013 ALT_ACCEPTOR. ALT_DONOR. coding. INTERNAL. intronic best transcript NM_003036; Transcript Identified by AceView

0.88	TREM2	triggering receptor expressed on myeloid cells 2
0.88	RPS6KA1	ribosomal protein S6 kinase. 90kDa. polypeptide 1
0.88	PPP1R3D	protein phosphatase 1. regulatory subunit 3D
0.88	NRROS	negative regulator of reactive oxygen species
0.88	RGPD4	RANBP2-like and GRIP domain containing 4
0.88	TLN2	talin 2
0.88	ZBTB32	zinc finger and BTB domain containing 32
0.88	OR4Q2	olfactory receptor. family 4. subfamily Q. member 2 (gene/pseudogene)
0.88	LASP1	LIM and SH3 protein 1
0.88	ZC3H7B	zinc finger CCCH-type containing 7B
0.88	CHODL	chondrolectin
0.88	ADAMTS6	ADAM metallopeptidase with thrombospondin type 1 motif 6
0.88	TAS2R42	taste receptor. type 2. member 42
0.88	EVPL	envoplakin
0.88	SEC11C	SEC11 homolog C. signal peptidase complex subunit
0.88	HES5	hes family bHLH transcription factor 5
0.88	KLK14	kallikrein related peptidase 14
0.88	OR51G2	olfactory receptor. family 51. subfamily G. member 2
0.88	KRTAP19-1	keratin associated protein 19-1
0.88	SMYD4	SET and MYND domain containing 4
0.88	AGAP3	ArfGAP with GTPase domain. ankyrin repeat and PH domain 3
0.88	ENTPD2	ectonucleoside triphosphate diphosphohydrolase 2
0.88	DPYD	dihydropyrimidine dehydrogenase
0.88	C12orf10	chromosome 12 open reading frame 10
0.88	DNAJC4	DnaJ (Hsp40) homolog. subfamily C. member 4
0.88	ACOT7	acyl-CoA thioesterase 7

0.88	PRCD	progressive rod-cone degeneration
0.88	TRHR	thyrotropin-releasing hormone receptor
0.88	OVCH2	ovochymase 2 (gene/pseudogene)
0.88	PPP1R1A	protein phosphatase 1. regulatory (inhibitor) subunit 1A
0.88	FHAD1	forkhead-associated (FHA) phosphopeptide binding domain 1
0.88	SUCNR1	succinate receptor 1
0.88	PLA2G3	phospholipase A2. group III
0.88	ZNF721; ABCA11P	zinc finger protein 721; ATP binding cassette subfamily A member 11. pseudogene
0.88	MAP4	microtubule associated protein 4
0.88	CCM2	cerebral cavernous malformation 2
0.88	AP1M1	Memczak2013 ALT_ACCEPTOR. ALT_DONOR. coding. INTERNAL. intronic best transcript NM_032493
0.88	PCNXL3	pecanex-like 3 (Drosophila)
0.88	KCNH5	potassium channel. voltage gated eag related subfamily H. member 5
0.88	WASF1	WAS protein family. member 1
0.88	VENTX	VENT homeobox
0.88	HGD	homogentisate 1.2-dioxygenase
0.88	CIAO1	cytosolic iron-sulfur assembly component 1
0.88	CDC20B	cell division cycle 20B
0.88	TMED4	transmembrane p24 trafficking protein 4
0.88	WI2-2373I1.2; pajoyby	forkhead box L1-like; novel transcript; Transcript Identified by AceView
0.88	MDC1	mediator of DNA-damage checkpoint 1
0.88	ITGB1BP1	integrin beta 1 binding protein 1
0.88	LRRC8A	leucine rich repeat containing 8 family. member A
0.88	OAZ2	ornithine decarboxylase antizyme 2
0.88	DNAAF5	dynein. axonemal. assembly factor 5

0.88	LGI2	leucine-rich repeat LGI family. member 2
0.88	COX14	COX14 cytochrome c oxidase assembly factor
0.88	RNF103-CHMP3; CHMP3	RNF103-CHMP3 readthrough; charged multivesicular body protein 3
0.88	ADH1B	alcohol dehydrogenase 1B (class I). beta polypeptide
0.88	THSD1	thrombospondin type 1 domain containing 1
0.88	SMG1	Salzman2013 ALT_ACCEPTOR. ALT_DONOR. coding. INTERNAL. intronic best transcript NM_015092
0.88	RSG1	REM2 and RAB-like small GTPase 1
0.88	RBM10	RNA binding motif protein 10
0.88	OR11H1	olfactory receptor. family 11. subfamily H. member 1
0.88	ADRA2A	adrenoceptor alpha 2A
0.88	C9orf3; RP11-49O14.3	Transcript Identified by AceView. Entrez Gene ID(s) 84909; novel transcript
0.88	GPR155	G protein-coupled receptor 155
0.88	YJEFN3	YjeF N-terminal domain containing 3
0.88	SRPK2	SRSF protein kinase 2
0.88	HIPK2	homeodomain interacting protein kinase 2
0.88	GBAS	glioblastoma amplified sequence
0.88	ZNF625-ZNF20; ZNF625	ZNF625-ZNF20 readthrough (NMD candidate); zinc finger protein 625
0.88	ADAD2	adenosine deaminase domain containing 2
0.88	ACOX3	acyl-CoA oxidase 3. pristanoyl
0.88	STYX	serine/threonine/tyrosine interacting protein
0.88	PSENEN	presenilin enhancer gamma secretase subunit
0.88	MCF2L	MCF.2 cell line derived transforming sequence like
0.88	HS1BP3	HCLS1 binding protein 3
0.88	MOV10	Mov10 RISC complex RNA helicase
0.88	CRY2	Memczak2013 ANTISENSE. CDS. coding. INTERNAL. UTR3 best transcript NM_001127457

0.88	MGST3	microsomal glutathione S-transferase 3
0.88	SLC43A3	solute carrier family 43. member 3
0.88	FBXO31	F-box protein 31
0.88	TRIO	trio Rho guanine nucleotide exchange factor
0.88	MED24; MIR6884	mediator complex subunit 24; microRNA 6884
0.88	TCEB3C	transcription elongation factor B polypeptide 3C (elongin A3)
0.88	BCAS1	breast carcinoma amplified sequence 1
0.88	C21orf58	chromosome 21 open reading frame 58
0.88	HENMT1	HEN1 methyltransferase homolog 1 (Arabidopsis)
0.88	KCTD3	potassium channel tetramerization domain containing 3
0.88	EFNB2	ephrin-B2
0.88	SLA	Src-like-adaptor
0.88	TMEM249	transmembrane protein 249
0.88	TMEM134	transmembrane protein 134
0.88	TMEM80	transmembrane protein 80
0.88	OR51A2	olfactory receptor. family 51. subfamily A. member 2
0.88	AHRR; PDCD6	aryl-hydrocarbon receptor repressor; programmed cell death 6
0.88	MMAA	methylmalonic aciduria (cobalamin deficiency) cblA type
0.88	TSPAN15	tetraspanin 15
0.88	DLGAP4	discs. large (Drosophila) homolog-associated protein 4
0.88	KRT81	keratin 81. type II
0.88	ENG	endoglin
0.88	CARD16	caspase recruitment domain family. member 16
0.88	PHF2	PHD finger protein 2
0.88	ADRA1D	adrenoceptor alpha 1D
0.88	ING1	inhibitor of growth family member 1

0.88	QRICH2	Jeck2013 ALT_DONOR. coding. INTERNAL. intronic best transcript NM_032134
0.88	C2CD3	C2 calcium-dependent domain containing 3
0.88	PRAMEF5	PRAME family member 5
0.88	TMEM106B	transmembrane protein 106B
0.88	ASB10	ankyrin repeat and SOCS box containing 10
0.88	ARPC4-TTLL3	ARPC4-TTLL3 readthrough
0.88	KRTAP22-1	keratin associated protein 22-1
0.88	CALU	calumenin
0.88	MCM3	minichromosome maintenance complex component 3
0.88	ASGR2	asialoglycoprotein receptor 2
0.88	RNF17	ring finger protein 17
0.88	GUK1	guanylate kinase 1
0.88	SLC37A2	solute carrier family 37 (glucose-6-phosphate transporter). member 2
0.88	SNAPC5	small nuclear RNA activating complex polypeptide 5
0.88	HMBS	hydroxymethylbilane synthase
0.88	SRGAP3	SLIT-ROBO Rho GTPase activating protein 3
0.88	ANO6	anoctamin 6
0.88	TRPV4	transient receptor potential cation channel. subfamily V. member 4
0.87	TRAF4	TNF receptor-associated factor 4
0.87	KNG1	kininogen 1
0.87	SUGP1	SURP and G-patch domain containing 1
0.87	USP7	ubiquitin specific peptidase 7 (herpes virus-associated)
0.87	ZNF793	zinc finger protein 793
0.87	PAIP2B	poly(A) binding protein interacting protein 2B
0.87	KCNN2	potassium channel. calcium activated intermediate/small conductance subfamily N alpha. member 2

0.87	NOTCH1	notch 1
0.87	MESDC2	mesoderm development candidate 2
0.87	TOB2	transducer of ERBB2. 2
0.87	MIEF2	mitochondrial elongation factor 2
0.87	SLC37A3	solute carrier family 37. member 3
0.87	POTEG; POTEM	POTE ankyrin domain family. member G; POTE ankyrin domain family. member M
0.87	UPF2	UPF2 regulator of nonsense transcripts homolog (yeast)
0.87	MGAT3	mannosyl (beta-1.4-)-glycoprotein beta-1.4-N-acetylglucosaminyltransferase
0.87	MSH6	mutS homolog 6
0.87	COL5A2	collagen. type V. alpha 2
0.87	SLIRP	SRA stem-loop interacting RNA binding protein
0.87	WBSCR28	Williams-Beuren syndrome chromosome region 28
0.87	DIAPH3	diaphanous-related formin 3
0.87	ARSI	arylsulfatase family. member I
0.87	AQP10	aquaporin 10
0.87	P2RX6	purinergic receptor P2X. ligand gated ion channel. 6
0.87	SLC35E1	solute carrier family 35. member E1
0.87	SHC4	SHC (Src homology 2 domain containing) family. member 4
0.87	ZNF429	zinc finger protein 429
0.87	WDR34	WD repeat domain 34
0.87	GRIK2	glutamate receptor. ionotropic. kainate 2
0.87	LOC102467081; CTD-2313F11.1; slygee	uncharacterized LOC102467081; novel transcript. antisense to GZMK; Transcript Identified by AceView
0.87	RWDD3; TMEM56; TMEM56-RWDD3	RWD domain containing 3; transmembrane protein 56; TMEM56-RWDD3 readthrough
0.87	OR10AC1	olfactory receptor. family 10. subfamily AC. member 1 (gene/pseudogene)

		[Source:HGNC Symbol;Acc:HGNC:14758]
0.87	CDC45	cell division cycle 45
0.87	CLDN9	claudin 9
0.87	GYPC	glycophorin C (Gerbich blood group)
0.87	SMYD5	SMYD family member 5
0.87	CIDEA	cell death-inducing DFFA-like effector a
0.87	GJA4	gap junction protein alpha 4
0.87	KIAA0825	KIAA0825
0.87	SPATA20	spermatogenesis associated 20
0.87	TAS2R30; TAS2R43; PRR4	taste receptor. type 2. member 30; taste receptor. type 2. member 43; proline rich 4 (lacrimal)
0.87	ODF2	outer dense fiber of sperm tails 2
0.87	ZNF77	zinc finger protein 77
0.87	TYMP; SCO2	thymidine phosphorylase; SCO2 cytochrome c oxidase assembly protein
0.87	SF3B2	splicing factor 3b subunit 2
0.87	RAP1GDS1	RAP1. GTP-GDP dissociation stimulator 1
0.87	POTEB2; POTEB; POTEB3	POTE ankyrin domain family. member B2; POTE ankyrin domain family. member B; POTE ankyrin domain family. member B3
0.87	PEAR1	platelet endothelial aggregation receptor 1
0.87	CEBPA	CCAAT/enhancer binding protein (C/EBP). alpha
0.87	KLHL25	kelch-like family member 25
0.87	HSD11B2	hydroxysteroid (11-beta) dehydrogenase 2
0.87	PKIG	protein kinase (cAMP-dependent. catalytic) inhibitor gamma
0.87	NKX2-3	NK2 homeobox 3
0.87	MAGEB16	MAGE family member B16
0.87	ZNF91	zinc finger protein 91
0.87	BEND2	BEN domain containing 2

0.87	CSF2RB	colony stimulating factor 2 receptor. beta. low-affinity (granulocyte-macrophage)
0.87	COX6A2	cytochrome c oxidase subunit VIa polypeptide 2
0.87	DCDC1	doublecortin domain containing 1
0.87	NOXA1	NADPH oxidase activator 1
0.87	FIBP	fibroblast growth factor (acidic) intracellular binding protein
0.87	EPCAM	epithelial cell adhesion molecule
0.87	KDM2A	lysine (K)-specific demethylase 2A
0.87	RUNDC1	RUN domain containing 1
0.87	ABCD2	Transcript Identified by AceView. Entrez Gene ID(s) 225
0.87	KDELR1	KDEL (Lys-Asp-Glu-Leu) endoplasmic reticulum protein retention receptor 1
0.87	UNC79	unc-79 homolog (C. elegans)
0.87	SETBP1	SET binding protein 1
0.87	DGAT2L6	diacylglycerol O-acyltransferase 2-like 6
0.87	TENM4	teneurin transmembrane protein 4
0.87	TFEC	transcription factor EC
0.87	NSUN5P1	NOP2/Sun domain family. member 5 pseudogene 1
0.87	TAF1C	TATA box binding protein (TBP)-associated factor. RNA polymerase I. C. 110kDa
0.87	CLASP2	Transcript Identified by AceView. Entrez Gene ID(s) 23122
0.87	TUBGCP5	tubulin. gamma complex associated protein 5
0.87	GNG5	guanine nucleotide binding protein (G protein). gamma 5
0.87	MRI1	methylthioribose-1-phosphate isomerase 1
0.87	CCDC130	coiled-coil domain containing 130
0.87	DCLK1	doublecortin-like kinase 1
0.87	AOC3	amine oxidase. copper containing 3
0.87	EWSR1	EWS RNA binding protein 1

0.87	PNMAL2	paraneoplastic Ma antigen family-like 2
0.87	KRT31	keratin 31. type I
0.87	CPTP	ceramide-1-phosphate transfer protein
0.87	OR13C8	olfactory receptor. family 13. subfamily C. member 8
0.87	CLPS	colipase. pancreatic
0.87	PURG	purine-rich element binding protein G
0.87	ANGPTL7	angiopoietin like 7
0.87	TAX1BP3	Tax1 (human T-cell leukemia virus type I) binding protein 3
0.87	C16orf54	chromosome 16 open reading frame 54
0.87	ZNF321P	zinc finger protein 321. pseudogene
0.87	CFAP20	cilia and flagella associated protein 20
0.87	ZBP1	Z-DNA binding protein 1
0.87	OAT	ornithine aminotransferase
0.87	CCL13	chemokine (C-C motif) ligand 13
0.87	ZNF512	zinc finger protein 512
0.87	ANXA8	annexin A8
0.87	FAM58A	family with sequence similarity 58. member A
0.87	MMP25-AS1	MMP25 antisense RNA 1
0.87	COL9A3	collagen. type IX. alpha 3
0.87	EFHD2	EF-hand domain family member D2
0.87	C12orf49	chromosome 12 open reading frame 49
0.87	LINC01270	long intergenic non-protein coding RNA 1270
0.87	TIMM8B	translocase of inner mitochondrial membrane 8 homolog B (yeast)
0.87	KIAA1549L	KIAA1549-like
0.87	CPNE4	copine IV
0.87	GH1	growth hormone 1

0.87	TMEM61	transmembrane protein 61
0.87	TAF12	TAF12 RNA polymerase II. TATA box binding protein (TBP)-associated factor. 20kDa
0.87	APCDD1L	adenomatosis polyposis coli down-regulated 1-like
0.87	GIGYF2	GRB10 interacting GYF protein 2
0.87	EXTL2	exostosin-like glycosyltransferase 2
0.87	DDX11	Zhang2013 ALT_ACCEPTOR. ALT_DONOR. coding. INTERNAL. intronic best transcript NM_030653
0.87	NBPF26	neuroblastoma breakpoint family. member 26
0.87	TEN1; CDK3; TEN1-CDK3	TEN1 CST complex subunit; cyclin-dependent kinase 3; TEN1-CDK3 readthrough (NMD candidate)
0.87	RARRES1	retinoic acid receptor responder (tazarotene induced) 1
0.87	EDAR	ectodysplasin A receptor
0.87	CCL17	chemokine (C-C motif) ligand 17
0.87	PSTK	phosphoseryl-tRNA kinase
0.87	C1orf145	chromosome 1 open reading frame 145
0.87	GCHFR	GTP cyclohydrolase I feedback regulator
0.87	NSUN3	NOP2/Sun domain family. member 3
0.87	KCNV1	potassium channel. voltage gated modifier subfamily V. member 1
0.87	ORAI2	ORAI calcium release-activated calcium modulator 2
0.87	GRK6	G protein-coupled receptor kinase 6
0.87	ADAMTS14	ADAM metallopeptidase with thrombospondin type 1 motif 14
0.87	CD226	CD226 molecule
0.87	KCNAB2	potassium channel. voltage gated subfamily A regulatory beta subunit 2
0.87	RANGRF	RAN guanine nucleotide release factor
0.87	GRIN2D	glutamate receptor. ionotropic. N-methyl D-aspartate 2D
0.87	CARHSP1	calcium regulated heat stable protein 1

0.87	SIX3	SIX homeobox 3
0.87	LY6D	lymphocyte antigen 6 complex. locus D
0.87	PRB3	proline-rich protein BstNI subfamily 3
0.87	UBAP2L	ubiquitin associated protein 2 like
0.87	CEACAM20	carcinoembryonic antigen-related cell adhesion molecule 20
0.87	SLC2A6	solute carrier family 2 (facilitated glucose transporter). member 6 [Source:HGNC Symbol;Acc:HGNC:11011]
0.87	STAG1	stromal antigen 1
0.87	ZNF611	zinc finger protein 611
0.87	PSIP1	PC4 and SFRS1 interacting protein 1
0.87	MIA; RAB4B; MIA-RAB4B	melanoma inhibitory activity; RAB4B. member RAS oncogene family; MIA-RAB4B readthrough (NMD candidate)
0.87	ALDH5A1	aldehyde dehydrogenase 5 family. member A1
0.87	ADAM21	ADAM metallopeptidase domain 21
0.87	ARHGEF10L	Rho guanine nucleotide exchange factor 10 like
0.87	SCGB2A1	secretoglobin. family 2A. member 1
0.87	CLASP2	Transcript Identified by AceView. Entrez Gene ID(s) 23122
0.87	DGKD	diacylglycerol kinase. delta 130kDa
0.87	EDN2	endothelin 2
0.87	SBSN	suprabasin
0.87	SPRR4	small proline-rich protein 4
0.87	SDC2	syndecan 2
0.87	OR6C6	olfactory receptor. family 6. subfamily C. member 6
0.87	CD244	CD244 molecule. natural killer cell receptor 2B4
0.87	GPR32	G protein-coupled receptor 32
0.87	NAIP	NLR family. apoptosis inhibitory protein
0.87	MRPS34	mitochondrial ribosomal protein S34

0.87	ZNF587	zinc finger protein 587
0.87	ERCC6; ERCC6-PGBD3; PGBD3	excision repair cross-complementation group 6; ERCC6-PGBD3 readthrough; piggyBac transposable element derived 3
0.87	AP3B2	adaptor-related protein complex 3. beta 2 subunit
0.87	ACTN3	actinin. alpha 3 (gene/pseudogene)
0.87	GATA1	GATA binding protein 1 (globin transcription factor 1)
0.87	C18orf42	chromosome 18 open reading frame 42
0.87	CSF1R	colony stimulating factor 1 receptor
0.87	PLXNB1	plexin B1
0.87	APH1A	APH1A gamma secretase subunit
0.87	EXOC3L1	exocyst complex component 3-like 1
0.87	ABCD3	ATP binding cassette subfamily D member 3
0.87	ARSD	arylsulfatase D
0.87	SERPINA5	serpin peptidase inhibitor. clade A (alpha-1 antiproteinase. antitrypsin). member 5
0.87	FN1	fibronectin 1
0.87	OGG1	8-oxoguanine DNA glycosylase
0.87	SLC6A9	solute carrier family 6 (neurotransmitter transporter. glycine). member 9
0.87	ALG1L	ALG1. chitobiosyldiphosphodolichol beta-mannosyltransferase-like
0.87	C1orf35	chromosome 1 open reading frame 35
0.87	CLDN7	claudin 7
0.87	GSDMC	gasdermin C
0.87	FRAS1	Fraser extracellular matrix complex subunit 1
0.87	WSCD1	WSC domain containing 1
0.87	SRD5A2	steroid-5-alpha-reductase. alpha polypeptide 2 (3-oxo-5 alpha-steroid delta 4-dehydrogenase alpha 2)
0.87	PPP2R1A	protein phosphatase 2. regulatory subunit A. alpha

0.87	MAS1	MAS1 proto-oncogene. G protein-coupled receptor
0.87	FAM189A2	family with sequence similarity 189. member A2
0.87	TBC1D3P1-DHX40P1	TBC1D3P1-DHX40P1 readthrough transcribed pseudogene
0.87	USO1	USO1 vesicle transport factor
0.87	CASP2	caspase 2
0.87	KRTAP4-11	keratin associated protein 4-11
0.87	GNB4	guanine nucleotide binding protein (G protein). beta polypeptide 4
0.87	CATIP	ciliogenesis associated TTC17 interacting protein
0.87	SH2D4B	SH2 domain containing 4B
0.87	PDXK	pyridoxal (pyridoxine. vitamin B6) kinase
0.87	MCM6	minichromosome maintenance complex component 6
0.87	ZFYVE21	zinc finger. FYVE domain containing 21
0.87	PDIA3	protein disulfide isomerase family A member 3
0.87	KRTAP4-9	keratin associated protein 4-9
0.87	ASL	argininosuccinate lyase
0.87	TBC1D20	TBC1 domain family. member 20
0.87	RTN4	reticulon 4
0.87	LRP1B	LDL receptor related protein 1B
0.87	OR4A5	olfactory receptor. family 4. subfamily A. member 5
0.87	TSR3	TSR3. 20S rRNA accumulation. homolog (S. cerevisiae)
0.87	ADAM22	ADAM metallopeptidase domain 22
0.87	DUX5	double homeobox 5
0.87	HOXD8	homeobox D8
0.87	RBM26	Memczak2013 ANTISENSE. CDS. coding. INTERNAL best transcript NM_022118
0.87	ST6GALNAC3	Memczak2013 ANTISENSE. coding. INTERNAL. intronic best transcript NM_152996

0.87	EDARADD	EDAR-associated death domain
0.87	ID4	inhibitor of DNA binding 4. dominant negative helix-loop-helix protein
0.87	TMEM114	transmembrane protein 114
0.87	OTOP2	otopetrin 2
0.87	RBM26	Transcript Identified by AceView. Entrez Gene ID(s) 64062
0.87	FCRL4	Fc receptor-like 4
0.87	DMTN	dematin actin binding protein
0.87	TCTE3	t-complex-associated-testis-expressed 3
0.87	CTDSP1	CTD small phosphatase 1
0.87	MYLIP; MIR4639	myosin regulatory light chain interacting protein; microRNA 4639
0.87	KANK2	KN motif and ankyrin repeat domains 2
0.87	APLN	apelin
0.87	ABHD3	abhydrolase domain containing 3
0.87	OR9A2	olfactory receptor. family 9. subfamily A. member 2
0.87	FEV	FEV (ETS oncogene family)
0.87	APLP1	amyloid beta (A4) precursor-like protein 1
0.87	DBX2	developing brain homeobox 2
0.87	HTR6	5-hydroxytryptamine (serotonin) receptor 6. G protein-coupled
0.87	WHAMM	WAS protein homolog associated with actin. golgi membranes and microtubules
0.87	DPM3	dolichyl-phosphate mannosyltransferase polypeptide 3
0.87	MARCKSL1	MARCKS-like 1
0.87	MPZL1	myelin protein zero-like 1
0.87	LCN12	lipocalin 12
0.87	RAB40AL	RAB40A. member RAS oncogene family-like
0.87	STPG1	sperm-tail PG-rich repeat containing 1

0.87	POLR3H	polymerase (RNA) III (DNA directed) polypeptide H (22.9kD)
0.87	KRTAP21-1	keratin associated protein 21-1
0.87	UQCC1	ubiquinol-cytochrome c reductase complex assembly factor 1
0.87	MYOM3	myomesin 3
0.87	CHMP2B	charged multivesicular body protein 2B
0.87	DLX4	distal-less homeobox 4
0.87	SHISA8	shisa family member 8
0.87	TUBB	tubulin. beta class I
0.87	NPY	neuropeptide Y
0.87	PLAC9	placenta specific 9
0.87	CCDC107	coiled-coil domain containing 107
0.87	IL18RAP	interleukin 18 receptor accessory protein
0.87	CYP27A1	cytochrome P450. family 27. subfamily A. polypeptide 1
0.87	TPP1	tripeptidyl peptidase I
0.87	C19orf43	chromosome 19 open reading frame 43
0.87	SLURP1	secreted LY6/PLAUR domain containing 1
0.87	FHIT	fragile histidine triad
0.87	DISC1	disrupted in schizophrenia 1
0.87	NINJ2	ninjurin 2
0.87	FAM53C	family with sequence similarity 53. member C
0.87	SPATA5	Transcript Identified by AceView. Entrez Gene ID(s) 166378
0.87	SOX13	SRY box 13
0.87	TDRD12	tudor domain containing 12
0.87	ANP32A	acidic nuclear phosphoprotein 32 family member A
0.87	FAM167B	family with sequence similarity 167. member B
0.87	FAM65C	family with sequence similarity 65. member C

0.87	KLHDC4	Transcript Identified by AceView. Entrez Gene ID(s) 54758; 100129637
0.87	FRMD6	FERM domain containing 6
0.87	SEC24C	SEC24 homolog C. COPII coat complex component
0.87	ARHGAP19	Rho GTPase activating protein 19
0.87	ZFY	zinc finger protein. Y-linked
0.87	FBXL15	F-box and leucine-rich repeat protein 15
0.87	NDUFB3	NADH dehydrogenase (ubiquinone) 1 beta subcomplex. 3. 12kDa
0.87	LTK	leukocyte receptor tyrosine kinase
0.87	CER1	cerberus 1. DAN family BMP antagonist
0.87	CHID1	chitinase domain containing 1
0.87	DGKD	diacylglycerol kinase. delta 130kDa
0.87	POGZ	Transcript Identified by AceView. Entrez Gene ID(s) 23126
0.87	ADH6	alcohol dehydrogenase 6 (class V)
0.87	OR14C36	olfactory receptor. family 14. subfamily C. member 36
0.87	SYT13	synaptotagmin XIII
0.87	ZNF184	zinc finger protein 184
0.87	OARD1	O-acyl-ADP-ribose deacylase 1
0.87	FARP1; MIR3170	FERM. ARH/RhoGEF and pleckstrin domain protein 1; microRNA 3170
0.87	DNA2	DNA replication helicase/nuclease 2
0.87	LRRC45	leucine rich repeat containing 45
0.87	RBM48	RNA binding motif protein 48
0.87	CTNNBIP1	catenin. beta interacting protein 1
0.87	PGK1	phosphoglycerate kinase 1
0.87	C8G	complement component 8. gamma polypeptide
0.87	BSG	basigin (Ok blood group)
0.87	TALDO1	transaldolase 1

0.87	PRRT2	proline-rich transmembrane protein 2
0.87	ASIC4	acid sensing ion channel family member 4
0.87	HYAL2	hyaluronoglucosaminidase 2
0.87	DUSP23	dual specificity phosphatase 23
0.87	CCDC181	coiled-coil domain containing 181
0.87	SSH1	slingshot protein phosphatase 1
0.87	IGFL4	IGF like family member 4
0.87	SLC35B2; MIR4647	solute carrier family 35 (adenosine 3-phospho 5-phosphosulfate transporter). member B2; microRNA 4647
0.87	KRTAP4-4	keratin associated protein 4-4
0.87	NTSR2	neurotensin receptor 2
0.87	PRG4	proteoglycan 4
0.87	TFCP2L1	transcription factor CP2-like 1
0.87	DGKD	diacylglycerol kinase. delta 130kDa
0.87	SFSWAP	splicing factor. suppressor of white-apricot family
0.87	RAB20	RAB20. member RAS oncogene family
0.87	FGF22	fibroblast growth factor 22
0.87	CALR3	calreticulin 3
0.87	NUDT10	nudix hydrolase 10
0.87	ENPP3	ectonucleotide pyrophosphatase/phosphodiesterase 3
0.87	SNX11	sorting nexin 11
0.87	SMPD1	sphingomyelin phosphodiesterase 1. acid lysosomal
0.87	PAFAH2	platelet-activating factor acetylhydrolase 2
0.87	HELZ	helicase with zinc finger
0.87	FXYD6; FXYD6-FXYD2; FXYD2	FXYD domain containing ion transport regulator 6; FXYD6-FXYD2 readthrough; FXYD domain containing ion transport regulator 2
0.87	TBC1D16	TBC1 domain family. member 16

0.87	HBZ	hemoglobin. zeta
0.87	FOXB2	forkhead box B2
0.87	KRT4	keratin 4. type II
0.87	DHRS1	dehydrogenase/reductase (SDR family) member 1
0.87	HNRNPA2B1	heterogeneous nuclear ribonucleoprotein A2/B1
0.87	BBS9	Bardet-Biedl syndrome 9
0.87	FUT3	fucosyltransferase 3 (galactoside 3(4)-L-fucosyltransferase. Lewis blood group)
0.87	ZNF687	zinc finger protein 687
0.87	RAB13	RAB13. member RAS oncogene family
0.87	FRYL	FRY like transcription coactivator
0.87	PASK	PAS domain containing serine/threonine kinase
0.87	ALDH16A1	aldehyde dehydrogenase 16 family. member A1
0.87	RBM17	RNA binding motif protein 17
0.87	KRTAP10-6	keratin associated protein 10-6
0.87	COLGALT1	collagen beta(1-O)galactosyltransferase 1
0.87	HDAC10	histone deacetylase 10
0.87	FAM127C	family with sequence similarity 127. member C
0.87	PLEKHJ1; MIR6789	pleckstrin homology domain containing. family J member 1; microRNA 6789
0.87	ASH1L-AS1	ASH1L antisense RNA 1
0.87	PIGX	phosphatidylinositol glycan anchor biosynthesis class X
0.87	C9orf69	chromosome 9 open reading frame 69
0.87	MTG2	mitochondrial ribosome-associated GTPase 2
0.87	AK9	adenylate kinase 9
0.87	GDNF	glial cell derived neurotrophic factor
0.87	MCF2L	MCF.2 cell line derived transforming sequence like

0.87	SPINT1	serine peptidase inhibitor. Kunitz type 1
0.87	TIFA	TRAF-interacting protein with forkhead-associated domain
0.87	SPATA5	spermatogenesis associated 5
0.87	KCNE1	potassium channel. voltage gated subfamily E regulatory beta subunit 1
0.87	FAM159B	family with sequence similarity 159. member B
0.87	DTNBP1	dystrobrevin binding protein 1
0.87	PRH1	proline-rich protein HaeIII subfamily 1
0.87	CD300E	CD300e molecule
0.87	KDM5C	Transcript Identified by AceView. Entrez Gene ID(s) 8242
0.87	AS3MT	arsenite methyltransferase
0.87	ARHGAP18	Rho GTPase activating protein 18
0.87	IQCE	IQ motif containing E
0.87	CUL3	cullin 3
0.87	FER1L5	fer-1-like family member 5
0.87	CBY3	chibby homolog 3 (Drosophila)
0.87	SUN2	Sad1 and UNC84 domain containing 2
0.87	SLC11A1	solute carrier family 11 (proton-coupled divalent metal ion transporter). member 1
0.87	SMUG1	single-strand-selective monofunctional uracil-DNA glycosylase 1
0.87	TMCO1	transmembrane and coiled-coil domains 1
0.87	NEFL	neurofilament. light polypeptide
0.87	CUL4A	Memczak2013 ALT_ACCEPTOR. ALT_DONOR. coding. INTERNAL. intronic best transcript NM_001008895
0.87	AMPH	amphiphysin
0.87	ATOX1	antioxidant 1 copper chaperone
0.87	TMEM183B	transmembrane protein 183B
0.87	MGMT	O-6-methylguanine-DNA methyltransferase

0.87	SPATA31A5; SPATA31A7	SPATA31 subfamily A. member 5; SPATA31 subfamily A. member 7
0.87	LINS1	lines homolog 1
0.87	DAB2IP	DAB2 interacting protein
0.87	ARPP21	Transcript Identified by AceView. Entrez Gene ID(s) 10777
0.87	HTR3C	5-hydroxytryptamine (serotonin) receptor 3C. ionotropic
0.87	ERI3; ERI3-IT1	ERI1 exoribonuclease family member 3; ERI3 intronic transcript 1
0.87	TESPA1	thymocyte expressed. positive selection associated 1
0.87	YPEL1	yippee like 1
0.87	CPB2	carboxypeptidase B2 (plasma)
0.87	CFAP44	cilia and flagella associated protein 44
0.87	FRMD4B	FERM domain containing 4B
0.87	DBNL; MIR6837	drebrin-like; microRNA 6837
0.87	CIDEB	cell death-inducing DFFA-like effector b
0.87	ACAD8	acyl-CoA dehydrogenase family. member 8
0.87	B4GALNT4	beta-1.4-N-acetyl-galactosaminyl transferase 4
0.87	ZNF763	zinc finger protein 763
0.87	ORMDL3	ORMDL sphingolipid biosynthesis regulator 3
0.87	CANT1	calcium activated nucleotidase 1
0.87	GCH1	GTP cyclohydrolase 1
0.87	NCKIPSD	NCK interacting protein with SH3 domain
0.87	CXCR3	chemokine (C-X-C motif) receptor 3
0.87	ELOVL5	ELOVL fatty acid elongase 5
0.87	GNAO1	guanine nucleotide binding protein (G protein). alpha activating activity polypeptide O
0.87	ADH1C	alcohol dehydrogenase 1C (class I). gamma polypeptide
0.87	COPRS	coordinator of PRMT5. differentiation stimulator

0.87	FABP7	fatty acid binding protein 7. brain
0.87	WRN	Transcript Identified by AceView. Entrez Gene ID(s) 7486
0.87	MED14OS	MED14 opposite strand
0.87	CROCC	ciliary rootlet coiled-coil. rootletin
0.87	SEC22B	Salzman2013 ALT_ACCEPTOR. CDS. coding. INTERNAL. OVCODE. OVEXON best transcript NM_004892; Salzman2013 ANNOTATED. CDS. coding. OVCODE. OVEXON. UTR3 best transcript NM_004892
0.87	RASA4	RAS p21 protein activator 4
0.87	STAMBP	STAM binding protein
0.87	NEDD1	neural precursor cell expressed. developmentally down-regulated 1
0.87	LILRB3	leukocyte immunoglobulin-like receptor. subfamily B (with TM and ITIM domains). member 3
0.87	FAM84A	family with sequence similarity 84. member A
0.87	ZNF500	zinc finger protein 500
0.87	CCDC42	coiled-coil domain containing 42
0.87	LTB	lymphotoxin beta (TNF superfamily. member 3)
0.87	LSR	lipolysis stimulated lipoprotein receptor
0.87	TP53TG3D	TP53 target 3D
0.87	RBAK; RBAK-RBAKDN; RBAKDN	RB-associated KRAB zinc finger; RBAK-RBAKDN readthrough; RBAK downstream neighbor (non-protein coding)
0.87	BRIP1	BRCA1 interacting protein C-terminal helicase 1
0.87	CD248	CD248 molecule. endosialin
0.87	ITM2B	integral membrane protein 2B
0.87	ENPP2	ectonucleotide pyrophosphatase/phosphodiesterase 2
0.87	TIMM17B	translocase of inner mitochondrial membrane 17 homolog B (yeast)
0.87	LRRC75B	leucine rich repeat containing 75B
0.87	ZBTB14	zinc finger and BTB domain containing 14

0.87	ATP9B	ATPase. class II. type 9B
0.87	PSMA3-AS1	PSMA3 antisense RNA 1
0.87	RGS9	regulator of G-protein signaling 9
0.87	TBC1D22A	TBC1 domain family. member 22A
0.87	TBC1D3G	TBC1 domain family. member 3G
0.87	MSR1	macrophage scavenger receptor 1
0.87	DEGS2	delta(4)-desaturase. sphingolipid 2
0.87	OR10H3	olfactory receptor. family 10. subfamily H. member 3
0.87	TRAFD1	TRAF-type zinc finger domain containing 1
0.87	RNF208	ring finger protein 208
0.87	SF3B1	splicing factor 3b. subunit 1. 155kDa
0.87	GPR12	G protein-coupled receptor 12
0.87	HELB	helicase (DNA) B
0.87	ACO2	aconitase 2. mitochondrial
0.87	BCLAF1	Memczak2013 ALT_DONOR. coding. INTERNAL. intronic best transcript NM_014739
0.87	HS3ST6	heparan sulfate (glucosamine) 3-O-sulfotransferase 6
0.87	C1orf94	chromosome 1 open reading frame 94
0.87	OCIAD2	OCIA domain containing 2
0.87	PGAP3	post-GPI attachment to proteins 3
0.87	RXRA	Memczak2013 ANTISENSE. CDS. coding. INTERNAL best transcript NM_002957
0.87	MAG	myelin associated glycoprotein
0.87	APOB	apolipoprotein B
0.87	TMEM255B	transmembrane protein 255B
0.87	OSER1	oxidative stress responsive serine-rich 1
0.87	PTGES	prostaglandin E synthase

0.87	TP53I11	tumor protein p53 inducible protein 11
0.87	KLRC2	killer cell lectin-like receptor subfamily C. member 2
0.87	GPER1	G protein-coupled estrogen receptor 1
0.87	TSPO	translocator protein (18kDa)
0.87	LIMK1	LIM domain kinase 1
0.87	KIAA0895L	KIAA0895-like
0.87	OXLD1	oxidoreductase-like domain containing 1
0.87	ZFP90	ZFP90 zinc finger protein
0.87	TPH2	tryptophan hydroxylase 2
0.87	ATP6V1D	ATPase. H+ transporting. lysosomal 34kDa. V1 subunit D
0.87	ENO3	enolase 3 (beta. muscle)
0.86	WNT3	wingless-type MMTV integration site family. member 3
0.86	NBR1	neighbor of BRCA1 gene 1
0.86	FGD6	FYVE. RhoGEF and PH domain containing 6
0.86	TONSL	tonsoku-like. DNA repair protein
0.86	HMBS	hydroxymethylbilane synthase
0.86	CDK12	cyclin-dependent kinase 12
0.86	MUTYH	mutY DNA glycosylase
0.86	SOD1	superoxide dismutase 1. soluble
0.86	PELP1	proline. glutamate and leucine rich protein 1
0.86	FAM107A	family with sequence similarity 107. member A
0.86	IFIT2	interferon-induced protein with tetratricopeptide repeats 2
0.86	SH3BGR	SH3 domain binding glutamate-rich protein
0.86	CYP2W1	cytochrome P450. family 2. subfamily W. polypeptide 1
0.86	FAM64A	family with sequence similarity 64. member A
0.86	APBB3	amyloid beta (A4) precursor protein-binding. family B. member 3

0.86	KLHL6	kelch-like family member 6
0.86	HUWE1	HECT. UBA and WWE domain containing 1. E3 ubiquitin protein ligase
0.86	SUN5	Sad1 and UNC84 domain containing 5
0.86	EAF2	ELL associated factor 2
0.86	CLEC3B	C-type lectin domain family 3. member B
0.86	FAM104B	family with sequence similarity 104. member B
0.86	CLEC17A	C-type lectin domain family 17. member A
0.86	RUNX1T1	runt-related transcription factor 1; translocated to. 1 (cyclin D-related)
0.86	GMPPA	GDP-mannose pyrophosphorylase A
0.86	HNRNPUL2-BSCL2	HNRNPUL2-BSCL2 readthrough (NMD candidate)
0.86	ALAS2	5-aminolevulinate synthase 2
0.86	SCMH1	sex comb on midleg homolog 1 (Drosophila)
0.86	SH3BGRL	SH3 domain binding glutamate-rich protein like
0.86	SCML4	sex comb on midleg-like 4 (Drosophila)
0.86	SHPRH	SNF2 histone linker PHD RING helicase. E3 ubiquitin protein ligase
0.86	TOX4	TOX high mobility group box family member 4
0.86	NHP2	NHP2 ribonucleoprotein
0.86	DEFB103A; DEFB103B	defensin. beta 103A; defensin. beta 103B
0.86	MSMO1	methylsterol monooxygenase 1
0.86	KLF2	Kruppel-like factor 2
0.86	SAG	S-antigen; retina and pineal gland (arrestin)
0.86	ENGASE	endo-beta-N-acetylglucosaminidase
0.86	FAM201A	family with sequence similarity 201. member A
0.86	NIPAL2	NIPA-like domain containing 2
0.86	NBPF20	neuroblastoma breakpoint family. member 20
0.86	PRAM1	PML-RARA regulated adaptor molecule 1

0.86	RBM4B	RNA binding motif protein 4B
0.86	TSSK4	testis-specific serine kinase 4
0.86	PTGS2	prostaglandin-endoperoxide synthase 2 (prostaglandin G/H synthase and cyclooxygenase)
0.86	MAN2B1	mannosidase. alpha. class 2B. member 1
0.86	CCNE2	cyclin E2
0.86	NUDC	nudC nuclear distribution protein
0.86	ZNF853	zinc finger protein 853
0.86	BAIAP2	BAI1-associated protein 2
0.86	KCNH4	potassium channel. voltage gated eag related subfamily H. member 4
0.86	DCAF8	DDB1 and CUL4 associated factor 8
0.86	SMPD2	sphingomyelin phosphodiesterase 2. neutral membrane (neutral sphingomyelinase)
0.86	ELMOD2	ELMO/CED-12 domain containing 2
0.86	SMC3	structural maintenance of chromosomes 3
0.86	AMELY	amelogenin. Y-linked
0.86	GUCY1B3	guanylate cyclase 1. soluble. beta 3
0.86	PRX	periaxin
0.86	OC90	otoconin 90
0.86	FAM114A2	family with sequence similarity 114. member A2
0.86	ZC3H13	zinc finger CCCH-type containing 13
0.86	HOGA1	4-hydroxy-2-oxoglutarate aldolase 1
0.86	P2RY8	purinergic receptor P2Y. G-protein coupled. 8
0.86	P2RY8	Homo sapiens purinergic receptor P2Y. G-protein coupled. 8 (P2RY8). mRNA.; purinergic receptor P2Y. G-protein coupled. 8 [Source:HGNC Symbol;Acc:HGNC:15524]; Homo sapiens purinergic receptor P2Y. G-protein coupled. 8. mRNA (cDNA clone MGC:50878 IMAGE:5787268). complete cds.
0.86	PIP5K1B	phosphatidylinositol-4-phosphate 5-kinase. type I. beta

0.86	ZNF564	zinc finger protein 564
0.86	PRAP1	proline-rich acidic protein 1
0.86	CD207	CD207 molecule. langerin
0.86	MON1B	MON1 secretory trafficking family member B
0.86	FAM20A	family with sequence similarity 20. member A
0.86	MCU	mitochondrial calcium uniporter
0.86	C19orf45	chromosome 19 open reading frame 45
0.86	HELT	helt bHLH transcription factor
0.86	MTR	5-methyltetrahydrofolate-homocysteine methyltransferase
0.86	LIM2	lens intrinsic membrane protein 2
0.86	PTCHD3	patched domain containing 3
0.86	SPANXN5	SPANX family. member N5
0.86	ZNF536	zinc finger protein 536
0.86	MBD3L3	methyl-CpG binding domain protein 3-like 3
0.86	AGPAT1; MIR6721	1-acylglycerol-3-phosphate O-acyltransferase 1; microRNA 6721
0.86	PLSCR3; TMEM256; TMEM256-PLSCR3	phospholipid scramblase 3; transmembrane protein 256; TMEM256-PLSCR3 readthrough (NMD candidate)
0.86	EID2	EP300 interacting inhibitor of differentiation 2
0.86	NT5E	5-nucleotidase. ecto (CD73)
0.86	FAM13A	family with sequence similarity 13. member A
0.86	PRKD2	protein kinase D2
0.86	PRPF40B	PRP40 homolog. pre-mRNA processing factor B
0.86	TOLLIP	toll interacting protein
0.86	EYS	eyes shut homolog (Drosophila)
0.86	SFTPD	surfactant protein D
0.86	DEFA5	defensin. alpha 5. Paneth cell-specific

0.86	CPA3	carboxypeptidase A3 (mast cell)
0.86	LRRC4	leucine rich repeat containing 4
0.86	TCOF1	Treacher Collins-Franceschetti syndrome 1
0.86	OR52E8	olfactory receptor. family 52. subfamily E. member 8
0.86	FAM161A	family with sequence similarity 161. member A
0.86	GDNF-AS1	GDNF antisense RNA 1 (head to head)
0.86	HHLA1	HERV-H LTR-associating 1
0.86	SCAPER	Transcript Identified by AceView. Entrez Gene ID(s) 49855
0.86	TMSB4X	thymosin beta 4. X-linked
0.86	C5orf64	chromosome 5 open reading frame 64
0.86	PPIAL4C	peptidylprolyl isomerase A (cyclophilin A)-like 4C
0.86	RTKN	rhotekin
0.86	ACAP1	ArfGAP with coiled-coil. ankyrin repeat and PH domains 1
0.86	SCNN1A	sodium channel. non voltage gated 1 alpha subunit
0.86	RASAL1	RAS protein activator like 1 (GAP1 like)
0.86	SGK3	serum/glucocorticoid regulated kinase family. member 3
0.86	ATP9A	ATPase. class II. type 9A
0.86	ATP8; ATP6; COX3	ATP synthase F0 subunit 8; ATP synthase F0 subunit 6; cytochrome c oxidase III
0.86	TNFAIP1	tumor necrosis factor. alpha-induced protein 1 (endothelial)
0.86	NFAM1	NFAT activating protein with ITAM motif 1
0.86	ZNF487	zinc finger protein 487
0.86	TRIM69	tripartite motif containing 69
0.86	HMBS	hydroxymethylbilane synthase
0.86	ASB4	ankyrin repeat and SOCS box containing 4
0.86	CCDC74B	coiled-coil domain containing 74B

0.86	TUSC5	tumor suppressor candidate 5
0.86	MAGI3	Jeck2013 ALT_ACCEPTOR. ALT_DONOR. coding. INTERNAL. intronic best transcript NM_152900
0.86	EHD1	EH domain containing 1
0.86	KCNMA1	potassium channel. calcium activated large conductance subfamily M alpha. member 1
0.86	RHO	rhodopsin
0.86	MLKL	mixed lineage kinase domain-like
0.86	AQP2	aquaporin 2 (collecting duct)
0.86	ACTN4	actinin. alpha 4
0.86	GALT	galactose-1-phosphate uridylyltransferase
0.86	MAPKAP1	mitogen-activated protein kinase associated protein 1
0.86	NCAM1	Transcript Identified by AceView. Entrez Gene ID(s) 4684
0.86	TFG	TRK-fused gene
0.86	AIG1	androgen-induced 1
0.86	SLC22A16	solute carrier family 22 (organic cation/carnitine transporter). member 16
0.86	CDCA7L	cell division cycle associated 7-like
0.86	SLC25A19	solute carrier family 25 (mitochondrial thiamine pyrophosphate carrier). member 19
0.86	COX6A1	cytochrome c oxidase subunit VIa polypeptide 1
0.86	ZDHHC20	zinc finger. DHHC-type containing 20
0.86	SMARCD2	SWI/SNF related. matrix associated. actin dependent regulator of chromatin. subfamily d. member 2
0.86	DEC1	deleted in esophageal cancer 1
0.86	PCDH9	protocadherin 9
0.86	KIF15	kinesin family member 15
0.86	TRIM39-RPP21	TRIM39-RPP21 readthrough

0.86	BCL2L14	BCL2-like 14 (apoptosis facilitator)
0.86	GBP1	guanylate binding protein 1. interferon-inducible
0.86	SRRM3	serine/arginine repetitive matrix 3
0.86	LRIT3	leucine-rich repeat. immunoglobulin-like and transmembrane domains 3
0.86	PITHD1	PITH (C-terminal proteasome-interacting domain of thioredoxin-like) domain containing 1
0.86	PAK3	p21 protein (Cdc42/Rac)-activated kinase 3
0.86	ASAH1	N-acylsphingosine amidohydrolase (acid ceramidase) 1
0.86	OCM	oncomodulin
0.86	RPS6KA3	ribosomal protein S6 kinase. 90kDa. polypeptide 3
0.86	GCSH	glycine cleavage system protein H (aminomethyl carrier)
0.86	GAGE2B; GAGE2A; GAGE2C	G antigen 2B; G antigen 2A; G antigen 2C
0.86	TIPARP	Memczak2013 ANTISENSE. CDS. coding. INTERNAL best transcript NM_001184717
0.86	CLCA2	chloride channel accessory 2
0.86	C8orf49	chromosome 8 open reading frame 49
0.86	YTHDC1	YTH domain containing 1
0.86	ARL5A	ADP-ribosylation factor like GTPase 5A
0.86	ZNF735	zinc finger protein 735
0.86	HYAL3	hyaluronoglucosaminidase 3
0.86	HOXB8	homeobox B8
0.86	ZDHHC16	zinc finger. DHHC-type containing 16
0.86	HBS1L	HBS1-like translational GTPase
0.86	CSPG5	chondroitin sulfate proteoglycan 5 (neuroglycan C)
0.86	SIM1	single-minded family bHLH transcription factor 1
0.86	OPN3	opsin 3
0.86	OTULIN	OTU deubiquitinase with linear linkage specificity

0.86	EIF1AD	eukaryotic translation initiation factor 1A domain containing
0.86	VHL	von Hippel-Lindau tumor suppressor. E3 ubiquitin protein ligase
0.86	USP12	Transcript Identified by AceView. Entrez Gene ID(s) 219333
0.86	INSL4	insulin-like 4 (placenta)
0.86	SASH1	SAM and SH3 domain containing 1
0.86	SHISA2	shisa family member 2
0.86	SYTL2	synaptotagmin-like 2
0.86	SLC38A9	Transcript Identified by AceView. Entrez Gene ID(s) 153129
0.86	LARP7	Zhang2013 ALT_DONOR. coding. INTERNAL. intronic. OVERLAPTX. OVEXON best transcript NM_016648
0.86	PTK6	protein tyrosine kinase 6
0.86	LIPA	lipase A. lysosomal acid. cholesterol esterase
0.86	THAP7	THAP domain containing 7
0.86	PYCRL	pyrroline-5-carboxylate reductase-like
0.86	SMARCC2	Memczak2013 ANTISENSE. CDS. coding. INTERNAL best transcript NM_003075
0.86	TAF11	TAF11 RNA polymerase II. TATA box binding protein (TBP)-associated factor. 28kDa
0.86	DLX6	distal-less homeobox 6
0.86	IRGC	immunity-related GTPase family. cinema
0.86	NEU2	sialidase 2 (cytosolic sialidase)
0.86	APBA1	amyloid beta (A4) precursor protein-binding. family A. member 1
0.86	AC009133.12; PAGR1	novel transcript. antisense to C16orf53 and MVP; PAXIP1 associated glutamate-rich protein 1 [Source:HGNC Symbol;Acc:HGNC:28707]
0.86	SLC5A11	solute carrier family 5 (sodium/inositol cotransporter). member 11
0.86	RAD51AP2	Transcript Identified by AceView. Entrez Gene ID(s) 729475
0.86	WDTC1	WD and tetratricopeptide repeats 1

0.86	CXXC1	CXXC finger protein 1
0.86	OR10G8	olfactory receptor. family 10. subfamily G. member 8
0.86	AKAP5	A kinase (PRKA) anchor protein 5
0.86	KIAA1614	KIAA1614
0.86	CASP14	caspase 14
0.86	BTNL10	butyrophilin-like 10
0.86	ZSWIM7	zinc finger. SWIM-type containing 7
0.86	CCDC189	coiled-coil domain containing 189
0.86	SLC28A3	solute carrier family 28 (concentrative nucleoside transporter). member 3
0.86	METTL16	methyltransferase like 16
0.86	ASB12	ankyrin repeat and SOCS box containing 12
0.86	PITPNB	Memczak2013 ANTISENSE. coding. INTERNAL. intronic best transcript NM_012399
0.86	PRF1	perforin 1 (pore forming protein)
0.86	PIDD1	p53-induced death domain protein 1
0.86	C1QA	complement component 1. q subcomponent. A chain
0.86	TSC2	tuberous sclerosis 2
0.86	SULT1A2	sulfotransferase family 1A member 2
0.86	KRTAP24-1	keratin associated protein 24-1
0.86	RHBDL3	rhomboid. veinlet-like 3 (Drosophila)
0.86	LRSAM1	leucine rich repeat and sterile alpha motif containing 1
0.86	LRRC55	leucine rich repeat containing 55
0.86	CENPB	centromere protein B
0.86	DLX3	distal-less homeobox 3
0.86	C7orf60	chromosome 7 open reading frame 60
0.86	ING2	inhibitor of growth family member 2

0.86	MAFIP; TEKT4P2	MAFF interacting protein (pseudogene); tektin 4 pseudogene 2
0.86	MLYCD	malonyl-CoA decarboxylase
0.86	SLCO1B7; SLCO1B1	solute carrier organic anion transporter family. member 1B7 (non-functional); solute carrier organic anion transporter family. member 1B1
0.86	APOC4-APOC2; APOC2	APOC4-APOC2 readthrough (NMD candidate); apolipoprotein C-II
0.86	NR3C2	nuclear receptor subfamily 3. group C. member 2
0.86	PAAF1	proteasomal ATPase-associated factor 1
0.86	ZMYND19	zinc finger. MYND-type containing 19
0.86	HOXA2	homeobox A2
0.86	TRIM63	tripartite motif containing 63. E3 ubiquitin protein ligase
0.86	CHD6	chromodomain helicase DNA binding protein 6
0.86	TGM6	transglutaminase 6
0.86	ULK4P1; ULK4P2	ULK4 pseudogene 1; ULK4 pseudogene 2
0.86	RND2	Rho family GTPase 2
0.86	UNC5A	unc-5 netrin receptor A
0.86	ZAR1L	zygote arrest 1-like
0.86	OR5AU1	olfactory receptor. family 5. subfamily AU. member 1
0.86	MAPK14	mitogen-activated protein kinase 14
0.86	AAGAB	Transcript Identified by AceView. Entrez Gene ID(s) 79719
0.86	ZHX3	zinc fingers and homeoboxes 3
0.86	TRMO	tRNA methyltransferase O
0.86	C2orf27A	chromosome 2 open reading frame 27A
0.86	MLN	motilin
0.86	ARHGAP9	Rho GTPase activating protein 9
0.86	BRD9	bromodomain containing 9
0.86	C14orf177	chromosome 14 open reading frame 177

0.86	sept-01	septin 1
0.86	SLC25A27	solute carrier family 25. member 27
0.86	ACADL	acyl-CoA dehydrogenase. long chain
0.86	FBL	fibrillarin
0.86	MRPS36	mitochondrial ribosomal protein S36
0.86	WDSUB1	WD repeat. sterile alpha motif and U-box domain containing 1
0.86	FRS3	fibroblast growth factor receptor substrate 3
0.86	FAM136A	family with sequence similarity 136. member A
0.86	CYP4F12	cytochrome P450. family 4. subfamily F. polypeptide 12
0.86	TRMT2B	tRNA methyltransferase 2 homolog B
0.86	INPP5J	inositol polyphosphate-5-phosphatase J
0.86	SYAP1	synapse associated protein 1
0.86	PRKCG	protein kinase C. gamma
0.86	C10orf111	chromosome 10 open reading frame 111
0.86	PLD2	phospholipase D2
0.86	FABP6	Memczak2013 ANTISENSE. coding. INTERNAL. intronic best transcript NM_001040442
0.86	TLR7	toll-like receptor 7
0.86	FAM220A	family with sequence similarity 220. member A
0.86	MFSD9	major facilitator superfamily domain containing 9
0.86	TSC22D1	TSC22 domain family. member 1
0.86	TMEM41A	transmembrane protein 41A
0.86	MYO6	Transcript Identified by AceView. Entrez Gene ID(s) 4646
0.86	VEGFB	vascular endothelial growth factor B
0.86	SNRPF	small nuclear ribonucleoprotein polypeptide F
0.86	CX3CR1	chemokine (C-X3-C motif) receptor 1

0.86	NAT8B	N-acetyltransferase 8B (GCN5-related. putative. gene/pseudogene)
0.86	CDK19	cyclin-dependent kinase 19
0.86	VPS13C	Memczak2013 ANTISENSE. coding. INTERNAL. intronic best transcript NM_020821
0.86	CYP1A2	cytochrome P450. family 1. subfamily A. polypeptide 2
0.86	SLC30A6	solute carrier family 30 (zinc transporter). member 6
0.86	GUSBP2	glucuronidase. beta pseudogene 2
0.86	OSBPL2	oxysterol binding protein-like 2
0.86	ZBTB3	zinc finger and BTB domain containing 3
0.86	C6orf106	chromosome 6 open reading frame 106
0.86	PCDH10	protocadherin 10
0.86	SLC6A19	solute carrier family 6 (neutral amino acid transporter). member 19
0.86	EXOC4	exocyst complex component 4
0.86	THTPA	thiamine triphosphatase
0.86	ZBTB22	zinc finger and BTB domain containing 22
0.86	GOLGA6L22	golgin A6 family-like 22
0.86	GALR1	galanin receptor 1
0.86	BSCL2	Berardinelli-Seip congenital lipodystrophy 2 (seipin)
0.86	ALYREF	Aly/REF export factor
0.86	SPCS1	signal peptidase complex subunit 1
0.86	C6orf226	chromosome 6 open reading frame 226
0.86	DNPH1	2-deoxynucleoside 5-phosphate N-hydrolase 1
0.86	ERLIN2	ER lipid raft associated 2
0.86	COX10	COX10 heme A:farnesyltransferase cytochrome c oxidase assembly factor
0.86	CEP55	centrosomal protein 55kDa
0.86	AIRE	autoimmune regulator

0.86	DNAL4	dynein. axonemal. light chain 4
0.86	C15orf48; MIR147B	chromosome 15 open reading frame 48; microRNA 147b
0.86	DAPP1	dual adaptor of phosphotyrosine and 3-phosphoinositides
0.86	FMO6P	flavin containing monooxygenase 6 pseudogene
0.86	AGTR1	angiotensin II receptor. type 1
0.86	TCTN3	tectonic family member 3
0.86	METTL7B	methyltransferase like 7B
0.86	OR9Q2	olfactory receptor. family 9. subfamily Q. member 2
0.86	PTN	pleiotrophin
0.86	CTNND2	catenin (cadherin-associated protein). delta 2
0.86	SLC36A1	solute carrier family 36 (proton/amino acid symporter). member 1
0.86	UBL7	ubiquitin-like 7
0.86	CSN2	casein beta
0.86	STRADB	STE20-related kinase adaptor beta
0.86	NUDT15	nudix hydrolase 15
0.86	ZBTB17	zinc finger and BTB domain containing 17
0.86	RASSF1-AS1	RASSF1 antisense RNA 1
0.86	APBB2	amyloid beta (A4) precursor protein-binding. family B. member 2
0.86	KLHL30	kelch-like family member 30
0.86	SP2	Sp2 transcription factor
0.86	SPI1	Spi-1 proto-oncogene
0.86	SMIM6	small integral membrane protein 6
0.86	ADGRA3	adhesion G protein-coupled receptor A3
0.86	KIF18B	Memczak2013 ALT_ACCEPTOR. coding. INTERNAL. intronic best transcript NM_001080443
0.86	WIPI1	WD repeat domain. phosphoinositide interacting 1

0.86	RNF183	ring finger protein 183
0.86	FMR1	fragile X mental retardation 1
0.86	RGS14	regulator of G-protein signaling 14
0.86	GNB2	guanine nucleotide binding protein (G protein). beta polypeptide 2
0.86	RNF185	Transcript Identified by AceView. Entrez Gene ID(s) 91445
0.86	GDE1	glycerophosphodiester phosphodiesterase 1
0.86	KLHL26	kelch-like family member 26
0.86	FAM83E	family with sequence similarity 83. member E
0.86	UPK3B	uroplakin 3B
0.86	PPP1R27	protein phosphatase 1. regulatory subunit 27
0.86	RRH	retinal pigment epithelium-derived rhodopsin homolog
0.86	ARGLU1	arginine and glutamate rich 1
0.86	POLR1D	polymerase (RNA) I polypeptide D
0.86	DNAI1	dynein. axonemal. intermediate chain 1
0.86	MRPL34	mitochondrial ribosomal protein L34
0.86	PARP10	poly(ADP-ribose) polymerase family member 10
0.86	DIXDC1	DIX domain containing 1
0.86	MEGF9	multiple EGF-like-domains 9
0.86	EME2	essential meiotic structure-specific endonuclease subunit 2
0.86	POLQ	polymerase (DNA directed). theta
0.86	ZNF141	zinc finger protein 141
0.86	MYBPH	myosin binding protein H
0.86	UNC93B1	unc-93 homolog B1 (C. elegans)
0.86	IMPA2	inositol(myo)-1(or 4)-monophosphatase 2
0.86	GCG	glucagon
0.86	PRM1	protamine 1

0.86	H2AFJ	H2A histone family. member J
0.86	EMSY	EMSY BRCA2-interacting transcriptional repressor
0.86	SLC25A23	solute carrier family 25 (mitochondrial carrier; phosphate carrier). member 23
0.86	C4orf48	chromosome 4 open reading frame 48
0.86	LOC100130705; RP11-309L24.4	uncharacterized LOC100130705; putative novel transcript
0.86	TNK1	tyrosine kinase. non-receptor. 1
0.86	ST6GALNAC6	ST6 (alpha-N-acetyl-neuraminyl-2.3-beta-galactosyl-1.3)-N-acetylgalactosaminide alpha-2.6-sialyltransferase 6
0.86	FAM208A	family with sequence similarity 208. member A
0.86	RPL4	ribosomal protein L4
0.86	MED27	mediator complex subunit 27
0.86	SLC25A26	solute carrier family 25 (S-adenosylmethionine carrier). member 26
0.86	C17orf64	chromosome 17 open reading frame 64
0.86	NUMB	numb homolog (Drosophila)
0.86	TYSND1	trypsin domain containing 1
0.86	LRG1	leucine-rich alpha-2-glycoprotein 1
0.86	BARD1	BRCA1 associated RING domain 1
0.86	NCAPH2	non-SMC condensin II complex subunit H2
0.86	PPM1H	protein phosphatase. Mg2+/Mn2+ dependent. 1H
0.86	GPR4	G protein-coupled receptor 4
0.86	DNAJC5G	DnaJ (Hsp40) homolog. subfamily C. member 5 gamma
0.86	STAT1	signal transducer and activator of transcription 1
0.86	DCLRE1C	DNA cross-link repair 1C
0.86	DHFRL1	dihydrofolate reductase like 1
0.86	TBX21	T-box 21

0.86	SCARF1	scavenger receptor class F. member 1
0.86	CD99	Homo sapiens CD99 molecule (CD99). transcript variant 2. mRNA.; Homo sapiens CD99 molecule (CD99). transcript variant 3. mRNA.; Homo sapiens CD99 molecule (CD99). transcript variant 1. mRNA.; CD99 molecule [Source:HGNC Symbol;Acc:HGNC:7082]; Homo sapiens CD99 molecule. mRNA (cDNA clone MGC:2282 IMAGE:3161673). complete cds.; Homo sapiens CD99 molecule. mRNA (cDNA clone MGC:3938 IMAGE:2906143). complete cds.; Homo sapiens CD99 molecule. mRNA (cDNA clone MGC:19734 IMAGE:3606974). complete cds.; Homo sapiens CD99 molecule. mRNA (cDNA clone MGC:4214 IMAGE:2957883). complete cds.; Homo sapiens CD99 molecule. mRNA (cDNA clone MGC:14521 IMAGE:4094268). complete cds.; Salzman2013 ANNOTATED. CDS. coding. INTERNAL. OVCODE. OVEXON best transcript NM_002414; Salzman2013 ANNOTATED. CDS. coding. OVCODE. OVEXON. UTR3 best transcript NM_002414
0.86	CCDC179	coiled-coil domain containing 179
0.86	ADAMTSL2	ADAMTS like 2
0.86	CYB561	cytochrome b561
0.86	C16orf58	chromosome 16 open reading frame 58
0.86	PIGT	phosphatidylinositol glycan anchor biosynthesis class T
0.86	OR11H6	olfactory receptor. family 11. subfamily H. member 6
0.86	COX1	cytochrome c oxidase subunit I
0.86	EMC10	ER membrane protein complex subunit 10
0.86	OTOG	otogelin
0.86	CD28	CD28 molecule
0.86	APOM	apolipoprotein M
0.86	CEP41	centrosomal protein 41kDa
0.86	OVGP1	oviductal glycoprotein 1
0.86	NEO1	neogenin 1
0.86	WDR26	Transcript Identified by AceView. Entrez Gene ID(s) 80232
0.86	POLR2H	polymerase (RNA) II (DNA directed) polypeptide H

0.86	TM9SF2	transmembrane 9 superfamily member 2
0.86	PRDX5	peroxiredoxin 5
0.86	JCHAIN	joining chain of multimeric IgA and IgM
0.86	PAGE3	P antigen family. member 3 (prostate associated)
0.86	ARMC2	armadillo repeat containing 2
0.86	VCY1B	variable charge. Y-linked 1B
0.86	IL10RB	interleukin 10 receptor. beta
0.86	LMAN2	lectin. mannose-binding 2
0.86	ZNF764	zinc finger protein 764
0.86	DEFB114	defensin. beta 114
0.86	TMEM81	transmembrane protein 81
0.86	SLC18A2	solute carrier family 18 (vesicular monoamine transporter). member 2
0.86	LIMCH1	LIM and calponin homology domains 1
0.86	CBR3	carbonyl reductase 3
0.86	GOLGA2P5	golgin A2 pseudogene 5
0.86	HTR3E	5-hydroxytryptamine (serotonin) receptor 3E. ionotropic
0.86	OR8B2	olfactory receptor. family 8. subfamily B. member 2
0.86	ADIPOR1	adiponectin receptor 1
0.86	PRR15L	proline rich 15-like
0.86	HHATL	hedgehog acyltransferase-like
0.86	OR5K4	olfactory receptor. family 5. subfamily K. member 4
0.86	VPS26B	VPS26 retromer complex component B
0.86	EXOSC3	exosome component 3
0.86	CGB8	chorionic gonadotropin. beta polypeptide 8
0.86	TMEM201	transmembrane protein 201
0.86	HMBS	hydroxymethylbilane synthase

0.86	PHF14	PHD finger protein 14
0.86	C1QTNF5; MFRP	C1q and tumor necrosis factor related protein 5; membrane frizzled-related protein
0.86	CYFIP2	cytoplasmic FMR1 interacting protein 2
0.86	SRRM1	serine/arginine repetitive matrix 1
0.86	NCBP1	nuclear cap binding protein subunit 1
0.86	HBE1	hemoglobin. epsilon 1
0.86	PRKAB1	protein kinase. AMP-activated. beta 1 non-catalytic subunit
0.86	BNIPL	BCL2/adenovirus E1B 19kD interacting protein like
0.86	PSG1	pregnancy specific beta-1-glycoprotein 1
0.86	PIK3CG	Memczak2013 ANTISENSE. CDS. coding. INTERNAL best transcript NM_002649
0.85	NOL7	nucleolar protein 7
0.85	HSPB1	heat shock 27kDa protein 1
0.85	DNAJB1	DnaJ (Hsp40) homolog. subfamily B. member 1
0.85	ALG12	ALG12. alpha-1.6-mannosyltransferase
0.85	PDE10A	phosphodiesterase 10A
0.85	SEMA4A	sema domain. immunoglobulin domain (Ig). transmembrane domain (TM) and short cytoplasmic domain. (semaphorin) 4A
0.85	MPC1	mitochondrial pyruvate carrier 1
0.85	RNF217	ring finger protein 217
0.85	ATXN1L	ataxin 1-like
0.85	BET1	Bet1 golgi vesicular membrane trafficking protein
0.85	SEC24A	SEC24 homolog A. COPII coat complex component
0.85	MOXD1	monooxygenase. DBH-like 1
0.85	PAPLN	papilin. proteoglycan-like sulfated glycoprotein
0.85	GRXCR2	glutaredoxin. cysteine rich 2

0.85	COL24A1	collagen. type XXIV. alpha 1
0.85	ANKFY1	ankyrin repeat and FYVE domain containing 1
0.85	NME9	NME/NM23 family member 9
0.85	TARSL2	threonyl-tRNA synthetase-like 2
0.85	SNRPD2P2	small nuclear ribonucleoprotein D2 pseudogene 2
0.85	DTWD2	DTW domain containing 2
0.85	FAM43B	family with sequence similarity 43. member B
0.85	DOCK9	dedicator of cytokinesis 9
0.85	TNP2	transition protein 2 (during histone to protamine replacement)
0.85	KIRREL3	kin of IRRE like 3 (Drosophila)
0.85	FMO5	flavin containing monooxygenase 5
0.85	PRELP	proline/arginine-rich end leucine-rich repeat protein
0.85	KRTAP9-3	keratin associated protein 9-3
0.85	NGFR	nerve growth factor receptor
0.85	CKLF; CKLF-CMTM1	chemokine-like factor; CKLF-CMTM1 readthrough
0.85	MGAT5B	mannosyl (alpha-1.6-)-glycoprotein beta-1.6-N-acetyl-glucosaminyltransferase. isozyme B
0.85	SEC13	SEC13 homolog. nuclear pore and COPII coat complex component
0.85	CPEB3	Transcript Identified by AceView. Entrez Gene ID(s) 22849
0.85	MED1	mediator complex subunit 1
0.85	NINL	Zhang2013 ALT_ACCEPTOR. ALT_DONOR. coding. INTERNAL. intronic best transcript NM_025176
0.85	RNF215	ring finger protein 215
0.85	DYRK1B	dual specificity tyrosine-(Y)-phosphorylation regulated kinase 1B
0.85	SURF1	surfeit 1
0.85	SEC16A	SEC16 homolog A. endoplasmic reticulum export factor
0.85	LINC00602	long intergenic non-protein coding RNA 602

0.85	HMCN2	hemicentin 2
0.85	MGEA5	Salzman2013 ALT_ACCEPTOR. ALT_DONOR. coding. INTERNAL. intronic best transcript NM_012215
0.85	OR10G7	olfactory receptor. family 10. subfamily G. member 7
0.85	TTC8	Transcript Identified by AceView. Entrez Gene ID(s) 123016
0.85	SAFB	scaffold attachment factor B
0.85	LINC01342	long intergenic non-protein coding RNA 1342
0.85	WDR83OS	WD repeat domain 83 opposite strand
0.85	TMEM86A	transmembrane protein 86A
0.85	UBTD1	ubiquitin domain containing 1
0.85	ZSCAN2	zinc finger and SCAN domain containing 2
0.85	CGREF1	cell growth regulator with EF-hand domain 1
0.85	SPRTN	SprT-like N-terminal domain
0.85	ZKSCAN7	zinc finger with KRAB and SCAN domains 7
0.85	IGSF11	immunoglobulin superfamily. member 11
0.85	DENND3	Memczak2013 ALT_ACCEPTOR. ALT_DONOR. coding. INTERNAL. intronic best transcript NM_014957
0.85	RXRA; MIR4669	retinoid X receptor alpha; microRNA 4669
0.85	FAT4	FAT atypical cadherin 4
0.85	PGM3	phosphoglucomutase 3
0.85	LMTK3	lemur tyrosine kinase 3
0.85	TIPARP	TCDD-inducible poly(ADP-ribose) polymerase
0.85	IL23A	interleukin 23. alpha subunit p19
0.85	VDR	vitamin D (1.25- dihydroxyvitamin D3) receptor
0.85	SLC25A4	solute carrier family 25 (mitochondrial carrier; adenine nucleotide translocator). member 4
0.85	OR6A2	olfactory receptor. family 6. subfamily A. member 2

0.85	SETD7	SET domain containing (lysine methyltransferase) 7
0.85	LY6K	lymphocyte antigen 6 complex. locus K
0.85	MRPS7	mitochondrial ribosomal protein S7
0.85	CDCA4	cell division cycle associated 4
0.85	SKP1	S-phase kinase-associated protein 1
0.85	LINC00504	long intergenic non-protein coding RNA 504
0.85	SRR	serine racemase
0.85	UAP1	UDP-N-acetylglucosamine pyrophosphorylase 1
0.85	RIPPLY2	ripply transcriptional repressor 2
0.85	ZNF557	zinc finger protein 557
0.85	KCNA10	potassium channel. voltage gated shaker related subfamily A. member 10
0.85	ZCCHC5	zinc finger. CCHC domain containing 5
0.85	TNNT1	troponin T type 1 (skeletal. slow)
0.85	ESRRB	estrogen-related receptor beta
0.85	LRRC14B	leucine rich repeat containing 14B
0.85	OR4F29; OR4F3; OR4F16	olfactory receptor. family 4. subfamily F. member 29; olfactory receptor. family 4. subfamily F. member 3; olfactory receptor. family 4. subfamily F. member 16
0.85	OR4F16; OR4F29	olfactory receptor. family 4. subfamily F. member 16; olfactory receptor. family 4. subfamily F. member 29
0.85	MRPL28	mitochondrial ribosomal protein L28
0.85	HERC6	HECT and RLD domain containing E3 ubiquitin protein ligase family member 6
0.85	PLK1	polo-like kinase 1
0.85	DCTPP1	dCTP pyrophosphatase 1
0.85	PPP1R16A	protein phosphatase 1. regulatory subunit 16A
0.85	HSD17B12	hydroxysteroid (17-beta) dehydrogenase 12

0.85	ORAI3	ORAI calcium release-activated calcium modulator 3
0.85	ARMC7	armadillo repeat containing 7
0.85	OR10A7	olfactory receptor. family 10. subfamily A. member 7
0.85	LOC79999; LOC388436; AC007952.5	Homo sapiens uncharacterized LOC79999 (LOC79999). mRNA.; uncharacterized protein ENSP00000382042; novel transcript
0.85	FLI1	Fli-1 proto-oncogene. ETS transcription factor
0.85	CIB1	calcium and integrin binding 1 (calmyrin)
0.85	DFFB	DNA fragmentation factor. 40kDa. beta polypeptide (caspase-activated DNase)
0.85	FAM153A	family with sequence similarity 153. member A
0.85	CD53	CD53 molecule
0.85	TMEM130	transmembrane protein 130
0.85	GRAMD1C	GRAM domain containing 1C
0.85	LRIG1	leucine-rich repeats and immunoglobulin-like domains 1
0.85	VWCE	von Willebrand factor C and EGF domains
0.85	RAB1B	RAB1B. member RAS oncogene family
0.85	NADK	NAD kinase
0.85	DEFB124	defensin. beta 124
0.85	PLOD2	procollagen-lysine. 2-oxoglutarate 5-dioxygenase 2
0.85	CCHCR1	coiled-coil alpha-helical rod protein 1
0.85	MTURN	maturin. neural progenitor differentiation regulator homolog (Xenopus)
0.85	MFAP2	microfibrillar associated protein 2
0.85	C17orf62	chromosome 17 open reading frame 62
0.85	LIMS3L; LIMS3	LIM and senescent cell antigen-like domains 3-like; LIM and senescent cell antigen-like domains 3
0.85	AMN	amnion associated transmembrane protein
0.85	HLA-DRB5	major histocompatibility complex. class II. DR beta 5

0.85	KLHL21	kelch-like family member 21
0.85	MAPK1	mitogen-activated protein kinase 1
0.85	HIST1H2BD	histone cluster 1. H2bd
0.85	B3GALT5	UDP-Gal:betaGlcNAc beta 1.3-galactosyltransferase 5
0.85	NR1H3	nuclear receptor subfamily 1. group H. member 3
0.85	INSL3	insulin-like 3 (Leydig cell)
0.85	OPRD1	opioid receptor. delta 1
0.85	ZNF98	zinc finger protein 98
0.85	IL12RB1	interleukin 12 receptor. beta 1
0.85	SLC2A10	solute carrier family 2 (facilitated glucose transporter). member 10
0.85	TUB	tubby bipartite transcription factor
0.85	RHOBTB2	Rho-related BTB domain containing 2
0.85	RNF8	ring finger protein 8. E3 ubiquitin protein ligase
0.85	SHROOM1	shroom family member 1
0.85	SLBP	stem-loop binding protein
0.85	CELF3	CUGBP. Elav-like family member 3
0.85	CISD3	CDGSH iron sulfur domain 3
0.85	PTPRN2	protein tyrosine phosphatase. receptor type. N polypeptide 2
0.85	PPARGC1B	peroxisome proliferator-activated receptor gamma. coactivator 1 beta
0.85	ARHGAP24	Rho GTPase activating protein 24
0.85	FBN1	fibrillin 1
0.85	IQCA1L	IQ motif containing with AAA domain 1 like
0.85	ZNF771	zinc finger protein 771
0.85	OR2B3	olfactory receptor. family 2. subfamily B. member 3
0.85	TMEM255A	transmembrane protein 255A
0.85	H2AFB1; H2AFB2; H2AFB3	H2A histone family. member B1; H2A histone family. member B2; H2A

		histone family. member B3
0.85	SF3B2	splicing factor 3b subunit 2
0.85	ZNF490	zinc finger protein 490
0.85	MS4A3	membrane-spanning 4-domains. subfamily A. member 3 (hematopoietic cell-specific)
0.85	LENEP	lens epithelial protein
0.85	GPR183	G protein-coupled receptor 183
0.85	MYT1L	myelin transcription factor 1-like
0.85	KCNA2	potassium channel. voltage gated shaker related subfamily A. member 2
0.85	ARID3C	AT rich interactive domain 3C (BRIGHT-like)
0.85	HIST1H3E	histone cluster 1. H3e
0.85	sept-11	septin 11
0.85	HOXD12	homeobox D12
0.85	RHOT2	ras homolog family member T2
0.85	C20orf27	chromosome 20 open reading frame 27
0.85	PCM1	pericentriolar material 1
0.85	UPK1A	uroplakin 1A
0.85	GRHL1	grainyhead-like transcription factor 1
0.85	RNF130	ring finger protein 130
0.85	DEFB116	defensin. beta 116
0.85	GOLGA8R	golgin A8 family. member R
0.85	SUFU	suppressor of fused homolog (Drosophila)
0.85	CLDN25	claudin 25
0.85	MTRNR2L1	MT-RNR2-like 1
0.85	IL6ST	interleukin 6 signal transducer
0.85	TMEM104	transmembrane protein 104

0.85	GOLGA3	golgin A3
0.85	WBP11	WW domain binding protein 11
0.85	CREB3	cAMP responsive element binding protein 3
0.85	BAGE5	Synthetic construct Homo sapiens clone IMAGE:100062553. MGC:190557 B melanoma antigen family. member 5 (BAGE5) mRNA. encodes complete protein.; B melanoma antigen family. member 5 [Source:EntrezGene;Acc:85316]
0.85	TMA7	translation machinery associated 7 homolog
0.85	SRP14	signal recognition particle 14kDa
0.85	NKAIN1	Na+/K+ transporting ATPase interacting 1
0.85	DNAJC3	DnaJ (Hsp40) homolog. subfamily C. member 3
0.85	TMEM8B	transmembrane protein 8B
0.85	TAF5	TAF5 RNA polymerase II. TATA box binding protein (TBP)-associated factor. 100kDa
0.85	TMEM223	transmembrane protein 223
0.85	ITGB7	integrin beta 7
0.85	CD70	CD70 molecule
0.85	LAG3	lymphocyte-activation gene 3
0.85	HOXD9	homeobox D9
0.85	OR7D2	olfactory receptor. family 7. subfamily D. member 2
0.85	SETD1A	SET domain containing 1A
0.85	COBL	cordon-bleu WH2 repeat protein
0.85	SLC13A1	solute carrier family 13 (sodium/sulfate symporter). member 1
0.85	CA4	carbonic anhydrase IV
0.85	LMO7	LIM domain 7
0.85	EP400NL	EP400 N-terminal like
0.85	HAUS4; MIR4707	HAUS augmin like complex subunit 4; microRNA 4707

0.85	AKR1B1	aldo-keto reductase family 1. member B1 (aldose reductase)
0.85	ACCS	1-aminocyclopropane-1-carboxylate synthase homolog (inactive)
0.85	CXorf40B	chromosome X open reading frame 40B
0.85	SH2B2	Memczak2013 ANTISENSE. CDS. coding. INTERNAL best transcript NM_020979
0.85	MTERF2	mitochondrial transcription termination factor 2
0.85	NPHP3-ACAD11	NPHP3-ACAD11 readthrough (NMD candidate)
0.85	PTGER1	prostaglandin E receptor 1
0.85	TRIM45	tripartite motif containing 45
0.85	ZHX2	zinc fingers and homeoboxes 2
0.85	LYSMD1	LysM. putative peptidoglycan-binding. domain containing 1
0.85	SCX	scleraxis bHLH transcription factor
0.85	PRR14	proline rich 14
0.85	CCDC70	coiled-coil domain containing 70
0.85	NR2C2	Transcript Identified by AceView. Entrez Gene ID(s) 7182
0.85	LSM7	LSM7 homolog. U6 small nuclear RNA and mRNA degradation associated
0.85	HDLBP	Transcript Identified by AceView. Entrez Gene ID(s) 3069
0.85	CD99	CD99 molecule
0.85	PHACTR4	phosphatase and actin regulator 4
0.85	TMED9	transmembrane p24 trafficking protein 9
0.85	MYCT1	myc target 1
0.85	SLX1A; SLX1B	SLX1 homolog A. structure-specific endonuclease subunit; SLX1 homolog B. structure-specific endonuclease subunit
0.85	ZSCAN18	zinc finger and SCAN domain containing 18
0.85	VPS11	VPS11. CORVET/HOPS core subunit [Source:HGNC Symbol;Acc:HGNC:14583]
0.85	TNS1	tensin 1
0.85	SPACA3	sperm acrosome associated 3

0.85	DIRAS2	DIRAS family. GTP-binding RAS-like 2
0.85	PENK	proenkephalin
0.85	ZPBP2	zona pellucida binding protein 2
0.85	NUDT8	nudix hydrolase 8
0.85	UNC45A	unc-45 myosin chaperone A
0.85	FAM98B	family with sequence similarity 98. member B
0.85	NUCB1	nucleobindin 1
0.85	SLC7A14	solute carrier family 7. member 14
0.85	FEM1C	fem-1 homolog c (C. elegans)
0.85	ZC3H3	zinc finger CCCH-type containing 3
0.85	NEK6	NIMA-related kinase 6
0.85	RASAL3	RAS protein activator like 3
0.85	FLRT2	fibronectin leucine rich transmembrane protein 2
0.85	CPSF7	cleavage and polyadenylation specific factor 7
0.85	MGC45922; CTD-2568A17.5	uncharacterized LOC284365; uncharacterized LOC284365 [Source:EntrezGene;Acc:284365]; novel transcript
0.85	ZNF544	zinc finger protein 544
0.85	OTOA	otoancorin
0.85	CCDC60	coiled-coil domain containing 60
0.85	TTF2	transcription termination factor. RNA polymerase II
0.85	SCRT1	scratch family zinc finger 1
0.85	TLR10	toll-like receptor 10
0.85	SRGN	Memczak2013 ANTISENSE. coding. INTERNAL. UTR3 best transcript NM_002727
0.85	TAC4	tachykinin 4 (hemokinin)
0.85	GPR162	G protein-coupled receptor 162
0.85	VSTM4	V-set and transmembrane domain containing 4

0.85	ZNF283	zinc finger protein 283
0.85	CDX2	caudal type homeobox 2
0.85	RAB5B	RAB5B. member RAS oncogene family
0.85	SFXN1	sideroflexin 1
0.85	PPM1J	protein phosphatase. Mg2+/Mn2+ dependent. 1J
0.85	THEGL	theg spermatid protein-like
0.85	CASR	calcium-sensing receptor
0.85	NOVA2	neuro-oncological ventral antigen 2
0.85	MS4A18	membrane-spanning 4-domains. subfamily A. member 18
0.85	CST6	cystatin E/M
0.85	CCDC64B	coiled-coil domain containing 64B
0.85	CYP3A4	cytochrome P450. family 3. subfamily A. polypeptide 4
0.85	PCCB	propionyl-CoA carboxylase beta subunit
0.85	GABRG1	gamma-aminobutyric acid (GABA) A receptor. gamma 1
0.85	RELA	v-rel avian reticuloendotheliosis viral oncogene homolog A
0.85	DNAI1	Transcript Identified by AceView. Entrez Gene ID(s) 27019
0.85	PGM2L1	phosphoglucomutase 2-like 1
0.85	BNC2	basonuclin 2
0.85	C21orf33	chromosome 21 open reading frame 33
0.85	HTR2C	5-hydroxytryptamine (serotonin) receptor 2C. G protein-coupled
0.85	THYN1	thymocyte nuclear protein 1
0.85	SMARCA2	SWI/SNF related. matrix associated. actin dependent regulator of chromatin. subfamily a. member 2
0.85	KLHL29	kelch-like family member 29
0.85	TRABD2A	TraB domain containing 2A
0.85	ZNF561	zinc finger protein 561

0.85	STARD10	StAR-related lipid transfer domain containing 10
0.85	WBP5	WW domain binding protein 5
0.85	TP53I11	Memczak2013 ALT_ACCEPTOR. ALT_DONOR. coding. INTERNAL. intronic best transcript NM_001076787
0.85	FAM101A	family with sequence similarity 101. member A
0.85	IRF1	interferon regulatory factor 1
0.85	ERP29	endoplasmic reticulum protein 29
0.85	QRFP	pyroglutamylated RFamide peptide
0.85	STK16	serine/threonine kinase 16
0.85	C21orf140	chromosome 21 open reading frame 140
0.85	C21orf140	chromosome 21 open reading frame 140
0.85	IYD	iodotyrosine deiodinase
0.85	TNFRSF18	tumor necrosis factor receptor superfamily. member 18
0.85	ABCC12	ATP binding cassette subfamily C member 12
0.85	SERPINA11	serpin peptidase inhibitor. clade A (alpha-1 antiproteinase. antitrypsin). member 11
0.85	SON; MIR6501	SON DNA binding protein; microRNA 6501
0.85	MRPL2	mitochondrial ribosomal protein L2
0.85	C14orf180	chromosome 14 open reading frame 180
0.85	PEPD	peptidase D
0.85	SPDYE2; SPDYE2B	speedy/RINGO cell cycle regulator family member E2; speedy/RINGO cell cycle regulator family member E2B
0.85	SPDYE2B	speedy/RINGO cell cycle regulator family member E2B
0.85	KSR2	kinase suppressor of ras 2
0.85	GSN	gelsolin
0.85	IDNK	idnK. gluconokinase homolog (E. coli)
0.85	DPH3	diphthamide biosynthesis 3

0.85	KCNJ8	potassium channel. inwardly rectifying subfamily J. member 8
0.85	RAG1	recombination activating gene 1
0.85	CLCN2	chloride channel. voltage-sensitive 2
0.85	CD82	CD82 molecule
0.85	NEUROD2	neuronal differentiation 2
0.85	DHTKD1	dehydrogenase E1 and transketolase domain containing 1
0.85	VCY; VCY1B	variable charge. Y-linked; variable charge. Y-linked 1B
0.85	VPS8	Transcript Identified by AceView. Entrez Gene ID(s) 23355
0.85	SLA2	Src-like-adaptor 2
0.85	CHRNB2	cholinergic receptor. nicotinic beta 2
0.85	CLN5	ceroid-lipofuscinosis. neuronal 5
0.85	DHX58	DEXH (Asp-Glu-X-His) box polypeptide 58
0.85	WBSCR27	Williams Beuren syndrome chromosome region 27
0.85	TMEM52B	transmembrane protein 52B
0.85	DACH2	dachshund family transcription factor 2
0.85	LMX1B	LIM homeobox transcription factor 1. beta
0.85	PAK4	p21 protein (Cdc42/Rac)-activated kinase 4
0.85	CCDC187	coiled-coil domain containing 187
0.85	COX6B1	cytochrome c oxidase subunit VIb polypeptide 1 (ubiquitous)
0.85	LYRM9	LYR motif containing 9
0.85	BLOC1S6	biogenesis of lysosomal organelles complex-1. subunit 6. pallidin
0.85	SEMA3G	sema domain. immunoglobulin domain (Ig). short basic domain. secreted. (semaphorin) 3G
0.85	PSD2	pleckstrin and Sec7 domain containing 2
0.85	SDR39U1	short chain dehydrogenase/reductase family 39U. member 1
0.85	RP1	retinitis pigmentosa 1 (autosomal dominant)

0.85	DYNLT3	dynein. light chain. Tctex-type 3
0.85	SLC25A36	solute carrier family 25 (pyrimidine nucleotide carrier). member 36
0.85	MICALL2	MICAL-like 2
0.85	TRIM11; MIR6742	tripartite motif containing 11; microRNA 6742
0.85	IKBKB	Transcript Identified by AceView. Entrez Gene ID(s) 3551
0.85	LOC338797; RP13-507P19.2; RP13-507P19.1	uncharacterized LOC338797; novel transcript
0.85	SSTR5	somatostatin receptor 5
0.85	FRS2	fibroblast growth factor receptor substrate 2
0.85	C16orf13	chromosome 16 open reading frame 13
0.85	NEIL2	nei-like DNA glycosylase 2
0.85	VRTN	vertebrae development associated
0.85	CD300LB	CD300 molecule-like family member b
0.85	SIPA1L3	signal-induced proliferation-associated 1 like 3
0.85	TBC1D3K	TBC1 domain family. member 3K
0.85	SLC12A5	solute carrier family 12 (potassium/chloride transporter). member 5
0.85	C1orf162	chromosome 1 open reading frame 162
0.85	ZGLP1	zinc finger. GATA-like protein 1
0.85	COL14A1	collagen. type XIV. alpha 1
0.85	TSPO2	translocator protein 2
0.85	PRH1; TAS2R14; PRR4	proline-rich protein HaeIII subfamily 1; taste receptor. type 2. member 14; proline rich 4 (lacrimal)
0.85	KRTAP10-7; KRTAP10-8	keratin associated protein 10-7; keratin associated protein 10-8
0.85	HPX	hemopexin
0.85	RMND5B	required for meiotic nuclear division 5 homolog B
0.85	FMN2; ADH5P3	formin 2; alcohol dehydrogenase 5 (class III). chi polypeptide. pseudogene 3
0.85	ZNF506	zinc finger protein 506

0.85	PER1; MIR6883	period circadian clock 1; microRNA 6883
0.85	TMEM160	transmembrane protein 160
0.85	CSDC2	cold shock domain containing C2. RNA binding
0.85	MAPK7	mitogen-activated protein kinase 7
0.85	TMEM191C	transmembrane protein 191C
0.85	SYTL1	synaptotagmin like 1
0.85	IFNA13	interferon. alpha 13
0.85	TPI1	triosephosphate isomerase 1
0.85	CARD8	caspase recruitment domain family. member 8
0.85	LOC100144595; AC009227.2; tikure	uncharacterized LOC100144595; novel transcript; Transcript Identified by AceView
0.85	TNRC6B	trinucleotide repeat containing 6B
0.85	SATL1	spermidine/spermine N1-acetyl transferase-like 1
0.85	FAM195B	family with sequence similarity 195. member B
0.85	LOC730668; CITF22-92A6.2	dynein heavy chain -like pseudogene; TEC
0.85	MX1	MX dynamin-like GTPase 1
0.85	ANKMY2	ankyrin repeat and MYND domain containing 2
0.85	APLF	aprataxin and PNKP like factor
0.85	C22orf31	chromosome 22 open reading frame 31
0.85	BTAF1	BTAF1 RNA polymerase II. B-TFIID transcription factor-associated. 170kDa
0.85	TMIGD1	transmembrane and immunoglobulin domain containing 1
0.85	HUNK	hormonally up-regulated Neu-associated kinase
0.85	NPEPL1	aminopeptidase-like 1
0.85	C10orf35	chromosome 10 open reading frame 35
0.85	HMBS	hydroxymethylbilane synthase
0.85	LMNTD2	lamin tail domain containing 2

0.85	TDGF1	teratocarcinoma-derived growth factor 1
0.85	CNN3	calponin 3. acidic
0.85	YIPF2	Yip1 domain family member 2
0.85	H2AFB2	H2A histone family. member B2
0.85	HBA1	hemoglobin. alpha 1
0.85	AMIGO2	adhesion molecule with Ig-like domain 2
0.85	SSBP4	single stranded DNA binding protein 4
0.85	GAS7	growth arrest-specific 7
0.85	THEM6	thioesterase superfamily member 6
0.85	RPP30	ribonuclease P/MRP 30kDa subunit
0.85	SORBS2	Transcript Identified by AceView. Entrez Gene ID(s) 8470
0.85	HMGN2	high mobility group nucleosomal binding domain 2
0.85	MRPS18C	mitochondrial ribosomal protein S18C
0.85	FOXD4L4	forkhead box D4-like 4
0.85	FAM200A	family with sequence similarity 200. member A
0.85	FBXL12	F-box and leucine-rich repeat protein 12
0.85	LTB4R; LTB4R2	leukotriene B4 receptor; leukotriene B4 receptor 2
0.85	SUMF1	sulfatase modifying factor 1
0.85	CLEC3A	C-type lectin domain family 3. member A
0.85	GAS2L1	growth arrest-specific 2 like 1
0.85	RNF122	ring finger protein 122
0.85	CNPY3	canopy FGF signaling regulator 3
0.85	ANP32E	acidic nuclear phosphoprotein 32 family member E
0.85	WDR18	WD repeat domain 18
0.85	RGS17	regulator of G-protein signaling 17
0.85	LANCL3	LanC lantibiotic synthetase component C-like 3 (bacterial)

0.85	MAD2L1	MAD2 mitotic arrest deficient-like 1 (yeast)
0.85	PRDM8	PR domain containing 8
0.85	RP11-12M5.1; FAM163A	Transcript Identified by AceView. Entrez Gene ID(s) 148753; novel transcript
0.85	ADAM29	ADAM metallopeptidase domain 29
0.85	BRICD5	BRICHOS domain containing 5
0.85	HMBS	hydroxymethylbilane synthase
0.85	ZBTB42	zinc finger and BTB domain containing 42
0.85	FAM228A	family with sequence similarity 228. member A
0.85	GDF6	growth differentiation factor 6
0.85	PPP1R17	protein phosphatase 1. regulatory subunit 17
0.85	TOR1AIP2	torsin A interacting protein 2
0.85	USP5	ubiquitin specific peptidase 5 (isopeptidase T)
0.85	GKN2	gastrokine 2
0.84	IQUB	IQ motif and ubiquitin domain containing
0.84	EPN1	epsin 1
0.84	TP53AIP1	tumor protein p53 regulated apoptosis inducing protein 1
0.84	YEATS4	YEATS domain containing 4
0.84	CARTPT	CART prepropeptide
0.84	GIPC1	GIPC PDZ domain containing family. member 1
0.84	SLC39A5	solute carrier family 39 (zinc transporter). member 5
0.84	DHDDS	dehydrodolichyl diphosphate synthase subunit
0.84	LLGL2	lethal giant larvae homolog 2 (Drosophila)
0.84	UBL4B	ubiquitin-like 4B
0.84	TTC23L	tetratricopeptide repeat domain 23-like
0.84	PRDM16	PR domain containing 16
0.84	PPID	peptidylprolyl isomerase D

0.84	FBN2	fibrillin 2
0.84	VPS11	VPS11. CORVET/HOPS core subunit [Source:HGNC Symbol;Acc:HGNC:14583]
0.84	MGLL	monoglyceride lipase
0.84	ZNF579	zinc finger protein 579
0.84	UNCX	UNC homeobox
0.84	GPR61	G protein-coupled receptor 61
0.84	DNMT3B	DNA (cytosine-5-)-methyltransferase 3 beta
0.84	RPS6KA1	ribosomal protein S6 kinase. 90kDa. polypeptide 1
0.84	C5orf52	chromosome 5 open reading frame 52
0.84	DIO3	deiodinase. iodothyronine. type III
0.84	RSPO3	R-spondin 3
0.84	FSTL5	follistatin-like 5
0.84	CENPQ	centromere protein Q
0.84	SPATA31D1	SPATA31 subfamily D. member 1
0.84	GPAM	glycerol-3-phosphate acyltransferase. mitochondrial
0.84	FAM133A	family with sequence similarity 133. member A
0.84	GRIN2B	glutamate receptor. ionotropic. N-methyl D-aspartate 2B
0.84	HRH3	histamine receptor H3
0.84	DNAJC6	DnaJ (Hsp40) homolog. subfamily C. member 6
0.84	FBLN7	fibulin 7
0.84	NUDT22	nudix hydrolase 22
0.84	RAB8B	RAB8B. member RAS oncogene family
0.84	MLLT4	myeloid/lymphoid or mixed-lineage leukemia; translocated to. 4
0.84	ARHGEF18	Rho/Rac guanine nucleotide exchange factor 18
0.84	FGGY	FGGY carbohydrate kinase domain containing
0.84	WDR63	WD repeat domain 63

0.84	GRIK5	glutamate receptor. ionotropic. kainate 5
0.84	MLIP	muscular LMNA-interacting protein
0.84	TMSB15B	thymosin beta 15B
0.84	DDN	dendrin
0.84	RP4-728D4.2; PSMB2	novel transcript; Transcript Identified by AceView. Entrez Gene ID(s) 5690
0.84	CCDC155	coiled-coil domain containing 155
0.84	ZNF234	zinc finger protein 234
0.84	KCNS3	potassium voltage-gated channel. modifier subfamily S. member 3
0.84	AKT2	v-akt murine thymoma viral oncogene homolog 2
0.84	BRINP1	bone morphogenetic protein/retinoic acid inducible neural-specific 1
0.84	NOX3	NADPH oxidase 3
0.84	BUD31	BUD31 homolog
0.84	KAT5	K(lysine) acetyltransferase 5
0.84	CCDC28B	coiled-coil domain containing 28B
0.84	MILR1	mast cell immunoglobulin-like receptor 1
0.84	RNF169	ring finger protein 169
0.84	PFKL	phosphofructokinase. liver
0.84	SNX22	sorting nexin 22
0.84	IGFBP2	insulin like growth factor binding protein 2
0.84	ANKRD26	ankyrin repeat domain 26
0.84	DERL2	derlin 2
0.84	C11orf16	chromosome 11 open reading frame 16
0.84	DECR1	2.4-dienoyl-CoA reductase 1. mitochondrial
0.84	ACTB	actin. beta
0.84	LRP4	LDL receptor related protein 4
0.84	CFDP1	craniofacial development protein 1

0.84	OSBP	oxysterol binding protein
0.84	TMPRSS7	transmembrane protease. serine 7
0.84	TLX1	T-cell leukemia homeobox 1
0.84	CDC40	cell division cycle 40
0.84	CSN3	casein kappa
0.84	GALNT14	polypeptide N-acetylgalactosaminyltransferase 14
0.84	SYS1	Sys1 golgi trafficking protein
0.84	DMBT1	deleted in malignant brain tumors 1
0.84	TKTL1	transketolase-like 1
0.84	PGRMC2	progesterone receptor membrane component 2
0.84	SEC63	SEC63 homolog. protein translocation regulator
0.84	GUCY2D	guanylate cyclase 2D. membrane (retina-specific)
0.84	RFX5	regulatory factor X. 5 (influences HLA class II expression)
0.84	TNNI2	troponin I type 2 (skeletal. fast)
0.84	CNN2	calponin 2
0.84	TIMELESS	timeless circadian clock
0.84	ZP3	zona pellucida glycoprotein 3 (sperm receptor)
0.84	C16orf74	chromosome 16 open reading frame 74
0.84	SPACA6	sperm acrosome associated 6
0.84	MTX1	metaxin 1
0.84	C19orf73	chromosome 19 open reading frame 73
0.84	POTEB3	POTE ankyrin domain family. member B3
0.84	TMEM169	transmembrane protein 169
0.84	NUDCD2	NudC domain containing 2
0.84	MRGPRG	MAS-related GPR. member G
0.84	MSANTD1	Myb/SANT-like DNA-binding domain containing 1

0.84	UBASH3A	ubiquitin associated and SH3 domain containing A
0.84	SSR1	signal sequence receptor. alpha
0.84	CCDC84	coiled-coil domain containing 84
0.84	CNIH2	cornichon family AMPA receptor auxiliary protein 2
0.84	FNDC3B	fibronectin type III domain containing 3B
0.84	MIR99AHG	mir-99a-let-7c cluster host gene
0.84	HERC3	HECT and RLD domain containing E3 ubiquitin protein ligase 3
0.84	HLA-V	major histocompatibility complex. class I. V (pseudogene)
0.84	LOC100288966; POTED; BX072566.1	POTE ankyrin domain family member D-like; Synthetic construct Homo sapiens clone IMAGE:100066427. MGC:195564 POTE ankyrin domain family. member D (POTED) mRNA. encodes complete protein.; uncharacterized protein LOC100288966 [Source:RefSeq peptide;Acc:NP_001244291]
0.84	CD300C	CD300c molecule
0.84	SLC1A3	solute carrier family 1 (glial high affinity glutamate transporter). member 3
0.84	HOXD4	homeobox D4
0.84	MFSD5	major facilitator superfamily domain containing 5
0.84	C5orf15	chromosome 5 open reading frame 15
0.84	PITPNM3	PITPNM family member 3
0.84	HBG2; HBG1	hemoglobin. gamma G; hemoglobin. gamma A
0.84	SPATA6	spermatogenesis associated 6
0.84	LOC101927550; AC004893.11; SMURF1	uncharacterized LOC101927550; Salzman2013 ANTISENSE. CDS. coding. INTERNAL. intronic. OVCODE. OVERLAPTX. OVEXON best transcript NM_020429; novel transcript
0.84	KCNH3	potassium channel. voltage gated eag related subfamily H. member 3
0.84	EME1	essential meiotic structure-specific endonuclease 1
0.84	RELL2	RELT-like 2
0.84	GGA1	golgi-associated. gamma adaptin ear containing. ARF binding protein 1
0.84	GTF3C5	general transcription factor IIIC subunit 5

0.84	SDS	serine dehydratase
0.84	TOP3A	topoisomerase (DNA) III alpha
0.84	HLA-A	major histocompatibility complex. class I. A
0.84	SLC25A30	solute carrier family 25. member 30
0.84	SH2D1A	SH2 domain containing 1A
0.84	MPDU1	mannose-P-dolichol utilization defect 1
0.84	BCL6B	B-cell CLL/lymphoma 6. member B
0.84	PCDHB3	protocadherin beta 3
0.84	SMIM3	small integral membrane protein 3
0.84	BRD1	bromodomain containing 1
0.84	HIPK1	Memczak2013 ALT_DONOR. coding. INTERNAL. intronic best transcript NM_198268
0.84	C5orf45	chromosome 5 open reading frame 45
0.84	PNCK	pregnancy up-regulated nonubiquitous CaM kinase
0.84	LSM11	LSM11. U7 small nuclear RNA associated
0.84	TFIP11	tuftelin interacting protein 11
0.84	PRDX6	peroxiredoxin 6
0.84	GAL3ST1	galactose-3-O-sulfotransferase 1
0.84	C19orf60	chromosome 19 open reading frame 60
0.84	NME4	NME/NM23 nucleoside diphosphate kinase 4
0.84	BACH2	Transcript Identified by AceView. Entrez Gene ID(s) 60468
0.84	DNAJC30	DnaJ (Hsp40) homolog. subfamily C. member 30
0.84	SLC16A9	solute carrier family 16. member 9
0.84	FANCL	Fanconi anemia complementation group L
0.84	NLRP4	NLR family. pyrin domain containing 4
0.84	HIST1H4J	histone cluster 1. H4j

0.84	ETV2	ets variant 2
0.84	FREM1	FRAS1 related extracellular matrix 1
0.84	LINC00282	long intergenic non-protein coding RNA 282
0.84	TYK2	tyrosine kinase 2
0.84	TJAP1	Zhang2013 ALT_ACCEPTOR. ALT_DONOR. coding. INTERNAL. intronic best transcript NM_001146018
0.84	TTLL2	tubulin tyrosine ligase-like family member 2
0.84	MRPS15	mitochondrial ribosomal protein S15
0.84	SLITRK4	SLIT and NTRK-like family. member 4
0.84	HNRNPL	heterogeneous nuclear ribonucleoprotein L
0.84	TUBA1B	tubulin. alpha 1b
0.84	SREBF2	sterol regulatory element binding transcription factor 2
0.84	CHRND	cholinergic receptor. nicotinic delta
0.84	CELA1	chymotrypsin-like elastase family. member 1
0.84	ZNF844	zinc finger protein 844
0.84	FNDC8	fibronectin type III domain containing 8
0.84	LINC01567	long intergenic non-protein coding RNA 1567
0.84	CNTROB	Transcript Identified by AceView. Entrez Gene ID(s) 116840
0.84	DDX56	DEAD (Asp-Glu-Ala-Asp) box helicase 56
0.84	MS4A15	membrane-spanning 4-domains. subfamily A. member 15
0.84	TSLP	thymic stromal lymphopoietin
0.84	SQLE	squalene epoxidase
0.84	CIR1	corepressor interacting with RBPJ. 1
0.84	GPR19	G protein-coupled receptor 19
0.84	PKP4	Transcript Identified by AceView. Entrez Gene ID(s) 8502
0.84	PRPF6	pre-mRNA processing factor 6

0.84	APOE	apolipoprotein E
0.84	HSPB3	heat shock 27kDa protein 3
0.84	CADPS2	Ca++-dependent secretion activator 2
0.84	GBF1	golgi brefeldin A resistant guanine nucleotide exchange factor 1
0.84	PLEK2	pleckstrin 2
0.84	ZNF681	zinc finger protein 681
0.84	IL2RA	interleukin 2 receptor. alpha
0.84	CALR	calreticulin
0.84	NPFFR2	neuropeptide FF receptor 2
0.84	DDX60	DEAD (Asp-Glu-Ala-Asp) box polypeptide 60
0.84	NFIA	nuclear factor I/A
0.84	GLIPR1L1	GLI pathogenesis-related 1 like 1
0.84	RSPH10B2; RSPH10B	radial spoke head 10 homolog B2 (Chlamydomonas); radial spoke head 10 homolog B (Chlamydomonas)
0.84	USP50	ubiquitin specific peptidase 50
0.84	ZNF652	zinc finger protein 652
0.84	MYL2	myosin light chain 2
0.84	CDK4	cyclin-dependent kinase 4
0.84	EMILIN2	elastin microfibril interfacer 2
0.84	EIF2AK3	eukaryotic translation initiation factor 2-alpha kinase 3
0.84	CD27	CD27 molecule
0.84	CPSF6	cleavage and polyadenylation specific factor 6
0.84	ATP10D	ATPase. class V. type 10D
0.84	MUC3A	mucin 3A. cell surface associated
0.84	GAB4	GRB2-associated binding protein family. member 4
0.84	ZNF570	zinc finger protein 570

0.84	HOXC13	homeobox C13
0.84	ATF6B	activating transcription factor 6 beta
0.84	RHOH	ras homolog family member H
0.84	MPHOSPH9	M-phase phosphoprotein 9
0.84	MAP4K1	mitogen-activated protein kinase kinase kinase kinase 1
0.84	NDUFA8	Transcript Identified by AceView. Entrez Gene ID(s) 4702
0.84	TMEM63A	transmembrane protein 63A
0.84	ZNF618	zinc finger protein 618
0.84	CARNS1	carnosine synthase 1
0.84	MYO7B	myosin VIIB
0.84	CD46	Transcript Identified by AceView. Entrez Gene ID(s) 4179
0.84	MRPL38	mitochondrial ribosomal protein L38
0.84	LRP3	LDL receptor related protein 3
0.84	CD84	CD84 molecule
0.84	CCDC84	coiled-coil domain containing 84
0.84	DGCR6	DiGeorge syndrome critical region gene 6
0.84	CCL3	chemokine (C-C motif) ligand 3
0.84	ZG16	zymogen granule protein 16
0.84	TPPP2	tubulin polymerization-promoting protein family member 2
0.84	TMEM145	transmembrane protein 145
0.84	SULT1A1	sulfotransferase family 1A member 1
0.84	CYP2D6	cytochrome P450. family 2. subfamily D. polypeptide 6
0.84	FBRS	fibrosin
0.84	ARMT1	acidic residue methyltransferase 1
0.84	ANKS6	ankyrin repeat and sterile alpha motif domain containing 6
0.84	NFYC	nuclear transcription factor Y subunit gamma

0.84	FAM120AOS	family with sequence similarity 120A opposite strand
0.84	ABHD18	abhydrolase domain containing 18
0.84	ATG10	autophagy related 10
0.84	PNPLA4	patatin-like phospholipase domain containing 4
0.84	HIST1H4K	histone cluster 1. H4k
0.84	SRPK1	SRSF protein kinase 1
0.84	C4orf27	chromosome 4 open reading frame 27
0.84	LIAS	lipoic acid synthetase
0.84	TSPAN31	tetraspanin 31
0.84	LMAN1L	lectin. mannose-binding. 1 like
0.84	PROX1	prospero homeobox 1
0.84	TRIM46	tripartite motif containing 46
0.84	DUOX2	dual oxidase 2
0.84	SERPINH1	Jeck2013 ANTISENSE. coding. INTERNAL. intronic best transcript NM_001207014
0.84	GUCY2F	guanylate cyclase 2F. retinal
0.84	HBA2	hemoglobin. alpha 2
0.84	MLLT1	myeloid/lymphoid or mixed-lineage leukemia; translocated to. 1
0.84	PCBP3	poly(rC) binding protein 3
0.84	NDNF	neuron-derived neurotrophic factor
0.84	CNGA2	cyclic nucleotide gated channel alpha 2
0.84	ZC2HC1C	zinc finger. C2HC-type containing 1C
0.84	FGF16	fibroblast growth factor 16
0.84	STAG3	stromal antigen 3
0.84	NPR2	natriuretic peptide receptor 2
0.84	ECH1	enoyl-CoA hydratase 1. peroxisomal

0.84	ZAN	zonadhesin (gene/pseudogene)
0.84	NYAP1	neuronal tyrosine-phosphorylated phosphoinositide-3-kinase adaptor 1
0.84	NPNT	nephronectin
0.84	ZNF311	zinc finger protein 311
0.84	ZBTB8OS	zinc finger and BTB domain containing 8 opposite strand
0.84	MANEA	mannosidase. endo-alpha
0.84	PLK5	polo-like kinase 5
0.84	CRKL	Memczak2013 ANTISENSE. CDS. coding. INTERNAL best transcript NM_005207
0.84	PAN2; CNPY2	PAN2 poly(A) specific ribonuclease subunit; canopy FGF signaling regulator 2
0.84	ZFP41	ZFP41 zinc finger protein
0.84	CSAG3	CSAG family. member 3
0.84	PDGFD	platelet derived growth factor D
0.84	PNISR	PNN-interacting serine/arginine-rich protein
0.84	LAMP5	lysosomal-associated membrane protein family. member 5
0.84	CCDC115	coiled-coil domain containing 115
0.84	TTC24	tetratricopeptide repeat domain 24
0.84	HOXA-AS3	HOXA cluster antisense RNA 3
0.84	BAG3	BCL2-associated athanogene 3
0.84	BARHL1	BarH-like homeobox 1
0.84	NUTM2F	NUT family member 2F
0.84	CYBRD1	cytochrome b reductase 1
0.84	ELFN1	extracellular leucine-rich repeat and fibronectin type III domain containing 1
0.84	CRIP2	cysteine-rich protein 2
0.84	ASB16-AS1	ASB16 antisense RNA 1
0.84	TP73-AS1	TP73 antisense RNA 1

0.84	FAM20B	family with sequence similarity 20. member B
0.84	NEK8	NIMA-related kinase 8
0.84	DUX3	double homeobox 3
0.84	NADSYN1	NAD synthetase 1
0.84	TPRX1	tetra-peptide repeat homeobox 1
0.84	ARF5	ADP-ribosylation factor 5
0.84	PHF6	PHD finger protein 6
0.84	SPG7	spastic paraplegia 7 (pure and complicated autosomal recessive)
0.84	COMMD3-BMI1; BMI1; COMMD3	COMMD3-BMI1 readthrough; BMI1 proto-oncogene. polycomb ring finger; COMM domain containing 3
0.84	KDELR2	KDEL (Lys-Asp-Glu-Leu) endoplasmic reticulum protein retention receptor 2
0.84	SFXN5	sideroflexin 5
0.84	CCDC71L	coiled-coil domain containing 71-like
0.84	DMGDH	dimethylglycine dehydrogenase
0.84	STRA6	stimulated by retinoic acid 6
0.84	WISP1	WNT1 inducible signaling pathway protein 1
0.84	TMEM121	Memczak2013 ANTISENSE. CDS. coding. INTERNAL best transcript NM_025268
0.84	BCL2L12	BCL2-like 12 (proline rich)
0.84	TCEB3CL; TCEB3C	transcription elongation factor B polypeptide 3C-like; transcription elongation factor B polypeptide 3C (elongin A3)
0.84	USP51	ubiquitin specific peptidase 51
0.84	AGAP5	ArfGAP with GTPase domain. ankyrin repeat and PH domain 5
0.84	ACBD3	acyl-CoA binding domain containing 3
0.84	C9orf116	chromosome 9 open reading frame 116
0.84	PDE6D	phosphodiesterase 6D. cGMP-specific. rod. delta
0.84	ATP5H	ATP synthase. H+ transporting. mitochondrial Fo complex subunit D

0.84	LZTS2	leucine zipper. putative tumor suppressor 2
0.84	KIAA1644	KIAA1644
0.84	SERP2	stress-associated endoplasmic reticulum protein family member 2
0.84	RPP40	ribonuclease P/MRP 40kDa subunit
0.84	CACNB1	calcium channel. voltage-dependent. beta 1 subunit
0.84	CEBPD	CCAAT/enhancer binding protein (C/EBP). delta
0.84	BTN2A3P	butyrophilin. subfamily 2. member A3. pseudogene
0.84	NDEL1	nudE neurodevelopment protein 1-like 1
0.84	OR2A42; OR2A1	olfactory receptor. family 2. subfamily A. member 42; olfactory receptor. family 2. subfamily A. member 1
0.84	COX18	COX18 cytochrome c oxidase assembly factor
0.84	POP7	POP7 homolog. ribonuclease P/MRP subunit
0.84	TCEB3CL2; TCEB3CL	transcription elongation factor B polypeptide 3C-like 2; transcription elongation factor B polypeptide 3C-like
0.84	DNPEP	aspartyl aminopeptidase
0.84	BTBD9	BTB (POZ) domain containing 9
0.84	LBP	lipopolysaccharide binding protein
0.84	LONRF1	Transcript Identified by AceView. Entrez Gene ID(s) 91694
0.84	OPA3	optic atrophy 3 (autosomal recessive. with chorea and spastic paraplegia)
0.84	LOC100129924; C1orf50; RP5-994D16.11	uncharacterized LOC100129924; Transcript Identified by AceView. Entrez Gene ID(s) 79078; novel transcript
0.84	FDFT1	farnesyl-diphosphate farnesyltransferase 1
0.84	HSPA1A; HSPA1B	heat shock 70kDa protein 1A; heat shock 70kDa protein 1B
0.84	C10orf62	chromosome 10 open reading frame 62
0.84	BTG1; LINC01619	B-cell translocation gene 1. anti-proliferative; long intergenic non-protein coding RNA 1619
0.84	CLEC7A	C-type lectin domain family 7. member A

0.84	HGF	Transcript Identified by AceView. Entrez Gene ID(s) 3082
0.84	GJA5	gap junction protein alpha 5
0.84	PCED1A	PC-esterase domain containing 1A
0.84	C15orf56	chromosome 15 open reading frame 56
0.84	EXT1; spawla	Jeck2013 ALT_ACCEPTOR. ALT_DONOR. coding. INTERNAL. intronic best transcript NM_000127; Transcript Identified by AceView
0.84	RMDN1	regulator of microtubule dynamics 1
0.84	TMEM106C	transmembrane protein 106C
0.84	ZNF546	zinc finger protein 546
0.84	SLC39A4	solute carrier family 39 (zinc transporter). member 4
0.84	PRAMEF5	PRAME family member 5
0.84	NOD2	nucleotide-binding oligomerization domain containing 2
0.84	FST	follistatin
0.84	PTPRD	protein tyrosine phosphatase. receptor type. D
0.84	ZNF785	zinc finger protein 785
0.84	CA5A	carbonic anhydrase VA. mitochondrial
0.84	NDUFB7	NADH dehydrogenase (ubiquinone) 1 beta subcomplex. 7. 18kDa
0.84	GALK2	galactokinase 2
0.84	PANK2	pantothenate kinase 2
0.84	ANKRD53	ankyrin repeat domain 53
0.84	ZNF81	zinc finger protein 81
0.84	WNK2	WNK lysine deficient protein kinase 2
0.84	ASPM	abnormal spindle microtubule assembly
0.84	SF3B5	splicing factor 3b subunit 5
0.84	HOOK3	hook microtubule-tethering protein 3
0.84	RAD54L	Transcript Identified by AceView. Entrez Gene ID(s) 8438

0.84	SUPT5H	SPT5 homolog. DSIF elongation factor subunit
0.84	GSC	goosecoid homeobox
0.84	STK32C	serine/threonine kinase 32C
0.84	VPS28	vacuolar protein sorting 28 homolog (S. cerevisiae)
0.84	OPALIN	oligodendrocytic myelin paranodal and inner loop protein
0.84	RANBP1	RAN binding protein 1
0.84	TSPAN12	tetraspanin 12
0.84	SNX29P2; SNX29P1	sorting nexin 29 pseudogene 2; sorting nexin 29 pseudogene 1
0.84	PI15	peptidase inhibitor 15
0.84	PROC	protein C (inactivator of coagulation factors Va and VIIIa)
0.83	KIAA1919	KIAA1919
0.83	BPIFB1	BPI fold containing family B. member 1
0.83	MRPL4	mitochondrial ribosomal protein L4
0.83	ARF4	ADP-ribosylation factor 4
0.83	ARPIN	actin-related protein 2/3 complex inhibitor
0.83	SELPLG	selectin P ligand
0.83	CCZ1B	CCZ1 homolog B. vacuolar protein trafficking and biogenesis associated
0.83	SEMA6A	sema domain. transmembrane domain (TM). and cytoplasmic domain. (semaphorin) 6A
0.83	MEIS2	Meis homeobox 2
0.83	C19orf81	chromosome 19 open reading frame 81
0.83	MSLN	mesothelin
0.83	KMT2D	lysine (K)-specific methyltransferase 2D
0.83	FBXO39	F-box protein 39
0.83	LGR4	leucine-rich repeat containing G protein-coupled receptor 4
0.83	GNLY	granulysin

0.83	SLC2A9	solute carrier family 2 (facilitated glucose transporter). member 9
0.83	PTCD2	pentatricopeptide repeat domain 2
0.83	NBPF3	neuroblastoma breakpoint family. member 3
0.83	ZNF430	zinc finger protein 430
0.83	NDUFB6	NADH dehydrogenase (ubiquinone) 1 beta subcomplex. 6. 17kDa
0.83	LTBP4	latent transforming growth factor beta binding protein 4
0.83	GUCA2A	guanylate cyclase activator 2A (guanylin)
0.83	PPP1R32	protein phosphatase 1. regulatory subunit 32
0.83	INTS3	integrator complex subunit 3
0.83	ACBD4	acyl-CoA binding domain containing 4
0.83	ZNF280D	zinc finger protein 280D
0.83	ITIH6	inter-alpha-trypsin inhibitor heavy chain family. member 6
0.83	OR5B12	olfactory receptor. family 5. subfamily B. member 12
0.83	HIGD1C	HIG1 hypoxia inducible domain family. member 1C
0.83	NUPR1	nuclear protein 1. transcriptional regulator
0.83	EHF	ets homologous factor
0.83	PLPPR2	phospholipid phosphatase related 2
0.83	TCP10L2	t-complex 10-like 2
0.83	KLHDC8A	kelch domain containing 8A
0.83	SEPP1	selenoprotein P. plasma. 1
0.83	CLDN3	claudin 3
0.83	RPRD2	regulation of nuclear pre-mRNA domain containing 2
0.83	ARRDC3	arrestin domain containing 3
0.83	HYI	hydroxypyruvate isomerase (putative)
0.83	TNFRSF10C	tumor necrosis factor receptor superfamily. member 10c. decoy without an intracellular domain

0.83	CATSPER3	cation channel. sperm associated 3
0.83	KLF1	Kruppel-like factor 1 (erythroid)
0.83	OIP5	Opa interacting protein 5
0.83	MYB	v-myb avian myeloblastosis viral oncogene homolog
0.83	LOC100131315; RP11-758N13.1	uncharacterized LOC100131315; novel transcript
0.83	C11orf85	chromosome 11 open reading frame 85
0.83	SNF8	SNF8. ESCRT-II complex subunit
0.83	FAM171B	family with sequence similarity 171. member B
0.83	IGFBP6	insulin like growth factor binding protein 6
0.83	NID1	nidogen 1
0.83	HABP2	hyaluronan binding protein 2
0.83	ASH2L	ash2 (absent. small. or homeotic)-like (Drosophila)
0.83	PCK2	phosphoenolpyruvate carboxykinase 2 (mitochondrial)
0.83	SURF4	surfeit 4
0.83	ZRSR2	zinc finger (CCCH type). RNA binding motif and serine/arginine rich 2
0.83	CDH13	cadherin 13
0.83	ADAM8	ADAM metallopeptidase domain 8
0.83	AACSP1	acetoacetyl-CoA synthetase pseudogene 1
0.83	CCNB2	cyclin B2
0.83	OGFOD3	2-oxoglutarate and iron-dependent oxygenase domain containing 3
0.83	TCHP	trichoplein. keratin filament binding
0.83	FRG2C	FSHD region gene 2 family. member C
0.83	MORN2	MORN repeat containing 2
0.83	CSRP2	cysteine and glycine-rich protein 2
0.83	COQ7	coenzyme Q7 homolog. ubiquinone (yeast)
0.83	GTF2H2; GTF2H2B	general transcription factor IIH subunit 2; general transcription factor IIH

		subunit 2B (pseudogene)
0.83	ADRB3	adrenoceptor beta 3
0.83	USE1	unconventional SNARE in the ER 1 homolog (S. cerevisiae)
0.83	GRPR	gastrin-releasing peptide receptor
0.83	MTRNR2L2	MT-RNR2-like 2
0.83	SPATS1	spermatogenesis associated. serine-rich 1
0.83	ANKRD18A; FAM95C	ankyrin repeat domain 18A; family with sequence similarity 95. member C
0.83	DUSP19	dual specificity phosphatase 19
0.83	ZCCHC14	zinc finger. CCHC domain containing 14
0.83	SCGN	secretagogin. EF-hand calcium binding protein
0.83	TMEM132B	transmembrane protein 132B
0.83	LOC100134391; CTD-2532D12.4	uncharacterized LOC100134391; novel transcript
0.83	SLC25A14	solute carrier family 25 (mitochondrial carrier. brain). member 14
0.83	FN3KRP	fructosamine 3 kinase related protein
0.83	SULT2A1	sulfotransferase family 2A member 1
0.83	USP49	ubiquitin specific peptidase 49
0.83	VWA7	von Willebrand factor A domain containing 7
0.83	XPNPEP3	X-prolyl aminopeptidase 3. mitochondrial
0.83	BAHD1	bromo adjacent homology domain containing 1
0.83	ZNF268	zinc finger protein 268
0.83	TMEM183A	transmembrane protein 183A
0.83	LHB	luteinizing hormone beta polypeptide
0.83	TUBGCP6	tubulin. gamma complex associated protein 6
0.83	CHMP1A	charged multivesicular body protein 1A
0.83	TJP1	Transcript Identified by AceView. Entrez Gene ID(s) 7082

0.83	IGLL5; MIR5571; IGLC1; IGLC2; IGLV2-5; IGLV3-1; IGLJ1	immunoglobulin lambda-like polypeptide 5; microRNA 5571; immunoglobulin lambda constant 1 (Mcg marker); immunoglobulin lambda constant 2 (Kern-Oz- marker); immunoglobulin lambda variable 2-5 (pseudogene); immunoglobulin lambda variable 3-1; immunoglobulin lambda joining 1
0.83	PROM2	prominin 2
0.83	GMPR	guanosine monophosphate reductase
0.83	ENTPD5	ectonucleoside triphosphate diphosphohydrolase 5
0.83	ABHD4	abhydrolase domain containing 4
0.83	ZSWIM4	zinc finger. SWIM-type containing 4
0.83	TMPRSS2	transmembrane protease. serine 2
0.83	EFCAB8	EF-hand calcium binding domain 8
0.83	LGALS17A	Charcot-Leyden crystal protein pseudogene
0.83	ZNF696	zinc finger protein 696
0.83	SLC25A33	solute carrier family 25 (pyrimidine nucleotide carrier). member 33
0.83	RUSC1	RUN and SH3 domain containing 1
0.83	DNM3	dynamin 3
0.83	GATB	glutamyl-tRNA(Gln) amidotransferase. subunit B
0.83	CPNE6	copine VI (neuronal)
0.83	LINC00336	long intergenic non-protein coding RNA 336
0.83	BEAN1-AS1	BEAN1 antisense RNA 1
0.83	MXD3	MAX dimerization protein 3
0.83	TMEM55B	transmembrane protein 55B
0.83	LYST	lysosomal trafficking regulator
0.83	RQCD1	RCD1 required for cell differentiation1 homolog (S. pombe)
0.83	SCTR	secretin receptor
0.83	RECQL	RecQ helicase-like

0.83	ERCC5	excision repair cross-complementation group 5
0.83	GJC3	gap junction protein gamma 3
0.83	ZNF552	zinc finger protein 552
0.83	PSME1	proteasome activator subunit 1
0.83	WASF3	WAS protein family. member 3
0.83	LSM4	LSM4 homolog. U6 small nuclear RNA and mRNA degradation associated
0.83	TXNDC16	thioredoxin domain containing 16
0.83	CAMKMT	Memczak2013 ALT_ACCEPTOR. ALT_DONOR. coding. INTERNAL. intronic best transcript NM_024766
0.83	PIGN	phosphatidylinositol glycan anchor biosynthesis class N
0.83	SPAG1	sperm associated antigen 1
0.83	LMO7DN	LMO7 downstream neighbor
0.83	KCTD7	potassium channel tetramerization domain containing 7
0.83	MAST3	microtubule associated serine/threonine kinase 3
0.83	KRTAP1-1	keratin associated protein 1-1
0.83	PEX11G	peroxisomal biogenesis factor 11 gamma
0.83	PRRC1	proline-rich coiled-coil 1
0.83	LIG3	ligase III. DNA. ATP-dependent
0.83	MYL1	myosin light chain 1
0.83	KCNK2	potassium channel. two pore domain subfamily K. member 2
0.83	BOLA2; BOLA2B	bolA family member 2; bolA family member 2B
0.83	THAP2	THAP domain containing. apoptosis associated protein 2
0.83	RUNX1	runt-related transcription factor 1
0.83	ZNF747	zinc finger protein 747
0.83	PRAMEF9	PRAME family member 9
0.83	WDR24	WD repeat domain 24

0.83	GSC2	goosecoid homeobox 2
0.83	SSUH2	ssu-2 homolog (C. elegans)
0.83	ORMDL2	ORMDL sphingolipid biosynthesis regulator 2
0.83	DLL3	delta-like 3 (Drosophila)
0.83	TF	transferrin
0.83	HEXA	hexosaminidase A (alpha polypeptide)
0.83	REL	v-rel avian reticuloendotheliosis viral oncogene homolog
0.83	ARFGAP2	ADP-ribosylation factor GTPase activating protein 2
0.83	HMBS	hydroxymethylbilane synthase
0.83	DNAH10OS	dynein. axonemal. heavy chain 10 opposite strand
0.83	ZNF891	zinc finger protein 891
0.83	ANKRD23	ankyrin repeat domain 23
0.83	KPTN	kaptin (actin binding protein)
0.83	FHDC1	FH2 domain containing 1
0.83	PCDHB2	protocadherin beta 2
0.83	STAC2	SH3 and cysteine rich domain 2
0.83	GDPD5	glycerophosphodiester phosphodiesterase domain containing 5
0.83	GIPC3	GIPC PDZ domain containing family. member 3
0.83	DIRC3	disrupted in renal carcinoma 3
0.83	CCDC74A	coiled-coil domain containing 74A
0.83	GYG2	glycogenin 2
0.83	LRRN1	leucine rich repeat neuronal 1
0.83	PCDH11Y	protocadherin 11 Y-linked
0.83	SLC46A2	solute carrier family 46. member 2
0.83	PRRT1	proline-rich transmembrane protein 1
0.83	LINC01272	long intergenic non-protein coding RNA 1272

0.83	KCTD4	potassium channel tetramerization domain containing 4
0.83	RSAD2	radical S-adenosyl methionine domain containing 2
0.83	CPNE4	Transcript Identified by AceView. Entrez Gene ID(s) 131034
0.83	DHX8	DEAH (Asp-Glu-Ala-His) box polypeptide 8
0.83	GDPGP1	GDP-D-glucose phosphorylase 1
0.83	MAPK3	mitogen-activated protein kinase 3
0.83	TNFRSF11A	Transcript Identified by AceView. Entrez Gene ID(s) 8792
0.83	NUP62; IL4I1	nucleoporin 62kDa; interleukin 4 induced 1
0.83	HS3ST1	heparan sulfate (glucosamine) 3-O-sulfotransferase 1
0.83	WNK3	WNK lysine deficient protein kinase 3
0.83	XKR3	X-linked Kx blood group related 3
0.83	GVQW2	GVQW motif containing 2
0.83	MYLPF	myosin light chain. phosphorylatable. fast skeletal muscle
0.83	IFNGR1	interferon gamma receptor 1
0.83	OR4C3	olfactory receptor. family 4. subfamily C. member 3
0.83	AQP8	aquaporin 8
0.83	BOD1L1	biorientation of chromosomes in cell division 1-like 1
0.83	ACACB	acetyl-CoA carboxylase beta
0.83	OTX2	orthodenticle homeobox 2
0.83	UQCC3	ubiquinol-cytochrome c reductase complex assembly factor 3
0.83	USP24	Transcript Identified by AceView. Entrez Gene ID(s) 23358
0.83	GANAB	glucosidase. alpha; neutral AB
0.83	NPHS2	nephrosis 2. idiopathic. steroid-resistant (podocin)
0.83	IPP	intracisternal A particle-promoted polypeptide
0.83	TERF1	telomeric repeat binding factor (NIMA-interacting) 1
0.83	DPP4	dipeptidyl-peptidase 4

0.83	SRP54	signal recognition particle 54kDa
0.83	METTL3	methyltransferase like 3
0.83	SOSTDC1	sclerostin domain containing 1
0.83	TRMT10B	tRNA methyltransferase 10B
0.83	CGB; CGB5	chorionic gonadotropin. beta polypeptide; chorionic gonadotropin. beta polypeptide 5
0.83	SEZ6	Memczak2013 ANTISENSE. CDS. coding. INTERNAL. intronic best transcript NM_178860
0.83	NCOA5	nuclear receptor coactivator 5
0.83	GOLGB1	golgin B1
0.83	FAM174B	family with sequence similarity 174. member B
0.83	CCDC138	coiled-coil domain containing 138
0.83	SORD	sorbitol dehydrogenase
0.83	ZDHHC11	zinc finger. DHHC-type containing 11
0.83	ZNF546	zinc finger protein 546
0.83	SCARA5	scavenger receptor class A. member 5
0.83	EPT1	ethanolaminephosphotransferase 1
0.83	HMGCS1	3-hydroxy-3-methylglutaryl-CoA synthase 1 (soluble)
0.83	RNF167	ring finger protein 167
0.83	HECW1	HECT. C2 and WW domain containing E3 ubiquitin protein ligase 1
0.83	GLI2	GLI family zinc finger 2
0.83	SH2D6	SH2 domain containing 6
0.83	ARHGAP36	Rho GTPase activating protein 36
0.83	DAO	D-amino-acid oxidase
0.83	GRK7	G protein-coupled receptor kinase 7
0.83	DUSP21	dual specificity phosphatase 21
0.83	GPHB5	glycoprotein hormone beta 5

0.83	NABP2	nucleic acid binding protein 2
0.83	WBSCR17	Williams-Beuren syndrome chromosome region 17
0.83	CTSC	cathepsin C
0.83	RAE1	ribonucleic acid export 1
0.83	EIF4E3	eukaryotic translation initiation factor 4E family member 3
0.83	SPAG11B	sperm associated antigen 11B
0.83	INHBC	inhibin beta C
0.83	N6AMT2	N-6 adenine-specific DNA methyltransferase 2 (putative)
0.83	ZNF16	zinc finger protein 16
0.83	ICMT	isoprenylcysteine carboxyl methyltransferase
0.83	PRKAG1	protein kinase. AMP-activated. gamma 1 non-catalytic subunit
0.83	GCM2	glial cells missing homolog 2 (Drosophila)
0.83	MAOB	monoamine oxidase B
0.83	ARHGAP33	Rho GTPase activating protein 33
0.83	MPZ	myelin protein zero
0.83	WDR6	WD repeat domain 6
0.83	LRRC37A3	leucine rich repeat containing 37. member A3
0.83	PHTF1	putative homeodomain transcription factor 1
0.83	ORC1	origin recognition complex subunit 1
0.83	ZNF691	zinc finger protein 691
0.83	SP1	Sp1 transcription factor
0.83	PRKAG3	protein kinase. AMP-activated. gamma 3 non-catalytic subunit
0.83	AQP4	aquaporin 4
0.83	SLC13A5	solute carrier family 13 (sodium-dependent citrate transporter). member 5
0.83	ZMAT5	zinc finger. matrin-type 5
0.83	MICAL3	microtubule associated monooxygenase. calponin and LIM domain

		containing 3
0.83	NELFE; MIR1236	negative elongation factor complex member E; microRNA 1236
0.83	ADAMTS13	ADAM metallopeptidase with thrombospondin type 1 motif 13
0.83	ZNF727	zinc finger protein 727
0.83	ZBTB37	zinc finger and BTB domain containing 37
0.83	KDM8	lysine (K)-specific demethylase 8
0.83	SPAG4	sperm associated antigen 4
0.83	RAPGEF3	Rap guanine nucleotide exchange factor 3
0.83	ANKS4B	ankyrin repeat and sterile alpha motif domain containing 4B
0.83	UROS	uroporphyrinogen III synthase
0.83	TTC3	tetratricopeptide repeat domain 3
0.83	TUBB3; MC1R	tubulin. beta 3 class III; melanocortin 1 receptor (alpha melanocyte stimulating hormone receptor)
0.83	MYH7	myosin. heavy chain 7. cardiac muscle. beta
0.83	KISS1R	KISS1 receptor
0.83	BAALC-AS2	BAALC antisense RNA 2
0.83	DCAF6	DDB1 and CUL4 associated factor 6
0.83	CENPE	centromere protein E
0.83	RTF1	RTF1 homolog. Paf1/RNA polymerase II complex component
0.83	NEURL1B	neuralized E3 ubiquitin protein ligase 1B
0.83	DNAJC28	DnaJ (Hsp40) homolog. subfamily C. member 28
0.83	TENM2	teneurin transmembrane protein 2
0.83	ALKBH4	alkB homolog 4. lysine demthylase
0.83	CLLU1OS	chronic lymphocytic leukemia up-regulated 1 opposite strand
0.83	RAB40B	RAB40B. member RAS oncogene family
0.83	NLRC5	NLR family. CARD domain containing 5

0.83	ARHGAP4	Rho GTPase activating protein 4
0.83	TMEM129	transmembrane protein 129. E3 ubiquitin protein ligase
0.83	TINF2	TERF1 (TRF1)-interacting nuclear factor 2
0.83	PRKG1	protein kinase. cGMP-dependent. type I
0.83	CRTC1	CREB regulated transcription coactivator 1
0.83	SIKE1	suppressor of IKBKE 1
0.83	ACOT8	acyl-CoA thioesterase 8
0.83	ARL2BP	ADP-ribosylation factor like GTPase 2 binding protein
0.83	KPNA6	Jeck2013 ALT_ACCEPTOR. ALT_DONOR. coding. INTERNAL. intronic best transcript NM_012316
0.83	HAO2	hydroxyacid oxidase 2 (long chain)
0.83	GSTM3	glutathione S-transferase mu 3 (brain)
0.83	RARB	retinoic acid receptor. beta
0.83	SFTA3	surfactant associated 3
0.83	VSIG10	V-set and immunoglobulin domain containing 10
0.83	ABCA13	Memczak2013 ALT_ACCEPTOR. ALT_DONOR. coding. INTERNAL. intronic best transcript NM_152701
0.83	FOXRED2	FAD-dependent oxidoreductase domain containing 2
0.83	MAP3K13	mitogen-activated protein kinase kinase kinase 13
0.83	C14orf37	chromosome 14 open reading frame 37
0.83	ZNF273	zinc finger protein 273
0.83	TAAR9	trace amine associated receptor 9 (gene/pseudogene)
0.83	PRUNE	prune exopolyphosphatase
0.83	AP4B1	adaptor-related protein complex 4. beta 1 subunit
0.83	THAP8	THAP domain containing 8
0.83	CCDC162P	coiled-coil domain containing 162. pseudogene
0.83	SIGIRR	single immunoglobulin and toll-interleukin 1 receptor (TIR) domain

0.83	LY6G6F	lymphocyte antigen 6 complex. locus G6F
0.83	GMEB2	glucocorticoid modulatory element binding protein 2
0.83	LINC00482	long intergenic non-protein coding RNA 482
0.83	DAB2	Dab. mitogen-responsive phosphoprotein. homolog 2 (Drosophila)
0.83	KCTD16	potassium channel tetramerization domain containing 16
0.83	PTCHD1	patched domain containing 1
0.83	AGAP4	ArfGAP with GTPase domain. ankyrin repeat and PH domain 4
0.83	FAM13B	family with sequence similarity 13. member B
0.83	CXorf38	chromosome X open reading frame 38
0.83	WNT11	wingless-type MMTV integration site family. member 11
0.83	HEATR9	HEAT repeat containing 9
0.83	DNMT1	DNA (cytosine-5-)-methyltransferase 1
0.83	CADM1	cell adhesion molecule 1
0.83	ABHD8	abhydrolase domain containing 8
0.83	PDZD2	PDZ domain containing 2
0.83	ACSS2	acyl-CoA synthetase short-chain family member 2
0.83	CHST4	carbohydrate (N-acetylglucosamine 6-O) sulfotransferase 4
0.83	CXorf51A; CXorf51B	chromosome X open reading frame 51A; chromosome X open reading frame 51B
0.83	PANK3	pantothenate kinase 3
0.83	MIR5195; IGHV5-78	microRNA 5195; immunoglobulin heavy variable 5-78 (pseudogene)
0.83	H3F3C	H3 histone. family 3C
0.83	NAA60	N(alpha)-acetyltransferase 60. NatF catalytic subunit
0.83	TCEB3CL	transcription elongation factor B polypeptide 3C-like
0.83	ANTXR1	anthrax toxin receptor 1
0.83	MCUR1	mitochondrial calcium uniporter regulator 1

0.83	PIP5KL1	phosphatidylinositol-4-phosphate 5-kinase-like 1
0.83	AKAP1	A kinase (PRKA) anchor protein 1
0.83	SERPINB13	serpin peptidase inhibitor. clade B (ovalbumin). member 13
0.83	SPIRE2	spire-type actin nucleation factor 2
0.83	TDP2	tyrosyl-DNA phosphodiesterase 2
0.83	SETD6	SET domain containing 6
0.83	IFNG	interferon. gamma
0.83	MPO	Memczak2013 ANTISENSE. CDS. coding. INTERNAL best transcript NM_000250
0.83	PNMA6A	paraneoplastic Ma antigen family member 6A
0.83	KLF3	Kruppel-like factor 3 (basic)
0.83	NR1H2	nuclear receptor subfamily 1. group H. member 2
0.83	DDHD2	DDHD domain containing 2
0.83	NFS1	NFS1 cysteine desulfurase
0.83	ZNF222	zinc finger protein 222
0.83	STK38L	serine/threonine kinase 38 like
0.83	IDE	insulin-degrading enzyme
0.83	TUSC2	tumor suppressor candidate 2
0.83	MSTO1; MSTO2P	misato 1. mitochondrial distribution and morphology regulator; misato family member 2. pseudogene
0.83	CHRAC1	Transcript Identified by AceView. Entrez Gene ID(s) 54108
0.83	PNCK	pregnancy up-regulated nonubiquitous CaM kinase
0.83	SUMO3	small ubiquitin-like modifier 3
0.83	NFKB2	nuclear factor of kappa light polypeptide gene enhancer in B-cells 2 (p49/p100)
0.83	DCPS	decapping enzyme. scavenger
0.83	TBX4	T-box 4

0.83	AHSA2	AHA1. activator of heat shock 90kDa protein ATPase homolog 2 (yeast)
0.83	GRM2	glutamate receptor. metabotropic 2
0.83	SLC22A17	solute carrier family 22. member 17
0.83	IL17C	interleukin 17C
0.83	LRRC37A11P	leucine rich repeat containing 37. member A11. pseudogene
0.83	GGH	gamma-glutamyl hydrolase (conjugase. folylpolygammaglutamyl hydrolase)
0.83	BPIFB3	BPI fold containing family B. member 3
0.83	POLD4	polymerase (DNA-directed). delta 4. accessory subunit
0.83	MTRNR2L10	MT-RNR2-like 10
0.83	NKX2-8	NK2 homeobox 8
0.83	FAM35A	family with sequence similarity 35. member A
0.83	PYGO2	pygopus family PHD finger 2
0.83	ITGBL1	integrin beta like 1
0.83	TMSB15A	thymosin beta 15a
0.83	RBL2	retinoblastoma-like 2
0.82	LAMC3	laminin. gamma 3
0.82	ALDOA	aldolase A. fructose-bisphosphate
0.82	GLA	galactosidase. alpha
0.82	RARA	retinoic acid receptor. alpha
0.82	GLIPR1L2	GLI pathogenesis-related 1 like 2
0.82	CTBS	chitobiase. di-N-acetyl-
0.82	LAT	linker for activation of T-cells
0.82	GPX4	glutathione peroxidase 4
0.82	GSTA5	glutathione S-transferase alpha 5
0.82	SYCP2L	synaptonemal complex protein 2-like
0.82	MAVS	mitochondrial antiviral signaling protein

0.82	GGCX	gamma-glutamyl carboxylase
0.82	WRAP53	WD repeat containing. antisense to TP53
0.82	KRTAP10-10	keratin associated protein 10-10
0.82	C3orf20	chromosome 3 open reading frame 20
0.82	SPATA33	spermatogenesis associated 33
0.82	JAZF1	JAZF zinc finger 1
0.82	SLC30A3	solute carrier family 30 (zinc transporter). member 3
0.82	PIK3IP1	phosphoinositide-3-kinase interacting protein 1
0.82	NDUFS8; MIR4691; MIR7113	NADH dehydrogenase (ubiquinone) Fe-S protein 8. 23kDa (NADH-coenzyme Q reductase); microRNA 4691; microRNA 7113
0.82	GAL3ST3	galactose-3-O-sulfotransferase 3
0.82	SGMS2	sphingomyelin synthase 2
0.82	SLC25A20	solute carrier family 25 (carnitine/acylcarnitine translocase). member 20
0.82	TUBD1	tubulin. delta 1
0.82	ZNF331	zinc finger protein 331
0.82	NEUROG2	neurogenin 2
0.82	CYP26A1	cytochrome P450. family 26. subfamily A. polypeptide 1
0.82	PSMC4	proteasome 26S subunit. ATPase 4 [Source:HGNC Symbol;Acc:HGNC:9551]
0.82	C2CD4D	C2 calcium-dependent domain containing 4D
0.82	CCDC120	coiled-coil domain containing 120
0.82	CDKN2C	cyclin-dependent kinase inhibitor 2C (p18. inhibits CDK4)
0.82	DEFB109P1B	defensin. beta 109. pseudogene 1B
0.82	NEUROD4	neuronal differentiation 4
0.82	LAMA5; MIR4758	laminin. alpha 5; microRNA 4758
0.82	CLTCL1	clathrin. heavy chain-like 1
0.82	IDUA	iduronidase. alpha-L-

0.82	PBXIP1	pre-B-cell leukemia homeobox interacting protein 1
0.82	RAVER1	ribonucleoprotein. PTB-binding 1
0.82	PNPO	pyridoxamine 5-phosphate oxidase
0.82	NMT2	N-myristoyltransferase 2
0.82	ARRDC5	arrestin domain containing 5
0.82	EPOR	erythropoietin receptor
0.82	APOF	apolipoprotein F
0.82	PHYHD1	phytanoyl-CoA dioxygenase domain containing 1
0.82	SYT4	synaptotagmin IV
0.82	DSC1	desmocollin 1
0.82	DSCR4	Down syndrome critical region 4
0.82	CPN1	carboxypeptidase N. polypeptide 1
0.82	LRRC27	leucine rich repeat containing 27
0.82	GJB5	gap junction protein beta 5
0.82	RTN4IP1	reticulon 4 interacting protein 1
0.82	CCND2	cyclin D2
0.82	MUT	methylmalonyl-CoA mutase
0.82	KSR1	kinase suppressor of ras 1
0.82	TCF25	transcription factor 25 (basic helix-loop-helix)
0.82	CRYBB2	crystallin beta B2
0.82	SLC25A25	solute carrier family 25 (mitochondrial carrier; phosphate carrier). member 25
0.82	PRKACA	protein kinase. cAMP-dependent. catalytic. alpha
0.82	ZNF492	zinc finger protein 492
0.82	STYK1	serine/threonine/tyrosine kinase 1
0.82	EGLN2	egl-9 family hypoxia-inducible factor 2

0.82	LAMB4	laminin. beta 4
0.82	CABIN1	calcineurin binding protein 1
0.82	TFDP1	transcription factor Dp-1
0.82	FSCN2	fascin actin-bundling protein 2. retinal
0.82	PRAMEF20	PRAME family member 20
0.82	SUV39H2	suppressor of variegation 3-9 homolog 2 (Drosophila)
0.82	TTC22	tetratricopeptide repeat domain 22
0.82	EXOC7; MIR6868	exocyst complex component 7; microRNA 6868
0.82	NUP37	nucleoporin 37kDa
0.82	PDGFRB	platelet-derived growth factor receptor. beta polypeptide
0.82	FAHD2A	fumarylacetoacetate hydrolase domain containing 2A
0.82	POTEB	POTE ankyrin domain family. member B
0.82	CXCL2	chemokine (C-X-C motif) ligand 2
0.82	RAB28	RAB28. member RAS oncogene family
0.82	GHSR	growth hormone secretagogue receptor
0.82	EPHX3	epoxide hydrolase 3
0.82	BPNT1	3(2). 5-bisphosphate nucleotidase 1
0.82	TAB3	TGF-beta activated kinase 1/MAP3K7 binding protein 3
0.82	SZT2	seizure threshold 2 homolog (mouse)
0.82	TNFRSF13C; MIR378I	tumor necrosis factor receptor superfamily. member 13C; microRNA 378i
0.82	PLEKHG4B	pleckstrin homology domain containing. family G (with RhoGef domain) member 4B
0.82	FAM21A	family with sequence similarity 21. member A
0.82	KRAS	Memczak2013 ANTISENSE. coding. INTERNAL. intronic best transcript NM_033360
0.82	FAM21C	family with sequence similarity 21. member C
0.82	METTL7A	methyltransferase like 7A

0.82	DNTTIP1	deoxynucleotidyltransferase. terminal. interacting protein 1
0.82	ZNF101	zinc finger protein 101
0.82	YWHAG	Memczak2013 ALT_ACCEPTOR. ALT_DONOR. coding. INTERNAL. intronic best transcript NM_012479
0.82	IKZF4	IKAROS family zinc finger 4
0.82	TMEM50B	transmembrane protein 50B
0.82	SLCO1A2	Transcript Identified by AceView. Entrez Gene ID(s) 6579
0.82	SENP5	Transcript Identified by AceView. Entrez Gene ID(s) 205564
0.82	INAFM1	InaF-motif containing 1
0.82	PILRB; STAG3L5P; PVRIG2P; MIR6840; STAG3L5P-PVRIG2P-PILRB	paired immunoglobin-like type 2 receptor beta; stromal antigen 3-like 5 pseudogene; poliovirus receptor related immunoglobulin domain containing 2. pseudogene; microRNA 6840; STAG3L5P-PVRIG2P-PILRB readthrough
0.82	FLJ38576; RP11-173M11.6	uncharacterized LOC651430; uncharacterized LOC651430 [Source:EntrezGene;Acc:651430]; novel transcript
0.82	PCYOX1	prenylcysteine oxidase 1
0.82	ITGB6	integrin beta 6
0.82	RAB3C	RAB3C. member RAS oncogene family
0.82	WDR61	WD repeat domain 61
0.82	VEGFC	vascular endothelial growth factor C
0.82	HAGHL	hydroxyacylglutathione hydrolase-like
0.82	B9D1	B9 protein domain 1
0.82	NPRL3	NPR3-like. GATOR1 complex subunit
0.82	CCDC7	coiled-coil domain containing 7
0.82	MYL6	myosin light chain 6
0.82	RUNX3	runt-related transcription factor 3
0.82	C1orf106	chromosome 1 open reading frame 106
0.82	CRYBB1	crystallin beta B1

0.82	OR4F21	olfactory receptor. family 4. subfamily F. member 21
0.82	SURF4	surfeit 4
0.82	SGTA	small glutamine-rich tetratricopeptide repeat (TPR)-containing. alpha
0.82	PDHB	pyruvate dehydrogenase (lipoamide) beta
0.82	FAM234A; ARHGDIG	family with sequence similarity 234. member A; Rho GDP dissociation inhibitor (GDI) gamma
0.82	RFPL3	ret finger protein-like 3
0.82	ATP6V1G1	ATPase. H+ transporting. lysosomal 13kDa. V1 subunit G1
0.82	ARGFX	arginine-fifty homeobox
0.82	HOXB2	homeobox B2
0.82	ZNF662	zinc finger protein 662
0.82	RNF4	ring finger protein 4
0.82	SRSF10	serine/arginine-rich splicing factor 10
0.82	RNF5	ring finger protein 5. E3 ubiquitin protein ligase
0.82	GPR137C	G protein-coupled receptor 137C
0.82	DENND2A	DENN/MADD domain containing 2A
0.82	EBF2	early B-cell factor 2
0.82	DEDD2	death effector domain containing 2
0.82	ATP5I	ATP synthase. H+ transporting. mitochondrial Fo complex subunit E
0.82	C8orf87	chromosome 8 open reading frame 87
0.82	ASB6	ankyrin repeat and SOCS box containing 6
0.82	SLC35F1	solute carrier family 35. member F1
0.82	SRD5A3	steroid 5 alpha-reductase 3
0.82	BTN3A2	butyrophilin. subfamily 3. member A2
0.82	SEC61B	Sec61 translocon beta subunit
0.82	EEA1	early endosome antigen 1

0.82	ST5	suppression of tumorigenicity 5
0.82	TPPP	tubulin polymerization promoting protein
0.82	HMBS	hydroxymethylbilane synthase
0.82	ZNF626	zinc finger protein 626
0.82	ATP6AP1	ATPase. H+ transporting. lysosomal accessory protein 1
0.82	GALNT10	polypeptide N-acetylgalactosaminyltransferase 10
0.82	OSR2	odd-skipped related transciption factor 2
0.82	FAM65A	Memczak2013 ALT_ACCEPTOR. ALT_DONOR. coding. INTERNAL. intronic best transcript NM_001193522
0.82	NARFL	nuclear prelamin A recognition factor-like
0.82	CHRM4	cholinergic receptor. muscarinic 4
0.82	VRK1	vaccinia related kinase 1
0.82	LRRC63	leucine rich repeat containing 63
0.82	LRRC20	leucine rich repeat containing 20
0.82	POC5	POC5 centriolar protein
0.82	DDX53	DEAD (Asp-Glu-Ala-Asp) box polypeptide 53
0.82	LRRC16A	leucine rich repeat containing 16A
0.82	PRR34	proline rich 34
0.82	CACNA1D	calcium channel. voltage-dependent. L type. alpha 1D subunit
0.82	CENPH	centromere protein H
0.82	AMER1	APC membrane recruitment protein 1
0.82	PRR20D; PRR20B	proline rich 20D; proline rich 20B
0.82	PRR20D; PRR20B	proline rich 20D; proline rich 20B
0.82	PRR20C; PRR20E	proline rich 20C; proline rich 20E
0.82	PRR20B; PRR20E; PRR20A; PRR20D	proline rich 20B; proline rich 20E; proline rich 20A; proline rich 20D
0.82	PRR20C; PRR20D	proline rich 20C; proline rich 20D

0.82	AATK	apoptosis-associated tyrosine kinase
0.82	UBE2QL1	ubiquitin conjugating enzyme E2Q family-like 1
0.82	POU4F1	POU class 4 homeobox 1
0.82	ZNF385A	zinc finger protein 385A
0.82	HPN	hepsin
0.82	CEP72	centrosomal protein 72kDa
0.82	C2CD4B	C2 calcium-dependent domain containing 4B
0.82	STOX1	storkhead box 1
0.82	USP54	ubiquitin specific peptidase 54
0.82	WFIKKN1	WAP. follistatin/kazal. immunoglobulin. kunitz and netrin domain containing 1
0.82	DLEC1	deleted in lung and esophageal cancer 1
0.82	NRG2	neuregulin 2
0.82	E2F4	E2F transcription factor 4. p107/p130-binding
0.82	RFESD	Rieske (Fe-S) domain containing
0.82	SYNE3	spectrin repeat containing. nuclear envelope family member 3
0.82	RPP25L	ribonuclease P/MRP 25kDa subunit-like
0.82	OR8D1	olfactory receptor. family 8. subfamily D. member 1
0.82	NT5DC4	5-nucleotidase domain containing 4
0.82	HES7	hes family bHLH transcription factor 7
0.82	CASQ1	calsequestrin 1 (fast-twitch. skeletal muscle)
0.82	GIPC2	GIPC PDZ domain containing family. member 2
0.82	ZNF446	zinc finger protein 446
0.82	DPEP1	dipeptidase 1 (renal)
0.82	CENPF	centromere protein F
0.82	RNF31	ring finger protein 31

0.82	SORBS1	Transcript Identified by AceView. Entrez Gene ID(s) 10580
0.82	BST2	bone marrow stromal cell antigen 2
0.82	COPE	coatomer protein complex subunit epsilon
0.82	MNS1	meiosis-specific nuclear structural 1
0.82	PTGIR	prostaglandin I2 (prostacyclin) receptor (IP)
0.82	PAK7	p21 protein (Cdc42/Rac)-activated kinase 7
0.82	MDGA1	MAM domain containing glycosylphosphatidylinositol anchor 1
0.82	AP1S1	Transcript Identified by AceView. Entrez Gene ID(s) 1174
0.82	ZNF253	zinc finger protein 253
0.82	ADD2	adducin 2 (beta)
0.82	OXNAD1	oxidoreductase NAD-binding domain containing 1
0.82	NAIF1	nuclear apoptosis inducing factor 1
0.82	CA9	carbonic anhydrase IX
0.82	PTGES3	prostaglandin E synthase 3 (cytosolic)
0.82	CAAP1	caspase activity and apoptosis inhibitor 1
0.82	NELFCD	negative elongation factor complex member C/D
0.82	OR13G1	olfactory receptor. family 13. subfamily G. member 1
0.82	MED18	mediator complex subunit 18
0.82	ZFC3H1	zinc finger. C3H1-type containing
0.82	SLC45A2	solute carrier family 45. member 2
0.82	ZDHHC3	zinc finger. DHHC-type containing 3
0.82	SH2D3A	SH2 domain containing 3A
0.82	OSCP1	organic solute carrier partner 1
0.82	PFKM	phosphofructokinase. muscle
0.82	GABRB3	gamma-aminobutyric acid (GABA) A receptor. beta 3
0.82	BSX	brain-specific homeobox

0.82	SBK1	SH3 domain binding kinase 1
0.82	SHANK3	SH3 and multiple ankyrin repeat domains 3
0.82	COX5B	cytochrome c oxidase subunit Vb
0.82	IRX1	iroquois homeobox 1
0.82	OBSL1	obscurin-like 1
0.82	SLC1A1	solute carrier family 1 (neuronal/epithelial high affinity glutamate transporter. system Xag). member 1
0.82	OR51S1	olfactory receptor. family 51. subfamily S. member 1
0.82	PTGDR	prostaglandin D2 receptor (DP)
0.82	ZFR	Transcript Identified by AceView. Entrez Gene ID(s) 51663
0.82	TMEM150C	transmembrane protein 150C
0.82	TM6SF1	transmembrane 6 superfamily member 1
0.82	GPN3	GPN-loop GTPase 3
0.82	C6orf191andARHGAP18; RP1-69D17.4; RP1-69D17.3; TCONS_l2_00025470; TCONS_l2_00024911; LAMA2	Transcript Identified by AceView. Entrez Gene ID(s) 93663; 253582; Jeck2013 ALT_ACCEPTOR. ALT_DONOR. downstream_end. ncRNA. OVEXON. upstream_start best transcript TCONS_l2_00025470; Jeck2013 ALT_ACCEPTOR. ncRNA. OVEXON. upstream_start best transcript TCONS_l2_00024911; Salzman2013 ANTISENSE. CDS. coding. downstream_end. intronic. OVCODE. OVEXON. UTR3 best transcript NM_000426; Salzman2013 ANNOTATED. INTERNAL. ncRNA. OVEXON best transcript TCONS_l2_00025470; putative novel transcript; novel transcript
0.82	CLRN3	clarin 3
0.82	DAPL1; OR7E89P	death associated protein like 1; olfactory receptor. family 7. subfamily E. member 89 pseudogene
0.82	FAM92B	family with sequence similarity 92. member B
0.82	MAVS	Jeck2013 ANTISENSE. coding. INTERNAL. OVEXON. UTR3 best transcript NM_020746
0.82	C11orf45	chromosome 11 open reading frame 45
0.82	TEKT5	tektin 5

0.82	HNRNPUL2	heterogeneous nuclear ribonucleoprotein U-like 2
0.82	ZNF628	zinc finger protein 628
0.82	GLTSCR1	glioma tumor suppressor candidate region gene 1
0.82	FAM162B	family with sequence similarity 162. member B
0.82	REEP4	receptor accessory protein 4
0.82	GPR107	G protein-coupled receptor 107
0.82	UBA1	ubiquitin-like modifier activating enzyme 1
0.82	HSD17B14	hydroxysteroid (17-beta) dehydrogenase 14
0.82	FOSB	FBJ murine osteosarcoma viral oncogene homolog B
0.82	THBD	thrombomodulin
0.82	ABCF3	ATP binding cassette subfamily F member 3
0.82	DUSP10	dual specificity phosphatase 10
0.82	MEIS1	Meis homeobox 1
0.82	REXO4	REX4 homolog. 3'-5' exonuclease [Source:HGNC Symbol;Acc:HGNC:12820]
0.82	KPNB1	Memczak2013 ANTISENSE. CDS. coding. INTERNAL best transcript NM_002265
0.82	MBNL3	muscleblind-like splicing regulator 3
0.82	RNF20	ring finger protein 20. E3 ubiquitin protein ligase
0.82	ARL15	ADP-ribosylation factor like GTPase 15
0.82	IFT27	intraflagellar transport 27
0.82	BOLA2B; BOLA2	bolA family member 2B; bolA family member 2
0.82	PPP6R3	Memczak2013 ALT_ACCEPTOR. ALT_DONOR. coding. INTERNAL. intronic best transcript NM_001164162
0.82	POLR2L	polymerase (RNA) II (DNA directed) polypeptide L. 7.6kDa
0.82	QRFPR	pyroglutamylated RFamide peptide receptor
0.82	SARS2	seryl-tRNA synthetase 2. mitochondrial
0.82	LAMTOR4	late endosomal/lysosomal adaptor. MAPK and MTOR activator 4

0.82	ARHGAP11B	Rho GTPase activating protein 11B
0.82	GEMIN2	gem nuclear organelle associated protein 2
0.82	PNKP	polynucleotide kinase 3-phosphatase
0.82	NMRAL1	NmrA-like family domain containing 1
0.82	ORMDL1	ORMDL sphingolipid biosynthesis regulator 1
0.82	C1orf131	chromosome 1 open reading frame 131
0.82	COL26A1	collagen. type XXVI. alpha 1
0.82	PCDHB11	protocadherin beta 11
0.82	IL27	interleukin 27
0.82	PELI1	pellino E3 ubiquitin protein ligase 1
0.82	EDN3	endothelin 3
0.82	ZNF513	zinc finger protein 513
0.82	PTDSS1	Memczak2013 ANTISENSE. coding. INTERNAL. intronic best transcript NM_014754
0.82	DESI1	desumoylating isopeptidase 1
0.82	AMDHD1	amidohydrolase domain containing 1
0.82	FAM19A4	family with sequence similarity 19 (chemokine (C-C motif)-like). member A4
0.82	OR5H1	olfactory receptor. family 5. subfamily H. member 1
0.82	ZNF837	zinc finger protein 837
0.82	GSAP	gamma-secretase activating protein
0.82	C1orf61	chromosome 1 open reading frame 61
0.82	TMEM14A	transmembrane protein 14A
0.82	CTXN2	cortexin 2
0.82	GOLGA6L22; GOLGA6L1	golgin A6 family-like 22; golgin A6 family-like 1
0.82	NOSIP	nitric oxide synthase interacting protein
0.82	PPP2R5B	protein phosphatase 2. regulatory subunit B. beta

0.82	ANKEF1	ankyrin repeat and EF-hand domain containing 1
0.82	NKX3-1	NK3 homeobox 1
0.82	PLCZ1	phospholipase C. zeta 1
0.82	PKMYT1	protein kinase. membrane associated tyrosine/threonine 1
0.82	TECPR2	tectonin beta-propeller repeat containing 2
0.82	MEN1	Jeck2013 ANTISENSE. CDS. coding. INTERNAL. OVCODE. OVEXON best transcript NM_130803
0.82	PPEF1	protein phosphatase. EF-hand calcium binding domain 1
0.82	ST6GALNAC5	ST6 (alpha-N-acetyl-neuraminyl-2.3-beta-galactosyl-1.3)-N-acetylgalactosaminide alpha-2.6-sialyltransferase 5
0.82	NPY5R	neuropeptide Y receptor Y5
0.82	FAM117B	family with sequence similarity 117. member B
0.82	CAV1	caveolin 1
0.82	FAM53B	family with sequence similarity 53. member B
0.82	RAD9A	RAD9 checkpoint clamp component A
0.82	KIF15	kinesin family member 15
0.82	CP	ceruloplasmin (ferroxidase)
0.82	TLR5	toll-like receptor 5
0.82	CFAP47	cilia and flagella associated protein 47
0.82	PMS2P3	PMS1 homolog 2. mismatch repair system component pseudogene 3
0.82	ISY1-RAB43	ISY1-RAB43 readthrough
0.82	HIVEP3	human immunodeficiency virus type I enhancer binding protein 3
0.82	TRIM24	tripartite motif containing 24
0.82	DLK1	delta-like 1 homolog (Drosophila)
0.82	ITFG2	integrin alpha FG-GAP repeat containing 2
0.82	C11orf84	chromosome 11 open reading frame 84

0.82	CST9L	cystatin 9-like
0.82	SNAI3	snail family zinc finger 3
0.82	TMEM179B; MIR6748	transmembrane protein 179B; microRNA 6748
0.82	BMP4	bone morphogenetic protein 4
0.81	PSMC6	Memczak2013 ALT_ACCEPTOR. ALT_DONOR. coding. INTERNAL. intronic best transcript NM_002806
0.81	CYB561D2	cytochrome b561 family. member D2
0.81	PIP5K1C	phosphatidylinositol-4-phosphate 5-kinase. type I. gamma
0.81	VPS33A	vacuolar protein sorting 33 homolog A (S. cerevisiae)
0.81	RWDD2A	RWD domain containing 2A
0.81	ZFPL1	zinc finger protein-like 1
0.81	GPR52	G protein-coupled receptor 52
0.81	C15orf40	chromosome 15 open reading frame 40
0.81	ATP7B	ATPase. Cu++ transporting. beta polypeptide
0.81	AHNAK	AHNAK nucleoprotein
0.81	NPEPPS	aminopeptidase puromycin sensitive
0.81	ONECUT1	one cut homeobox 1
0.81	GTPBP6	GTP binding protein 6 (putative)
0.81	TUBA1C	tubulin. alpha 1c
0.81	CRNKL1	crooked neck pre-mRNA splicing factor 1
0.81	KIF12	kinesin family member 12
0.81	CHAF1A	chromatin assembly factor 1. subunit A (p150)
0.81	LYPLA2	lysophospholipase II
0.81	ALDH1A1	aldehyde dehydrogenase 1 family. member A1
0.81	ADI1	acireductone dioxygenase 1
0.81	DDOST	dolichyl-diphosphooligosaccharide--protein glycosyltransferase subunit (non-catalytic)

0.81	SLC5A4	solute carrier family 5 (glucose activated ion channel). member 4
0.81	TRPV1	transient receptor potential cation channel. subfamily V. member 1
0.81	OR7G1	olfactory receptor. family 7. subfamily G. member 1
0.81	GCSAM	germinal center-associated. signaling and motility
0.81	MTFR1	mitochondrial fission regulator 1
0.81	ANKHD1; EIF4EBP3; ANKHD1-EIF4EBP3	ankyrin repeat and KH domain containing 1; eukaryotic translation initiation factor 4E binding protein 3; ANKHD1-EIF4EBP3 readthrough
0.81	SURF4	surfeit 4
0.81	HERC1	HECT and RLD domain containing E3 ubiquitin protein ligase family member 1
0.81	SPAG16	sperm associated antigen 16
0.81	ZNF491	zinc finger protein 491
0.81	HMG20A	Transcript Identified by AceView. Entrez Gene ID(s) 10363
0.81	AKAP7	A kinase (PRKA) anchor protein 7
0.81	RBM6	RNA binding motif protein 6
0.81	FAM174B	family with sequence similarity 174. member B
0.81	ANKRD29	ankyrin repeat domain 29
0.81	KCNN4	potassium channel. calcium activated intermediate/small conductance subfamily N alpha. member 4
0.81	MAP2K2	mitogen-activated protein kinase kinase 2
0.81	GTF2H2	general transcription factor IIH subunit 2
0.81	RNPC3	RNA binding region (RNP1. RRM) containing 3
0.81	KHDC1L	KH homology domain containing 1-like
0.81	PLIN5	perilipin 5
0.81	COG4	component of oligomeric golgi complex 4
0.81	ASPHD2	aspartate beta-hydroxylase domain containing 2
0.81	RIMS4	regulating synaptic membrane exocytosis 4

0.81	GATSL2	GATS protein-like 2
0.81	FOXG1	forkhead box G1
0.81	PHACTR2	phosphatase and actin regulator 2
0.81	ICAM5	intercellular adhesion molecule 5
0.81	HTRA3	Transcript Identified by AceView. Entrez Gene ID(s) 94031
0.81	ANKLE1	ankyrin repeat and LEM domain containing 1
0.81	MSH2	mutS homolog 2
0.81	C21orf33	chromosome 21 open reading frame 33
0.81	RASA4	Salzman2013 ALT_ACCEPTOR. ALT_DONOR. coding. INTERNAL. intronic best transcript NM_006989
0.81	CROCC2	ciliary rootlet coiled-coil. rootletin family member 2 [Source:HGNC Symbol;Acc:HGNC:51677]
0.81	TECTA	tectorin alpha
0.81	ZBED9	zinc finger. BED-type containing 9
0.81	BCL2L14	BCL2-like 14 (apoptosis facilitator)
0.81	POLR2K	polymerase (RNA) II (DNA directed) polypeptide K. 7.0kDa
0.81	SYNGR1	synaptogyrin 1
0.81	LPAR4	lysophosphatidic acid receptor 4
0.81	PLCB1	Memczak2013 ANTISENSE. coding. INTERNAL. intronic best transcript NM_182734
0.81	ANK2	ankyrin 2. neuronal
0.81	C6orf203	chromosome 6 open reading frame 203
0.81	BAIAP3	BAI1-associated protein 3
0.81	RASL11A	RAS-like. family 11. member A
0.81	TMEM218	transmembrane protein 218
0.81	TCERG1	Transcript Identified by AceView. Entrez Gene ID(s) 10915
0.81	ANKRD39	ankyrin repeat domain 39

0.81	NPRL2	NPR2-like. GATOR1 complex subunit
0.81	SYNDIG1L	synapse differentiation inducing 1-like
0.81	ZDHHC24	zinc finger. DHHC-type containing 24
0.81	TBC1D3H; TBC1D3L; TBC1D3I; LOC101060389; TBC1D3C; TBC1D3; TBC1D3D	Homo sapiens TBC1 domain family. member 3H (TBC1D3H). mRNA.; Homo sapiens TBC1 domain family. member 3L (TBC1D3L). mRNA.; Homo sapiens TBC1 domain family. member 3I (TBC1D3I). mRNA.; Homo sapiens TBC1 domain family member-like (LOC101060389). mRNA.; TBC1 domain family member-like; Homo sapiens TBC1 domain family. member 3C. mRNA (cDNA clone MGC:87891 IMAGE:5165385). complete cds.; Homo sapiens TBC1 domain family. member 3. mRNA (cDNA clone MGC:87893 IMAGE:5742850). complete cds.; TBC1 domain family. member 3D [Source:HGNC Symbol;Acc:HGNC:28944]
0.81	ABHD11	abhydrolase domain containing 11
0.81	SNX21	sorting nexin family member 21
0.81	MRPS27	mitochondrial ribosomal protein S27
0.81	COX7B	cytochrome c oxidase subunit VIIb
0.81	DNAH2	dynein. axonemal. heavy chain 2
0.81	KLHL22	kelch-like family member 22
0.81	PREB	prolactin regulatory element binding
0.81	GPR119	G protein-coupled receptor 119
0.81	STMN3	stathmin-like 3
0.81	CT83	cancer/testis antigen 83
0.81	PPP1R12B	protein phosphatase 1. regulatory subunit 12B
0.81	STX10	syntaxin 10
0.81	MZT2A; MIR4784	mitotic spindle organizing protein 2A; microRNA 4784
0.81	DOK2	docking protein 2
0.81	PGC	progastricsin (pepsinogen C)
0.81	DHCR24	24-dehydrocholesterol reductase
0.81	IBA57	IBA57 homolog. iron-sulfur cluster assembly

0.81	SLC2A2	solute carrier family 2 (facilitated glucose transporter). member 2
0.81	OR4F3	olfactory receptor. family 4. subfamily F. member 3
0.81	GPX8	glutathione peroxidase 8 (putative)
0.81	C8orf59	chromosome 8 open reading frame 59
0.81	RBBP6	retinoblastoma binding protein 6
0.81	CENPK	centromere protein K
0.81	MCFD2	multiple coagulation factor deficiency 2
0.81	ATP6V0C	ATPase. H+ transporting. lysosomal 16kDa. V0 subunit c
0.81	PAPOLA	poly(A) polymerase alpha
0.81	FLYWCH1	Transcript Identified by AceView. Entrez Gene ID(s) 84256
0.81	GRIP1	glutamate receptor interacting protein 1
0.81	LRRCC1	leucine rich repeat and coiled-coil centrosomal protein 1
0.81	CHIT1	chitinase 1 (chitotriosidase)
0.81	E2F1	E2F transcription factor 1
0.81	JOSD2	Josephin domain containing 2
0.81	MAGED4; MAGED4B; SNORA11D; SNORA11E	melanoma antigen family D4; melanoma antigen family D4B; small nucleolar RNA. H/ACA box 11D; small nucleolar RNA. H/ACA box 11E
0.81	IRAK3; MIR6502	interleukin 1 receptor associated kinase 3; microRNA 6502
0.81	CHST5	carbohydrate (N-acetylglucosamine 6-O) sulfotransferase 5
0.81	ZSCAN1	zinc finger and SCAN domain containing 1
0.81	DNAH1	dynein. axonemal. heavy chain 1
0.81	PITX2	paired-like homeodomain 2
0.81	FBLL1	fibrillarin-like 1
0.81	MIIP	migration and invasion inhibitory protein
0.81	TIGD4	tigger transposable element derived 4
0.81	BLNK	B-cell linker

0.81	GSG2	germ cell associated 2 (haspin)
0.81	PGF	placental growth factor
0.81	RWDD4	RWD domain containing 4
0.81	SPAG7	sperm associated antigen 7
0.81	DBF4	DBF4 zinc finger
0.81	ISY1	ISY1 splicing factor homolog
0.81	ST8SIA6	ST8 alpha-N-acetyl-neuraminide alpha-2.8-sialyltransferase 6
0.81	SCRN2	secernin 2
0.81	ESYT3	extended synaptotagmin-like protein 3
0.81	KIF15	kinesin family member 15
0.81	ERVK13-1	endogenous retrovirus group K13. member 1
0.81	YTHDF3	YTH N(6)-methyladenosine RNA binding protein 3
0.81	MAEL	maelstrom spermatogenic transposon silencer
0.81	CHCHD2	coiled-coil-helix-coiled-coil-helix domain containing 2
0.81	KIAA0895	KIAA0895
0.81	TMEM161A	transmembrane protein 161A
0.81	LRRC16A	Transcript Identified by AceView. Entrez Gene ID(s) 55604
0.81	LIN7B	lin-7 homolog B (C. elegans)
0.81	NPTXR	neuronal pentraxin receptor
0.81	LRR1; RHOQP1	leucine rich repeat protein 1; ras homolog family member Q pseudogene 1
0.81	GNPTG	N-acetylglucosamine-1-phosphate transferase. gamma subunit
0.81	OGFR	opioid growth factor receptor
0.81	GRB7	growth factor receptor bound protein 7
0.81	CNP	Memczak2013 ANTISENSE. CDS. coding. INTERNAL best transcript NM_033133
0.81	ATP1A3	ATPase. Na+/K+ transporting. alpha 3 polypeptide

0.81	KCNMB1	potassium channel subfamily M regulatory beta subunit 1
0.81	WDR90	WD repeat domain 90
0.81	PIP5K1C	Zhang2013 ALT_ACCEPTOR. ALT_DONOR. coding. INTERNAL. intronic best transcript NM_012398
0.81	SEC14L4	SEC14-like lipid binding 4
0.81	ANXA8L1; ANXA8	annexin A8-like 1; annexin A8
0.81	BABAM1	BRISC and BRCA1 A complex member 1
0.81	WBP1L	WW domain binding protein 1-like
0.81	GRM5	glutamate receptor. metabotropic 5
0.81	ACTL8	actin-like 8
0.81	IL3RA	Jeck2013 ANTISENSE. CDS. coding. INTERNAL. intronic. OVCODE. OVEXON best transcript NM_002183
0.81	RIBC1	RIB43A domain with coiled-coils 1
0.81	OXNAD1	oxidoreductase NAD-binding domain containing 1
0.81	PLK4	polo-like kinase 4
0.81	TCTN2	tectonic family member 2
0.81	TMEM253	transmembrane protein 253
0.81	TAGLN	transgelin
0.81	CNTNAP2	contactin associated protein-like 2
0.81	SLC25A2	solute carrier family 25 (mitochondrial carrier; ornithine transporter) member 2
0.81	DMRT1	doublesex and mab-3 related transcription factor 1
0.81	APEX2	Transcript Identified by AceView. Entrez Gene ID(s) 27301
0.81	FAM122C	family with sequence similarity 122C
0.81	ARHGAP22	Rho GTPase activating protein 22
0.81	ECHDC1	ethylmalonyl-CoA decarboxylase 1
0.81	PKMYT1	protein kinase. membrane associated tyrosine/threonine 1

0.81	AMPD3	adenosine monophosphate deaminase 3
0.81	PHF21B	PHD finger protein 21B
0.81	CMIP	Memczak2013 ANTISENSE. coding. INTERNAL. intronic best transcript NM_198390
0.81	LRRC41	leucine rich repeat containing 41
0.81	ADARB2	adenosine deaminase. RNA-specific. B2 (inactive)
0.81	SEMA3A	sema domain. immunoglobulin domain (Ig). short basic domain. secreted. (semaphorin) 3A
0.81	FPGS	folylpolyglutamate synthase
0.81	GNAT1	guanine nucleotide binding protein (G protein). alpha transducing activity polypeptide 1
0.81	PCNT	pericentrin
0.81	FUK	fucokinase
0.81	E2F2	E2F transcription factor 2
0.81	PFDN4	prefoldin subunit 4
0.81	DAND5	DAN domain family member 5. BMP antagonist
0.81	GALNS	galactosamine (N-acetyl)-6-sulfatase
0.81	MED30	mediator complex subunit 30
0.81	LDLRAD4	low density lipoprotein receptor class A domain containing 4
0.81	PRAMEF25; PRAMEF26	PRAME family member 25; PRAME family member 26
0.81	TMEM239; C20orf141	transmembrane protein 239; chromosome 20 open reading frame 141
0.81	LCMT2	leucine carboxyl methyltransferase 2
0.81	PRELID1	PRELI domain containing 1
0.81	PRDM12	PR domain containing 12
0.81	DMXL1	Dmx-like 1
0.81	ACOX2	acyl-CoA oxidase 2. branched chain
0.81	FRG2	FSHD region gene 2

0.81	TFPT	TCF3 (E2A) fusion partner (in childhood Leukemia)
0.81	YIPF3	Yip1 domain family member 3
0.81	L3MBTL1	l(3)mbt-like 1 (Drosophila)
0.81	C7orf73	chromosome 7 open reading frame 73
0.81	ZNF846	zinc finger protein 846
0.81	UNC13C	unc-13 homolog C (C. elegans)
0.81	KIAA1191	KIAA1191
0.81	MSX1	msh homeobox 1
0.81	HOXA4	homeobox A4
0.81	SCOC	short coiled-coil protein
0.81	SLC35A2	solute carrier family 35 (UDP-galactose transporter). member A2
0.81	PYCARD-AS1	PYCARD antisense RNA 1
0.81	RHBDL1	rhomboid. veinlet-like 1 (Drosophila)
0.81	DNASE2	deoxyribonuclease II. lysosomal
0.81	BCL6	B-cell CLL/lymphoma 6
0.81	CEP135	centrosomal protein 135kDa
0.81	ARTN	artemin
0.81	ATOH7	atonal bHLH transcription factor 7
0.81	GJD2	gap junction protein delta 2
0.81	RBM15	RNA binding motif protein 15
0.81	CYP2D6	cytochrome P450. family 2. subfamily D. polypeptide 6
0.81	DPY19L3	dpy-19-like 3 (C. elegans)
0.81	C12orf66	chromosome 12 open reading frame 66
0.81	PVRIG	poliovirus receptor related immunoglobulin domain containing
0.81	ACAT2	acetyl-CoA acetyltransferase 2
0.81	U2AF1L4	U2 small nuclear RNA auxiliary factor 1-like 4

0.81	MS4A6E	membrane-spanning 4-domains. subfamily A. member 6E
0.81	NDUFA3	NADH dehydrogenase (ubiquinone) 1 alpha subcomplex. 3. 9kDa
0.81	NCKAP5L	NCK-associated protein 5-like
0.81	ANP32D	acidic nuclear phosphoprotein 32 family member D
0.81	IFITM5	interferon induced transmembrane protein 5
0.81	ATPIF1	ATPase inhibitory factor 1
0.81	NATD1	N-acetyltransferase domain containing 1
0.81	DNAJB5	DnaJ (Hsp40) homolog. subfamily B. member 5
0.81	RHBDL2	rhomboid. veinlet-like 2 (Drosophila)
0.81	PCSK1N	proprotein convertase subtilisin/kexin type 1 inhibitor
0.81	SPATA6L	spermatogenesis associated 6-like
0.81	FCGR1A	Fc fragment of IgG. high affinity Ia. receptor (CD64)
0.81	TMEM102	transmembrane protein 102
0.81	COPB2	coatomer protein complex subunit beta 2 (beta prime)
0.81	LMBR1	limb development membrane protein 1
0.81	CD276	CD276 molecule
0.81	CDC34	cell division cycle 34
0.81	SLC2A6	solute carrier family 2 (facilitated glucose transporter). member 6 [Source:HGNC Symbol;Acc:HGNC:11011]
0.81	GAMT	guanidinoacetate N-methyltransferase
0.81	LPGAT1	lysophosphatidylglycerol acyltransferase 1
0.81	POTED	POTE ankyrin domain family. member D
0.81	MED22	mediator complex subunit 22
0.81	ALDH18A1	aldehyde dehydrogenase 18 family. member A1
0.81	ZNF143	zinc finger protein 143
0.81	SLC20A2	solute carrier family 20 (phosphate transporter). member 2

0.81	GCAT	glycine C-acetyltransferase
0.81	AC010894.3; SCRN3	novel transcript; Transcript Identified by AceView. Entrez Gene ID(s) 79634
0.81	ERAS	ES cell expressed Ras
0.81	BLCAP	bladder cancer associated protein
0.81	LINC01587	long intergenic non-protein coding RNA 1587
0.81	TSEN54	TSEN54 tRNA splicing endonuclease subunit
0.81	GPLD1	glycosylphosphatidylinositol specific phospholipase D1
0.81	TCEANC2; MIR4781	transcription elongation factor A (SII) N-terminal and central domain containing 2; microRNA 4781
0.81	ZNF728	zinc finger protein 728
0.81	LGALS8-AS1	LGALS8 antisense RNA 1
0.81	MTMR12	myotubularin related protein 12
0.81	CRAT	carnitine O-acetyltransferase
0.81	ATP2A3	Memczak2013 ANTISENSE. CDS. coding. INTERNAL best transcript NM_174956
0.81	RORA	Jeck2013 ALT_ACCEPTOR. ALT_DONOR. coding. INTERNAL. intronic best transcript NM_134261
0.81	SLC7A7	solute carrier family 7 (amino acid transporter light chain. y+L system). member 7
0.81	PIGM	phosphatidylinositol glycan anchor biosynthesis class M
0.81	UBE2H	ubiquitin conjugating enzyme E2H
0.81	LRRC49	leucine rich repeat containing 49
0.81	MRPL11	mitochondrial ribosomal protein L11
0.81	TPST2	tyrosylprotein sulfotransferase 2
0.81	OSBPL9	oxysterol binding protein-like 9
0.81	ATAT1	alpha tubulin acetyltransferase 1
0.81	PVRL3	poliovirus receptor-related 3

0.81	TMEM121	transmembrane protein 121
0.81	FAM166A	family with sequence similarity 166. member A
0.81	MDM1	Mdm1 nuclear protein
0.81	PARD6G	par-6 family cell polarity regulator gamma
0.81	TRA2B	transformer 2 beta homolog (Drosophila)
0.81	HCRTR1	hypocretin (orexin) receptor 1
0.81	WWP2	WW domain containing E3 ubiquitin protein ligase 2
0.81	DNAAF2	dynein. axonemal. assembly factor 2
0.81	PABPN1	poly(A) binding protein. nuclear 1
0.81	ANXA6	annexin A6
0.81	GSTA2	glutathione S-transferase alpha 2
0.81	MME	membrane metallo-endopeptidase
0.81	SPACA4	sperm acrosome associated 4
0.81	LINC01140	long intergenic non-protein coding RNA 1140
0.81	RAB37	RAB37. member RAS oncogene family
0.81	TPTE2	transmembrane phosphoinositide 3-phosphatase and tensin homolog 2
0.81	GLUD1	glutamate dehydrogenase 1
0.81	POTEB3; POTEB; AC136352.4	Homo sapiens POTE ankyrin domain family. member B3 (POTEB3). transcript variant 1. mRNA.; Homo sapiens POTE ankyrin domain family. member B. mRNA (cDNA clone MGC:119371 IMAGE:40006483). complete cds.; Homo sapiens POTE ankyrin domain family. member B. mRNA (cDNA clone MGC:119372 IMAGE:40006485). complete cds.; Homo sapiens POTE ankyrin domain family. member B. mRNA (cDNA clone MGC:119373 IMAGE:40006489). complete cds.; Homo sapiens POTE ankyrin domain family. member B. mRNA (cDNA clone MGC:156116 IMAGE:40006490). complete cds.
0.81	SPA17	sperm autoantigenic protein 17
0.81	TRAPPC1	trafficking protein particle complex 1
0.81	CHEK1	checkpoint kinase 1

0.81	C17orf51	chromosome 17 open reading frame 51
0.81	FITM1	fat storage-inducing transmembrane protein 1
0.81	MISP	mitotic spindle positioning
0.81	CLSTN2	calsyntenin 2
0.81	DGCR6L	DiGeorge syndrome critical region gene 6-like
0.80	PDLIM4	PDZ and LIM domain 4
0.80	MSMB	microseminoprotein. beta-
0.80	SAP30	Sin3A associated protein 30kDa
0.80	DSPP	dentin sialophosphoprotein
0.80	SCYL1	SCY1-like. kinase-like 1
0.80	SAP30L	SAP30-like
0.80	CASP9	caspase 9
0.80	TNK2	tyrosine kinase. non-receptor. 2
0.80	FAM196B	family with sequence similarity 196. member B
0.80	ZNF852	zinc finger protein 852
0.80	TNFRSF19	tumor necrosis factor receptor superfamily. member 19
0.80	C14orf178	chromosome 14 open reading frame 178
0.80	C11orf44	chromosome 11 open reading frame 44
0.80	ZC3H3	Transcript Identified by AceView. Entrez Gene ID(s) 23144
0.80	ROM1	retinal outer segment membrane protein 1
0.80	CEP250	centrosomal protein 250kDa
0.80	NUDT18	nudix hydrolase 18
0.80	DDX60L	DEAD (Asp-Glu-Ala-Asp) box polypeptide 60-like
0.80	ZNF672	zinc finger protein 672
0.80	PGLS	6-phosphogluconolactonase
0.80	LRRC56	leucine rich repeat containing 56

0.80	NEDD4L	neural precursor cell expressed. developmentally down-regulated 4-like. E3 ubiquitin protein ligase
0.80	TMEM101	transmembrane protein 101
0.80	GMPR2	guanosine monophosphate reductase 2
0.80	TAOK2	TAO kinase 2
0.80	AGPAT4	1-acylglycerol-3-phosphate O-acyltransferase 4
0.80	SIK2	salt-inducible kinase 2
0.80	MAGED4B; MAGED4; SNORA11D	melanoma antigen family D4B; melanoma antigen family D4; small nucleolar RNA. H/ACA box 11D
0.80	AQP11	aquaporin 11
0.80	FRMPD1	FERM and PDZ domain containing 1
0.80	GTF2H5	general transcription factor IIH subunit 5
0.80	MDFI	MyoD family inhibitor
0.80	MYO1F	myosin IF
0.80	ACO1	aconitase 1. soluble
0.80	SPC24	SPC24. NDC80 kinetochore complex component
0.80	POLDIP2	polymerase (DNA-directed). delta interacting protein 2
0.80	BBS4	Bardet-Biedl syndrome 4
0.80	C3orf56	chromosome 3 open reading frame 56
0.80	ACAD9	acyl-CoA dehydrogenase family. member 9
0.80	CNTFR	ciliary neurotrophic factor receptor
0.80	CELF6	CUGBP. Elav-like family member 6
0.80	UNC93A	unc-93 homolog A (C. elegans)
0.80	ACADS	acyl-CoA dehydrogenase. C-2 to C-3 short chain
0.80	CDK10	cyclin-dependent kinase 10
0.80	OTUD7A	OTU deubiquitinase 7A
0.80	FLYWCH2	FLYWCH family member 2

0.80	OR4F5	olfactory receptor. family 4. subfamily F. member 5
0.80	RALGAPA2	Ral GTPase activating protein. alpha subunit 2 (catalytic)
0.80	ERBB2	Salzman2013 ANNOTATED. coding. OVEXON. UTR5 best transcript NM_001005862
0.80	SULT1C2	sulfotransferase family 1C member 2
0.80	MDK	midkine (neurite growth-promoting factor 2)
0.80	KRTAP7-1	keratin associated protein 7-1 (gene/pseudogene)
0.80	MMD	monocyte to macrophage differentiation-associated
0.80	P2RY6	pyrimidinergic receptor P2Y. G-protein coupled. 6
0.80	ZSWIM8	zinc finger. SWIM-type containing 8
0.80	CCDC84	coiled-coil domain containing 84
0.80	HLA-C	major histocompatibility complex. class I. C
0.80	CTR9	CTR9 homolog. Paf1/RNA polymerase II complex component
0.80	MYD88	myeloid differentiation primary response 88
0.80	CXXC5	Memczak2013 ANTISENSE. coding. INTERNAL. intronic best transcript NM_016463
0.80	SLC25A47	solute carrier family 25. member 47
0.80	ZNF660	zinc finger protein 660
0.80	FANCD2OS	FANCD2 opposite strand
0.80	PUS10	pseudouridylate synthase 10
0.80	DUSP9	dual specificity phosphatase 9
0.80	IGF2-AS	IGF2 antisense RNA
0.80	KDM2B	lysine (K)-specific demethylase 2B
0.80	UNC80	unc-80 homolog (C. elegans)
0.80	UROC1	urocanate hydratase 1
0.80	C1orf115	chromosome 1 open reading frame 115
0.80	LGALS9C; LGALS9B	lectin. galactoside-binding. soluble. 9C; lectin. galactoside-binding. soluble.

		9B
0.80	KRTAP5-10	keratin associated protein 5-10
0.80	SRSF1	serine/arginine-rich splicing factor 1
0.80	AKR7A2	aldo-keto reductase family 7. member A2
0.80	PEX11A	peroxisomal biogenesis factor 11 alpha
0.80	PYCR1	pyrroline-5-carboxylate reductase 1
0.80	HNRNPA0	heterogeneous nuclear ribonucleoprotein A0
0.80	WBP1	WW domain binding protein 1
0.80	LAMTOR1	late endosomal/lysosomal adaptor. MAPK and MTOR activator 1
0.80	LOC101060389; TBC1D3D	TBC1 domain family member-like; TBC1 domain family. member 3D [Source:HGNC Symbol;Acc:HGNC:28944]
0.80	C17orf97	chromosome 17 open reading frame 97
0.80	RRM1	ribonucleotide reductase M1
0.80	PIGP	phosphatidylinositol glycan anchor biosynthesis class P
0.80	ZNF738	zinc finger protein 738
0.80	XKR9	X-linked Kx blood group related 9
0.80	COMMD4	COMM domain containing 4
0.80	IGSF8	immunoglobulin superfamily. member 8
0.80	C1orf21	chromosome 1 open reading frame 21
0.80	ZNF439	zinc finger protein 439
0.80	CYTH1	cytohesin 1
0.80	LOC650293	seven transmembrane helix receptor; Transcript Identified by AceView. Entrez Gene ID(s) 650293. RefSeq ID(s) NM_001040071
0.80	LRRC15	leucine rich repeat containing 15
0.80	SSX2IP	synovial sarcoma. X breakpoint 2 interacting protein
0.80	SLC30A8	solute carrier family 30 (zinc transporter). member 8
0.80	OXCT2P1	3-oxoacid CoA-transferase 2 pseudogene 1

0.80	VCP	valosin containing protein
0.80	STPG2	sperm-tail PG-rich repeat containing 2
0.80	WAS	Wiskott-Aldrich syndrome
0.80	KDM4A	lysine (K)-specific demethylase 4A
0.80	CACTIN-AS1	CACTIN antisense RNA 1
0.80	CDCA7	cell division cycle associated 7
0.80	SMG9	SMG9 nonsense mediated mRNA decay factor
0.80	SAMD4B	sterile alpha motif domain containing 4B
0.80	LSM3	LSM3 homolog. U6 small nuclear RNA and mRNA degradation associated
0.80	MOB1A	MOB kinase activator 1A
0.80	PPFIA2	protein tyrosine phosphatase. receptor type. f polypeptide (PTPRF). interacting protein (liprin). alpha 2
0.80	KLRG1	killer cell lectin-like receptor subfamily G. member 1
0.80	ZNF221	zinc finger protein 221
0.80	TGOLN2	Memczak2013 ANTISENSE. CDS. coding. INTERNAL best transcript NM_001206844
0.80	NECAB2	N-terminal EF-hand calcium binding protein 2
0.80	NRN1	neuritin 1
0.80	PCDHB6	protocadherin beta 6
0.80	PBRM1	polybromo 1
0.80	CCDC25	coiled-coil domain containing 25
0.80	RHBDF2	rhomboid 5 homolog 2 (Drosophila)
0.80	KRTAP3-1	keratin associated protein 3-1
0.80	CD300A	CD300a molecule
0.80	DENND2D	DENN/MADD domain containing 2D
0.80	PUSL1	pseudouridylate synthase-like 1
0.80	EXOC3	exocyst complex component 3

0.80	EBNA1BP2	EBNA1 binding protein 2
0.80	FAM83C	family with sequence similarity 83. member C
0.80	ITPKC	Transcript Identified by AceView. Entrez Gene ID(s) 80271
0.80	APOC1	apolipoprotein C-I
0.80	HNF1B	HNF1 homeobox B
0.80	UBAC1	UBA domain containing 1
0.80	ST7; ST7-OT4; ST7-OT3; MIR6132	suppression of tumorigenicity 7; ST7 overlapping transcript 4; ST7 overlapping transcript 3; microRNA 6132
0.80	TAF7L	TATA box binding protein associated factor 7 like
0.80	NPDC1	neural proliferation. differentiation and control. 1
0.80	ING5	inhibitor of growth family member 5
0.80	ZNF660	zinc finger protein 660
0.80	WTH3DI; LOC150786	RAB6C-like; Synthetic construct Homo sapiens clone IMAGE:100016523. MGC:184337 RAB6C-like (LOC150786) mRNA. encodes complete protein.
0.80	FAM231B	family with sequence similarity 231. member B
0.80	B4GALT4	UDP-Gal:betaGlcNAc beta 1.4- galactosyltransferase. polypeptide 4
0.80	RGAG4	retrotransposon gag domain containing 4
0.80	POC1A	POC1 centriolar protein A
0.80	MMRN2	multimerin 2
0.80	NDST1	N-deacetylase/N-sulfotransferase (heparan glucosaminyl) 1
0.80	LGALS9B	lectin. galactoside-binding. soluble. 9B
0.80	TIMMDC1	translocase of inner mitochondrial membrane domain containing 1
0.80	CDK1	cyclin-dependent kinase 1
0.80	TBC1D2	TBC1 domain family. member 2
0.80	SORBS3	sorbin and SH3 domain containing 3
0.80	C1orf229	chromosome 1 open reading frame 229
0.80	RAPSN	receptor-associated protein of the synapse

0.80	DRICH1	aspartate-rich 1
0.80	LILRA6	leukocyte immunoglobulin-like receptor. subfamily A (with TM domain). member 6
0.80	AMY2B; ACTG1P4	amylase. alpha 2B (pancreatic); actin gamma 1 pseudogene 4
0.80	TMEM79	transmembrane protein 79
0.80	EXOC3-AS1	EXOC3 antisense RNA 1
0.80	GYLTL1B	glycosyltransferase-like 1B
0.80	NCOA7	Transcript Identified by AceView. Entrez Gene ID(s) 135112
0.80	WDR5B	WD repeat domain 5B
0.80	SLC22A9	solute carrier family 22 (organic anion transporter). member 9
0.80	PIP	prolactin-induced protein
0.80	KLF15	Kruppel-like factor 15
0.80	LINC01496	long intergenic non-protein coding RNA 1496
0.80	NUF2	NUF2. NDC80 kinetochore complex component
0.80	CHP1	calcineurin-like EF-hand protein 1
0.80	PTTG1	pituitary tumor-transforming 1
0.80	EXOC7	Zhang2013 ALT_ACCEPTOR. ALT_DONOR. coding. INTERNAL. intronic best transcript NM_001145297
0.80	CCDC61	coiled-coil domain containing 61
0.80	PRAC1	prostate cancer susceptibility candidate 1
0.80	EIF4EBP2	eukaryotic translation initiation factor 4E binding protein 2
0.80	CAMKK1	calcium/calmodulin-dependent protein kinase kinase 1. alpha
0.80	HCFC1	host cell factor C1
0.80	SDK2	sidekick cell adhesion molecule 2
0.80	TTC38	tetratricopeptide repeat domain 38
0.80	LMAN2L	lectin. mannose-binding 2-like
0.80	CCDC130	coiled-coil domain containing 130

0.80	RPS6KA1	ribosomal protein S6 kinase. 90kDa. polypeptide 1
0.80	UBL5	ubiquitin-like 5
0.80	CDX4	caudal type homeobox 4
0.80	FAU	Finkel-Biskis-Reilly murine sarcoma virus (FBR-MuSV) ubiquitously expressed
0.80	XXYLT1	xyloside xylosyltransferase 1
0.80	PAGE4	P antigen family. member 4 (prostate associated)
0.80	TPGS2	tubulin polyglutamylase complex subunit 2
0.80	LDB3	LIM domain binding 3
0.80	PDE1C	phosphodiesterase 1C. calmodulin-dependent 70kDa
0.80	SPPL2C	signal peptide peptidase like 2C
0.80	MAP2K3	mitogen-activated protein kinase kinase 3
0.80	ST3GAL6	ST3 beta-galactoside alpha-2.3-sialyltransferase 6
0.80	C1QTNF3-AMACR	C1QTNF3-AMACR readthrough (NMD candidate)
0.80	CLCNKB	chloride channel. voltage-sensitive Kb
0.80	KCNB2	potassium channel. voltage gated Shab related subfamily B. member 2
0.80	ZNF45	zinc finger protein 45
0.80	ACBD7	acyl-CoA binding domain containing 7
0.80	ZNF138	zinc finger protein 138
0.80	TMEM151A	transmembrane protein 151A
0.80	LPAR1	lysophosphatidic acid receptor 1
0.80	TEX264	testis expressed 264
0.80	COX2	cytochrome c oxidase subunit II
0.80	FAM118A	family with sequence similarity 118. member A
0.80	TIMM10B	translocase of inner mitochondrial membrane 10 homolog B (yeast)
0.80	VIPAS39	VPS33B interacting protein. apical-basolateral polarity regulator. spe-39 homolog

0.80	LPO	lactoperoxidase
0.80	ADAM11	ADAM metallopeptidase domain 11
0.80	PDE10A	phosphodiesterase 10A
0.80	ZKSCAN1	zinc finger with KRAB and SCAN domains 1
0.80	DECR2	2.4-dienoyl-CoA reductase 2. peroxisomal
0.80	HSP90AA1	heat shock protein 90kDa alpha (cytosolic). class A member 1
0.80	FAM72D	family with sequence similarity 72. member D
0.80	ENPP4	ectonucleotide pyrophosphatase/phosphodiesterase 4 (putative)
0.80	ZDHHC11	Transcript Identified by AceView. Entrez Gene ID(s) 79844
0.80	SRRM2	serine/arginine repetitive matrix 2
0.80	SFXN2	sideroflexin 2
0.80	PAMR1	peptidase domain containing associated with muscle regeneration 1
0.80	FKBP3	FK506 binding protein 3
0.80	CTSD	cathepsin D
0.80	FCRL1	Fc receptor-like 1
0.80	OBP2B	odorant binding protein 2B
0.80	TMEM35	transmembrane protein 35
0.80	MAML1	mastermind-like transcriptional coactivator 1
0.80	FAM127A	family with sequence similarity 127. member A
0.80	ZNF211	zinc finger protein 211
0.80	FBXL22	F-box and leucine-rich repeat protein 22
0.80	NDUFA7	NADH dehydrogenase (ubiquinone) 1 alpha subcomplex. 7. 14.5kDa
0.80	BAG1	BCL2-associated athanogene
0.80	CA1	carbonic anhydrase I
0.80	SURF4	surfeit 4
0.80	C11orf40	chromosome 11 open reading frame 40

0.80	FGD3	FYVE. RhoGEF and PH domain containing 3
0.80	CECR6	cat eye syndrome chromosome region. candidate 6
0.80	RHOXF2B; RHOXF2	Rhox homeobox family. member 2B; Rhox homeobox family. member 2
0.80	PTK7	protein tyrosine kinase 7 (inactive)
0.80	DDX58	DEAD (Asp-Glu-Ala-Asp) box polypeptide 58
0.80	PHYHIP	phytanoyl-CoA 2-hydroxylase interacting protein
0.80	TBXAS1	thromboxane A synthase 1 (platelet)
0.80	LYPD3	LY6/PLAUR domain containing 3
0.80	TPRA1	transmembrane protein. adipocyte asscociated 1
0.80	TIGD3	tigger transposable element derived 3
0.80	NBPF7	neuroblastoma breakpoint family. member 7
0.80	CASZ1	castor zinc finger 1
0.80	UCK1	uridine-cytidine kinase 1
0.80	RCAN1	regulator of calcineurin 1
0.80	RBM14-RBM4	RBM14-RBM4 readthrough
0.80	CDH17	cadherin 17. LI cadherin (liver-intestine)
0.80	TMC4	transmembrane channel like 4
0.80	SAMD4A	Jeck2013 ALT_ACCEPTOR. ALT_DONOR. coding. INTERNAL. intronic best transcript NM_015589
0.80	MGST1	microsomal glutathione S-transferase 1
0.80	KRTAP1-4	keratin associated protein 1-4
0.80	ZNF362	zinc finger protein 362
0.80	CGN	cingulin
0.80	HIST2H2BE	histone cluster 2. H2be
0.80	COLEC11	collectin subfamily member 11
0.80	PLEKHA2	pleckstrin homology domain containing. family A (phosphoinositide binding specific) member 2

0.80	PCYOX1L	prenylcysteine oxidase 1 like
0.79	SH2D1B	SH2 domain containing 1B
0.79	KLHL34	kelch-like family member 34
0.79	LPL	lipoprotein lipase
0.79	GPC2	glypican 2
0.79	STXBP2	syntaxin binding protein 2
0.79	LOC102723701; RP11-863K10.7	uncharacterized LOC102723701; novel transcript. antisense to ERLIN2
0.79	NTN3	netrin 3
0.79	NCAPG2	non-SMC condensin II complex subunit G2
0.79	NUDT12	nudix hydrolase 12
0.79	ALDOC	aldolase C. fructose-bisphosphate
0.79	GLYATL1	glycine-N-acyltransferase-like 1
0.79	MTRNR2L8; MIR4485	MT-RNR2-like 8; microRNA 4485
0.79	ZNF648	zinc finger protein 648
0.79	NINL	ninein-like
0.79	POLI	polymerase (DNA directed) iota
0.79	NCF4	neutrophil cytosolic factor 4
0.79	ZNF843	zinc finger protein 843
0.79	PP2D1	protein phosphatase 2C-like domain containing 1
0.79	POLE3	polymerase (DNA directed). epsilon 3. accessory subunit
0.79	SLC35E2	solute carrier family 35. member E2
0.79	ERP27	endoplasmic reticulum protein 27
0.79	TMEM125	transmembrane protein 125
0.79	RC3H1	ring finger and CCCH-type domains 1
0.79	SLC22A31	solute carrier family 22. member 31
0.79	PIK3C2A	phosphatidylinositol-4-phosphate 3-kinase. catalytic subunit type 2 alpha

0.79	MBOAT4	membrane bound O-acyltransferase domain containing 4
0.79	ENSA	endosulfine alpha
0.79	DHCR7	7-dehydrocholesterol reductase
0.79	KLK2	kallikrein related peptidase 2
0.79	MAPK8IP2	mitogen-activated protein kinase 8 interacting protein 2
0.79	STOX2	storkhead box 2
0.79	ADCY2	Transcript Identified by AceView. Entrez Gene ID(s) 108
0.79	SHANK2-AS3	SHANK2 antisense RNA 3
0.79	DNAJB11	DnaJ (Hsp40) homolog. subfamily B. member 11
0.79	CLIC3	chloride intracellular channel 3
0.79	DGCR14	DiGeorge syndrome critical region gene 14
0.79	PRRT3	proline-rich transmembrane protein 3
0.79	LEXM	lymphocyte expansion molecule
0.79	CEACAM7	carcinoembryonic antigen-related cell adhesion molecule 7
0.79	MCM2	minichromosome maintenance complex component 2
0.79	ADPRHL2	ADP-ribosylhydrolase like 2
0.79	ATG16L1	autophagy related 16-like 1
0.79	IQCF1	IQ motif containing F1
0.79	TRO	trophinin
0.79	HMOX1	heme oxygenase 1
0.79	PLAGL1; HYMAI	pleiomorphic adenoma gene-like 1; hydatidiform mole associated and imprinted (non-protein coding)
0.79	PRG2	proteoglycan 2. bone marrow (natural killer cell activator. eosinophil granule major basic protein)
0.79	XPNPEP2	X-prolyl aminopeptidase (aminopeptidase P) 2. membrane-bound
0.79	ZNF705B	zinc finger protein 705B
0.79	PPOX	protoporphyrinogen oxidase

0.79	FAM206A	family with sequence similarity 206. member A
0.79	C2orf72	chromosome 2 open reading frame 72
0.79	L2HGDH	L-2-hydroxyglutarate dehydrogenase
0.79	COMMD9	COMM domain containing 9
0.79	GALK1	galactokinase 1
0.79	ABCD1	ATP binding cassette subfamily D member 1
0.79	SLC44A3	solute carrier family 44. member 3
0.79	SSH2	slingshot protein phosphatase 2
0.79	SAMHD1	SAM domain and HD domain 1
0.79	NPHP4	nephronophthisis 4
0.79	GALR2	galanin receptor 2
0.79	GTPBP6	Homo sapiens GTP binding protein 6 (putative) (GTPBP6). mRNA.; GTP binding protein 6 (putative) [Source:HGNC Symbol;Acc:HGNC:30189]; Homo sapiens GTP binding protein 6 (putative). mRNA (cDNA clone MGC:1170 IMAGE:3528598). complete cds.; Salzman2013 ANNOTATED. CDS. coding. INTERNAL. OVCODE. OVERLAPTX. OVEXON best transcript NM_012227
0.79	GDF7	growth differentiation factor 7
0.79	CXCR4	chemokine (C-X-C motif) receptor 4
0.79	KLHL10	kelch-like family member 10
0.79	MCM9	minichromosome maintenance 9 homologous recombination repair factor
0.79	LOC283710; RP11-16E12.2; rukama	uncharacterized LOC283710; novel transcript; Transcript Identified by AceView
0.79	PQLC1	PQ loop repeat containing 1
0.79	PRSS21	protease. serine. 21 (testisin)
0.79	MRAP	melanocortin 2 receptor accessory protein
0.79	GRIPAP1	GRIP1 associated protein 1
0.79	NDUFB10	NADH dehydrogenase (ubiquinone) 1 beta subcomplex. 10. 22kDa
0.79	SGSH	N-sulfoglucosamine sulfohydrolase

0.79	FCRLB	Fc receptor-like B
0.79	HSDL2	hydroxysteroid dehydrogenase like 2
0.79	ZNRF4	zinc and ring finger 4
0.79	EPM2A	epilepsy. progressive myoclonus type 2A. Lafora disease (laforin)
0.79	SLC15A3	solute carrier family 15 (oligopeptide transporter). member 3
0.79	VRK3	vaccinia related kinase 3
0.79	FAM83H-AS1	FAM83H antisense RNA 1 (head to head)
0.79	C1orf122	chromosome 1 open reading frame 122
0.79	TNFSF13B	tumor necrosis factor (ligand) superfamily. member 13b
0.79	CEP295	centrosomal protein 295kDa
0.79	NUP107	nucleoporin 107kDa
0.79	SDSL	serine dehydratase-like
0.79	FAM110D	family with sequence similarity 110. member D
0.79	TSSK6	testis-specific serine kinase 6
0.79	CETN3	centrin 3
0.79	MANEAL	mannosidase. endo-alpha-like
0.79	ITGB6	integrin beta 6
0.79	FOXD4L6	forkhead box D4-like 6
0.79	GJA1	gap junction protein alpha 1
0.79	WT1	Wilms tumor 1
0.79	VIMP	VCP-interacting membrane selenoprotein
0.79	IZUMO4	IZUMO family member 4
0.79	H2BFM	H2B histone family. member M
0.79	KIF7	kinesin family member 7
0.79	RMI2	RecQ mediated genome instability 2
0.79	CYTB	cytochrome b

0.79	RAX2	retina and anterior neural fold homeobox 2
0.79	CHCHD7	coiled-coil-helix-coiled-coil-helix domain containing 7
0.79	SIGLEC6	sialic acid binding Ig-like lectin 6
0.79	TJP3	tight junction protein 3
0.79	SRGAP2C	SLIT-ROBO Rho GTPase activating protein 2C
0.79	C11orf98; LBHD1	chromosome 11 open reading frame 98; LBH domain containing 1
0.79	TM2D2	TM2 domain containing 2
0.79	DIRC1	disrupted in renal carcinoma 1
0.79	NLGN4X	neuroligin 4. X-linked
0.79	PSMC6	proteasome 26S subunit. ATPase 6
0.79	PHLPP1	PH domain and leucine rich repeat protein phosphatase 1
0.79	KIAA0319L	KIAA0319-like
0.79	SPATA21	spermatogenesis associated 21
0.79	ORC6	origin recognition complex subunit 6
0.79	ZNF345	zinc finger protein 345
0.79	ECEL1	endothelin converting enzyme-like 1
0.79	CERCAM	cerebral endothelial cell adhesion molecule
0.79	ATP6V1D	ATPase. H+ transporting. lysosomal 34kDa. V1 subunit D
0.79	PNPLA7	Transcript Identified by AceView. Entrez Gene ID(s) 375775
0.79	STAT4	signal transducer and activator of transcription 4
0.79	MALSU1	mitochondrial assembly of ribosomal large subunit 1
0.79	GPR50	G protein-coupled receptor 50
0.79	RSPH10B2	radial spoke head 10 homolog B2 (Chlamydomonas)
0.79	EVL	Enah/Vasp-like
0.79	FBN3	fibrillin 3
0.79	C2orf15andLIPT1andMRPL30;	Transcript Identified by AceView. Entrez Gene ID(s) 150590; 51601; 51263; chromosome 2 open reading frame 15 [Source:HGNC

	C2orf15	Symbol;Acc:HGNC:28436]
0.79	SLC16A4	solute carrier family 16. member 4
0.79	OCEL1	occludin/ELL domain containing 1
0.79	ANKRD24	ankyrin repeat domain 24
0.79	EBF3	early B-cell factor 3
0.79	DNAJC25	DnaJ (Hsp40) homolog. subfamily C . member 25
0.79	TSC22D3	TSC22 domain family. member 3
0.79	PHF7	PHD finger protein 7
0.79	LMF1	lipase maturation factor 1
0.79	FZD5	frizzled class receptor 5
0.79	CD5	CD5 molecule
0.79	ATP2A3	ATPase. Ca++ transporting. ubiquitous
0.79	HDAC4	Transcript Identified by AceView. Entrez Gene ID(s) 9759
0.79	ARL3	ADP-ribosylation factor like GTPase 3
0.79	SKA3	spindle and kinetochore associated complex subunit 3
0.79	C1orf194	chromosome 1 open reading frame 194
0.79	TUBG1	tubulin. gamma 1
0.79	HIST1H4I	histone cluster 1. H4i
0.79	CDNF	cerebral dopamine neurotrophic factor
0.79	CREB3L4	cAMP responsive element binding protein 3-like 4
0.79	PGA5	pepsinogen 5. group I (pepsinogen A)
0.79	ZNF90	zinc finger protein 90
0.79	IRF7	interferon regulatory factor 7
0.79	DNAJC27	DnaJ (Hsp40) homolog. subfamily C. member 27
0.79	UQCR11	ubiquinol-cytochrome c reductase. complex III subunit XI
0.79	LOC115110; RP3-395M20.8	uncharacterized LOC115110; novel transcript

0.79	LCE4A	late cornified envelope 4A
0.79	PROSC	proline synthetase co-transcribed homolog (bacterial)
0.79	EML2-AS1	EML2 antisense RNA 1
0.79	UFD1L	ubiquitin fusion degradation 1 like (yeast)
0.79	SYNGAP1; MIR5004	synaptic Ras GTPase activating protein 1; microRNA 5004
0.79	STX4	syntaxin 4
0.79	ANGEL2	angel homolog 2 (Drosophila)
0.79	C8orf86	chromosome 8 open reading frame 86
0.79	DAXX	death-domain associated protein
0.79	LINC00311	long intergenic non-protein coding RNA 311
0.79	SEC24D	SEC24 homolog D. COPII coat complex component
0.79	FKBP1A-SDCBP2	FKBP1A-SDCBP2 readthrough (NMD candidate)
0.79	COA3	cytochrome c oxidase assembly factor 3
0.79	PTPRS	protein tyrosine phosphatase. receptor type. S
0.79	SLC16A7	solute carrier family 16 (monocarboxylate transporter). member 7
0.79	PHF12	PHD finger protein 12
0.79	CCDC183; RABL6	coiled-coil domain containing 183; RAB. member RAS oncogene family-like 6
0.79	HTR3B	5-hydroxytryptamine (serotonin) receptor 3B. ionotropic
0.79	ULBP3	UL16 binding protein 3
0.79	ZNF546	zinc finger protein 546
0.79	PF4	platelet factor 4
0.79	GSTCD	glutathione S-transferase. C-terminal domain containing
0.79	SCARB1	scavenger receptor class B. member 1
0.79	MTMR7	myotubularin related protein 7
0.79	IFNA2	interferon. alpha 2
0.79	HOXB1	homeobox B1

0.79	CCDC92	coiled-coil domain containing 92
0.79	SLC35E2B	solute carrier family 35. member E2B
0.79	XRCC1	X-ray repair complementing defective repair in Chinese hamster cells 1
0.79	INO80B-WBP1	INO80B-WBP1 readthrough (NMD candidate)
0.79	OR10AD1	olfactory receptor. family 10. subfamily AD. member 1
0.79	BCL2L11	BCL2-like 11 (apoptosis facilitator)
0.79	TKTL2	transketolase-like 2
0.79	SYNDIG1	synapse differentiation inducing 1
0.79	ZNF701; ZNF137P	zinc finger protein 701; zinc finger protein 137. pseudogene
0.79	UBP1	upstream binding protein 1 (LBP-1a)
0.79	DHRS7C	dehydrogenase/reductase (SDR family) member 7C
0.79	PINLYP	phospholipase A2 inhibitor and LY6/PLAUR domain containing
0.79	LINC00935	long intergenic non-protein coding RNA 935
0.79	ZNF582-AS1	ZNF582 antisense RNA 1 (head to head)
0.79	RAP1A	RAP1A. member of RAS oncogene family
0.79	TSPEAR	thrombospondin-type laminin G domain and EAR repeats
0.79	SH3PXD2A	SH3 and PX domains 2A
0.79	ECHS1	enoyl-CoA hydratase. short chain. 1. mitochondrial
0.79	ASS1	argininosuccinate synthase 1
0.79	SEC22C	SEC22 homolog C. vesicle trafficking protein
0.79	FAM135B	family with sequence similarity 135. member B
0.79	TMEM27	transmembrane protein 27
0.79	MUC6	mucin 6. oligomeric mucus/gel-forming
0.79	TTLL5	tubulin tyrosine ligase-like family member 5
0.79	GFI1	growth factor independent 1 transcription repressor
0.79	MTTP	microsomal triglyceride transfer protein

0.79	ZYG11B	zyg-11 family member B. cell cycle regulator
0.79	TRAPPC4	trafficking protein particle complex 4
0.79	RNASE8	ribonuclease. RNase A family. 8
0.79	SLC38A11	solute carrier family 38. member 11
0.79	OCRL	oculocerebrorenal syndrome of Lowe
0.79	CCDC14	coiled-coil domain containing 14
0.79	RARG	retinoic acid receptor. gamma
0.79	OR1L6	olfactory receptor. family 1. subfamily L. member 6
0.79	FAM3B	family with sequence similarity 3. member B
0.79	GSTK1	glutathione S-transferase kappa 1
0.79	TRIM2	tripartite motif containing 2
0.79	MUC22	mucin 22
0.79	UBQLN1	ubiquilin 1
0.79	DMRTC1; FAM226B; FAM226A; DMRTC1B	DMRT-like family C1; family with sequence similarity 226. member B (non-protein coding); family with sequence similarity 226. member A (non-protein coding); DMRT-like family C1B
0.79	BUD13	BUD13 homolog
0.79	MIR4728; ERBB2	microRNA 4728; erb-b2 receptor tyrosine kinase 2
0.79	KIAA1683	KIAA1683
0.79	ATRN	attractin
0.79	CSRP1	cysteine and glycine-rich protein 1
0.79	NKX2-6	NK2 homeobox 6
0.79	ANKS1A	ankyrin repeat and sterile alpha motif domain containing 1A
0.79	EFHB	EF-hand domain family. member B

0.79	UGT1A1; UGT1A6; UGT1A4; UGT1A10; UGT1A8; UGT1A7; UGT1A5; UGT1A3; UGT1A9	UDP glucuronosyltransferase 1 family. polypeptide A1; UDP glucuronosyltransferase 1 family. polypeptide A6; UDP glucuronosyltransferase 1 family. polypeptide A4; UDP glucuronosyltransferase 1 family. polypeptide A10; UDP glucuronosyltransferase 1 family. polypeptide A8; UDP glucuronosyltransferase 1 family. polypeptide A7; UDP glucuronosyltransferase 1 family. polypeptide A5; UDP glucuronosyltransferase 1 family. polypeptide A3; UDP glucuronosyltransferase 1 family. polypeptide A9
0.79	SLC10A1	Memczak2013 ANTISENSE. CDS. coding. INTERNAL. intronic best transcript NM_003049
0.79	ZNF417	zinc finger protein 417
0.79	RAD18	RAD18 E3 ubiquitin protein ligase
0.79	MRPL12	mitochondrial ribosomal protein L12
0.79	SELK	selenoprotein K; selenoprotein K [Source:EntrezGene;Acc:58515]; Transcript Identified by AceView. Entrez Gene ID(s) 58515. RefSeq ID(s) NM_021237
0.79	DLK2	delta-like 2 homolog (Drosophila)
0.79	MRPL30	mitochondrial ribosomal protein L30
0.79	SIRT5	sirtuin 5
0.79	WDR88	WD repeat domain 88
0.79	VSTM5	V-set and transmembrane domain containing 5
0.79	CTBP1-AS2	CTBP1 antisense RNA 2 (head to head)
0.79	TMEM265; SRCAP	transmembrane protein 265; Snf2-related CREBBP activator protein
0.79	SLC39A11	solute carrier family 39. member 11
0.79	EMP2	epithelial membrane protein 2
0.79	LRRC52	leucine rich repeat containing 52
0.79	TTC39C	tetratricopeptide repeat domain 39C
0.79	PI4K2B	phosphatidylinositol 4-kinase type 2 beta
0.79	SLC27A1	solute carrier family 27 (fatty acid transporter). member 1

0.79	FBXO41	F-box protein 41
0.79	RAD9A	RAD9 checkpoint clamp component A
0.79	SCN10A	sodium channel. voltage gated. type X alpha subunit
0.79	NAA40	N(alpha)-acetyltransferase 40. NatD catalytic subunit
0.79	LIN9	lin-9 DREAM MuvB core complex component
0.79	PAQR4	progestin and adipoQ receptor family member IV
0.79	ALDH8A1	aldehyde dehydrogenase 8 family. member A1
0.79	CRYBA1	crystallin beta A1
0.79	FAM160A1	family with sequence similarity 160. member A1
0.79	NMI	N-myc (and STAT) interactor
0.79	MYCBPAP	MYCBP associated protein
0.79	SRP19; ZRSR1	signal recognition particle 19kDa; zinc finger (CCCH type). RNA binding motif and serine/arginine rich 1
0.79	RHBDD3	rhomboid domain containing 3
0.79	SCRIB	Memczak2013 ANTISENSE. CDS. coding. INTERNAL best transcript NM_015356
0.79	CCDC102B	coiled-coil domain containing 102B
0.79	EPG5	ectopic P-granules autophagy protein 5 homolog (C. elegans)
0.79	SURF1	surfeit 1
0.79	CHMP7	charged multivesicular body protein 7
0.79	MAN1C1	mannosidase. alpha. class 1C. member 1
0.78	HIST1H1A	histone cluster 1. H1a
0.78	GLYAT	glycine-N-acyltransferase
0.78	MEX3D	mex-3 RNA binding family member D
0.78	ACSM2A	acyl-CoA synthetase medium-chain family member 2A
0.78	TMEM170B	transmembrane protein 170B
0.78	WDR37	WD repeat domain 37

0.78	THAP7-AS1	THAP7 antisense RNA 1
0.78	SCN7A	sodium channel. voltage gated. type VII alpha subunit
0.78	ZNF8	zinc finger protein 8
0.78	MMP2	matrix metallopeptidase 2
0.78	KCNE1	potassium channel. voltage gated subfamily E regulatory beta subunit 1
0.78	SPTBN4	spectrin. beta. non-erythrocytic 4
0.78	MYO5C	myosin VC
0.78	SLAIN1	SLAIN motif family member 1
0.78	HIP1R	Memczak2013 ALT_ACCEPTOR. ALT_DONOR. coding. INTERNAL. intronic best transcript NM_003959
0.78	PRDX2	peroxiredoxin 2
0.78	CDKN2D	cyclin-dependent kinase inhibitor 2D (p19. inhibits CDK4)
0.78	MUC20; SDHAP2; MIR570; LINC00969	mucin 20. cell surface associated; succinate dehydrogenase complex subunit A. flavoprotein pseudogene 2; microRNA 570; long intergenic non-protein coding RNA 969
0.78	CBLN3	cerebellin 3 precursor
0.78	SCNN1B	sodium channel. non voltage gated 1 beta subunit
0.78	LYRM7	LYR motif containing 7
0.78	OR12D3	olfactory receptor. family 12. subfamily D. member 3
0.78	KCNQ5	Jeck2013 ALT_ACCEPTOR. ALT_DONOR. coding. INTERNAL. intronic best transcript NM_001160133
0.78	RBM43	RNA binding motif protein 43
0.78	ENC1	ectodermal-neural cortex 1 (with BTB domain)
0.78	UXT-AS1	UXT antisense RNA 1
0.78	TBC1D26	TBC1 domain family. member 26
0.78	NPM1	Zhang2013 ALT_ACCEPTOR. ALT_DONOR. coding. INTERNAL. intronic best transcript NM_002520
0.78	OTUD6A	OTU deubiquitinase 6A

0.78	MYO10	myosin X
0.78	PSKH1	protein serine kinase H1
0.78	SCN9A	sodium channel. voltage gated. type IX alpha subunit
0.78	ZNF438	zinc finger protein 438
0.78	LELP1	late cornified envelope-like proline-rich 1
0.78	TBC1D8	TBC1 domain family. member 8 (with GRAM domain)
0.78	PHACTR3	phosphatase and actin regulator 3
0.78	HIST1H2AH	histone cluster 1. H2ah
0.78	PTK2B	protein tyrosine kinase 2 beta
0.78	EDNRA	endothelin receptor type A
0.78	MRPS12	mitochondrial ribosomal protein S12
0.78	SLC39A7	solute carrier family 39 (zinc transporter). member 7
0.78	SFTPB	surfactant protein B
0.78	EMD	emerin
0.78	NASP	nuclear autoantigenic sperm protein (histone-binding)
0.78	PRM3	protamine 3
0.78	MAGIX	MAGI family member. X-linked
0.78	KBTBD13	kelch repeat and BTB (POZ) domain containing 13
0.78	UBTD2	ubiquitin domain containing 2
0.78	PKDREJ	polycystin (PKD) family receptor for egg jelly
0.78	ARID5B	AT rich interactive domain 5B (MRF1-like)
0.78	STKLD1	serine/threonine kinase-like domain containing 1 [Source:HGNC Symbol;Acc:HGNC:28669]
0.78	TPRG1	tumor protein p63 regulated 1
0.78	UBXN10	UBX domain protein 10
0.78	HSBP1	heat shock factor binding protein 1

0.78	SLC39A14	solute carrier family 39 (zinc transporter). member 14
0.78	CCDC146	coiled-coil domain containing 146
0.78	MAP3K3	mitogen-activated protein kinase kinase kinase 3
0.78	BDNF	brain-derived neurotrophic factor
0.78	GGN	gametogenetin
0.78	ASPDH	aspartate dehydrogenase domain containing
0.78	SOX5	Transcript Identified by AceView. Entrez Gene ID(s) 6660
0.78	HIST1H4B	histone cluster 1. H4b
0.78	DHRS4L2	dehydrogenase/reductase (SDR family) member 4 like 2
0.78	LMF2	lipase maturation factor 2
0.78	MLEC	malectin
0.78	OR4K13	olfactory receptor. family 4. subfamily K. member 13
0.78	PKN3	protein kinase N3
0.78	HIST1H3G	histone cluster 1. H3g
0.78	SIGMAR1	sigma non-opioid intracellular receptor 1
0.78	RAB11B	RAB11B. member RAS oncogene family
0.78	ZNF680	zinc finger protein 680
0.78	VMAC	vimentin-type intermediate filament associated coiled-coil protein
0.78	TAOK3	TAO kinase 3
0.78	TMF1	TATA element modulatory factor 1
0.78	RAD21-AS1	RAD21 antisense RNA 1
0.78	OST4	oligosaccharyltransferase complex subunit 4 (non-catalytic)
0.78	MYOF	myoferlin
0.78	GTF2H2C_2; GTF2H2C; GTF2H2B	GTF2H2 family member C. copy 2; GTF2H2 family member C; general transcription factor IIH subunit 2B (pseudogene)
0.78	SDF2L1	stromal cell-derived factor 2-like 1

0.78	SSH3	slingshot protein phosphatase 3
0.78	LCE2B	late cornified envelope 2B
0.78	AKR1E2	aldo-keto reductase family 1. member E2
0.78	RARRES2	retinoic acid receptor responder (tazarotene induced) 2
0.78	ZSWIM3	zinc finger. SWIM-type containing 3
0.78	ADH5	alcohol dehydrogenase 5 (class III). chi polypeptide
0.78	SURF1	surfeit 1
0.78	LRRN3	leucine rich repeat neuronal 3
0.78	KRT222	keratin 222. type II
0.78	MBOAT2	Memczak2013 ALT_ACCEPTOR. ALT_DONOR. coding. INTERNAL. intronic best transcript NM_138799
0.78	ZNF236	zinc finger protein 236
0.78	SELL	Memczak2013 ALT_ACCEPTOR. ALT_DONOR. coding. INTERNAL. intronic best transcript NM_000655
0.78	CASP8AP2	caspase 8 associated protein 2
0.78	BPIFC	BPI fold containing family C
0.78	SCRN3	secernin 3
0.78	BIRC6	Transcript Identified by AceView. Entrez Gene ID(s) 57448
0.78	HIST2H2AA3; HIST2H2AA4	histone cluster 2. H2aa3; histone cluster 2. H2aa4
0.78	SORBS2	sorbin and SH3 domain containing 2
0.78	TP53INP2	tumor protein p53 inducible nuclear protein 2
0.78	ND1	NADH dehydrogenase. subunit 1 (complex I)
0.78	PARD6A	par-6 family cell polarity regulator alpha
0.78	ZRANB3	zinc finger. RAN-binding domain containing 3
0.78	ZFHX3	zinc finger homeobox 3
0.78	DYRK1B	dual specificity tyrosine-(Y)-phosphorylation regulated kinase 1B
0.78	ARFIP2	ADP-ribosylation factor interacting protein 2

0.78	NLRP9	NLR family. pyrin domain containing 9
0.78	ACACA	acetyl-CoA carboxylase alpha
0.78	VPS37C	vacuolar protein sorting 37 homolog C (S. cerevisiae)
0.78	TMEM120A	transmembrane protein 120A
0.78	SLC26A6; MIR6824	solute carrier family 26 (anion exchanger). member 6; microRNA 6824
0.78	SYCN	syncollin
0.78	MROH8	maestro heat-like repeat family member 8
0.78	PDE7A	phosphodiesterase 7A
0.78	MFGE8	milk fat globule-EGF factor 8 protein
0.78	ZBTB9	zinc finger and BTB domain containing 9
0.78	PTPN5	protein tyrosine phosphatase. non-receptor type 5 (striatum-enriched)
0.78	CEP126	centrosomal protein 126kDa
0.78	TRPT1	tRNA phosphotransferase 1
0.78	IDH3G	isocitrate dehydrogenase 3 (NAD+) gamma
0.78	TMUB2	transmembrane and ubiquitin-like domain containing 2
0.78	STARD3	StAR-related lipid transfer domain containing 3
0.78	ZNHIT1	zinc finger. HIT-type containing 1
0.78	KHK	ketohexokinase
0.78	FAHD2A	Transcript Identified by AceView. Entrez Gene ID(s) 51011
0.78	PDIA4	protein disulfide isomerase family A. member 4
0.78	NIF3L1	NIF3 NGG1 interacting factor 3-like 1
0.78	TTC21B	tetratricopeptide repeat domain 21B
0.78	CBFA2T2	core-binding factor. runt domain. alpha subunit 2; translocated to. 2
0.78	ZNF528	zinc finger protein 528
0.78	ZNF324B	zinc finger protein 324B
0.78	CHKB	choline kinase beta

0.78	CNTD2	cyclin N-terminal domain containing 2
0.78	EPHB2	EPH receptor B2
0.78	VSNL1	visinin like 1
0.78	ICAM2	intercellular adhesion molecule 2
0.78	P4HB	prolyl 4-hydroxylase. beta polypeptide
0.78	ZNF526	zinc finger protein 526
0.78	SPNS1	spinster homolog 1 (Drosophila)
0.78	FNBP1L	Transcript Identified by AceView. Entrez Gene ID(s) 54874
0.78	SH3RF2	SH3 domain containing ring finger 2
0.78	UMODL1-AS1	UMODL1 antisense RNA 1
0.78	NME3	NME/NM23 nucleoside diphosphate kinase 3
0.78	NKAIN4	Na+/K+ transporting ATPase interacting 4
0.78	TTC32	tetratricopeptide repeat domain 32
0.78	GJC2	gap junction protein gamma 2
0.78	GPX3	glutathione peroxidase 3
0.78	ZC3H10	zinc finger CCCH-type containing 10
0.78	FAM222A	family with sequence similarity 222. member A
0.78	GPS2	G protein pathway suppressor 2
0.78	DNM1	dynamin 1
0.78	TRAF3IP3	TRAF3 interacting protein 3
0.78	RBM14	RNA binding motif protein 14
0.78	CD40	CD40 molecule. TNF receptor superfamily member 5
0.78	STC1	stanniocalcin 1
0.78	OR6P1	olfactory receptor. family 6. subfamily P. member 1
0.78	FZD1	frizzled class receptor 1
0.78	PROKR2	prokineticin receptor 2

0.78	SYNE2	spectrin repeat containing. nuclear envelope 2
0.78	ALG10	ALG10. alpha-1.2-glucosyltransferase
0.78	MTRF1	mitochondrial translational release factor 1
0.78	CYP2D6	cytochrome P450. family 2. subfamily D. polypeptide 6
0.78	MGAT4EP	MGAT4 family. member E. pseudogene
0.78	NISCH	nischarin
0.78	SIN3A	SIN3 transcription regulator family member A
0.78	TBC1D3	TBC1 domain family. member 3
0.78	OPRK1	opioid receptor. kappa 1
0.78	OR10J3	olfactory receptor. family 10. subfamily J. member 3
0.78	TMEM82	transmembrane protein 82
0.78	PCTP	phosphatidylcholine transfer protein
0.78	OR11G2	olfactory receptor. family 11. subfamily G. member 2
0.78	TRAPPC2L	trafficking protein particle complex 2-like
0.78	ZDHHC12	zinc finger. DHHC-type containing 12
0.78	PELI3	pellino E3 ubiquitin protein ligase family member 3
0.78	GBA3	glucosidase. beta. acid 3 (gene/pseudogene)
0.78	EPO	erythropoietin
0.78	IGFBPL1	insulin like growth factor binding protein-like 1
0.78	SRGAP2B	SLIT-ROBO Rho GTPase activating protein 2B
0.78	PSMG4	Transcript Identified by AceView. Entrez Gene ID(s) 389362
0.78	CDC37L1	cell division cycle 37-like 1
0.78	FAM76A	family with sequence similarity 76. member A
0.78	APOC2	apolipoprotein C-II
0.78	PRKCA	protein kinase C. alpha
0.78	SH2B3	Transcript Identified by AceView. Entrez Gene ID(s) 10019

0.78	C4orf3	chromosome 4 open reading frame 3
0.78	GPATCH2	G-patch domain containing 2
0.78	RECQL4	RecQ helicase-like 4
0.78	GPHA2	glycoprotein hormone alpha 2
0.78	TFF3	trefoil factor 3
0.78	SCLT1	sodium channel and clathrin linker 1
0.78	FOXRED1	FAD-dependent oxidoreductase domain containing 1
0.78	ACADSB	acyl-CoA dehydrogenase. short/branched chain
0.78	VIL1	villin 1
0.78	ZNF660	zinc finger protein 660
0.78	NCDN	neurochondrin
0.78	LOC149950; RP11-410N8.4	uncharacterized LOC149950; novel transcript
0.78	ARMCX2	armadillo repeat containing. X-linked 2
0.78	SRRT	serrate. RNA effector molecule
0.78	HCN3	hyperpolarization activated cyclic nucleotide gated potassium channel 3
0.78	KIAA0125	KIAA0125
0.78	C5orf51	chromosome 5 open reading frame 51
0.78	CSHL1	chorionic somatomammotropin hormone-like 1
0.78	HRASLS	HRAS-like suppressor
0.78	MRPL52	mitochondrial ribosomal protein L52
0.78	UVSSA	UV stimulated scaffold protein A
0.78	KLHL15	kelch-like family member 15
0.78	ZKSCAN7	zinc finger with KRAB and SCAN domains 7
0.78	SMLR1	small leucine-rich protein 1
0.78	ELF5	E74-like factor 5 (ets domain transcription factor)
0.78	XKRX	X-linked Kx blood group related. X-linked

0.78	PDPK1	3-phosphoinositide dependent protein kinase 1
0.78	FN3K	fructosamine 3 kinase
0.78	ANKRD6	ankyrin repeat domain 6
0.78	BANF1	barrier to autointegration factor 1
0.78	ZNF511	zinc finger protein 511
0.78	BORA	bora. aurora kinase A activator
0.78	POTEA	POTE ankyrin domain family. member A
0.78	MRPL24	mitochondrial ribosomal protein L24
0.77	HOXA1	homeobox A1
0.77	FAM159A	family with sequence similarity 159. member A
0.77	KRTAP5-7	keratin associated protein 5-7
0.77	TMEM178B	transmembrane protein 178B
0.77	GNG4	guanine nucleotide binding protein (G protein). gamma 4
0.77	ERN2	endoplasmic reticulum to nucleus signaling 2
0.77	PIK3R1	phosphoinositide-3-kinase. regulatory subunit 1 (alpha)
0.77	ZNF813	zinc finger protein 813
0.77	PRAMEF1	PRAME family member 1
0.77	GJB6	gap junction protein beta 6
0.77	RSPO4	R-spondin 4
0.77	PXMP2	peroxisomal membrane protein 2
0.77	POLR2E	polymerase (RNA) II (DNA directed) polypeptide E. 25kDa
0.77	HSPA12A	heat shock 70kDa protein 12A
0.77	CENPC	centromere protein C
0.77	GDA	guanine deaminase
0.77	DRGX	dorsal root ganglia homeobox
0.77	WBP2	WW domain binding protein 2

0.77	PHRF1	PHD and ring finger domains 1
0.77	AGPAT4-IT1	AGPAT4 intronic transcript 1
0.77	CBX2	chromobox homolog 2
0.77	WFS1	Wolfram syndrome 1 (wolframin)
0.77	CCDC84	coiled-coil domain containing 84
0.77	FCER1A	Fc fragment of IgE. high affinity I. receptor for; alpha polypeptide
0.77	QRICH1	Transcript Identified by AceView. Entrez Gene ID(s) 54870
0.77	GLMP	glycosylated lysosomal membrane protein
0.77	NT5DC2	5-nucleotidase domain containing 2
0.77	MED11	mediator complex subunit 11
0.77	ENPEP	glutamyl aminopeptidase (aminopeptidase A)
0.77	RBPJL	recombination signal binding protein for immunoglobulin kappa J region-like
0.77	VDAC3	voltage-dependent anion channel 3
0.77	OCRL	Transcript Identified by AceView. Entrez Gene ID(s) 4952
0.77	HDHD3	haloacid dehalogenase-like hydrolase domain containing 3
0.77	COCH	cochlin
0.77	F7	coagulation factor VII (serum prothrombin conversion accelerator)
0.77	HILPDA	hypoxia inducible lipid droplet-associated
0.77	VSTM2A; VSTM2A-OT1	V-set and transmembrane domain containing 2A; VSTM2A overlapping transcript 1
0.77	CNOT11	CCR4-NOT transcription complex subunit 11
0.77	WDFY2	WD repeat and FYVE domain containing 2
0.77	C2CD4A	C2 calcium-dependent domain containing 4A
0.77	EPS15L1	epidermal growth factor receptor pathway substrate 15-like 1
0.77	SLC28A2	solute carrier family 28 (concentrative nucleoside transporter). member 2
0.77	STUB1	STIP1 homology and U-box containing protein 1. E3 ubiquitin protein ligase

0.77	FAM126A	family with sequence similarity 126. member A
0.77	ST5	Transcript Identified by AceView. Entrez Gene ID(s) 6764
0.77	TBC1D3C	TBC1 domain family. member 3C
0.77	STX17	syntaxin 17
0.77	SOD2	superoxide dismutase 2. mitochondrial
0.77	ADAMTS18	ADAM metallopeptidase with thrombospondin type 1 motif 18
0.77	MSI2	Memczak2013 ALT_ACCEPTOR. ALT_DONOR. coding. INTERNAL. intronic best transcript NM_170721
0.77	PRR26	proline rich 26
0.77	SFXN3	sideroflexin 3
0.77	POU2F3	POU class 2 homeobox 3
0.77	ACTR1B	ARP1 actin-related protein 1 homolog B. centractin beta (yeast)
0.77	KCNH6	potassium channel. voltage gated eag related subfamily H. member 6
0.77	DOC2A	double C2-like domains. alpha
0.77	RABL2A	RAB. member of RAS oncogene family-like 2A
0.77	LRRN4CL	LRRN4 C-terminal like
0.77	CREM	cAMP responsive element modulator
0.77	MNX1	motor neuron and pancreas homeobox 1
0.77	ASPRV1; PCBP1-AS1	aspartic peptidase. retroviral-like 1; PCBP1 antisense RNA 1
0.77	HSPBP1	HSPA (heat shock 70kDa) binding protein. cytoplasmic cochaperone 1
0.77	IFI6	interferon. alpha-inducible protein 6
0.77	SRSF2; MIR636	serine/arginine-rich splicing factor 2; microRNA 636
0.77	SYNGR2	synaptogyrin 2
0.77	AC208162.1	
0.77	HOXD1; MIR7704	homeobox D1; microRNA 7704
0.77	OXCT2	3-oxoacid CoA-transferase 2

0.77	CENPW	centromere protein W
0.77	ALDH3B1	aldehyde dehydrogenase 3 family. member B1
0.77	SWT1	SWT1 RNA endoribonuclease homolog
0.77	GPA33	glycoprotein A33 (transmembrane)
0.77	ABHD14A-ACY1	ABHD14A-ACY1 readthrough
0.77	IL22RA1	interleukin 22 receptor. alpha 1
0.77	PAQR8	progestin and adipoQ receptor family member VIII
0.77	EXOC8	exocyst complex component 8
0.77	SIRT6	sirtuin 6
0.77	TMPRSS6	transmembrane protease. serine 6
0.77	USP43	ubiquitin specific peptidase 43
0.77	C20orf196	chromosome 20 open reading frame 196
0.77	ERAP2	endoplasmic reticulum aminopeptidase 2
0.77	MINOS1-NBL1	MINOS1-NBL1 readthrough
0.77	ASPHD1	aspartate beta-hydroxylase domain containing 1
0.77	MYO1F	Memczak2013 ANTISENSE. CDS. coding. INTERNAL best transcript NM_012335
0.77	GATSL3	GATS protein-like 3
0.77	TICRR	TOPBP1-interacting checkpoint and replication regulator
0.77	PCOLCE	procollagen C-endopeptidase enhancer
0.77	OR1A1	olfactory receptor. family 1. subfamily A. member 1
0.77	MAGEE2	MAGE family member E2
0.77	SLC30A4	solute carrier family 30 (zinc transporter). member 4
0.77	WFDC12	WAP four-disulfide core domain 12
0.77	BUD31	Transcript Identified by AceView. Entrez Gene ID(s) 8896
0.77	SMYD3	SET and MYND domain containing 3

0.77	DEFB118	defensin. beta 118
0.77	LGALS2	lectin. galactoside-binding. soluble. 2
0.77	RNF26	ring finger protein 26
0.77	SLC22A2	solute carrier family 22 (organic cation transporter). member 2
0.77	TMEM178A	transmembrane protein 178A
0.77	UBE2T	ubiquitin conjugating enzyme E2T
0.77	AGAP9; CTGLF11P	Salzman2013 ANNOTATED. CDS. coding. INTERNAL. OVCODE. OVEXON best transcript NM_001190810; centaurin. gamma-like family. member 11 pseudogene [Source:HGNC Symbol;Acc:HGNC:23660]
0.77	TSPAN33	tetraspanin 33
0.77	TMEM110-MUSTN1; TMEM110	TMEM110-MUSTN1 readthrough; transmembrane protein 110
0.77	FAM151B	family with sequence similarity 151. member B
0.77	TNR	tenascin R
0.77	C9orf50	chromosome 9 open reading frame 50
0.77	PAQR9	progestin and adipoQ receptor family member IX
0.77	TTC12	tetratricopeptide repeat domain 12
0.77	SIX5	SIX homeobox 5
0.77	HEXIM1	hexamethylene bis-acetamide inducible 1
0.77	RHPN1	rhophilin. Rho GTPase binding protein 1
0.77	CCDC170	coiled-coil domain containing 170
0.77	PLBD2	phospholipase B domain containing 2
0.77	RBM42	RNA binding motif protein 42
0.77	CLEC18B	C-type lectin domain family 18. member B
0.77	ZNF737	zinc finger protein 737
0.77	ACLY	ATP citrate lyase
0.77	MTFR1L	mitochondrial fission regulator 1-like
0.77	UQCR10	ubiquinol-cytochrome c reductase. complex III subunit X

0.77	KRTAP4-2	keratin associated protein 4-2
0.77	CLIC2	chloride intracellular channel 2
0.77	ZNF833P	zinc finger protein 833. pseudogene
0.77	FHAD1	forkhead-associated (FHA) phosphopeptide binding domain 1
0.77	SCYL3	SCY1-like. kinase-like 3
0.77	LRP11	LDL receptor related protein 11
0.77	SLC13A2	solute carrier family 13 (sodium-dependent dicarboxylate transporter). member 2
0.77	PRR36	proline rich 36
0.77	PRAMEF8	PRAME family member 8
0.77	SGK1	serum/glucocorticoid regulated kinase 1
0.77	ITSN1	Transcript Identified by AceView. Entrez Gene ID(s) 6453
0.77	SELE	selectin E
0.77	RASGRP2	RAS guanyl releasing protein 2 (calcium and DAG-regulated)
0.77	BUB1B	BUB1 mitotic checkpoint serine/threonine kinase B
0.77	UPF1	UPF1 regulator of nonsense transcripts homolog (yeast)
0.77	SCAMP3	secretory carrier membrane protein 3
0.77	HIGD2B	HIG1 hypoxia inducible domain family. member 2B
0.77	CELF5	CUGBP. Elav-like family member 5
0.77	GFOD2	glucose-fructose oxidoreductase domain containing 2
0.77	AKR7A3	aldo-keto reductase family 7. member A3 (aflatoxin aldehyde reductase)
0.77	MAPRE2	microtubule-associated protein. RP/EB family. member 2
0.77	GPBAR1	G protein-coupled bile acid receptor 1
0.77	NPFFR1	neuropeptide FF receptor 1
0.77	SCN8A	sodium channel. voltage gated. type VIII alpha subunit
0.77	ACAD11	acyl-CoA dehydrogenase family. member 11

0.77	TCAF1	TRPM8 channel-associated factor 1
0.77	PDXDC1	pyridoxal-dependent decarboxylase domain containing 1
0.77	ASB8	ankyrin repeat and SOCS box containing 8
0.77	ZNF624	zinc finger protein 624
0.77	RELN	reelin
0.77	ZNF705D	zinc finger protein 705D
0.77	CCDC150	coiled-coil domain containing 150
0.77	ZNF25	zinc finger protein 25
0.77	KIF18B	kinesin family member 18B
0.77	SUSD5	sushi domain containing 5
0.77	USP9X	Memczak2013 ANTISENSE. CDS. coding. INTERNAL best transcript NM_001039590
0.77	FAM163B	family with sequence similarity 163. member B
0.77	PET100	PET100 homolog
0.77	DCDC5; DCDC1	doublecortin domain containing 5; doublecortin domain containing 1
0.77	CCDC33	coiled-coil domain containing 33
0.77	OR4F15	olfactory receptor. family 4. subfamily F. member 15
0.77	SPATA2	spermatogenesis associated 2
0.77	ZNF716	zinc finger protein 716
0.77	CCT6B	chaperonin containing TCP1. subunit 6B (zeta 2)
0.77	CRB3	crumbs family member 3
0.77	SURF1	surfeit 1
0.77	RHBG	Rh family. B glycoprotein (gene/pseudogene)
0.77	CHI3L2	chitinase 3-like 2
0.77	FOXD4L5	forkhead box D4-like 5
0.77	TMEM212	transmembrane protein 212

0.77	RFC4	replication factor C subunit 4
0.77	ART4	ADP-ribosyltransferase 4 (Dombrock blood group)
0.77	HELZ2	helicase with zinc finger 2. transcriptional coactivator
0.77	MTERF4	mitochondrial transcription termination factor 4
0.77	SRSF6	serine/arginine-rich splicing factor 6
0.77	IQCD	IQ motif containing D
0.77	ROMO1	reactive oxygen species modulator 1
0.77	CDK2	cyclin-dependent kinase 2
0.77	MTRNR2L13	MT-RNR2-like 13
0.77	SHPK; TRPV1	sedoheptulokinase; transient receptor potential cation channel. subfamily V. member 1
0.77	MRGPRD	MAS-related GPR. member D
0.77	GPATCH3	G-patch domain containing 3
0.77	SLC44A2	solute carrier family 44 (choline transporter). member 2
0.77	PIGV	phosphatidylinositol glycan anchor biosynthesis class V
0.77	CLIC4	chloride intracellular channel 4
0.77	RPAP1	RNA polymerase II associated protein 1
0.77	CLIP3	CAP-GLY domain containing linker protein 3
0.77	MICB	MHC class I polypeptide-related sequence B
0.76	GIGYF1	GRB10 interacting GYF protein 1
0.76	PCGF5	polycomb group ring finger 5
0.76	NUP210	nucleoporin 210kDa
0.76	PEBP1	Memczak2013 ANTISENSE. coding. INTERNAL. UTR3 best transcript NM_002567
0.76	FAM219A	family with sequence similarity 219. member A
0.76	TCEA3	transcription elongation factor A (SII). 3
0.76	ARHGAP44	Rho GTPase activating protein 44

0.76	FAM187B	family with sequence similarity 187. member B
0.76	CCDC34	coiled-coil domain containing 34
0.76	ART1	ADP-ribosyltransferase 1
0.76	CD8A	CD8a molecule
0.76	NDRG3	NDRG family member 3
0.76	PSMC3IP	PSMC3 interacting protein
0.76	ACOT13	acyl-CoA thioesterase 13
0.76	CDC42EP5	CDC42 effector protein (Rho GTPase binding) 5
0.76	YPEL3	yippee like 3
0.76	SYNE4	spectrin repeat containing. nuclear envelope family member 4
0.76	ITGB1BP2	integrin beta 1 binding protein (melusin) 2
0.76	HMGN1	high mobility group nucleosome binding domain 1
0.76	CLSTN3	calsyntenin 3
0.76	MYL6	Memczak2013 ANTISENSE. coding. INTERNAL. intronic best transcript NM_079423
0.76	MAGEA2; MAGEA2B	MAGE family member A2; MAGE family member A2B
0.76	TMEM99	transmembrane protein 99
0.76	OR2B6	olfactory receptor. family 2. subfamily B. member 6
0.76	CCDC92	coiled-coil domain containing 92
0.76	CHMP7	Transcript Identified by AceView. Entrez Gene ID(s) 91782
0.76	POLR2J	polymerase (RNA) II (DNA directed) polypeptide J. 13.3kDa
0.76	CENPN	centromere protein N
0.76	BIRC5	baculoviral IAP repeat containing 5
0.76	PTGS1	prostaglandin-endoperoxide synthase 1 (prostaglandin G/H synthase and cyclooxygenase)
0.76	KANSL1L	KAT8 regulatory NSL complex subunit 1 like
0.76	RAB3A	RAB3A. member RAS oncogene family

0.76	C6orf120	chromosome 6 open reading frame 120
0.76	PGP	phosphoglycolate phosphatase
0.76	NR2E1	nuclear receptor subfamily 2. group E. member 1
0.76	PRDM10	PR domain containing 10
0.76	LYSMD4	LysM. putative peptidoglycan-binding. domain containing 4
0.76	VPS53; RPS4XP17	vacuolar protein sorting 53 homolog (S. cerevisiae); ribosomal protein S4X pseudogene 17
0.76	ANKRD7	ankyrin repeat domain 7
0.76	HAGH	hydroxyacylglutathione hydrolase
0.76	NUP50	nucleoporin 50kDa
0.76	KRTAP15-1	keratin associated protein 15-1
0.76	CDH7	cadherin 7. type 2
0.76	C1orf198	chromosome 1 open reading frame 198
0.76	GPIHBP1	glycosylphosphatidylinositol anchored high density lipoprotein binding protein 1
0.76	FAM227A	family with sequence similarity 227. member A
0.76	SNAPC4	Zhang2013 ALT_ACCEPTOR. ALT_DONOR. coding. INTERNAL. intronic best transcript NM_003086
0.76	BIN1	bridging integrator 1
0.76	ZBTB12	zinc finger and BTB domain containing 12
0.76	CD300LG	CD300 molecule-like family member g
0.76	PAQR6	progestin and adipoQ receptor family member VI
0.76	ABCC10	ATP binding cassette subfamily C member 10
0.76	XRCC3	X-ray repair complementing defective repair in Chinese hamster cells 3
0.76	AGBL3	ATP/GTP binding protein-like 3
0.76	LIN54	lin-54 DREAM MuvB core complex component
0.76	C5orf66-AS1	C5orf66 antisense RNA 1

0.76	IQCF6	IQ motif containing F6
0.76	DPF3	D4. zinc and double PHD fingers. family 3
0.76	ARHGAP11B; ARHGAP11A	Rho GTPase activating protein 11B; Rho GTPase activating protein 11A
0.76	ZC3H7A	zinc finger CCCH-type containing 7A
0.76	SLC9A8	solute carrier family 9. subfamily A (NHE8. cation proton antiporter 8). member 8
0.76	SMARCD3	SWI/SNF related. matrix associated. actin dependent regulator of chromatin. subfamily d. member 3
0.76	PALB2	partner and localizer of BRCA2
0.76	PUDP	pseudouridine 5-phosphatase
0.76	PRSS37	protease. serine. 37
0.76	LINC01126	long intergenic non-protein coding RNA 1126
0.76	ATP13A3	Jeck2013 ALT_ACCEPTOR. ALT_DONOR. coding. INTERNAL. intronic best transcript NM_024524
0.76	TTC34	tetratricopeptide repeat domain 34
0.76	RNPEP	arginyl aminopeptidase (aminopeptidase B)
0.76	CENPJ	centromere protein J
0.76	KPNA2	karyopherin alpha 2 (RAG cohort 1. importin alpha 1)
0.76	CEP19	centrosomal protein 19kDa
0.76	BCAM	basal cell adhesion molecule (Lutheran blood group)
0.76	SFR1	SWI5-dependent homologous recombination repair protein 1
0.76	GTF3A	general transcription factor IIIA
0.76	RP11-35G9.5; ATP8B1	Salzman2013 ANTISENSE. coding. INTERNAL. intronic. OVERLAPTX best transcript NM_005603; novel transcript. antisense to ATP8B1
0.76	DCK	deoxycytidine kinase
0.76	SNRPN; SNURF; SNORD107; PWARSN	small nuclear ribonucleoprotein polypeptide N; SNRPN upstream reading frame; small nucleolar RNA. C/D box 107; Prader Willi/Angelman region RNA. SNRPN neighbor

0.76	NPC2; MIR4709	Niemann-Pick disease. type C2; microRNA 4709
0.76	CCL16	chemokine (C-C motif) ligand 16
0.76	ADAMTS15	ADAM metallopeptidase with thrombospondin type 1 motif 15
0.76	FBXO33	F-box protein 33
0.76	ABCB8	ATP binding cassette subfamily B member 8
0.76	STK11IP	serine/threonine kinase 11 interacting protein
0.76	TRIOBP; NOL12	TRIO and F-actin binding protein; nucleolar protein 12
0.76	ZBED8	zinc finger. BED-type containing 8
0.76	SEMA4G	sema domain. immunoglobulin domain (Ig). transmembrane domain (TM) and short cytoplasmic domain. (semaphorin) 4G
0.76	CEP89	centrosomal protein 89kDa
0.76	PRDM6	PR domain containing 6
0.76	LSM14B	LSM family member 14B
0.76	GLE1	GLE1 RNA export mediator
0.76	NREP	neuronal regeneration related protein
0.76	ZNF784	zinc finger protein 784
0.76	CDC42EP4	CDC42 effector protein (Rho GTPase binding) 4
0.76	UCP3	uncoupling protein 3 (mitochondrial. proton carrier)
0.76	DHRS12	dehydrogenase/reductase (SDR family) member 12
0.76	LDOC1L	leucine zipper. down-regulated in cancer 1-like
0.76	GDPD1	glycerophosphodiester phosphodiesterase domain containing 1
0.76	PDP2	pyruvate dehyrogenase phosphatase catalytic subunit 2
0.76	FIS1	fission. mitochondrial 1
0.76	TRADD	TNFRSF1A-associated via death domain
0.76	CGNL1	cingulin-like 1
0.76	SCD	stearoyl-CoA desaturase (delta-9-desaturase)

0.76	HSD17B1	hydroxysteroid (17-beta) dehydrogenase 1
0.76	NIPSNAP1	nipsnap homolog 1 (C. elegans)
0.76	SPRY3	sprouty RTK signaling antagonist 3
0.76	SPRY3	Homo sapiens sprouty RTK signaling antagonist 3 (SPRY3). transcript variant 1. mRNA.; sprouty homolog 3 (Drosophila) [Source:HGNC Symbol;Acc:HGNC:11271]; Homo sapiens sprouty homolog 3 (Drosophila). mRNA (cDNA clone MGC:97072 IMAGE:7262284). complete cds.; Homo sapiens sprouty homolog 3 (Drosophila). mRNA (cDNA clone MGC:103904 IMAGE:30915293). complete cds.; Homo sapiens sprouty homolog 3 (Drosophila). mRNA (cDNA clone MGC:104063 IMAGE:30915511). complete cds.
0.76	LOC155060; ZNF783	AI894139 pseudogene; Transcript Identified by AceView. Entrez Gene ID(s) 155060
0.76	KLK15	kallikrein related peptidase 15
0.76	SELO	selenoprotein O; selenoprotein O [Source:EntrezGene;Acc:83642]; Salzman2013 ANNOTATED. CDS. coding. INTERNAL. OVCODE. OVERLAPTX. OVEXON best transcript NM_031454; Salzman2013 ANNOTATED. CDS. coding. OVCODE. OVERLAPTX. OVEXON. UTR3 best transcript NM_031454; Transcript Identified by AceView. Entrez Gene ID(s) 83642. RefSeq ID(s) NM_031454
0.76	P3H1	prolyl 3-hydroxylase 1
0.76	KIF14	kinesin family member 14
0.76	HCAR1	hydroxycarboxylic acid receptor 1
0.76	NHSL2	NHS-like 2
0.76	GPR137	G protein-coupled receptor 137
0.76	DRC1	dynein regulatory complex subunit 1
0.76	GLO1	glyoxalase I
0.76	NMRK1	nicotinamide riboside kinase 1
0.76	CD44	CD44 molecule (Indian blood group)
0.76	LINC00654	long intergenic non-protein coding RNA 654

0.76	SMO	smoothened. frizzled class receptor
0.76	IFT140	intraflagellar transport 140
0.76	ABCC6	ATP binding cassette subfamily C member 6
0.76	ZNF488	zinc finger protein 488
0.76	CALM3	calmodulin 3 (phosphorylase kinase. delta)
0.76	ZNF789	zinc finger protein 789
0.76	ZNF428	zinc finger protein 428
0.76	HIST2H4A; HIST2H4B	histone cluster 2. H4a; histone cluster 2. H4b
0.76	ZNF660	zinc finger protein 660
0.76	FOXO4	forkhead box O4
0.76	MYL6B	myosin light chain 6B
0.76	ZNF100	zinc finger protein 100
0.76	PATE2	prostate and testis expressed 2
0.76	PRPF31	pre-mRNA processing factor 31
0.76	MRPL43	mitochondrial ribosomal protein L43
0.76	CXorf67	chromosome X open reading frame 67
0.76	ACTA1	actin. alpha 1. skeletal muscle
0.76	MSTN	myostatin
0.76	PHLDB2; PLCXD2	pleckstrin homology-like domain. family B. member 2; phosphatidylinositol-specific phospholipase C. X domain containing 2
0.76	ACAA1	acetyl-CoA acyltransferase 1
0.76	POMT1	protein-O-mannosyltransferase 1
0.76	RECQL5	RecQ helicase-like 5
0.76	PDK3	pyruvate dehydrogenase kinase. isozyme 3
0.76	EFCAB2	EF-hand calcium binding domain 2
0.76	HIST3H2BB	histone cluster 3. H2bb

0.76	DGUOK	deoxyguanosine kinase
0.76	PAXIP1	PAX interacting (with transcription-activation domain) protein 1
0.76	SYNE1	spectrin repeat containing. nuclear envelope 1
0.76	ANKRD9	ankyrin repeat domain 9
0.76	CHTOP	chromatin target of PRMT1
0.76	ST14	suppression of tumorigenicity 14 (colon carcinoma)
0.76	DSCC1	DNA replication and sister chromatid cohesion 1
0.76	CDKN1C	cyclin-dependent kinase inhibitor 1C (p57. Kip2)
0.76	CFC1B; CFC1	cripto. FRL-1. cryptic family 1B; cripto. FRL-1. cryptic family 1
0.76	CYP2E1	cytochrome P450. family 2. subfamily E. polypeptide 1
0.76	EDC4	enhancer of mRNA decapping 4
0.76	TRIM37	tripartite motif containing 37
0.76	KRT72	keratin 72. type II
0.76	ZFP36L2	ZFP36 ring finger protein-like 2
0.76	KCND2	potassium channel. voltage gated Shal related subfamily D. member 2
0.76	F10	coagulation factor X
0.76	LMX1A	LIM homeobox transcription factor 1. alpha
0.76	YIF1A	Yip1 interacting factor homolog A (S. cerevisiae)
0.76	RFC2	replication factor C subunit 2
0.76	LRRC17	leucine rich repeat containing 17
0.76	NFKBIB	nuclear factor of kappa light polypeptide gene enhancer in B-cells inhibitor. beta
0.76	CYP4V2	cytochrome P450. family 4. subfamily V. polypeptide 2
0.76	GNAL	guanine nucleotide binding protein (G protein). alpha activating activity polypeptide. olfactory type
0.76	HIST1H1C	histone cluster 1. H1c
0.76	ELOVL2	ELOVL fatty acid elongase 2

0.76	ZNF454	zinc finger protein 454
0.76	PTK2	protein tyrosine kinase 2
0.76	PKP2	plakophilin 2
0.76	CLDN1	claudin 1
0.76	ZNF436	zinc finger protein 436
0.76	CDK2AP1	cyclin-dependent kinase 2 associated protein 1
0.76	SASS6	SAS-6 centriolar assembly protein
0.76	GOLGA1	golgin A1
0.76	DKK4	dickkopf WNT signaling pathway inhibitor 4
0.76	OR10K2	olfactory receptor. family 10. subfamily K. member 2
0.76	FAM69B	family with sequence similarity 69. member B
0.76	BCL2L15	BCL2-like 15
0.76	LPXN	leupaxin
0.76	VPS52	vacuolar protein sorting 52 homolog (S. cerevisiae)
0.76	TTC7A	tetratricopeptide repeat domain 7A
0.76	RASSF2	Ras association (RalGDS/AF-6) domain family member 2
0.76	SMC2	structural maintenance of chromosomes 2
0.76	PMEL	premelanosome protein
0.76	FOXJ1	forkhead box J1
0.76	HSPG2	Jeck2013 ANTISENSE. CDS. coding. INTERNAL. intronic. OVCODE. OVEXON best transcript NM_005529
0.75	THBS1	thrombospondin 1
0.75	S1PR5	sphingosine-1-phosphate receptor 5
0.75	DKK1	dickkopf WNT signaling pathway inhibitor 1
0.75	LLGL1	lethal giant larvae homolog 1 (Drosophila)
0.75	KRTAP10-2	keratin associated protein 10-2

0.75	CNGA4	cyclic nucleotide gated channel alpha 4
0.75	ZNF660	zinc finger protein 660
0.75	SOS1	SOS Ras/Rac guanine nucleotide exchange factor 1
0.75	NCR1	natural cytotoxicity triggering receptor 1
0.75	MTRNR2L7	MT-RNR2-like 7
0.75	SORT1	sortilin 1
0.75	COLGALT2	collagen beta(1-O)galactosyltransferase 2
0.75	LRRN4	leucine rich repeat neuronal 4
0.75	FCRL6	Fc receptor-like 6
0.75	MMP24	matrix metallopeptidase 24 (membrane-inserted)
0.75	SPAG5	sperm associated antigen 5
0.75	NKTR	natural killer cell triggering receptor
0.75	SLC5A2	solute carrier family 5 (sodium/glucose cotransporter). member 2
0.75	CTSK	cathepsin K
0.75	ZNF862	zinc finger protein 862
0.75	DGKD	diacylglycerol kinase. delta 130kDa
0.75	WSCD2	WSC domain containing 2
0.75	TECPR1	tectonin beta-propeller repeat containing 1
0.75	C7orf13	chromosome 7 open reading frame 13
0.75	ZNF98	zinc finger protein 98
0.75	S100A1	S100 calcium binding protein A1
0.75	TMEM57	transmembrane protein 57
0.75	ZNF85	zinc finger protein 85
0.75	AMPD2	adenosine monophosphate deaminase 2
0.75	ZNF675	zinc finger protein 675
0.75	RSRC2	arginine/serine-rich coiled-coil 2

0.75	HSPB2; C11orf52; HSPB2-C11orf52	heat shock 27kDa protein 2; chromosome 11 open reading frame 52; HSPB2-C11orf52 readthrough (NMD candidate)
0.75	ARID4A	AT rich interactive domain 4A (RBP1-like)
0.75	ZIK1	zinc finger protein interacting with K protein 1
0.75	POMGNT2	protein O-linked mannose N-acetylglucosaminyltransferase 2 (beta 1.4-)
0.75	TCN2	transcobalamin II
0.75	KIAA0101; CSNK1G1	KIAA0101; casein kinase 1. gamma 1
0.75	ADAR	adenosine deaminase. RNA-specific
0.75	RIPPLY1	ripply transcriptional repressor 1
0.75	LYN	LYN proto-oncogene. Src family tyrosine kinase
0.75	IFT122	intraflagellar transport 122
0.75	CALCA	calcitonin-related polypeptide alpha
0.75	MYOC	myocilin. trabecular meshwork inducible glucocorticoid response
0.75	RNF207	ring finger protein 207
0.75	ASMTL	acetylserotonin O-methyltransferase-like
0.75	ASMTL	acetylserotonin O-methyltransferase-like
0.75	OR52M1	olfactory receptor. family 52. subfamily M. member 1
0.75	TMEM208	transmembrane protein 208
0.75	SLCO2B1	solute carrier organic anion transporter family. member 2B1
0.75	TCEAL8	transcription elongation factor A (SII)-like 8
0.75	NUDT4P1; NUDT4P2; NUDT4	nudix hydrolase 4 pseudogene 1; nudix hydrolase 4 pseudogene 2; nudix hydrolase 4
0.75	NUDT4; NUDT4P1	nudix hydrolase 4; nudix hydrolase 4 pseudogene 1
0.75	KCTD19	potassium channel tetramerization domain containing 19
0.75	APOA1	apolipoprotein A-I
0.75	KIF11	kinesin family member 11
0.75	CST9	cystatin 9 (testatin)

0.75	ZNF551	zinc finger protein 551
0.75	KIF27	kinesin family member 27
0.75	OSBPL5	oxysterol binding protein-like 5
0.75	PEG3; ZIM2	paternally expressed 3; zinc finger. imprinted 2
0.75	LHPP	phospholysine phosphohistidine inorganic pyrophosphate phosphatase
0.75	NCAPD3	non-SMC condensin II complex subunit D3
0.75	FAM71B	family with sequence similarity 71. member B
0.75	ODF3L2	outer dense fiber of sperm tails 3-like 2
0.75	SMC6	structural maintenance of chromosomes 6
0.75	LINC01588	long intergenic non-protein coding RNA 1588
0.75	CKB	creatine kinase. brain
0.75	CACNA1F	calcium channel. voltage-dependent. L type. alpha 1F subunit
0.75	MBD6	methyl-CpG binding domain protein 6
0.75	OR5M11	olfactory receptor. family 5. subfamily M. member 11
0.75	KCNC3	potassium channel. voltage gated Shaw related subfamily C. member 3
0.75	PPIB	Jeck2013 ALT_ACCEPTOR. ALT_DONOR. coding. INTERNAL. intronic. OVERLAPTX. OVEXON best transcript NM_000942
0.75	PRKACB	protein kinase. cAMP-dependent. catalytic. beta
0.75	COX8A	cytochrome c oxidase subunit VIIIA (ubiquitous)
0.75	PECR	peroxisomal trans-2-enoyl-CoA reductase
0.75	TSPYL1	TSPY-like 1
0.75	CLDN6	claudin 6
0.75	OR4A15	olfactory receptor. family 4. subfamily A. member 15
0.75	CLPTM1	cleft lip and palate associated transmembrane protein 1
0.75	CNNM3	cyclin and CBS domain divalent metal cation transport mediator 3
0.75	ZNF792	zinc finger protein 792

0.75	C14orf142	chromosome 14 open reading frame 142
0.75	RP11-875O11.1; RHOBTB2	Salzman2013 ANTISENSE. CDS. coding. INTERNAL. intronic. OVCODE. OVERLAPTX. OVEXON. UTR3 best transcript NM_015178; novel transcript. antisense to RHOBTB2
0.75	SLC22A25	solute carrier family 22. member 25
0.75	ARSF	arylsulfatase F
0.75	HSD3B7	hydroxy-delta-5-steroid dehydrogenase. 3 beta- and steroid delta-isomerase 7
0.75	KRTAP29-1	keratin associated protein 29-1
0.75	ZNF75A	zinc finger protein 75a
0.75	PAFAH1B3	platelet-activating factor acetylhydrolase 1b. catalytic subunit 3 (29kDa)
0.75	ZNF529-AS1	ZNF529 antisense RNA 1
0.75	C17orf100	chromosome 17 open reading frame 100
0.75	ZNF578	zinc finger protein 578
0.75	FRMD8	FERM domain containing 8
0.75	FZD2	frizzled class receptor 2
0.75	C20orf202	chromosome 20 open reading frame 202
0.75	MCEE	methylmalonyl CoA epimerase
0.75	P4HA1	prolyl 4-hydroxylase. alpha polypeptide I
0.75	ZWILCH	zwilch kinetochore protein
0.75	LYSMD3	LysM. putative peptidoglycan-binding. domain containing 3
0.75	ZNF367	zinc finger protein 367
0.75	F10	coagulation factor X
0.75	RNFT2	ring finger protein. transmembrane 2
0.75	CCDC77	coiled-coil domain containing 77
0.75	ADH1A	alcohol dehydrogenase 1A (class I). alpha polypeptide
0.75	CNNM1	cyclin and CBS domain divalent metal cation transport mediator 1

0.75	ND3	NADH dehydrogenase. subunit 3 (complex I)
0.75	RABL2B	RAB. member of RAS oncogene family-like 2B
0.75	PPP1R16B	protein phosphatase 1. regulatory subunit 16B
0.75	ENTPD7	ectonucleoside triphosphate diphosphohydrolase 7
0.75	ANKRD34A	ankyrin repeat domain 34A
0.75	ABCA7	ATP binding cassette subfamily A member 7
0.75	CETN2	centrin 2
0.75	AMICA1	adhesion molecule. interacts with CXADR antigen 1
0.75	MRPL41	mitochondrial ribosomal protein L41
0.75	LDHD	lactate dehydrogenase D
0.75	FAHD1	fumarylacetoacetate hydrolase domain containing 1
0.75	KRTAP12-2	keratin associated protein 12-2
0.75	SLC29A4	solute carrier family 29 (equilibrative nucleoside transporter). member 4
0.75	CYP2D6	cytochrome P450. family 2. subfamily D. polypeptide 6
0.75	LYL1	Memczak2013 ANTISENSE. coding. INTERNAL. UTR5 best transcript NM_005583
0.75	GPS1	G protein pathway suppressor 1
0.75	FAM20C	family with sequence similarity 20. member C
0.75	RPA2	replication protein A2
0.75	FABP1	fatty acid binding protein 1. liver
0.75	CNN1	calponin 1. basic. smooth muscle
0.75	MOS	v-mos Moloney murine sarcoma viral oncogene homolog
0.75	CCDC134	coiled-coil domain containing 134
0.75	CRMP1	Salzman2013 ANTISENSE. CDS. coding. INTERNAL. intronic. OVCODE. OVERLAPTX. OVEXON best transcript NM_001014809
0.75	RRBP1	Zhang2013 ALT_ACCEPTOR. ALT_DONOR. coding. INTERNAL. intronic best transcript NM_001042576

0.75	SERPINH1	serpin peptidase inhibitor. clade H (heat shock protein 47). member 1. (collagen binding protein 1)
0.75	BCAS3	breast carcinoma amplified sequence 3
0.75	KNSTRN	kinetochore-localized astrin/SPAG5 binding protein
0.75	NDUFB11	NADH dehydrogenase (ubiquinone) 1 beta subcomplex. 11. 17.3kDa
0.75	LBR	lamin B receptor
0.75	ENPP7	ectonucleotide pyrophosphatase/phosphodiesterase 7
0.75	TRIM4	tripartite motif containing 4
0.75	FHOD1	formin homology 2 domain containing 1
0.75	PARP6	poly(ADP-ribose) polymerase family member 6
0.75	HLA-DMA	major histocompatibility complex. class II. DM alpha
0.75	DOK4	docking protein 4
0.75	MOGAT2	monoacylglycerol O-acyltransferase 2
0.75	MXRA8	matrix-remodelling associated 8
0.75	MYBPC3	myosin binding protein C. cardiac
0.75	EFEMP2	EGF containing fibulin-like extracellular matrix protein 2
0.75	MAMDC4	MAM domain containing 4
0.75	C16orf71	chromosome 16 open reading frame 71
0.75	CEP44	centrosomal protein 44kDa
0.75	TSSK3	testis-specific serine kinase 3
0.75	CTIF; MIR4743	CBP80/20-dependent translation initiation factor; microRNA 4743
0.75	MFSD11	major facilitator superfamily domain containing 11
0.75	MGAT2	mannosyl (alpha-1.6-)-glycoprotein beta-1.2-N-acetylglucosaminyltransferase
0.75	COQ2	coenzyme Q2 4-hydroxybenzoate polyprenyltransferase
0.75	RIIAD1	regulatory subunit of type II PKA R-subunit (RIIa) domain containing 1

0.75	LOC389831; TCONS_00030034; TCONS_00030035; AC011043.1	uncharacterized LOC389831; Salzman2013 ANNOTATED. CDS. coding. OVCODE. OVEXON. UTR3 best transcript NM_001242480; Salzman2013 ANNOTATED. CDS. coding. OVCODE. OVEXON. UTR3. UTR5 best transcript NM_001242480; Salzman2013 ANNOTATED. ncRNA. OVEXON best transcript TCONS_00030034; Salzman2013 ANNOTATED. ncRNA. OVCODE. OVEXON best transcript TCONS_00030034; Salzman2013 ALT_ACCEPTOR. ncRNA. OVCODE. OVEXON. upstream_start best transcript TCONS_00030034; Salzman2013 ALT_DONOR. downstream_end. ncRNA. OVEXON best transcript TCONS_00030035; Salzman2013 ALT_DONOR. coding. intronic. OVEXON. UTR5 best transcript NM_001242480; uncharacterized protein LOC389831 [Source:RefSeq peptide;Acc:NP_001229409]
0.75	KREMEN1	kringle containing transmembrane protein 1
0.75	NAGPA	N-acetylglucosamine-1-phosphodiester alpha-N-acetylglucosaminidase
0.75	IDH1	isocitrate dehydrogenase 1 (NADP+)
0.75	GPX7	glutathione peroxidase 7
0.75	NAPEPLD	N-acyl phosphatidylethanolamine phospholipase D
0.75	PRIMPOL	primase and DNA directed polymerase
0.75	HECTD3	HECT domain containing E3 ubiquitin protein ligase 3
0.75	PLOD1	procollagen-lysine. 2-oxoglutarate 5-dioxygenase 1
0.75	JUND	jun D proto-oncogene
0.75	LINC01521	long intergenic non-protein coding RNA 1521
0.75	NDUFV3	Transcript Identified by AceView. Entrez Gene ID(s) 4731
0.75	CCDC144NL	coiled-coil domain containing 144 family. N-terminal like
0.75	CDK12	Transcript Identified by AceView. Entrez Gene ID(s) 51755
0.75	MYBPC1	myosin binding protein C. slow type
0.75	ACP2	acid phosphatase 2. lysosomal
0.75	PGM5	phosphoglucomutase 5
0.75	NEIL3	nei-like DNA glycosylase 3
0.75	TMEM91	transmembrane protein 91

0.75	ENDOV	endonuclease V
0.75	TBC1D5	TBC1 domain family. member 5
0.75	MARCH8	Transcript Identified by AceView. Entrez Gene ID(s) 220972
0.75	ATP5G1	ATP synthase. H+ transporting. mitochondrial Fo complex subunit C1 (subunit 9)
0.75	CDH9	cadherin 9. type 2 (T1-cadherin)
0.75	NCAPD2	non-SMC condensin I complex subunit D2
0.75	CHD3	chromodomain helicase DNA binding protein 3
0.75	FAM155A	family with sequence similarity 155. member A
0.75	NEK2	NIMA-related kinase 2
0.75	ZNF584	zinc finger protein 584
0.75	CRISPLD1	cysteine-rich secretory protein LCCL domain containing 1
0.75	FANCA	Fanconi anemia complementation group A
0.74	PCOLCE-AS1	PCOLCE antisense RNA 1
0.74	DLG4	discs. large homolog 4 (Drosophila)
0.74	PLA2G12A	Memczak2013 ANTISENSE. CDS. coding. INTERNAL best transcript NM_030821
0.74	KLHL3	Transcript Identified by AceView. Entrez Gene ID(s) 26249
0.74	GNAI2	guanine nucleotide binding protein (G protein). alpha inhibiting activity polypeptide 2
0.74	RAD51AP1	RAD51 associated protein 1
0.74	DVL2	dishevelled segment polarity protein 2
0.74	LYNX1	Ly6/neurotoxin 1
0.74	DDX11	Zhang2013 ALT_ACCEPTOR. ALT_DONOR. coding. INTERNAL. intronic best transcript NM_030653
0.74	MLXIPL	MLX interacting protein-like
0.74	LIG1	ligase I. DNA. ATP-dependent

0.74	OTX1	orthodenticle homeobox 1
0.74	OR7C2	olfactory receptor. family 7. subfamily C. member 2
0.74	SLC22A14	solute carrier family 22. member 14
0.74	ZKSCAN7	zinc finger with KRAB and SCAN domains 7
0.74	LPAR5	lysophosphatidic acid receptor 5
0.74	FZD8; MIR4683	frizzled class receptor 8; microRNA 4683
0.74	PKDCC	protein kinase domain containing. cytoplasmic
0.74	C1orf167	chromosome 1 open reading frame 167
0.74	SPSB2	splA/ryanodine receptor domain and SOCS box containing 2
0.74	TUBG2	tubulin. gamma 2
0.74	HIST2H2AC	histone cluster 2. H2ac
0.74	NHSL1	NHS-like 1
0.74	RUNDC3A	RUN domain containing 3A
0.74	HLA-B	major histocompatibility complex. class I. B
0.74	ZDHHC11B	zinc finger. DHHC-type containing 11B
0.74	TEX22	testis expressed 22
0.74	TM2D3	TM2 domain containing 3
0.74	STARD9	StAR-related lipid transfer domain containing 9
0.74	KCTD11	potassium channel tetramerization domain containing 11
0.74	LOC100133315; RP11-849H4.2	transient receptor potential cation channel. subfamily C. member 2-like; Salzman2013 ANNOTATED. INTERNAL. ncRNA. OVEXON best transcript NR_029192; Transcript Identified by AceView. Entrez Gene ID(s) 100133315. RefSeq ID(s) NR_029192; Transcript Identified by AceView. Entrez Gene ID(s) 100133315; Putative short transient receptor potential channel 2-like protein [Source:UniProtKB/Swiss-Prot;Acc:Q6ZNB5]
0.74	APBA3	amyloid beta (A4) precursor protein-binding. family A. member 3
0.74	SAP130	Sin3A associated protein 130kDa
0.74	SOCS1	suppressor of cytokine signaling 1

0.74	SOX5	SRY box 5
0.74	OXER1	oxoeicosanoid (OXE) receptor 1
0.74	CBY1	chibby homolog 1 (Drosophila)
0.74	TTC6	tetratricopeptide repeat domain 6
0.74	RILPL2	Rab interacting lysosomal protein-like 2
0.74	POLR2J3	polymerase (RNA) II (DNA directed) polypeptide J3
0.74	THOC3	THO complex 3
0.74	ZNF850	zinc finger protein 850
0.74	PRODH2	proline dehydrogenase (oxidase) 2
0.74	PSME2; MIR7703	proteasome activator subunit 2; microRNA 7703
0.74	ZC3H4	zinc finger CCCH-type containing 4
0.74	HNF4A	hepatocyte nuclear factor 4. alpha
0.74	HFE	hemochromatosis
0.74	MTRNR2L2; MTRNR2L12	MT-RNR2-like 2; MT-RNR2-like 12
0.74	CLN3; NPIPB7	ceroid-lipofuscinosis. neuronal 3; nuclear pore complex interacting protein family. member B7
0.74	PLD3	phospholipase D family. member 3
0.74	MRPL17	mitochondrial ribosomal protein L17
0.74	CIART	circadian associated repressor of transcription
0.74	PPFIBP2	PTPRF interacting protein. binding protein 2 (liprin beta 2)
0.74	ANGPTL4	angiopoietin like 4
0.74	OTUB1	OTU deubiquitinase. ubiquitin aldehyde binding 1
0.74	CTB-75G16.3; MRM1	Transcript Identified by AceView. Entrez Gene ID(s) 79922; novel transcript
0.74	SIVA1	SIVA1. apoptosis-inducing factor
0.74	LRRC28	Transcript Identified by AceView. Entrez Gene ID(s) 123355
0.74	PPAP2B	Transcript Identified by AceView. Entrez Gene ID(s) 8613

0.74	SPZ1	spermatogenic leucine zipper 1
0.74	KCNK3	potassium channel. two pore domain subfamily K. member 3
0.74	XPA	xeroderma pigmentosum. complementation group A
0.74	CCL2	chemokine (C-C motif) ligand 2
0.74	SMIM10	small integral membrane protein 10
0.74	AKT1S1	AKT1 substrate 1 (proline rich)
0.74	ANKRD37	ankyrin repeat domain 37
0.74	C21orf2	chromosome 21 open reading frame 2
0.74	LCA5	Leber congenital amaurosis 5
0.74	KCNIP2	Kv channel interacting protein 2
0.74	PAX5	paired box 5
0.74	RNF215	ring finger protein 215
0.74	ZMYND12	zinc finger. MYND-type containing 12
0.74	PKHD1	polycystic kidney and hepatic disease 1 (autosomal recessive)
0.74	AVPR1A	arginine vasopressin receptor 1A
0.74	NR5A2	nuclear receptor subfamily 5. group A. member 2
0.74	PDZK1	PDZ domain containing 1
0.74	CHCHD5	coiled-coil-helix-coiled-coil-helix domain containing 5
0.74	TMEM97	transmembrane protein 97
0.74	PPFIA3	protein tyrosine phosphatase. receptor type. f polypeptide (PTPRF). interacting protein (liprin). alpha 3
0.74	TMEM72	transmembrane protein 72
0.74	PAX2	paired box 2
0.74	RNF212	ring finger protein 212
0.74	MELK	maternal embryonic leucine zipper kinase
0.74	RFX3	Memczak2013 ALT_ACCEPTOR. ALT_DONOR. coding. INTERNAL. intronic best transcript NM_002919

0.74	SLC25A44	solute carrier family 25. member 44
0.74	G6PC3	glucose 6 phosphatase. catalytic. 3
0.74	LYRM2	LYR motif containing 2
0.74	FAM193B	family with sequence similarity 193. member B
0.74	L3HYPDH	L-3-hydroxyproline dehydratase (trans-)
0.74	TMEM63B	transmembrane protein 63B
0.74	STXBP4	syntaxin binding protein 4
0.74	HEYL	hes-related family bHLH transcription factor with YRPW motif-like
0.74	ACD	adrenocortical dysplasia homolog (mouse)
0.74	TGM3	transglutaminase 3
0.74	SIGLEC7	sialic acid binding Ig-like lectin 7
0.74	TACR1	tachykinin receptor 1
0.74	MYO7A	myosin VIIA
0.74	LOC339803; AC016747.3	uncharacterized LOC339803; Transcript Identified by AceView. Entrez Gene ID(s) 339803; novel transcript
0.74	FUOM	fucose mutarotase
0.74	SELENBP1	selenium binding protein 1
0.74	ACKR2	atypical chemokine receptor 2
0.74	SOX12	SRY box 12
0.74	SOX5	Transcript Identified by AceView. Entrez Gene ID(s) 6660
0.74	DHFR; DHFRP1	dihydrofolate reductase; dihydrofolate reductase pseudogene 1
0.74	FOXD4L3	forkhead box D4-like 3
0.74	KIAA1161	KIAA1161
0.74	TRIM50	tripartite motif containing 50
0.74	ANPEP	alanyl (membrane) aminopeptidase
0.74	ALDH2	aldehyde dehydrogenase 2 family (mitochondrial)

0.74	BNIP3	BCL2/adenovirus E1B 19kDa interacting protein 3
0.74	AGBL2	ATP/GTP binding protein-like 2
0.74	ZNF208	zinc finger protein 208
0.74	TGM4	transglutaminase 4
0.74	STMN1; MIR3917	stathmin 1; microRNA 3917
0.74	C10orf10	chromosome 10 open reading frame 10
0.74	EBP	emopamil binding protein (sterol isomerase)
0.74	SEZ6L2	seizure related 6 homolog (mouse)-like 2
0.74	PTPN18	protein tyrosine phosphatase. non-receptor type 18 (brain-derived)
0.74	SPDYC	speedy/RINGO cell cycle regulator family member C
0.74	PLCXD1	phosphatidylinositol-specific phospholipase C. X domain containing 1
0.74	ERICH6	glutamate rich 6
0.74	MYBL1	v-myb avian myeloblastosis viral oncogene homolog-like 1
0.74	CUBN	cubilin (intrinsic factor-cobalamin receptor)
0.74	TRA2A	transformer 2 alpha homolog (Drosophila)
0.74	SLC2A4	solute carrier family 2 (facilitated glucose transporter). member 4
0.74	DYRK1B	dual specificity tyrosine-(Y)-phosphorylation regulated kinase 1B
0.74	FTCD	formimidoyltransferase cyclodeaminase
0.74	KLHDC10	kelch domain containing 10
0.74	KIAA1024	KIAA1024
0.74	OR51I2	olfactory receptor. family 51. subfamily I. member 2
0.74	EFCAB11	EF-hand calcium binding domain 11
0.74	PHF23	PHD finger protein 23
0.74	FAM213A	family with sequence similarity 213. member A
0.74	HIST1H2BJ	histone cluster 1. H2bj
0.74	HIST2H2BF	histone cluster 2. H2bf

0.74	ITPR2	inositol 1.4.5-trisphosphate receptor. type 2
0.74	MFSD8	major facilitator superfamily domain containing 8
0.74	MOSPD3	motile sperm domain containing 3
0.74	LOC403312; AP000708.1	putative uncharacterized protein MGC39545; novel transcript. antisense to FEZ1
0.74	ZNF821	zinc finger protein 821
0.74	TMEM37	transmembrane protein 37
0.74	IFFO1	intermediate filament family orphan 1
0.74	CDT1	chromatin licensing and DNA replication factor 1
0.74	ZMIZ1	Zhang2013 ALT_ACCEPTOR. ALT_DONOR. coding. INTERNAL. intronic best transcript NM_020338
0.74	TNNC1	troponin C type 1 (slow)
0.74	HIBCH	Transcript Identified by AceView. Entrez Gene ID(s) 26275
0.74	SSNA1	Sjogren syndrome nuclear autoantigen 1
0.74	TBC1D3L; TBC1D3E	TBC1 domain family. member 3L; TBC1 domain family. member 3E
0.74	COL18A1	collagen. type XVIII. alpha 1
0.74	LRP2BP	LRP2 binding protein
0.74	IRAK4	interleukin 1 receptor associated kinase 4
0.74	FAM163A	family with sequence similarity 163. member A
0.74	CRIP3	cysteine-rich protein 3
0.74	MASP2	mannan-binding lectin serine peptidase 2
0.74	LRRC28	leucine rich repeat containing 28
0.74	NEU1	sialidase 1 (lysosomal sialidase)
0.74	ALDH7A1	aldehyde dehydrogenase 7 family. member A1
0.74	PIP4K2B	phosphatidylinositol-5-phosphate 4-kinase. type II. beta
0.74	GLUL	glutamate-ammonia ligase
0.74	FBLN1	fibulin 1

0.74	TMEM108	transmembrane protein 108
0.74	FKRP	fukutin related protein
0.73	PICK1	protein interacting with PRKCA 1
0.73	SHCBP1	SHC SH2-domain binding protein 1
0.73	FGFR1	fibroblast growth factor receptor 1
0.73	GATS	GATS. stromal antigen 3 opposite strand
0.73	FGD1	FYVE. RhoGEF and PH domain containing 1
0.73	A1CF	APOBEC1 complementation factor
0.73	TMEM150B	transmembrane protein 150B
0.73	DCAF7	DDB1 and CUL4 associated factor 7
0.73	CCDC51	coiled-coil domain containing 51
0.73	ZBTB40	zinc finger and BTB domain containing 40
0.73	CRYL1	crystallin lambda 1
0.73	TTC6	tetratricopeptide repeat domain 6
0.73	NIT1	nitrilase 1
0.73	PIFO	primary cilia formation
0.73	HIST1H1C	Memczak2013 ANTISENSE. CDS. coding. INTERNAL best transcript NM_005319
0.73	PPP1R3E	protein phosphatase 1. regulatory subunit 3E
0.73	RAB40A	RAB40A. member RAS oncogene family
0.73	TRIM22	Memczak2013 ANTISENSE. CDS. coding. INTERNAL. UTR5 best transcript NM_001199573
0.73	SIN3B	SIN3 transcription regulator family member B
0.73	ZNF462	zinc finger protein 462
0.73	FSTL4	follistatin-like 4
0.73	KLHL36	kelch-like family member 36
0.73	SC5D	sterol-C5-desaturase

0.73	MAPKBP1	mitogen-activated protein kinase binding protein 1
0.73	COQ4	coenzyme Q4
0.73	FXN	frataxin
0.73	C14orf28	chromosome 14 open reading frame 28
0.73	GPRIN2	G protein regulated inducer of neurite outgrowth 2
0.73	COL5A3	collagen. type V. alpha 3
0.73	NTNG2	netrin G2
0.73	MEIKIN	meiotic kinetochore factor
0.73	RFX7	regulatory factor X. 7
0.73	DMGDH	dimethylglycine dehydrogenase
0.73	TOP2A	topoisomerase (DNA) II alpha
0.73	POLD3	polymerase (DNA-directed). delta 3. accessory subunit
0.73	FAM107B	family with sequence similarity 107. member B
0.73	PRIM2	primase. DNA. polypeptide 2 (58kDa)
0.73	OR4C15	olfactory receptor. family 4. subfamily C. member 15
0.73	TUBB8	tubulin. beta 8 class VIII
0.73	DNASE1	deoxyribonuclease I
0.73	TWIST2	twist family bHLH transcription factor 2
0.73	MGME1	mitochondrial genome maintenance exonuclease 1
0.73	IFT80	intraflagellar transport 80
0.73	CHRAC1	chromatin accessibility complex 1
0.73	FAM157B	family with sequence similarity 157. member B
0.73	DTL	denticleless E3 ubiquitin protein ligase homolog (Drosophila)
0.73	GOLGA4	golgin A4
0.73	ACP5	acid phosphatase 5. tartrate resistant
0.73	MYEF2	myelin expression factor 2

0.73	SLC10A6	solute carrier family 10 (sodium/bile acid cotransporter). member 6
0.73	HIST2H2AA4; HIST2H2AA3	histone cluster 2. H2aa4; histone cluster 2. H2aa3
0.73	PDE7B	phosphodiesterase 7B
0.73	CRY2	cryptochrome circadian clock 2
0.73	ZNF846	zinc finger protein 846
0.73	DDIAS	DNA damage-induced apoptosis suppressor
0.73	ANKRD19P	ankyrin repeat domain 19. pseudogene
0.73	TBC1D24	TBC1 domain family. member 24
0.73	GPI	glucose-6-phosphate isomerase
0.73	MCCC2	methylcrotonoyl-CoA carboxylase 2
0.73	ZNF418	zinc finger protein 418
0.73	KDM4C	Transcript Identified by AceView. Entrez Gene ID(s) 23081
0.73	SMARCAL1	SWI/SNF related. matrix associated. actin dependent regulator of chromatin. subfamily a-like 1
0.73	TMEM175	transmembrane protein 175
0.73	GABRQ	gamma-aminobutyric acid (GABA) A receptor. theta
0.73	RABL3	RAB. member of RAS oncogene family-like 3
0.73	NHLH1	nescient helix-loop-helix 1
0.73	ZNF407	zinc finger protein 407
0.73	LOC728554; THOC3	THO complex 3 pseudogene; Homo sapiens THO complex 3. mRNA (cDNA clone MGC:5469 IMAGE:3451612). complete cds.; Homo sapiens THO complex 3. mRNA (cDNA clone MGC:87233 IMAGE:5262880). complete cds.; Salzman2013 ANNOTATED. INTERNAL. ncRNA. OVERLAPTX. OVEXON best transcript NR_003615; Transcript Identified by AceView. Entrez Gene ID(s) 728554
0.73	FAM161B	family with sequence similarity 161. member B
0.73	CEP78	centrosomal protein 78kDa
0.73	PSMB8-AS1	PSMB8 antisense RNA 1 (head to head)

0.73	ZIC4	Zic family member 4
0.73	MAGED2	MAGE family member D2
0.73	HS3ST3A1	heparan sulfate (glucosamine) 3-O-sulfotransferase 3A1
0.73	RFC3	replication factor C subunit 3
0.73	SLC5A9	solute carrier family 5 (sodium/sugar cotransporter). member 9
0.73	BRCA1	breast cancer 1. early onset
0.73	RNASE13	ribonuclease. RNase A family. 13 (non-active)
0.73	RIN2	Transcript Identified by AceView. Entrez Gene ID(s) 54453
0.73	ADCK5	aarF domain containing kinase 5
0.73	LOC100506388; RP11-1260E13.4; hosaru	uncharacterized LOC100506388; Transcript Identified by AceView; novel transcript. atisense to RPH3AL
0.73	KIF25	kinesin family member 25
0.73	APOL2	apolipoprotein L. 2
0.73	ARRDC1	arrestin domain containing 1
0.73	BAZ2B	bromodomain adjacent to zinc finger domain 2B
0.73	C14orf1	chromosome 14 open reading frame 1
0.73	PRC1	protein regulator of cytokinesis 1
0.73	CUTA	cutA divalent cation tolerance homolog (E. coli)
0.73	CCNB1	cyclin B1
0.73	EPPK1	epiplakin 1
0.73	POLR2J4	polymerase (RNA) II (DNA directed) polypeptide J4. pseudogene
0.73	SCN11A	sodium channel. voltage gated. type XI alpha subunit
0.73	ABHD15	abhydrolase domain containing 15
0.73	BTN3A3	butyrophilin. subfamily 3. member A3
0.73	HIST1H2BE	histone cluster 1. H2be
0.73	DTYMK	deoxythymidylate kinase

0.73	UCP2	uncoupling protein 2 (mitochondrial. proton carrier)
0.73	CNOT3	CCR4-NOT transcription complex subunit 3
0.73	BHMT	betaine--homocysteine S-methyltransferase
0.73	HIBCH	3-hydroxyisobutyryl-CoA hydrolase
0.73	SDHAF3	succinate dehydrogenase complex assembly factor 3
0.73	TNNC2	troponin C type 2 (fast)
0.73	CHTF8	Jeck2013 ANTISENSE. coding. INTERNAL. OVEXON. UTR3 best transcript NM_001039690
0.73	UNC5CL	unc-5 family C-terminal like
0.73	DTX4	deltex 4. E3 ubiquitin ligase
0.73	ZBTB49	zinc finger and BTB domain containing 49
0.73	RNASEH2A	ribonuclease H2. subunit A
0.73	FAM172A; POU5F2; MIR2277	family with sequence similarity 172. member A; POU domain class 5. transcription factor 2; microRNA 2277
0.73	ZNF43	zinc finger protein 43
0.73	C2orf54	chromosome 2 open reading frame 54
0.73	ND6	NADH dehydrogenase. subunit 6 (complex I)
0.73	STK35	serine/threonine kinase 35
0.73	RRP12	Zhang2013 ALT_ACCEPTOR. ALT_DONOR. coding. INTERNAL. intronic best transcript NM_015179
0.73	PDZD7	PDZ domain containing 7
0.73	CITED1	Cbp/p300-interacting transactivator. with Glu/Asp rich carboxy-terminal domain. 1
0.73	CEP162	centrosomal protein 162kDa
0.73	NLGN3	neuroligin 3
0.73	CAPN12	calpain 12
0.73	PIK3C2B	phosphatidylinositol-4-phosphate 3-kinase. catalytic subunit type 2 beta

0.73	CCDC30	coiled-coil domain containing 30
0.73	CTAG2	cancer/testis antigen 2
0.73	SPON2	spondin 2. extracellular matrix protein
0.73	SSSCA1-AS1	SSSCA1 antisense RNA 1 (head to head)
0.73	RAB4B; MIA-RAB4B; RAB4B-EGLN2	RAB4B. member RAS oncogene family; MIA-RAB4B readthrough (NMD candidate); RAB4B-EGLN2 readthrough (NMD candidate)
0.73	XKR5	X-linked Kx blood group related 5
0.73	ATF7IP2	activating transcription factor 7 interacting protein 2
0.73	PTGR2	prostaglandin reductase 2
0.73	FDPS	farnesyl diphosphate synthase
0.73	ENOX1	ecto-NOX disulfide-thiol exchanger 1
0.73	OR8G1	olfactory receptor. family 8. subfamily G. member 1 (gene/pseudogene)
0.73	SGSM3	small G protein signaling modulator 3
0.73	WDR62	WD repeat domain 62
0.73	PIP5K1B	Transcript Identified by AceView. Entrez Gene ID(s) 8395
0.73	KANK1	KN motif and ankyrin repeat domains 1
0.73	MAEA	macrophage erythroblast attacher
0.73	TRIM39	tripartite motif containing 39
0.73	SBK2	SH3 domain binding kinase family. member 2
0.73	EFCAB5	EF-hand calcium binding domain 5
0.73	CXorf57	chromosome X open reading frame 57
0.73	PGRMC1	progesterone receptor membrane component 1
0.73	RITA1	RBPJ interacting and tubulin associated 1
0.73	MSRB1	methionine sulfoxide reductase B1
0.73	RBP4	retinol binding protein 4. plasma
0.73	RSPH6A	radial spoke head 6 homolog A (Chlamydomonas)

0.73	CHMP2A	charged multivesicular body protein 2A
0.73	FIGNL1	fidgetin-like 1
0.73	HEXIM2	hexamethylene bis-acetamide inducible 2
0.73	AASS	aminoadipate-semialdehyde synthase
0.73	DUX1	double homeobox 1
0.73	CEP72	centrosomal protein 72kDa
0.73	HAUS5	HAUS augmin like complex subunit 5
0.72	NPPA	natriuretic peptide A
0.72	ETNPPL	ethanolamine-phosphate phospho-lyase
0.72	DCUN1D2	DCN1. defective in cullin neddylation 1. domain containing 2
0.72	TIAF1; MYO18A	TGFB1-induced anti-apoptotic factor 1; myosin XVIIIA
0.72	ALLC	allantoicase
0.72	NFKBIA	nuclear factor of kappa light polypeptide gene enhancer in B-cells inhibitor. alpha
0.72	IQCH	IQ motif containing H
0.72	TPX2	TPX2. microtubule-associated
0.72	G6PD	glucose-6-phosphate dehydrogenase
0.72	ZNF432	zinc finger protein 432
0.72	GSTT2	glutathione S-transferase theta 2 (gene/pseudogene)
0.72	SHISA4	shisa family member 4
0.72	CORIN	corin. serine peptidase
0.72	DQX1	DEAQ box RNA-dependent ATPase 1
0.72	ACRBP	acrosin binding protein
0.72	NQO2	NAD(P)H dehydrogenase. quinone 2
0.72	CLU; MIR6843	clusterin; microRNA 6843
0.72	PIRT	phosphoinositide-interacting regulator of transient receptor potential channels

0.72	ZNF724P	zinc finger protein 724. pseudogene
0.72	CELSR3; MIR4793	cadherin. EGF LAG seven-pass G-type receptor 3; microRNA 4793
0.72	IL17RD	interleukin 17 receptor D
0.72	NDC80	NDC80 kinetochore complex component
0.72	TSPYL2	TSPY-like 2
0.72	ZNF608	zinc finger protein 608
0.72	CD83	CD83 molecule
0.72	RACGAP1	Rac GTPase activating protein 1
0.72	ADPRHL1	ADP-ribosylhydrolase like 1
0.72	DISP2	dispatched homolog 2 (Drosophila)
0.72	MPP7	membrane protein. palmitoylated 7
0.72	SMIM1	small integral membrane protein 1 (Vel blood group)
0.72	EPHX1	epoxide hydrolase 1. microsomal (xenobiotic)
0.72	CTSA	cathepsin A
0.72	HMGCR	3-hydroxy-3-methylglutaryl-CoA reductase
0.72	WDR81	WD repeat domain 81
0.72	ADAMTS2	ADAM metallopeptidase with thrombospondin type 1 motif 2
0.72	NUDT7	nudix hydrolase 7
0.72	FAM149B1	family with sequence similarity 149. member B1
0.72	LGSN	lengsin. lens protein with glutamine synthetase domain
0.72	ID2	inhibitor of DNA binding 2. dominant negative helix-loop-helix protein
0.72	ADAMTS10	ADAM metallopeptidase with thrombospondin type 1 motif 10
0.72	ABCG5	ATP binding cassette subfamily G member 5
0.72	SOAT1	sterol O-acyltransferase 1
0.72	TMEM219	transmembrane protein 219
0.72	KIF4A	kinesin family member 4A

0.72	ITSN1	intersectin 1
0.72	ZNF248	zinc finger protein 248
0.72	CDC26	cell division cycle 26
0.72	PEBP1	phosphatidylethanolamine binding protein 1
0.72	VSIG10	V-set and immunoglobulin domain containing 10
0.72	ARHGAP26	Rho GTPase activating protein 26
0.72	PLCXD1	phosphatidylinositol-specific phospholipase C. X domain containing 1
0.72	CCDC87	coiled-coil domain containing 87
0.72	SESN3	sestrin 3
0.72	FAM156B; FAM156A	family with sequence similarity 156. member B; family with sequence similarity 156. member A
0.72	ZNF318	zinc finger protein 318
0.72	OCLM	Transcript Identified by AceView. Entrez Gene ID(s) 10896
0.72	SYT17	synaptotagmin XVII
0.72	CGA	glycoprotein hormones. alpha polypeptide
0.72	MST1L	macrophage stimulating 1-like
0.72	sept-06	septin 6
0.72	SP5	Sp5 transcription factor
0.72	CYB5D2	cytochrome b5 domain containing 2
0.72	REG3A	regenerating islet-derived 3 alpha
0.72	NEURL2	neuralized E3 ubiquitin protein ligase 2
0.72	PIK3R2; IFI30	phosphoinositide-3-kinase. regulatory subunit 2 (beta); interferon. gamma-inducible protein 30
0.72	DDR2	discoidin domain receptor tyrosine kinase 2
0.72	F11	coagulation factor XI
0.72	TNFSF13	tumor necrosis factor (ligand) superfamily. member 13
0.72	KHSRP; MIR3940	KH-type splicing regulatory protein; microRNA 3940

0.72	PDCL3	phosducin like 3
0.72	CDK5RAP3	CDK5 regulatory subunit associated protein 3
0.72	MAN2B2	mannosidase. alpha. class 2B. member 2
0.72	AURKB	aurora kinase B
0.72	DDTL; DDT	D-dopachrome tautomerase-like; D-dopachrome tautomerase
0.72	KMT5C	lysine (K)-specific methyltransferase 5C
0.72	POLR2G	polymerase (RNA) II (DNA directed) polypeptide G
0.72	SERPINF2	serpin peptidase inhibitor. clade F (alpha-2 antiplasmin. pigment epithelium derived factor). member 2
0.72	DEPDC5	DEP domain containing 5
0.72	TMEM132D	transmembrane protein 132D
0.72	FRZB	frizzled-related protein
0.72	APOL1	apolipoprotein L1
0.72	ECE1	endothelin converting enzyme 1
0.72	ATP2B2	ATPase. Ca++ transporting. plasma membrane 2
0.72	MCM5	minichromosome maintenance complex component 5
0.72	IFT57	intraflagellar transport 57
0.72	FNIP2	folliculin interacting protein 2
0.72	SLC38A3	solute carrier family 38. member 3
0.72	MIA2	melanoma inhibitory activity 2
0.72	UBE3B	ubiquitin protein ligase E3B
0.72	RNF123	ring finger protein 123
0.72	DMD	dystrophin
0.72	FGF4	fibroblast growth factor 4
0.72	HOPX	HOP homeobox
0.72	EHHADH	enoyl-CoA. hydratase/3-hydroxyacyl CoA dehydrogenase

0.72	SKP2	S-phase kinase-associated protein 2. E3 ubiquitin protein ligase
0.72	HIST1H3D; HIST1H2AD	histone cluster 1. H3d; histone cluster 1. H2ad
0.72	SCAND1	SCAN domain containing 1
0.72	SNX29	sorting nexin 29
0.72	ND2	MTND2
0.72	OSR1	odd-skipped related transciption factor 1
0.72	ZNF714; VN1R81P	zinc finger protein 714; vomeronasal 1 receptor 81 pseudogene
0.72	APBB2	Jeck2013 ALT_ACCEPTOR. ALT_DONOR. coding. INTERNAL. intronic best transcript NM_004307
0.72	PEX6	peroxisomal biogenesis factor 6
0.72	ATP1B4	ATPase. Na+/K+ transporting. beta 4 polypeptide
0.72	OR4S1	olfactory receptor. family 4. subfamily S. member 1
0.72	USP30	ubiquitin specific peptidase 30
0.72	GMNN	geminin. DNA replication inhibitor
0.72	RNF152	ring finger protein 152
0.72	TTC28	Jeck2013 ALT_ACCEPTOR. ALT_DONOR. coding. INTERNAL. intronic best transcript NM_001145418
0.72	AC208162.1	
0.72	ACAD10	acyl-CoA dehydrogenase family. member 10
0.72	UROD	uroporphyrinogen decarboxylase
0.72	BEX1	brain expressed X-linked 1
0.72	PMS2	PMS1 homolog 2. mismatch repair system component
0.72	APOA1BP	apolipoprotein A-I binding protein
0.72	PIGZ	phosphatidylinositol glycan anchor biosynthesis class Z
0.72	GLUD2	glutamate dehydrogenase 2
0.72	CABLES2	Cdk5 and Abl enzyme substrate 2
0.72	HRSP12	heat-responsive protein 12

0.72	CCDC18	coiled-coil domain containing 18
0.72	MORC4	MORC family CW-type zinc finger 4
0.72	CTBP2	C-terminal binding protein 2
0.72	CKS2	CDC28 protein kinase regulatory subunit 2
0.72	TCEANC	transcription elongation factor A (SII) N-terminal and central domain containing
0.72	DCLRE1B	DNA cross-link repair 1B
0.72	PI3	peptidase inhibitor 3. skin-derived
0.72	AFMID	arylformamidase
0.72	USF3	upstream transcription factor family member 3
0.71	XRCC2	X-ray repair complementing defective repair in Chinese hamster cells 2
0.71	LCAT	lecithin-cholesterol acyltransferase
0.71	EDEM2	ER degradation enhancer. mannosidase alpha-like 2
0.71	ACE2	angiotensin I converting enzyme 2
0.71	CHRNB1	cholinergic receptor. nicotinic beta 1
0.71	GGPS1	geranylgeranyl diphosphate synthase 1
0.71	GATA4	GATA binding protein 4
0.71	HCFC1R1	host cell factor C1 regulator 1 (XPO1 dependent)
0.71	BCKDHB	branched chain keto acid dehydrogenase E1. beta polypeptide
0.71	TOM1L2	target of myb1 like 2 membrane trafficking protein
0.71	SSTR1	somatostatin receptor 1
0.71	C1QTNF3	C1q and tumor necrosis factor related protein 3
0.71	AGR2	anterior gradient 2. protein disulphide isomerase family member
0.71	IQCG	IQ motif containing G
0.71	METTL21A	methyltransferase like 21A
0.71	MRE11A	MRE11 homolog A. double strand break repair nuclease

0.71	DGCR2; DGCR11	DiGeorge syndrome critical region gene 2; DiGeorge syndrome critical region gene 11 (non-protein coding)
0.71	CNNM2	cyclin and CBS domain divalent metal cation transport mediator 2
0.71	BTN2A2	butyrophilin. subfamily 2. member A2
0.71	GLRX5	glutaredoxin 5
0.71	CYB5D1	cytochrome b5 domain containing 1
0.71	MOB3C	MOB kinase activator 3C
0.71	ABHD17A	abhydrolase domain containing 17A
0.71	ALG14	ALG14. UDP-N-acetylglucosaminyltransferase subunit
0.71	TOPBP1	topoisomerase (DNA) II binding protein 1
0.71	LIN37	lin-37 DREAM MuvB core complex component
0.71	ACSL4	acyl-CoA synthetase long-chain family member 4
0.71	NEB	nebulin
0.71	NRBP2; MIR6845	nuclear receptor binding protein 2; microRNA 6845
0.71	CHST12	carbohydrate (chondroitin 4) sulfotransferase 12
0.71	AIF1	allograft inflammatory factor 1
0.71	IDH2	isocitrate dehydrogenase 2 (NADP+). mitochondrial
0.71	UBD	ubiquitin D
0.71	SPC25	SPC25. NDC80 kinetochore complex component
0.71	NUDT2	nudix hydrolase 2
0.71	HS3ST3B1	heparan sulfate (glucosamine) 3-O-sulfotransferase 3B1
0.71	PECAM1	platelet/endothelial cell adhesion molecule 1
0.71	TRAF7	TNF receptor-associated factor 7. E3 ubiquitin protein ligase
0.71	ZFX	zinc finger protein. X-linked
0.71	SSTR4	somatostatin receptor 4
0.71	GLB1L	galactosidase beta 1 like

0.71	MIF-AS1	MIF antisense RNA 1
0.71	LYPD1	LY6/PLAUR domain containing 1
0.71	PEG10	paternally expressed 10
0.71	MYH9	Jeck2013 ANTISENSE. CDS. coding. INTERNAL. intronic. OVCODE. OVEXON best transcript NM_002473
0.71	LRRC20	Transcript Identified by AceView. Entrez Gene ID(s) 55222
0.71	ACYP2	acylphosphatase 2. muscle type
0.71	FAM127B	family with sequence similarity 127. member B
0.71	SIRT7	sirtuin 7
0.71	SIL1	SIL1 nucleotide exchange factor
0.71	NDUFA12	NADH dehydrogenase (ubiquinone) 1 alpha subcomplex. 12
0.71	SLC6A5	solute carrier family 6 (neurotransmitter transporter). member 5
0.71	XAGE3	X antigen family. member 3
0.71	PDX1	pancreatic and duodenal homeobox 1
0.71	CAND1	cullin-associated and neddylation-dissociated 1
0.71	C8orf82; LRRC24	chromosome 8 open reading frame 82; leucine rich repeat containing 24
0.71	MASTL	microtubule associated serine/threonine kinase-like
0.71	THAP5	THAP domain containing 5
0.71	SLC35B3	solute carrier family 35 (adenosine 3-phospho 5-phosphosulfate transporter). member B3
0.71	OGFRL1	opioid growth factor receptor-like 1
0.71	PIGH	phosphatidylinositol glycan anchor biosynthesis class H
0.71	NOX1	NADPH oxidase 1
0.71	OXSM	3-oxoacyl-ACP synthase. mitochondrial
0.71	CDKL3	cyclin-dependent kinase-like 3
0.71	TMED3	transmembrane p24 trafficking protein 3
0.71	PDPR	pyruvate dehydrogenase phosphatase regulatory subunit

0.71	CHPF	chondroitin polymerizing factor
0.71	LILRB4	leukocyte immunoglobulin-like receptor. subfamily B (with TM and ITIM domains). member 4
0.71	GSTT2; GSTT2B	glutathione S-transferase theta 2 (gene/pseudogene); glutathione S-transferase theta 2B (gene/pseudogene)
0.71	NME7	NME/NM23 family member 7
0.71	ZFP14	ZFP14 zinc finger protein
0.71	AGFG2	ArfGAP with FG repeats 2
0.71	RNF144B	ring finger protein 144B
0.71	NAGLU	N-acetylglucosaminidase. alpha
0.71	KIF24	kinesin family member 24
0.71	STAB1	stabilin 1
0.71	C7	complement component 7
0.71	NSDHL	NAD(P) dependent steroid dehydrogenase-like
0.71	C6	complement component 6
0.71	MLST8	MTOR associated protein. LST8 homolog
0.71	ECT2	epithelial cell transforming 2
0.71	HPGD	hydroxyprostaglandin dehydrogenase 15-(NAD)
0.71	NUSAP1	nucleolar and spindle associated protein 1
0.71	CPN2	carboxypeptidase N. polypeptide 2
0.71	DBT	dihydrolipoamide branched chain transacylase E2
0.71	SMAGP	small cell adhesion glycoprotein
0.71	CREB3L2	cAMP responsive element binding protein 3-like 2
0.71	STAG3L2	stromal antigen 3-like 2 (pseudogene)
0.71	HACE1	HECT domain and ankyrin repeat containing E3 ubiquitin protein ligase 1
0.71	PHKG2	phosphorylase kinase. gamma 2 (testis)
0.71	ZNF141	Transcript Identified by AceView. Entrez Gene ID(s) 100288237; 7700

0.71	HS3ST4	heparan sulfate (glucosamine) 3-O-sulfotransferase 4
0.71	PTOV1; MIR4749	prostate tumor overexpressed 1; microRNA 4749
0.71	EMID1	EMI domain containing 1
0.71	DFNB59	deafness. autosomal recessive 59
0.71	ZNF169	zinc finger protein 169
0.71	HGFAC	HGF activator
0.71	NOXO1	NADPH oxidase organizer 1
0.71	NBL1	neuroblastoma 1. DAN family BMP antagonist
0.71	LZTFL1	leucine zipper transcription factor like 1
0.71	ZNF627	zinc finger protein 627
0.71	ZNF483	zinc finger protein 483
0.71	HTR1D	5-hydroxytryptamine (serotonin) receptor 1D. G protein-coupled
0.71	ZNF93	zinc finger protein 93
0.71	PRR11	proline rich 11
0.71	DDT	D-dopachrome tautomerase
0.71	PCP2	Purkinje cell protein 2
0.71	MPC2	mitochondrial pyruvate carrier 2
0.71	FKBPL	FK506 binding protein like
0.71	SARDH	sarcosine dehydrogenase
0.71	CYP19A1	cytochrome P450. family 19. subfamily A. polypeptide 1
0.71	SERF2	small EDRK-rich factor 2
0.71	ago-01	argonaute RISC catalytic component 1
0.70	CRACR2A	calcium release activated channel regulator 2A
0.70	CBX5; MIR3198-2	chromobox homolog 5; microRNA 3198-2
0.70	TECR; MIR639	trans-2.3-enoyl-CoA reductase; microRNA 639
0.70	ANGPTL6	angiopoietin like 6

0.70	IFITM10	interferon induced transmembrane protein 10
0.70	USP18	ubiquitin specific peptidase 18
0.70	ELMOD3	ELMO/CED-12 domain containing 3
0.70	KRT76	keratin 76. type II
0.70	GINS4	GINS complex subunit 4 (Sld5 homolog)
0.70	THNSL1	threonine synthase-like 1
0.70	BTD	biotinidase
0.70	KIAA1551	KIAA1551
0.70	FARP1	Jeck2013 ALT_ACCEPTOR. ALT_DONOR. coding. INTERNAL. intronic best transcript NM_005766
0.70	TOPORS-AS1; GVQW1	TOPORS antisense RNA 1; GVQW motif containing 1
0.70	UQCRQ	ubiquinol-cytochrome c reductase complex III subunit VII
0.70	DLGAP3	discs. large (Drosophila) homolog-associated protein 3
0.70	GTF2H2	general transcription factor IIH subunit 2
0.70	PTPRN	protein tyrosine phosphatase. receptor type. N
0.70	HSD11B1L; RPL36	hydroxysteroid (11-beta) dehydrogenase 1-like; ribosomal protein L36
0.70	PSMB9	proteasome subunit beta 9
0.70	USP41	ubiquitin specific peptidase 41
0.70	TP73	tumor protein p73
0.70	AC007566.10; GATAD1	Transcript Identified by AceView. Entrez Gene ID(s) 57798; novel transcript. antisense to ERVW-1 and PEX1
0.70	INSM2	insulinoma-associated 2
0.70	ARL17B; ARL17A	ADP-ribosylation factor like GTPase 17B; ADP-ribosylation factor like GTPase 17A
0.70	CLDN11	claudin 11
0.70	NCSTN	nicastrin
0.70	CCDC84	coiled-coil domain containing 84

0.70	MVB12A; BISPR	multivesicular body subunit 12A; BST2 interferon stimulated positive regulator (non-protein coding)
0.70	MPV17L2	MPV17 mitochondrial membrane protein-like 2
0.70	ZNF92	zinc finger protein 92
0.70	CAT	catalase
0.70	ESPN	espin
0.70	SLC25A29	solute carrier family 25 (mitochondrial carnitine/acylcarnitine carrier). member 29
0.70	HIST1H4D	histone cluster 1. H4d
0.70	CXCL1	chemokine (C-X-C motif) ligand 1 (melanoma growth stimulating activity. alpha)
0.70	PFKFB2	6-phosphofructo-2-kinase/fructose-2.6-biphosphatase 2
0.70	CYB561A3	cytochrome b561 family. member A3
0.70	TPCN2	two pore segment channel 2
0.70	SPACA5	sperm acrosome associated 5
0.70	D2HGDH	D-2-hydroxyglutarate dehydrogenase
0.70	CCDC69	coiled-coil domain containing 69
0.70	GLDC	glycine dehydrogenase (decarboxylating)
0.70	FCGBP	Fc fragment of IgG binding protein
0.70	DNAJC14	DnaJ (Hsp40) homolog. subfamily C. member 14
0.70	PPM1K	protein phosphatase. Mg2+/Mn2+ dependent. 1K
0.70	SMPDL3A	sphingomyelin phosphodiesterase. acid-like 3A
0.70	FADS1; MIR1908	fatty acid desaturase 1; microRNA 1908
0.70	NPM2	nucleophosmin/nucleoplasmin 2
0.70	TSGA10	testis specific 10
0.70	SULT1B1	sulfotransferase family 1B member 1
0.70	PDE1A	phosphodiesterase 1A. calmodulin-dependent

0.70	ZNF431; VN1R82P	zinc finger protein 431; vomeronasal 1 receptor 82 pseudogene
0.70	MYL5	myosin light chain 5
0.70	GSTM4	glutathione S-transferase mu 4
0.70	PLCB3	phospholipase C. beta 3 (phosphatidylinositol-specific)
0.70	C18orf54	chromosome 18 open reading frame 54
0.70	PCDHGC3; PCDHGA12; PCDHGB4; PCDHGA8; PCDHGA1; PCDHGA10; PCDHGA11; PCDHGA2; PCDHGA3; PCDHGA4; PCDHGA5; PCDHGA6; PCDHGA7; PCDHGA9; PCDHGB1; PCDHGB2; PCDHGB3; PCDHGB5; PCDHGB6; PCDHGB7; PCDHGC4; PCDHGC5	protocadherin gamma subfamily C. 3; protocadherin gamma subfamily A. 12; protocadherin gamma subfamily B. 4; protocadherin gamma subfamily A. 8; protocadherin gamma subfamily A. 1; protocadherin gamma subfamily A. 10; protocadherin gamma subfamily A. 11; protocadherin gamma subfamily A. 2; protocadherin gamma subfamily A. 3; protocadherin gamma subfamily A. 4; protocadherin gamma subfamily A. 5; protocadherin gamma subfamily A. 6; protocadherin gamma subfamily A. 7; protocadherin gamma subfamily A. 9; protocadherin gamma subfamily B. 1; protocadherin gamma subfamily B. 2; protocadherin gamma subfamily B. 3; protocadherin gamma subfamily B. 5; protocadherin gamma subfamily B. 6; protocadherin gamma subfamily B. 7; protocadherin gamma subfamily C. 4; protocadherin gamma subfamily C. 5
0.70	PSRC1	proline/serine-rich coiled-coil 1
0.70	CCNT2-AS1	CCNT2 antisense RNA 1
0.70	DCAKD	dephospho-CoA kinase domain containing
0.70	SYNJ1	synaptojanin 1
0.70	NET1	neuroepithelial cell transforming 1
0.70	ARL17B	ADP-ribosylation factor like GTPase 17B
0.70	CEP97	Transcript Identified by AceView. Entrez Gene ID(s) 79598
0.70	ZNF75D	zinc finger protein 75D
0.70	RP11-295G24.4; GFI1B	Transcript Identified by AceView. Entrez Gene ID(s) 8328; novel transcript
0.70	SLC7A9	solute carrier family 7 (amino acid transporter light chain. bo.+ system). member 9
0.70	DHFR	dihydrofolate reductase
0.70	RAB17	RAB17. member RAS oncogene family

0.70	MYLK3	myosin light chain kinase 3
0.70	MGST3	microsomal glutathione S-transferase 3
0.70	CYB5B	cytochrome b5 type B (outer mitochondrial membrane)
0.70	KIAA1841	KIAA1841
0.70	FAM151A	family with sequence similarity 151. member A
0.70	MST1	macrophage stimulating 1
0.70	PRRT1	proline-rich transmembrane protein 1
0.70	BORCS6	BLOC-1 related complex subunit 6
0.70	DCXR	dicarbonyl/L-xylulose reductase
0.70	GCNT6	glucosaminyl (N-acetyl) transferase 6 [Source:HGNC Symbol;Acc:HGNC:21623]
0.70	TMEM135	transmembrane protein 135
0.70	ZKSCAN7	zinc finger with KRAB and SCAN domains 7
0.70	APOA5	apolipoprotein A-V
0.70	RBP7	retinol binding protein 7. cellular
0.70	ZNF670; ZNF695; ZNF670-ZNF695	zinc finger protein 670; zinc finger protein 695; ZNF670-ZNF695 readthrough (NMD candidate)
0.70	OLFML3	olfactomedin like 3
0.70	OPCML	opioid binding protein/cell adhesion molecule-like
0.70	ZNF574	zinc finger protein 574
0.70	C11orf54	chromosome 11 open reading frame 54
0.70	SLC26A1	solute carrier family 26 (anion exchanger). member 1
0.70	SMTNL2	smoothelin-like 2
0.70	NKX1-2	NK1 homeobox 2
0.70	GCDH	glutaryl-CoA dehydrogenase
0.70	NAPA	N-ethylmaleimide-sensitive factor attachment protein. alpha
0.70	FAM111A	family with sequence similarity 111. member A

0.70	STRADA	STE20-related kinase adaptor alpha
0.69	MCM7	minichromosome maintenance complex component 7
0.69	NUP188	Memczak2013 ANTISENSE. coding. INTERNAL. UTR3 best transcript NM_015354
0.69	BUB1	BUB1 mitotic checkpoint serine/threonine kinase
0.69	DAG1	dystroglycan 1 (dystrophin-associated glycoprotein 1)
0.69	NUDT4	nudix hydrolase 4
0.69	CBR1	carbonyl reductase 1
0.69	CYP4F3	cytochrome P450. family 4. subfamily F. polypeptide 3
0.69	QPCTL	glutaminyl-peptide cyclotransferase-like
0.69	C5orf34	chromosome 5 open reading frame 34
0.69	ATXN2L	Transcript Identified by AceView. Entrez Gene ID(s) 11273
0.69	DPYSL2	dihydropyrimidinase-like 2
0.69	CDKN3	cyclin-dependent kinase inhibitor 3
0.69	BLOC1S3	biogenesis of lysosomal organelles complex-1. subunit 3
0.69	MPHOSPH10	M-phase phosphoprotein 10 (U3 small nucleolar ribonucleoprotein)
0.69	DAPK1	death-associated protein kinase 1
0.69	ACSM1	acyl-CoA synthetase medium-chain family member 1
0.69	TST	thiosulfate sulfurtransferase (rhodanese)
0.69	ZNF230	zinc finger protein 230
0.69	PXYLP1	2-phosphoxylose phosphatase 1
0.69	CHAD	chondroadherin
0.69	NLGN4Y	neuroligin 4. Y-linked
0.69	PAH	phenylalanine hydroxylase
0.69	VASN	vasorin
0.69	KIF16B	kinesin family member 16B

0.69	CLCN6	chloride channel. voltage-sensitive 6
0.69	ARMC5	armadillo repeat containing 5
0.69	C2orf15	chromosome 2 open reading frame 15
0.69	CD7	CD7 molecule
0.69	BLOC1S1-RDH5; RP11-644F5.10	BLOC1S1-RDH5 readthrough; Uncharacterized protein [Source:UniProtKB/TrEMBL;Acc:F8VNQ1]
0.69	SSBP2	single-stranded DNA binding protein 2
0.69	ATP7A	ATPase. Cu++ transporting. alpha polypeptide
0.69	NUDT16	nudix hydrolase 16
0.69	ADAMTS13	ADAM metallopeptidase with thrombospondin type 1 motif 13
0.69	ACOT2	acyl-CoA thioesterase 2
0.69	ZSCAN30	zinc finger and SCAN domain containing 30
0.69	LRP5L	LDL receptor related protein 5 like
0.69	TTPA	tocopherol (alpha) transfer protein
0.69	RASSF4	Ras association (RalGDS/AF-6) domain family member 4
0.69	DDX11	DEAD/H (Asp-Glu-Ala-Asp/His) box helicase 11
0.69	TSPYL4	TSPY-like 4
0.69	AKR1D1	aldo-keto reductase family 1. member D1
0.69	BPGM	2.3-bisphosphoglycerate mutase
0.69	UPRT	uracil phosphoribosyltransferase (FUR1) homolog (S. cerevisiae)
0.69	ERI2	ERI1 exoribonuclease family member 2
0.69	AP1B1	Zhang2013 ALT_ACCEPTOR. ALT_DONOR. coding. INTERNAL. intronic best transcript NM_001127
0.69	ABCB6	ATP binding cassette subfamily B member 6 (Langereis blood group)
0.69	TAF10	TAF10 RNA polymerase II. TATA box binding protein (TBP)-associated factor. 30kDa
0.69	NUMA1	nuclear mitotic apparatus protein 1

0.69	IDI1	isopentenyl-diphosphate delta isomerase 1
0.69	LIPA	lipase A. lysosomal acid. cholesterol esterase
0.69	HHEX	hematopoietically expressed homeobox
0.69	UPK3BL; POLR2J2	uroplakin 3B-like; polymerase (RNA) II (DNA directed) polypeptide J2
0.69	MOGAT1	monoacylglycerol O-acyltransferase 1
0.69	MOGS	mannosyl-oligosaccharide glucosidase
0.69	L3MBTL4	l(3)mbt-like 4 (Drosophila)
0.69	GRN	granulin
0.69	PLA2G6	phospholipase A2. group VI (cytosolic. calcium-independent)
0.69	TKFC	triokinase/FMN cyclase
0.69	SLC37A4	solute carrier family 37 (glucose-6-phosphate transporter). member 4
0.69	IFT74	intraflagellar transport 74
0.69	TCP10L2	t-complex 10-like 2
0.69	MVK	mevalonate kinase
0.69	ZNF720	zinc finger protein 720
0.69	CST3	cystatin C
0.69	DGKQ	diacylglycerol kinase theta
0.69	AMACR	alpha-methylacyl-CoA racemase
0.69	SLC26A2	solute carrier family 26 (anion exchanger). member 2
0.69	ZGRF1	zinc finger. GRF-type containing 1
0.69	EFNA4	ephrin-A4
0.69	ATP5D	ATP synthase. H+ transporting. mitochondrial F1 complex. delta subunit
0.69	ROGDI	rogdi homolog
0.69	RIPK4	receptor-interacting serine-threonine kinase 4
0.69	CACNG8; MIR935	calcium channel. voltage-dependent. gamma subunit 8; microRNA 935
0.69	PPDPF	pancreatic progenitor cell differentiation and proliferation factor

0.69	HSD17B7	hydroxysteroid (17-beta) dehydrogenase 7
0.69	CTRL	chymotrypsin-like
0.69	PLPPR1	phospholipid phosphatase related 1
0.69	FBXO5	F-box protein 5
0.69	ARHGAP17	Memczak2013 ALT_ACCEPTOR. ALT_DONOR. coding. INTERNAL. intronic best transcript NM_001006634
0.69	ICAM4	intercellular adhesion molecule 4 (Landsteiner-Wiener blood group)
0.69	PDF; COG8; TMED6	peptide deformylase (mitochondrial); component of oligomeric golgi complex 8; transmembrane p24 trafficking protein 6
0.69	PBLD	phenazine biosynthesis-like protein domain containing
0.69	KIF20B	kinesin family member 20B
0.69	SLC7A10	solute carrier family 7 (neutral amino acid transporter light chain. asc system). member 10
0.69	HTATIP2	HIV-1 Tat interactive protein 2
0.69	AMDHD2	amidohydrolase domain containing 2
0.69	PIH1D2	PIH1 domain containing 2
0.69	ACOT1	acyl-CoA thioesterase 1
0.69	CENPU	centromere protein U
0.69	PAPSS1	3-phosphoadenosine 5-phosphosulfate synthase 1
0.69	IFT172	intraflagellar transport 172
0.69	DCAF11	DDB1 and CUL4 associated factor 11
0.69	ZNF335	zinc finger protein 335
0.69	ACY1	aminoacylase 1
0.69	GSTM1	glutathione S-transferase mu 1
0.69	TFDP2	transcription factor Dp-2 (E2F dimerization partner 2)
0.69	CPT2	carnitine palmitoyltransferase 2
0.69	SCN2A	sodium channel. voltage gated. type II alpha subunit

0.68	SIRT2	sirtuin 2
0.68	SAG	S-antigen; retina and pineal gland (arrestin)
0.68	GAS2L3	growth arrest-specific 2 like 3
0.68	EARS2	glutamyl-tRNA synthetase 2. mitochondrial
0.68	ZNF678	zinc finger protein 678
0.68	OSBP2	oxysterol binding protein 2
0.68	PLCH1	phospholipase C. eta 1
0.68	HADH	hydroxyacyl-CoA dehydrogenase
0.68	CTSZ	cathepsin Z
0.68	GLYCTK	glycerate kinase
0.68	GMPPB	GDP-mannose pyrophosphorylase B
0.68	LIMS1	LIM and senescent cell antigen-like domains 1
0.68	ISX	intestine-specific homeobox
0.68	LYPD5	LY6/PLAUR domain containing 5
0.68	MID1	midline 1
0.68	CRIP1	cysteine-rich protein 1 (intestinal)
0.68	TPCN1	two pore segment channel 1
0.68	ZNF326	zinc finger protein 326
0.68	ZBTB1	zinc finger and BTB domain containing 1
0.68	RICTOR	Memczak2013 ANTISENSE. coding. INTERNAL. intronic best transcript NM_152756
0.68	LPCAT3	lysophosphatidylcholine acyltransferase 3
0.68	GPT	glutamic-pyruvate transaminase (alanine aminotransferase)
0.68	POLE	polymerase (DNA directed). epsilon. catalytic subunit
0.68	FES	FES proto-oncogene. tyrosine kinase
0.68	FCGBP	Fc fragment of IgG binding protein

0.68	SNCA	synuclein alpha
0.68	CIB3	calcium and integrin binding family member 3
0.68	BBX	Memczak2013 ANTISENSE. coding. INTERNAL. intronic best transcript NM_020235
0.68	TRPV6	transient receptor potential cation channel. subfamily V. member 6
0.68	MCM4	minichromosome maintenance complex component 4
0.68	MAFTRR	MAF transcriptional regulator RNA
0.68	TUBB2B	tubulin. beta 2B class IIb
0.68	OSGEPL1	O-sialoglycoprotein endopeptidase-like 1
0.68	FADS6	fatty acid desaturase 6
0.68	CENPA	centromere protein A
0.68	FLVCR1	feline leukemia virus subgroup C cellular receptor 1
0.68	F10	coagulation factor X
0.68	ABCA1	ATP binding cassette subfamily A member 1
0.68	FKBP2	FK506 binding protein 2
0.68	GATA6	GATA binding protein 6
0.68	NLRP10	NLR family. pyrin domain containing 10
0.68	POU2AF1	POU class 2 associating factor 1
0.68	CKAP2L	cytoskeleton associated protein 2-like
0.68	TECTB	tectorin beta
0.68	EN2	engrailed homeobox 2
0.68	NAGK	N-acetylglucosamine kinase
0.68	KCTD21-AS1	KCTD21 antisense RNA 1
0.68	SHF	Src homology 2 domain containing F
0.68	CYP2D6	cytochrome P450. family 2. subfamily D. polypeptide 6
0.68	MPND	MPN domain containing

0.68	REEP6	receptor accessory protein 6
0.68	MAP3K12	mitogen-activated protein kinase kinase kinase 12
0.68	ZNF257	zinc finger protein 257
0.68	HIP1	huntingtin interacting protein 1
0.68	RMI1	RecQ mediated genome instability 1
0.68	LGALS3BP	lectin. galactoside-binding. soluble. 3 binding protein
0.68	ARIH2OS	ariadne homolog 2 opposite strand
0.68	MARC1	mitochondrial amidoxime reducing component 1
0.68	TMPO	thymopoietin
0.68	MYBL2	v-myb avian myeloblastosis viral oncogene homolog-like 2
0.68	TMEM53	transmembrane protein 53
0.68	MPP5	membrane protein. palmitoylated 5
0.68	SUPT20HL1	SPT20 homolog. SAGA complex component-like 1
0.68	SLCO4C1	solute carrier organic anion transporter family. member 4C1
0.68	TDRKH	tudor and KH domain containing
0.68	POLDIP3	polymerase (DNA-directed). delta interacting protein 3
0.68	TUBA1A	tubulin. alpha 1a
0.68	PDZK1IP1	PDZK1 interacting protein 1
0.68	KBTBD4	kelch repeat and BTB (POZ) domain containing 4
0.68	TMEM187	transmembrane protein 187
0.68	SEPHS2	selenophosphate synthetase 2
0.68	BRCA2	breast cancer 2. early onset
0.68	XPNPEP1	Transcript Identified by AceView. Entrez Gene ID(s) 7511
0.68	ZNF486	zinc finger protein 486
0.68	SLC22A1	solute carrier family 22 (organic cation transporter). member 1
0.68	PLEKHH3	pleckstrin homology domain containing. family H (with MyTH4 domain)

		member 3
0.68	SLC9A3R1; MIR3615	solute carrier family 9. subfamily A (NHE3. cation proton antiporter 3). member 3 regulator 1; microRNA 3615
0.68	MXD4; MIR4800	MAX dimerization protein 4; microRNA 4800
0.68	IQSEC1	IQ motif and Sec7 domain 1
0.67	DGAT1; MIR6848	diacylglycerol O-acyltransferase 1; microRNA 6848
0.67	HJURP	Holliday junction recognition protein
0.67	DHRS2	dehydrogenase/reductase (SDR family) member 2
0.67	SLC2A4RG	SLC2A4 regulator
0.67	PRADC1	protease-associated domain containing 1
0.67	TOMM40L; MIR5187	translocase of outer mitochondrial membrane 40 homolog (yeast)-like; microRNA 5187
0.67	AK4	adenylate kinase 4
0.67	MEA1	male-enhanced antigen 1
0.67	EXOC3	Memczak2013 ANTISENSE. CDS. coding. INTERNAL best transcript NM_007277
0.67	AP5S1	adaptor-related protein complex 5. sigma 1 subunit
0.67	SPOCK1	sparc/osteonectin. cwcv and kazal-like domains proteoglycan (testican) 1
0.67	EXT1; hunera	Jeck2013 ALT_ACCEPTOR. ALT_DONOR. coding. INTERNAL. intronic best transcript NM_000127; Transcript Identified by AceView
0.67	NAALADL1	N-acetylated alpha-linked acidic dipeptidase-like 1
0.67	TXNL4B	thioredoxin-like 4B
0.67	C3orf70	chromosome 3 open reading frame 70
0.67	LYSMD4	LysM. putative peptidoglycan-binding. domain containing 4
0.67	NSMCE1	NSE1 homolog. SMC5-SMC6 complex component
0.67	APOC3	apolipoprotein C-III
0.67	ACSM3	acyl-CoA synthetase medium-chain family member 3

0.67	CLPSL2	colipase-like 2
0.67	MTFR2	mitochondrial fission regulator 2
0.67	FKBP7	FK506 binding protein 7
0.67	ZNF69	zinc finger protein 69
0.67	WWOX	WW domain containing oxidoreductase
0.67	RRM2	ribonucleotide reductase M2
0.67	GDF11	growth differentiation factor 11
0.67	ATP8B1	ATPase. aminophospholipid transporter. class I. type 8B. member 1
0.67	AXIN1	Memczak2013 ALT_ACCEPTOR. ALT_DONOR. coding. INTERNAL. intronic best transcript NM_003502
0.67	COTL1	coactosin-like F-actin binding protein 1
0.67	LIPC	lipase. hepatic
0.67	ALG1; NAGPA-AS1	ALG1. chitobiosyldiphosphodolichol beta-mannosyltransferase; NAGPA antisense RNA 1
0.67	LIPG	lipase. endothelial
0.67	RNF157	ring finger protein 157
0.67	CHST13	carbohydrate (chondroitin 4) sulfotransferase 13
0.67	GJB1	gap junction protein beta 1
0.67	MIS18A	MIS18 kinetochore protein A
0.67	HOXB6	homeobox B6
0.67	LYG2	lysozyme G-like 2
0.67	SLC25A34	solute carrier family 25. member 34
0.67	TOR3A	torsin family 3. member A
0.67	FAM72C	family with sequence similarity 72. member C
0.67	HIST1H2BB	histone cluster 1. H2bb
0.67	SUOX	sulfite oxidase
0.67	LRRC3	leucine rich repeat containing 3

0.67	LOC389834; AL354822.1	ankyrin repeat domain 57 pseudogene
0.67	KIF23	kinesin family member 23
0.67	KIAA1524	KIAA1524
0.67	SLC2A14	solute carrier family 2 (facilitated glucose transporter). member 14
0.67	KLHDC7A	kelch domain containing 7A
0.67	ERCC2	excision repair cross-complementation group 2
0.67	CNP	2.3-cyclic nucleotide 3 phosphodiesterase
0.67	GNMT	glycine N-methyltransferase
0.67	POTEJ	POTE ankyrin domain family. member J
0.67	SOX9	SRY box 9
0.67	PPARA	peroxisome proliferator-activated receptor alpha
0.67	UBE2C	ubiquitin-conjugating enzyme E2C
0.67	BET1L	Bet1 golgi vesicular membrane trafficking protein-like
0.67	KIF22	kinesin family member 22
0.67	ALAD	aminolevulinate dehydratase
0.67	ABCG8	ATP binding cassette subfamily G member 8
0.67	PSMB10	proteasome subunit beta 10
0.66	PCDH11X	protocadherin 11 X-linked
0.66	CD74	CD74 molecule. major histocompatibility complex. class II invariant chain
0.66	ACOX1	acyl-CoA oxidase 1. palmitoyl
0.66	TLCD1	TLC domain containing 1
0.66	KLC4	kinesin light chain 4
0.66	PEF1	penta-EF-hand domain containing 1
0.66	FAAP24	Fanconi anemia core complex associated protein 24
0.66	TRMT10A	tRNA methyltransferase 10A
0.66	TESK2	testis-specific kinase 2

0.66	CTSV	cathepsin V
0.66	PPIH	peptidylprolyl isomerase H (cyclophilin H)
0.66	COA5	cytochrome c oxidase assembly factor 5
0.66	SPACA5B	sperm acrosome associated 5B
0.66	MARCH3	membrane associated ring finger 3
0.66	SLC25A10	solute carrier family 25 (mitochondrial carrier; dicarboxylate transporter). member 10
0.66	GULP1	GULP. engulfment adaptor PTB domain containing 1
0.66	C2orf74	chromosome 2 open reading frame 74
0.66	KIFC1	kinesin family member C1
0.66	TPM1	tropomyosin 1 (alpha)
0.66	AURKAIP1	aurora kinase A interacting protein 1
0.66	AUNIP	aurora kinase A and ninein interacting protein
0.66	ALDH1B1	aldehyde dehydrogenase 1 family. member B1
0.66	ZNF519	zinc finger protein 519
0.66	PRIM1	primase. DNA. polypeptide 1 (49kDa)
0.66	TNFAIP8L1	tumor necrosis factor. alpha-induced protein 8-like 1
0.66	SHARPIN	SHANK-associated RH domain interactor
0.66	GATM	glycine amidinotransferase (L-arginine:glycine amidinotransferase)
0.66	NR2F2	nuclear receptor subfamily 2. group F. member 2
0.66	MTERF1	mitochondrial transcription termination factor 1
0.66	ST3GAL1	ST3 beta-galactoside alpha-2.3-sialyltransferase 1
0.66	SMC4	structural maintenance of chromosomes 4
0.66	PIEZO2	piezo-type mechanosensitive ion channel component 2
0.66	ETNK2	ethanolamine kinase 2
0.66	BDH1	3-hydroxybutyrate dehydrogenase. type 1

0.66	DEPDC7	DEP domain containing 7
0.66	C11orf24	chromosome 11 open reading frame 24
0.66	LOC81691; AC004381.6; U4atac	exonuclease NEF-sp; Putative RNA exonuclease NEF-sp [Source:UniProtKB/Swiss-Prot;Acc:Q96IC2]; Salzman2013 ANNOTATED. CDS. coding. OVCODE. OVERLAPTX. OVEXON. UTR3. UTR5 best transcript NM_001199053; Salzman2013 ANNOTATED. CDS. coding. INTERNAL. OVCODE. OVERLAPTX. OVEXON best transcript NM_001199053; Salzman2013 ANNOTATED. CDS. coding. OVCODE. OVERLAPTX. OVEXON. UTR3 best transcript NM_001199053; Transcript Identified by AceView. Entrez Gene ID(s) 81691; U4atac minor spliceosomal RNA [Source:RFAM;Acc:RF00618]
0.66	CENPM	centromere protein M
0.66	TUBB4B	tubulin. beta 4B class IVb
0.66	MTSS1	metastasis suppressor 1
0.66	STAC3	SH3 and cysteine rich domain 3
0.66	ZNF503	zinc finger protein 503
0.66	NDUFA10	NADH dehydrogenase (ubiquinone) 1 alpha subcomplex. 10. 42kDa
0.66	SH3YL1	SH3 and SYLF domain containing 1
0.66	PARK2	parkin RBR E3 ubiquitin protein ligase
0.66	MTRNR2L3	MT-RNR2-like 3
0.66	CDH11	cadherin 11. type 2. OB-cadherin (osteoblast)
0.66	MAPT	microtubule associated protein tau
0.66	TATDN2; GHRLOS	TatD DNase domain containing 2; ghrelin opposite strand/antisense RNA
0.66	MAGED1	MAGE family member D1
0.66	FAM72B	family with sequence similarity 72. member B
0.66	TRIM74	tripartite motif containing 74
0.66	TMEM107; SNORD118	transmembrane protein 107; small nucleolar RNA. C/D box 118
0.66	MAT1A	methionine adenosyltransferase I. alpha
0.66	EPHX2	epoxide hydrolase 2. cytoplasmic

0.66	AK7	adenylate kinase 7
0.66	HIST1H2BI	histone cluster 1. H2bi
0.66	HIST2H3D	histone cluster 2. H3d
0.66	HIST2H4B; HIST2H4A	histone cluster 2. H4b; histone cluster 2. H4a
0.66	CRELD2	cysteine rich with EGF-like domains 2
0.66	SMIM4	small integral membrane protein 4
0.66	NAP1L5	nucleosome assembly protein 1-like 5
0.66	HYKK	hydroxylysine kinase
0.66	KRTAP20-2	keratin associated protein 20-2
0.66	HIST1H2BK	histone cluster 1. H2bk
0.66	DGKD	diacylglycerol kinase. delta 130kDa
0.66	SMIM19	small integral membrane protein 19
0.66	NTN4	netrin 4
0.66	HDAC6	histone deacetylase 6
0.66	NDST1	N-deacetylase/N-sulfotransferase (heparan glucosaminyl) 1
0.66	LILRA5	Memczak2013 ANTISENSE. coding. INTERNAL. intronic best transcript NM_181985
0.66	IL17B	interleukin 17B
0.66	KCNN3	potassium channel. calcium activated intermediate/small conductance subfamily N alpha. member 3
0.66	FAM117A	family with sequence similarity 117. member A
0.66	SKA1	spindle and kinetochore associated complex subunit 1
0.65	LRP1	LDL receptor related protein 1
0.65	COG6	component of oligomeric golgi complex 6
0.65	ARHGEF26	Rho guanine nucleotide exchange factor 26
0.65	C1orf233	chromosome 1 open reading frame 233
0.65	SPICE1	spindle and centriole associated protein 1

0.65	UBXN8	UBX domain protein 8
0.65	SNAP29	synaptosome associated protein 29kDa
0.65	FUCA1	fucosidase. alpha-L- 1. tissue
0.65	HECTD2	HECT domain containing E3 ubiquitin protein ligase 2
0.65	RBP5	retinol binding protein 5. cellular
0.65	MRPL55	mitochondrial ribosomal protein L55
0.65	VAV3	vav 3 guanine nucleotide exchange factor
0.65	MVB12B	multivesicular body subunit 12B
0.65	HELLS	helicase. lymphoid-specific
0.65	KIF15	kinesin family member 15
0.65	WFDC8	WAP four-disulfide core domain 8
0.65	JUP	junction plakoglobin
0.65	MCM10	minichromosome maintenance 10 replication initiation factor
0.65	CHST9	carbohydrate (N-acetylgalactosamine 4-0) sulfotransferase 9
0.65	NFE2	nuclear factor. erythroid 2
0.65	GSTM2	glutathione S-transferase mu 2 (muscle)
0.65	ACSS3	acyl-CoA synthetase short-chain family member 3
0.65	LRRC23	leucine rich repeat containing 23
0.65	CKMT2	creatine kinase. mitochondrial 2 (sarcomeric)
0.65	CCDC125	coiled-coil domain containing 125
0.65	HSPG2	heparan sulfate proteoglycan 2
0.65	SNRNP25	small nuclear ribonucleoprotein. U11/U12 25kDa subunit
0.65	HIST1H3A	histone cluster 1. H3a
0.65	GREB1	growth regulation by estrogen in breast cancer 1
0.65	CPLX3	complexin 3
0.65	ZNF165	zinc finger protein 165

0.65	HAUS8	HAUS augmin like complex subunit 8
0.65	DBI	diazepam binding inhibitor (GABA receptor modulator. acyl-CoA binding protein)
0.65	TMEM45A	transmembrane protein 45A
0.65	SMAD9	SMAD family member 9
0.65	ASB9	ankyrin repeat and SOCS box containing 9
0.65	HSCB	HscB mitochondrial iron-sulfur cluster co-chaperone
0.65	GPX3	glutathione peroxidase 3
0.65	ZNF329	zinc finger protein 329
0.65	GEN1	GEN1 Holliday junction 5 flap endonuclease
0.65	DLX5	distal-less homeobox 5
0.65	SLC25A42	solute carrier family 25. member 42
0.65	LGMN	legumain
0.65	IFIT1	interferon-induced protein with tetratricopeptide repeats 1
0.65	FZD4	frizzled class receptor 4
0.65	ARRDC4	arrestin domain containing 4
0.65	AURKA	aurora kinase A
0.65	CDK2AP2	cyclin-dependent kinase 2 associated protein 2
0.65	PODNL1	podocan-like 1
0.65	CPZ; GPR78	carboxypeptidase Z; G protein-coupled receptor 78
0.65	RFNG	RFNG O-fucosylpeptide 3-beta-N-acetylglucosaminyltransferase
0.65	CDCA8	cell division cycle associated 8
0.65	FOXM1	forkhead box M1
0.65	CKS1B	CDC28 protein kinase regulatory subunit 1B
0.65	RAD51	RAD51 recombinase
0.65	IGDCC3	immunoglobulin superfamily. DCC subclass. member 3

0.65	KSR1	Zhang2013 ALT_ACCEPTOR. ALT_DONOR. coding. INTERNAL. intronic best transcript NM_014238
0.65	ARSA	arylsulfatase A
0.65	RPA3	replication protein A3
0.65	HMGB2	high mobility group box 2
0.65	PIK3R3	phosphoinositide-3-kinase. regulatory subunit 3 (gamma)
0.64	CCDC84	coiled-coil domain containing 84
0.64	LEPR; LEPROT	leptin receptor; leptin receptor overlapping transcript
0.64	ST8SIA5	ST8 alpha-N-acetyl-neuraminide alpha-2.8-sialyltransferase 5
0.64	STAT2	signal transducer and activator of transcription 2
0.64	FASN	fatty acid synthase
0.64	TEF	thyrotrophic embryonic factor
0.64	SLC2A6	solute carrier family 2 (facilitated glucose transporter). member 6
0.64	MCCC1	methylcrotonoyl-CoA carboxylase 1
0.64	SPHK2	sphingosine kinase 2
0.64	PLGLB2	plasminogen-like B2
0.64	TLX2	T-cell leukemia homeobox 2
0.64	ERI1	exoribonuclease 1
0.64	TACC3	transforming. acidic coiled-coil containing protein 3
0.64	FAM72A	family with sequence similarity 72. member A
0.64	KCNH2	potassium channel. voltage gated eag related subfamily H. member 2
0.64	FAM200B	family with sequence similarity 200. member B
0.64	BSN	bassoon presynaptic cytomatrix protein
0.64	CORO1A	coronin. actin binding protein. 1A
0.64	CHDH	choline dehydrogenase
0.64	SYP	synaptophysin

0.64	ZWINT	ZW10 interacting kinetochore protein
0.64	RCN3	reticulocalbin 3. EF-hand calcium binding domain
0.64	TMEM141	transmembrane protein 141
0.64	PCK1	phosphoenolpyruvate carboxykinase 1 (soluble)
0.64	KNTC1	kinetochore associated 1
0.64	SERPINA6	serpin peptidase inhibitor. clade A (alpha-1 antiproteinase. antitrypsin). member 6
0.64	BPIFB6	BPI fold containing family B. member 6
0.64	CSAD	cysteine sulfinic acid decarboxylase
0.64	PARD3B	par-3 family cell polarity regulator beta
0.64	SHMT1; MIR6778	serine hydroxymethyltransferase 1 (soluble); microRNA 6778
0.64	BAD	BCL2-associated agonist of cell death
0.64	TM7SF2	transmembrane 7 superfamily member 2
0.64	IL1RAP	interleukin 1 receptor accessory protein
0.64	C1orf105	chromosome 1 open reading frame 105
0.64	C1orf210	chromosome 1 open reading frame 210
0.64	EXO1	exonuclease 1
0.64	HIF3A	hypoxia inducible factor 3. alpha subunit
0.64	EPHA1	EPH receptor A1
0.64	HIST1H2BF	histone cluster 1. H2bf
0.64	FBXW11	F-box and WD repeat domain containing 11
0.64	TRMT2A	Memczak2013 ANTISENSE. CDS. coding. INTERNAL. UTR3 best transcript NM_022727
0.64	UPK3A	uroplakin 3A
0.64	RBP2	retinol binding protein 2. cellular
0.64	SREBF1	sterol regulatory element binding transcription factor 1
0.63	SLC37A4	solute carrier family 37 (glucose-6-phosphate transporter). member 4

		[Source:HGNC Symbol;Acc:HGNC:4061]
0.63	TMEM116	transmembrane protein 116
0.63	TRIP13	thyroid hormone receptor interactor 13
0.63	PFKFB1	6-phosphofructo-2-kinase/fructose-2.6-biphosphatase 1
0.63	LOC339059; RP5-1142A6.8	uncharacterized LOC339059; novel transcript. antisense to FAM38A
0.63	HIST1H2AJ	histone cluster 1. H2aj
0.63	HES6	hes family bHLH transcription factor 6
0.63	TTK	TTK protein kinase
0.63	MCM8	minichromosome maintenance 8 homologous recombination repair factor
0.63	HMGN3	high mobility group nucleosomal binding domain 3
0.63	SMPD3	sphingomyelin phosphodiesterase 3. neutral membrane (neutral sphingomyelinase II)
0.63	CCNF	cyclin F
0.63	CDC25B	cell division cycle 25B
0.63	SEC16B	SEC16 homolog B. endoplasmic reticulum export factor
0.63	CDC25A	cell division cycle 25A
0.63	MTMR4	myotubularin related protein 4
0.63	PDXDC2P	pyridoxal-dependent decarboxylase domain containing 2. pseudogene
0.63	EHD3	EH domain containing 3
0.63	NGEF	neuronal guanine nucleotide exchange factor
0.63	MAP2K6	mitogen-activated protein kinase kinase 6
0.63	CYB5A	cytochrome b5 type A (microsomal)
0.63	SLC34A3	solute carrier family 34 (type II sodium/phosphate cotransporter). member 3
0.63	NPC1L1	NPC1-like 1
0.63	SORBS1	sorbin and SH3 domain containing 1
0.63	KIAA1107	KIAA1107

0.63	FAR2P1	fatty acyl-CoA reductase 2 pseudogene 1
0.63	ZNF778	zinc finger protein 778
0.63	HINT2	histidine triad nucleotide binding protein 2
0.63	TCF19	transcription factor 19
0.63	DEPDC1	DEP domain containing 1
0.63	DNAH5	Transcript Identified by AceView. Entrez Gene ID(s) 1767
0.63	CDHR3	cadherin-related family member 3
0.63	CLDN19	claudin 19
0.63	ATP8B3	ATPase. aminophospholipid transporter. class I. type 8B. member 3
0.63	SPIRE2	spire-type actin nucleation factor 2
0.63	HIST1H2AL; HIST1H2BN	histone cluster 1. H2al; histone cluster 1. H2bn
0.63	FOXA1	forkhead box A1
0.63	TUBB1	tubulin. beta 1 class VI
0.63	BORCS7	BLOC-1 related complex subunit 7
0.63	HIST2H2AB	histone cluster 2. H2ab
0.63	GINS1	GINS complex subunit 1 (Psf1 homolog)
0.63	KANK4	KN motif and ankyrin repeat domains 4
0.63	CKMT1A	creatine kinase. mitochondrial 1A
0.63	SERPINA10	serpin peptidase inhibitor. clade A (alpha-1 antiproteinase. antitrypsin). member 10
0.63	PDK2	pyruvate dehydrogenase kinase. isozyme 2
0.63	C22orf46	chromosome 22 open reading frame 46
0.63	C1orf112	chromosome 1 open reading frame 112
0.63	SERPINA4	serpin peptidase inhibitor. clade A (alpha-1 antiproteinase. antitrypsin). member 4
0.63	CNBD2	cyclic nucleotide binding domain containing 2
0.63	DCDC2	doublecortin domain containing 2

0.63	LOC101927322; RP11-126O1.5	uncharacterized LOC101927322; novel transcript. antisense to MALT1
0.63	PGLYRP2	peptidoglycan recognition protein 2
0.63	GMCL1	germ cell-less. spermatogenesis associated 1
0.63	CEP70	centrosomal protein 70kDa
0.63	CALML4	calmodulin-like 4
0.63	ABAT	4-aminobutyrate aminotransferase
0.63	SWSAP1	SWIM-type zinc finger 7 associated protein 1
0.62	KLHL14	kelch-like family member 14
0.62	FANCI	Fanconi anemia complementation group I
0.62	RPGRIP1L	RPGRIP1-like
0.62	HIST1H3F	histone cluster 1. H3f
0.62	SUV39H1	suppressor of variegation 3-9 homolog 1 (Drosophila)
0.62	HOMEZ	homeobox and leucine zipper encoding
0.62	UPF3B	UPF3 regulator of nonsense transcripts homolog B (yeast)
0.62	SVBP	small vasohibin binding protein
0.62	COBLL1	cordon-bleu WH2 repeat protein like 1
0.62	ASF1B	anti-silencing function 1B histone chaperone
0.62	ADCK3	aarF domain containing kinase 3
0.62	MTRNR2L6	MT-RNR2-like 6
0.62	SWI5	SWI5 homologous recombination repair protein
0.62	TMEM86B	transmembrane protein 86B
0.62	RFC5	replication factor C subunit 5
0.62	TMEM238	transmembrane protein 238
0.62	C4orf46	chromosome 4 open reading frame 46
0.62	MFSD13A	major facilitator superfamily domain containing 13A
0.62	CDC6	cell division cycle 6

0.62	PPP1R3F	protein phosphatase 1. regulatory subunit 3F
0.62	TIMP3	TIMP metallopeptidase inhibitor 3
0.62	CCNA2	cyclin A2
0.62	CENPO	centromere protein O
0.62	STARD4	StAR-related lipid transfer domain containing 4
0.62	ONECUT2	one cut homeobox 2
0.62	GXYLT2	glucoside xylosyltransferase 2
0.62	MMAB	methylmalonic aciduria (cobalamin deficiency) cblB type
0.62	C15orf59	chromosome 15 open reading frame 59
0.62	PFKFB4; MIR6823	6-phosphofructo-2-kinase/fructose-2.6-biphosphatase 4; microRNA 6823
0.62	AKAP14	A kinase (PRKA) anchor protein 14
0.62	HSPB11	heat shock protein family B (small). member 11
0.62	IHH	indian hedgehog
0.62	PBK	PDZ binding kinase
0.62	CDKL5	cyclin-dependent kinase-like 5
0.62	CLSPN	claspin
0.61	SLC23A1	solute carrier family 23 (ascorbic acid transporter). member 1
0.61	TTR	transthyretin
0.61	ANAPC15	anaphase promoting complex subunit 15
0.61	INCENP	inner centromere protein
0.61	RDH16	retinol dehydrogenase 16 (all-trans)
0.61	STAG3L3; STAG3L2	stromal antigen 3-like 3 (pseudogene); stromal antigen 3-like 2 (pseudogene)
0.61	GYS1	glycogen synthase 1 (muscle)
0.61	ETFDH	electron-transferring-flavoprotein dehydrogenase
0.61	STAG3L1	stromal antigen 3-like 1 (pseudogene)
0.61	SCAMP5	secretory carrier membrane protein 5

0.61	H2AFX	H2A histone family. member X
0.61	ACSM3	acyl-CoA synthetase medium-chain family member 3
0.61	HIST1H2BL	histone cluster 1. H2bl
0.61	CERS4	ceramide synthase 4
0.61	BCAT2	branched chain amino-acid transaminase 2. mitochondrial
0.61	SALL2	spalt-like transcription factor 2
0.61	POTEI	POTE ankyrin domain family. member I
0.61	BLOC1S1	biogenesis of lysosomal organelles complex-1. subunit 1
0.61	TMPRSS2	transmembrane protease. serine 2
0.61	C2orf68	chromosome 2 open reading frame 68
0.61	HAAO	3-hydroxyanthranilate 3.4-dioxygenase
0.61	STIM2	stromal interaction molecule 2
0.61	ZNF682	zinc finger protein 682
0.61	HIST1H1E	histone cluster 1. H1e
0.61	DLEU2; MIR15A; MIR16-1; MIR3613	deleted in lymphocytic leukemia 2 (non-protein coding); microRNA 15a; microRNA 16-1; microRNA 3613
0.61	RP11-54C4.3; LOC100652758	uncharacterized protein LOC100652758 [Source:RefSeq peptide;Acc:NP_001265011]; uncharacterized LOC100652758; novel transcript
0.61	PIM2	Pim-2 proto-oncogene. serine/threonine kinase
0.61	SPG7	spastic paraplegia 7 (pure and complicated autosomal recessive)
0.61	COL2A1	collagen. type II. alpha 1
0.61	SLC2A6	solute carrier family 2 (facilitated glucose transporter). member 6 [Source:HGNC Symbol;Acc:HGNC:11011]
0.61	NCAPG	non-SMC condensin I complex subunit G
0.61	SLC2A3	solute carrier family 2 (facilitated glucose transporter). member 3
0.61	KLB	klotho beta

0.61	HMGCS2	3-hydroxy-3-methylglutaryl-CoA synthase 2 (mitochondrial)
0.61	CYP8B1	cytochrome P450. family 8. subfamily B. polypeptide 1
0.61	SLC38A4	solute carrier family 38. member 4
0.61	C11orf71	chromosome 11 open reading frame 71
0.61	SLC10A5	solute carrier family 10. member 5
0.61	SNX33	sorting nexin 33
0.60	FAM83D	family with sequence similarity 83. member D
0.60	NRM	nurim (nuclear envelope membrane protein)
0.60	CDH2	cadherin 2. type 1. N-cadherin (neuronal)
0.60	ATP6V0E2	ATPase. H+ transporting V0 subunit e2
0.60	SOX21	SRY box 21
0.60	CAPN3	calpain 3
0.60	FANCG	Fanconi anemia complementation group G
0.60	HIST1H2AB	histone cluster 1. H2ab
0.60	OPTC	opticin
0.60	NFATC2	nuclear factor of activated T-cells. cytoplasmic. calcineurin-dependent 2
0.60	UHRF1	ubiquitin-like with PHD and ring finger domains 1
0.60	ERGIC1	Memczak2013 ALT_ACCEPTOR. ALT_DONOR. coding. INTERNAL. intronic best transcript NM_001031711
0.60	DAP	death-associated protein
0.60	C1orf186	chromosome 1 open reading frame 186
0.60	PCSK9	proprotein convertase subtilisin/kexin type 9
0.60	NMNAT1	nicotinamide nucleotide adenylyltransferase 1
0.60	CCDC15	coiled-coil domain containing 15
0.60	C3orf35	chromosome 3 open reading frame 35
0.60	RAD54B; FSBP	RAD54 homolog B (S. cerevisiae); fibrinogen silencer binding protein

0.60	IGFL1	IGF like family member 1
0.60	LRRC38	leucine rich repeat containing 38
0.60	ACYP2	acylphosphatase 2. muscle type
0.60	ZNF107; MIR6839	zinc finger protein 107; microRNA 6839
0.60	PCYT2	phosphate cytidylyltransferase 2. ethanolamine
0.60	CTSE	cathepsin E
0.60	CFAP126	cilia and flagella associated protein 126
0.60	DZIP3	DAZ interacting zinc finger protein 3
0.60	SLC41A1	solute carrier family 41 (magnesium transporter). member 1
0.60	SLC46A1	solute carrier family 46 (folate transporter). member 1
0.60	CNPY4	canopy FGF signaling regulator 4
0.60	SEC61A2	Sec61 translocon alpha 2 subunit
0.60	B3GNT9	UDP-GlcNAc:betaGal beta-1.3-N-acetylglucosaminyltransferase 9
0.60	VTN	vitronectin
0.60	ARNTL	aryl hydrocarbon receptor nuclear translocator-like
0.60	PARPBP	PARP1 binding protein
0.60	NDUFAF6	NADH dehydrogenase (ubiquinone) complex I. assembly factor 6
0.59	FBLN5	fibulin 5
0.59	CA13	carbonic anhydrase XIII
0.59	CCDC142; MRPL53	coiled-coil domain containing 142; mitochondrial ribosomal protein L53
0.59	SEMA6C	sema domain. transmembrane domain (TM). and cytoplasmic domain. (semaphorin) 6C
0.59	HHLA3	HERV-H LTR-associating 3
0.59	PLA2G12B	phospholipase A2. group XIIB
0.59	THRSP	thyroid hormone responsive
0.59	MCOLN1	mucolipin 1

0.59	KIF15	kinesin family member 15
0.59	PLXNC1	plexin C1
0.59	KLHL13	kelch-like family member 13
0.59	RAD9B	RAD9 checkpoint clamp component B
0.59	MCM3AP-AS1	MCM3AP antisense RNA 1
0.59	CACNB4	calcium channel. voltage-dependent. beta 4 subunit
0.59	GOLGA8O	golgin A8 family. member O
0.59	ALKBH6	alkB homolog 6
0.59	CNTD1	cyclin N-terminal domain containing 1
0.59	KIAA1217	KIAA1217
0.59	C19orf44	chromosome 19 open reading frame 44
0.59	SERPINI1	serpin peptidase inhibitor. clade I (neuroserpin). member 1
0.59	ZNF76	zinc finger protein 76
0.59	C12orf4	chromosome 12 open reading frame 4
0.59	XRCC4	X-ray repair complementing defective repair in Chinese hamster cells 4
0.59	SCN1A	sodium channel. voltage gated. type I alpha subunit
0.59	CCBL1	cysteine conjugate-beta lyase. cytoplasmic
0.59	SLC2A6	solute carrier family 2 (facilitated glucose transporter). member 6 [Source:HGNC Symbol;Acc:HGNC:11011]
0.59	GATM	glycine amidinotransferase (L-arginine:glycine amidinotransferase)
0.59	RTKN2	rhotekin 2
0.59	GM2A	GM2 ganglioside activator
0.59	IGSF1	immunoglobulin superfamily. member 1
0.59	ACSM2B	acyl-CoA synthetase medium-chain family member 2B
0.59	CCDC159	coiled-coil domain containing 159
0.59	CDCA3	cell division cycle associated 3

0.58	MTRNR2L9	MT-RNR2-like 9
0.58	TMEM139	transmembrane protein 139
0.58	MKI67	marker of proliferation Ki-67
0.58	TRAIP	TRAF interacting protein
0.58	FEN1	flap structure-specific endonuclease 1
0.58	HSD17B6	hydroxysteroid (17-beta) dehydrogenase 6
0.58	NXT2	nuclear transport factor 2-like export factor 2
0.58	ACY3	aminoacylase 3
0.58	C17orf53	chromosome 17 open reading frame 53
0.58	MAF	v-maf avian musculoaponeurotic fibrosarcoma oncogene homolog
0.58	FYB	FYN binding protein
0.58	RAD54L	RAD54-like (S. cerevisiae)
0.58	CTGF	connective tissue growth factor
0.58	RALGPS1	Ral GEF with PH domain and SH3 binding motif 1
0.58	IL17RB	interleukin 17 receptor B
0.58	POLA2	polymerase (DNA directed). alpha 2. accessory subunit
0.58	DENND6B	DENN/MADD domain containing 6B
0.58	TRIM73	tripartite motif containing 73
0.57	CITED2	Cbp/p300-interacting transactivator. with Glu/Asp rich carboxy-terminal domain. 2
0.57	SLC17A1	solute carrier family 17 (organic anion transporter). member 1
0.57	SLC2A6	solute carrier family 2 (facilitated glucose transporter). member 6 [Source:HGNC Symbol;Acc:HGNC:11011]
0.57	PRELID3A	PRELI domain containing 3A
0.57	CROT	carnitine O-octanoyltransferase
0.57	KLF7	Kruppel-like factor 7 (ubiquitous)
0.57	NCAPH	non-SMC condensin I complex subunit H

0.57	HIST1H2AE	histone cluster 1. H2ae
0.57	CASC5	cancer susceptibility candidate 5
0.57	CKAP2	cytoskeleton associated protein 2
0.57	ABCC11	ATP binding cassette subfamily C member 11
0.57	CDC20	cell division cycle 20
0.57	PER3	period circadian clock 3
0.57	POTEE	POTE ankyrin domain family. member E
0.57	TK1	thymidine kinase 1. soluble
0.57	TMEM198	transmembrane protein 198
0.57	C1orf101	chromosome 1 open reading frame 101
0.57	COL27A1	collagen. type XXVII. alpha 1
0.56	IFIH1	interferon induced. with helicase C domain 1
0.56	HIST1H3C	histone cluster 1. H3c
0.56	ENPP2	ectonucleotide pyrophosphatase/phosphodiesterase 2
0.56	HIST1H2AM; HIST1H3J	histone cluster 1. H2am; histone cluster 1. H3j
0.56	C12orf60	chromosome 12 open reading frame 60
0.56	MYL9	myosin light chain 9
0.56	HIST1H2AI	histone cluster 1. H2ai
0.56	GBA	glucosidase. beta. acid
0.56	TIAM2	T-cell lymphoma invasion and metastasis 2
0.56	CNBD2	cyclic nucleotide binding domain containing 2
0.56	ANLN	anillin actin binding protein
0.56	SUSD2	sushi domain containing 2
0.56	LACTB2	lactamase. beta 2
0.56	ARL5A	ADP-ribosylation factor like GTPase 5A
0.56	TSPAN1	tetraspanin 1

0.56	CDCA5	cell division cycle associated 5
0.56	LOC730183; RP11-146F11.1; hitema	uncharacterized LOC730183; Transcript Identified by AceView; novel transcript. antisense to SRCAP
0.56	PKLR	pyruvate kinase. liver and RBC
0.56	HIST1H2BH	histone cluster 1. H2bh
0.56	KLKB1	kallikrein B1
0.56	SPATA25	spermatogenesis associated 25
0.56	TMEM106A	transmembrane protein 106A
0.56	FGD4	FYVE. RhoGEF and PH domain containing 4
0.56	ACSM5	acyl-CoA synthetase medium-chain family member 5
0.56	ALDH6A1	aldehyde dehydrogenase 6 family. member A1
0.56	EID1	EP300 interacting inhibitor of differentiation 1
0.55	SARM1	sterile alpha and TIR motif containing 1
0.55	SPDL1	spindle apparatus coiled-coil protein 1
0.55	KNDC1	kinase non-catalytic C-lobe domain (KIND) containing 1
0.55	CLDN2	claudin 2
0.55	PLG	plasminogen
0.55	CD47	CD47 molecule
0.55	HIST1H2BG	histone cluster 1. H2bg
0.55	TNFSF10	tumor necrosis factor (ligand) superfamily. member 10
0.55	DEPTOR	DEP domain containing MTOR-interacting protein
0.55	HTRA2	HtrA serine peptidase 2
0.55	DSN1	DSN1 homolog. MIS12 kinetochore complex component
0.55	KIF20A	kinesin family member 20A
0.55	GPR75	G protein-coupled receptor 75
0.55	FCGRT	Fc fragment of IgG. receptor. transporter. alpha

0.55	SAR1B	Transcript Identified by AceView. Entrez Gene ID(s) 51128
0.55	SMIM24	small integral membrane protein 24
0.55	ELMO1	engulfment and cell motility 1
0.55	KIF15	kinesin family member 15
0.54	LSS	lanosterol synthase (2.3-oxidosqualene-lanosterol cyclase)
0.54	UNG	uracil DNA glycosylase
0.54	RAB11FIP4; MIR4724	RAB11 family interacting protein 4 (class II); microRNA 4724
0.54	FSD1L	fibronectin type III and SPRY domain containing 1-like
0.54	PALMD	palmdelphin
0.54	CCDC64	coiled-coil domain containing 64
0.54	MVD	mevalonate (diphospho) decarboxylase
0.54	SECTM1	secreted and transmembrane 1
0.54	CREB3L3	cAMP responsive element binding protein 3-like 3
0.54	OTC	ornithine carbamoyltransferase
0.54	BBOF1	basal body orientation factor 1
0.54	NAA38	N(alpha)-acetyltransferase 38. NatC auxiliary subunit
0.54	GDF9	growth differentiation factor 9
0.54	PLGLB1; PLGLB2	plasminogen-like B1; plasminogen-like B2
0.54	DLGAP5	discs. large (Drosophila) homolog-associated protein 5
0.54	DDX12P; DDX11	DEAD/H (Asp-Glu-Ala-Asp/His) box polypeptide 12. pseudogene; DEAD/H (Asp-Glu-Ala-Asp/His) box helicase 11
0.54	SRY	sex determining region Y
0.54	DSE	dermatan sulfate epimerase
0.54	HIST1H2BO	histone cluster 1. H2bo
0.54	GPD1	glycerol-3-phosphate dehydrogenase 1
0.54	PRSS56	protease. serine. 56

0.54	PROM1	prominin 1
0.54	FBXO43	F-box protein 43
0.54	GCA	grancalcin. EF-hand calcium binding protein
0.53	HRG	histidine-rich glycoprotein
0.53	TAT	tyrosine aminotransferase
0.53	LMNB1	lamin B1
0.53	HIST1H2AK	histone cluster 1. H2ak
0.53	SYT11	synaptotagmin XI
0.53	KIF18A	kinesin family member 18A
0.53	UBA7; MIR5193	ubiquitin-like modifier activating enzyme 7; microRNA 5193
0.53	ERV3-1; ZNF117	endogenous retrovirus group 3. member 1; zinc finger protein 117
0.53	ZNF189	zinc finger protein 189
0.53	C12orf75	chromosome 12 open reading frame 75
0.53	SLX4	SLX4 structure-specific endonuclease subunit
0.53	EFCAB12	EF-hand calcium binding domain 12
0.53	FLJ45513; RP11-304F15.3	uncharacterized LOC729220; novel transcript antisense to TAC4
0.52	ZNF385B	zinc finger protein 385B
0.52	STIL	SCL/TAL1 interrupting locus
0.52	SLC25A1	solute carrier family 25 (mitochondrial carrier; citrate transporter). member 1
0.52	STARD5	StAR-related lipid transfer domain containing 5
0.52	CHRNE	cholinergic receptor. nicotinic epsilon
0.52	HIST1H3H	histone cluster 1. H3h
0.52	C1QTNF9B-AS1	C1QTNF9B antisense RNA 1
0.52	PCYT1B	phosphate cytidylyltransferase 1. choline. beta
0.51	CIT; MIR1178	citron rho-interacting serine/threonine kinase; microRNA 1178

0.51	C2orf76	chromosome 2 open reading frame 76
0.51	SPTLC3	serine palmitoyltransferase. long chain base subunit 3
0.51	SLC16A13	solute carrier family 16. member 13
0.50	ATG4A	autophagy related 4A. cysteine peptidase
0.50	PANK1	pantothenate kinase 1
0.50	CYP17A1	cytochrome P450. family 17. subfamily A. polypeptide 1
0.50	TNFAIP3	tumor necrosis factor. alpha-induced protein 3
0.50	CYB561D2	cytochrome b561 family. member D2
0.50	ROR1	receptor tyrosine kinase-like orphan receptor 1
0.50	FAM111B	family with sequence similarity 111. member B
0.50	CDCA2	cell division cycle associated 2
0.50	DIO1	deiodinase. iodothyronine. type I
0.50	HIST1H1B	histone cluster 1. H1b
0.50	HOOK2	hook microtubule-tethering protein 2
0.50	CYP7A1	cytochrome P450. family 7. subfamily A. polypeptide 1
0.50	CYP4F2	cytochrome P450. family 4. subfamily F. polypeptide 2
0.50	PNPLA3	patatin-like phospholipase domain containing 3
0.49	TSACC	TSSK6 activating co-chaperone
0.49	HIST1H4A	histone cluster 1. H4a
0.49	ANGPTL3	angiopoietin like 3
0.49	ADHFE1; C8orf46	alcohol dehydrogenase. iron containing 1; chromosome 8 open reading frame 46
0.49	GINS2	GINS complex subunit 2 (Psf2 homolog)
0.49	ARHGEF39	Rho guanine nucleotide exchange factor 39
0.49	POTEF	POTE ankyrin domain family. member F
0.49	HIST1H2AG	histone cluster 1. H2ag

0.49	ZNF341	zinc finger protein 341
0.49	NAT6	N-acetyltransferase 6 (GCN5-related)
0.48	FRY	FRY microtubule binding protein
0.48	SOX6; MIR6073	SRY box 6; microRNA 6073
0.48	SGOL2	shugoshin-like 2 (S. pombe)
0.48	MTHFR	methylenetetrahydrofolate reductase (NAD(P)H)
0.48	CD24	CD24 molecule
0.47	ITIH1	inter-alpha-trypsin inhibitor heavy chain 1
0.47	SCARA3	scavenger receptor class A. member 3
0.47	TMEM143	transmembrane protein 143
0.47	CDC25C	cell division cycle 25C
0.46	LEAP2	liver expressed antimicrobial peptide 2
0.46	ZNF565	zinc finger protein 565
0.46	E2F8	E2F transcription factor 8
0.46	CYP3A7; CYP3A7-CYP3A51P	cytochrome P450. family 3. subfamily A. polypeptide 7; CYP3A7-CYP3A51P readthrough
0.45	ESCO2	establishment of sister chromatid cohesion N-acetyltransferase 2
0.45	ODAM	odontogenic. ameloblast asssociated
0.45	C1orf116	chromosome 1 open reading frame 116
0.44	HIST2H3A	histone cluster 2. H3a
0.44	CHGB	chromogranin B
0.44	PRLR	prolactin receptor
0.44	HIST2H3A; HIST2H3C	histone cluster 2. H3a; histone cluster 2. H3c
0.44	CYP3A5	cytochrome P450. family 3. subfamily A. polypeptide 5
0.43	HIST1H2BM	histone cluster 1. H2bm
0.43	SNAI2	snail family zinc finger 2

0.43	PRRG2	proline rich Gla (G-carboxyglutamic acid) 2
0.42	SGOL1	shugoshin-like 1 (S. pombe)
0.42	SLC22A7	solute carrier family 22 (organic anion transporter). member 7
0.41	CYP3A43	cytochrome P450. family 3. subfamily A. polypeptide 43
0.41	LINC00612	long intergenic non-protein coding RNA 612
0.41	IFIT3	interferon-induced protein with tetratricopeptide repeats 3
0.40	HMMR	hyaluronan-mediated motility receptor (RHAMM)
0.39	CDH1	cadherin 1. type 1
0.39	HIST1H3B	histone cluster 1. H3b
0.38	AFM	afamin
0.36	DEPDC4	DEP domain containing 4
0.36	YPEL2	yippee like 2
0.32	G6PC	glucose-6-phosphatase. catalytic subunit
0.30	ANGPTL8	angiopoietin like 8
0.29	CENPI	centromere protein I
0.20	ALDOB	aldolase B. fructose-bisphosphate

References

1. Guo, X.; Fang, L.; Tan, Y. Solution-phase hierarchical self-organization of ultralong Se nanowires into diverse macroarchitectures and their enhanced field emission. *Phys. Chem. Chem. Phys.* **2014**, *17*, 2794–2803, doi:10.1039/C4CP04051A.
2. Saeed, M.; Ansari, M.T.; Kaleem, I.; Bajwa, S.Z.; Rehman, A.; Bano, K.; Tehseen, B.; Jamil, N.; Zahoor, M.; Shaheen, A.; et al. Assessment of Antimicrobial Features of Selenium Nanoparticles (SeNPs) Using Cyclic Voltammetric Strategy. *J. Nanosci. Nanotechnol.* **2019**, *19*, 7363–7368, doi:10.1166/jnn.2019.16627.
3. Chen, Y.-Z.; You, Y.-T.; Chen, P.-J.; Li, D.; Su, T.-Y.; Lee, L.; Shih, Y.-C.; Chen, C.-W.; Chang, C.-C.; Wang, Y.-C.; et al. Environmentally and Mechanically Stable Selenium 1D/2D Hybrid Structures for Broad-Range Photoresponse from Ultraviolet to Infrared Wavelengths. *ACS Appl. Mater. Interfaces* **2018**, *10*, 35477–35486, doi:10.1021/acsami.8b11676.
4. Coelho, D.; Luiz, G.M.; Machado, S.A. A photoelectrochemical methodology to obtain nanorods of crystalline hexagonal trigonal selenium. *J. Electroanal. Chem.* **2018**, *820*, 89–96, doi:10.1016/j.jelechem.2018.04.050.
5. Zhang, H.; Zhou, H.; Bai, J.; Li, Y.; Yang, J.; Ma, Q.; Qu, Y. Biosynthesis of selenium nanoparticles mediated by fungus Mariannaea sp. HJ and their characterization. *Colloids Surfaces A: Physicochem. Eng. Asp.* **2019**, *571*, 9–16, doi:10.1016/j.colsurfa.2019.02.070.
